# LncRNA-AC009948.5 promotes invasion and metastasis of lung adenocarcinoma by binding to miR-186-5p

**DOI:** 10.3389/fonc.2022.949951

**Published:** 2022-08-19

**Authors:** Jun Bai, Hongli Li, Xinlu Chen, Lin Chen, Yaqiong Hu, Lu Liu, Yanqiao Zhao, Wei Zuo, Baogang Zhang, Chonggao Yin

**Affiliations:** ^1^ Department of Pathology, Weifang Medical University, Weifang, China; ^2^ Experimental Center for Medicine Research, Weifang Medical University, Weifang, China; ^3^ School of Clinical Medicine, Weifang Medical University, Weifang, China; ^4^ College of Nursing, Weifang Medical University, Weifang, China

**Keywords:** LncRNA-AC009948.5, miR-186-5p, epithelial-mesenchymal transition, invasion and metastasis, lung adenocarcinoma

## Abstract

**Background:**

Long non-coding RNAs (LncRNAs) has been confirmed to play a crucial role in the development and progression of various cancer types. Here we evaluated the expression profiles of LncRNAs in Lung adenocarcinoma (LUAD) tissues and identified a novel LncRNA, termed LncRNA-AC009948.5. However, the role and potential molecular mechanisms of this novel LncRNA in LUAD carcinogenesis is unknown.

**Methods:**

Regarding the public databases and based on integrating bioinformatics analyses, we determined whether LncRNA-AC009948.5 exerts its oncogenic functions *via* sponging miR-186-5p in LUAD. Furthermore, we determined whether NCAPG2 was a downstream target of miR-186-5p. Moreover, the expression level and biological function of LncRNA-AC009948.5 in LUAD were determined by qRT-PCR, cell apoptosis, Edu, transwell, wound healing and western blot assays. Besides, xenograft mice were established for validation. We explored the expression of LncRNA-AC009948.5 and its roles in the prognosis of LUAD.

**Results:**

LncRNA expression microarray data indicate that LncRNA-AC009948.5 is upregulated in LUAD samples. The present study confirmed the upregulation of LncRNA-AC009948.5 in LUAD tissues and cells. Encreased expression of LncRNA-AC009948.5 was correlated with tumor size, lymph nodes, distant metastasis and histological grade, and poor prognosis.LncRNA-AC009948.5 knockdown significantly inhibited cell proliferation, migration, and invasion *in vitro*, as well as tumorigenesis and metastasis *in vivo*. Conversely, LncRNA-AC009948.5 upregulated had opposite effects. Mechanistically, we elucidated that LncRNA-AC009948.5 could directly bind to miR-186-5p and subsequently suppress expression of the target gene of NCAPG2.

**Conclusions:**

LncRNA-AC009948.5 promotes lung adenocarcinoma cells metastasis *via* the miR-186-5p/NCAPG2 axis and activation of the EMT process. Which may serve as potential targets for the treatment of LUAD in the future.

## Introduction

Lung cancer remains the leading cause of cancer-related deaths worldwide, Lung cancer is a malignant tumor with fast growth, high mortality, and poor prognosis (1, 2). Lung adenocarcinoma is the most common histologic type of non-small-cell lung cancercan and accounts for 44%–55.7% of all lung cancer cases (3). Despite the great progress in researches on lung cancer, the incidence of lung adenocarcinoma remains increasing every year. The 5-year survival rate of patients with lung cancer is less than 15% (4, 5).

Long-chain non-coding RNAs (LncRNAs), previously considered nonfunctional, have gradually become the focus of cancer research. Dong (6) found that LncRNA DGCR5 could promote the development of lung cancer by inhibiting miR-22-3p. Qin (7) reported that MIR31HG could enhance the proliferation of lung adenocarcinoma cells. Sun et al. (8) indicated that LncRNA-XIST was able to promote cisplatin resistance in lung adenocarcinoma by regulating let-7i. Some LncRNAs, such as LncRNA-CASC9.5 (9), LncRNA-OIP5-AS1 (10), and LncRNA-00707 (11), function as an oncogene in lung adenocarcinoma. In addition, studies have shown that, LncRNA HOXA11-AS acted as a ceRNA to promote cisplatin resistance of human LUAD cells via the miR-454-3p/Stat3 axis. Shohreh Teimuri (13) found that LncRNA-AC009948.5 played an important role in autoimmune diseases. However, it remains unclear whether LncRNA-AC009948.5 plays a role in lung adenocarcinoma.

This study validated the role of a new LncRNA of LncRNA-AC009948.5 and the underlying mechanisms in lung adenocarcinoma through *in vivo* and *in vitro* experiments. We confirm that LncRNA-AC009948.5 can promote the proliferation, invasion, and metastasis of lung adenocarcinoma and the occurrence of epithelial-mesenchymal transition (EMT) by targeting miR-186-5p. Our results could provide ideas and theoretical basis for further research on LncRNAs.

## Materials and methods

### Cells and cell culture

Normal human lung epithelial cells of BEAS-2B, along with four human lung adenocarcinoma cell lines of H1299, H226, A549 and H1975, and human embryonic kidney cells (HEK-293T) were purchased from the ATCC (Manassas, VA, USA). BEAS-2B cells were cultured with Dulbecco’s modified Eagle’s medium (DMEM) (Invitrogen, Carlsbad, CA, USA). Four cell lines were cultured in DMEM media, containing 10% fetal bovine serum (FBS) and penicillin-streptomycin. HEK-293T cells were cultured in DMEM (Hyclone) containing 10% FBS (Every Green, Zhejiang, China). All cells were cultured in an incubator (ATCC, Manassas, VA, USA) at 37°C under 5% CO_2_ atmosphere.

### RNA extraction and reverse-transcription and quantitative real-time PCR

TRIzol (Invitrogen, Carlsbad, CA, USA) reagent was used to extract total RNA from tissues or cells. cDNA was synthesized by an MMLV first-strand cDNA synthesis kit (Promega, WI, USA). SYBR Green quantitative PCR Kit (Bio-Rad. Hercules, CA, USA) was used to detect LncRNA-AC009948.5 expression in tissues and cells. U6 and GAPDH were used as internal references for normalizing target genes. The relative expression of target genes was calculated by the 2^-ΔΔCT^ method. The expression of target genes in normal lung tissues was set as 1 to standardize their levels in the lung adenocarcinoma ones. Primers used for qRT-PCR were as follows: LncRNA-AC009948.5, forward primer: 5′-AAAGCAGGAACGAGTAGCGG-3′, reverse primer: 5′-AAGGCAGCTCCTTTCAGACA-3′; miR-186-5p, RT primer: 5’-GTCGTATCCAGTGCAGGGTCCGAGGTATTCGCACTGGATACGACAGCCCA-3’ forward primer: 5’-CCGCGCGCAAAGAATTCTCCTTT-3’, reverse primer: 5’-ATCCAGTGCAGGGTCCGAGG-3’; NCAPG2 forward primer: 5’-CACCGCCTGCACCAACATAGC-3’, reverse primer: 5’-GTCCTCTTCCTCCTCGTCCTCTG-3’.

### Subcellular fractionation location

The separation of nuclear and cytosolic fractions was performed using a PARIS kit (Invitrogen, Life Technologies Co, CA, USA) according to the manufacturer’s instructions. For qRT-PCR, GAPDH and U6 were used as fractionation indicators.

### Luciferase reporter assay

Fluorescein-labeled reporter gene detection was carried out using a Dual-Luciferase Assay System kit (Promega) after sequence comparisons of LncRNA-AC009948.5, NCAPG2, and miR-186-5p, according to the manufacturer’s instructions. Wild-type and mutant GV272- LncRNA-AC009948.5 and GV272-NCAPG2-3’UTR dual-luciferase reporter vectors incorporating miRNA binding sites were constructed by GeneChem Company (Shanghai, China). HEK-293T cells were seeded on 12-well plates and cultured for 24 h, and then co-transfected with wild-type or mutant vectors with miRNA mimics using transfection reagents. The cell supernatant was washed twice with ice-cold buffer 24-48 h after cell transfection with Lipofectamine 2000 (Invitrogen, Carlsbad, CA, USA). Then cells were lysed and the lysate was used for the determination of luciferase (Luc2) activity. Renilla luciferase activity was used as an internal standard.

### RNA pull-down assay

LncRNA-AC009948.5 and negative control (NC) were biotin-labeled using Biotin RNA Labeling Mix (Roche, Basel, Switzerland) and T7/SP6 RNA polymerase (Roche) to be bio- LncRNA-AC009948.5 and bio-NC by GenePharma (Shanghai, China). Then, H1299 and A549 cells were mixed and incubated with biotinylated RNAs for 48 h. Next, cells were collected and incubated with streptavidin agarose beads (Invitrogen, USA) for 1 h at 37°C. After the beads were washed with buffer, bound RNAs were quantified and analyzed by qRT-PCR assays.

### Establishment of stably transfected cell lines

Overexpression lentiviral vector of GV358, containing human LncRNA-AC009948.5 and siRNA lentiviral vector GV248, along with overexpression lentiviral vector of GV235 containing human miR-186 and siRNA lentiviral vector GV232, were purchased from Genechem (Shanghai, China). Scrambled (Scr), SiAC009948.5, NC, overAC009948.5, con, miR-186, sh-con, sh-miR-186-5p, and SiAC009948.5+sh-miR-186-5p are corresponding to knockdown control, LncRNA-AC009948.5 knockdown, overexpression control, LncRNA-AC009948.5 overexpression, miR-186 overexpression control, miR-186 overexpression, miR-186-5p knockdown, was miR-186 knockdown, LncRNA-AC009948.5 knockdown and miR-186-5p knockdown co-infection groups, respectively.

For cell apoptosis experiment, the empty plasmids or AC009948.5 siRNA were transiently transfected into cells using Lipofectamine 2000 (Invitrogen) according to the manufacturer’s instructions.

### Cell apoptosis assay

To detect the apoptosis of infected cells, a FITC Annexin V Apoptosis Detection Kit II (BD Biosciences, USA) was used to stain cells. In brief, transfected cells were digested and incubated with propidium iodide and FITC, and then the cells were immediately analyzed by flow cytometry for early and late apoptosis. The experiments were performed independently three times.

### Cell proliferation experiment

After being digested with trypsinized, cells were suspended in a complete medium. Three wells for each group were seeded at 5×10^4^ cells/well and the cells were incubated at 37°C with 5% CO_2_. From the second day after plating, the cell counts were detected by Celigo Image Cytometer (Celigo, Nexcelom, USA) once a day for 5 days. At each time point, cell counts of the 3 wells in each group were presented as mean values. The cell growth curves of each group were plotted for 5 consecutive days. The infected cells were cultured in a 96-well plate for five days and photographed in the same field every day to evaluate cell proliferation. The results were analyzed by the Celigo system. EdU assays were performed to test the proliferation ability of the cells with BeyoClick™ Edu-647 cell proliferation test kit (Beyotime, China) at 24 h according to the manufacturer’s protocol.

### Transwell assay

Transwell chambers (Corning) with and without Matrigel were used to determine the migration and invasion ability of LUAD cells. Matrigel (BD, Biosciences, CA, USA), stored at -20°C, was melted at 4°C, mixed evenly with serum-free medium, added to the upper chamber, and placed in an incubator at 37°C for 30 minutes successively. Cell suspension was prepared using serum-free medium. About 4-5×10^4^ cells were added into the upper chamber, with 500 μL serum added to the lower one. After being cultured for 24 h, cells on Matrigel gel surface and polycarbonate membrane were removed by wet cotton swab, cleaned with buffer, fixed with 4% paraformaldehyde, and stained with Giemsa successively. The cells were then counted in six randomly selected fields of view under a microscope, and the results were expressed as means ± standard deviation (SD). The experiment was performed independently three times.

### Wound healing experiment

Infected cells were cultured in a 35 mm Petri dish (NEST, Wuxi, China). After 2 days, the conventional medium was replaced with that containing 1% serum, and a uniform scratch was drawn with a sterile pipette tip at the bottom of the dish. Then, the Celigo Image Cytometer was used to analyze the images after cell migration for 0 and 24H in the same field and photographed, respectively. Celigo Image Cytometer was used to analyze the scanning images and obtain the cell migration area.

### Cellular F-actin measurement

F-actin content was measured based on a previously described method (14). Cultured cells were fixed with 4% paraformaldehyde at room temperature for 15 min and rinsed with PBS. After being subjected to 0.1% Triton X-100, the cells were incubated with Alexa Fluor 568 phalloidin (Beyotime Biotechnology, Shanghai, China) for 60 min. DAPI was used to stain the nucleus, with anti-quenching agent for cell blocking. F-actin content was measured using a microplate fluorescence reader with an excitation wavelength of 568 nm and emission one at 600 nm. Relative quantification of F-actin content in different time periods was calculated using the following equation: F-actin Δt/F-actin 0 = fluorescence Δt/fluorescence 0.

### Western blot analyses

Total proteins were extracted from lung adenocarcinoma cells and tissues at 4°C. Protein concentrations were detected by a BCA kit (Beyotime Biotechnology, Shanghai, China). Proteins were isolated by SDS-PAGE gel electrophoresis and then transferred onto PVDF membranes. After being blocked with 5% FBS, the membranes were incubated with the following antibodies: NCAPG2 (Abcam; 1:1000), E-cadherin (Santa Cruz, 1:1000), Vimentin (Santa Cruz, 1:1000), Snail (Abcam, 1:500), N-cadherin (Abcam, 1:500), Zeb1 (Abcam, 1:500), MMP2 (Abcam, 1:1000), MMP9 (Abcam, 1:1000), nucleolin (Abcam, 1:1000), p-LIMK (Cell Signaling Technology, 1:1000), LIMK (Cell Signaling Technology, 1:1000), p-cofilin (Cell Signaling Technology, 1:1000), cofilin (Cell Signaling Technology, 1:1000), p-Akt (Cell Signaling Technology, 1:1000), Akt (Cell Signaling Technology, 1:1000), β-actin (Cell Signaling Technology, 1:1000), and HRP-conjugated anti-rabbit IgG and anti-mouse IgG antibodies (Cell Signaling Technology; 1:2000) successively, with PBS rinse three times before and after the incubation with the secondary antibodies. All experiments were repeated independently at least three times. The membranes were exposed by X-ray films after ECL luminescence.

### RNA fish

Cy3-labeled LncRNA-AC009948.5 probes and fluorescein amidite labeled miR-186-5p probes were designed and synthesized by RiboBIO Company (Guangzhou, China). Cells were seeded into 6-well plates with cell climbing slice at a density of 2×10^5^ cells/well. After being fixed with 4% paraformaldehyde for 30 min and permeated with 0.5% triton X-100 for 10 min, the probe signals were detected using a FISH kit (RiboBIO) according to the manufacturer’s instruction. All operations were carried out in the dark.

### Immunofluorescence analyses

Cells were fixed with 4% paraformaldehyde at room temperature for 15 min, washed with buffer, and blocked for 60 min with blocking buffer successively. Then the cells were subjected to diluted antibodies (E-cadherin and Vimentin). After 24 h, the cells were washed with buffer and added with antibodies labeled by Cy3 (Beyotime Biotechnology, Shanghai, China), followed by being incubated in the cassette for 1.5 h. The cells were cleaned with buffer and subsequently stained with 4′,6-diamidino-2-phenylindole (DAPI) (Beyotime Biotechnology, Shanghai, China) for 15 min. Finally, the cells were added with the anti-quenching agent and mounted. The expression of E-cadherin and Vimentin in the cells was analyzed by a confocal laser scanning microscope (Leica SP8, Bensheim, Germany).

### Patients and clinical specimens

Fresh specimens from 80 patients were collected from the Affiliated Hospital of Weifang Medical University from July 2011 to March 2016. The patients were not treated with radiotherapy, chemotherapy, and immunotherapy before the operation. Half of the collected specimens (including normal lung tissues and cancer ones) were immediately placed in liquid nitrogen, with the other half examined by the pathology department of the hospital. All histologic types of the specimens were confirmed to be lung adenocarcinoma. Relevant data for the patients were collected and sorted. All the patients were informed of the study and agreed to participate. The research was approved by the Institutional Review Board (IRB).

### Animal experiment

All animal procedures complied with the National Institutes of Health guide for the care and use of Laboratory animals (NIH Publications No. 8023, revised 1978) and approval of the Ethics Committee (Committee on the Use of Live Animals in Teaching and Research) of Weifang Medical University. Nude mice aged 4-5 weeks were placed in ZH-EVC under specified conditions. The cells were subcutaneously injected into the back of nude mice to establish a subcutaneous tumor model. The growth and changes of the subcutaneous tumor in nude mice were observed regularly. After 30 days, the nude mice were killed and the subcutaneous tumors and lung tissues were collected. The metastasis of lung adenocarcinoma was analyzed by HE section. NCAPG2 expression in the subcutaneous tumors was detected by Western blot analyses.

### Bioinformatics analysis

Based on LncRNA microarray data in our previous study published in 2018 (15). Survival curves were plotted using the Kaplan–Meier method and the differences in overall or relapse-free survival were assessed with the Gehan-Breslow-Wilcoxon test. The interactions between ncRNAs–miRNAs and miRNAs-mRNAs were predicted using miRDB (http://mirdb.org/) and miRWALK (http://mirwalk.umm.uni-heidelberg.de/) online analytical tools. The relevant genes were input into aberrant gene expression analyses through fold change as well as a P-value calculated using Student’s t-test. The threshold for aberrantly regulated genes was set as a fold change ≥2.0. The data of NCAPG2 expression levels and clinical stages were obtained from GEO (#GSE19188 and #GSE33532) databases, and survival time was obtained from Kaplan–Meier plotter (http://www.kmplot.com/) databases. By Gene Expression Profiling Interactive Analysis (GEPIA) (http://gepia.cancerpku.cn/), a Pearson correlation test was conducted to evaluate the relationship between LncRNA-AC009948.5 and EMT proteins.

### Statistical analyses

All statistical analyses were performed using the GraphPad Prism 5 software (Graph-Pad Software, San Diego, USA). The correlation between the survival time of lung adenocarcinoma and LncRNA-AC009948.5 expression was analyzed by Kaplan-Meier analysis. Data from two groups were analyzed by independent sample t-test, with those from multiple ones analyzed by one-way ANOVA. Data were expressed by means ± SD. *P* values less than 0.05 are considered statistically significant.

## Results

### Expression of LncRNA-AC009948.5 is up-regulated in lung adenocarcinoma cells and tissues, and patients with high level of LncRNA-AC009948.5 demonstrate poor prognosis

**Figure 1:**
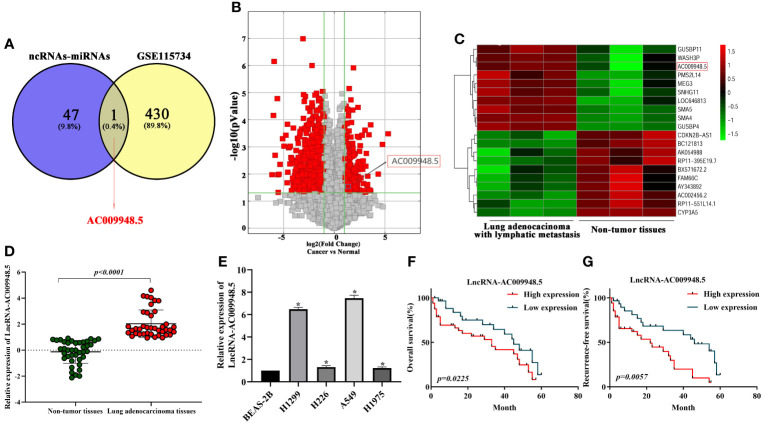
Expression of LncRNA-AC009948.5 is up-regulated in lung adenocarcinoma cells and tissues, and patients with a high level of LncRNA-AC009948.5 demonstrate poor prognosis. **(A)** Schematic flowchart showed the overlapping of LncRNA could combine with miR-186-5p and the upregulated and LncRNAs collection in LncRNA microarray data (GSE115734) (filtered by fold change ≥2 and p-value ≤ 0.05). **(B)** Volcano plots show that thousands of LncRNAs are significantly different by using lncRNA expression thresholds of more than twofold change with *P<*0.05 in GSE115734. **(C)** Clustered heatmap of significant differentially expressed LncRNAs in GSE115734. Each sample contained a mixture of three repeats, red represents high expression, and green represents low expression. **(D)** Expression of LncRNA-AC009948.5 in 80 lung adenocarcinoma tissues and paired non-tumor ones analyzed by qRT-PCR. GAPDH was used as an internal control. The results were expressed as Log2. **(E)** Expression of LncRNA-AC009948.5 in human normal lung epithelial cells (BEAS-2B) and human lung adenocarcinoma cell lines (H1299, H226, A549, and H1975). GAPDH was used as an internal control. Data represent the mean ± S. D, *P<0.05 versus on BEAS-2B cells. The results were repeated three times independently. **(F)** Kaplan-Meier analysis for overall survival based on differential expression levels of LncRNA-AC009948.5 in patients with lung adenocarcinoma. **(G)** Kaplan-Meier analysis for recurrence-free survival based on different expression levels of LncRNA-AC009948.5 in patients with lung adenocarcinoma.

To identify LncRNAs aberrantly expressed in LUAD, the interactions between ncRNAs–miRNAs and miRNAs-mRNAs were predicted using miRDB (http://mirdb.org/) and miRWALK (http://mirwalk.umm.uni-heidelberg.de/) online analytical tools. And the microarray results showed that the expression of LncRNA-AC009948.5 in lung adenocarcinoma with lymphatic metastasis was 2.1434076 times higher than that in normal ones (**Figures 1A–C**). Thus, we selected LncRNA-AC009948.5 as the research object in the present study. To validate the microarray results, we analyzed the expression of LncRNA-AC009948.5 through qRT-PCR in 40 pairs of non-tumor tissues and lung adenocarcinoma. The results showed that the expression level of LncRNA-AC009948.5 in lung adenocarcinoma tissues was significantly higher than that in paired non-tumor ones (**Figure 1D**). We detected the expression of LncRNA-AC009948.5 in human normal lung epithelial cells BEAS-2B and lung adenocarcinoma cell lines by qRT-PCR, and found higher expression of LncRNA-AC009948.5 in the latter than former (**Figure 1E**). By analyzing the relationship between the expression of LncRNA-AC009948.5 and clinicopathological parameters of patients, we found the expression of LncRNA-AC009948.5 was significantly correlated with tumor size, lymph node, distant metastasis, and histological grade but not gender or age (**Table 1**). We used Kaplan-Meier analysis to study the correlation between the survival time of patients with lung adenocarcinoma and the expression of LncRNA-AC009948.5. The total and relapse-free survival rates of patients with low LncRNA-AC009948.5 expression were higher than those with high expression (**Figures 1F, G**). These results suggest that LncRNA-AC009948.5 may function as an oncogene in lung adenocarcinoma.

**Table 1:** Correlation between the clinical pathologic features and expression of LncRNA-AC009948.5.

Variables	LncRNA-AC009948.5		*p* Value
	High expression	Low expression	
Gender			
Male	17	21	
Female	23	19	0.370
Age (years)			
≤50	18	24	
≥51	22	16	0.179
Tumor size (cm)			
≤5cm	23	9	
>5cm	17	31	0.001
Lymph node metastasis			
Yes	22	11	
No	18	29	0.012
Distant metastasis			
Yes	24	13	
No	16	27	0.014
Histological grade			
I ~ II	11	25	
III ~ IV	29	15	0.002

### Effects of LncRNA-AC009948.5 on lung adenocarcinoma cells apoptosis, proliferation and metastasis *in vitro* and *in vivo*

**Figure 2:**
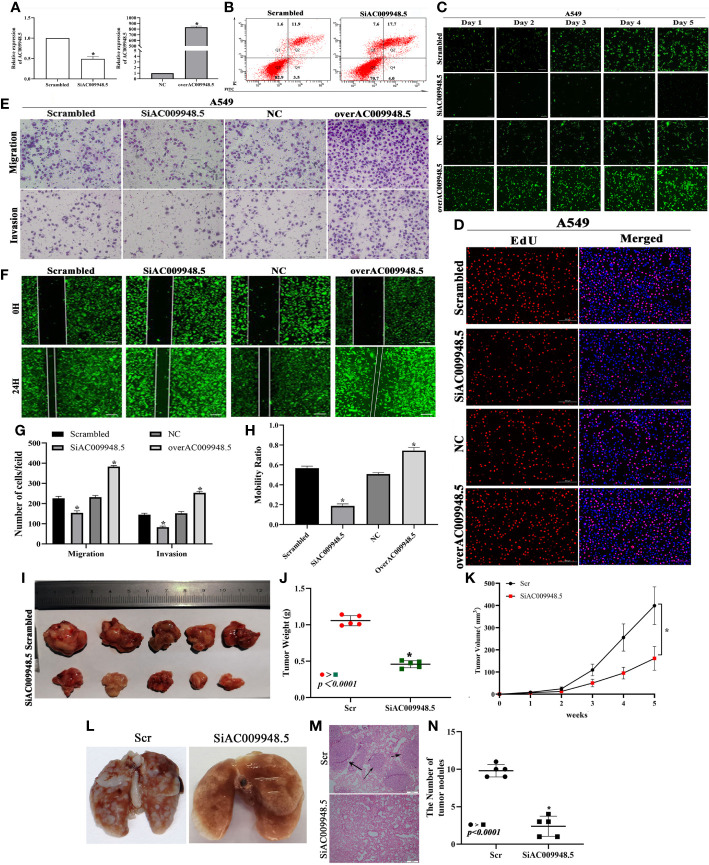
Effects of LncRNA-AC009948.5 on lung adenocarcinoma cells apoptosis, proliferation, and metastasis *in vitro* and *in vivo*. **(A)** Expression of LncRNA-AC009948.5 after infection of A549 cells detected by qRT-PCR. **(B)** Flow cytometric analysis of infected A549 cells. **(C)** Cell proliferation ability detected by Celigo system. **(D)** Cell proliferation ability detected by EdU staining. **(E)** The migration and invasion ability of the cells in each group were detected by Transwell assay, and the cells were counted and analyzed. The results are presented in the number of each fold. Scale bar: 100 μm. **(F)** Cell mobility ability was detected by wound healing assay, and the cells were counted and analyzed. The results are presented in percentages. Scale bar: 200 μm. Data are represented as mean ± S.D. *P<0.05. The results were repeated three times independently. **(G, H)** Migration and invasion assays for transfected cells. **(I, J)** The weight and image of tumors excised from xenograft models. **(K)** Tumor growth curve. *P<0.05 vs. Scr. **(L)** Representative pictures of pulmonary metastatic nodules of mice after injection. **(M)** H&E staining showed pulmonary sections with metastatic nodules (scale bar: 200 μm). **(N)** Scatter plot was used to count the number of lung metastases (n=5).

To detect the effects of LncRNA-AC009948.5 on lung adenocarcinoma cells, we used lentivirus to knock down LncRNA-AC009948.5 in A549 and H1299 cells. And the whole length of the LncRNA-AC009948.5 overexpression vector was constructed and infected into A549 and H1299 cells. The subsequent qRT-PCR verified the knockdown and overexpression efficiency of LncRNA-AC009948.5 (**Figures 2A**, **S1A**). Next, flow cytometry analysis demonstrated that LncRNA-AC009948.5 knockdown increased the proportion of early apoptosis cells and late apoptosis or already dead cells (**Figures 2B**, **S1B**). Then, we used HCS Celigo system and EdU staining to determine the biological roles of LncRNA-AC009948.5 in lung adenocarcinoma cell proliferation. All these results showed that cell proliferative capability was remarkably attenuated after LncRNA-AC009948.5 knockdown in A549 and H1299 cells. While, overexpression of LncRNA-AC009948.5 significantly enhanced the proliferative capability of A549 and H1299 cells (**Figures 2C, D**, **S1C, D**). Transwell assay was utilized to measure the migratory and invasion ability of LncRNA-AC009948.5 in A549 and H1299 cells. The migration and invasion ability of the A549 and H1299 cell lines significantly decreased after the knockdown of LncRNA-AC009948.5, while increased in the case of ectopic expression of LncRNA-AC009948.5, indicating that LncRNA-AC009948.5 could promote the migration and invasion of lung adenocarcinoma cells (**Figures 2E, G**, **S1E**). The results of wound healing experiment showed that after the knockdown of LncRNA-AC009948.5, the metastasis ability of the two groups of cells decreased significantly compared with control. In the presence of overexpression vector of LncRNA-AC009948.5, the metastasis ability of the cells increased (**Figures 2F, H**, **S1F**). Collectively, our results indicate that LncRNA-AC009948.5 significantly accelerates the progression of human lung adenocarcinoma.

**Figure 3:**
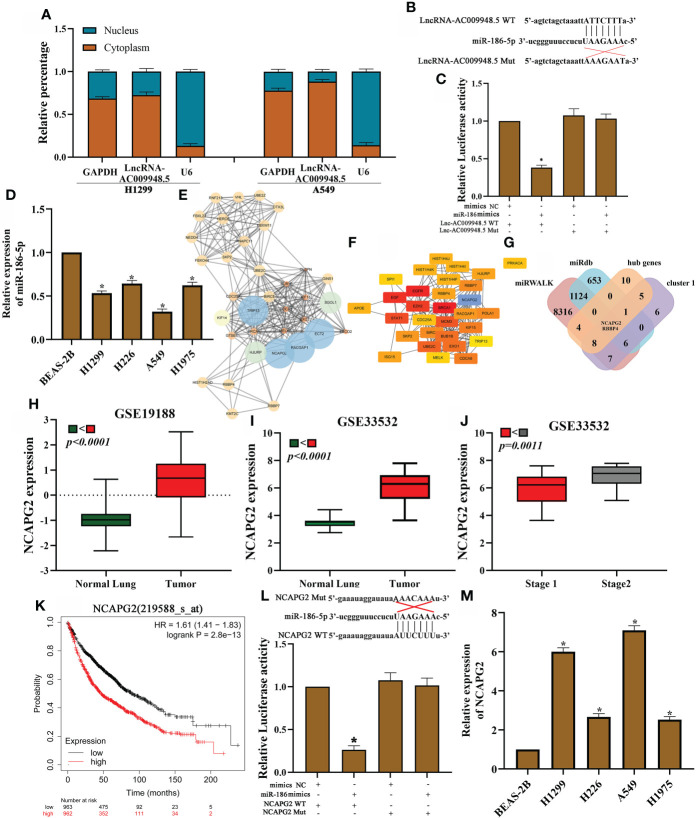
LncRNA-AC009948.5 by sponging miR-186-5p to regulate NCAPG2 expression. **(A)** Cytoplasmic and nuclear LncRNA-AC009948.5 expression detected by qRT-PCR. U6 and GAPDH acted as nuclear and cytoplasmic reference genes, respectively. Relative expression measured by the 2 ^- (cytoplasmic Ct value - nuclear Ct value)^ method. **(B)** Sequence alignments between LncRNA-AC009948.5 and seed sequences of miRNAs. wt, wild-type; Mut, mutant sequence. **(C)** Dual luciferase reporter gene assay to detect the interaction between LncRNA-AC009948.5 and miR-186-5p. **(D)** miR-186-5p expression in different cell lines detected by qRT-PCR. All experiments were repeated independently three times. **(E)** Function clusters determined by the MCODE algorithm (MCODE score=15). **(F)** The top 20 hub genes are based on protein-protein interaction network analyses by cutohubba. **(G)** The Venn diagram shows the overlap between the target genes of miR-186-5p, cluster 1 and hub genes. **(H)** NCAPG2 was highly expressed in NSCLC tissues as compared to the normal ones by GSE19188 dataset. **(I)** NCAPG2 was highly expressed in NSCLC tissues as compared to the normal ones by GSE33532 dataset. **(J)** High NCAPG2 expression was associated with advanced clinical tumor stage. **(K)** Kaplan-Meier analysis for overall survival based on differential expression levels of NCAPG2 in patients with lung adenocarcinoma. **(L)** Dual luciferase reporter gene assay to detect the interaction between NCAPG2 and miR-186-5p. Mimic NC, empty vector control; mimic, miR-186 mimic. **(M)** NCAPG2 expression in different cell lines was detected by qRT-PCR. All experiments were repeated independently three times.

To analyze the effects of LncRNA-AC009948.5 on LUAD cells *in vivo*, The infected cells were injected subcutaneously into the back of nude mice to establish a xenograft model. The results show that AC009948.5 promotes the growth, volumes, and weights of tumors (**Figures 2I–K**). And the metastasis of lung adenocarcinoma was evaluated by H&E section. The metastatic nodules in the group with low LncRNA-AC009948.5 expression decreased significantly compared with the control (**Figures 2L–N**). Hence, LncRNA-AC009948.5 promotes the metastasis of lung adenocarcinoma cells *in vivo*.

### LncRNA-AC009948.5 by sponging miR-186-5p to regulate NCAPG2 expression

Our previous studies suggest that miR-186-5p can inhibit the migration and invasion of human non-small-cell lung cancer cells by modulating PTTG1 (16). But whether the miR-186-5p could play a role in lung adenocarcinoma through ceRNA mechanism remains unknown. Based on LncRNA microarray data in our previous study published in 2018 (15). To investigate the specific role of LncRNA-AC009948.5 in lung adenocarcinoma, we isolated and extracted RNA from the nucleus and cytoplasm of H1299 and A549 cells, respectively. We determined the location of LncRNA-AC009948.5 through qRT-PCR and found it mainly located in the cytoplasm, suggesting its probable involvement in the ceRNA mechanism (**Figure 3A**). We further constructed wild-type and mutant GV272-LncRNA-AC009948.5 vectors incorporating miRNA binding sites (**Figure 3B**) for dual-luciferase reporter assays, and co-transfected these vectors with miRNA mimics to determine if LncRNA-AC009948.5 could bind to the miR-186-5p. Results showed the LncRNA-AC009948.5 directly bound to miR-186-5p (**Figure 3C**). We detected the expression of miR-186-5p in normal lung epithelial cells BEAS-2B and lung adenocarcinoma cell lines (H1299, H226, A549, and H1975) by qRT-PCR, while miR-186-5p was downregulated in LUAD cells compared with BEAS-2B ones (**Figure 3D**). Using miRWALK and miRdb, hub genes and cluster1 predicted target genes were identified in the prediction database of the miRNA target genes. The STRING and Cytoscape were used to predict protein interactions among the target genes of miR-186-5p signature. The module network was constructed by clustering module eigengene distances by MCODE and cytohubba plug-in (**Figures 3E, F**). Finally, after taking the intersection, we identified 2 candidate target genes for miR-186-5p (RBBP4 and NCAPG2) (**Figure 3G**). Based on the results of the mRNA microarray (15), the expression of NCAPG2 in lung adenocarcinoma with lymphatic metastasis was 3.120532 times higher than that in a normal one. And the expression of RBBP4 in lung adenocarcinoma with lymphatic metastasis was 2.0077027 times higher than that in a normal one. And NCAPG2 was highly expressed in NSCLC tissues when compared with the normal ones by analyses using GSE19188 and GSE33532 datasets (**Figures 3H, I**). And high NCAPG2 expression was associated with advanced clinical tumor stage (**Figure 3J**). High levels of NCAPG2 expression were associated with shorter overall survival time in patients with lung adenocarcinoma, as evidenced by Kaplan Meier-plotter (**Figure 3K**). Luciferase reporter assays demonstrated that miR-186-5p could bind to NCAGP2 (**Figure 3L**). In addition, RNA pull-down assays showed that the miR-186-5p expression was more enriched on the biotin-labeled LncRNA-AC009948.5 probe (**Figure S2A**). At the same time, we detected the expression of NCAPG2 in LUAD cells by qRT-PCR and western blot, and found upregulated in all these four kinds of cells compared with BEAS-2B ones (**Figures 3M**, **S2B**). Meanwhile, relative estimation of paired LUAD and ANT tissues by Western blot, demonstrated that the protein levels of NCAPG2 were increased in all LUAD specimens in comparison with related ANT ones (**Figure S2C**). The effects of overexpression miR-186 on NCAPG2 expression were also examined in LUAD cells by qRT-PCR. The results showed that NCAPG2 was notably down-regulated after transfection of miR-186 into A549 cells (**Figure S2D**). The Western blot results clarified that overexpression of miR-186 markedly reduced the protein levels of NCAPG2 (**Figures S2E, F**). Then, expression levels of NCAPG2 in the subcutaneous tumor were detected by Western blot analyses. The expression of NCAPG2 in the SiAC009948.5 group significantly decreased compared with control (**Figure S2G**). These results suggest that LncRNA-AC009948.5 functions as a ceRNA for miR-186-5p to regulate NCAPG2 expression.

### LncRNA-AC009948.5 affects the invasion, metastasis, and EMT of lung adenocarcinoma cells by targeting miR-186-5p

**Figure 4:**
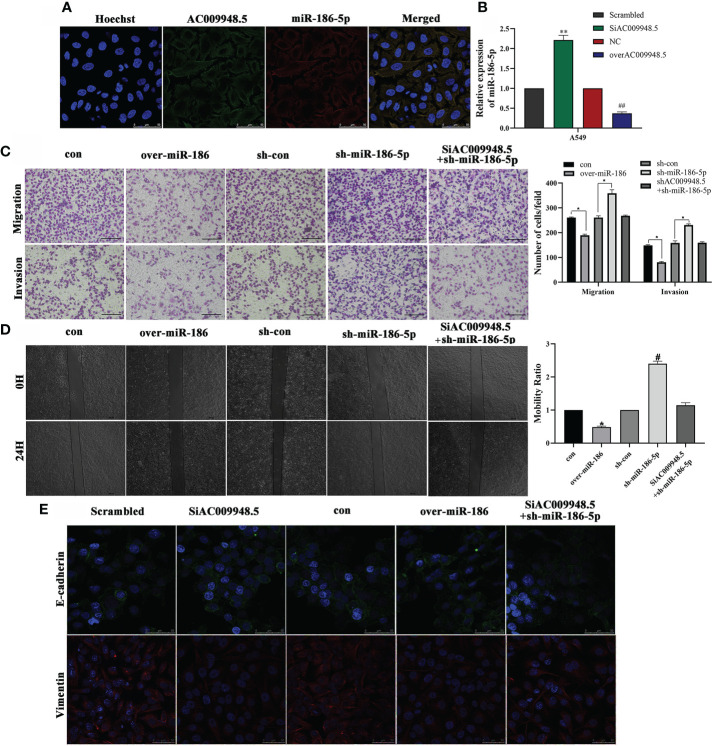
LncRNA-AC009948.5 affects the invasion, metastasis, and EMT of lung adenocarcinoma cells by targeting miR-186-5p. **(A)** RNA FISH showed that LncRNA-AC009948.5 was co-localized with miR-186-5p in A549cells. LncRNA-AC009948.5 probes were labeled with Cy-3, and miR-186-5p were labeled with Fam. The nuclei were stained with DAPI. Scale bar = 50 µm. **(B)** miR-186-5p expression in SiAC009948.5 and overAC009948.5 stably transfected lung adenocarcinoma cells detected by qPCR. All experiments were repeated independently three times. Data are presented as means ± standard deviation. **P<0.01 compared with Scrambled/A549 cells (5B); *## P< 0.01* compared with NC/A549 group. **(C)** The invasion ability of the cells after co-infection was detected by Transwell assay. Data are represented by mean ± S.D. * P<0.05 compared with con (5C), sh/con (5C) group, determined by Student’s t-test. Invasive cells were counted and analyzed. **(D)** The mobility ability of infected cells was detected by wound healing assay. Data are represented by mean ± S.D. *P<0.05 compared with con, *# P<0.05* compared with sh-con with NC/A549 group (5D). Scale bar, 500 µm. **(E)** Localization of EMT markers (E-cadherin (green) and Vimentin (red)) in A549 cells after co-infection by immunofluorescence analysis. DAPI (blue) was used to stain nuclear DNA (scale bar: 50 μm).

Given that LncRNA-AC009948.5 was mainly localized in the cytoplasm, we speculated that miR-186-5p was localized in the cytoplasm. FISH data indicated that miR-186-5p was also predominantly localized in cytoplasm and roughly overlapped in spatial distribution with LncRNA-AC009948.5 (**Figure 4A**). QRT-PCR showed that miR-186-5p expression was increased in SiAC009948.5 and decreased in overAC009948.5 A549 cells, compared with the Scrambled and NC groups, respectively (**Figure 4B**). The transwell assay and wound healing assays showed that miR-186-5p could decrease the migration and invasion ability of A549 cells. Rescue experiments were conducted to assess whether LncRNA-AC009948.5 promotes lung adenocarcinoma migration and invasion through the miR-186-5p/NCAPG2 axis. The results showed that this effect was partly abolished after co-transfection with SiAC009948.5 (**Figures 4C, D**). The immunofluorescence analysis showed that LncRNA-AC009948.5-knockdown or miR-186 overexpression significantly inhibited lung adenocarcinoma cells’ EMT progression, and these inhibiting effects could be partly reversed by co-transfection of SiAC009948.5 and sh-miR-186-5p (**Figure 4E**). MMP-2 and MMP-9 play an important role in tumor development progression by promoting migration and invasion. The Western blot data revealed that the downregulation of LncRNA-AC009948.5 reduced the levels of MMP-2 and MMP-9 in A549 cells. miR-186-5p knockdown could increase the levels of MMP-2 and MMP-9 in A549 cells. However, this effect of LncRNA-AC009948.5 on the occurrence of lung adenocarcinoma was partly abolished after co-transfection with sh-miR-186-5p (**Figure S3F**). Together, LncRNA-AC009948.5 affects the invasion, metastasis, and EMT of lung adenocarcinoma cells by targeting miR-186-5p.

### LncRNA-AC009948.5 promotes the occurrence of EMT by PI3K/Akt signaling pathways and thereby the invasion and metastasis of lung adenocarcinoma cells

**Figure 5:**
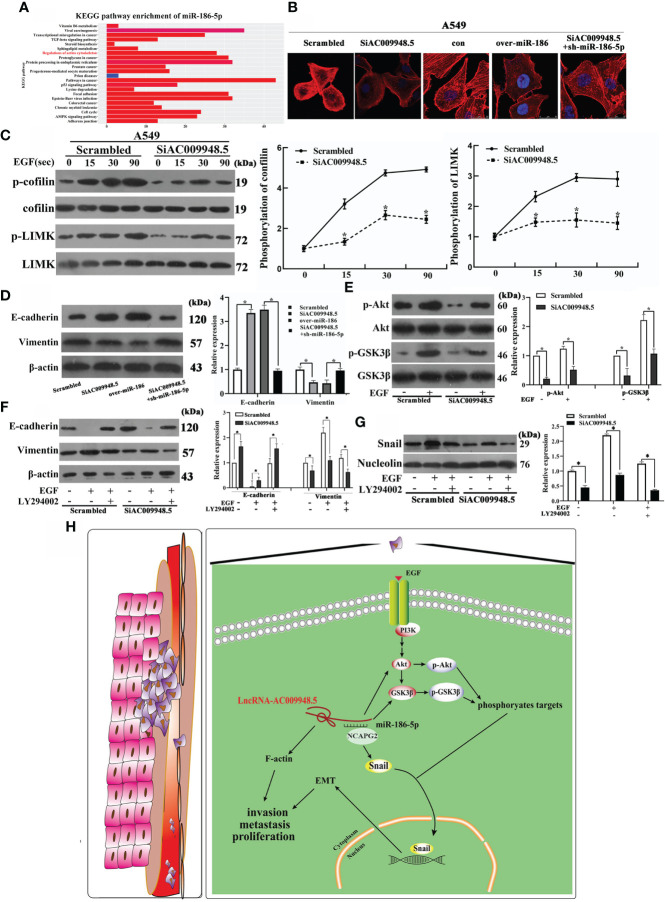
LncRNA-AC009948.5 promotes the occurrence of EMT by signaling pathways and the invasion and metastasis of lung adenocarcinoma cells. **(A)** KEGG analyses for miR-186-5p. **(B)** Observation of F-actin cytoskeleton by rhodamine-phalloidin staining in infected A549 cells. **(C)** Phosphorylation levels of cofilin and LIMK were detected by Western blot after stimulation with EGF (100 ng/mL) for 0, 15, 30, and 90 seconds, respectively. **(D)** Expression levels of E-cadherin and Vimentin were detected by Western blot analysis. **(E)** Total protein was extracted from each group after stimulation with EGF (100 ng/mL). Phosphorylation levels of Akt and GSK3β were detected by Western blot analysis. Akt and GSK3β were used as a loading control. **(F)** The expression of EMT markers (E-cadherin, Vimentin) in infected cells was detected by Western blot analysis. β-Actin was used as a loading control. **(G)** The expression level of transcription factors (Snail) in the nucleus was detected by Western blot. Nucleolin was used as a loading control. The gray value is represented by a histogram. *P<0.05. All experiments were conducted independently three times. **(H)** The proposed model that LncRNA-AC009948.5 promotes the proliferation, invasion, metastasis, and EMT of lung adenocarcinoma by miR-186-5p binding and F-actin polymerization.

The aggregation and depolymerization of F-actin play an important role in the invasion and metastasis of lung adenocarcinoma (16). The KEGG analyses suggest the association of miR-186-5p with regulations of actin cytoskeleton (**Figure 5A**). Therefore, we explored the roles of LncRNA-AC009948.5 in the invasion and metastasis of lung adenocarcinoma by investigating the cellular F-actin and protein level changes of cofilin and LIMK, both of which are closely related to F-actin. The co-transfection of SiAC009948.5 and sh-miR-186-5p increased the F-actin aggregation compared with LncRNA-AC009948.5 knockdown or miR-186 overexpression group (**Figure 5B**). After EGF treatment (100 ng/mL), the phosphorylation of cofilin and LIMK in the cells decreased after the knockdown of LncRNA-AC009948.5, suggesting that it could affect F-actin aggregation by modulating the phosphorylation of cofilin and LIMK (**Figure 5C**). Western blot assays were conducted to assess the mechanisms by which LncRNA-AC009948.5 influences the occurrence of lung adenocarcinoma EMT. The results showed that LncRNA-AC009948.5 promotes the occurrence of lung adenocarcinoma EMT, while, miR-186-5p could inhibit the occurrence of lung adenocarcinoma EMT. However, this effect of LncRNA-AC009948.5 on the occurrence of lung adenocarcinoma EMT was partly abolished by co-transfection with miR-186-5p inhibitors (**Figures 5D**, **S3A**). The PI3K/Akt/GSK3β signaling pathway is related to EMT in lung adenocarcinoma cells (17). In the mechanism study, we used EGF to activate the signaling pathway and detected the expression of related molecules. The levels of p-Akt and p-GSK3β decreased after LncRNA-AC009948.5 knockdown, suggesting that the change in LncRNA expression can affect the PI3K/Akt/GSK3β signaling pathway (**Figure 5E**). To explore the effects of LncRNA-AC009948.5 on EMT through the PI3K/Akt/GSK3β signaling pathway, we treated the infected cells with EGF and pathway inhibitor of LY294002 (20 μM) respectively or simultaneously. The expression of E-cadherin increased while that of Vimentin decreased after LncRNA-AC009948.5 knockdown by Western blot analyses, which could be reversed by activating the signaling pathway. After treatment with the PI3K inhibitor and EGF, the expression levels of E-cadherin and Vimentin recovered, indicating that LncRNA-AC009948.5 could promote EMT in lung adenocarcinoma cells through the PI3K/Akt/GSK3β signaling pathway (**Figure 5F**, **S3B**). The occurrence of EMT is related to the nuclear transfer of Snail (18). We then determined the expression of Snail in the nuclei of the treated cells by Western blot analyses to explore the relationship between the nuclear transfer of Snail and the occurrence of EMT and the PI3K/Akt/GSK3β signaling pathway. The expression of Snail in the nucleus decreased after LncRNA-AC009948.5 was knocked down, indicating that the changes in the expression of LncRNA-AC009948.5 affected the nuclear transfer of Snail. The expression of Snail increased after signaling pathway activation, indicating that LncRNA-AC009948.5 could promote the Snail nuclear metastasis through the signaling pathway. After treatment with the PI3K inhibitor and EGF, the expression of Snail decreased, indicating that LncRNA-AC009948.5 could promote Snail nuclear metastasis in lung adenocarcinoma cells through the PI3K/Akt/GSK3β signaling pathway (**Figure 5G**). In addition, these changes are reflected by regulating the activity of EMT transcription factors (19). The correlation between the expression of LncRNA-AC009948.5 and EMT markers was analyzed using the GEPIA2 database. The expression of LncRNA-AC009948.5 is positively correlated with EMT-related proteins of Zeb1 and N-cadherin (**Figure S3C, D**), the results were further verified by Western blot assay (**Figure S3E**). Hence, LncRNA-AC009948.5 can promote Snail nuclear metastasis in lung adenocarcinoma cells through the PI3K/Akt/GSK3β signaling pathway and consequently the occurrence of EMT. LncRNA-AC009948.5 could promote the proliferation, invasion, and metastasis of lung adenocarcinoma cells and the occurrence of EMT through various mechanisms, including F-actin polymerization induction, signaling pathways activation, and ceRNA (**Figure 5H**) (A model that LncRNA-AC009948.5 and NCAPG2 compete for the binding sites of miR-186-5p is proposed).

## Discussion

Our experimental results support the hypothesis that LncRNA-AC009948.5 affects the invasion and metastasis of lung adenocarcinoma through multiple mechanisms such as ceRNA. LncRNAs play a pivotal role in mediating crosstalk between various cellular components involved in signal transduction, including proteins, RNAs, and DNAs (20). Lots of studies have provided evidence that LncRNAs are involved in the development and progression of human cancers (21). LncRNAs are a diverse group of transcripts whose natural functions and potential as drug targets remain largely undefined (22). Previously we found that miR-186-5p inhibited the invasion and metastasis of lung adenocarcinoma by targeting PTTG1 (16). LncRNA HOXD-AS1 was reported involved in regulation of EMT of ovarian cancer cells by regulating miR-186-5p (23). Therefore, we focus on miR-186-5p. LncRNA and miRNA are key molecules involved in mechanisms of competing for endogenous RNAs (ceRNA) (24). Zhong G successfully constructed a novel ceRNA network, among which each component was significantly associated with breast cancer prognosis (25). It has been extensively reported that cytoplasmic LncRNAs could function as miRNA sponges to modulate mRNA stability or translation and affect related signaling pathways (26).

Recently, several ncRNAs have been used as novel therapeutic targets to treat cancers. Considering different roles of ncRNAs in specific cancer types, ncRNA mimics, antisense oligonucleotides (ASOs) or small molecule drugs have been applied for the treatment of cancers (27). For instance, the LncRNAs MAYA, MALAT1 and LncARSR, have each been targeted for *in vivo* silencing using ASOs to ameliorate the burden of metastatic disease in mouse models (28), miR-34a mimic packaged in a liposomal nanoparticle, called MRX34, has gone through a phase I clinical trial in patients with advanced solid tumor (29). Together, therapeutic targeting of noncoding RNAs (ncRNAs), such as microRNAs (miRNAs) and long noncoding RNAs (LncRNAs), represents an attractive approach for the treatment of cancers, as well as many other diseases (30). Therefore, we screened upstream LncRNAs associated with miR-186-5p by gene chip technology. The expression level of LncRNA-AC009948.5 in tissues and cells of lung adenocarcinoma significantly increased. The expression of LncRNA-AC009948.5 was positively proportional to that of NCAPG2 but inversely proportional to that of miR-186-5p. In this regard, we speculated that LncRNA-AC009948.5 might act as an oncogene in lung adenocarcinoma and was thus employed in the present work.

The analyses of clinicopathological parameters showed that the expression of LncRNA-AC009948.5 was related to the lymph node metastasis of lung adenocarcinoma. The results of survival analysis showed that the change in its expression could be a predictor of the prognosis of lung adenocarcinoma. Various LncRNAs promote the proliferation of cancer cells in lung adenocarcinoma (31–34). In the present study, we found that LncRNA-AC009948.5 could also promote the proliferation of lung adenocarcinoma cells. The invasion and metastasis of tumors are obvious under abnormal conditions, such as growth factors or other chemokines (35–37). The PI3K/Akt signaling pathway activated by EGF can induce EMT. The present study demonstrated that treatment with AC009948.5 knockdown significantly decreased EGF mediated Akt and GSK-3β phosphorylation. Furthermore, LY294002, a PI3K/Akt inhibitor, increased the expression levels of E-cadherin, while inhibiting those of vimentin in A549/H1299 cells treated with EGF. The results from the present study indicated that AC009948.5 knockdown may have an opposing role in EGF induced EMT by inhibiting the activation of Akt and GSK-3β phosphorylation.

The invasion and metastasis of cancer cells to the surrounding tissues are the main cause of cancer-related deaths (38–40). We found that LncRNA-AC009948.5 could promote the invasion and metastasis of cancer cells. The aggregation and depolymerization of F-actin play an important role in cell movement (41). Actin depolymerizing factor/cofilin (ADF/cofilin) can cleave actin and accelerate fibrous actin (F-actin) depolymerization, thereby affecting cell migration. LIMK, an upstream molecule of cofilin, can phosphorylate and inactivate cofilin, and reverse cofilin-mediated F-actin polymerization (42). Our findings suggest that LncRNA-AC009948.5 can promote F-actin polymerization through the cofilin/LIMK signaling pathway and the migration ability of lung adenocarcinoma cells.

An important sign of enhanced invasion and metastasis of lung adenocarcinoma cells is EMT, where epithelial cancer cells obtain interstitial phenotypes, thereby improving the invasion and metastasis of the tumor (43, 44). E-cadherin and Vimentin are typical epithelial and interstitial markers for EMT (45). In the present study, The results showed that LncRNA-AC009948.5 could promote the occurrence of EMT. In addition, various LncRNAs could affect the development of tumors through the PI3K/Akt signaling pathway. Our previous study indicated that PTTG1 could promote the occurrence of EMT through the PI3K/Akt/Snail signaling pathway in lung adenocarcinoma (46, 47). Therefore, we speculated that LncRNA-AC009948.5 might function as an upstream regulatory gene. Further detection of Akt phosphorylation and Snail nuclear transfer showed that LncRNA-AC009948.5 could promote the occurrence of EMT through the pathway.

Some RNAs, possessing microRNA response elements (MREs), can interact with one another through competitive microRNA and are called competitive endogenous RNAs (ceRNAs) (48–50). LncRNAs, such as LncRNA-HNF1A-AS1 (51), LncRNA-MUF (52), and LncRNA-00511 (53) can affect the occurrence and development of tumors as ceRNAs. The locations of LncRNAs in cells and their mechanisms of action vary. If LncRNAs were mainly expressed in the nucleus, their possible mode of action is chromatin regulation and transcription regulation. If in the cytoplasm, then the possible way of action was posttranscriptional regulation where ceRNA combines with microRNAs (54). We used qRT-PCR to quantify the RNA isolated from the nucleus and cytoplasm. LncRNA-AC009948.5 was found to be mainly located in the cytoplasm. These results suggest that LncRNA-AC009948.5 may play an important role in the invasion and metastasis of lung adenocarcinoma as a ceRNA. Furthermore, luciferase reporter assay and RNA pull-down confirmed that LncRNA-AC009948.5 could combine with miR-186-5p.

This study focused on the effects and mechanism of LncRNA-AC009948.5 involved in the development of lung adenocarcinoma. Further studies must be conducted to determine whether other differentially expressed LncRNAs from gene chip analyses also play roles in lung adenocarcinoma, whether LncRNA-AC009948.5 possesses other target genes to regulate lung adenocarcinoma, and whether LncRNA-AC009948.5 works through other mechanisms simultaneously as a ceRNA.

This study found that LncRNA-AC009948.5 could promote the proliferation, chemotaxis, invasion, metastasis, and EMT occurrence of lung adenocarcinoma. LncRNA-AC009948.5, as a ceRNA, competitively combines with miR-186-5p, thereby affecting the invasion and metastasis of lung adenocarcinoma and the occurrence of EMT. These results suggest that LncRNA-AC009948.5 can serve as a new target and prognostic indicator for the clinical treatment of lung adenocarcinoma.

## Data availability statement

The original contributions presented in the study are included in the article/**Supplementary Material**. Further inquiries can be directed to the corresponding authors.

## Ethics statement

All animal procedures complied with the National Institutes of Health guide for the care and use of Laboratory animals (NIH Publications No. 8023, revised 1978). All experiments were performed under the approval of the Ethics Committee at the Weifang Medical University.

## Author contributions

BZ and CY conceived and designed the experiments. JB, HL, XC and LC performed the main experiments. YH, LL, YZ and WZ analyzed the data. All authors read and approved the final manuscript.

## Funding

This work was supported by the National Natural Science Foundation of China (No. 81702932, 81672631, 81402389 and 81641111), the Natural Science Foundation of Shandong Province (No. ZR2019MH033). National Visiting Program of Weifang Medical University (20197-05) and Introduction Plan of Young Creative Talents in Colleges and Universities of Shandong Province (205).

## Conflict of interest

The authors declare that the research was conducted in the absence of any commercial or financial relationships that could be construed as a potential conflict of interest.

## Publisher’s note

All claims expressed in this article are solely those of the authors and do not necessarily represent those of their affiliated organizations, or those of the publisher, the editors and the reviewers. Any product that may be evaluated in this article, or claim that may be made by its manufacturer, is not guaranteed or endorsed by the publisher.

## Abbreviations

LncRNAs, Long-chain non-coding RNAs; EMT, epithelial-mesenchymal transition:; IRB, Institutional Review Board; Luc2, luciferase; DAPI, 4′,6-diamidino-2-phenylindole; ADF/cofilin, Actin depolymerizing factor/cofilin; F-actin, filamentous actin; MREs, microRNA response elements; ceRNAs, competitive endogenous RNAs.
