# Supplementary figures and images for: LncRNA-AC009948.5 promotes invasion and metastasis of lung adenocarcinoma by binding to miR-186-5p (part 1 of 4)
Source: Front Oncol. 2022 Aug 19;12:949951. doi: 10.3389/fonc.2022.949951 (PMC9437580; doi:10.3389/fonc.2022.949951)

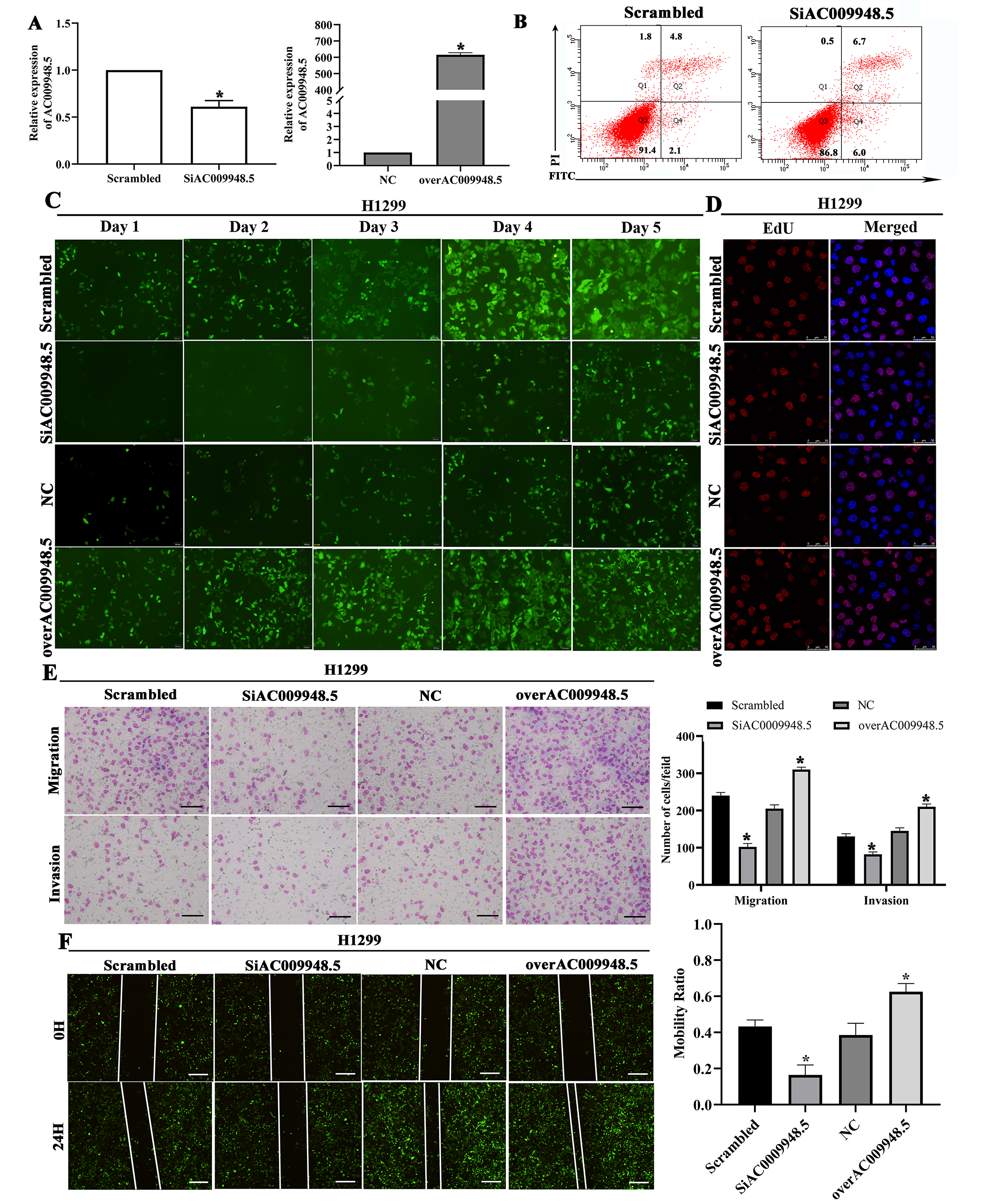

Supplement: Supplementary file 1 [file Image_1.tif]

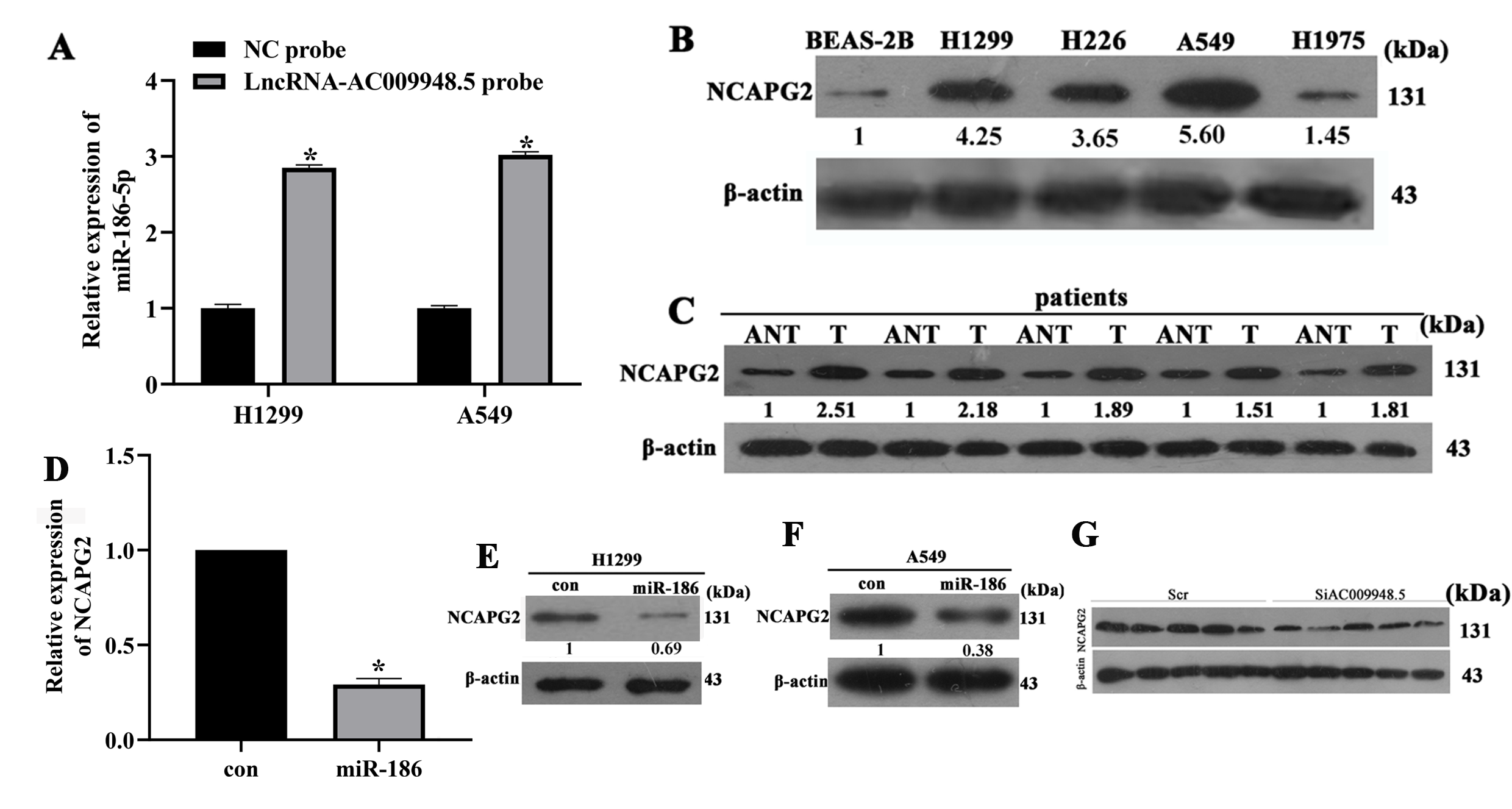

Supplement: Supplementary file 2 [file Image_2.tif]

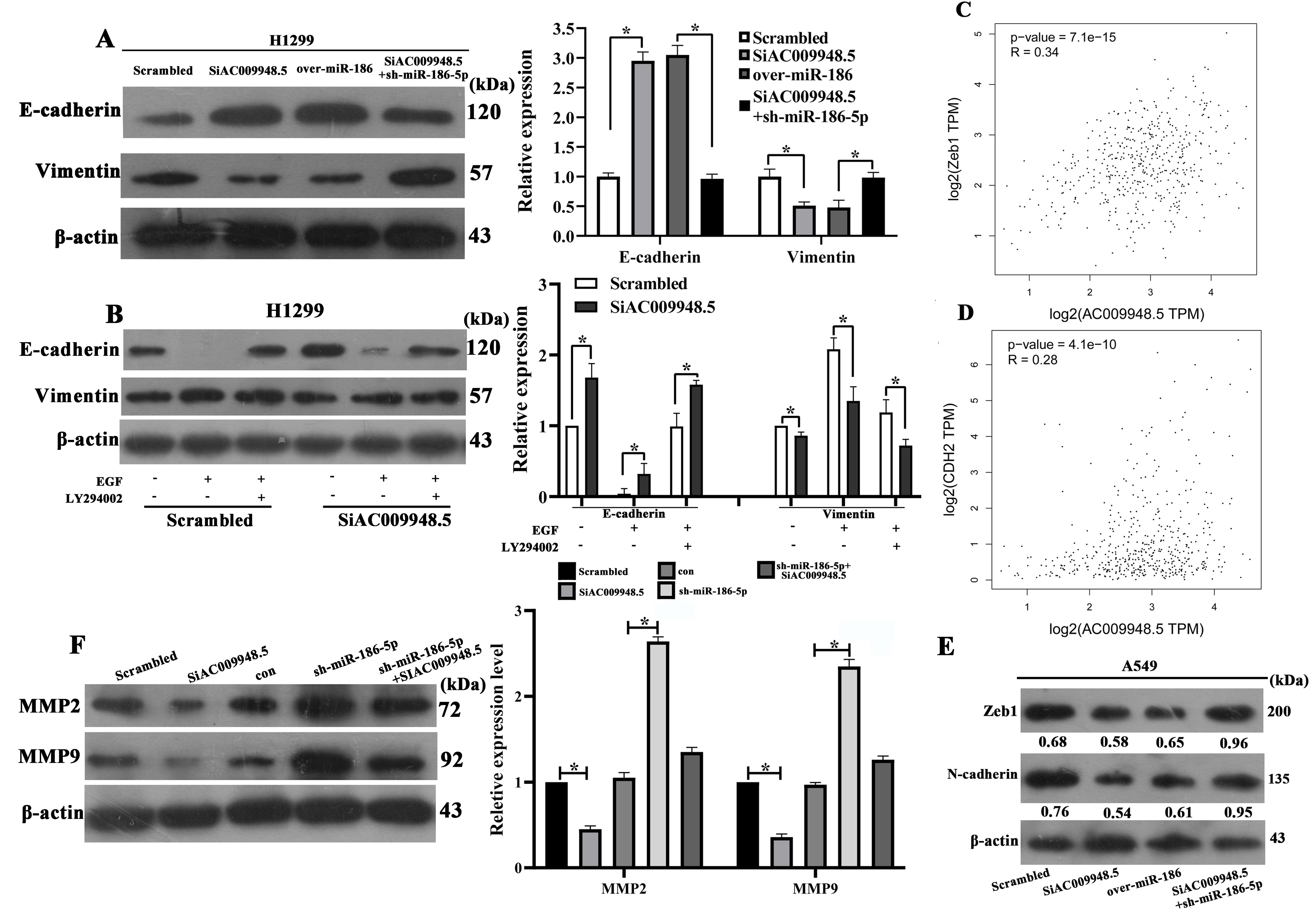

Supplement: Supplementary file 3 [file Image_3.tif]

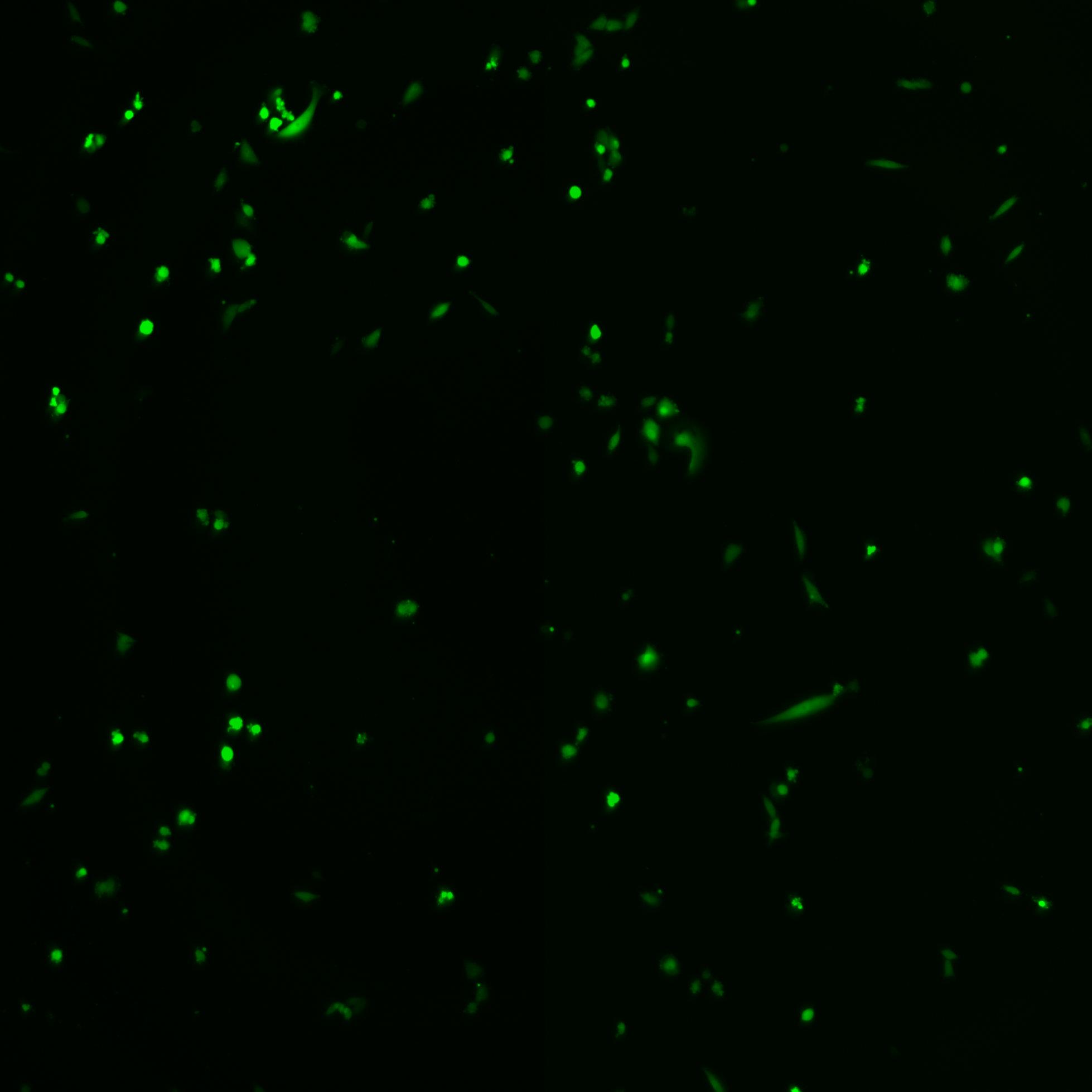

Supplement: Supplementary file 4 [file DataSheet_1.zip › Data Sheet 1/Fig2C/1-day1-NC-AC009948.5.jpg]

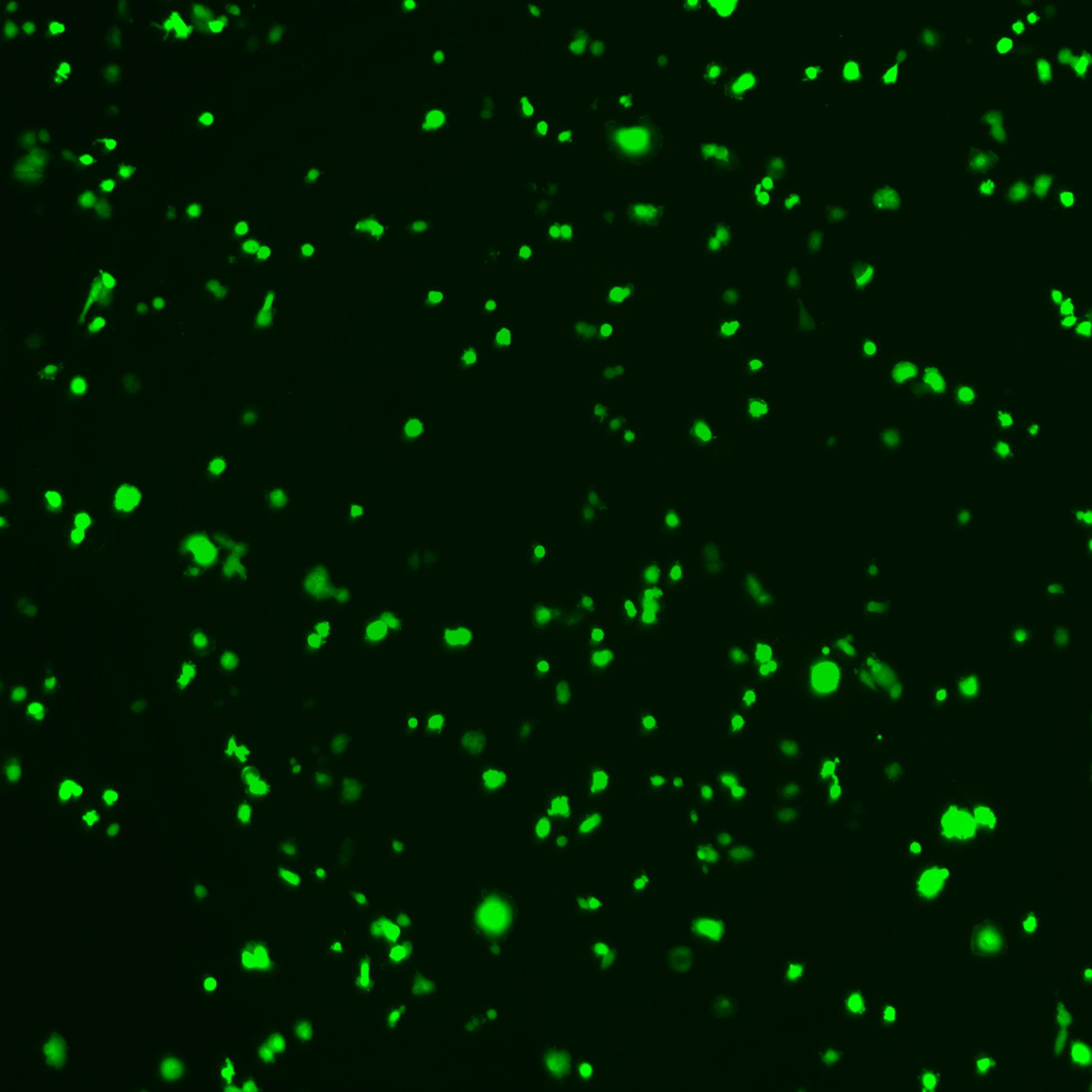

Supplement: Supplementary file 4 [file DataSheet_1.zip › Data Sheet 1/Fig2C/1-day1-overAC009948.5.jpg]

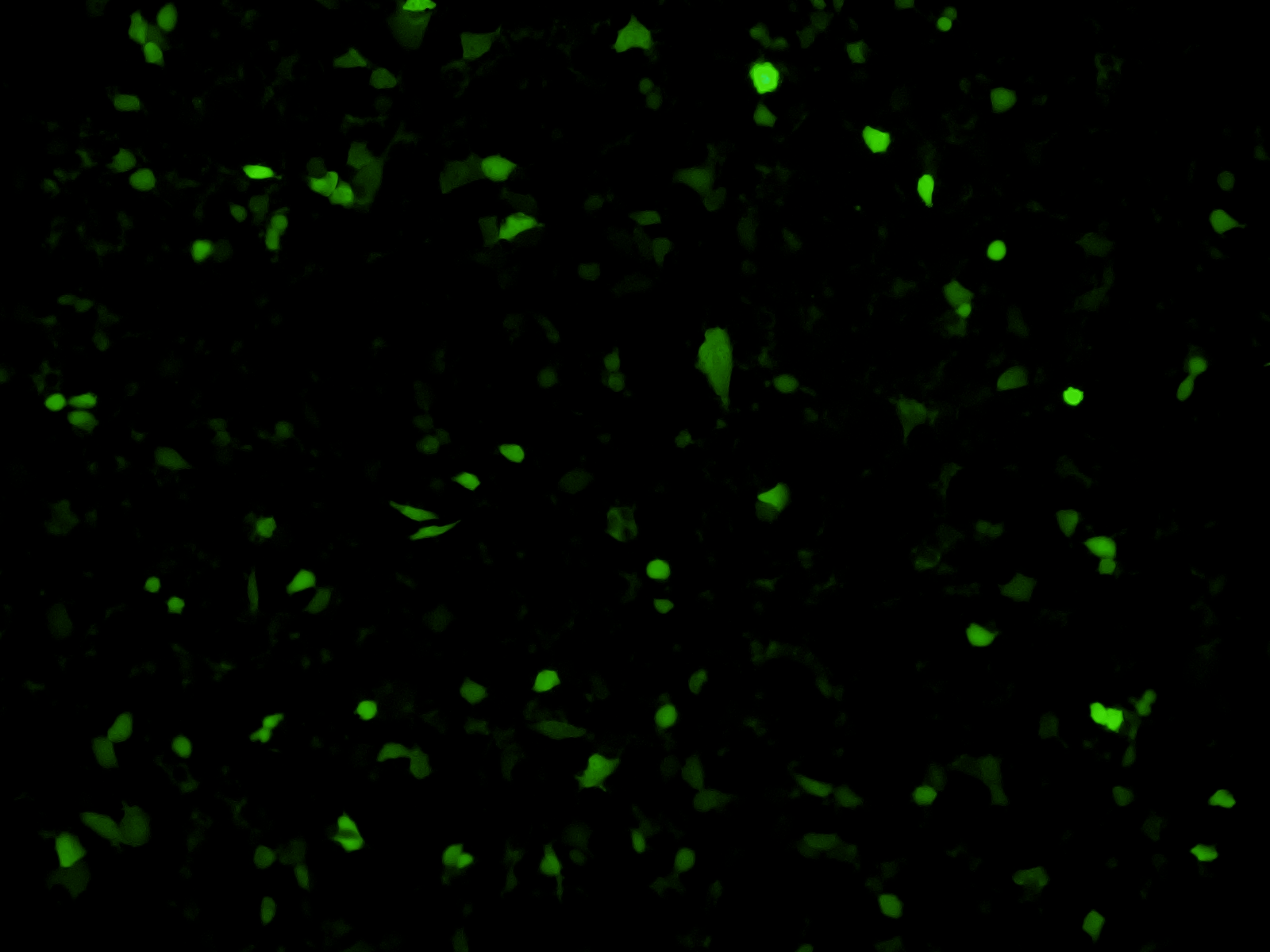

Supplement: Supplementary file 4 [file DataSheet_1.zip › Data Sheet 1/Fig2C/1-day1-Scrambled-AC009948.5.tif]

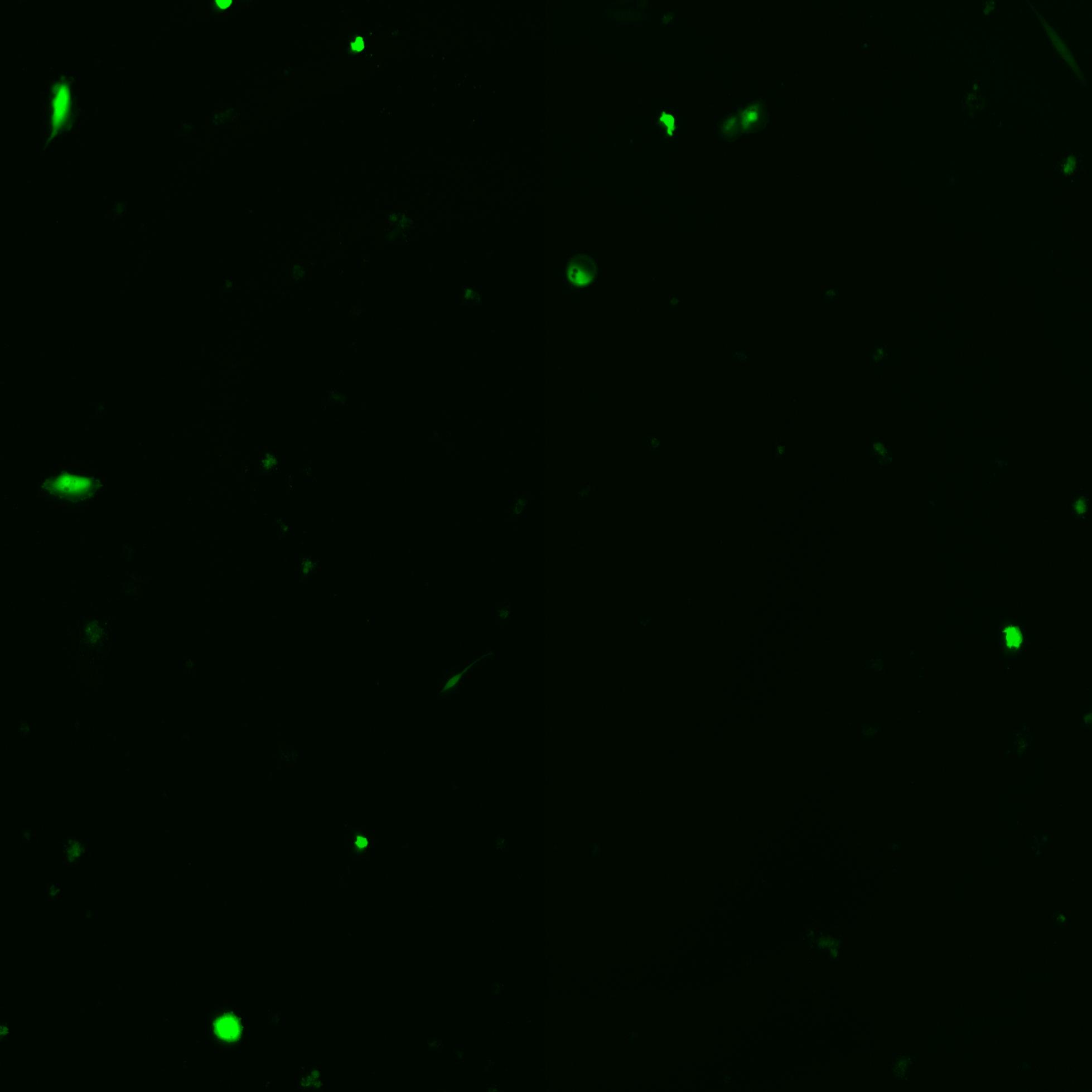

Supplement: Supplementary file 4 [file DataSheet_1.zip › Data Sheet 1/Fig2C/1-day1-siAP009948.5.jpg]

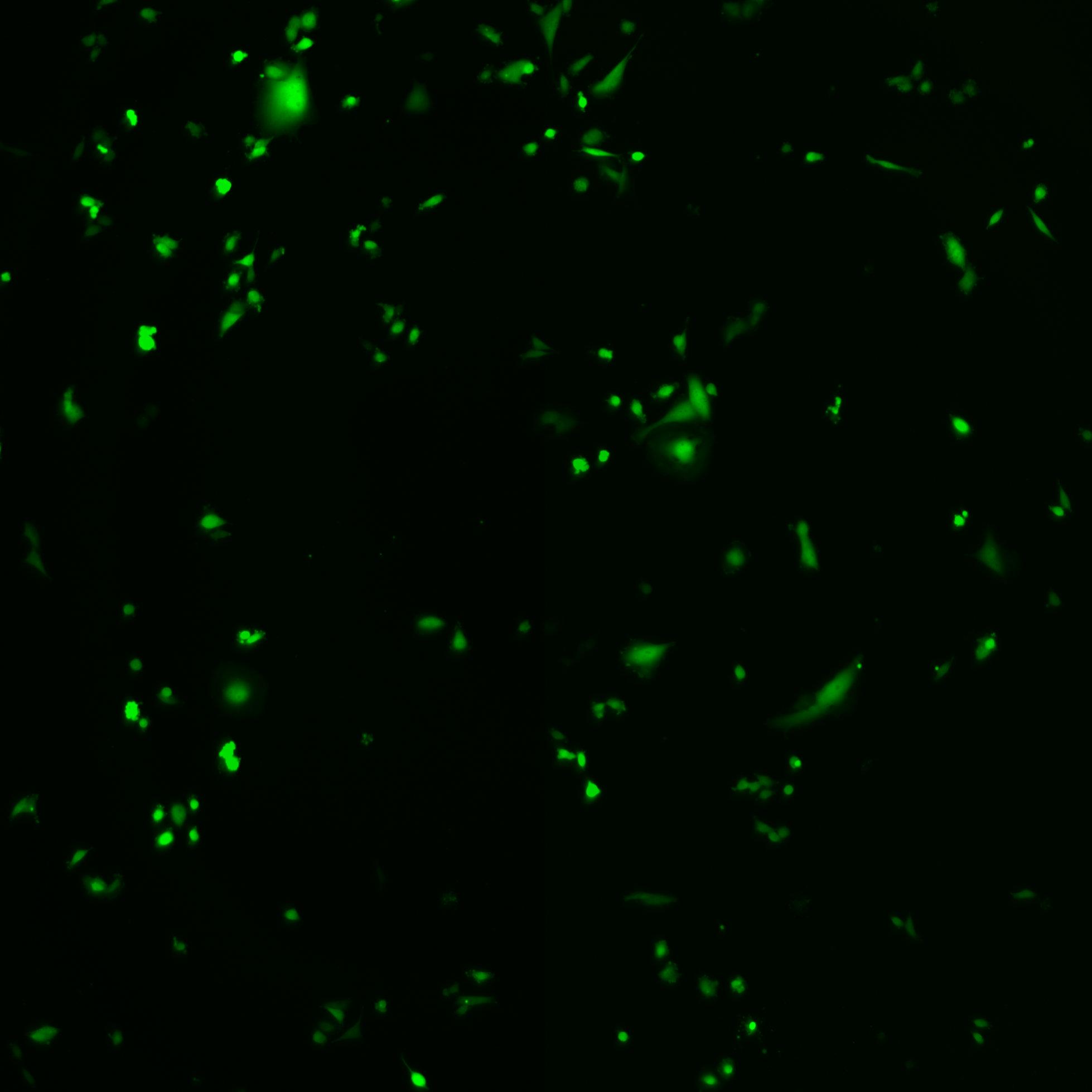

Supplement: Supplementary file 4 [file DataSheet_1.zip › Data Sheet 1/Fig2C/1-day2-NC-AC009948.5.jpg]

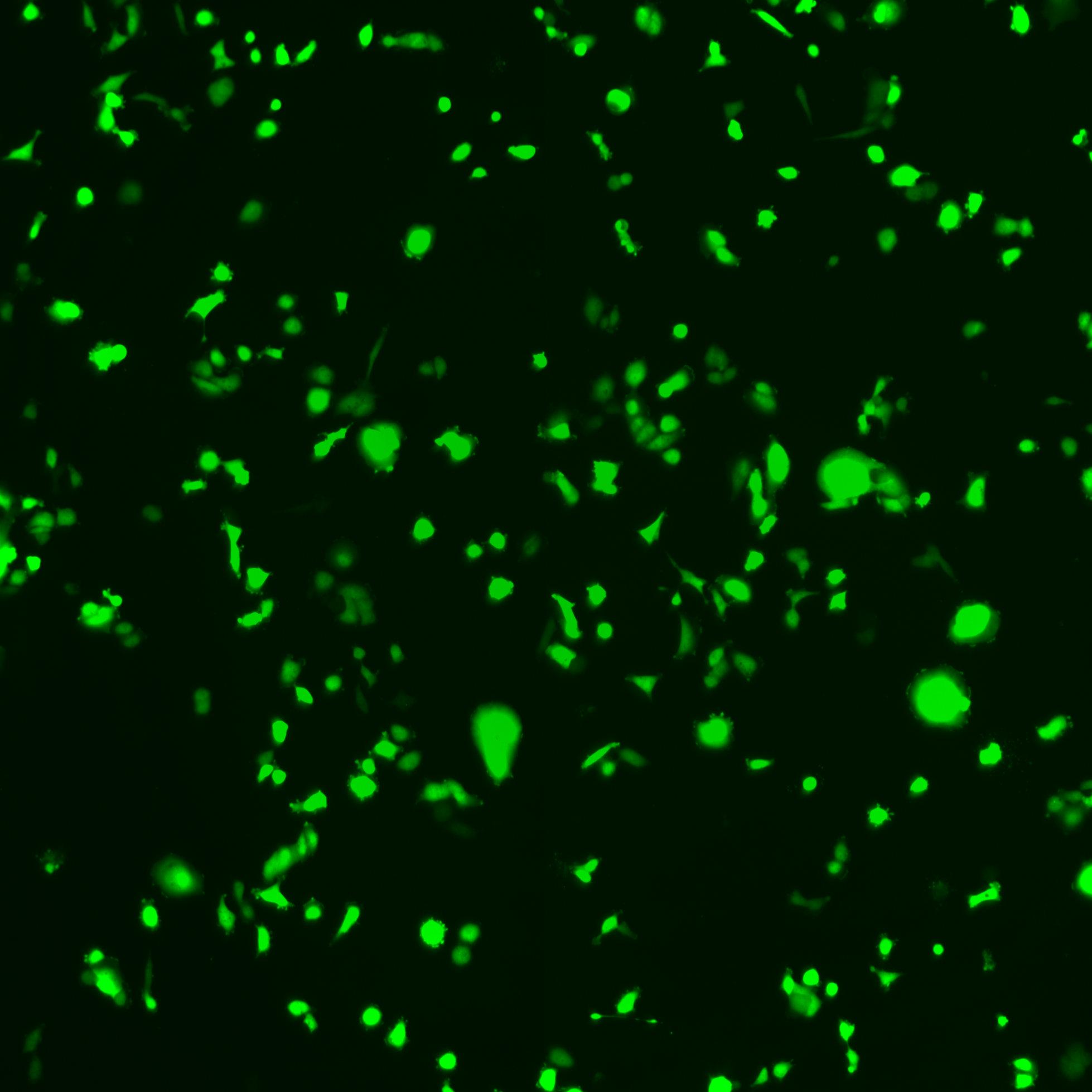

Supplement: Supplementary file 4 [file DataSheet_1.zip › Data Sheet 1/Fig2C/1-day2-overAC009948.5.jpg]

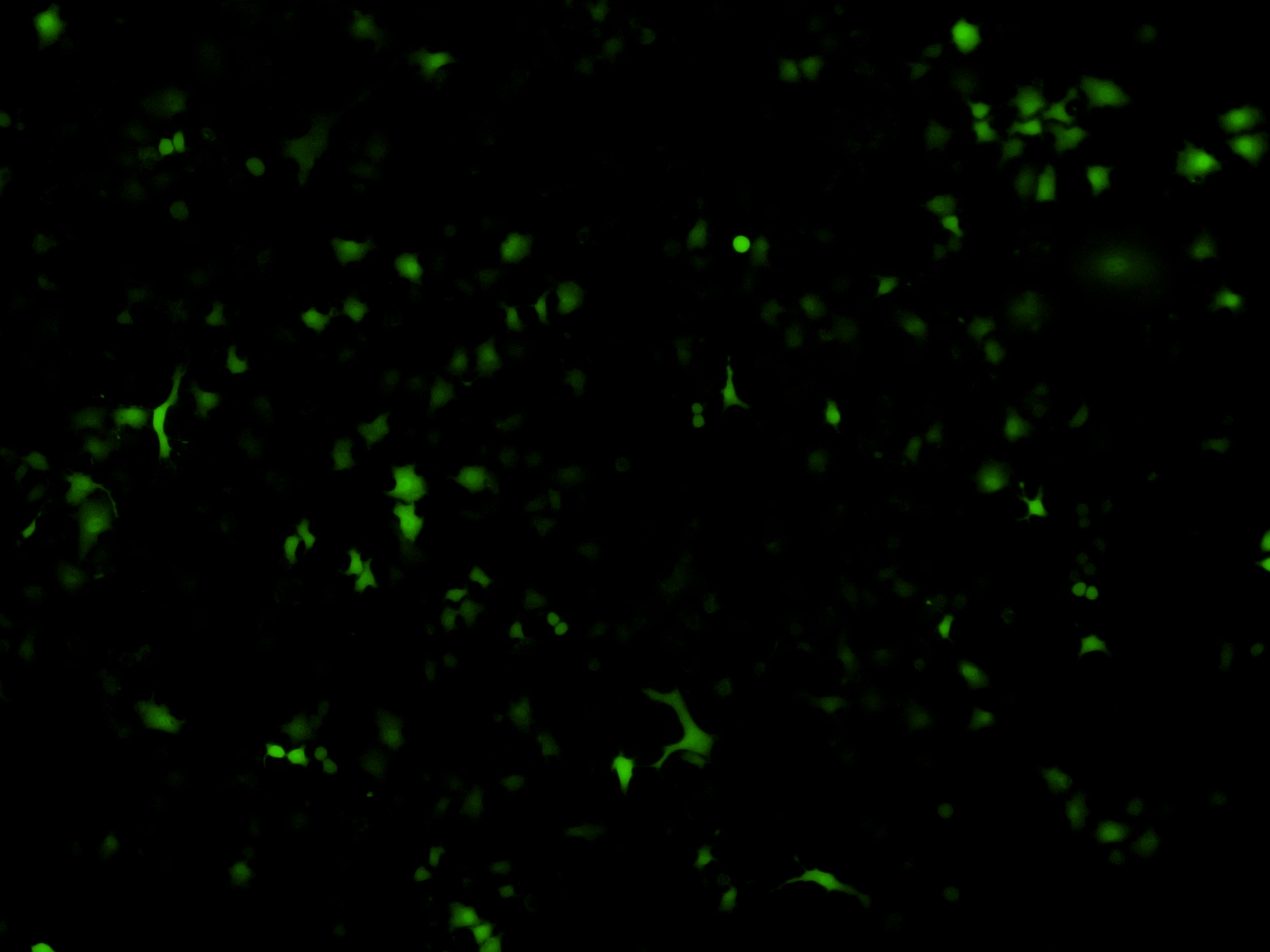

Supplement: Supplementary file 4 [file DataSheet_1.zip › Data Sheet 1/Fig2C/1-day2-Scrambled-AC009948.5.tif]

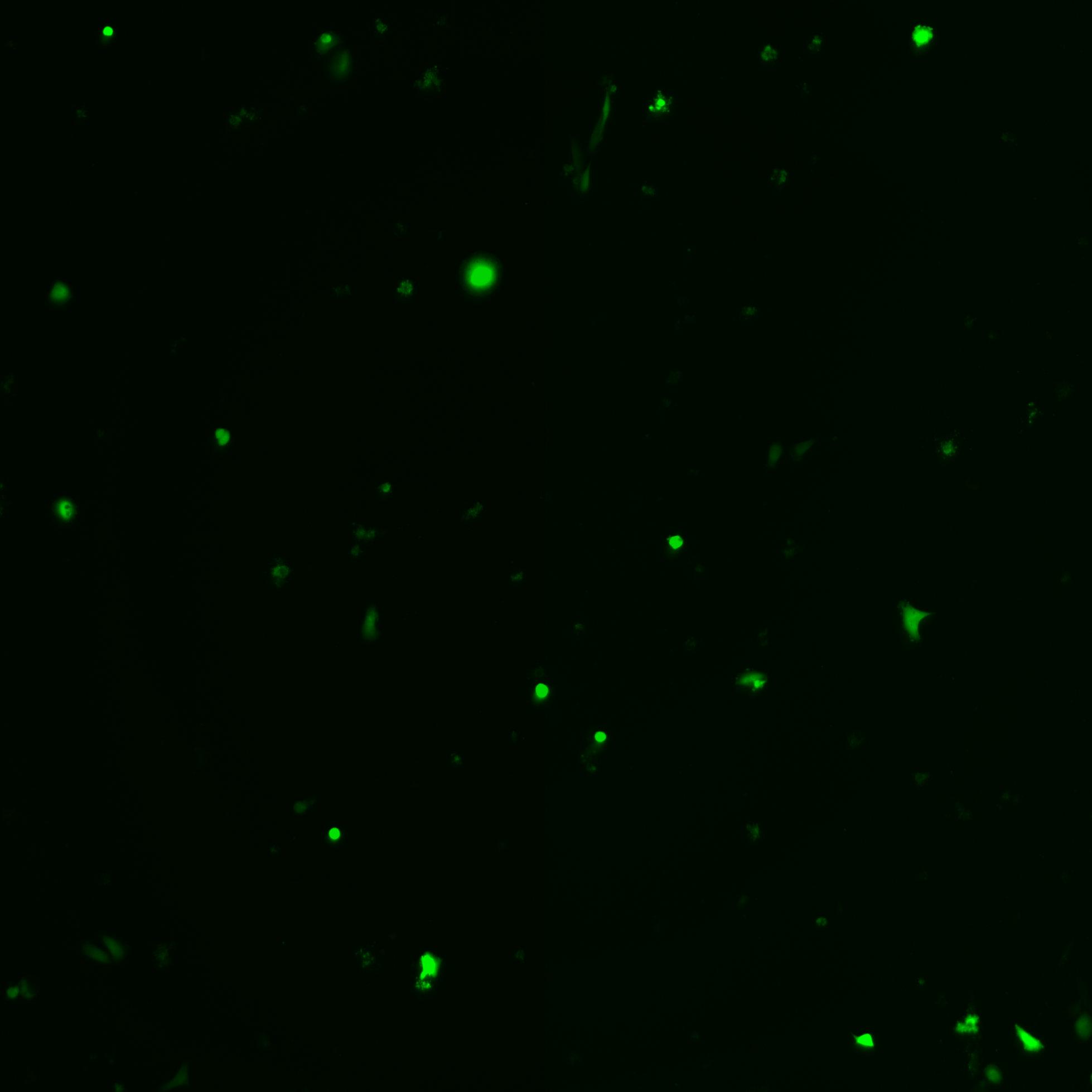

Supplement: Supplementary file 4 [file DataSheet_1.zip › Data Sheet 1/Fig2C/1-day2-siAC009948.5-.jpg]

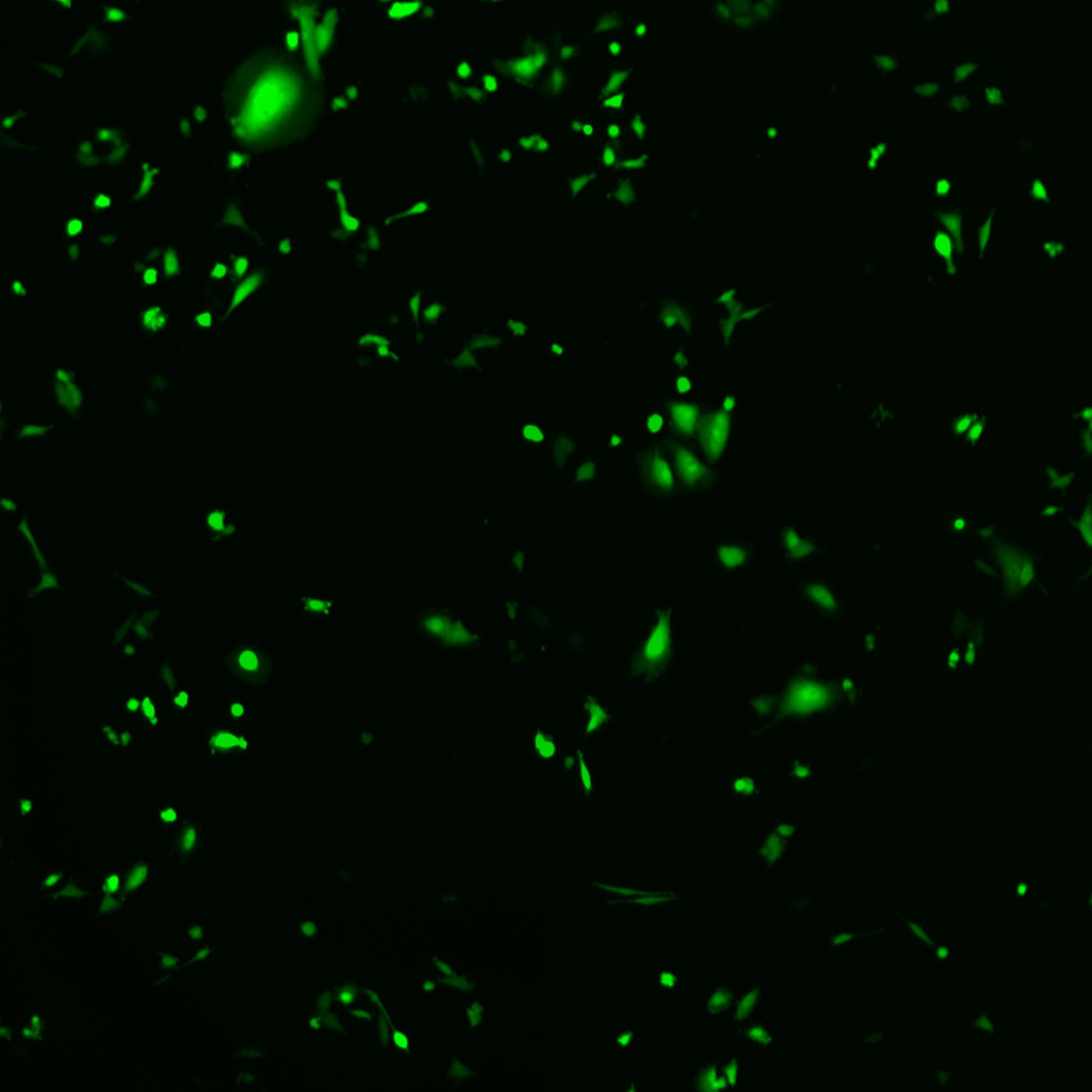

Supplement: Supplementary file 4 [file DataSheet_1.zip › Data Sheet 1/Fig2C/1-day3-NC-AC009948.5.jpg]

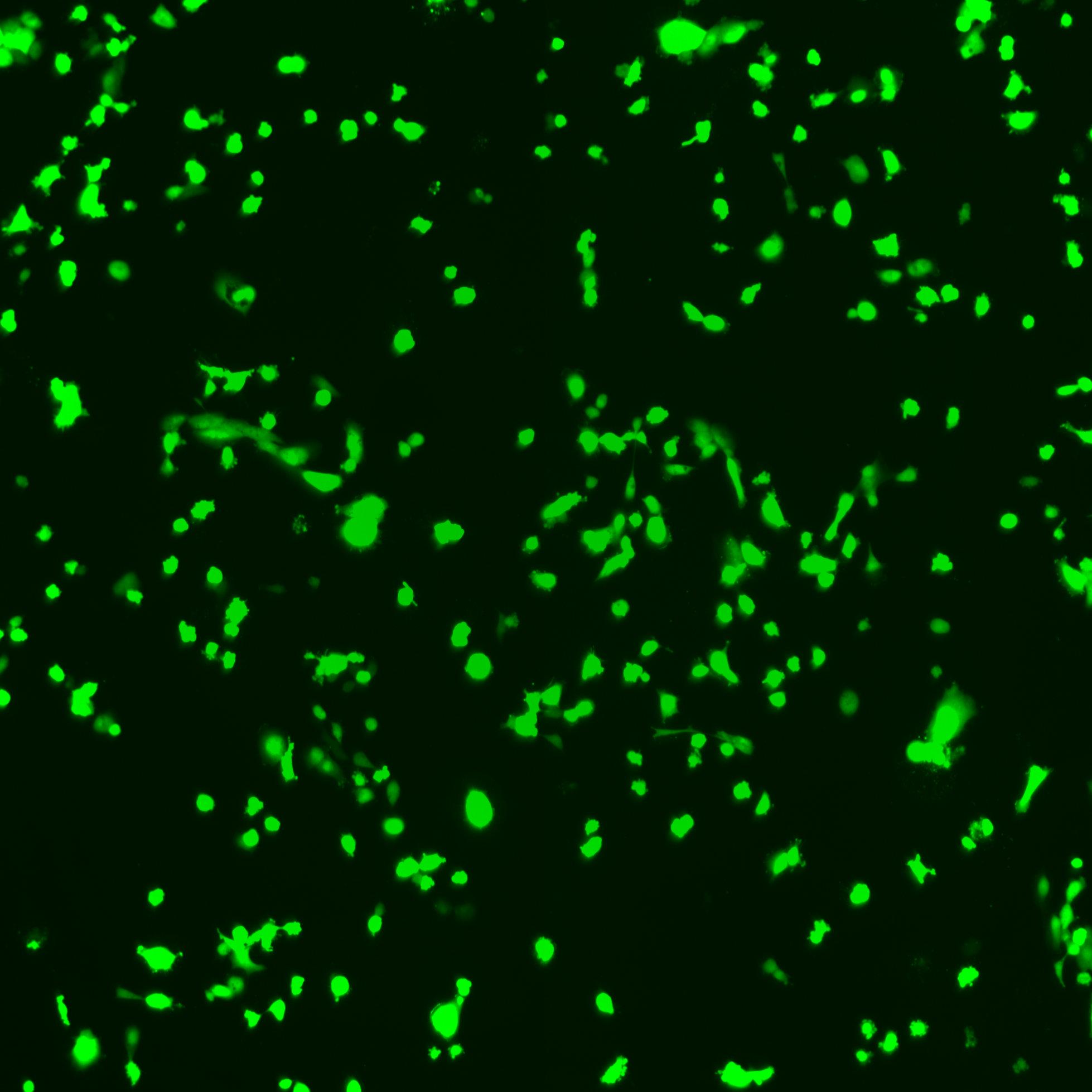

Supplement: Supplementary file 4 [file DataSheet_1.zip › Data Sheet 1/Fig2C/1-day3-overAC009948.5.jpg]

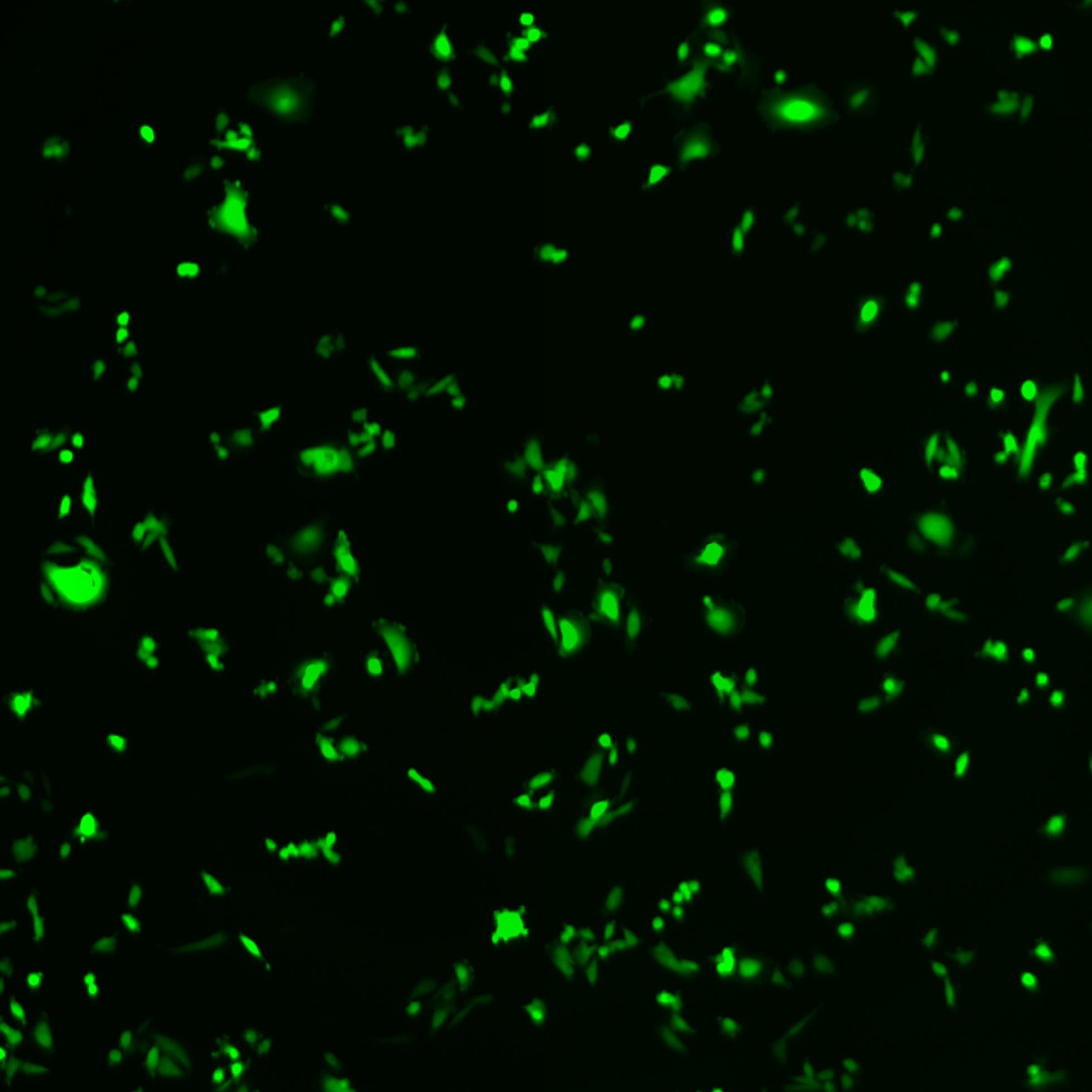

Supplement: Supplementary file 4 [file DataSheet_1.zip › Data Sheet 1/Fig2C/1-day3-Scrambled-AC009948.5.jpg]

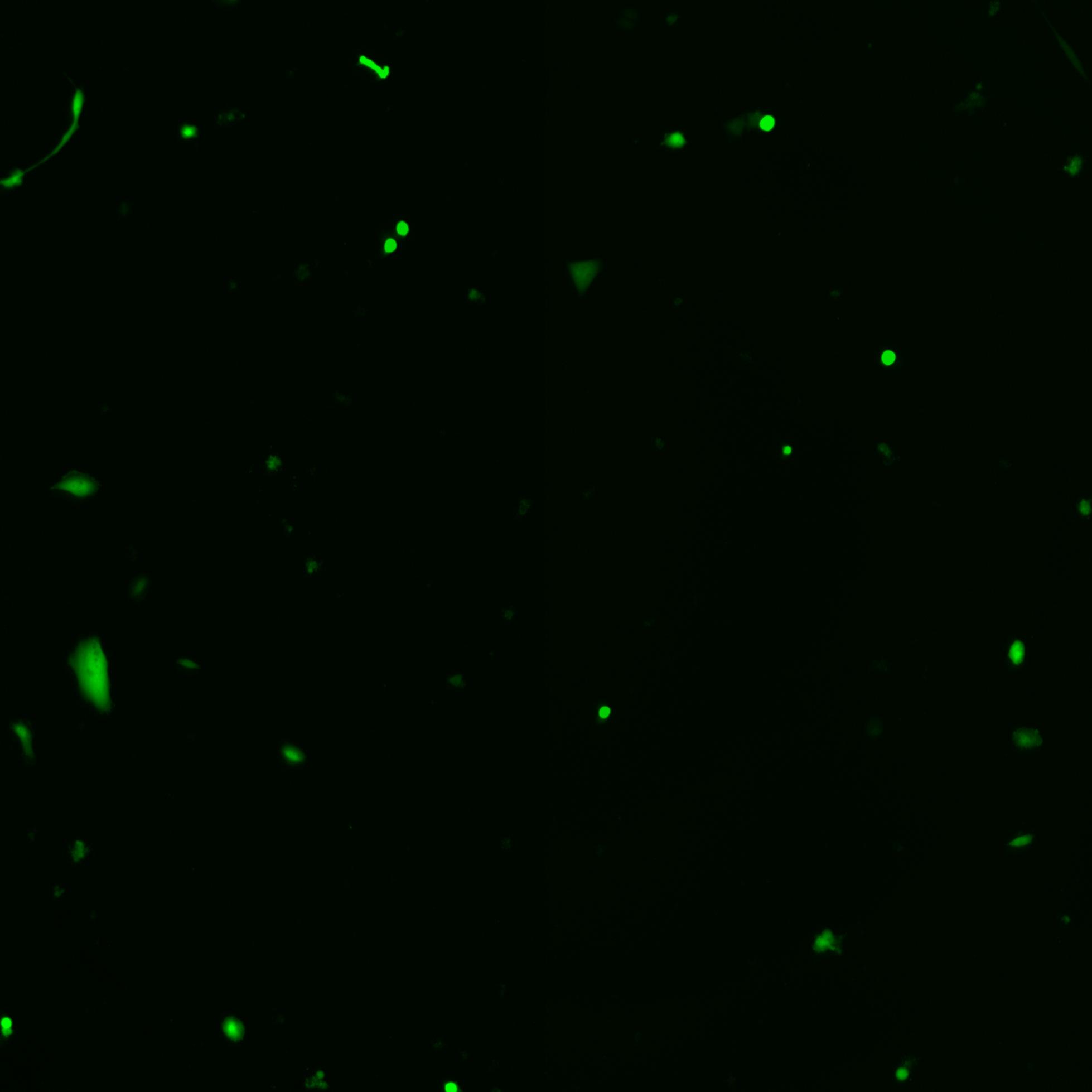

Supplement: Supplementary file 4 [file DataSheet_1.zip › Data Sheet 1/Fig2C/1-day3-siAP009948.5.jpg]

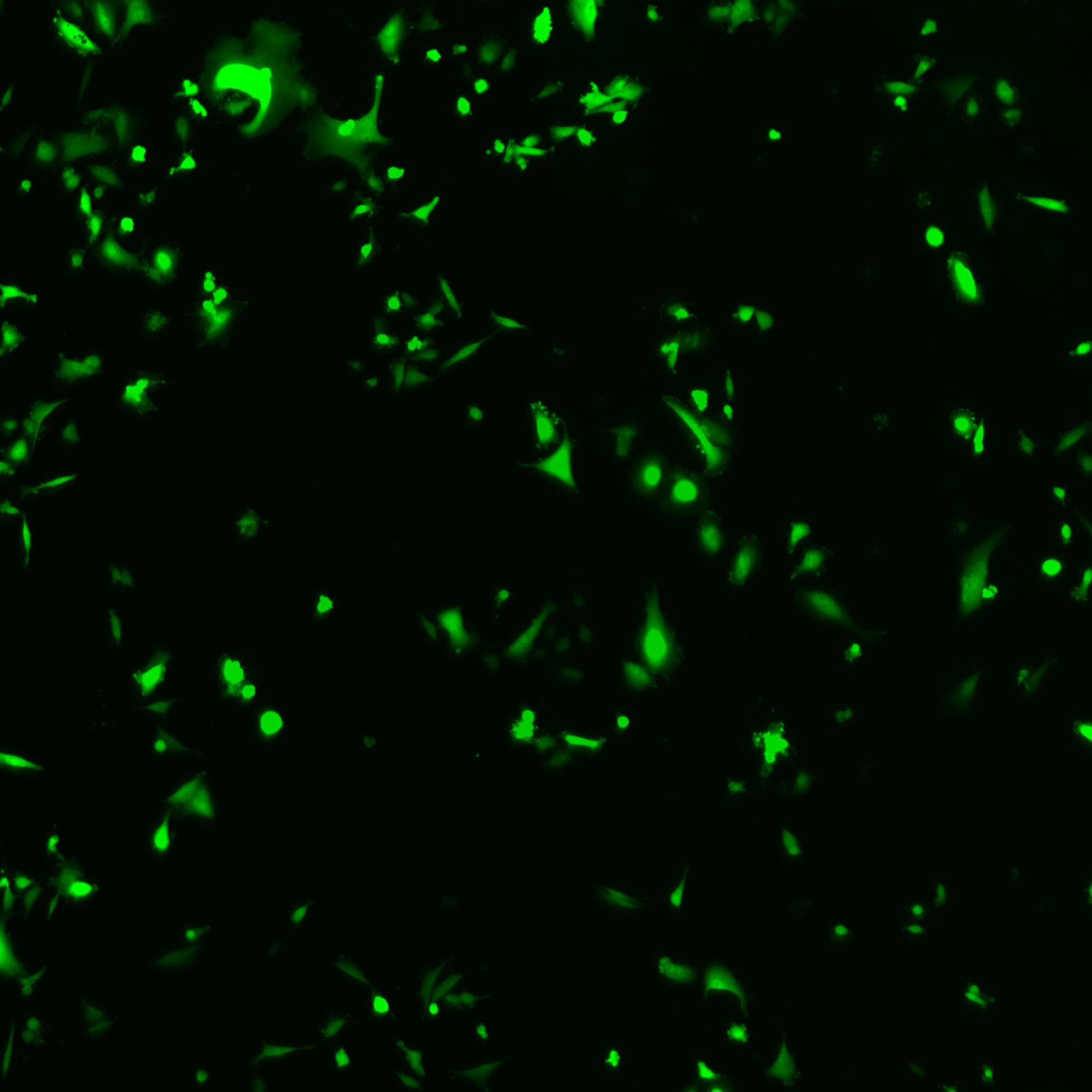

Supplement: Supplementary file 4 [file DataSheet_1.zip › Data Sheet 1/Fig2C/1-day4-NC-AC009948.5.jpg]

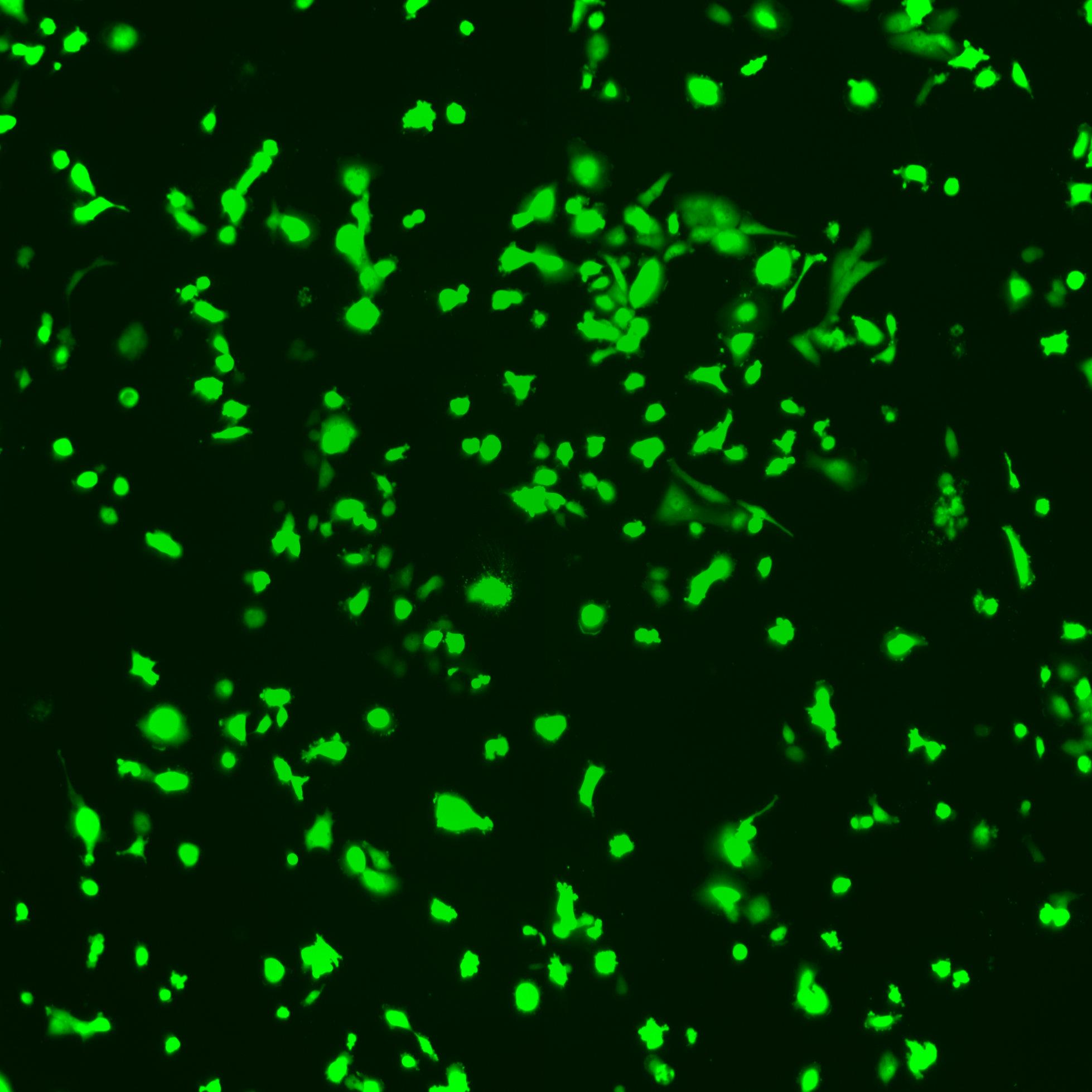

Supplement: Supplementary file 4 [file DataSheet_1.zip › Data Sheet 1/Fig2C/1-day4-overAC009948.5.jpg]

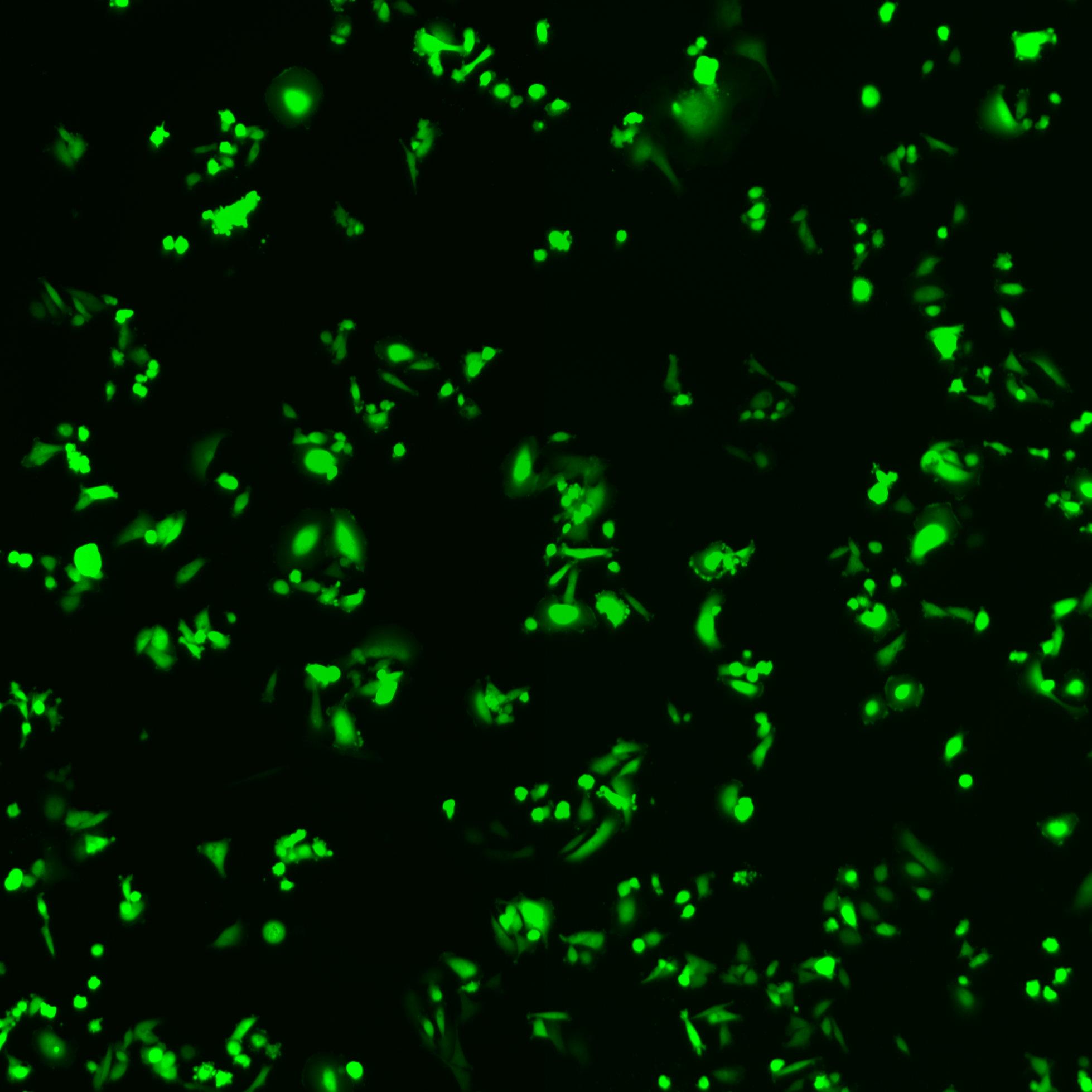

Supplement: Supplementary file 4 [file DataSheet_1.zip › Data Sheet 1/Fig2C/1-day4-Scrambled-AC009948.5.jpg]

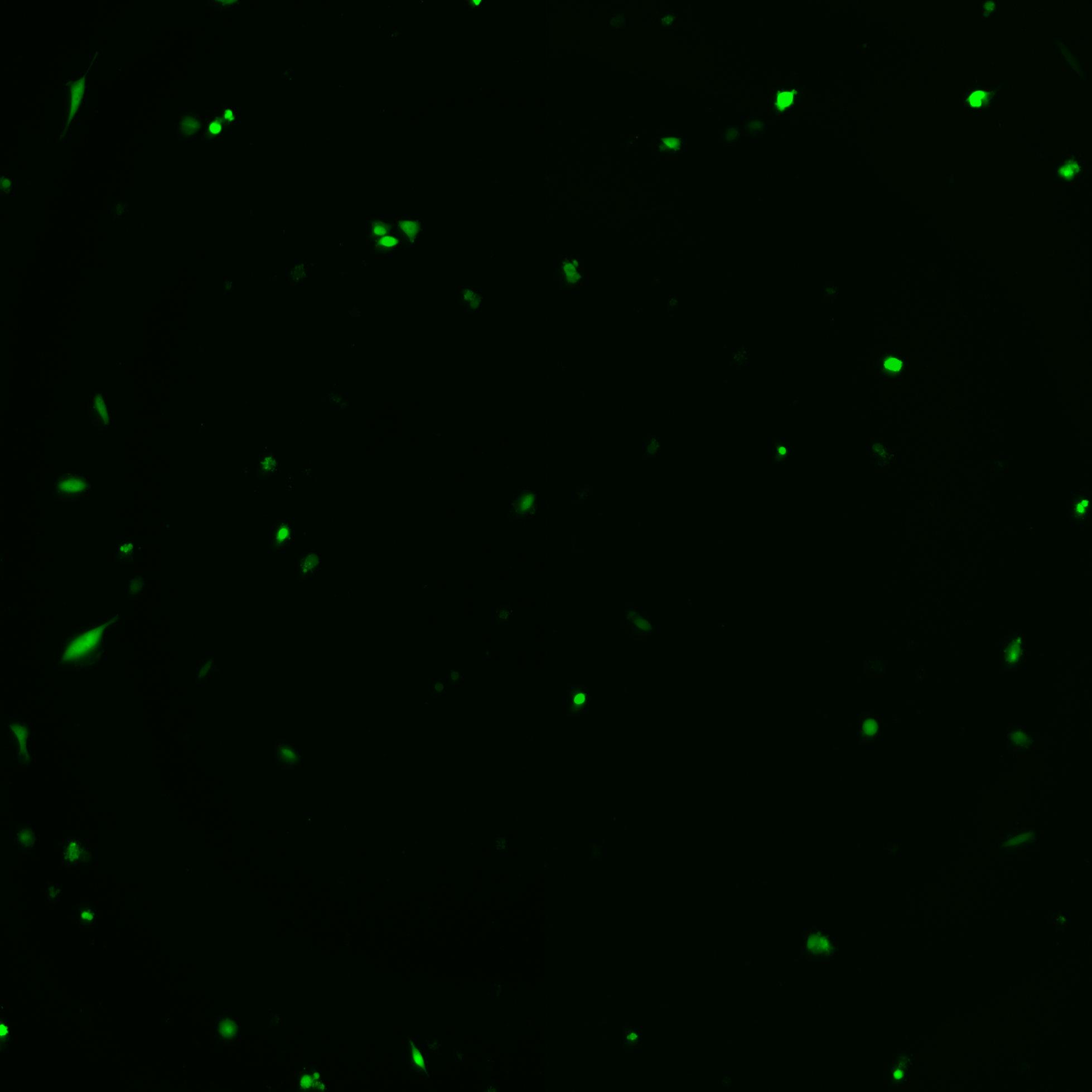

Supplement: Supplementary file 4 [file DataSheet_1.zip › Data Sheet 1/Fig2C/1-day4-siAP009948.5.jpg]

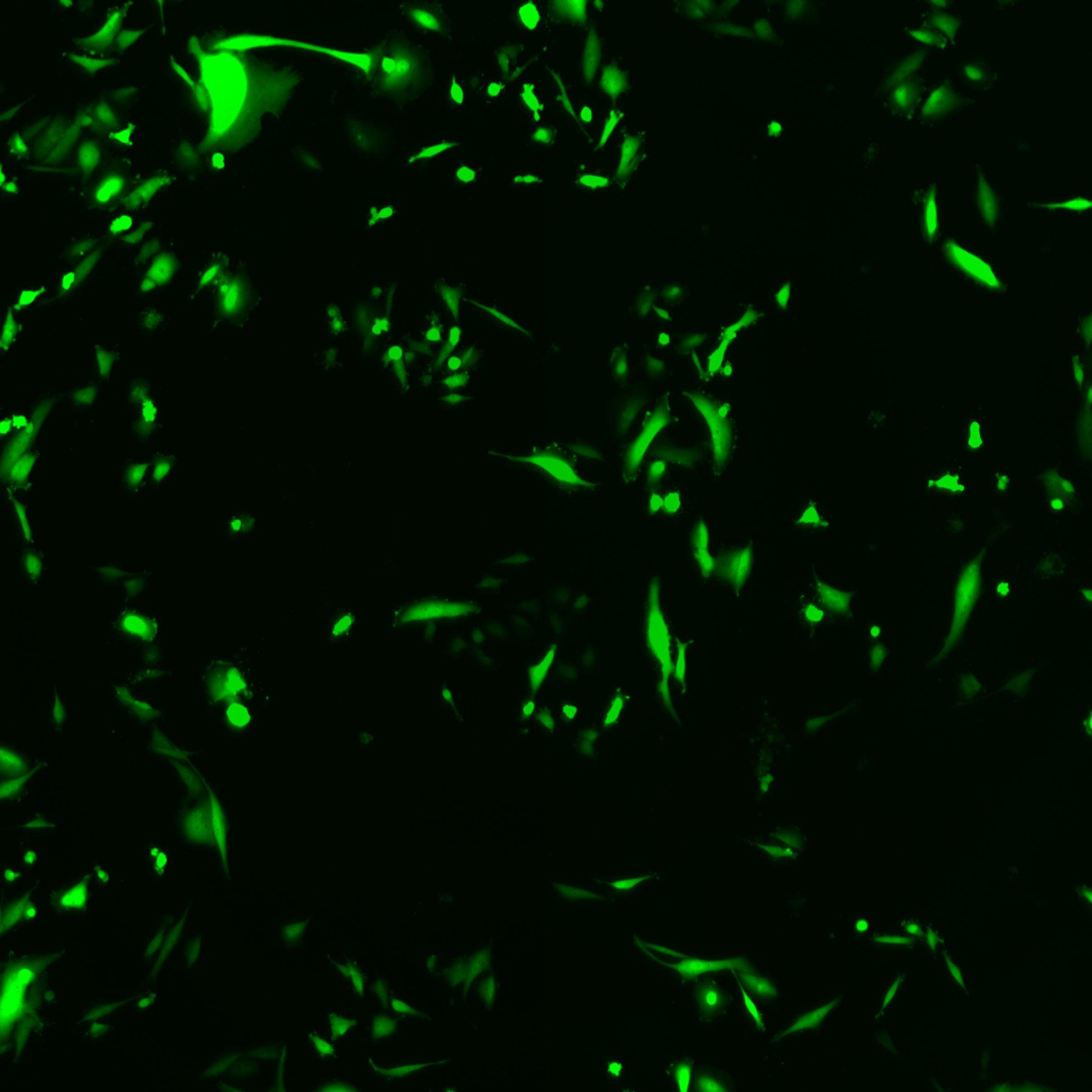

Supplement: Supplementary file 4 [file DataSheet_1.zip › Data Sheet 1/Fig2C/1-day5-NC-AC009948.5.jpg]

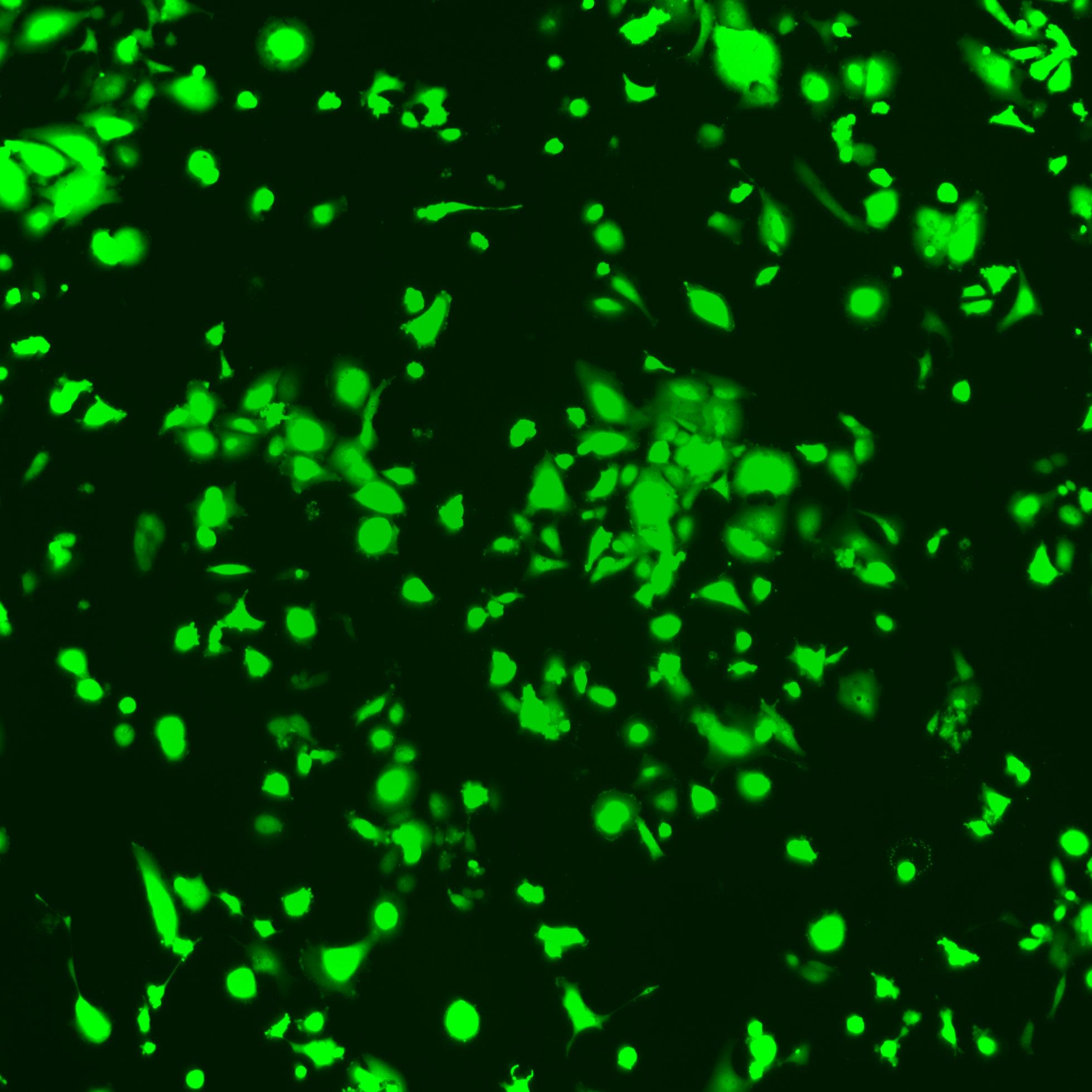

Supplement: Supplementary file 4 [file DataSheet_1.zip › Data Sheet 1/Fig2C/1-day5-overAC009948.5.jpg]

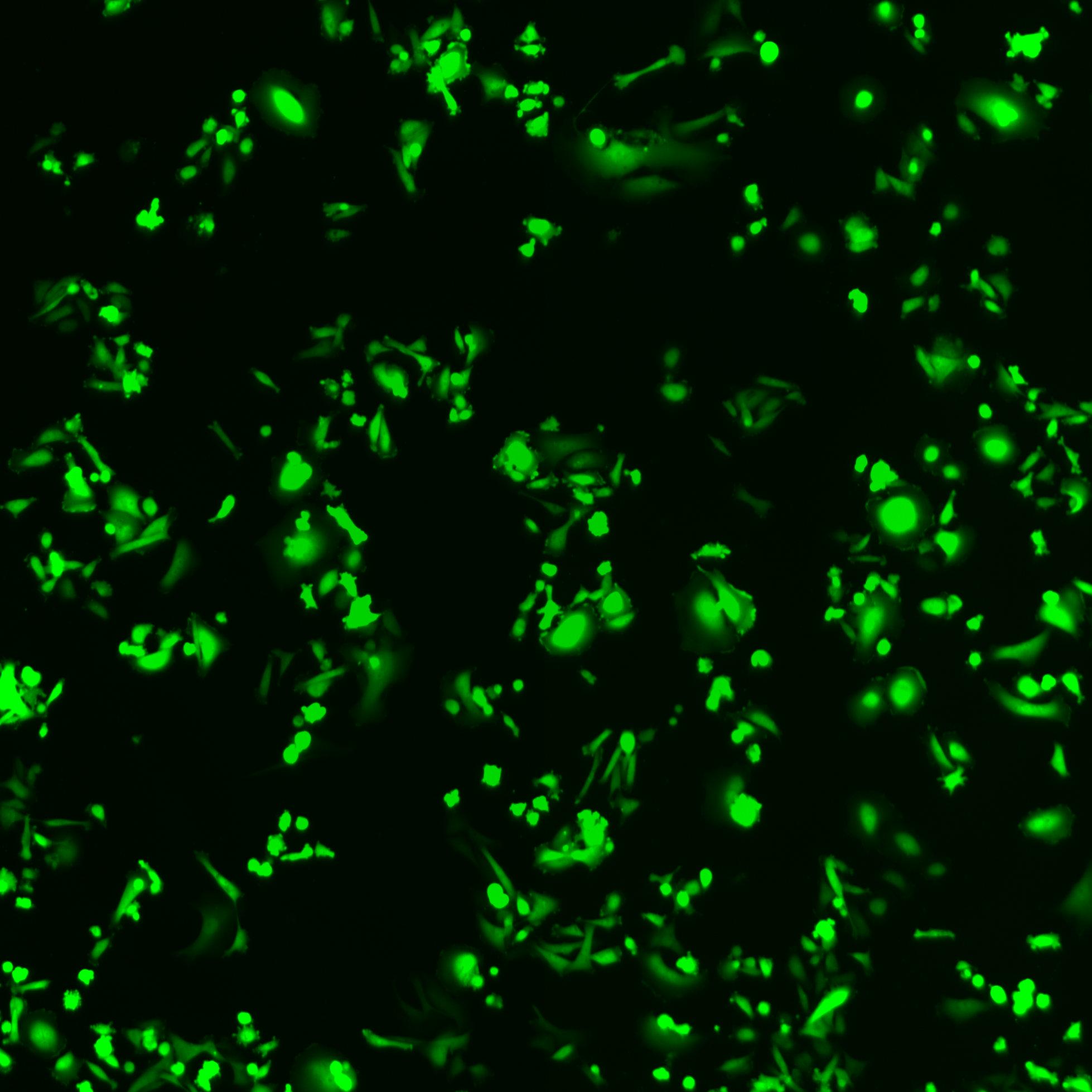

Supplement: Supplementary file 4 [file DataSheet_1.zip › Data Sheet 1/Fig2C/1-day5-Scrambled-AC009948.5.jpg]

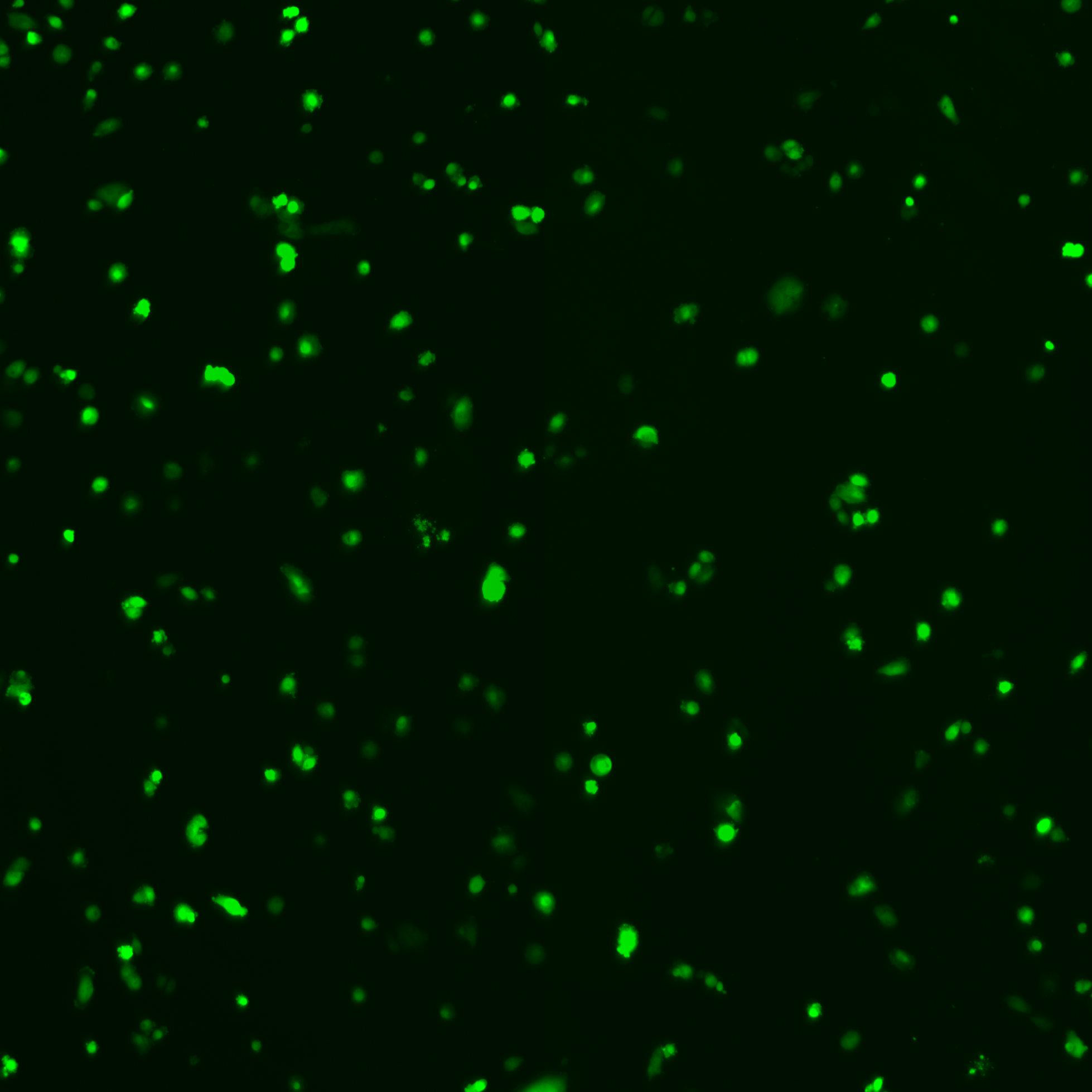

Supplement: Supplementary file 4 [file DataSheet_1.zip › Data Sheet 1/Fig2C/1-day5-siAC009948.5.jpg]

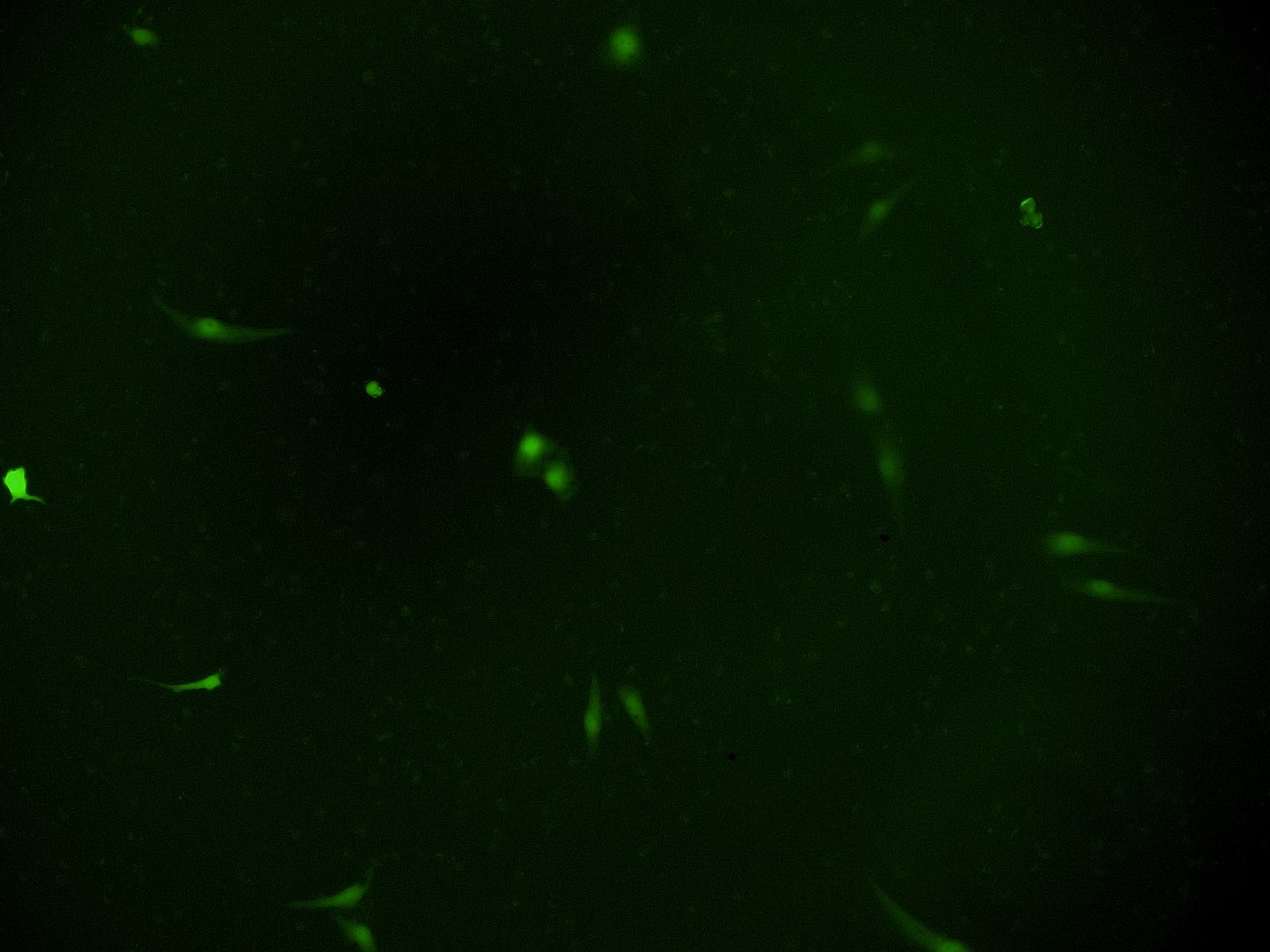

Supplement: Supplementary file 4 [file DataSheet_1.zip › Data Sheet 1/Fig2C/2-day1-NC-AC009948.5.jpg]

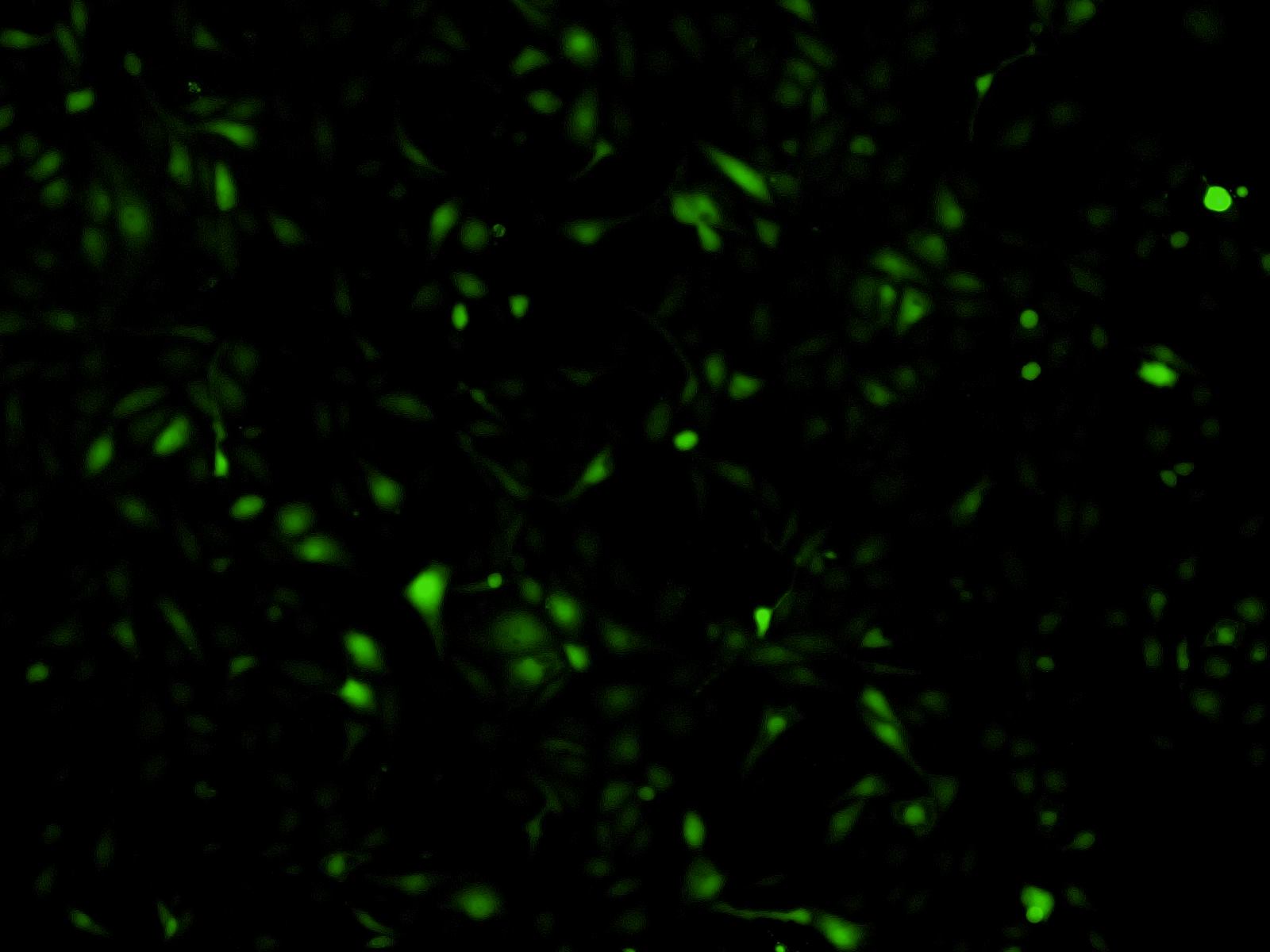

Supplement: Supplementary file 4 [file DataSheet_1.zip › Data Sheet 1/Fig2C/2-day1-overAC009948.5.jpg]

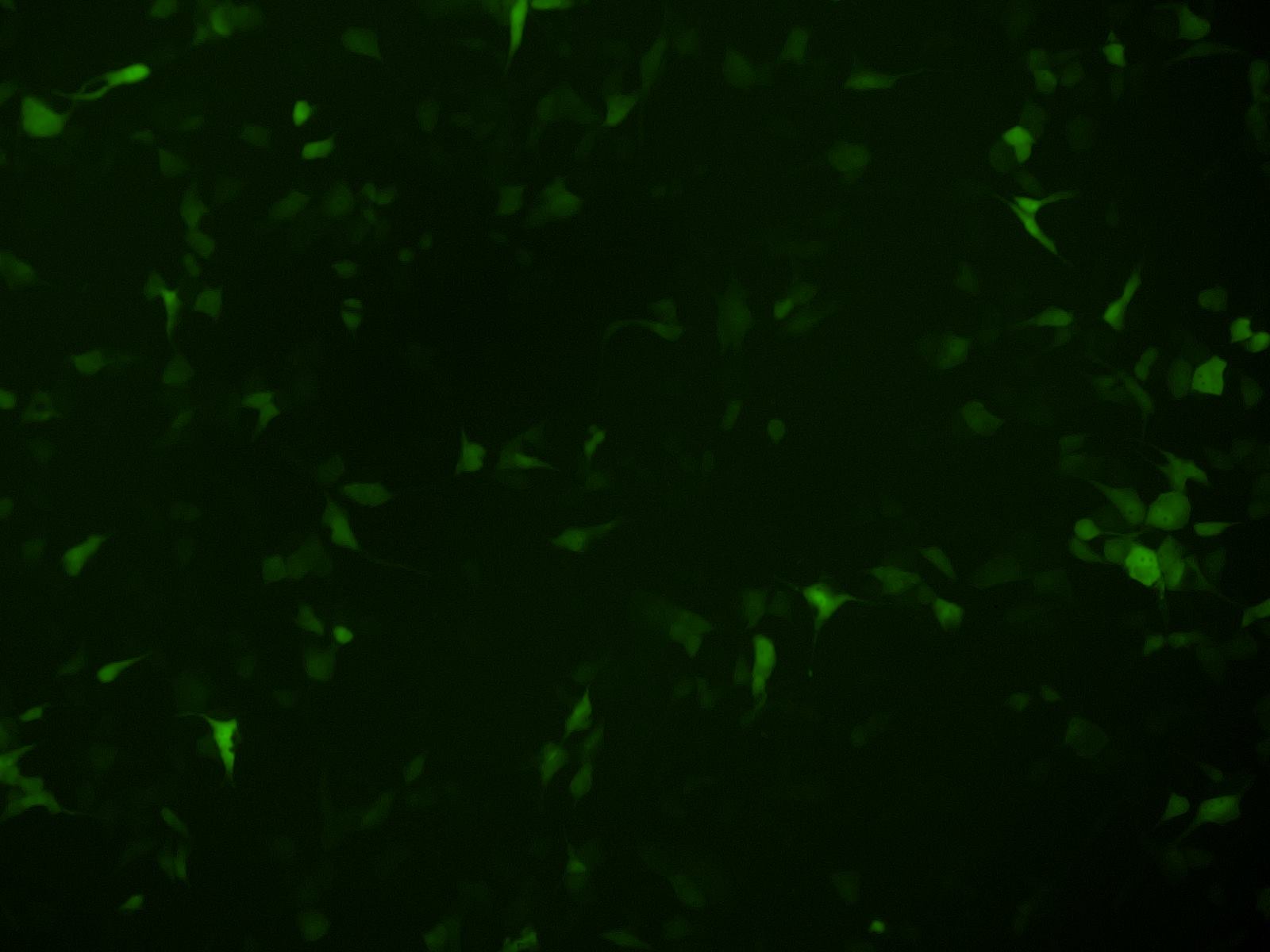

Supplement: Supplementary file 4 [file DataSheet_1.zip › Data Sheet 1/Fig2C/2-day1-Scrambled-AC009948.5.jpg]

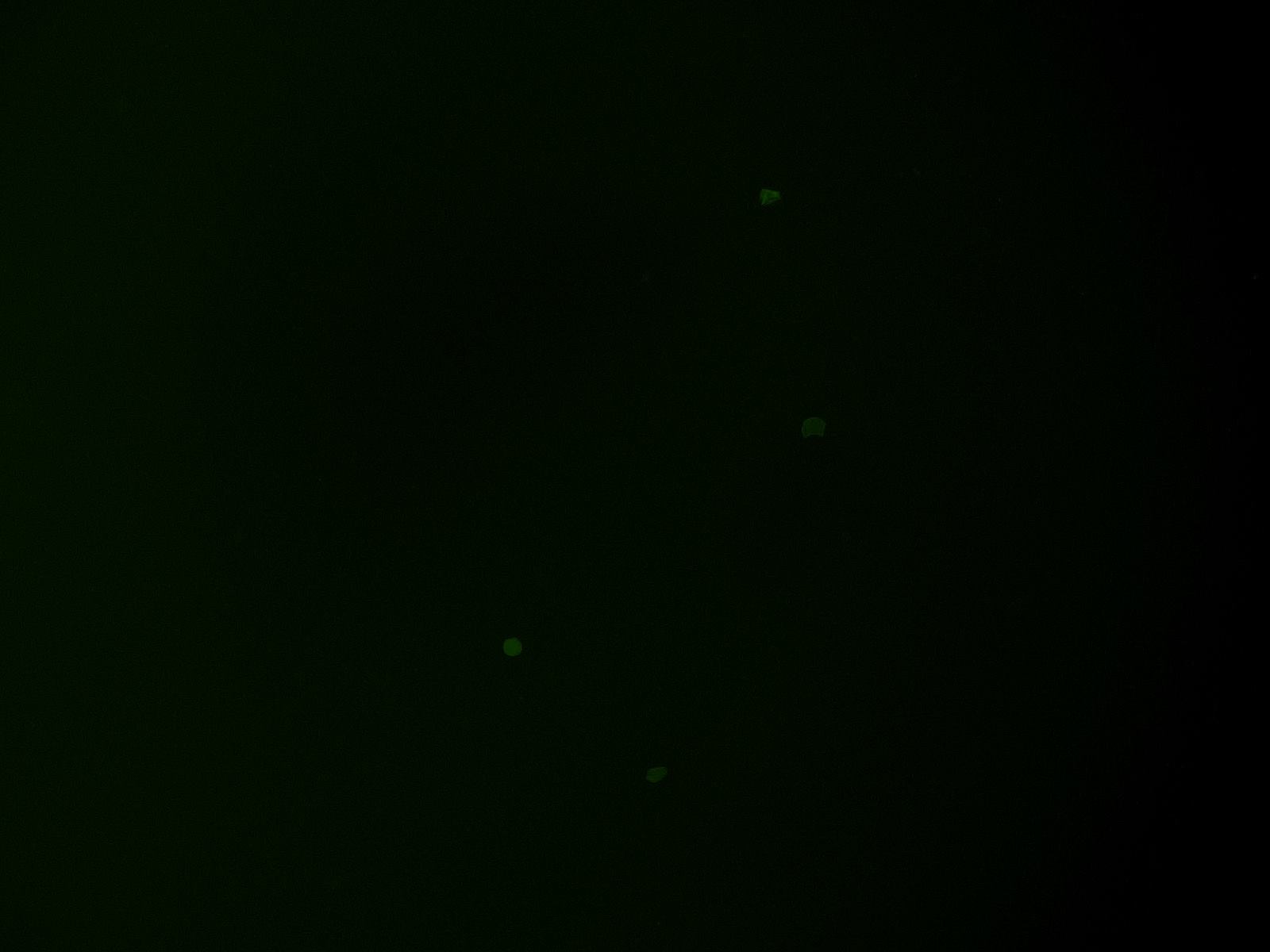

Supplement: Supplementary file 4 [file DataSheet_1.zip › Data Sheet 1/Fig2C/2-day1-siAC009948.5.jpg]

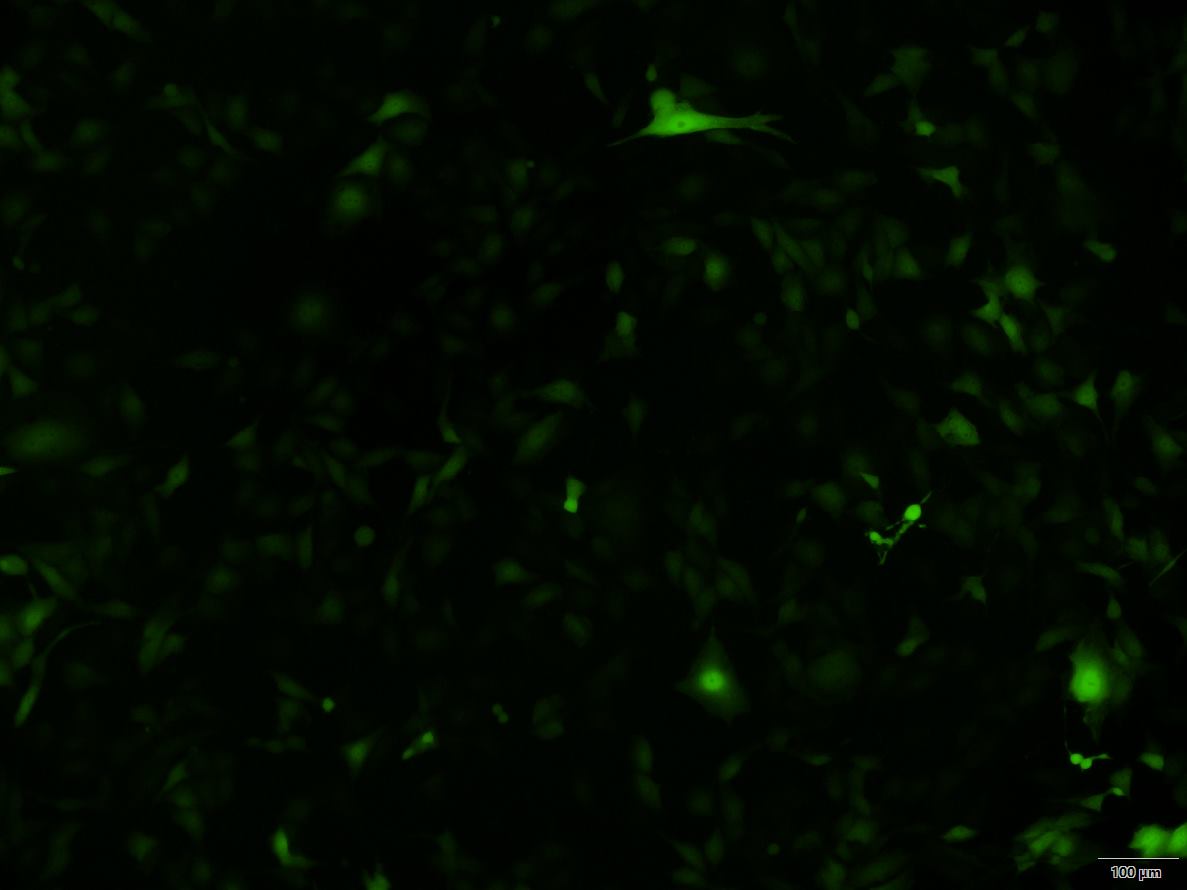

Supplement: Supplementary file 4 [file DataSheet_1.zip › Data Sheet 1/Fig2C/2-day2-NC-AC009948.5.jpg]

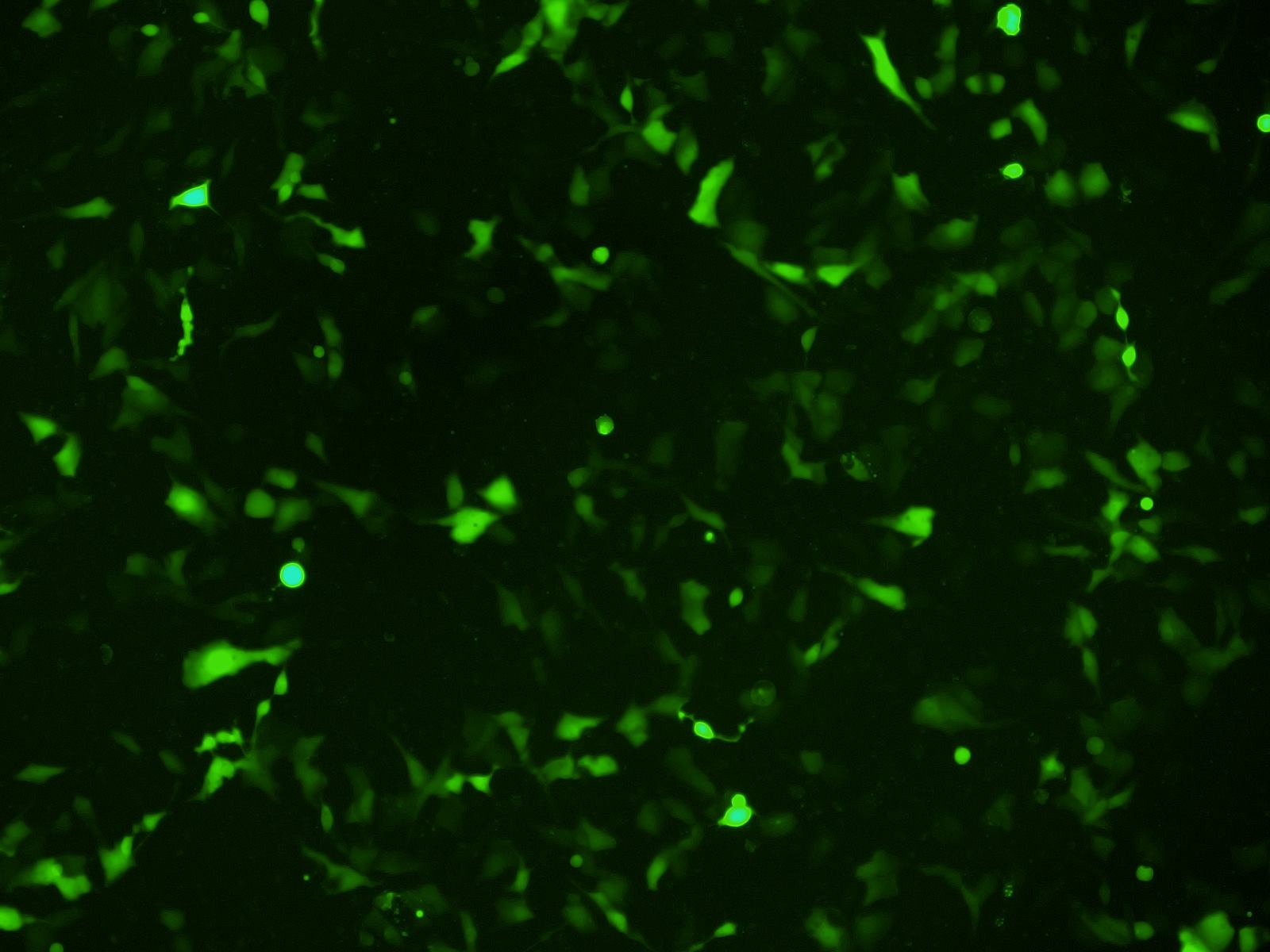

Supplement: Supplementary file 4 [file DataSheet_1.zip › Data Sheet 1/Fig2C/2-day2-overAC009948.5.jpg]

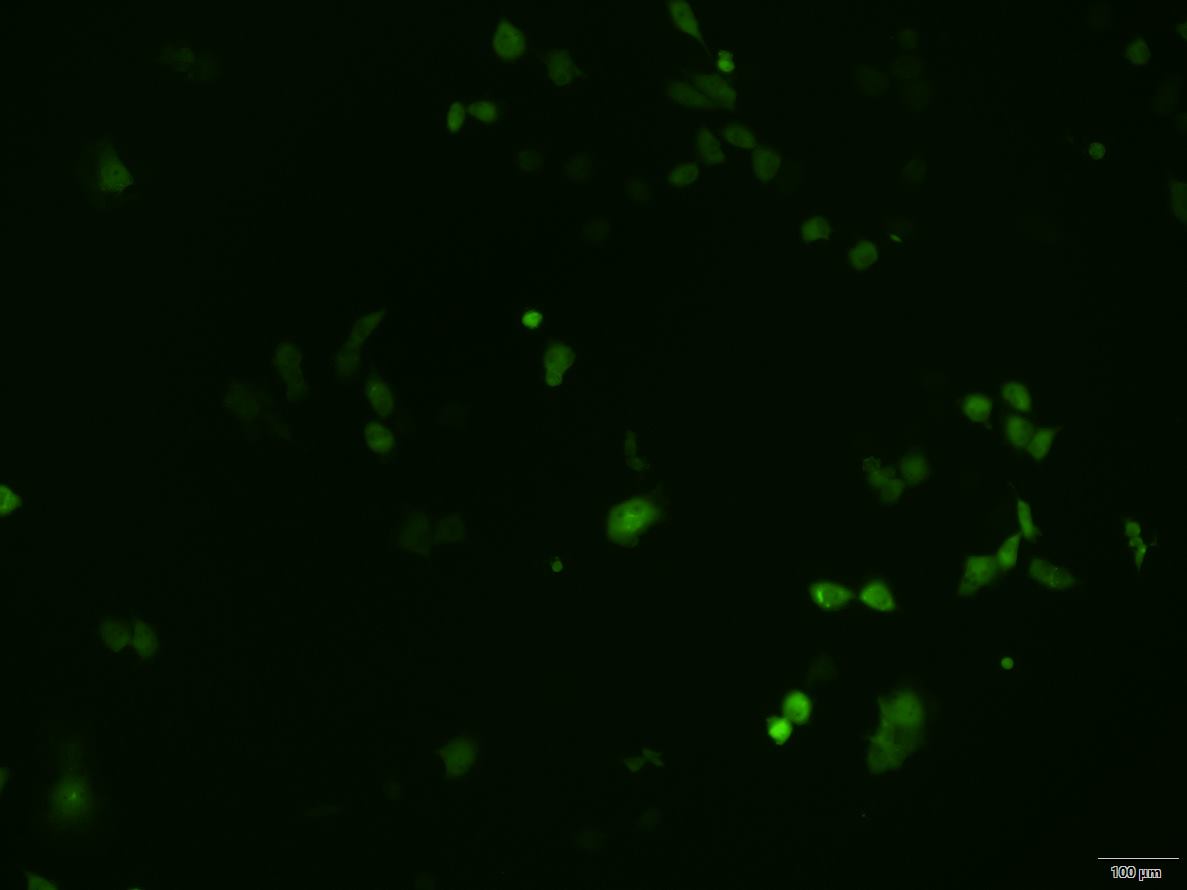

Supplement: Supplementary file 4 [file DataSheet_1.zip › Data Sheet 1/Fig2C/2-day2-Scrambled-AC009948.5.jpg]

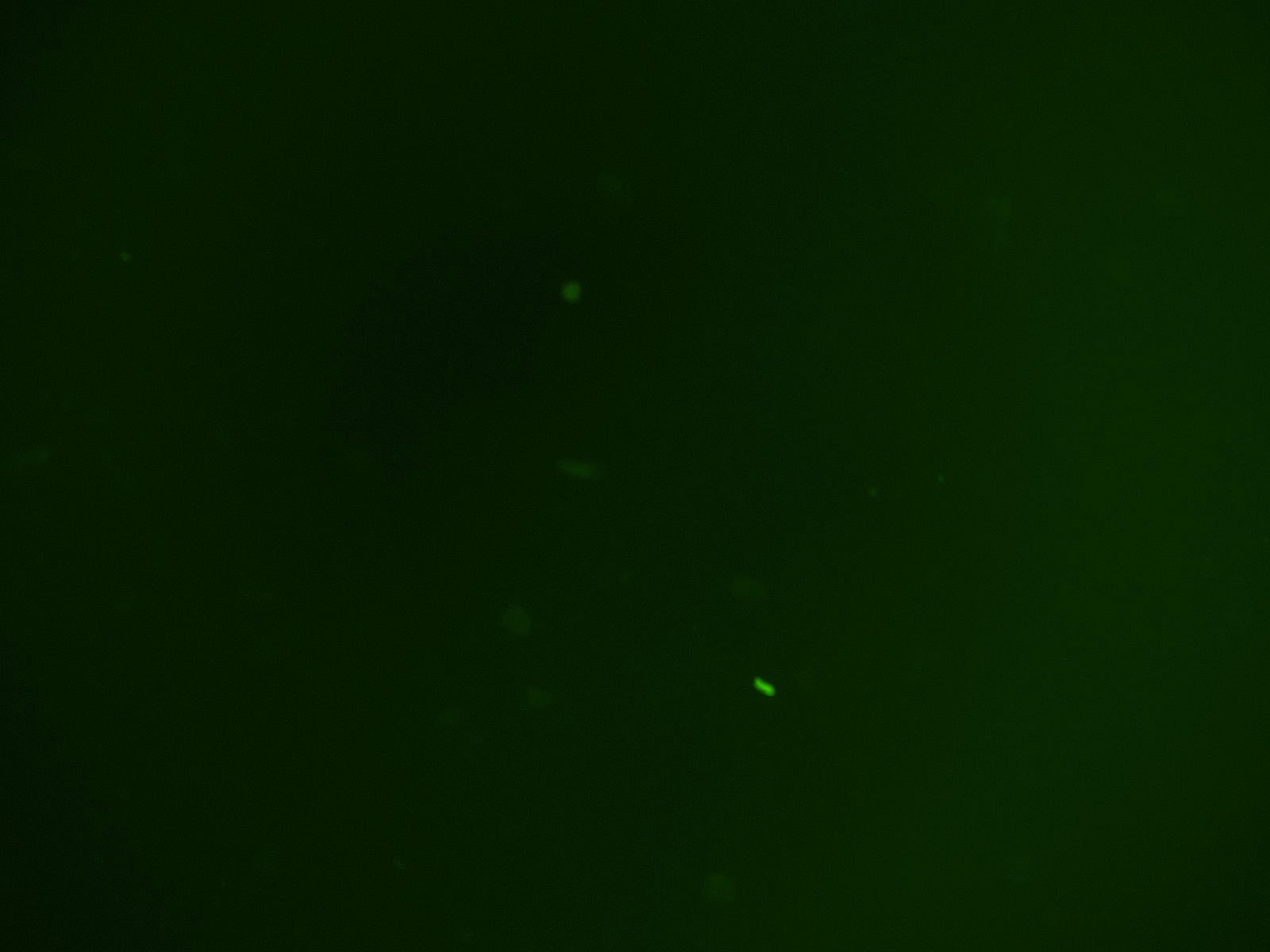

Supplement: Supplementary file 4 [file DataSheet_1.zip › Data Sheet 1/Fig2C/2-day2-siAC009948.5.jpg]

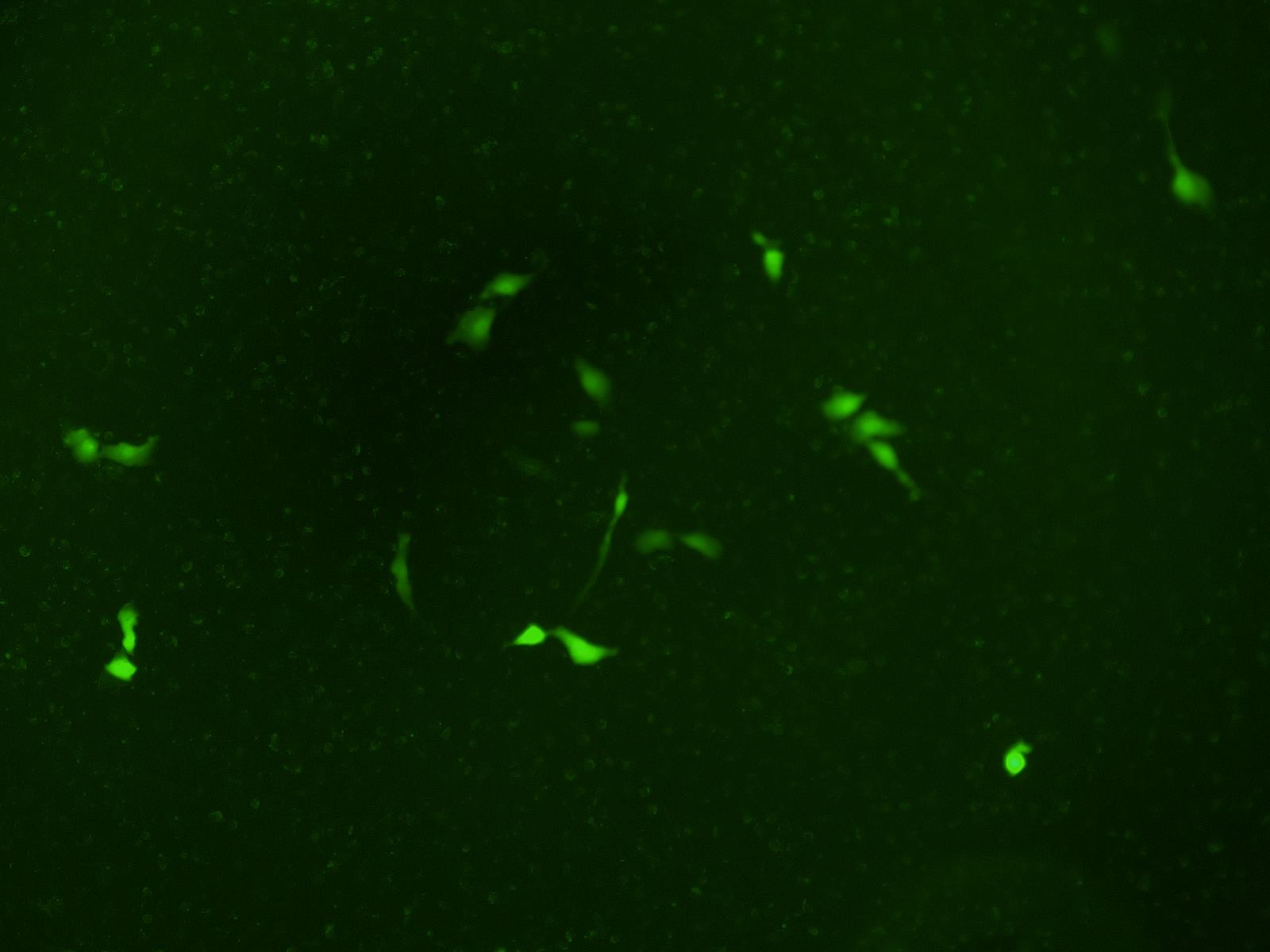

Supplement: Supplementary file 4 [file DataSheet_1.zip › Data Sheet 1/Fig2C/2-day3-NC-AC009948.5.jpg]

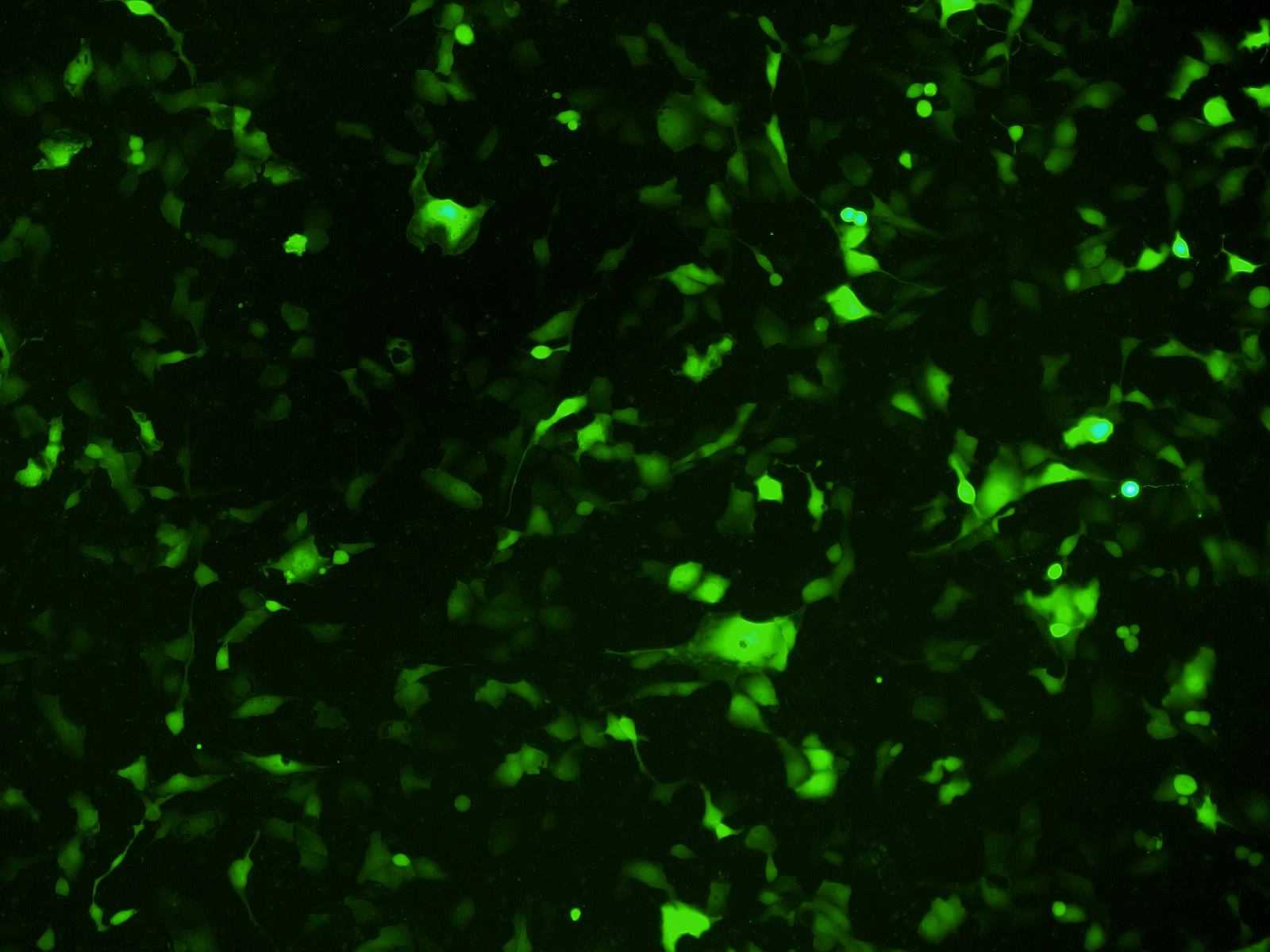

Supplement: Supplementary file 4 [file DataSheet_1.zip › Data Sheet 1/Fig2C/2-day3-overAC009948.5.jpg]

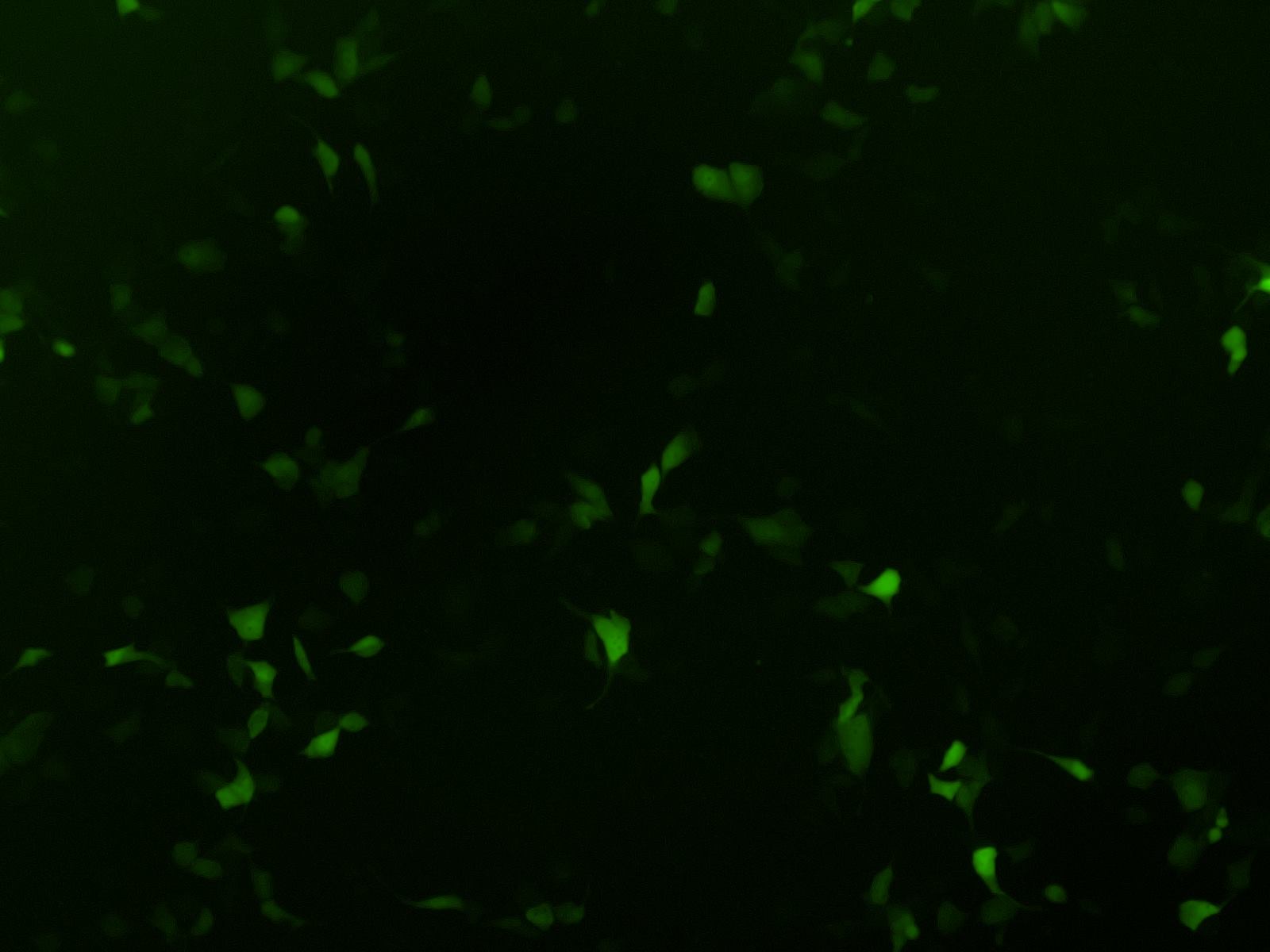

Supplement: Supplementary file 4 [file DataSheet_1.zip › Data Sheet 1/Fig2C/2-day3-Scrambled-AC009948.5.jpg]

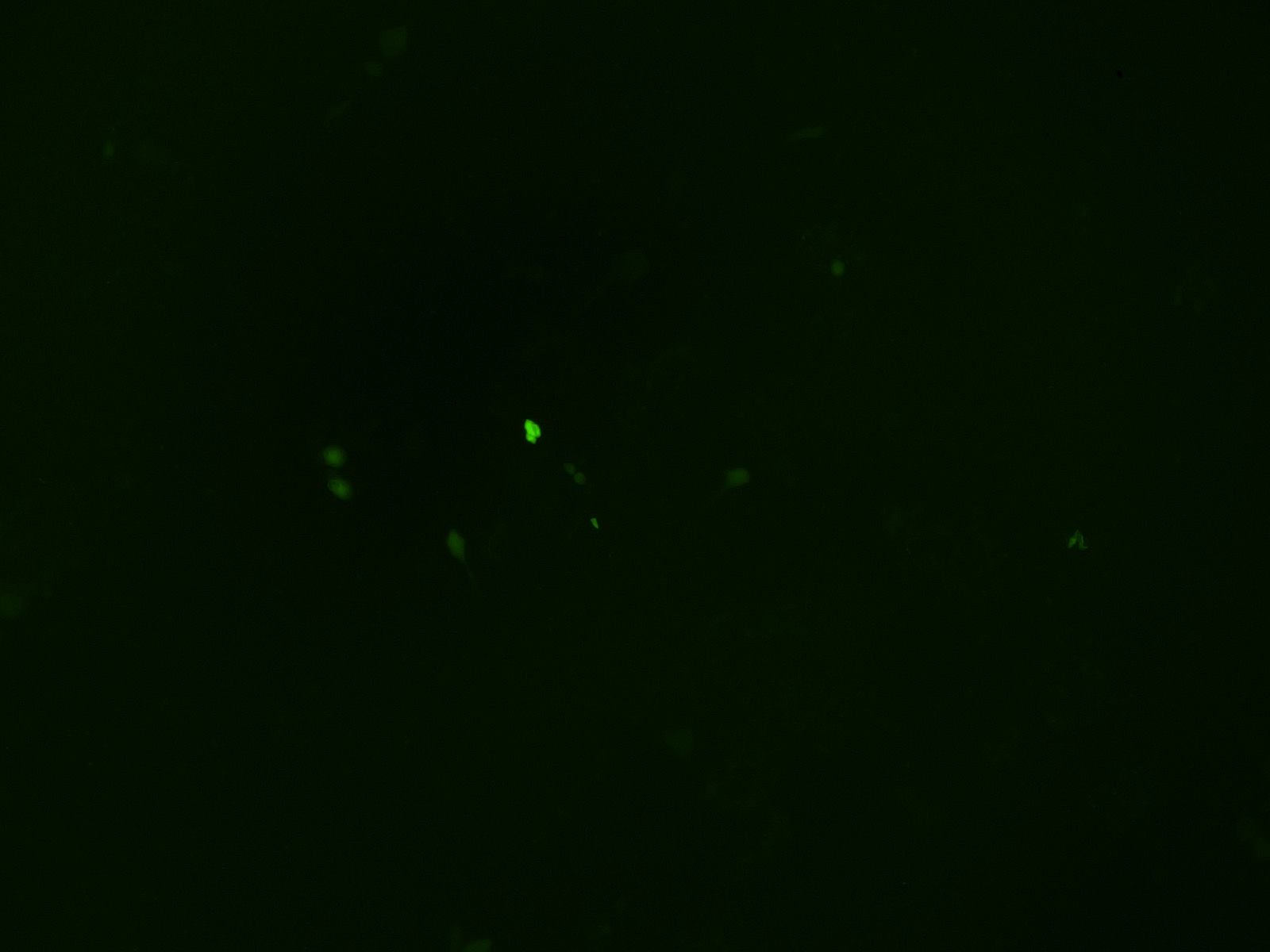

Supplement: Supplementary file 4 [file DataSheet_1.zip › Data Sheet 1/Fig2C/2-day3-siAC009948.5.jpg]

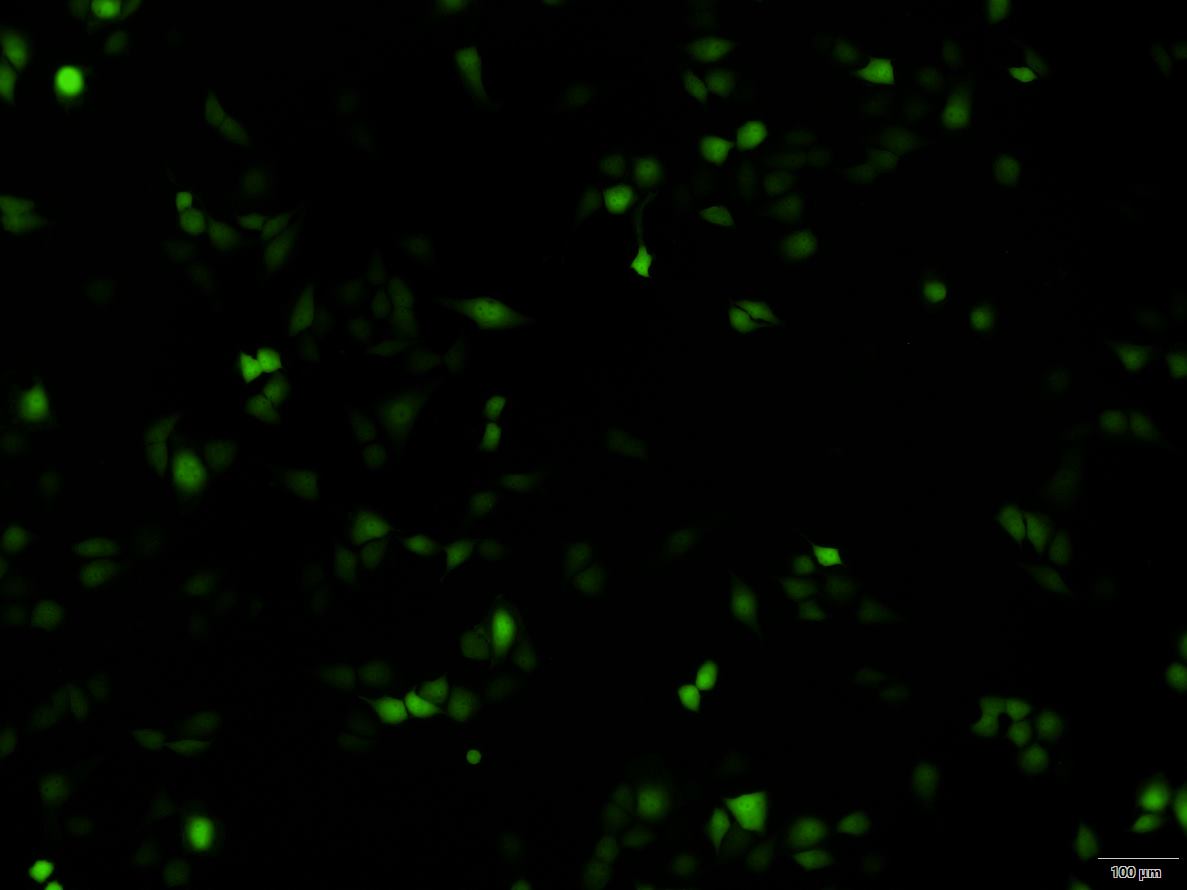

Supplement: Supplementary file 4 [file DataSheet_1.zip › Data Sheet 1/Fig2C/2-day4-NC-AC009948.5.jpg]

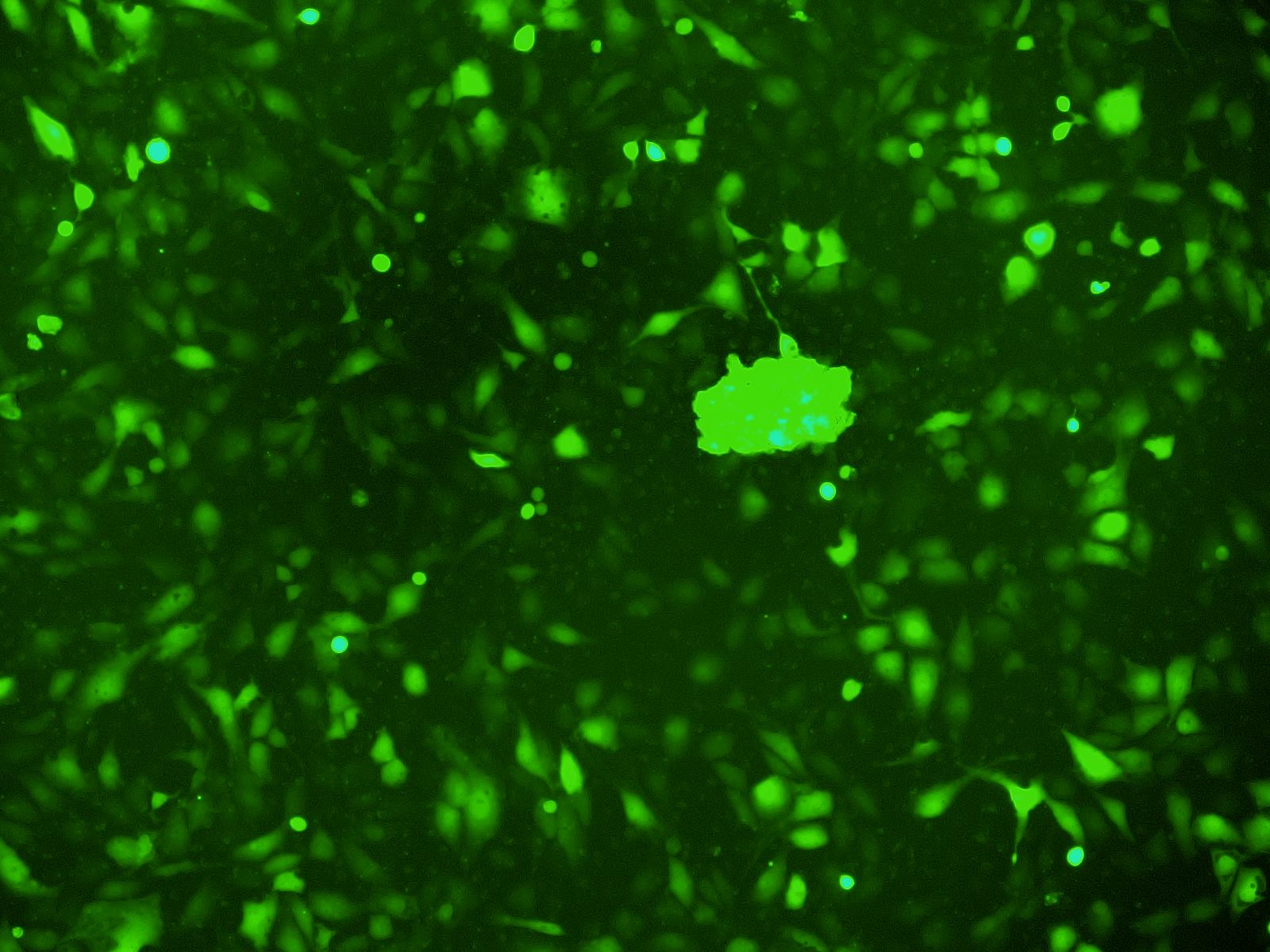

Supplement: Supplementary file 4 [file DataSheet_1.zip › Data Sheet 1/Fig2C/2-day4-overAC009948.5.jpg]

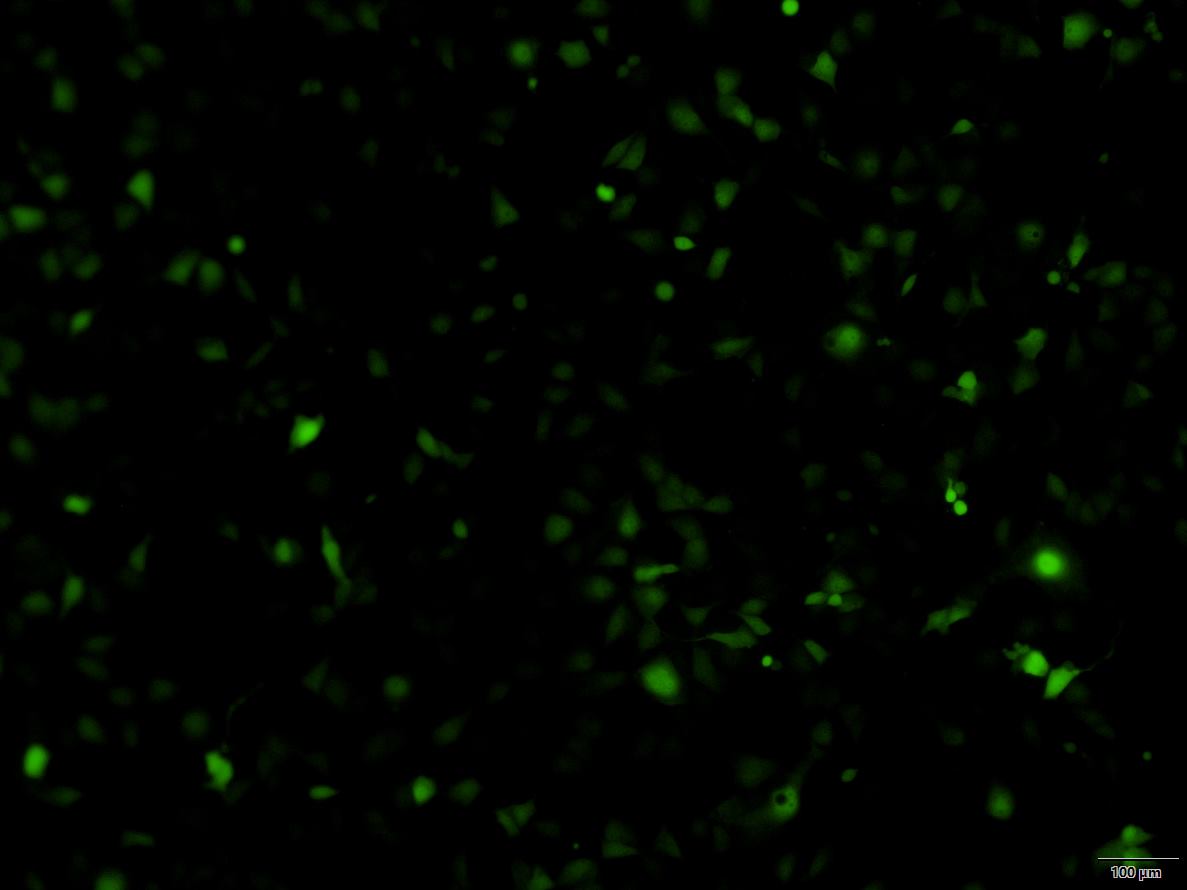

Supplement: Supplementary file 4 [file DataSheet_1.zip › Data Sheet 1/Fig2C/2-day4-Scrambled-AC009948.5.jpg]

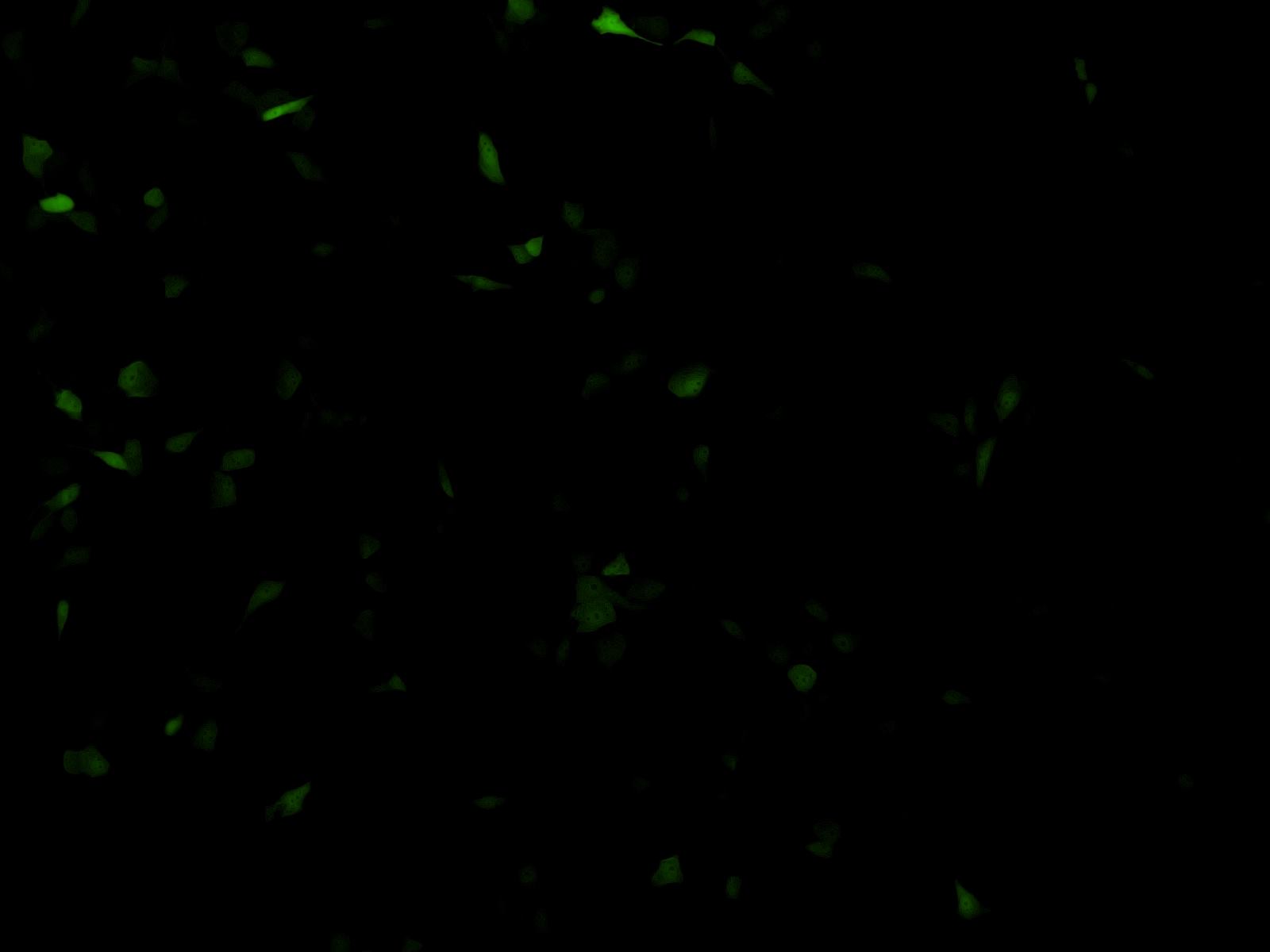

Supplement: Supplementary file 4 [file DataSheet_1.zip › Data Sheet 1/Fig2C/2-day4-siAC009948.5.jpg]

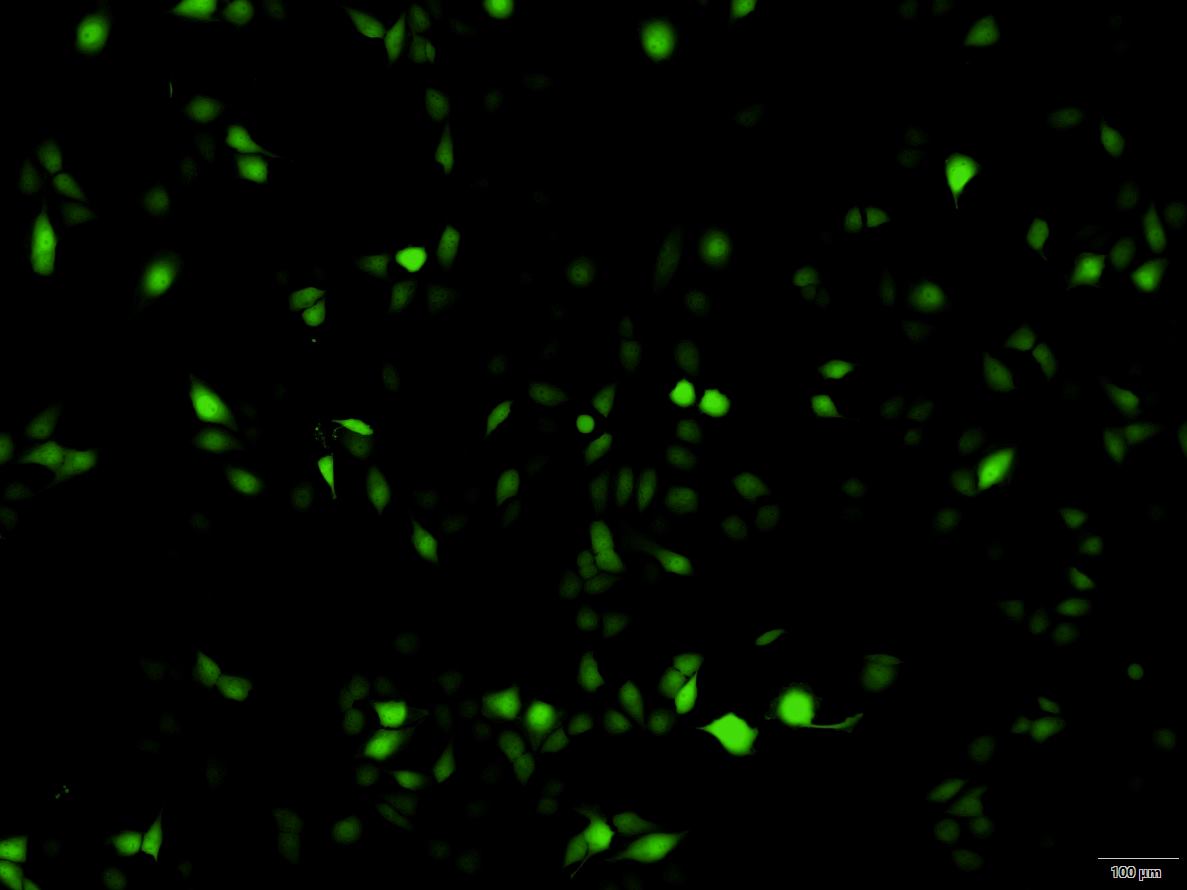

Supplement: Supplementary file 4 [file DataSheet_1.zip › Data Sheet 1/Fig2C/2-day5-NC-AC009948.5.jpg]

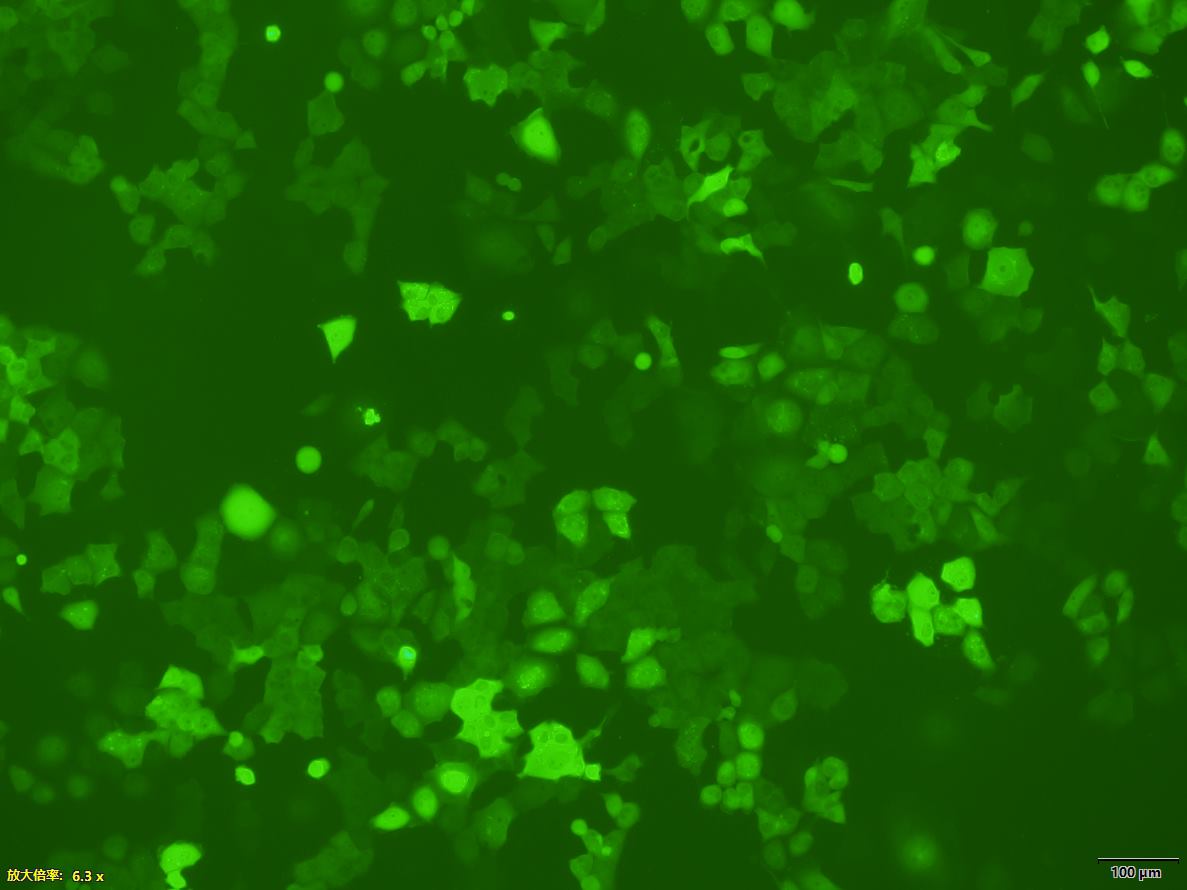

Supplement: Supplementary file 4 [file DataSheet_1.zip › Data Sheet 1/Fig2C/2-day5-overAC009948.5.jpg]

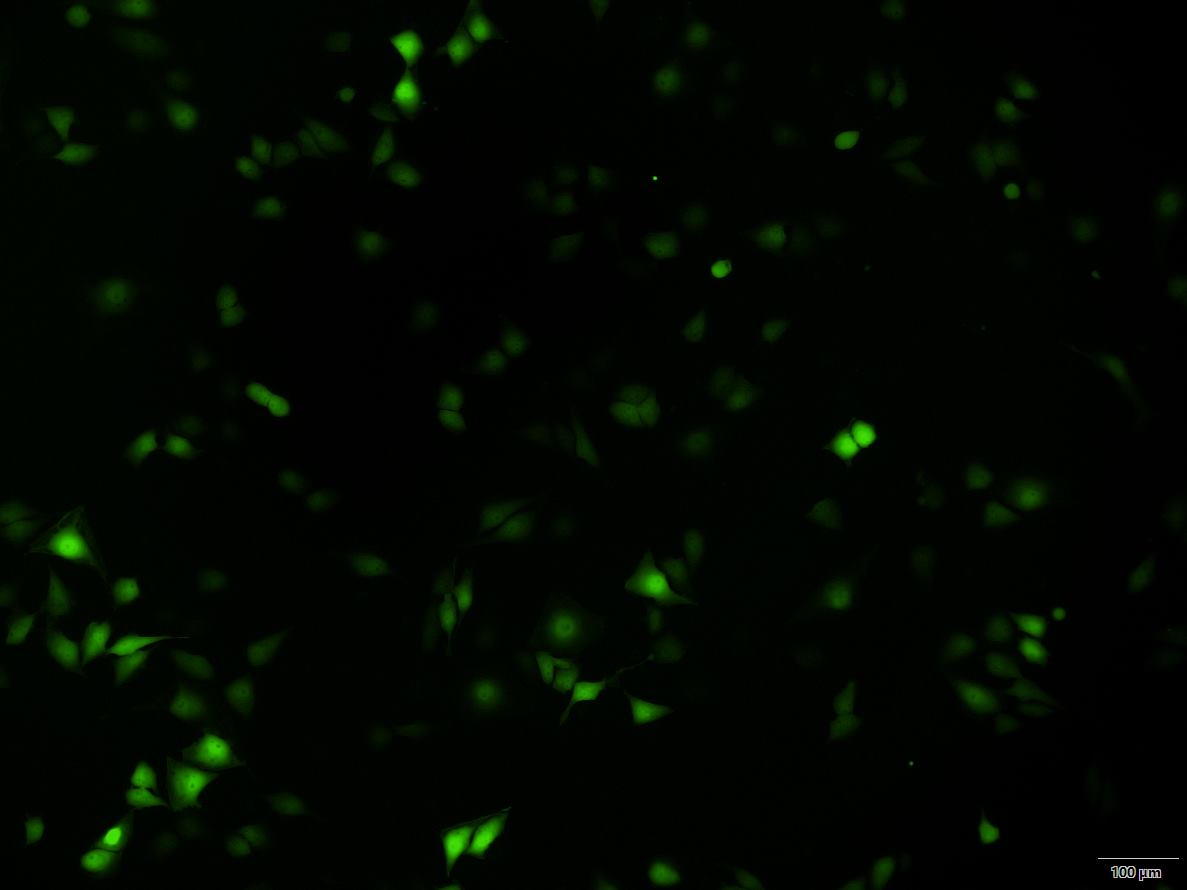

Supplement: Supplementary file 4 [file DataSheet_1.zip › Data Sheet 1/Fig2C/2-day5-Scrambled-AC009948.5.jpg]

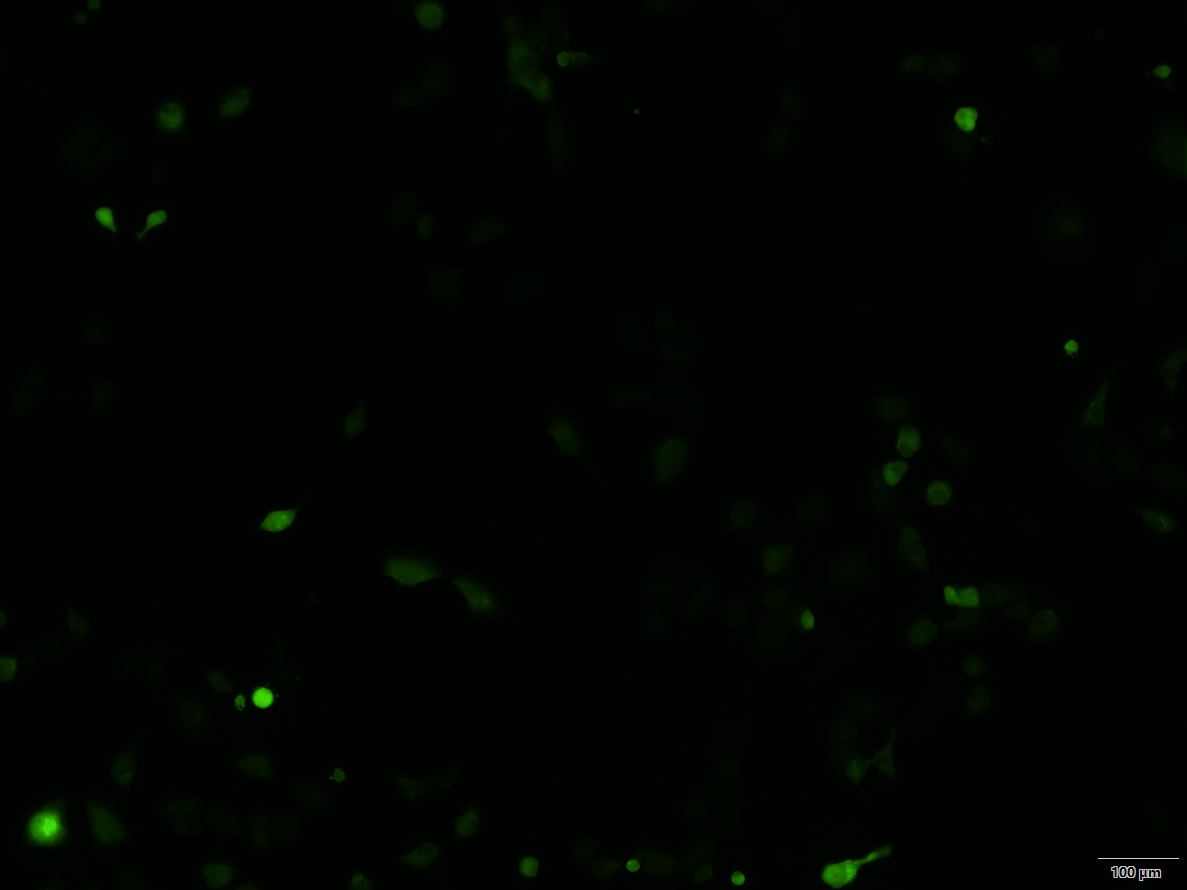

Supplement: Supplementary file 4 [file DataSheet_1.zip › Data Sheet 1/Fig2C/2-day5-siAC009948.5.jpg]

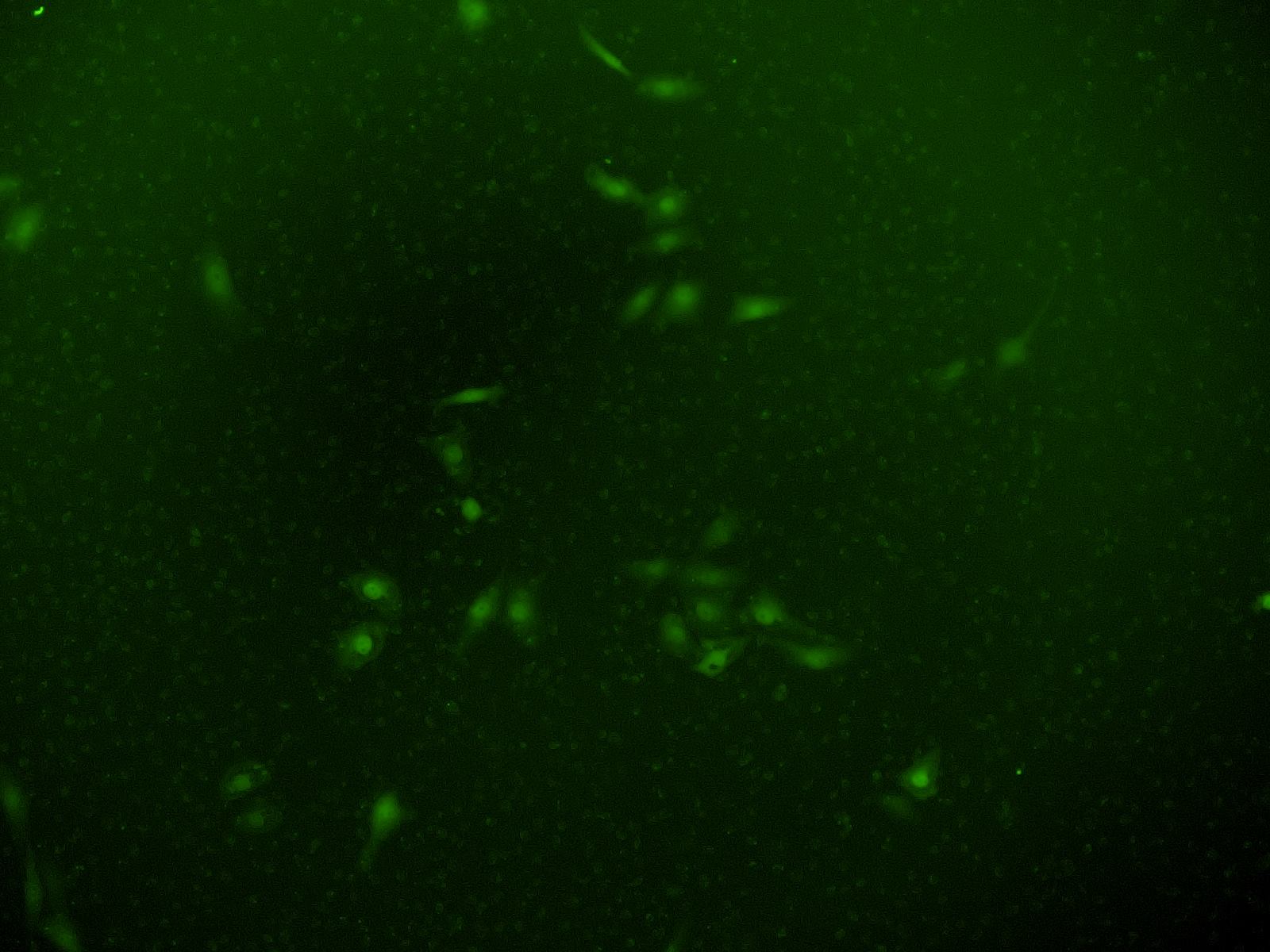

Supplement: Supplementary file 4 [file DataSheet_1.zip › Data Sheet 1/Fig2C/3-day1-NC-AC009948.5.jpg]

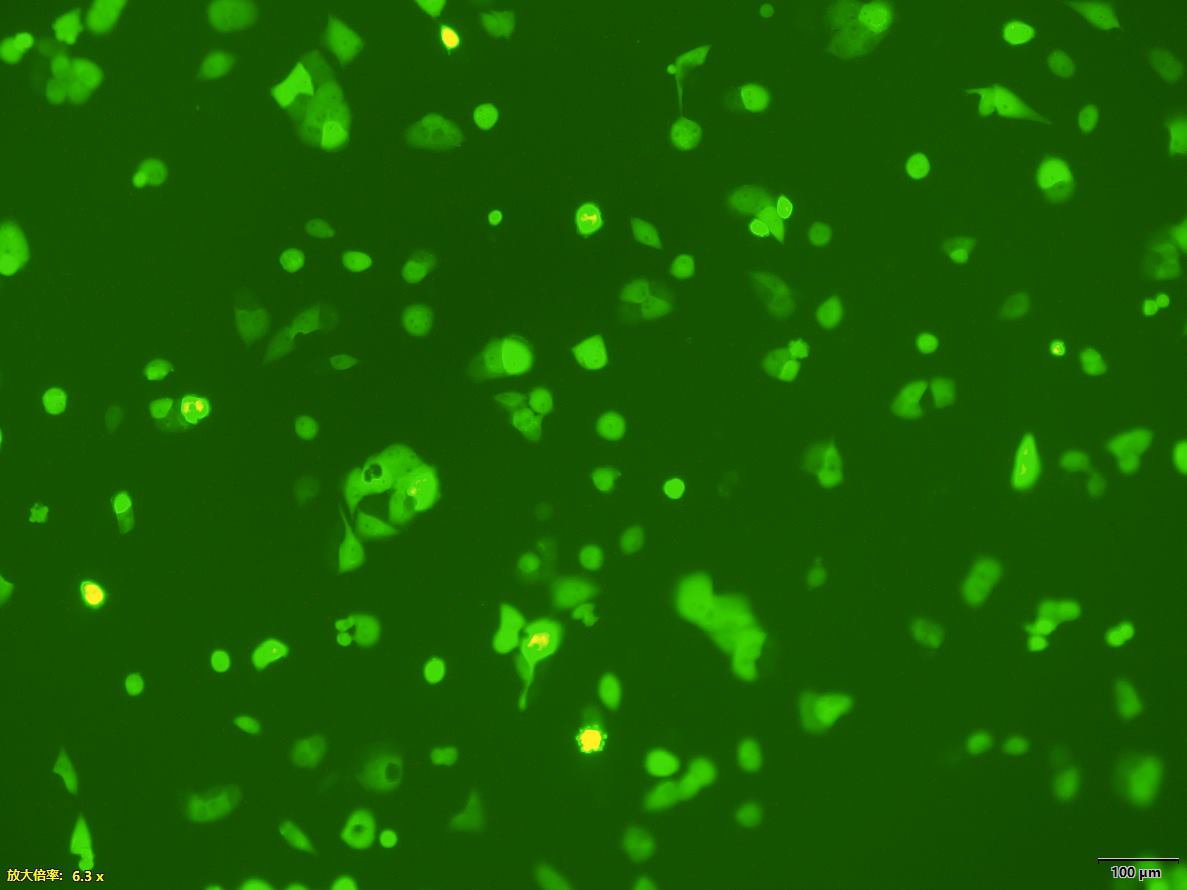

Supplement: Supplementary file 4 [file DataSheet_1.zip › Data Sheet 1/Fig2C/3-day1-overAC009948.5.jpg]

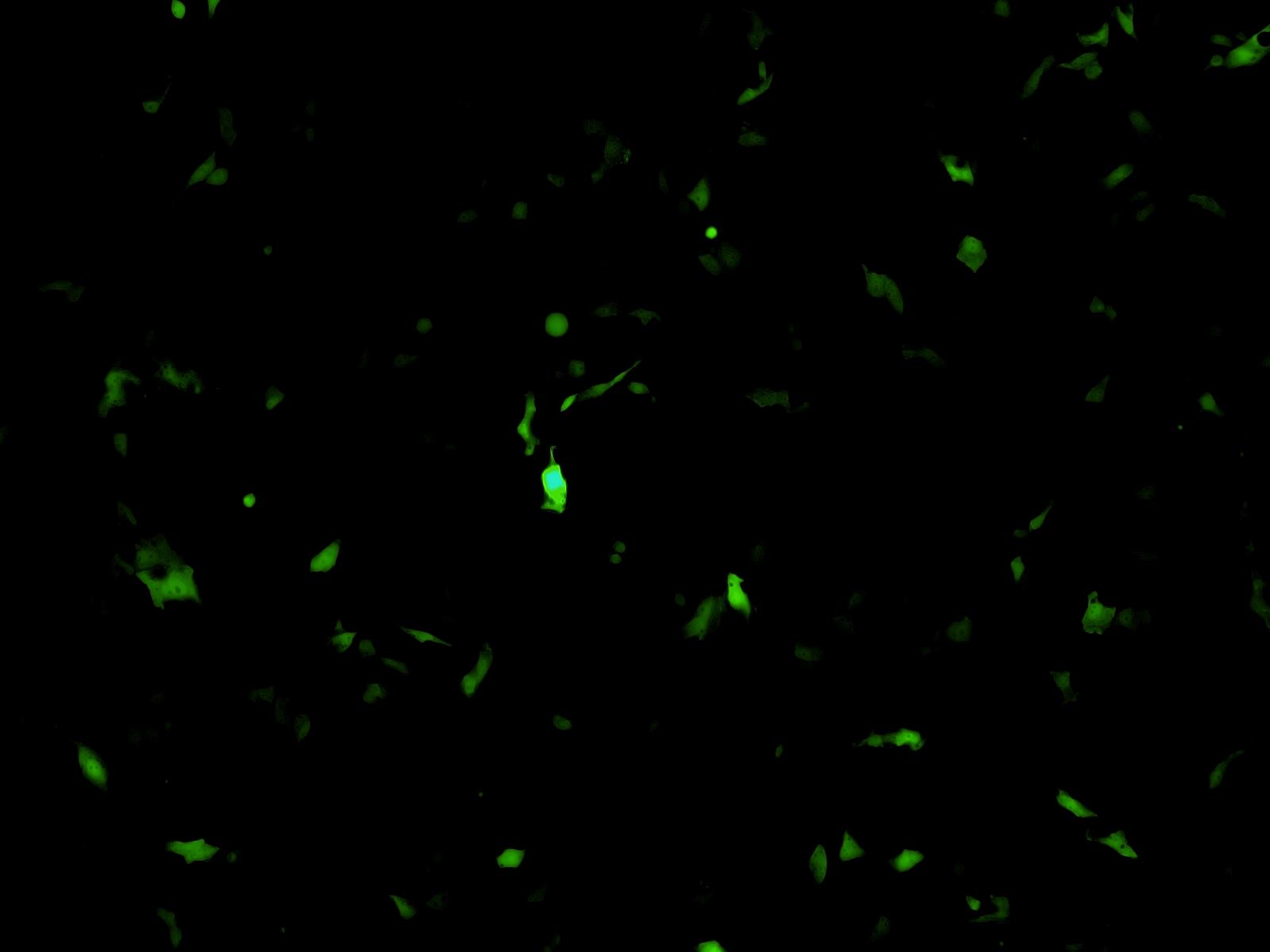

Supplement: Supplementary file 4 [file DataSheet_1.zip › Data Sheet 1/Fig2C/3-day1-Scrambled-AC009948.5.jpg]

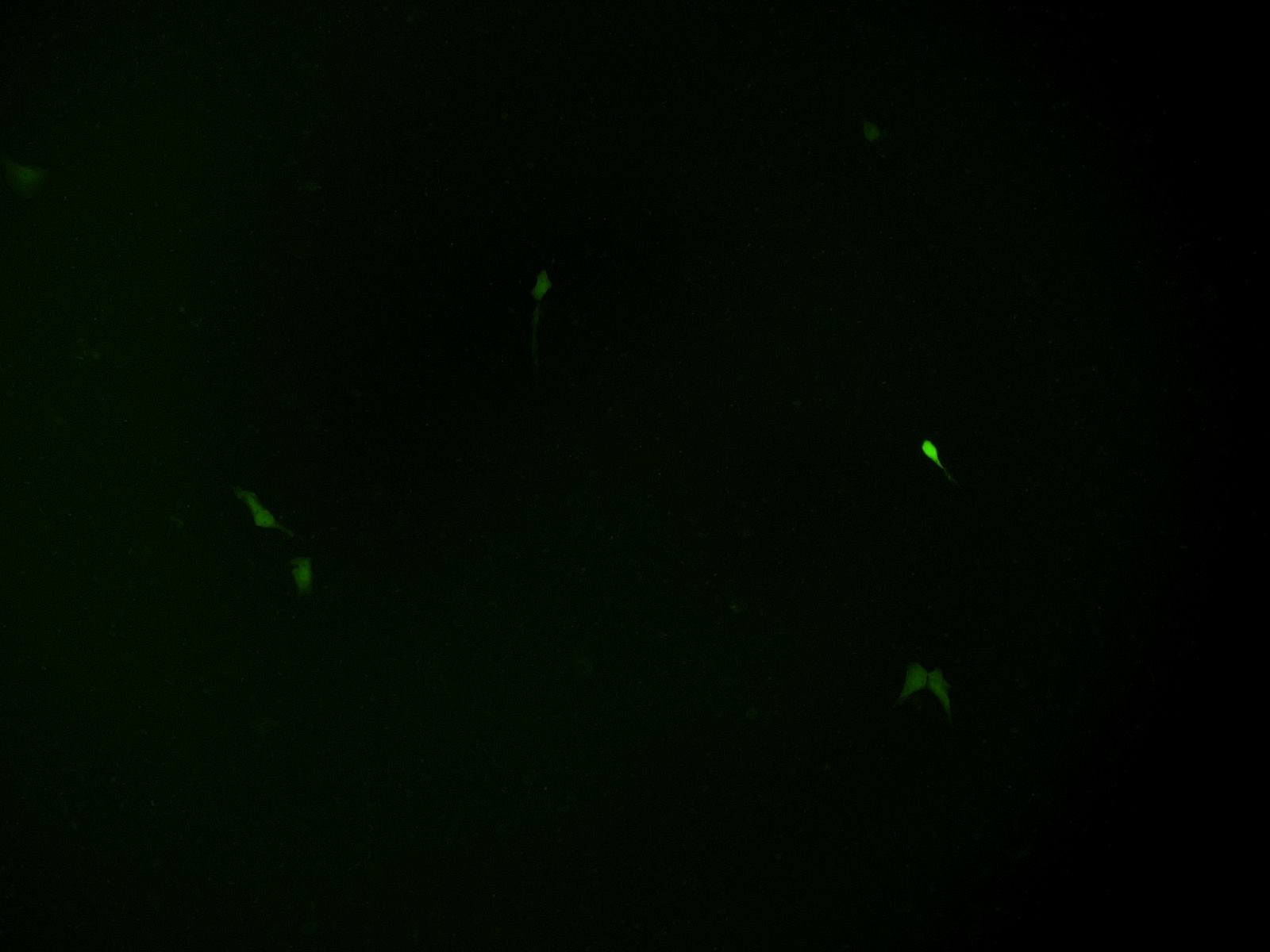

Supplement: Supplementary file 4 [file DataSheet_1.zip › Data Sheet 1/Fig2C/3-day1-siAC009948.5.jpg]

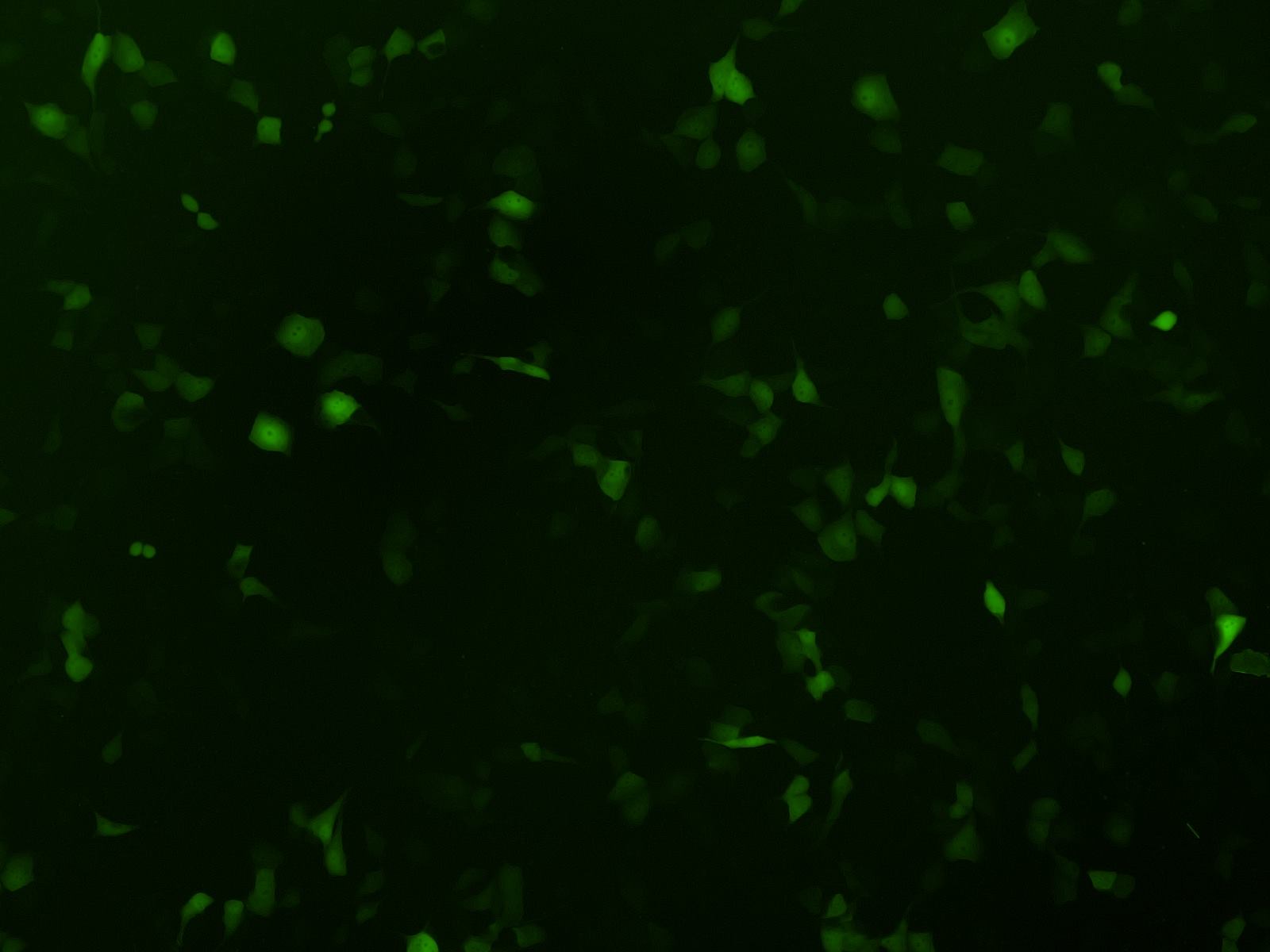

Supplement: Supplementary file 4 [file DataSheet_1.zip › Data Sheet 1/Fig2C/3-day2-NC-AC009948.5.jpg]

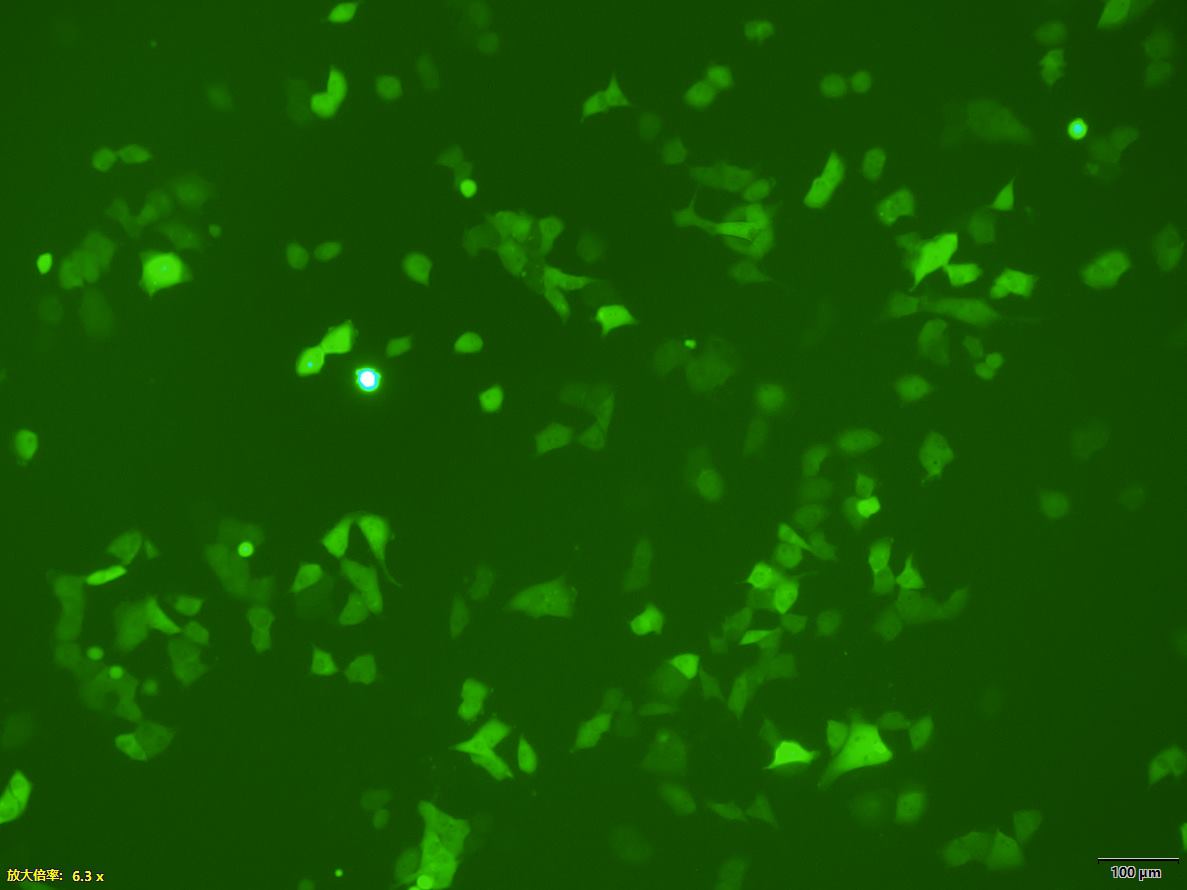

Supplement: Supplementary file 4 [file DataSheet_1.zip › Data Sheet 1/Fig2C/3-day2-overAC009948.5.jpg]

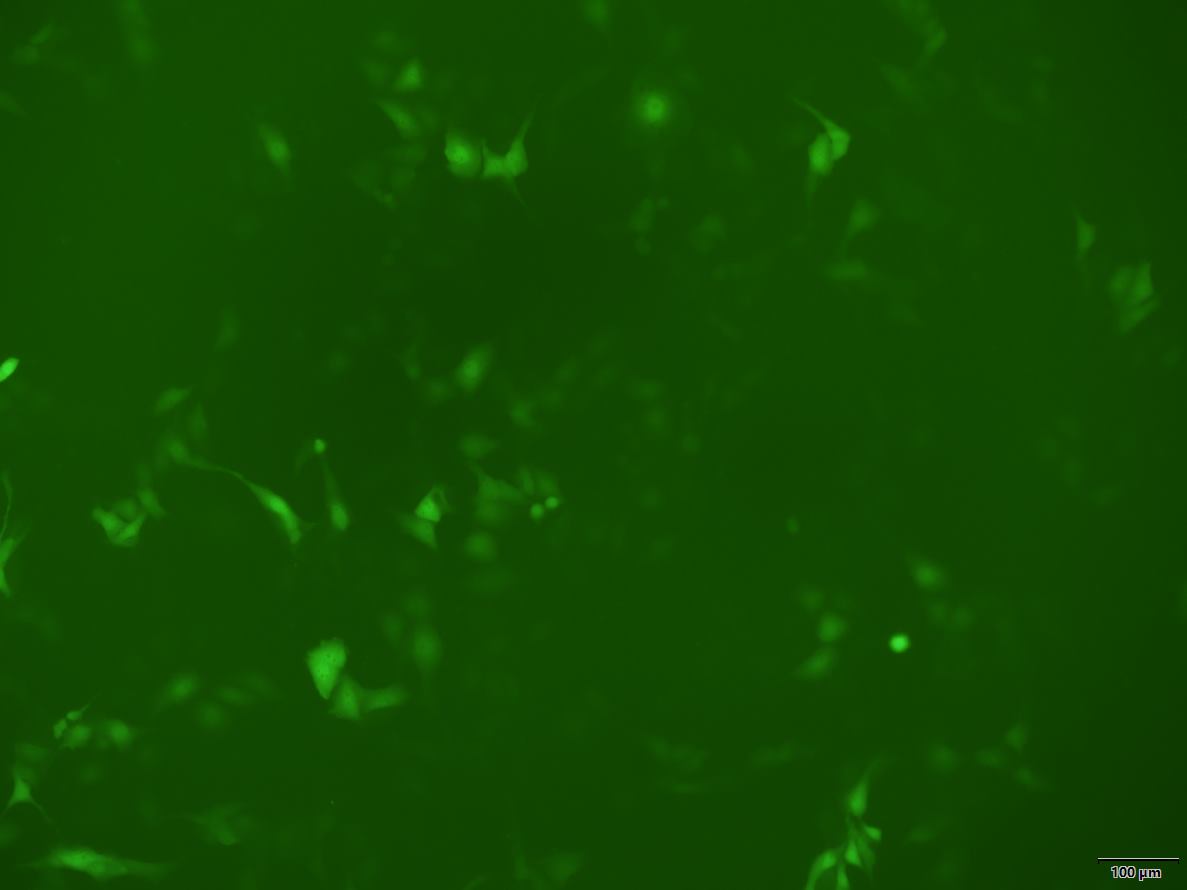

Supplement: Supplementary file 4 [file DataSheet_1.zip › Data Sheet 1/Fig2C/3-day2-Scrambled-AC009948.5.jpg]

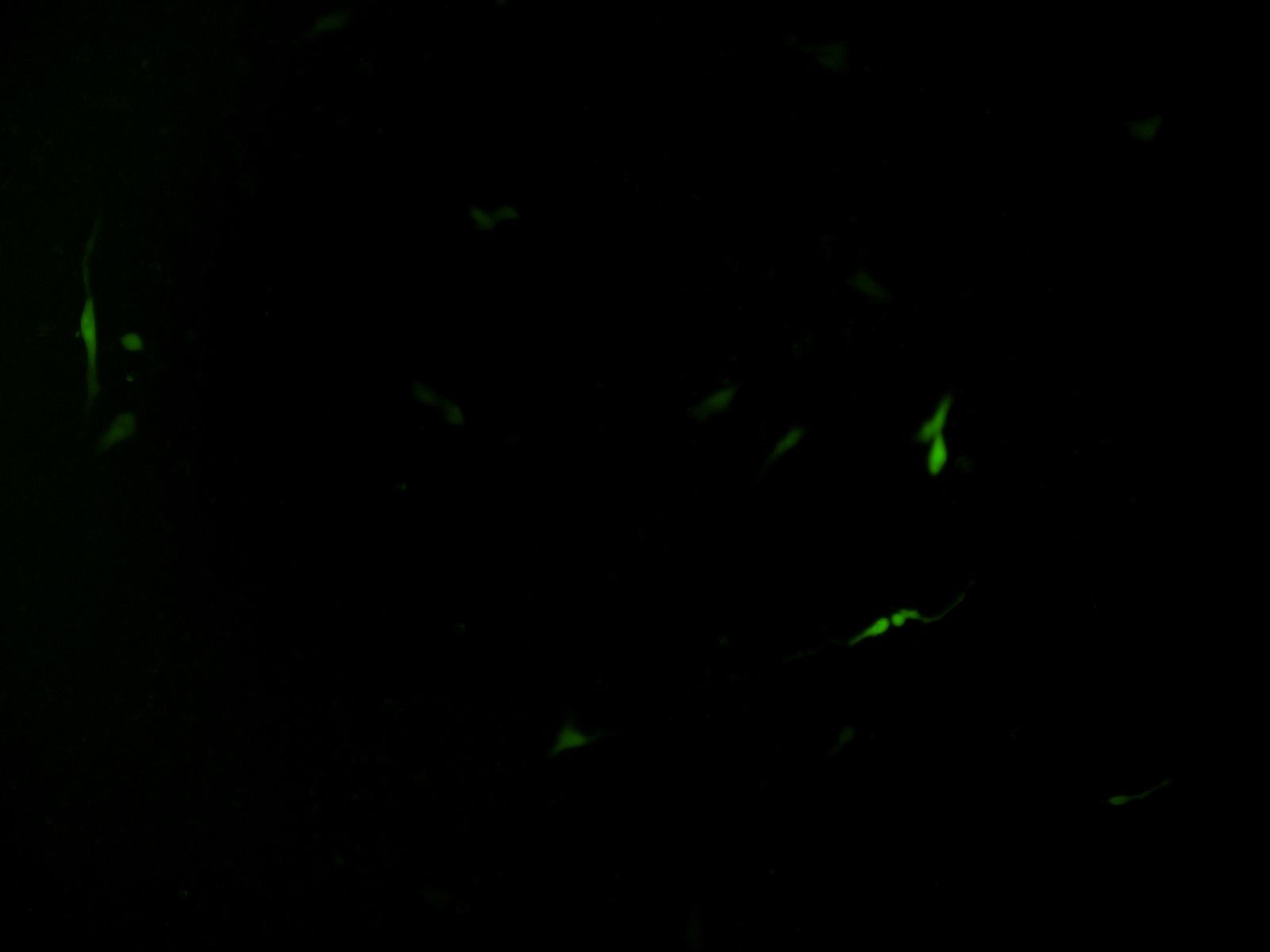

Supplement: Supplementary file 4 [file DataSheet_1.zip › Data Sheet 1/Fig2C/3-day2-siAC009948.5.jpg]

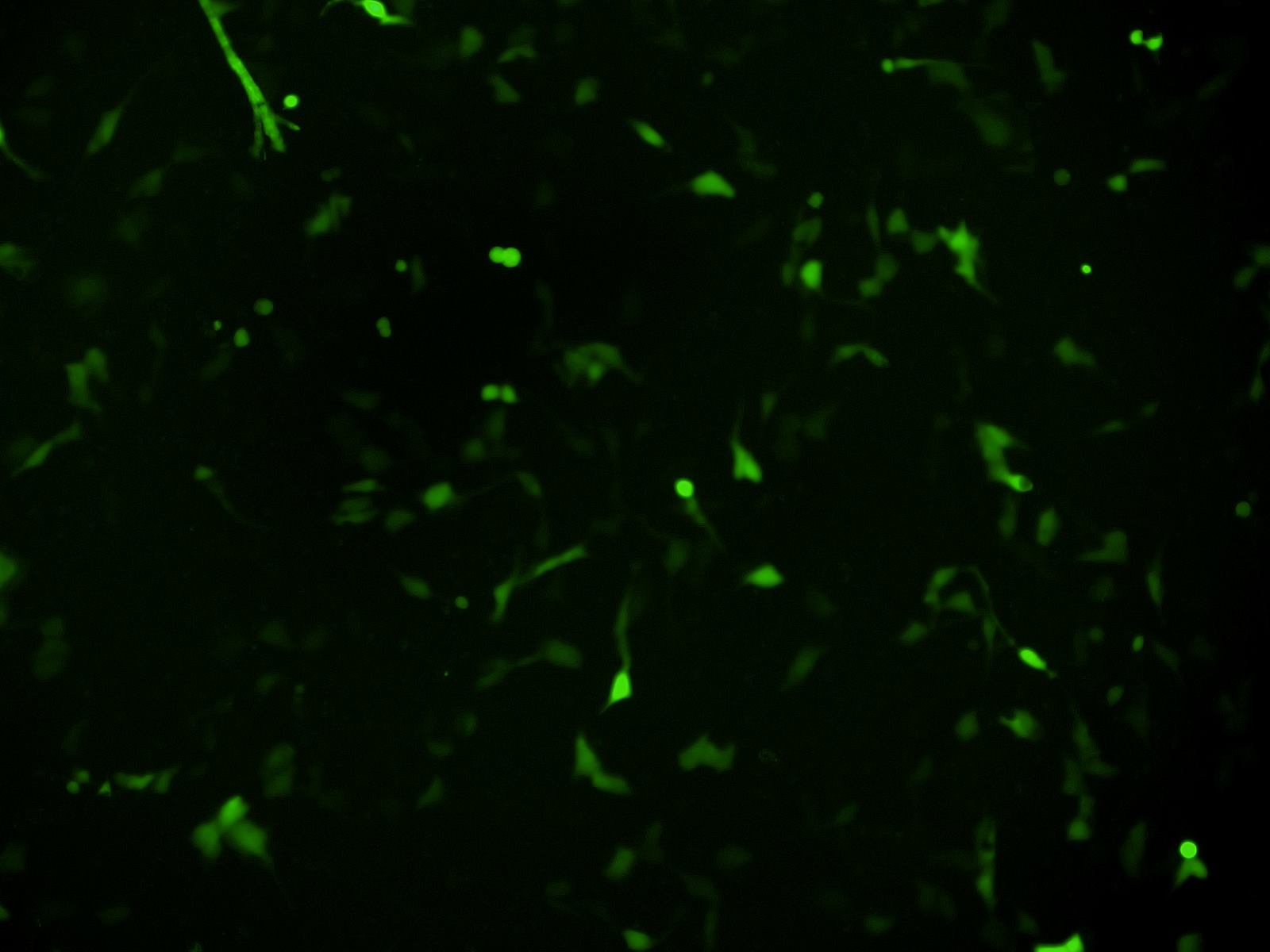

Supplement: Supplementary file 4 [file DataSheet_1.zip › Data Sheet 1/Fig2C/3-day3-NC-AC009948.5.jpg]

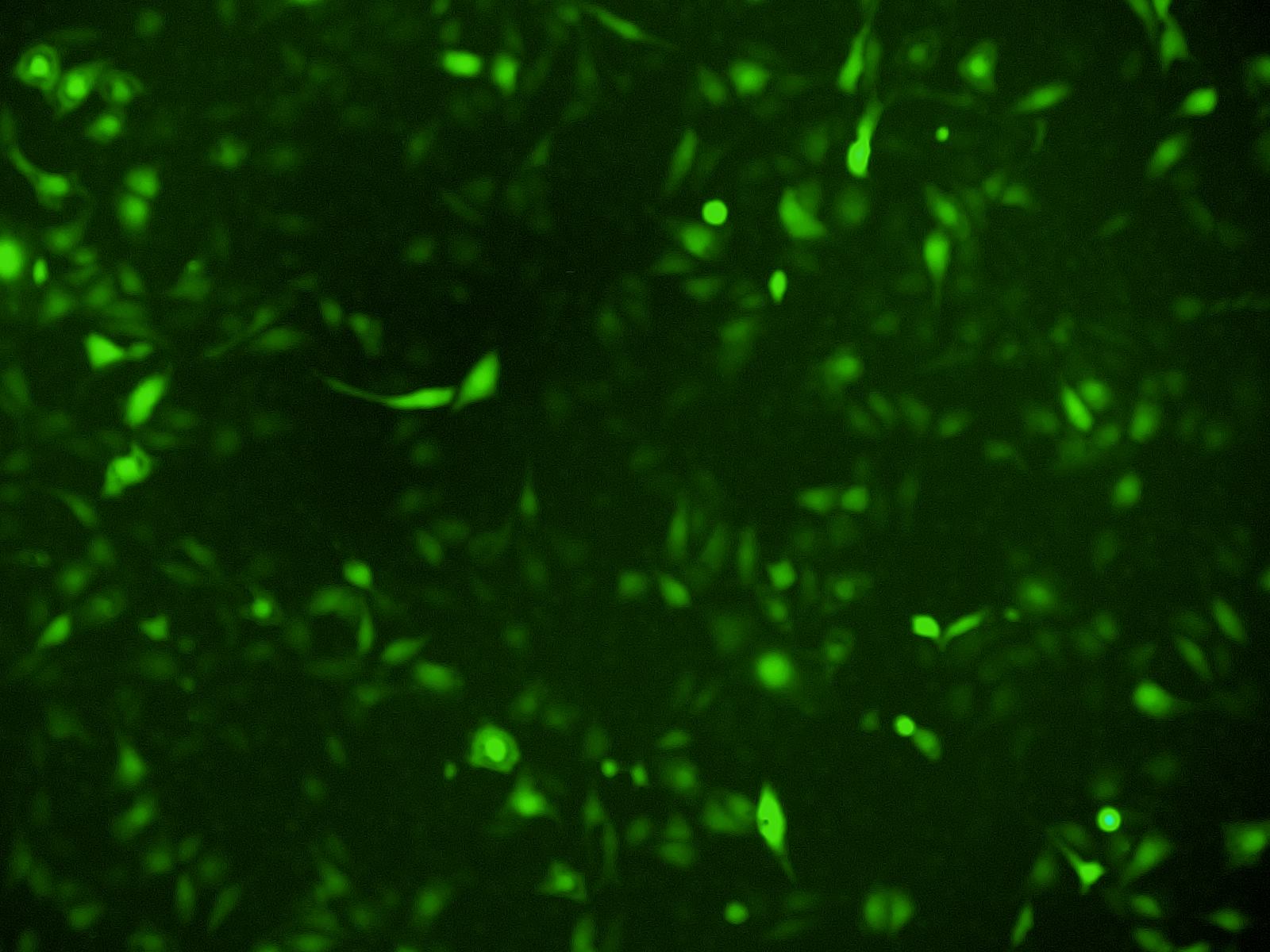

Supplement: Supplementary file 4 [file DataSheet_1.zip › Data Sheet 1/Fig2C/3-day3-overAC009948.5.jpg]

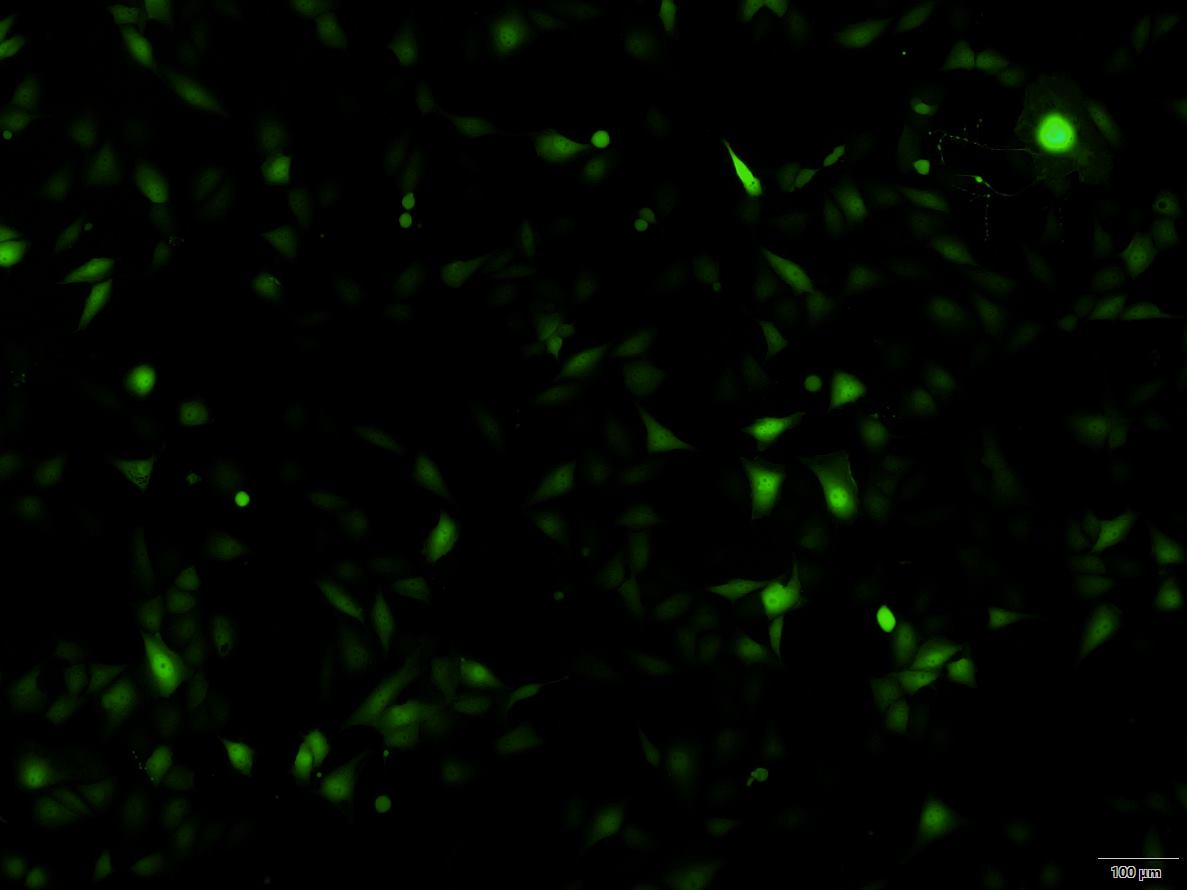

Supplement: Supplementary file 4 [file DataSheet_1.zip › Data Sheet 1/Fig2C/3-day3-Scrambled-AC009948.5.jpg]

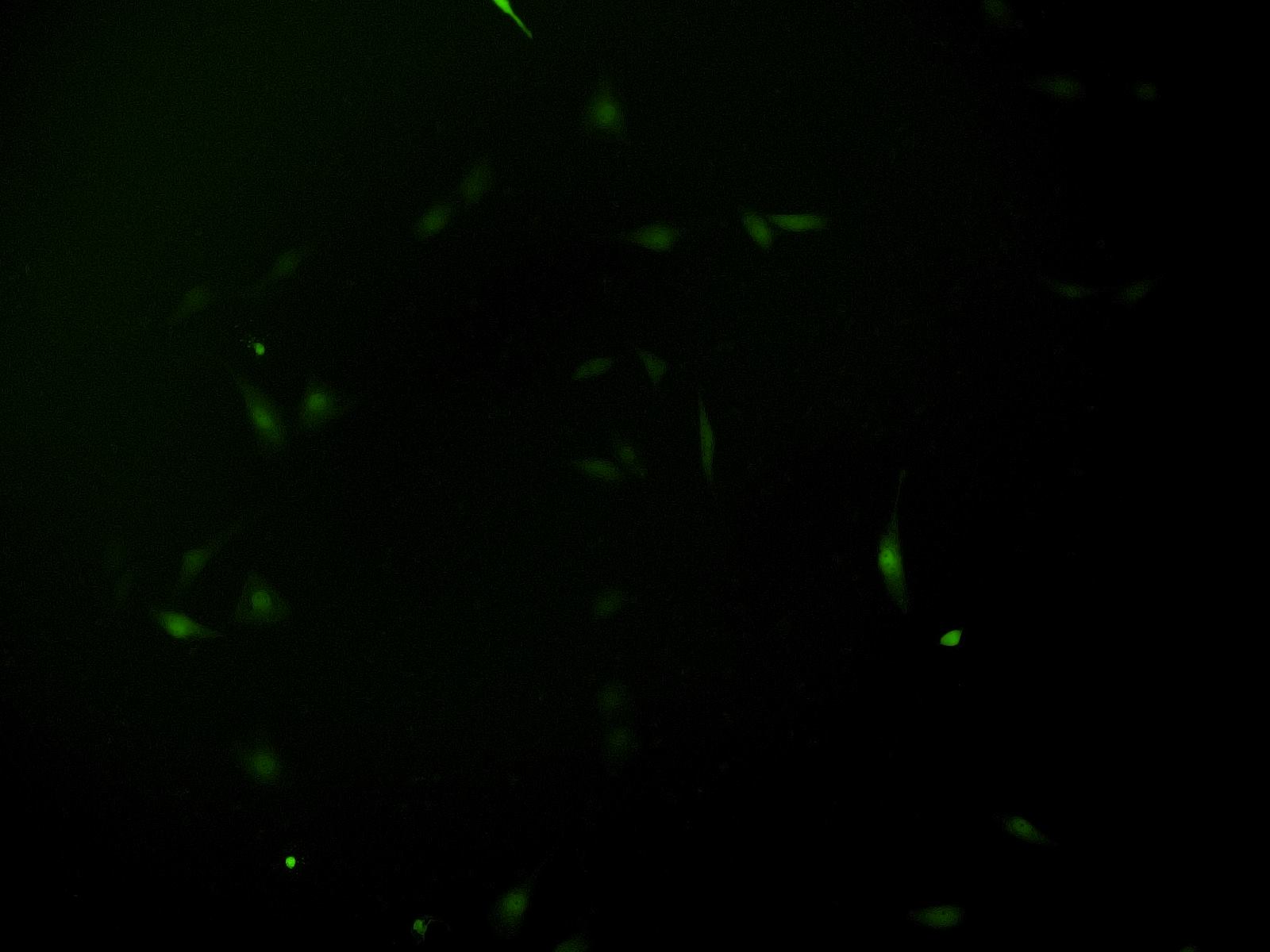

Supplement: Supplementary file 4 [file DataSheet_1.zip › Data Sheet 1/Fig2C/3-day3-siAC009948.5.jpg]

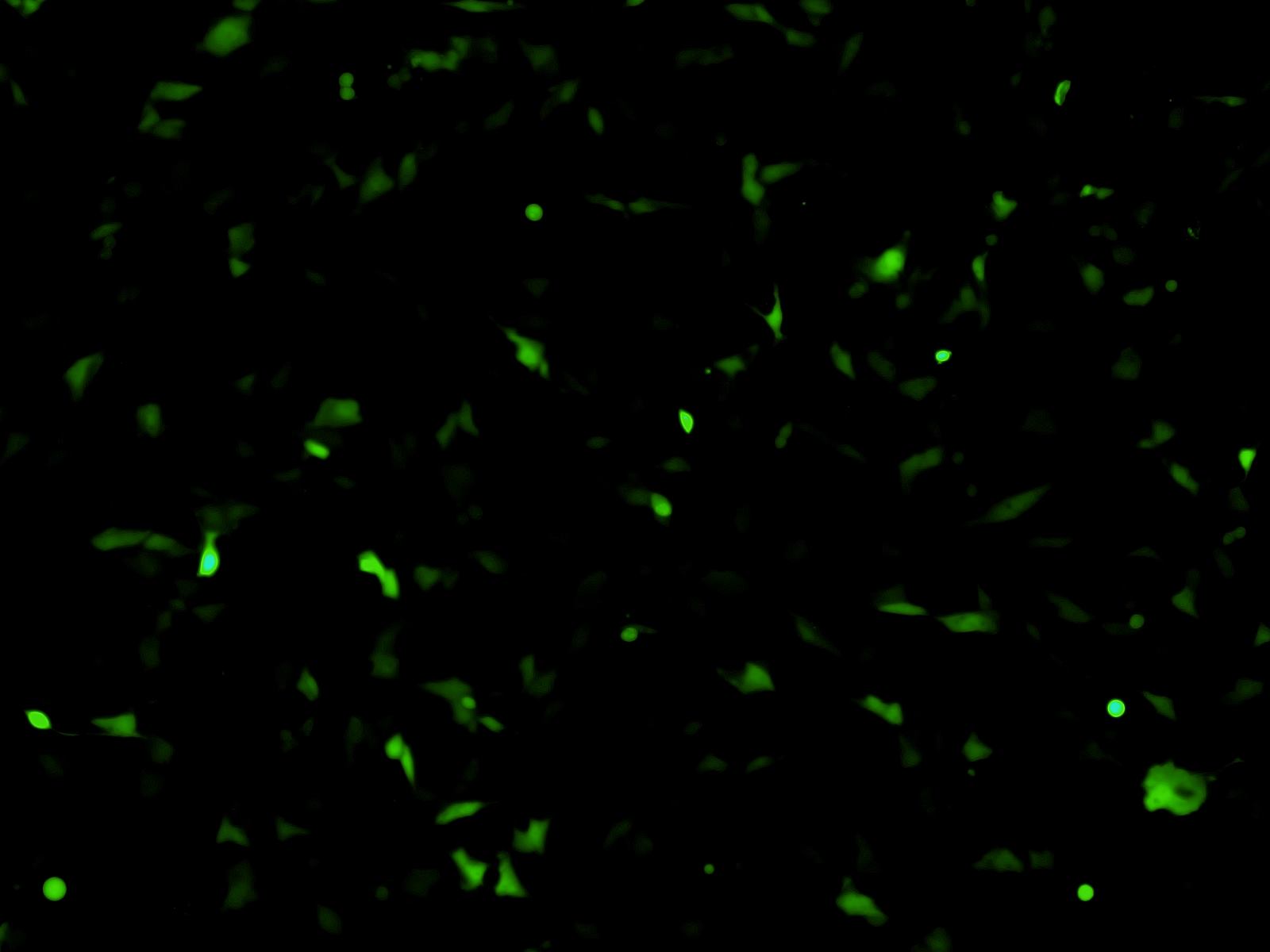

Supplement: Supplementary file 4 [file DataSheet_1.zip › Data Sheet 1/Fig2C/3-day4-NC-AC009948.5.jpg]

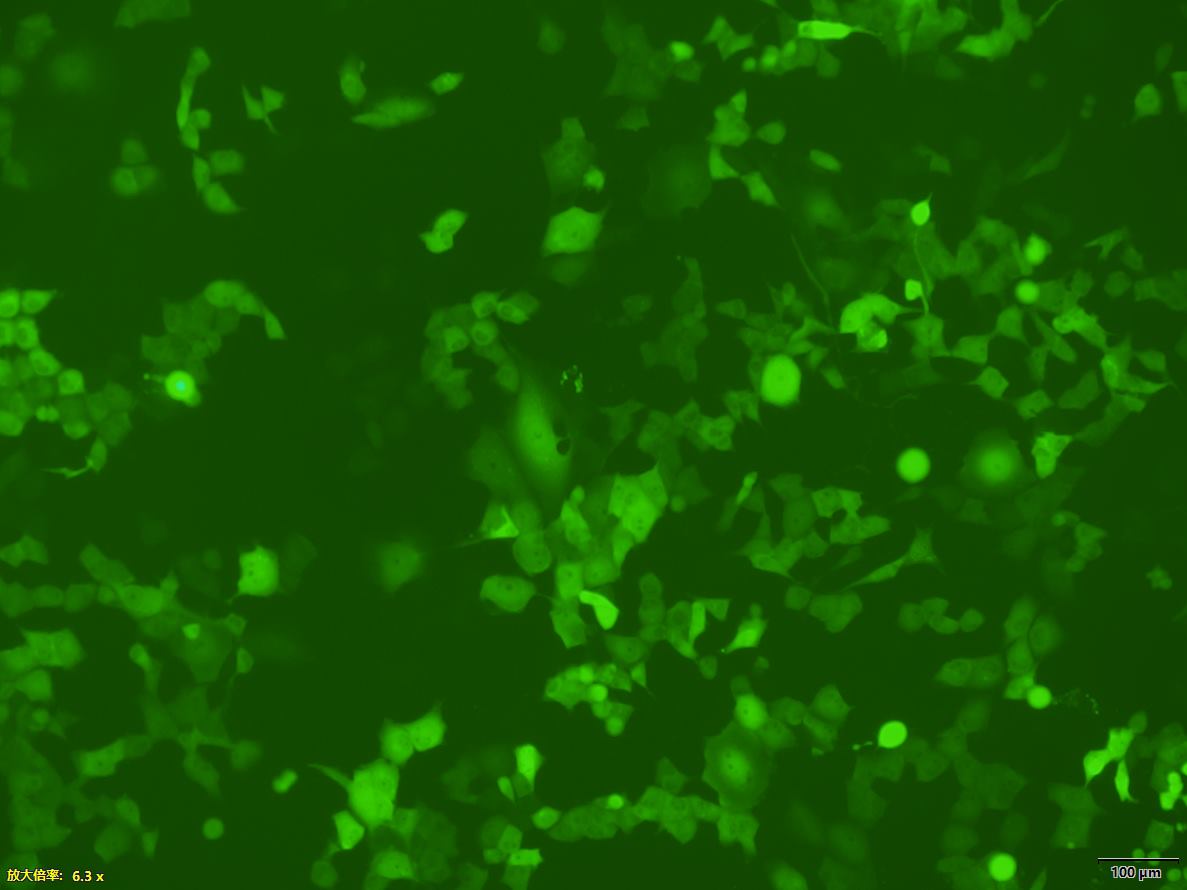

Supplement: Supplementary file 4 [file DataSheet_1.zip › Data Sheet 1/Fig2C/3-day4-overAC009948.5.jpg]

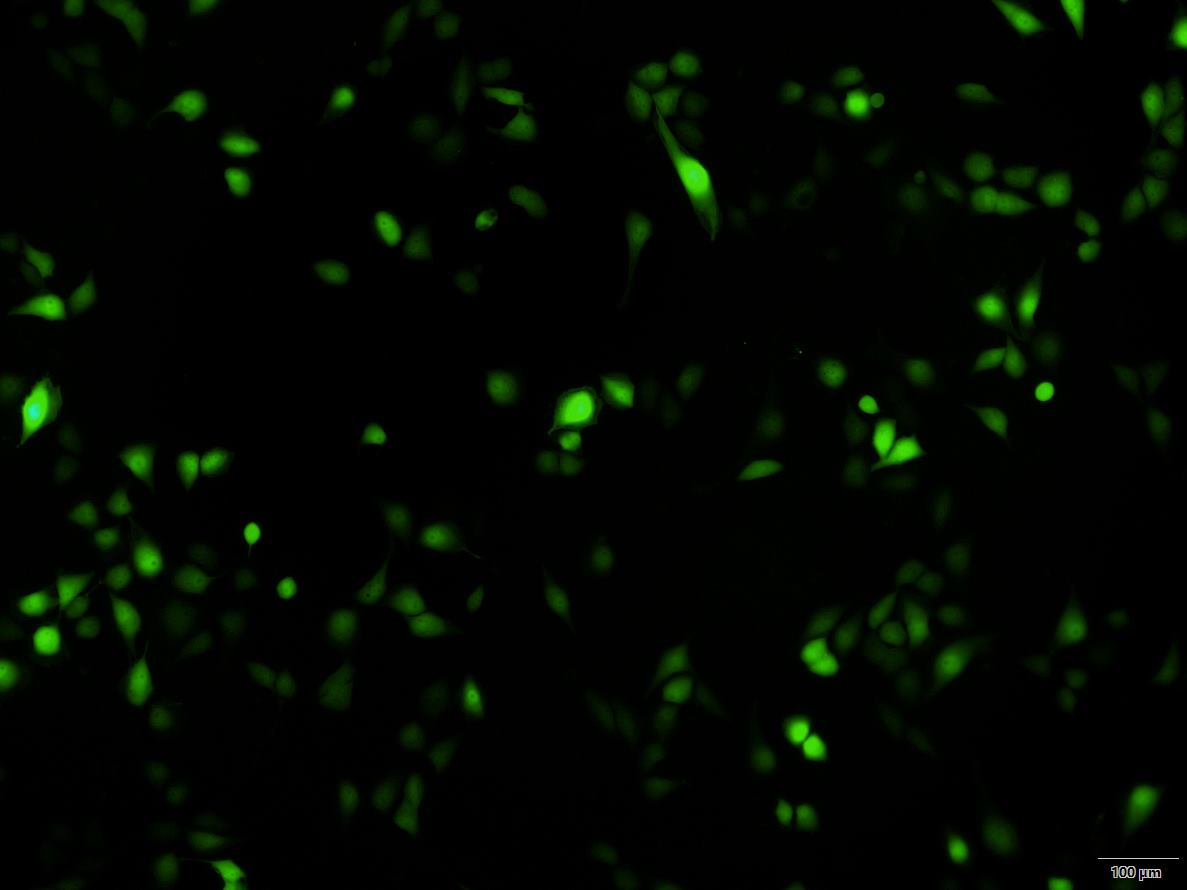

Supplement: Supplementary file 4 [file DataSheet_1.zip › Data Sheet 1/Fig2C/3-day4-Scrambled-AC009948.5.jpg]

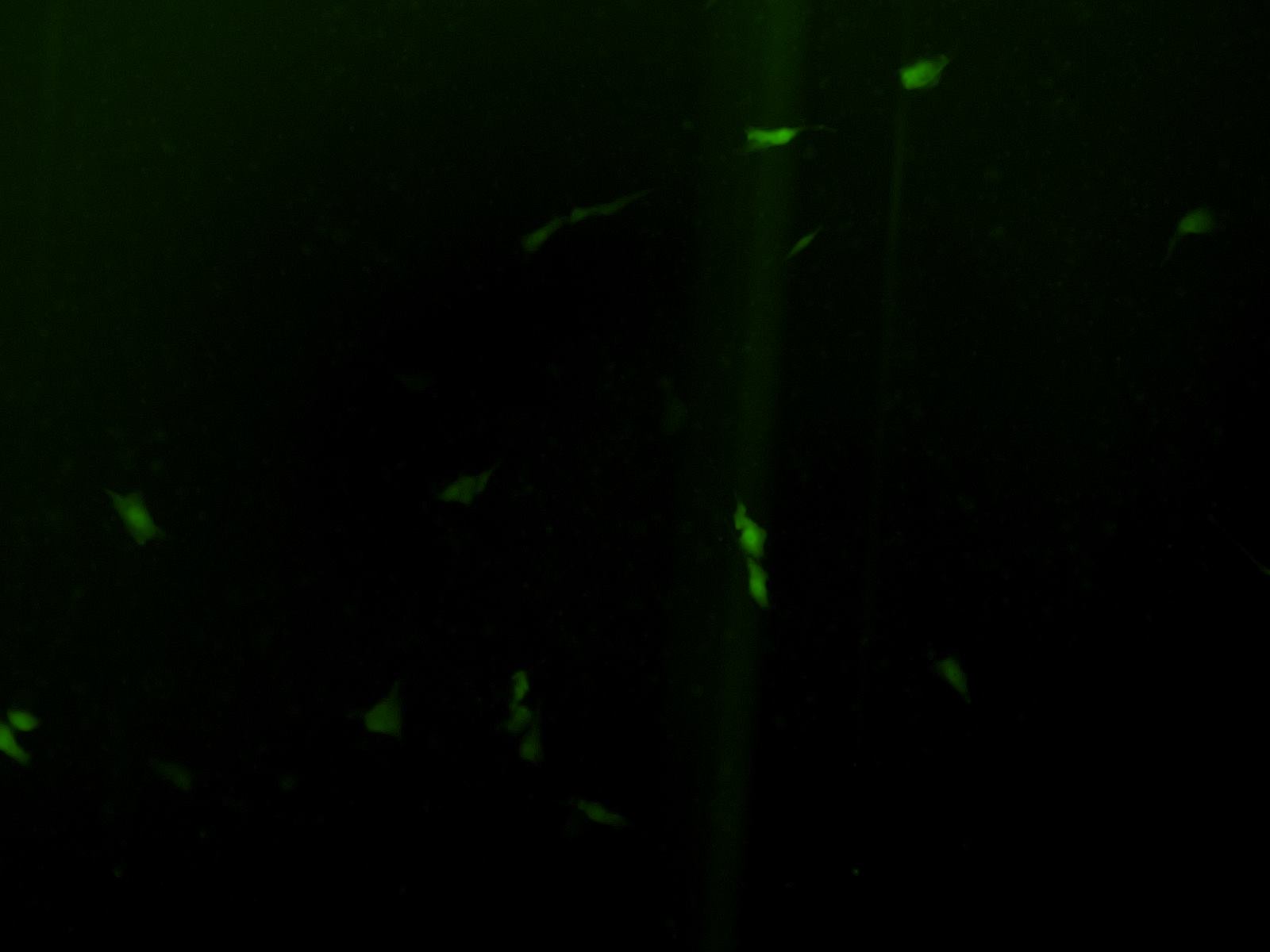

Supplement: Supplementary file 4 [file DataSheet_1.zip › Data Sheet 1/Fig2C/3-day4-siAC009948.5.jpg]

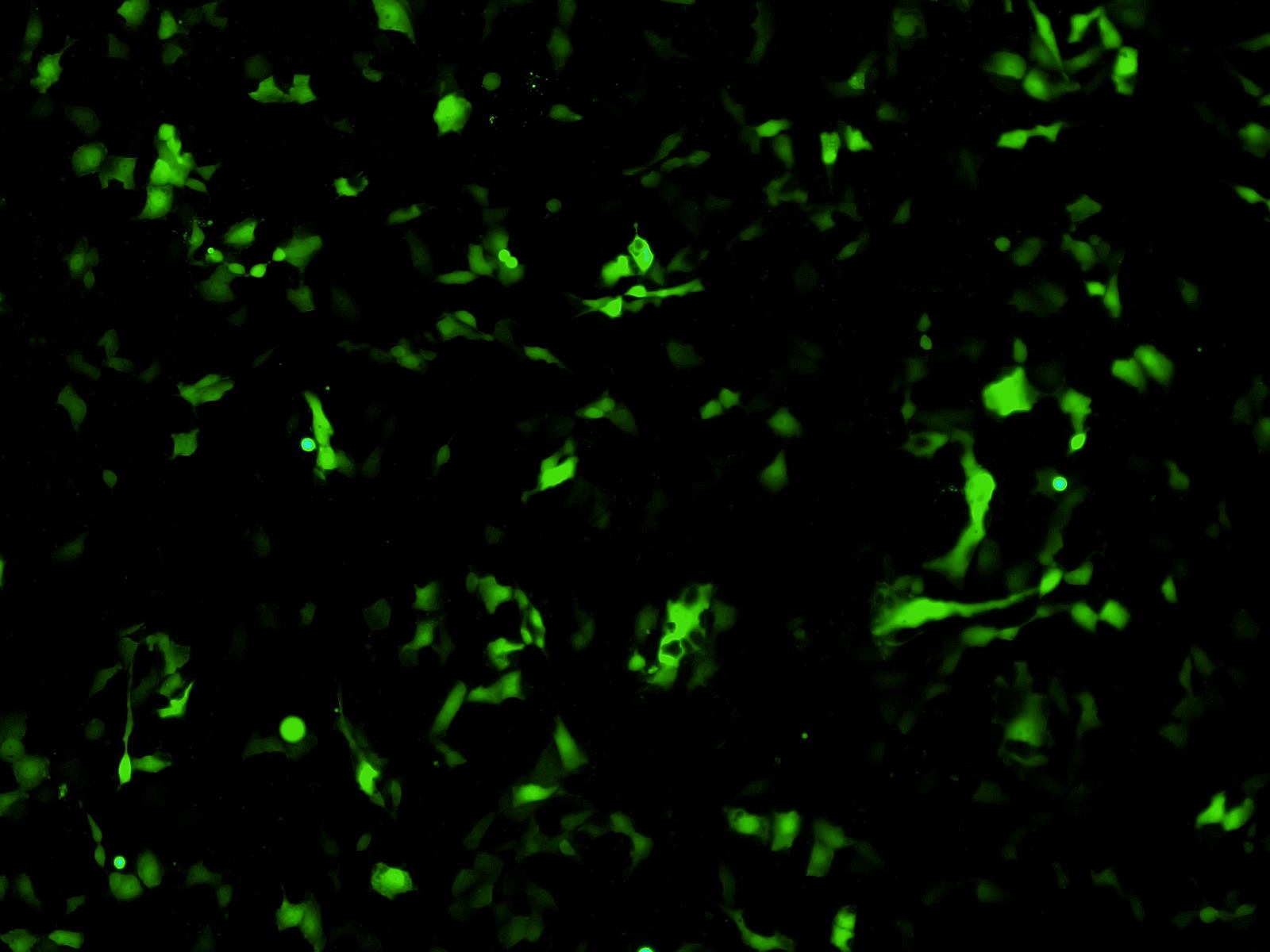

Supplement: Supplementary file 4 [file DataSheet_1.zip › Data Sheet 1/Fig2C/3-day5-NC-AC009948.5.jpg]

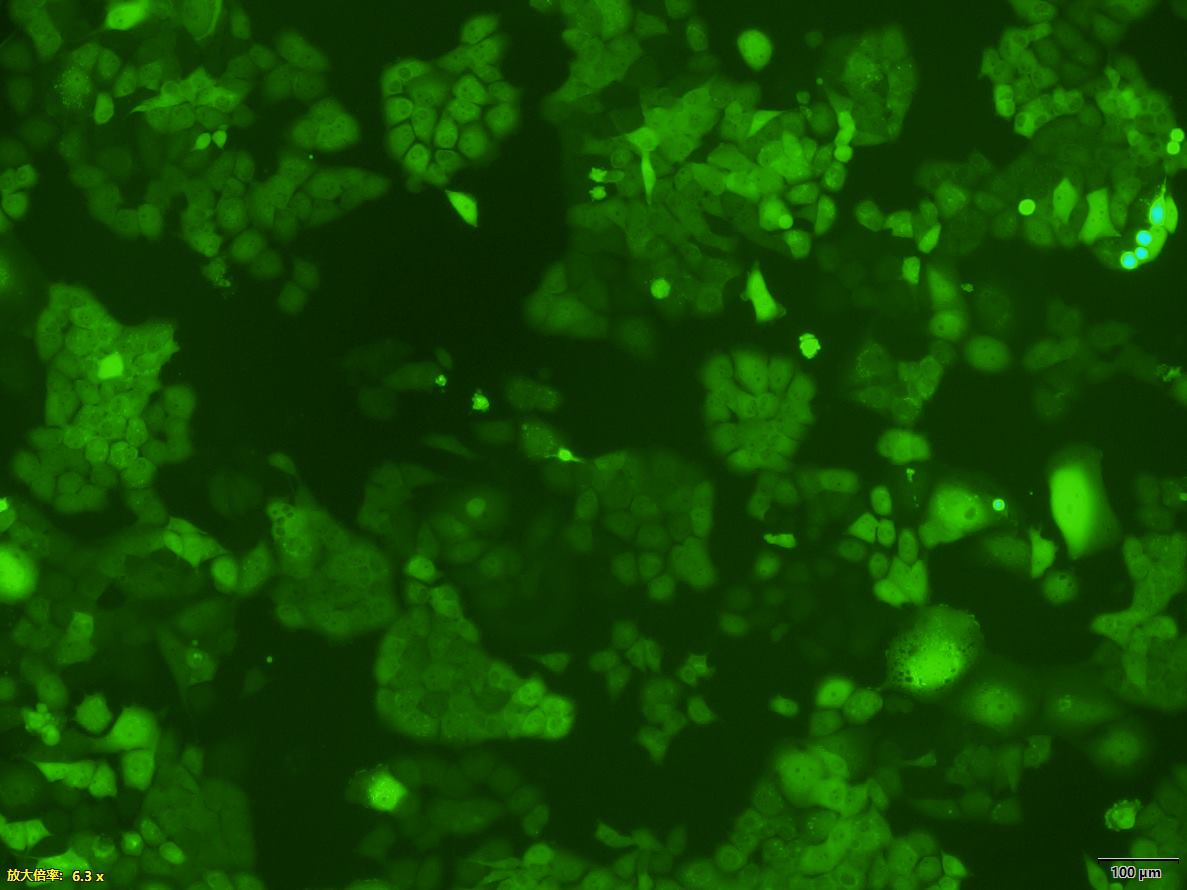

Supplement: Supplementary file 4 [file DataSheet_1.zip › Data Sheet 1/Fig2C/3-day5-overAC009948.5.jpg]

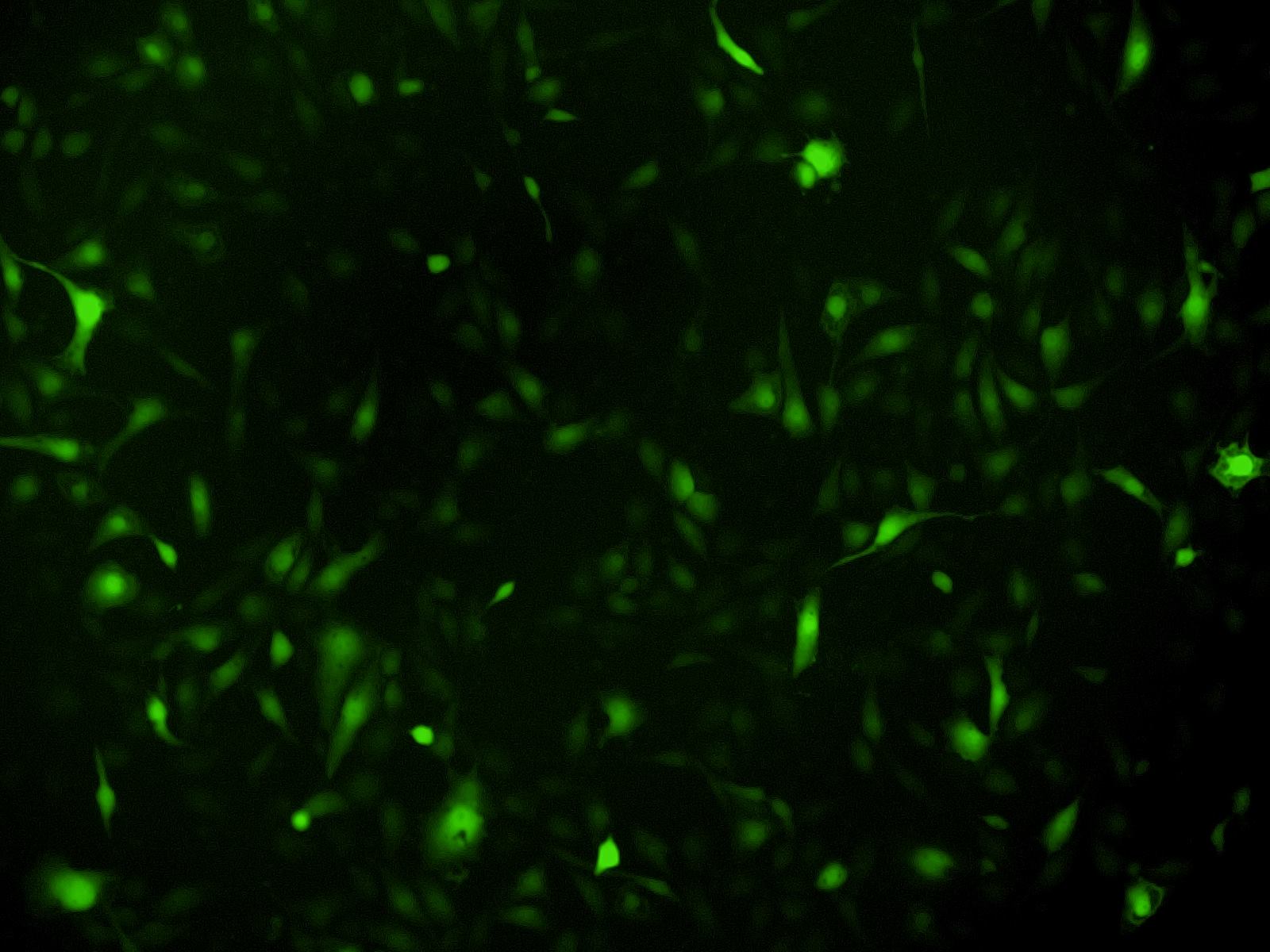

Supplement: Supplementary file 4 [file DataSheet_1.zip › Data Sheet 1/Fig2C/3-day5-Scrambled-AC009948.5.jpg]

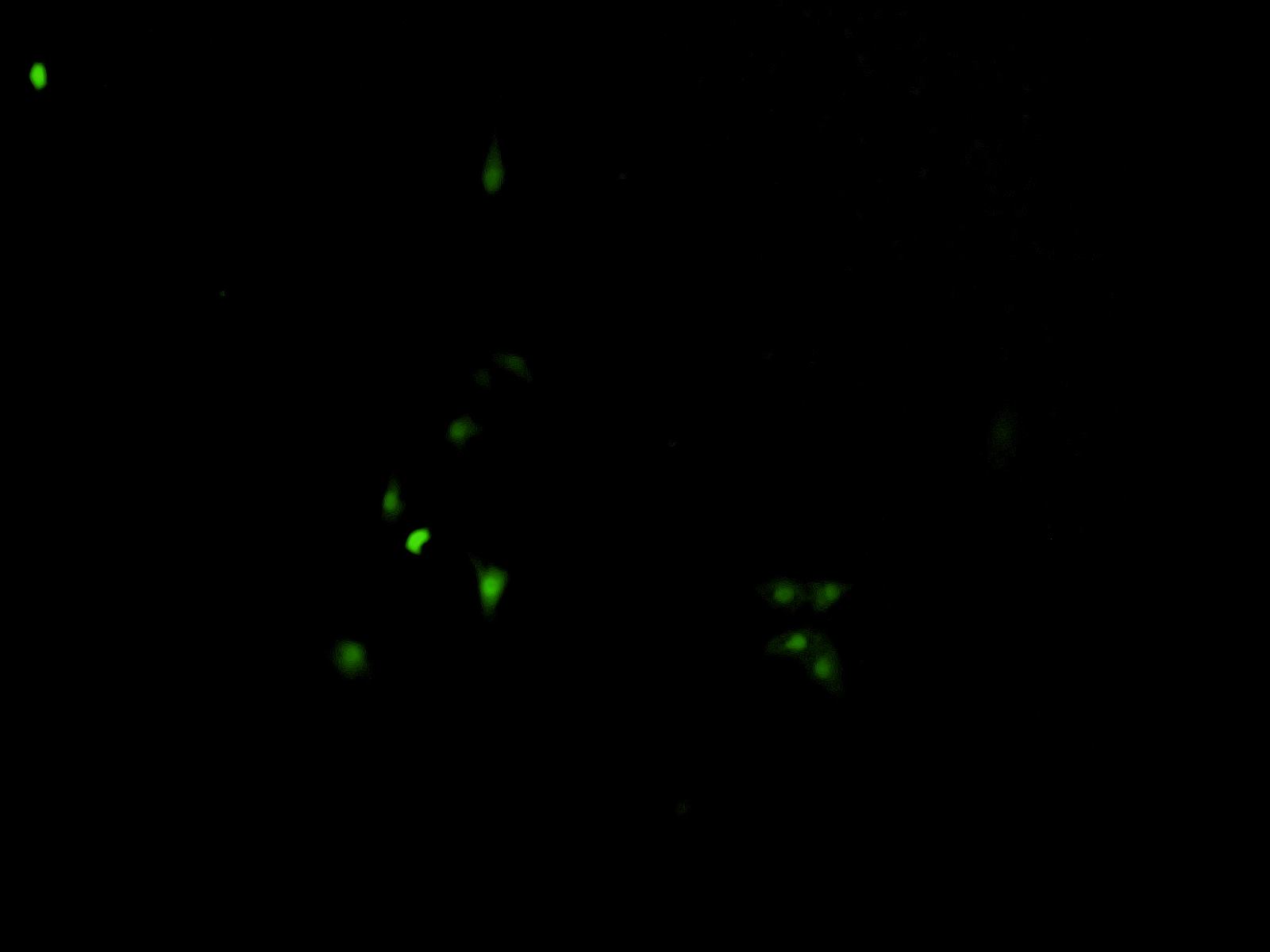

Supplement: Supplementary file 4 [file DataSheet_1.zip › Data Sheet 1/Fig2C/3-day5-siAC009948.5.jpg]

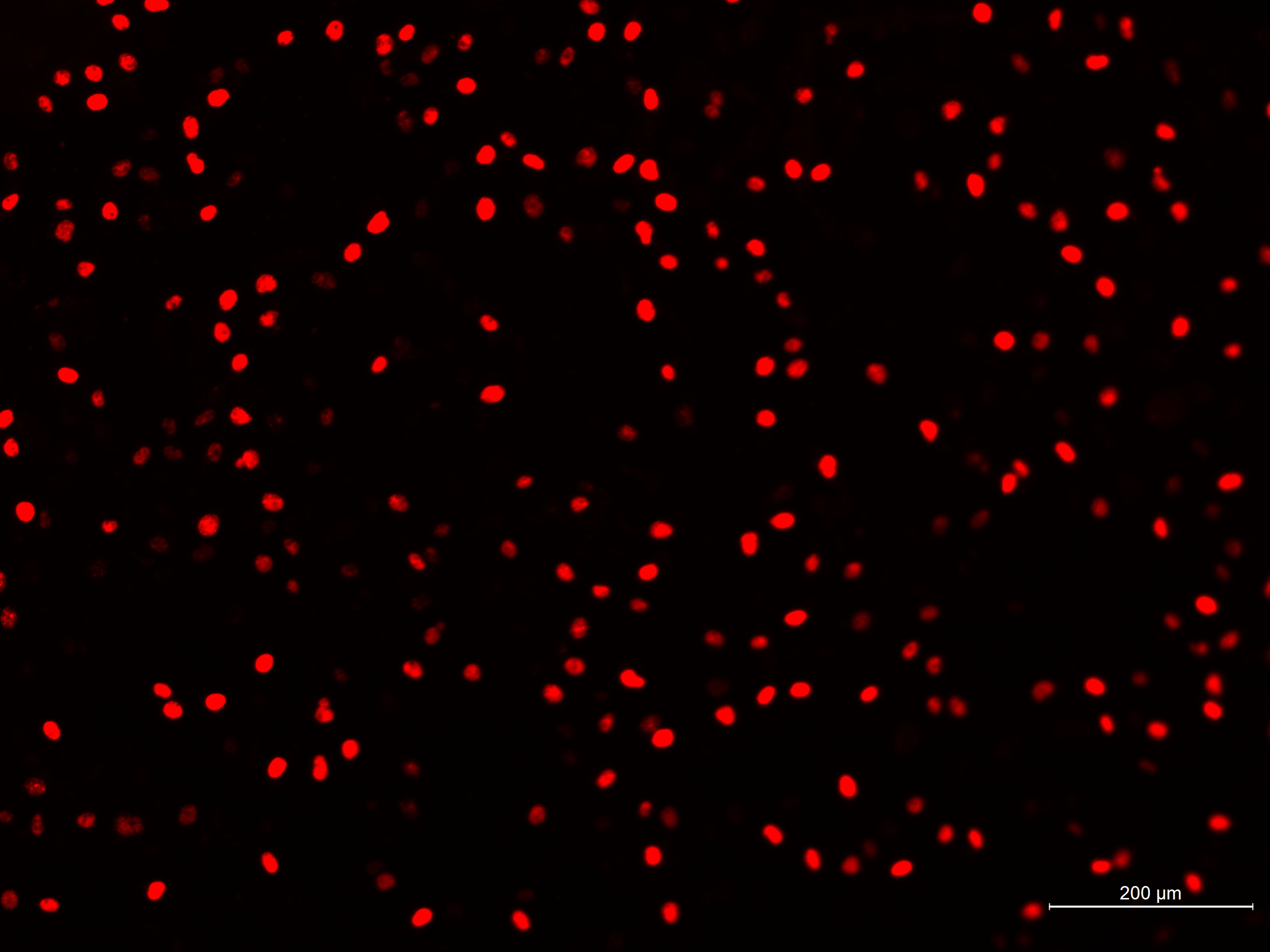

Supplement: Supplementary file 4 [file DataSheet_1.zip › Data Sheet 1/Fig2D/1-1-NC-AC009948.5.jpg]

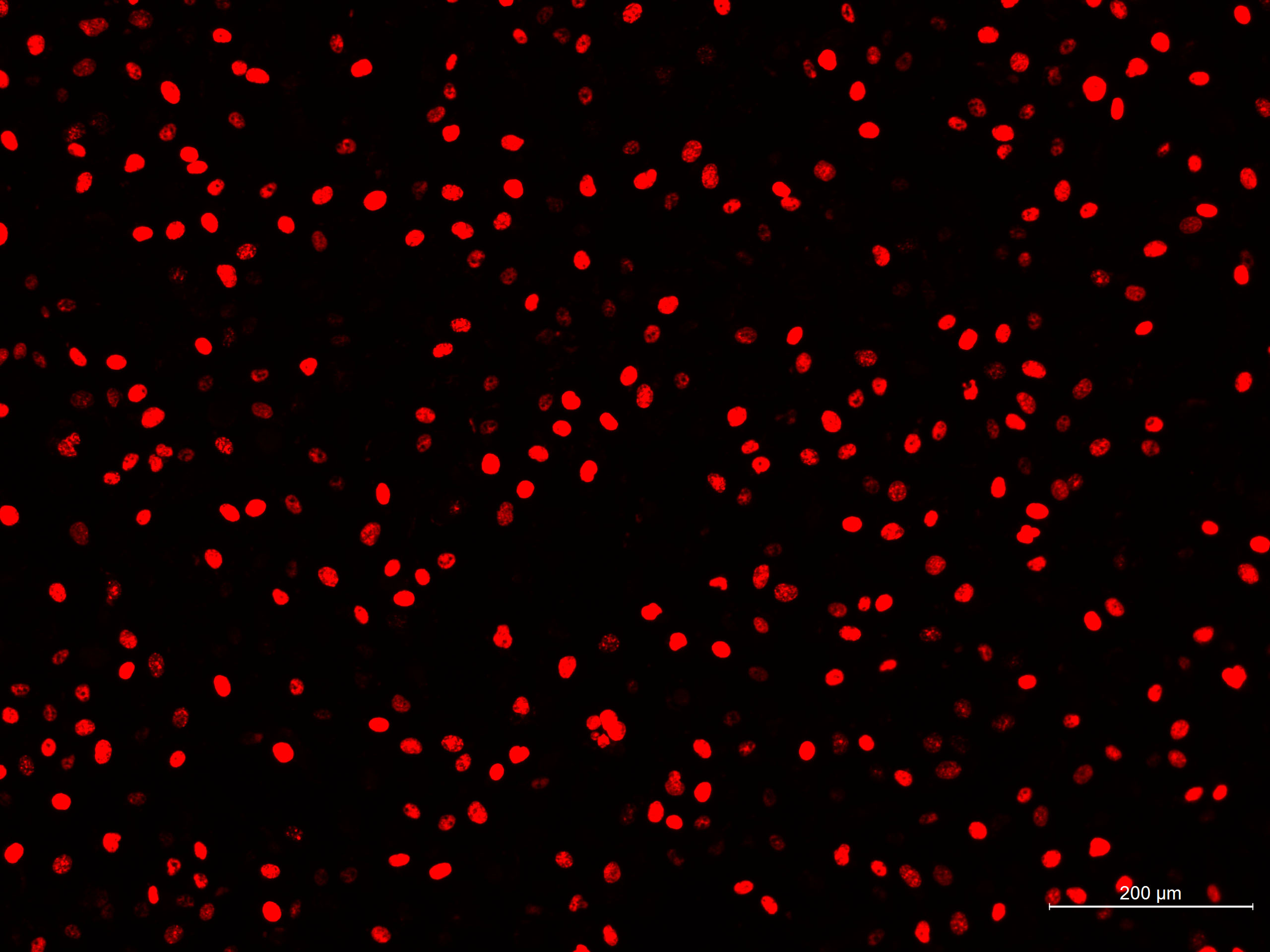

Supplement: Supplementary file 4 [file DataSheet_1.zip › Data Sheet 1/Fig2D/1-1-overAC009948.5.jpg]

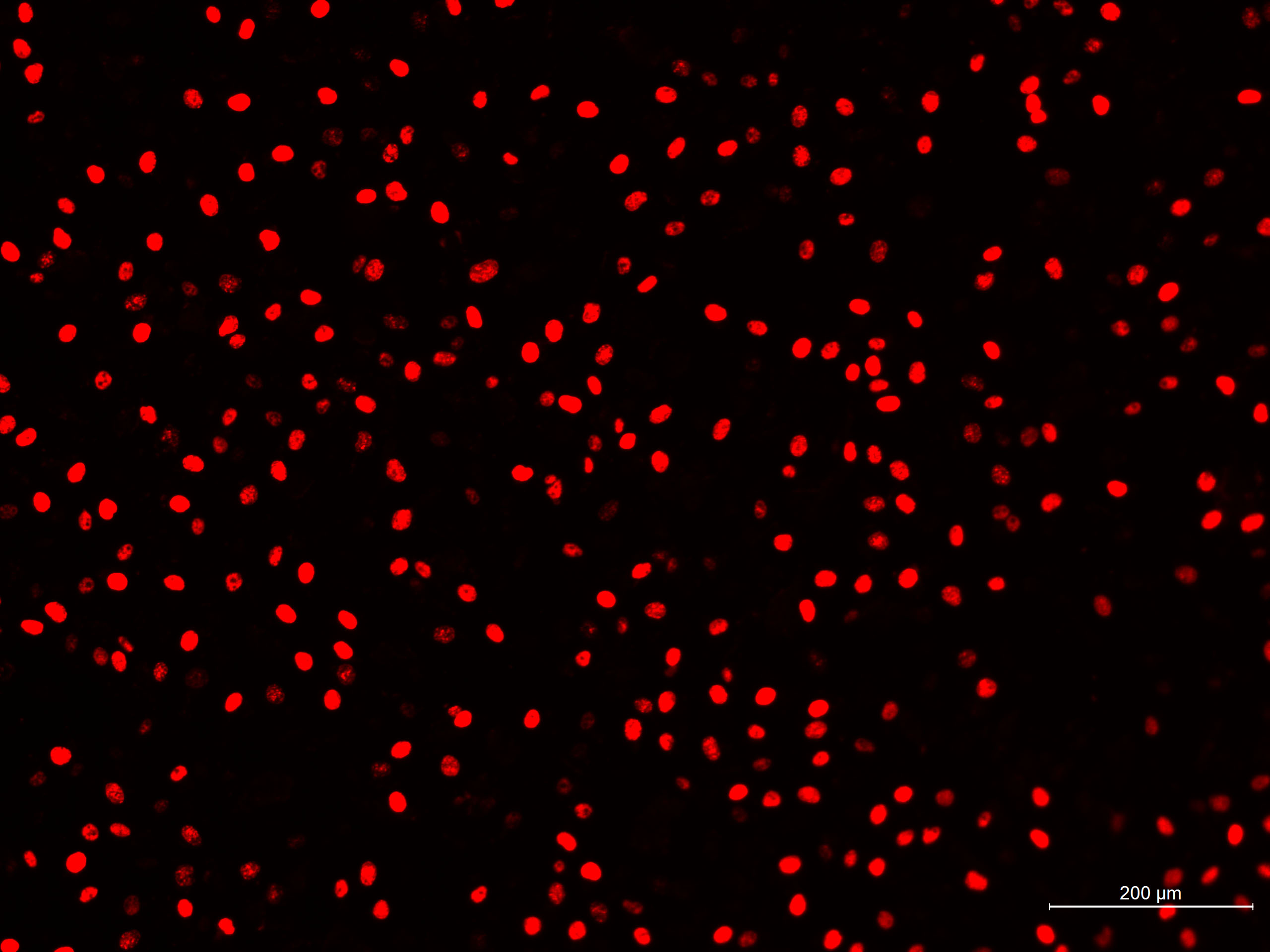

Supplement: Supplementary file 4 [file DataSheet_1.zip › Data Sheet 1/Fig2D/1-1-Scrambled-AC009948.5.jpg]

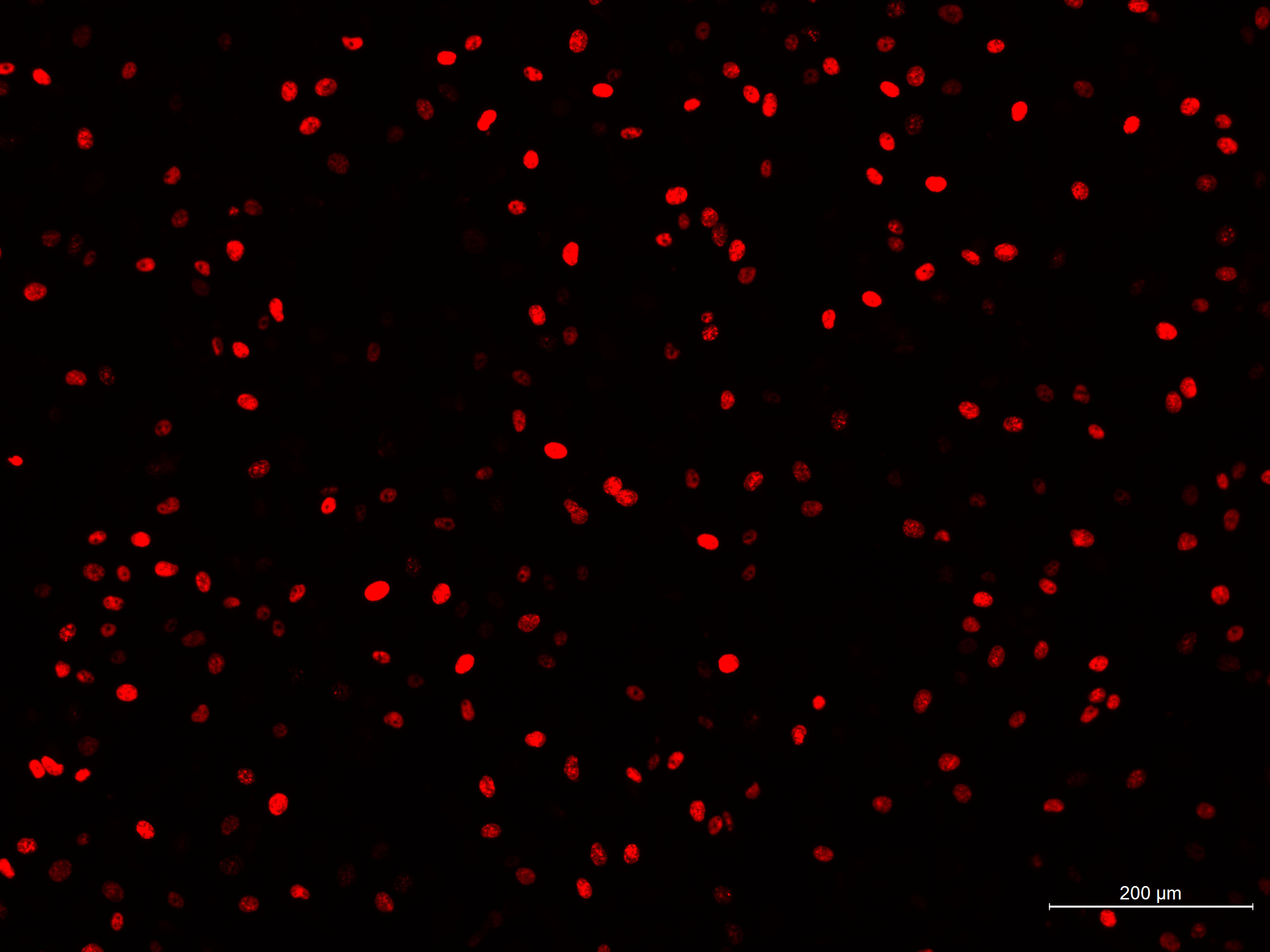

Supplement: Supplementary file 4 [file DataSheet_1.zip › Data Sheet 1/Fig2D/1-1-SiAC009948.5.jpg]

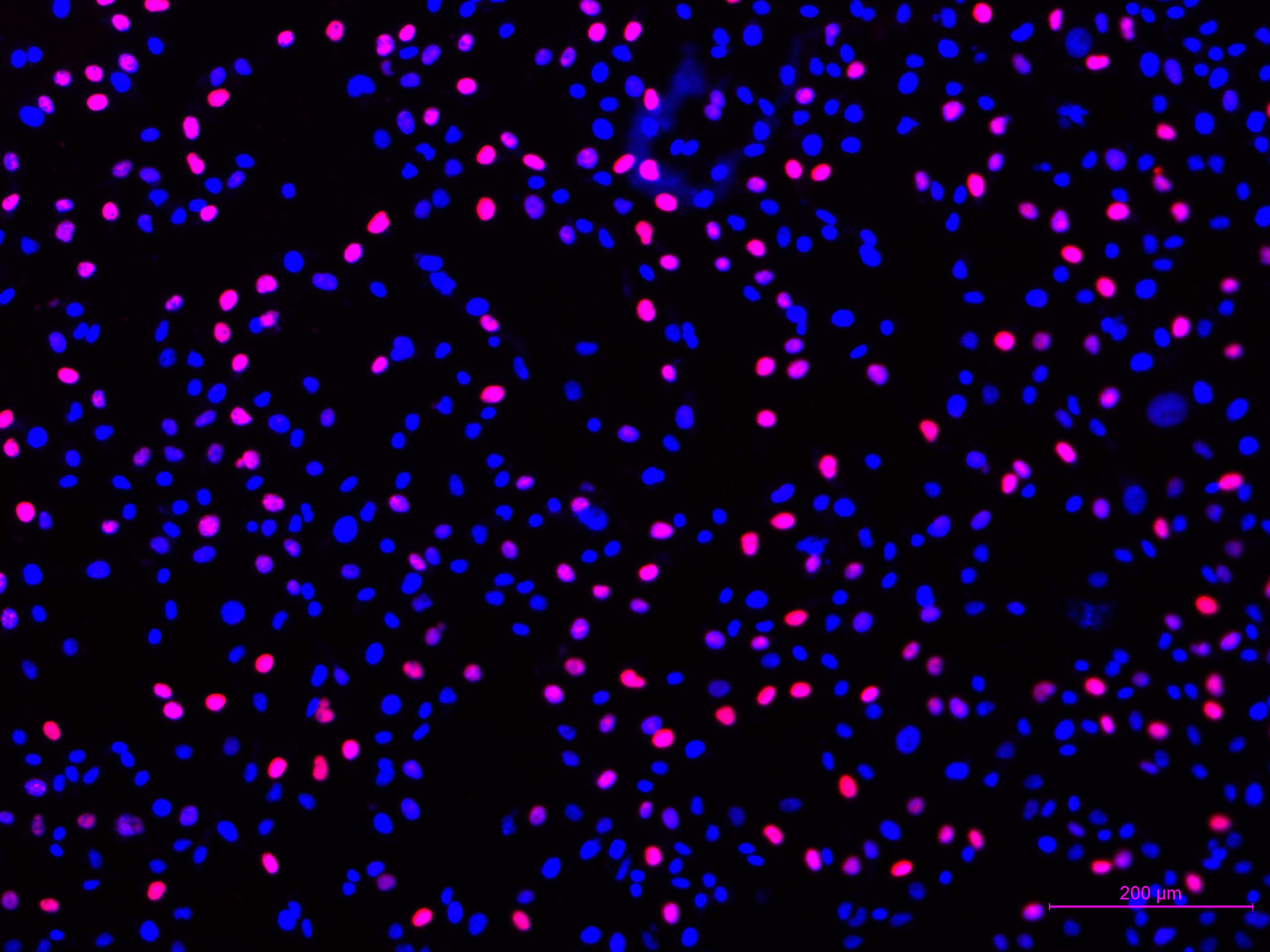

Supplement: Supplementary file 4 [file DataSheet_1.zip › Data Sheet 1/Fig2D/1-NC-AC009948.5.jpg]

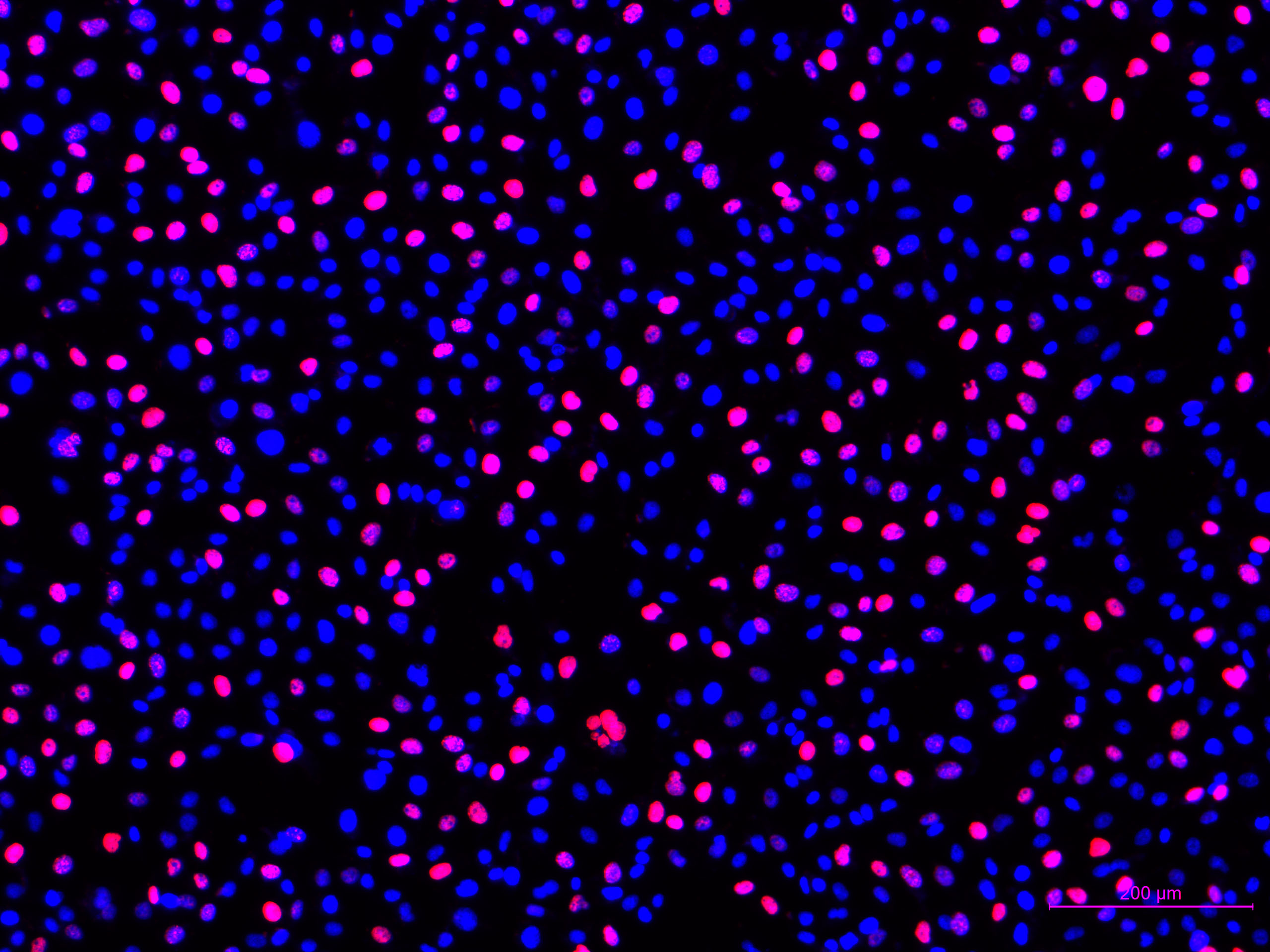

Supplement: Supplementary file 4 [file DataSheet_1.zip › Data Sheet 1/Fig2D/1-overAC009948.5.jpg]

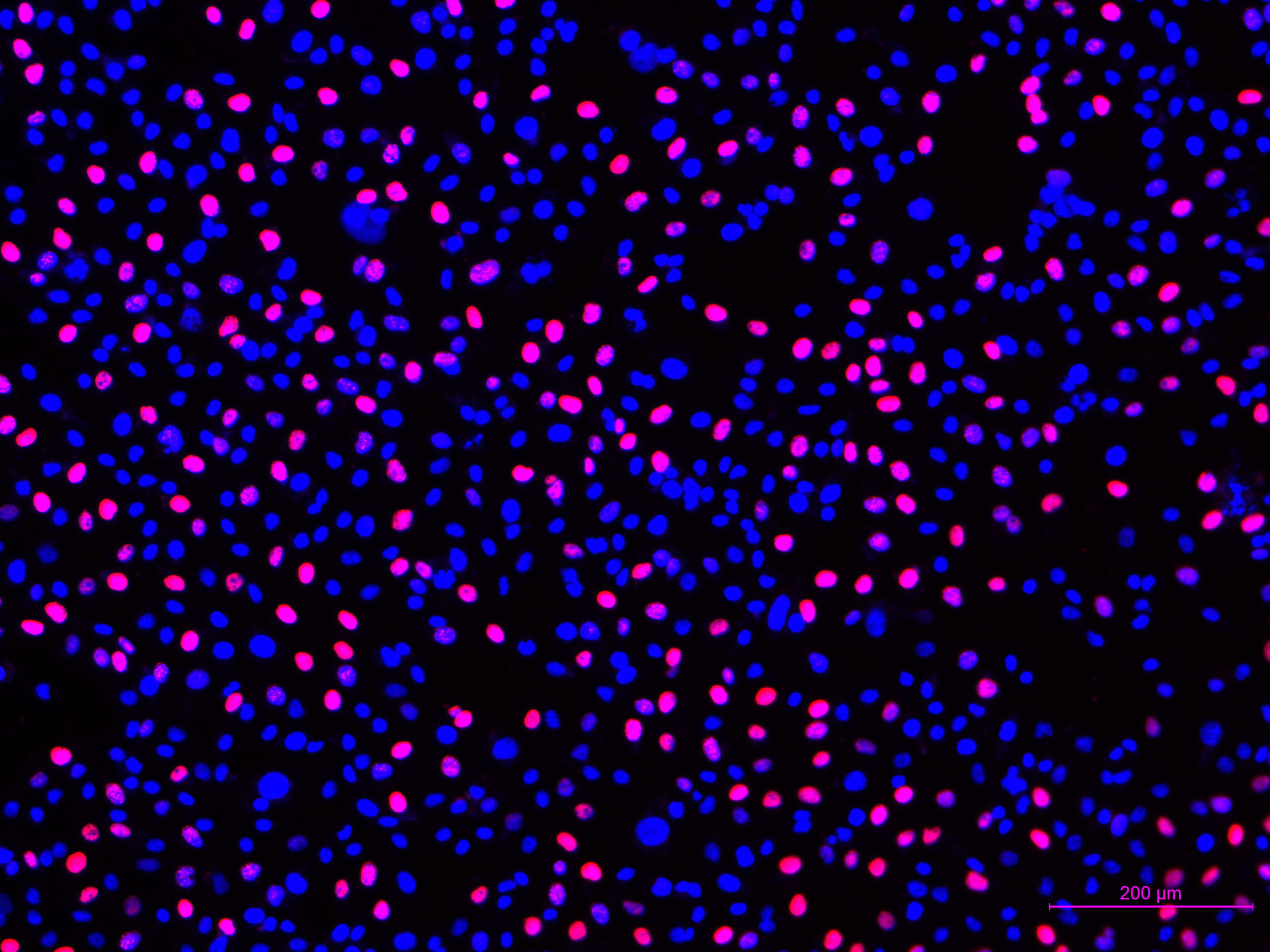

Supplement: Supplementary file 4 [file DataSheet_1.zip › Data Sheet 1/Fig2D/1-Scrambled-AC009948.5.jpg]

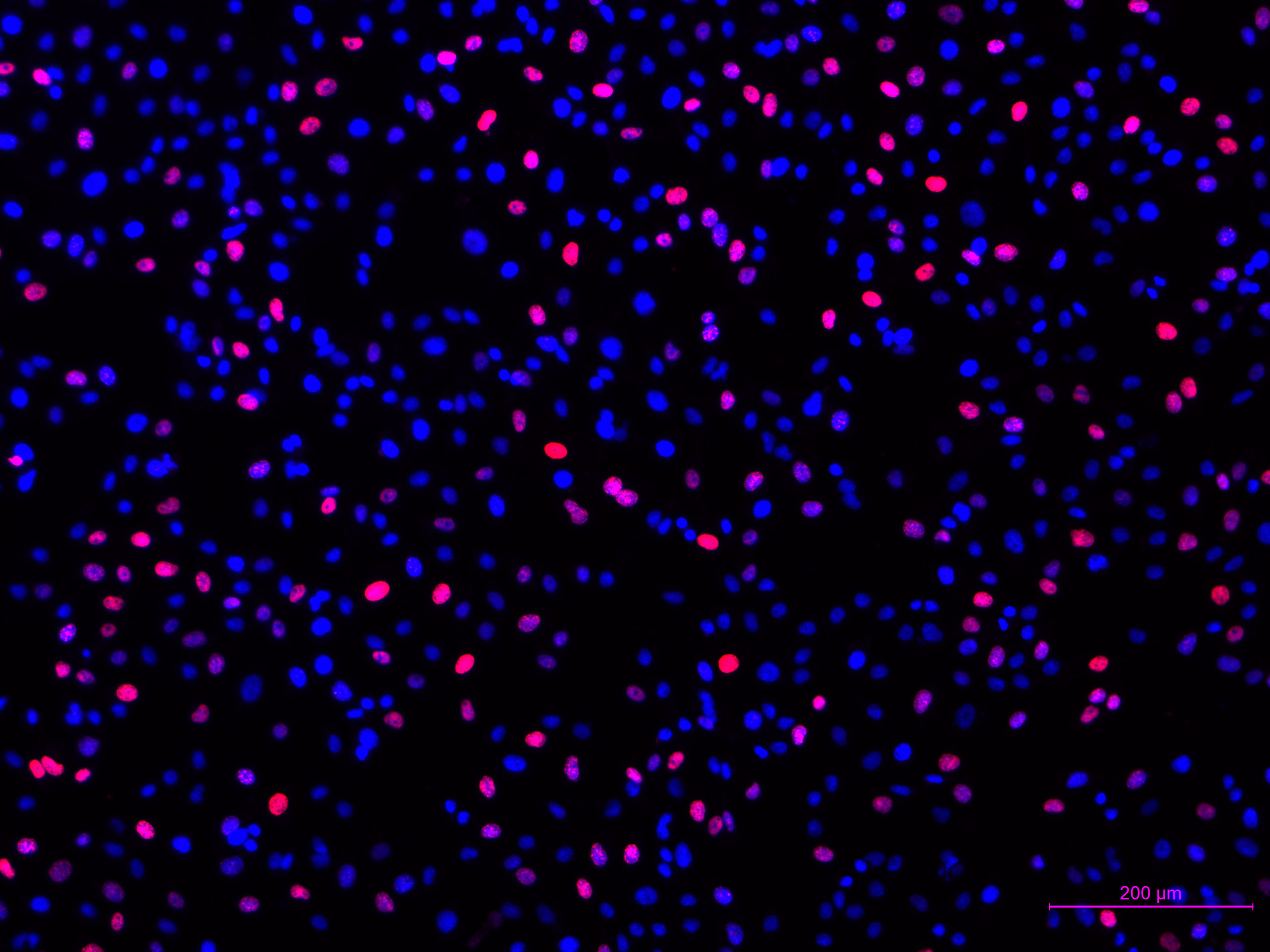

Supplement: Supplementary file 4 [file DataSheet_1.zip › Data Sheet 1/Fig2D/1-SiAC009948.5.jpg]

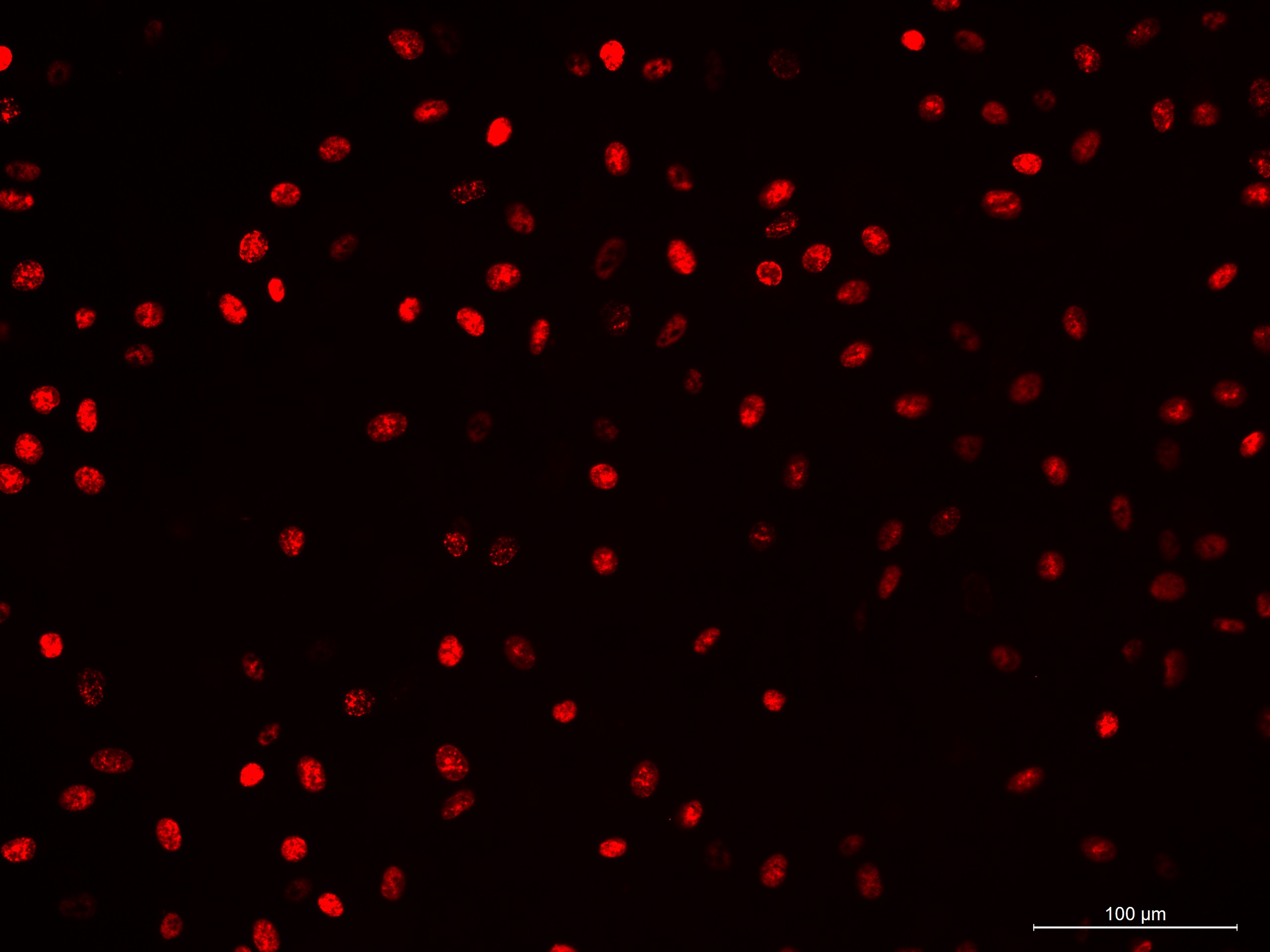

Supplement: Supplementary file 4 [file DataSheet_1.zip › Data Sheet 1/Fig2D/2-1-NC-AC009948.5.jpg]

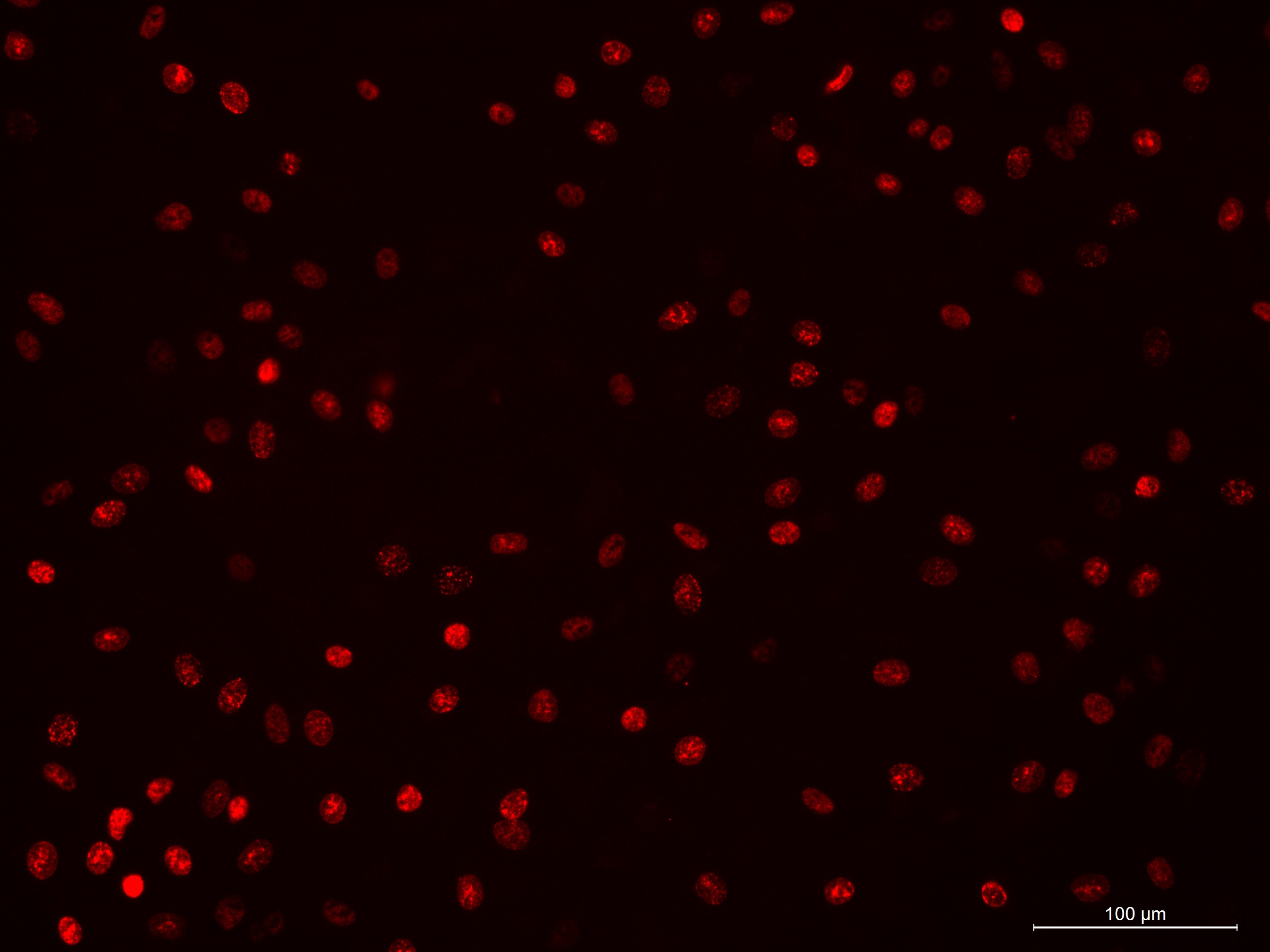

Supplement: Supplementary file 4 [file DataSheet_1.zip › Data Sheet 1/Fig2D/2-1-overAC009948.5.jpg]

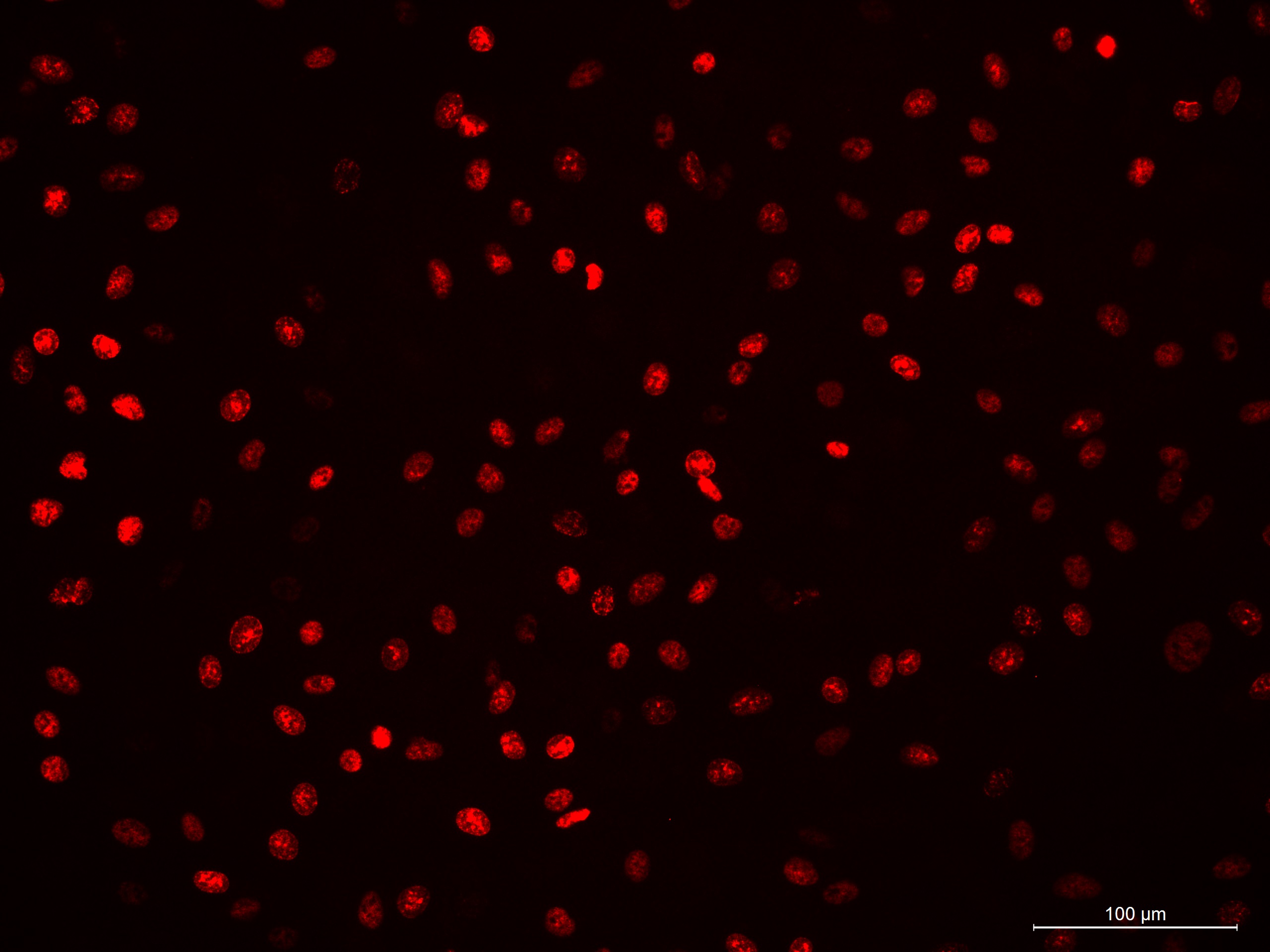

Supplement: Supplementary file 4 [file DataSheet_1.zip › Data Sheet 1/Fig2D/2-1-Scrambled-AC009948.5.jpg]

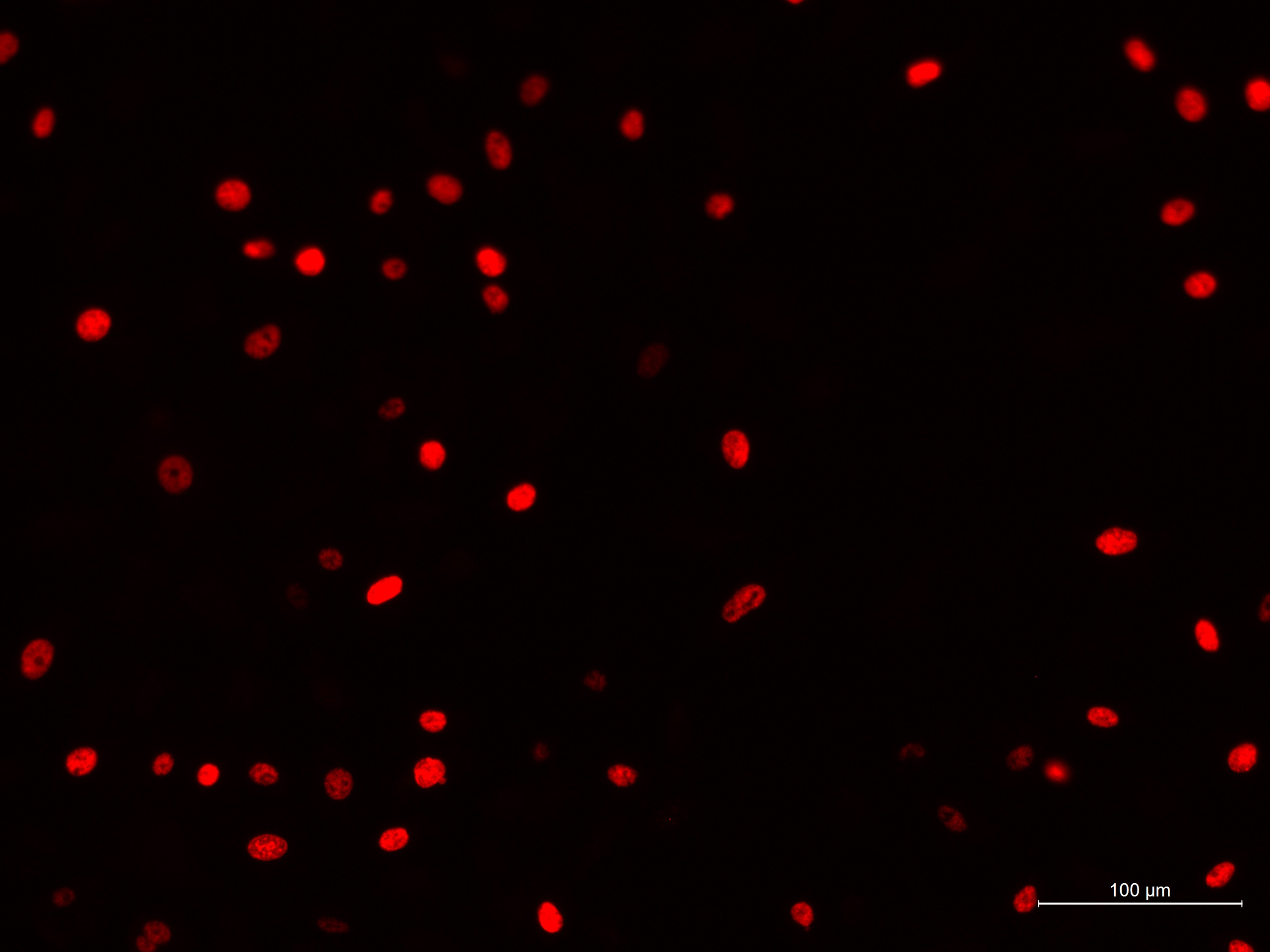

Supplement: Supplementary file 4 [file DataSheet_1.zip › Data Sheet 1/Fig2D/2-1-SiAC009948.5.jpg]

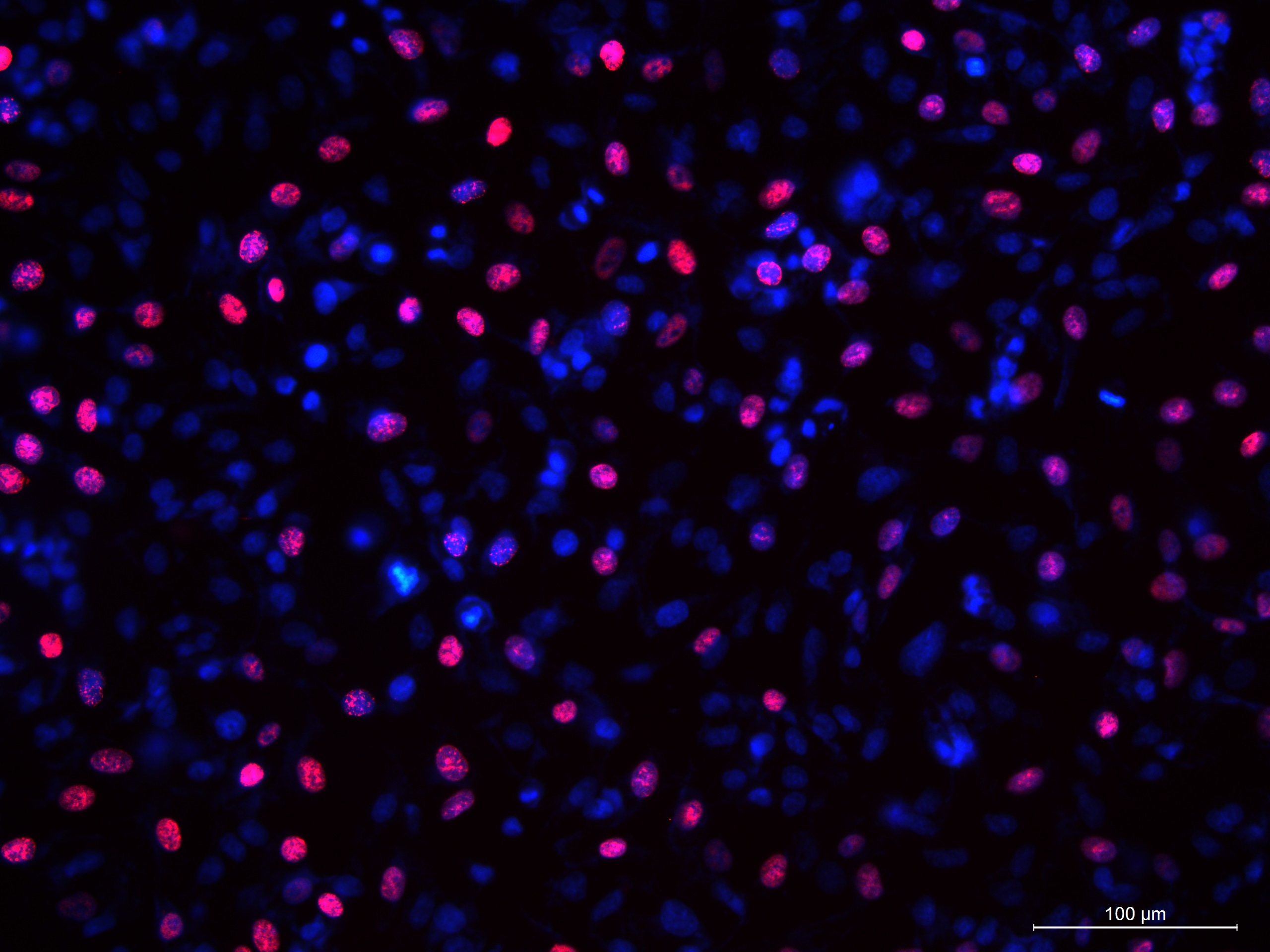

Supplement: Supplementary file 4 [file DataSheet_1.zip › Data Sheet 1/Fig2D/2-NC-AC009948.5.jpg]

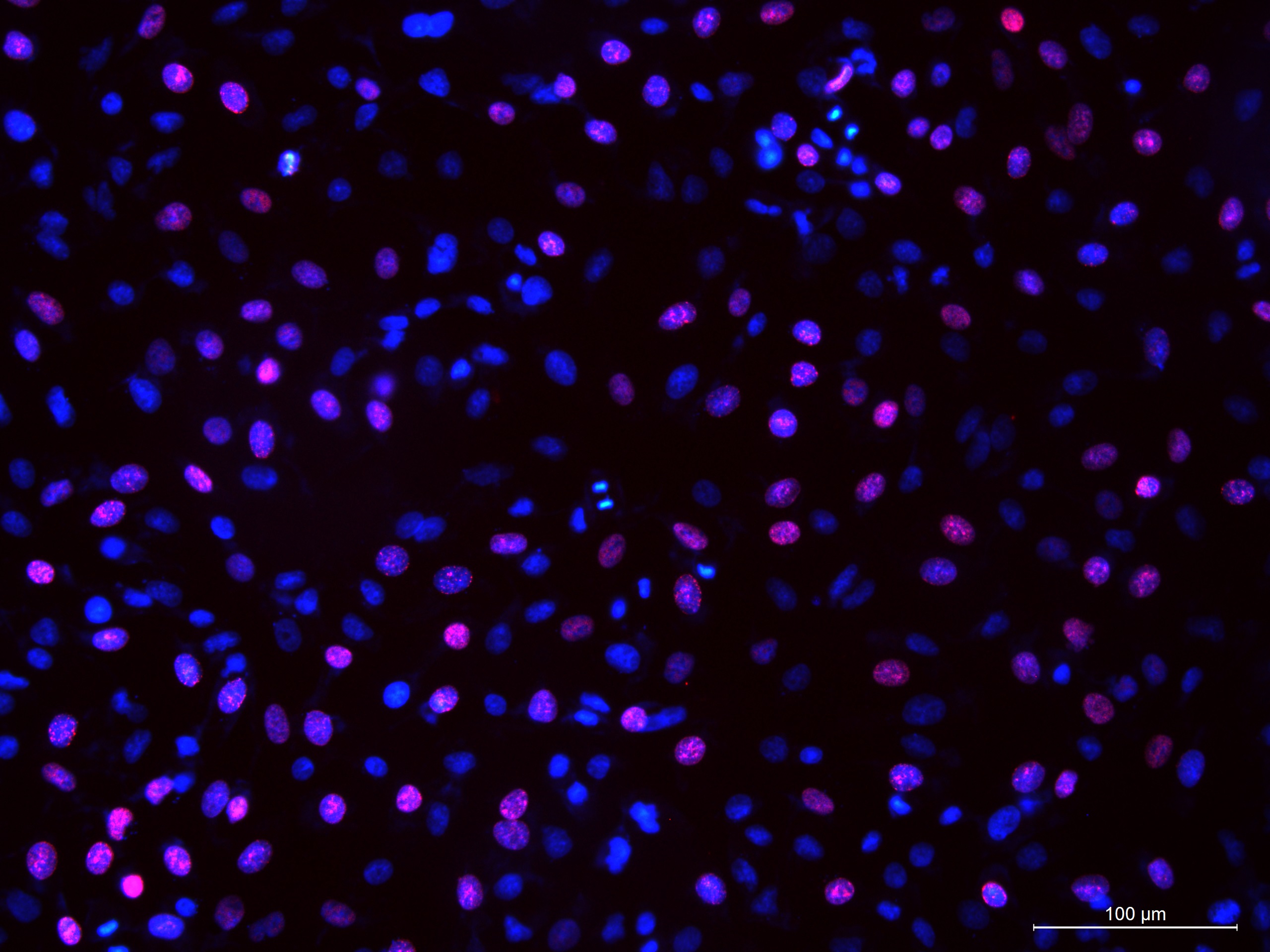

Supplement: Supplementary file 4 [file DataSheet_1.zip › Data Sheet 1/Fig2D/2-overAC009948.5.jpg]

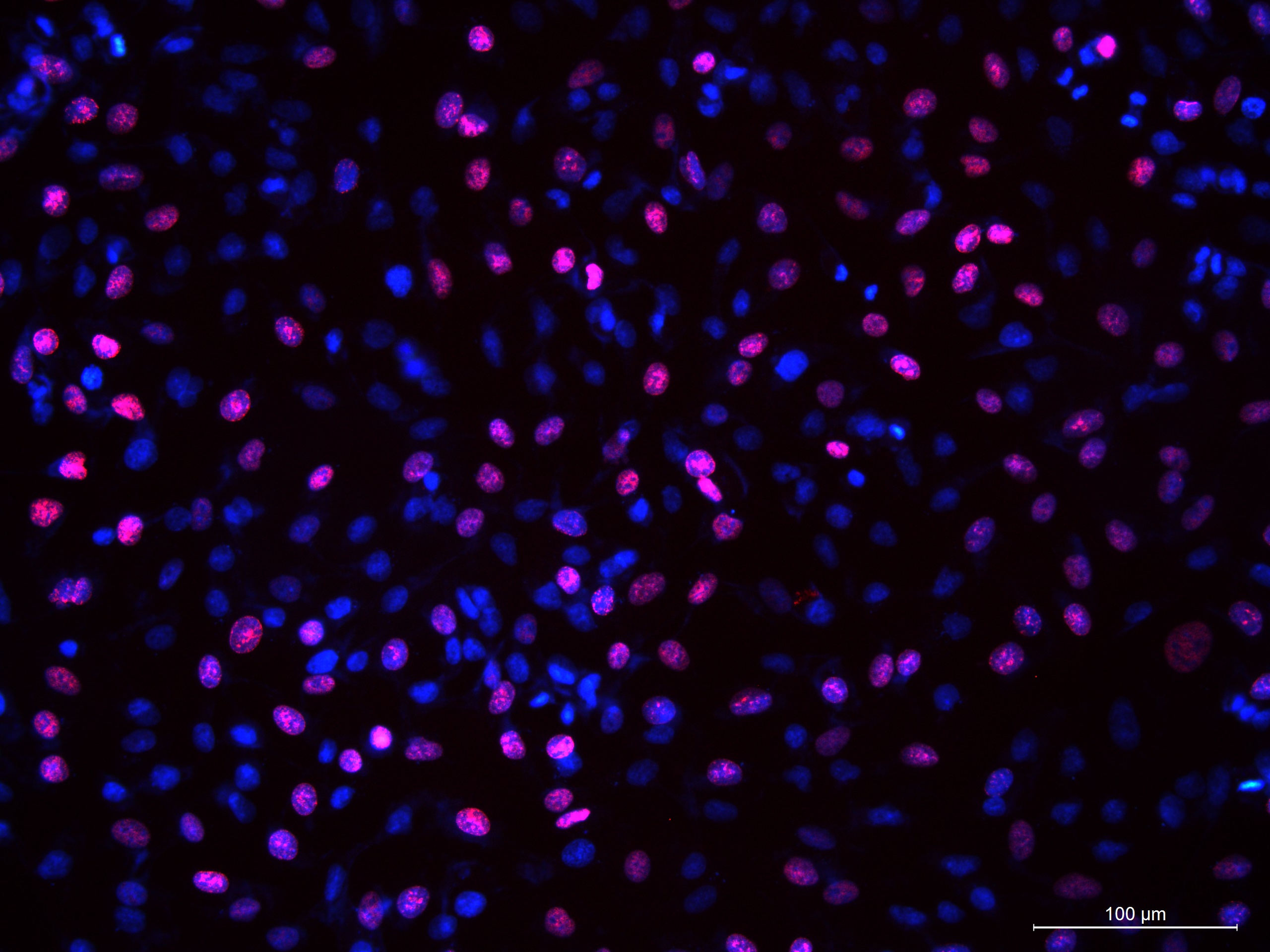

Supplement: Supplementary file 4 [file DataSheet_1.zip › Data Sheet 1/Fig2D/2-Scrambled-AC009948.5.jpg]

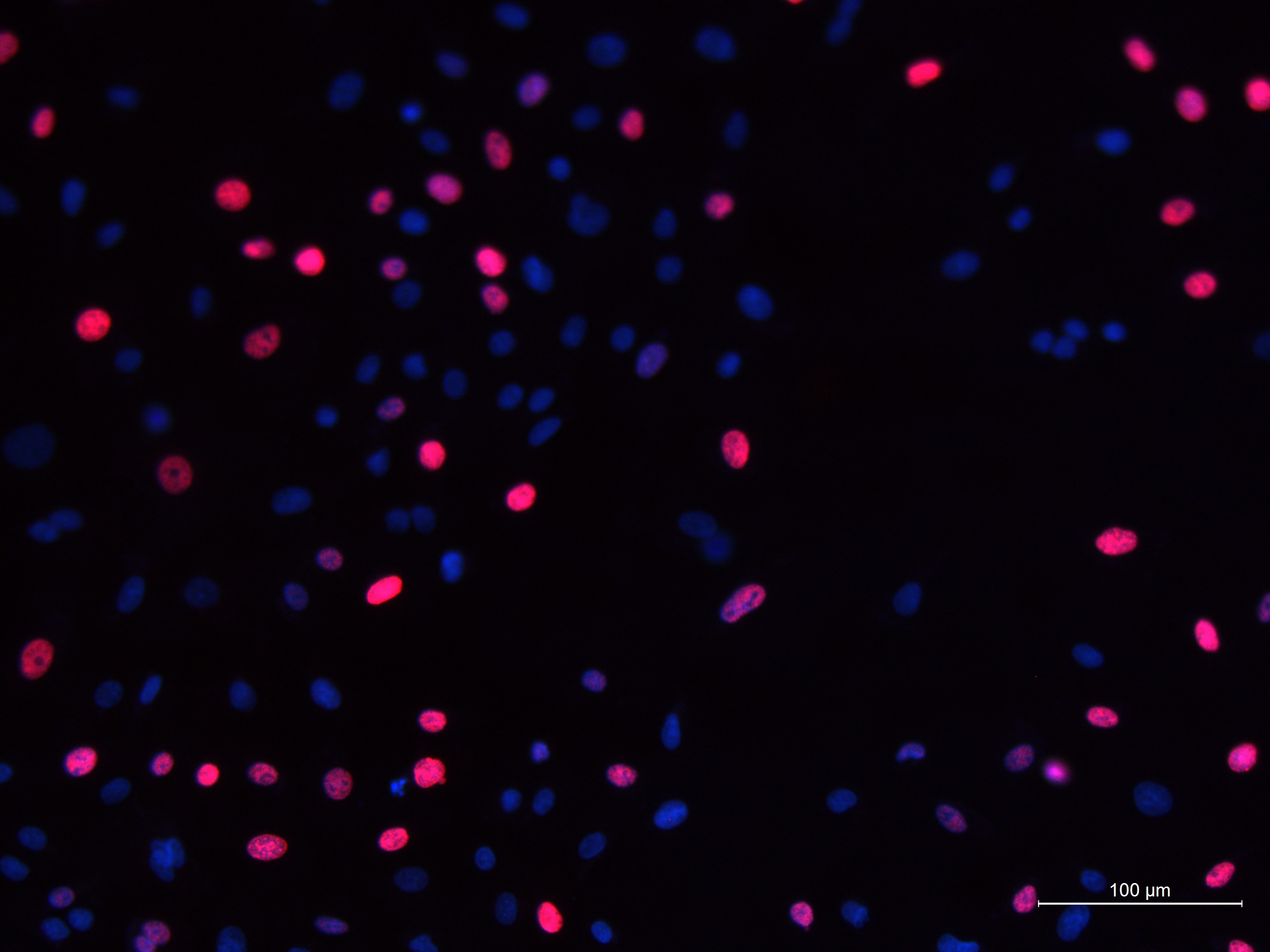

Supplement: Supplementary file 4 [file DataSheet_1.zip › Data Sheet 1/Fig2D/2-SiAC009948.5.jpg]

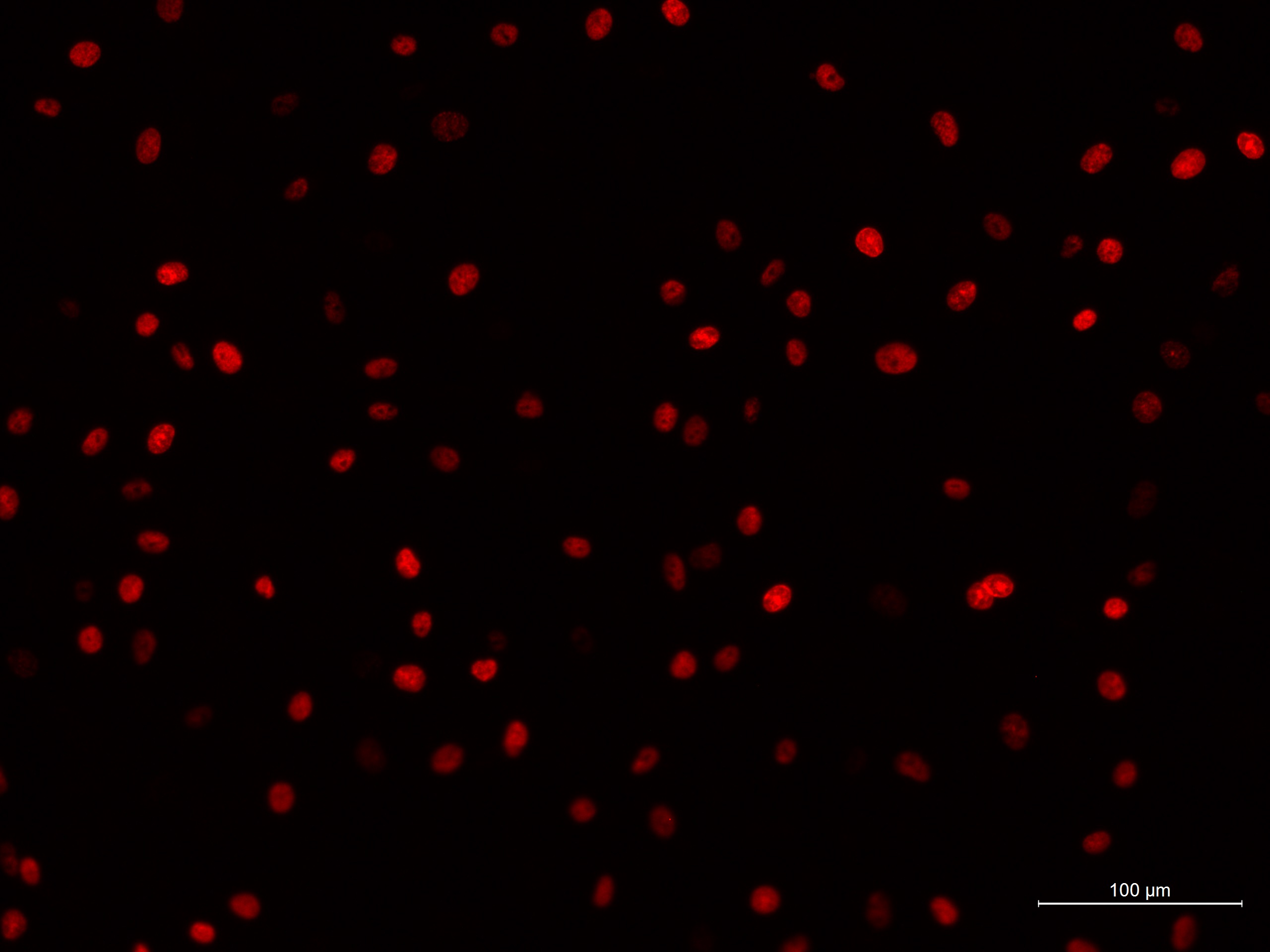

Supplement: Supplementary file 4 [file DataSheet_1.zip › Data Sheet 1/Fig2D/3-1-NC-AC009948.5.jpg]

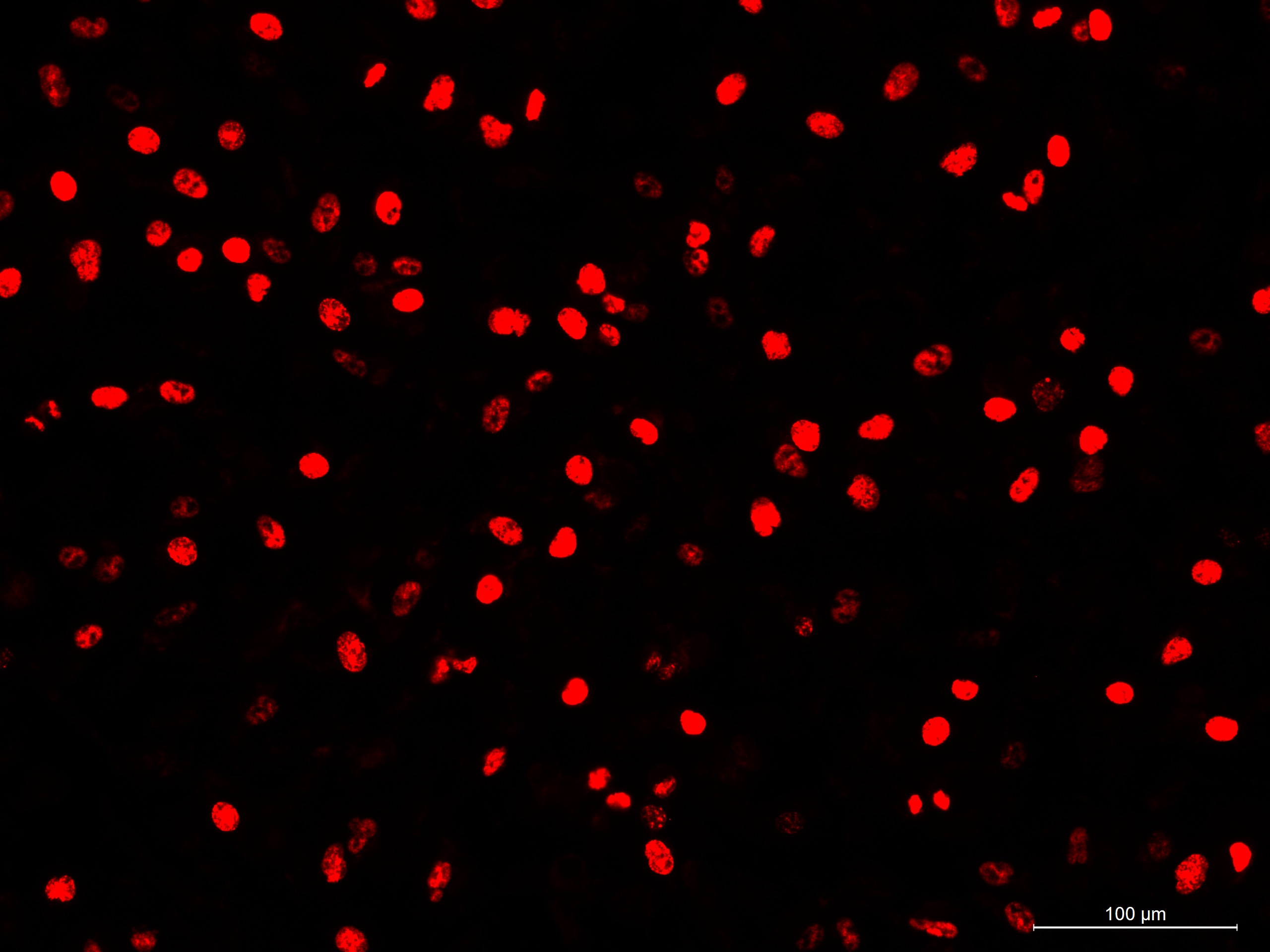

Supplement: Supplementary file 4 [file DataSheet_1.zip › Data Sheet 1/Fig2D/3-1-overAC009948.5.jpg]

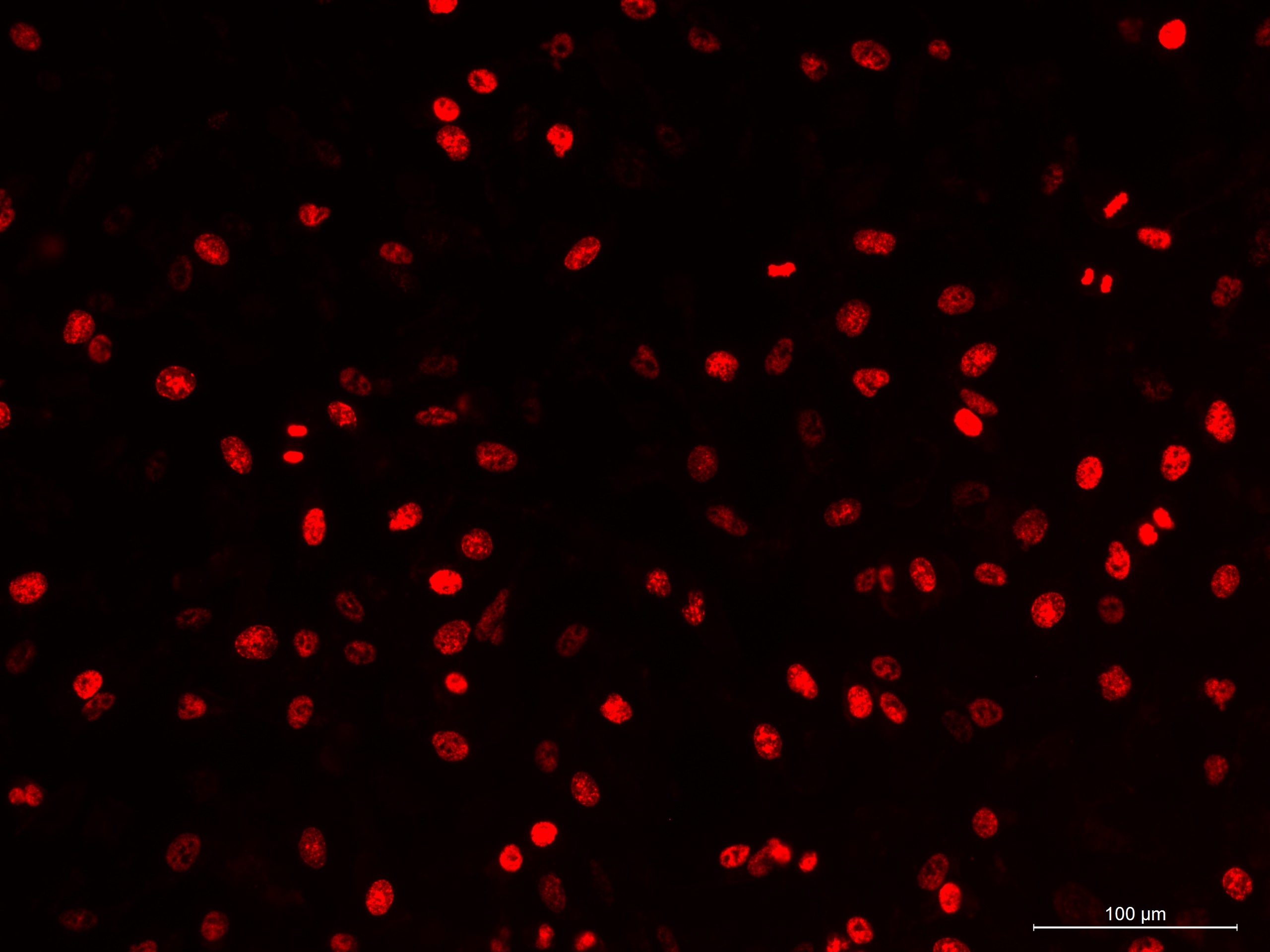

Supplement: Supplementary file 4 [file DataSheet_1.zip › Data Sheet 1/Fig2D/3-1-Scrambled-AC009948.5.jpg]

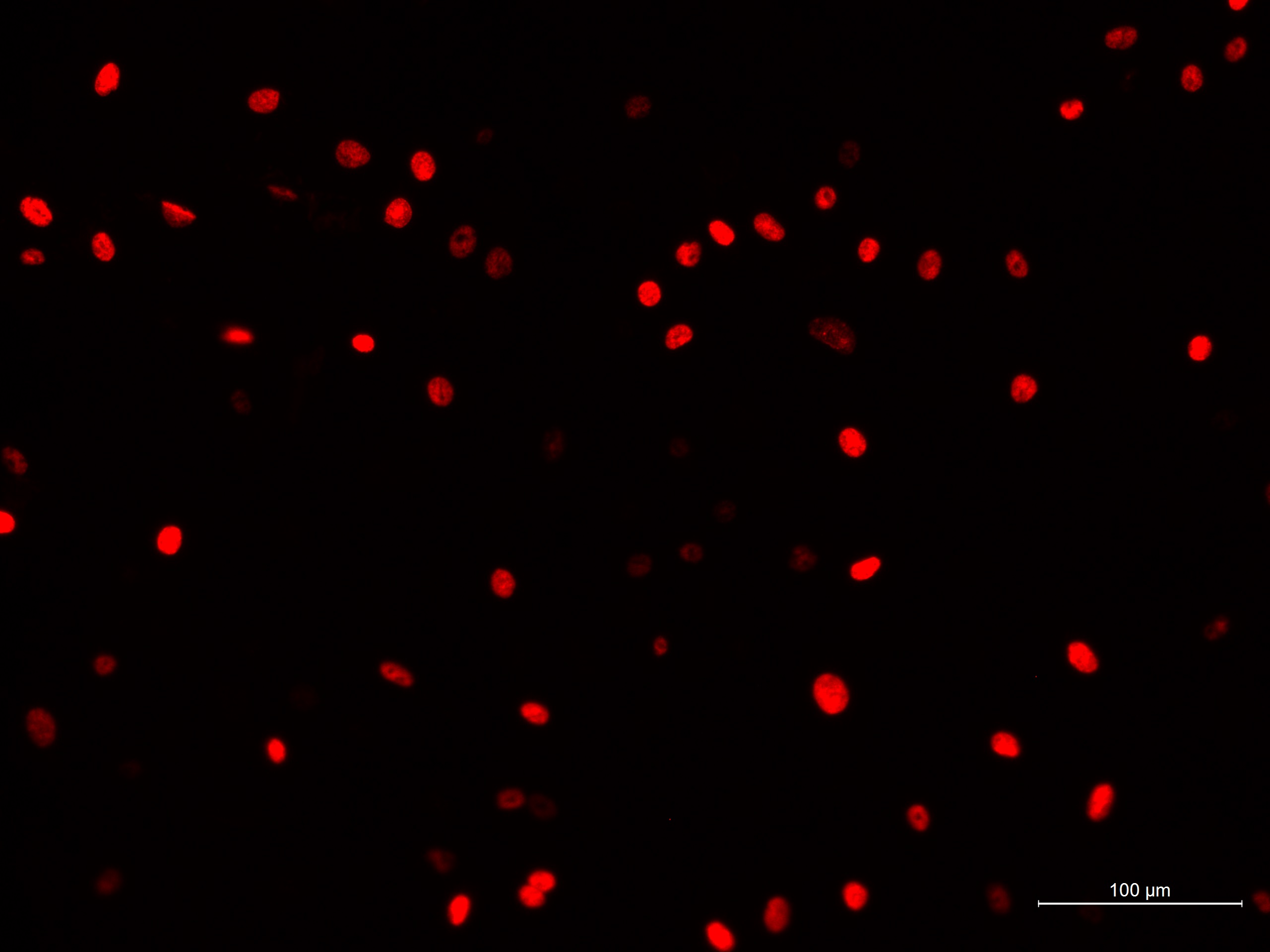

Supplement: Supplementary file 4 [file DataSheet_1.zip › Data Sheet 1/Fig2D/3-1-SiAC009948.5.jpg]

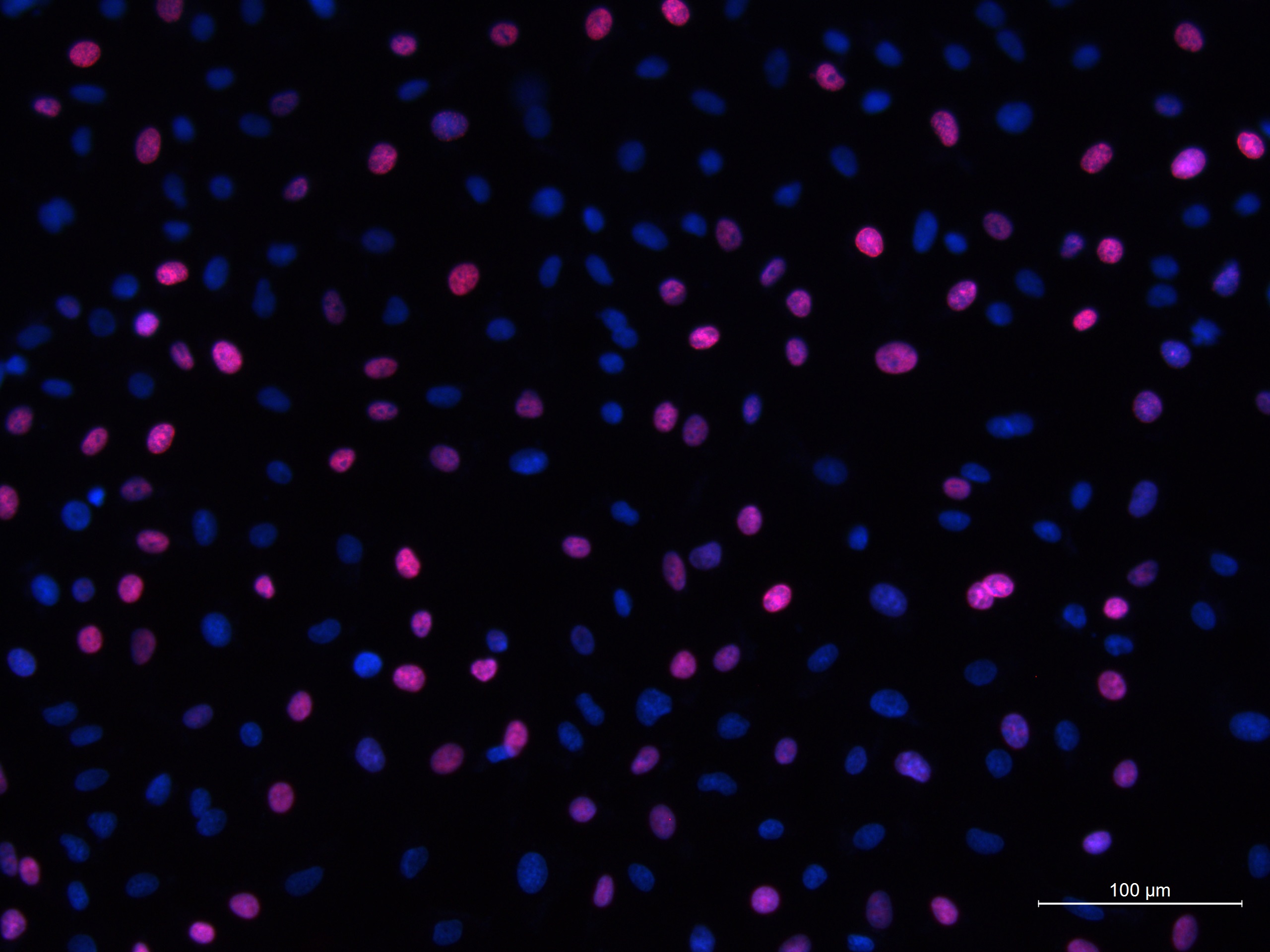

Supplement: Supplementary file 4 [file DataSheet_1.zip › Data Sheet 1/Fig2D/3-NC-AC009948.5.jpg]

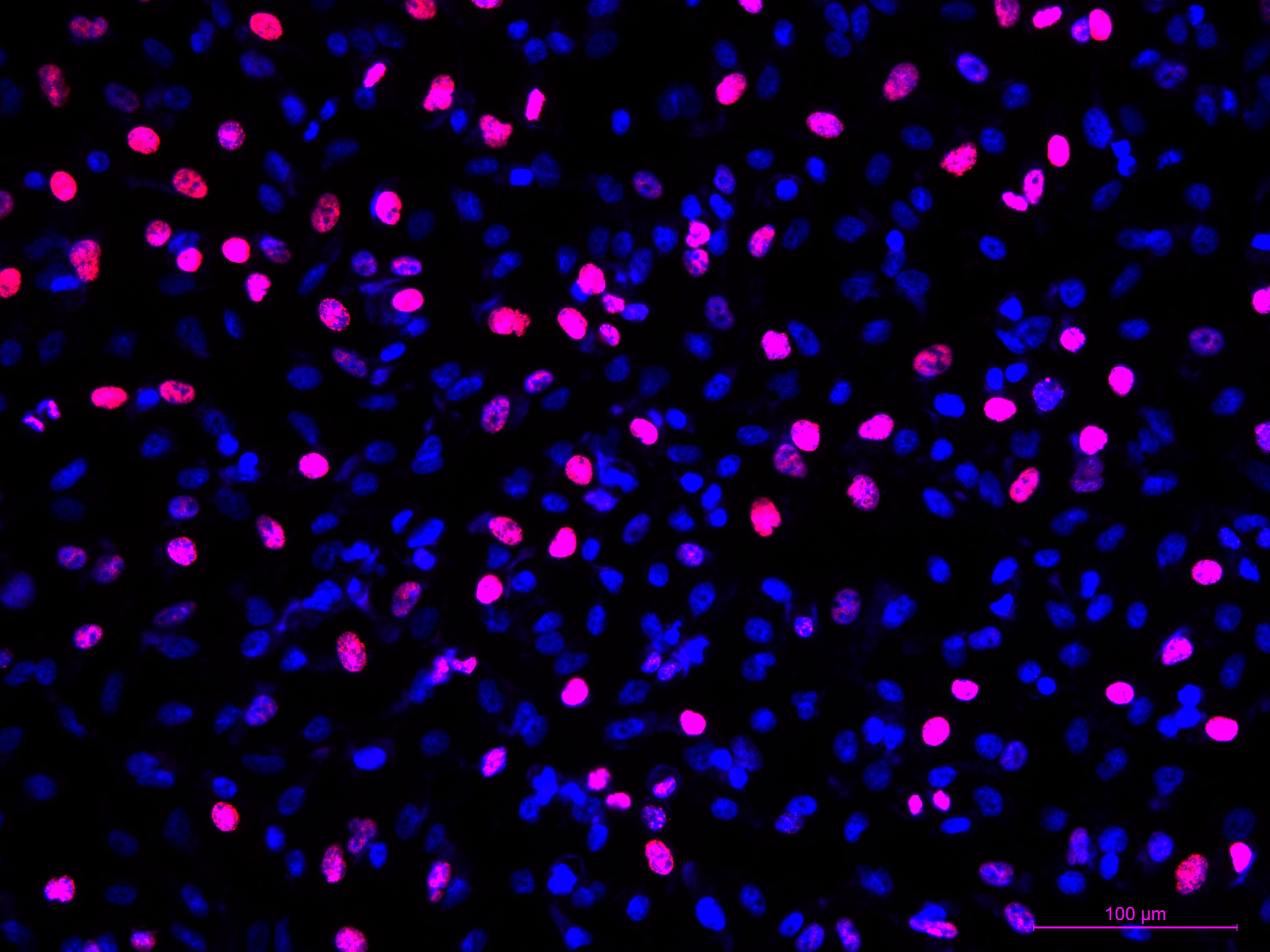

Supplement: Supplementary file 4 [file DataSheet_1.zip › Data Sheet 1/Fig2D/3-overAC009948.5.jpg]

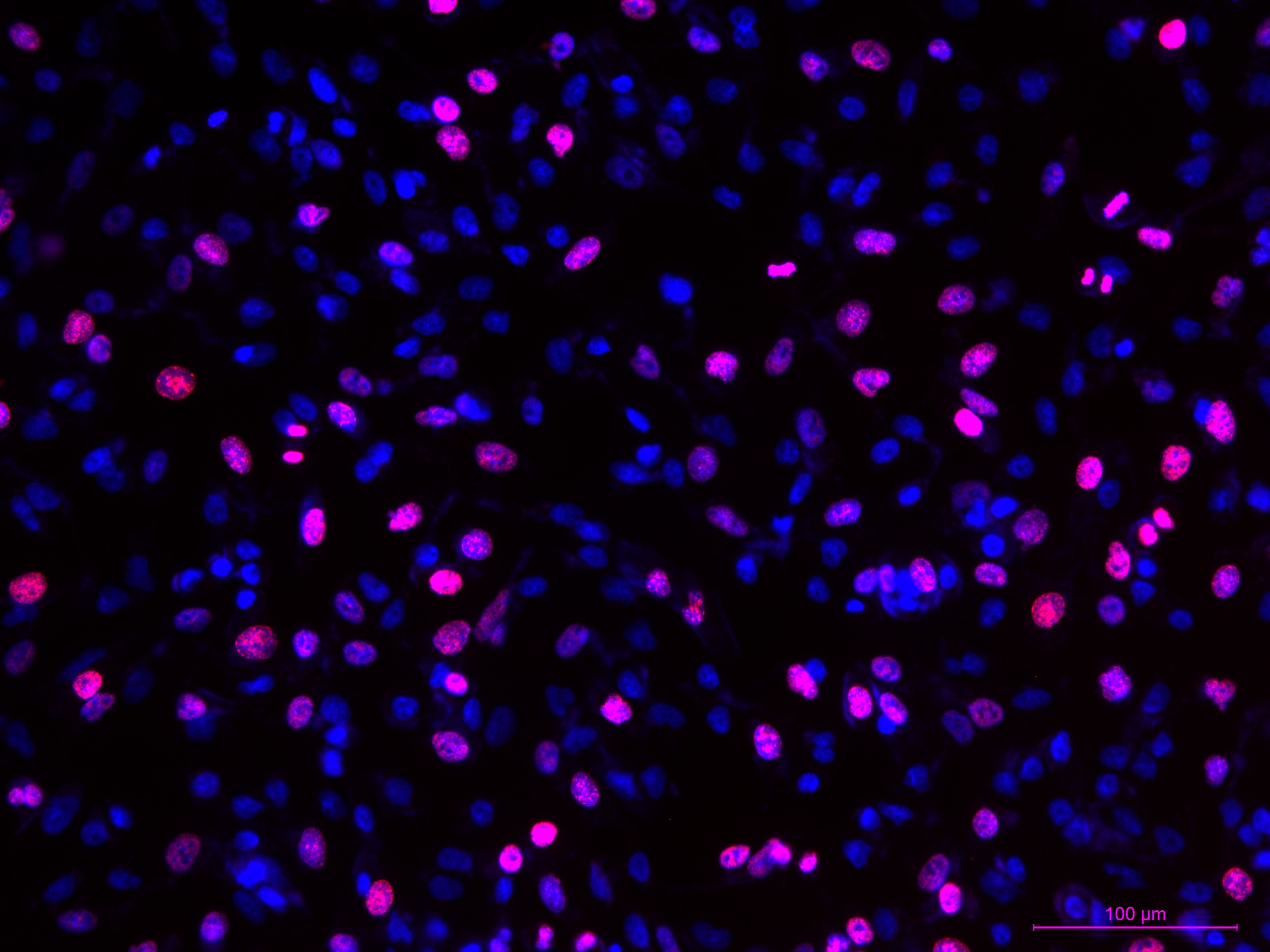

Supplement: Supplementary file 4 [file DataSheet_1.zip › Data Sheet 1/Fig2D/3-Scrambled-AC009948.5.jpg]

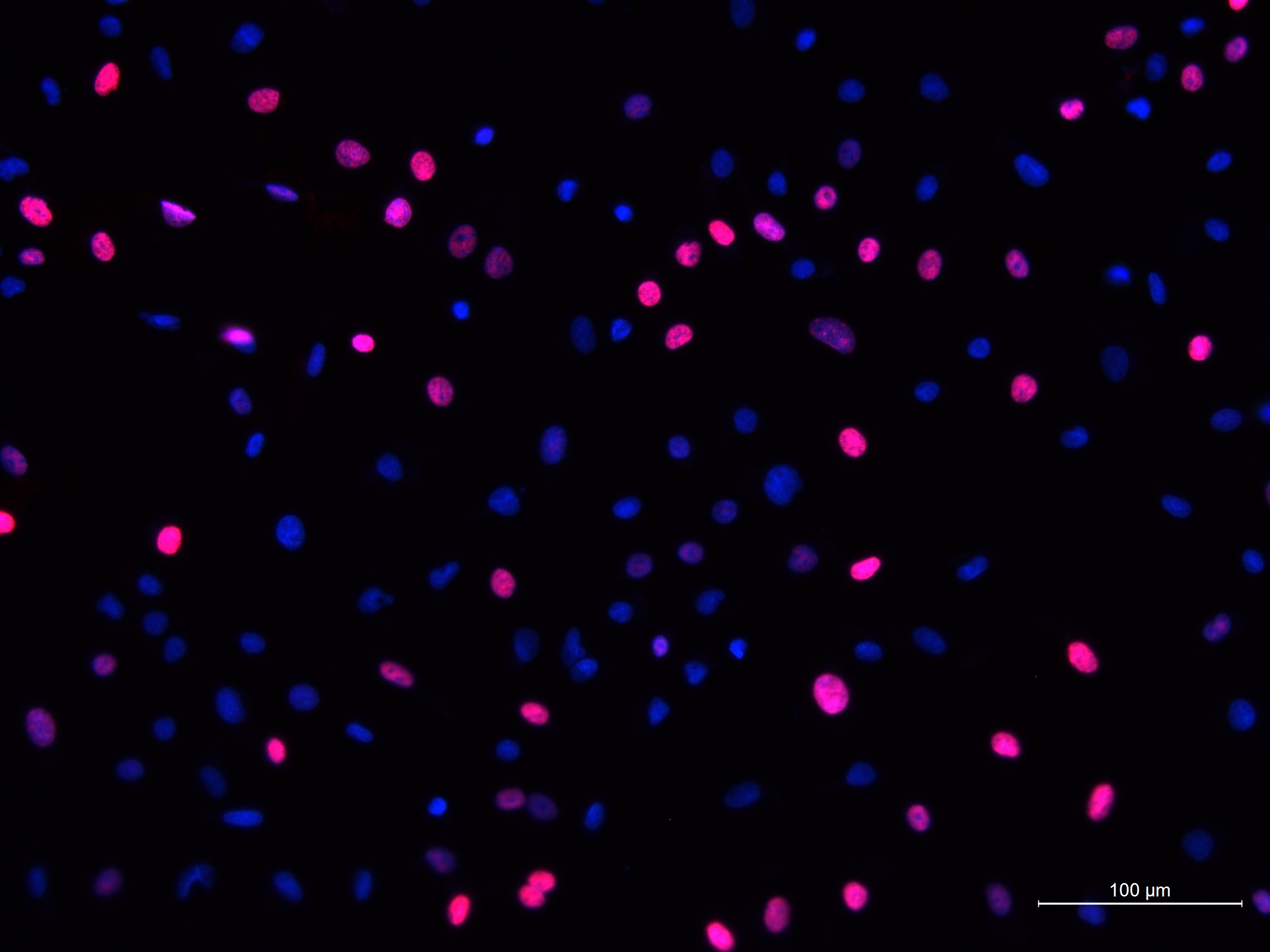

Supplement: Supplementary file 4 [file DataSheet_1.zip › Data Sheet 1/Fig2D/3-SiAC009948.5.jpg]

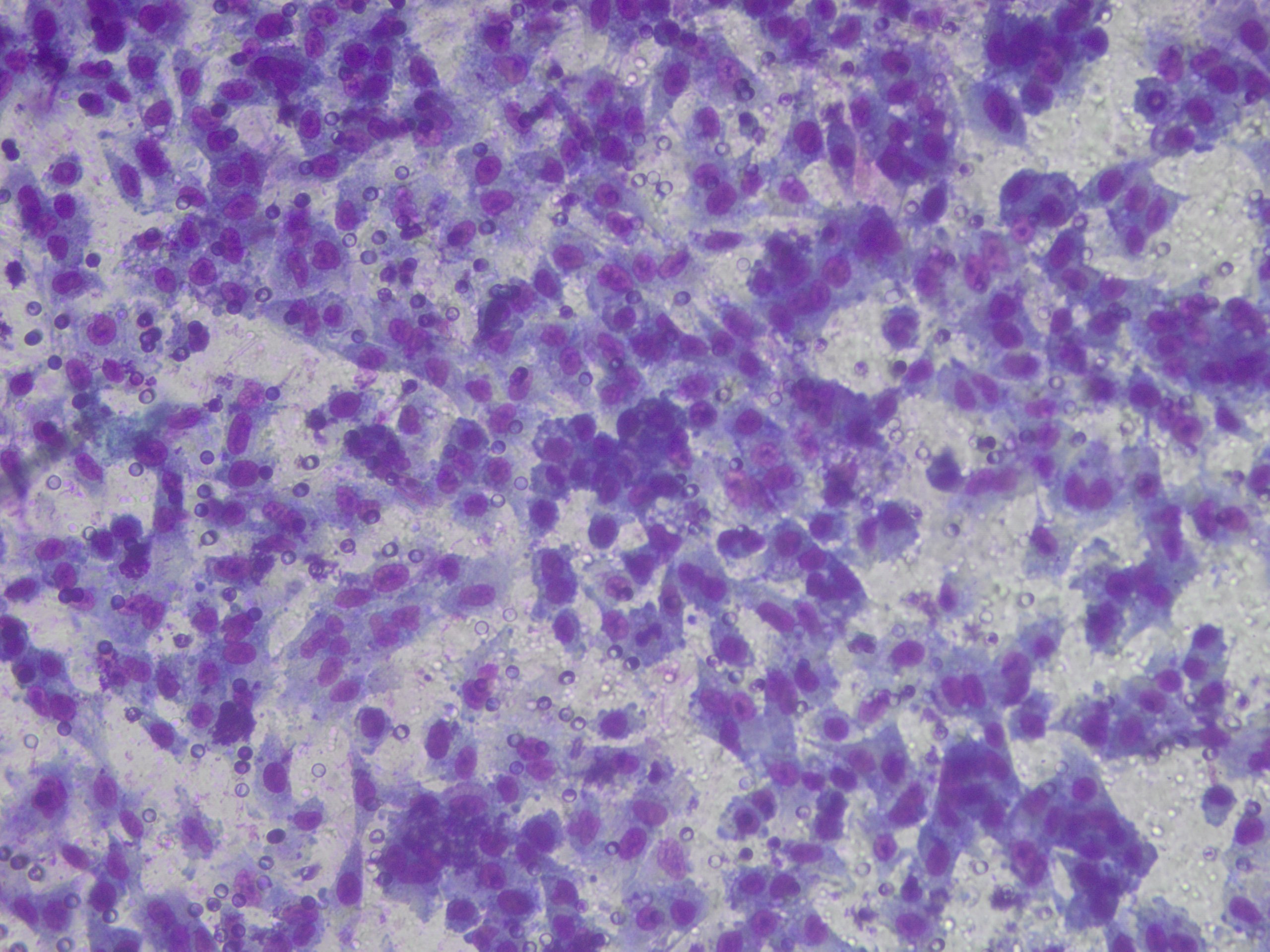

Supplement: Supplementary file 5 [file DataSheet_2.zip › Data Sheet 2/Fig2E/1-AC009948.5-NC-A549-M.jpg]

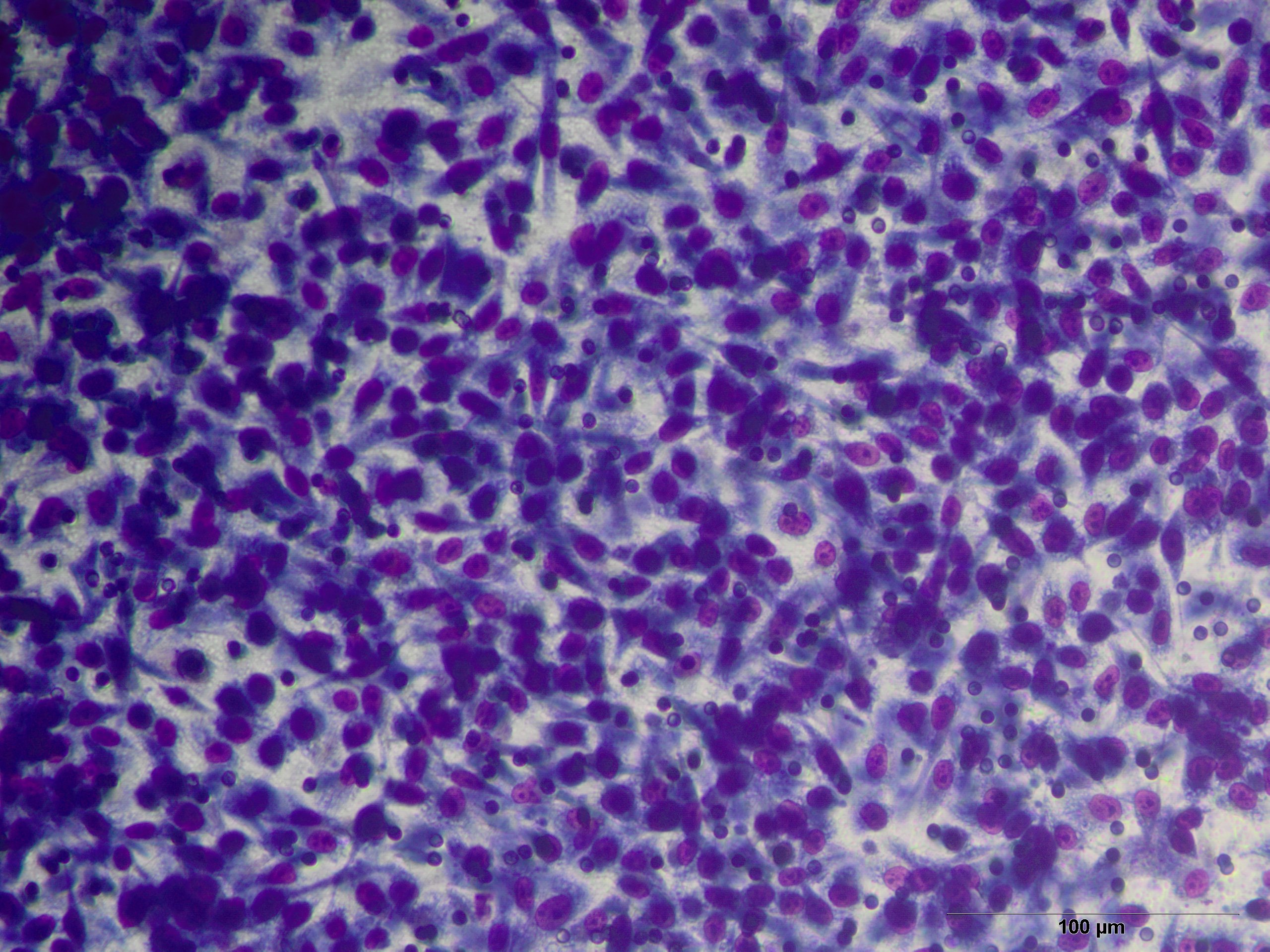

Supplement: Supplementary file 5 [file DataSheet_2.zip › Data Sheet 2/Fig2E/1-AC009948.5-over-A549-M.jpg]

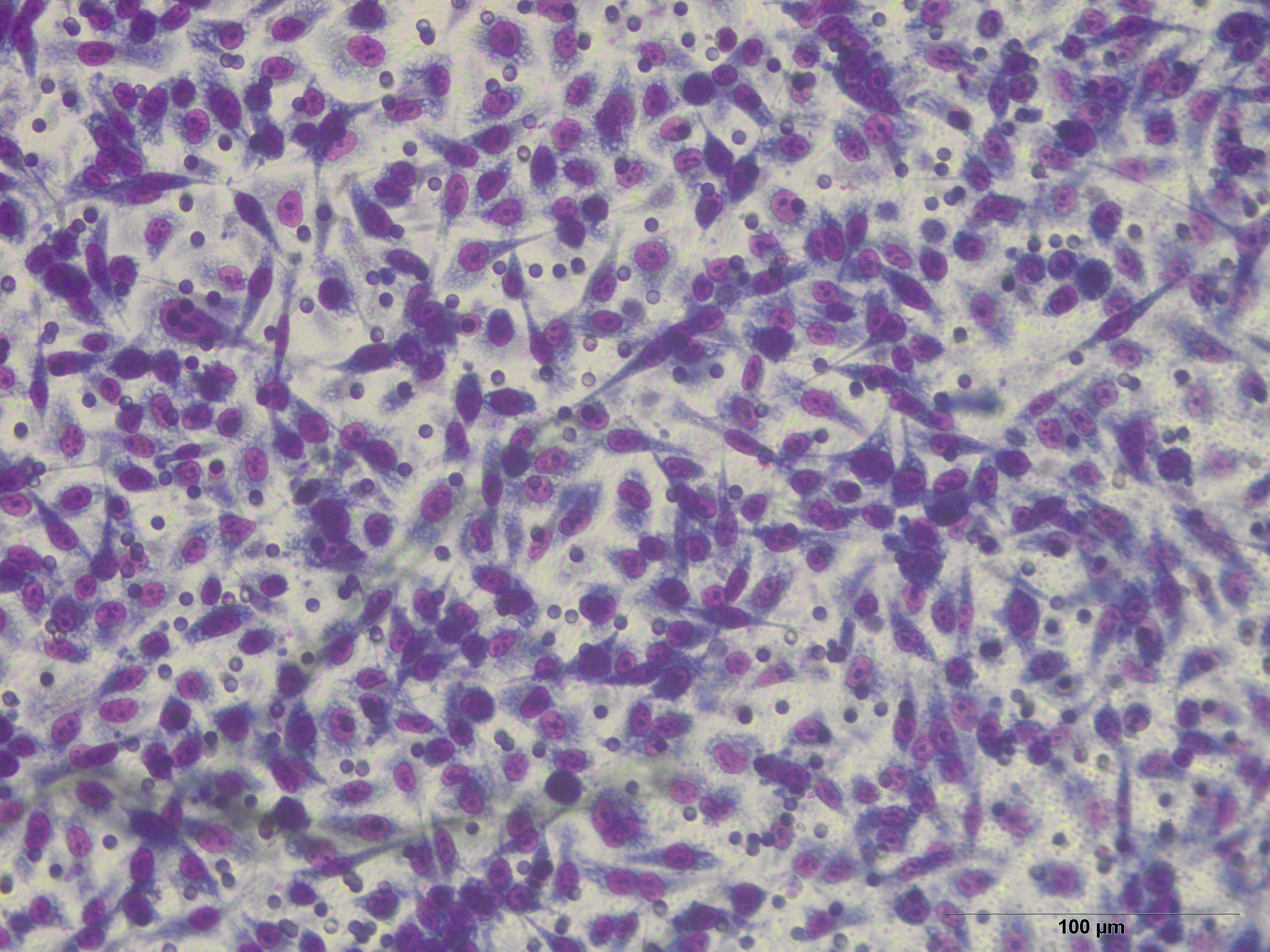

Supplement: Supplementary file 5 [file DataSheet_2.zip › Data Sheet 2/Fig2E/1-AC009948.5-Scrambled-A549-M.jpg]

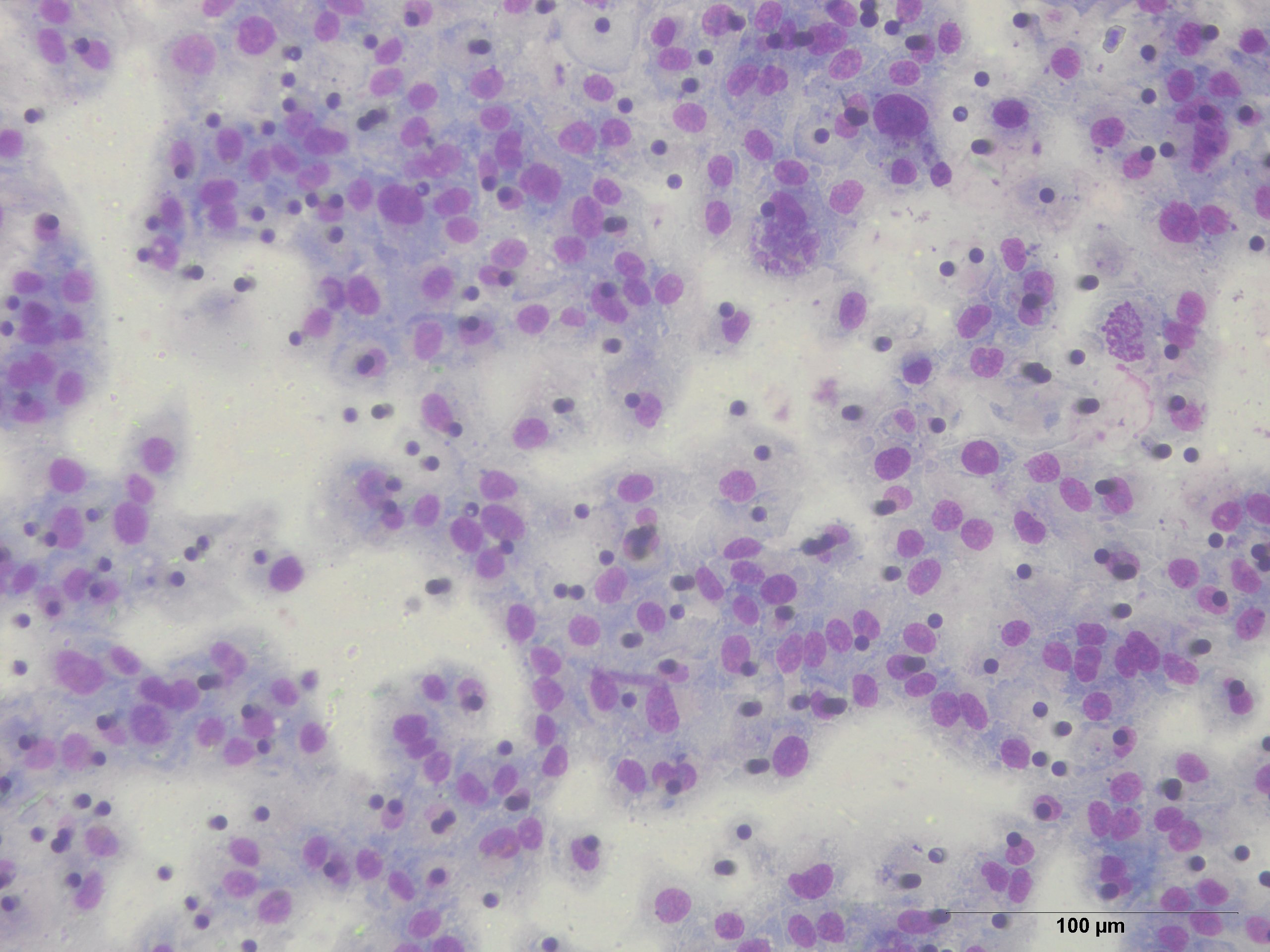

Supplement: Supplementary file 5 [file DataSheet_2.zip › Data Sheet 2/Fig2E/1-AC009948.5-Si-A549-M.jpg]

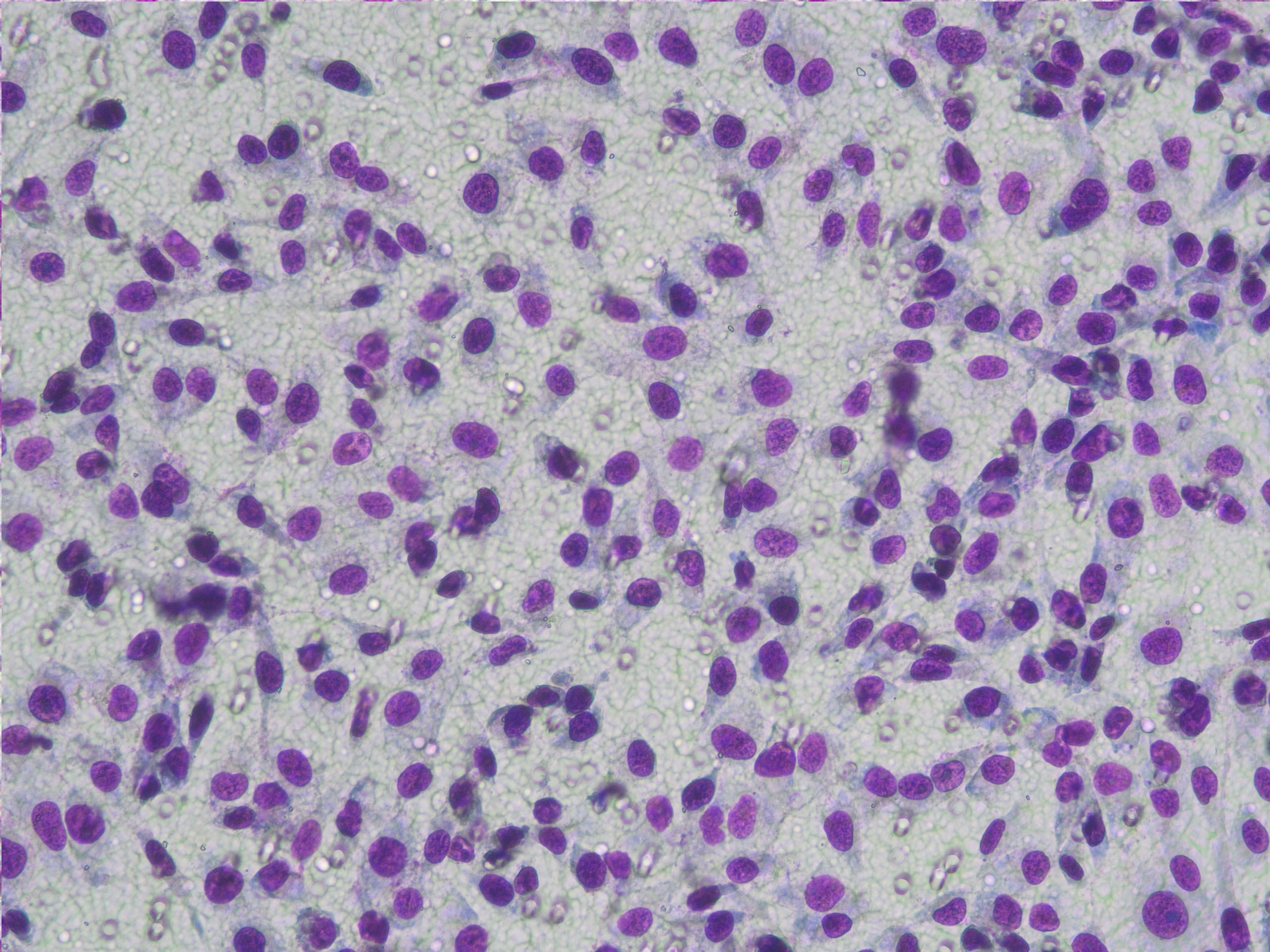

Supplement: Supplementary file 5 [file DataSheet_2.zip › Data Sheet 2/Fig2E/1-NC-AC009948.5-A549-INVASION.jpg]

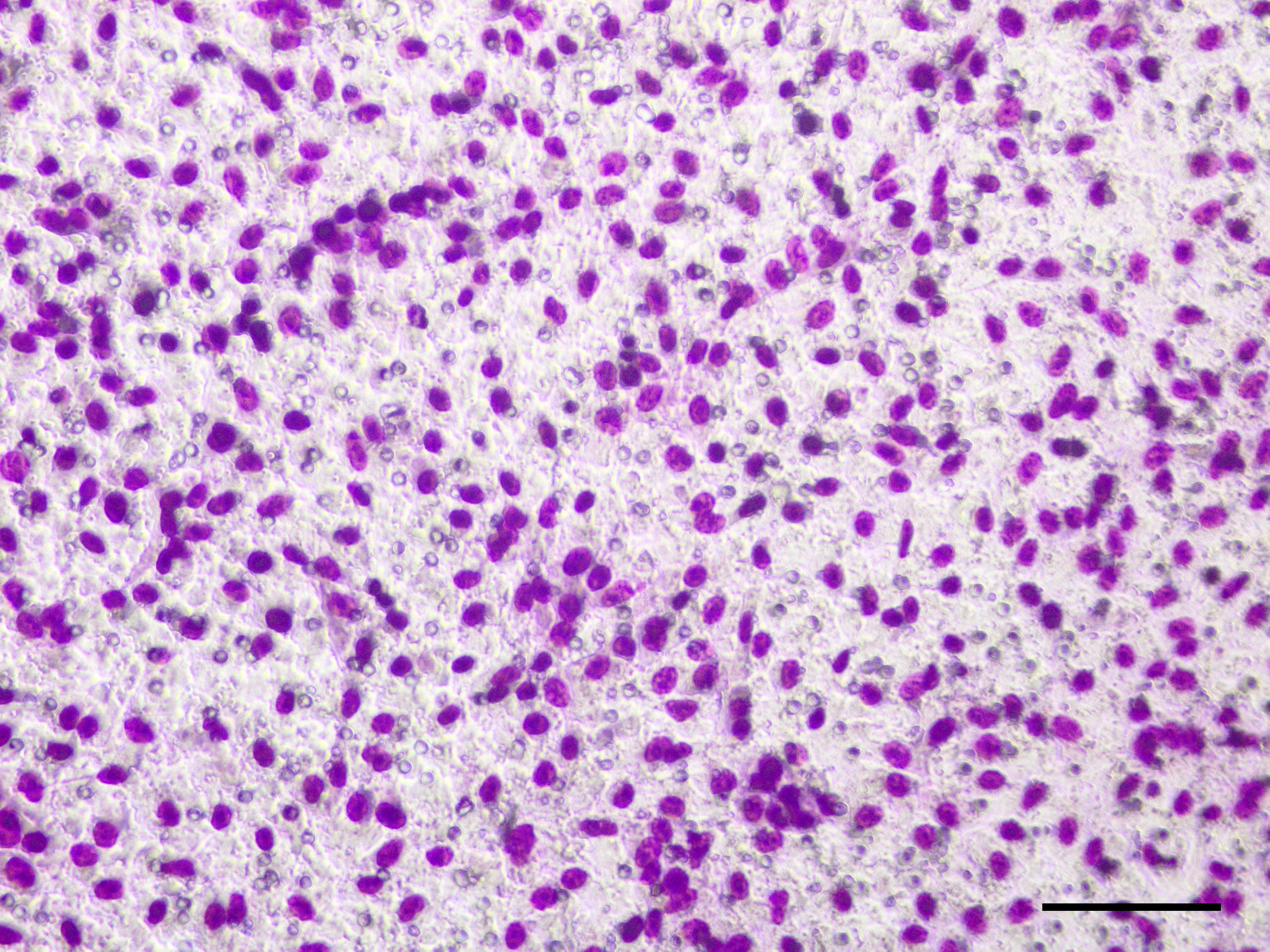

Supplement: Supplementary file 5 [file DataSheet_2.zip › Data Sheet 2/Fig2E/1-over-AC009948.5-INVASION.jpg]

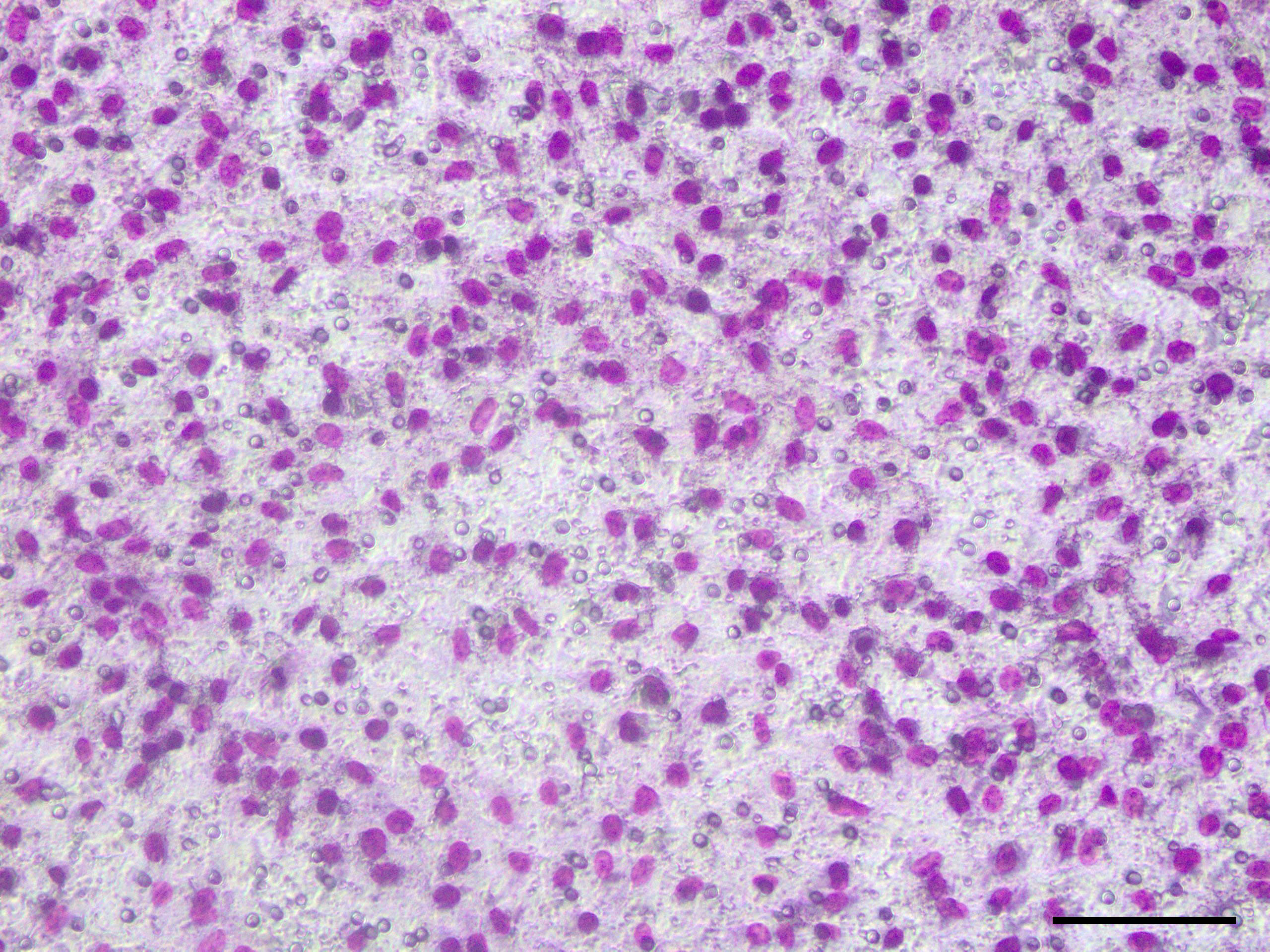

Supplement: Supplementary file 5 [file DataSheet_2.zip › Data Sheet 2/Fig2E/1-Scrambled-AC009948.5-A549-INVASION.jpg]

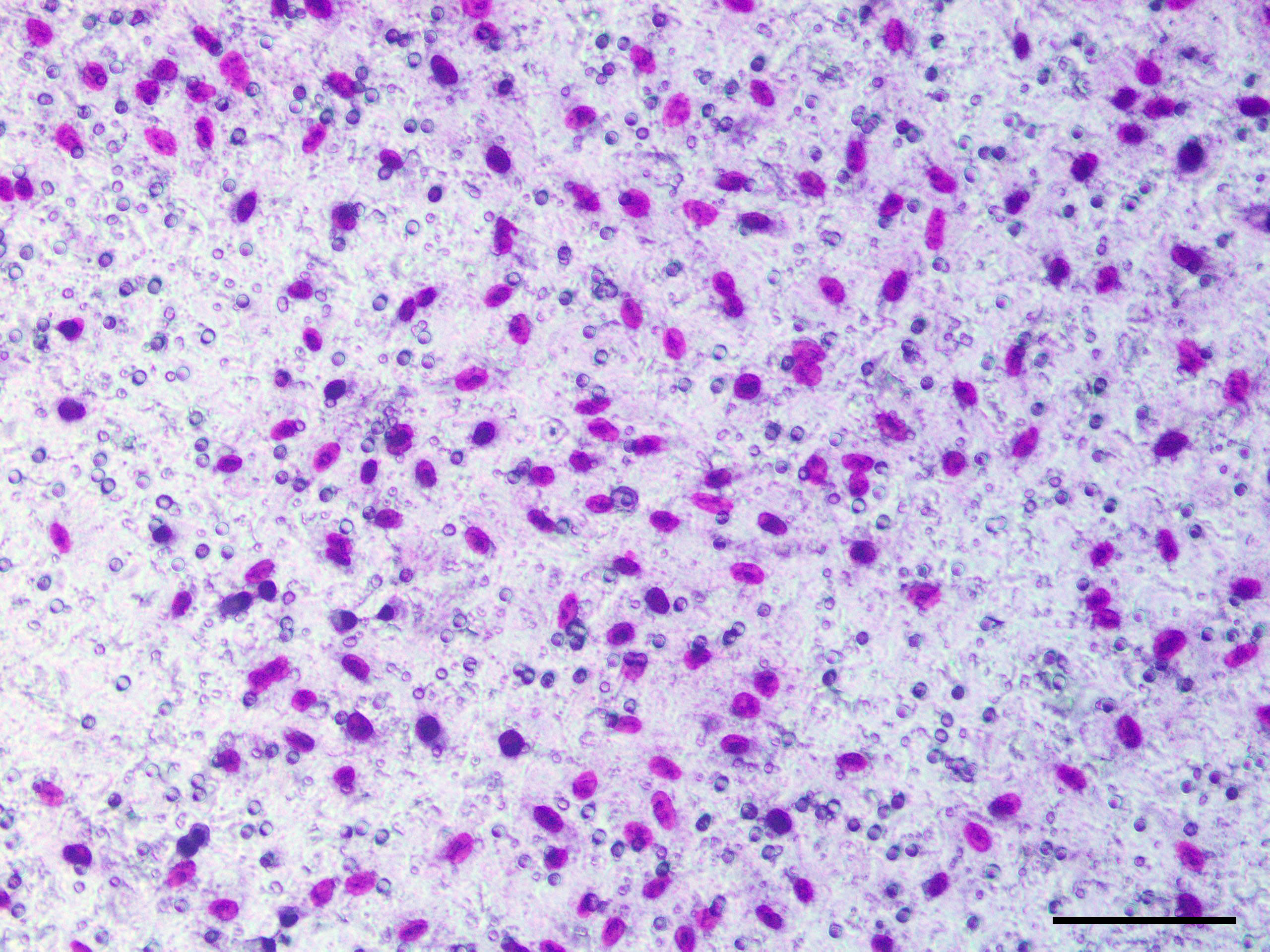

Supplement: Supplementary file 5 [file DataSheet_2.zip › Data Sheet 2/Fig2E/1-Si-AC009948.5-A549-INVASION.jpg]

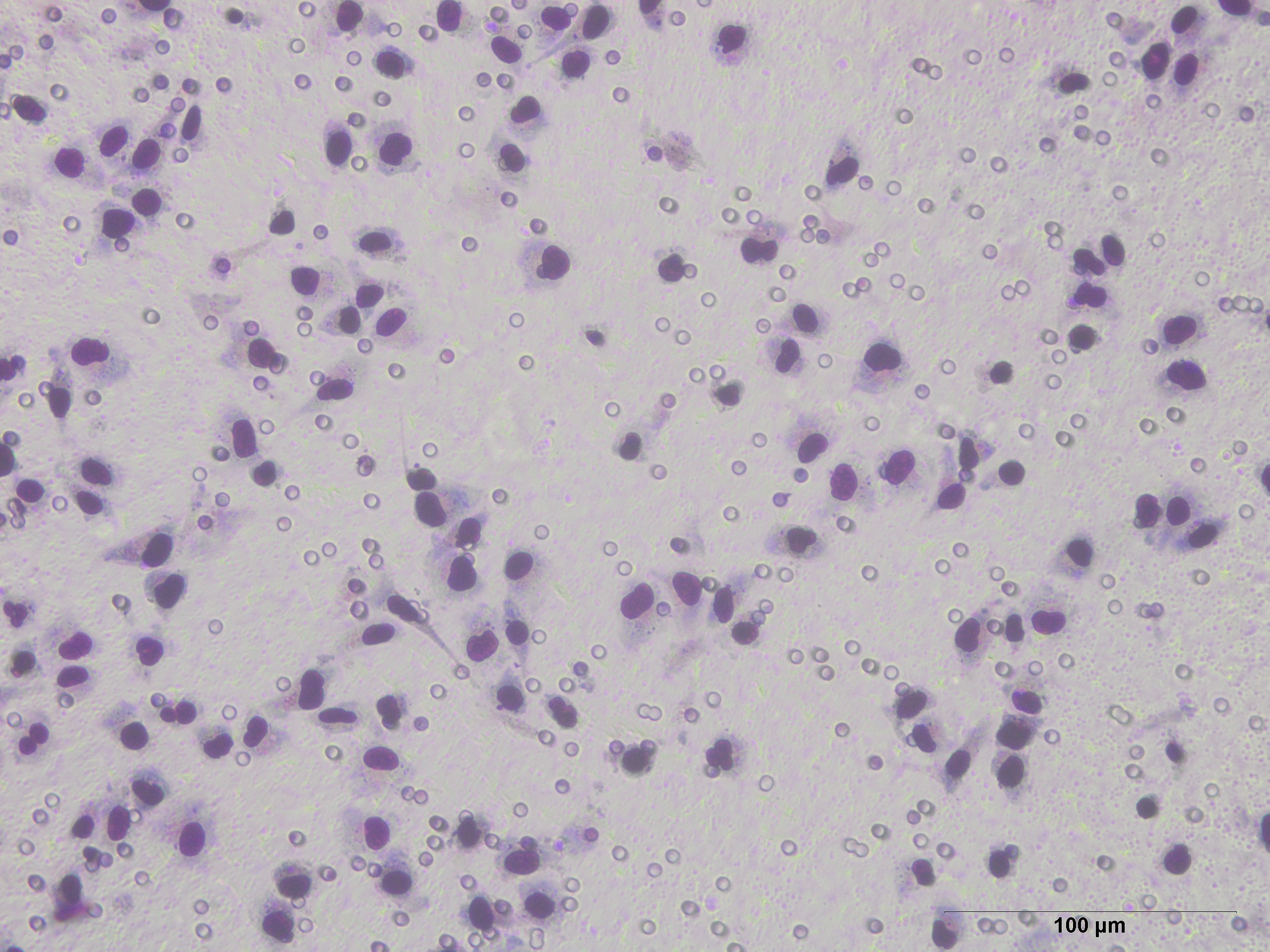

Supplement: Supplementary file 5 [file DataSheet_2.zip › Data Sheet 2/Fig2E/2-AC009948.5-NC-A549-INVASION.jpg]

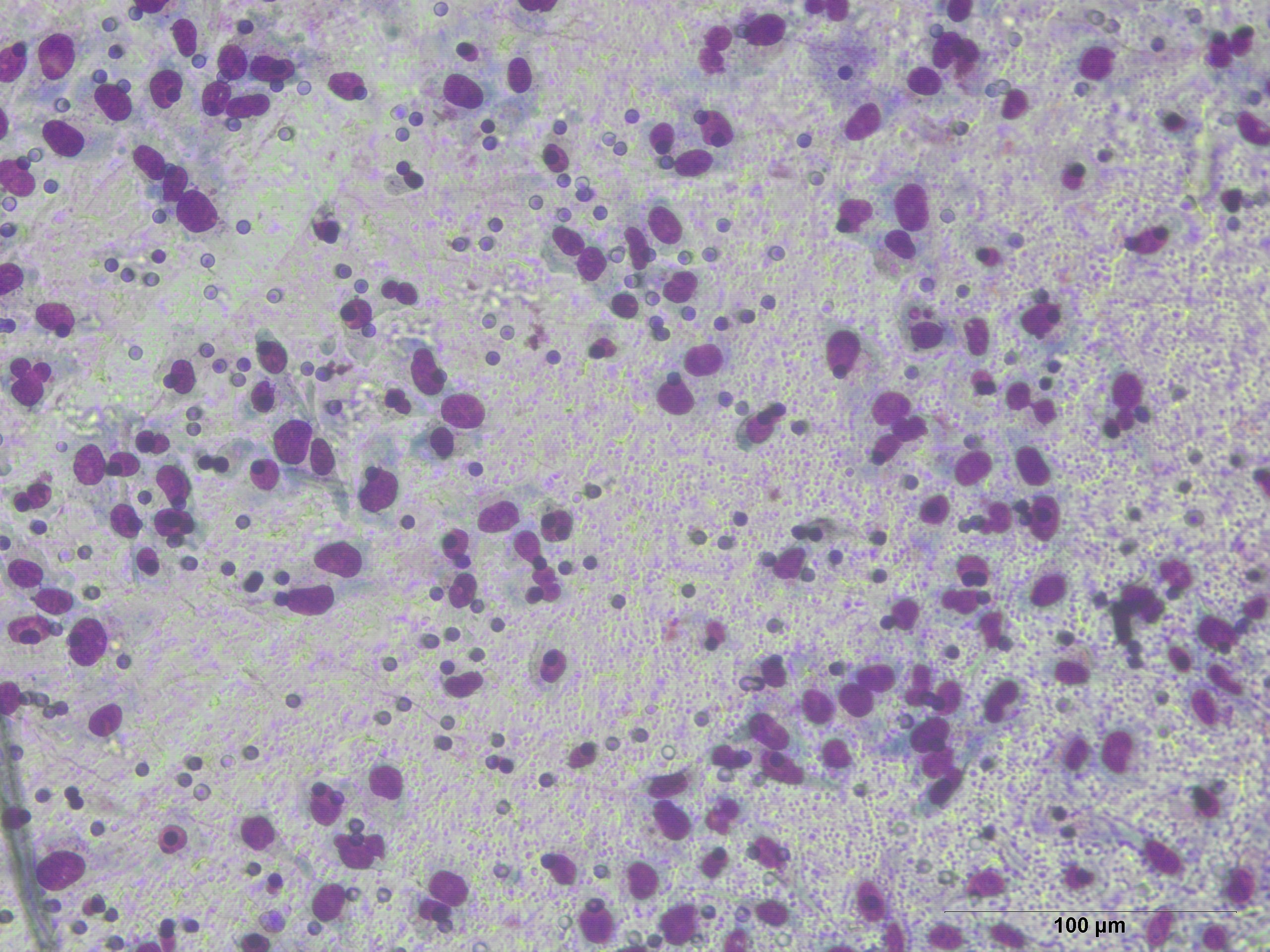

Supplement: Supplementary file 5 [file DataSheet_2.zip › Data Sheet 2/Fig2E/2-AC009948.5-NC-A549-M.jpg]

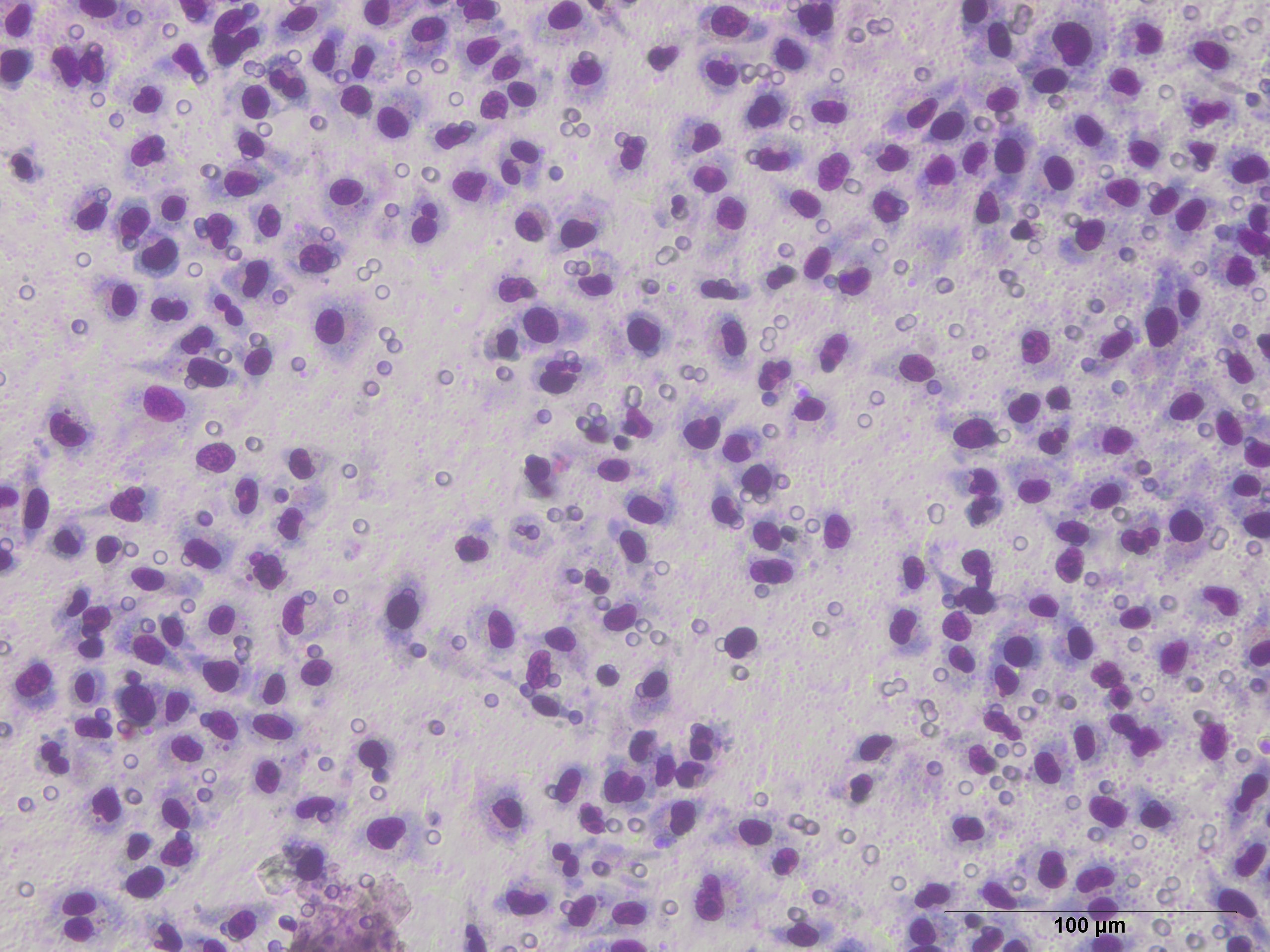

Supplement: Supplementary file 5 [file DataSheet_2.zip › Data Sheet 2/Fig2E/2-AC009948.5-over-A549-INVASION.jpg]

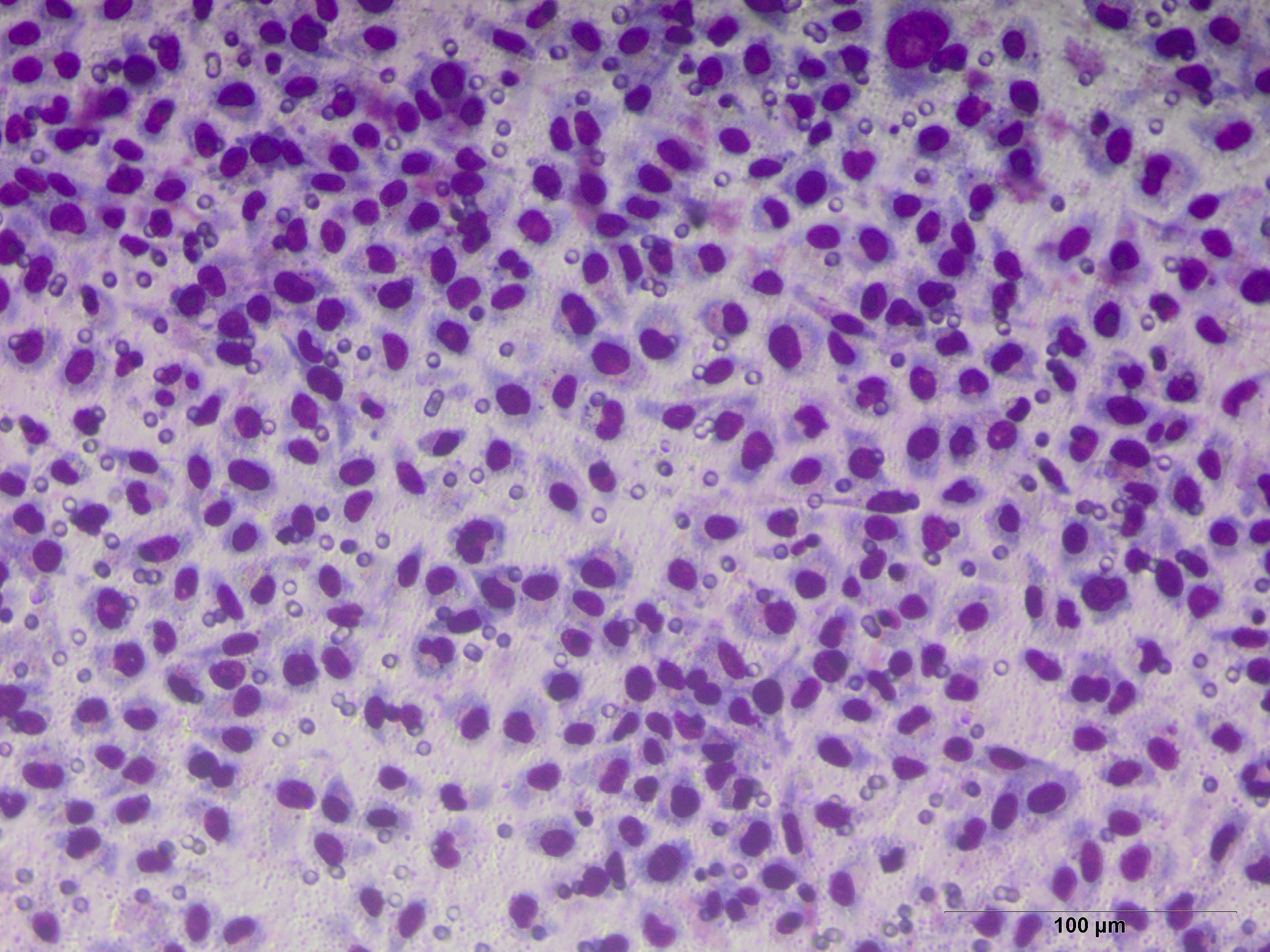

Supplement: Supplementary file 5 [file DataSheet_2.zip › Data Sheet 2/Fig2E/2-AC009948.5-over-A549-M.jpg]

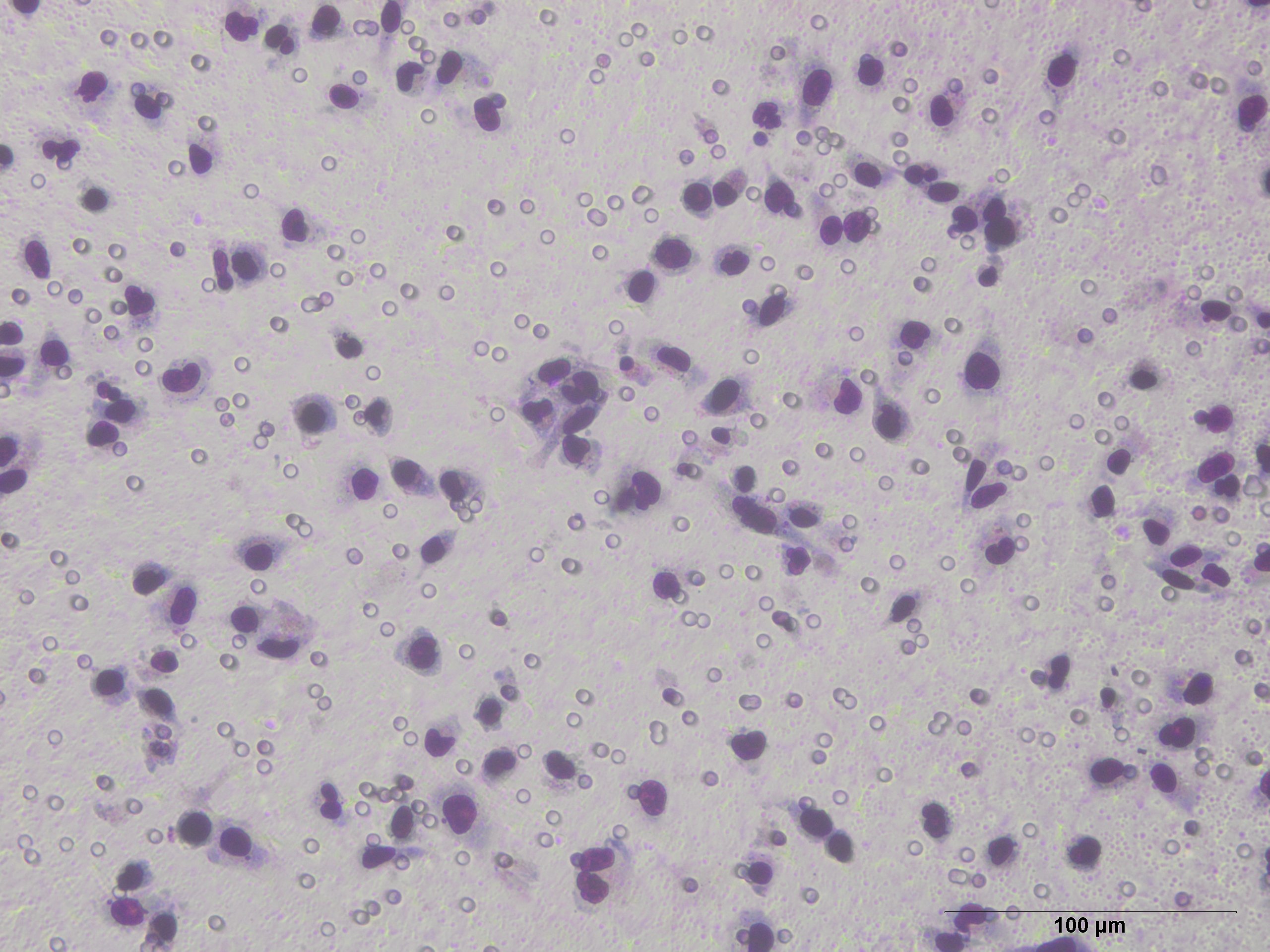

Supplement: Supplementary file 5 [file DataSheet_2.zip › Data Sheet 2/Fig2E/2-AC009948.5-Scrambled-A549-INVASION.jpg]
