# Supplementary material for: LncRNA-AC009948.5 promotes invasion and metastasis of lung adenocarcinoma by binding to miR-186-5p
Source: Front Oncol. 2022 Aug 19;12:949951. doi: 10.3389/fonc.2022.949951 (PMC9437580; doi:10.3389/fonc.2022.949951)
Supplement: Supplementary file 4 [file DataSheet_1.zip › Data Sheet 1/Fig2B/AC009948.5-1/Scrambled-Specimen_001_1_05052022090557.pdf]

BD FACSDiva 8.0.1

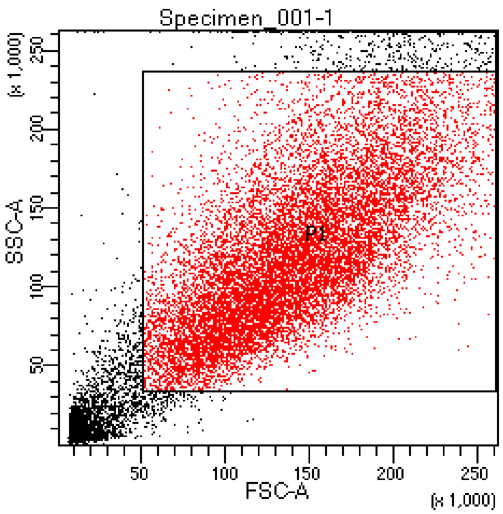

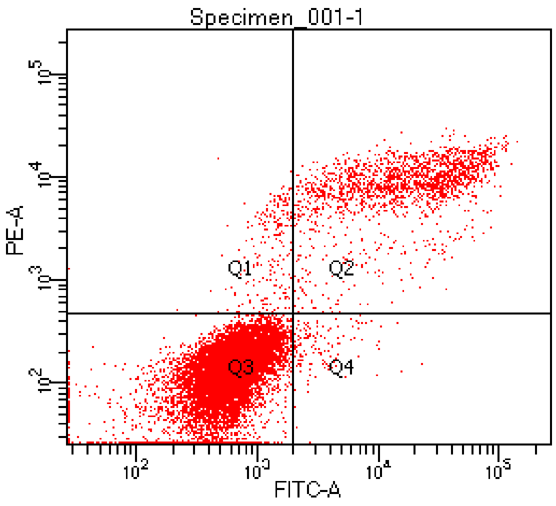

Experiment Name:	20220504-CL
Specimen Name:	Specimen_001
Tube Name:	1
Record Date:	May 4, 2022 2:30:14 PM
$OP:	Administrator
GUID:	315b10bc-0ec1-4d5d-9031-028...

Population	#Events	%Parent	FITC-A Mean	PE-A Mean
P1	14,089	70.4	3,928	1,393
Q1	####	2.2	1,352	2,068
Q2	####	17.7	21,456	7,538
Q3	####	75.2	627	140
Q4	####	4.9	3,911	294
