# Supplementary material for: LncRNA-AC009948.5 promotes invasion and metastasis of lung adenocarcinoma by binding to miR-186-5p
Source: Front Oncol. 2022 Aug 19;12:949951. doi: 10.3389/fonc.2022.949951 (PMC9437580; doi:10.3389/fonc.2022.949951)
Supplement: Supplementary file 4 [file DataSheet_1.zip › Data Sheet 1/Fig2B/AC009948.5-1/SiAC009948.5-Specimen_001_2_05052022090601.pdf]

BD FACSDiva 8.0.1

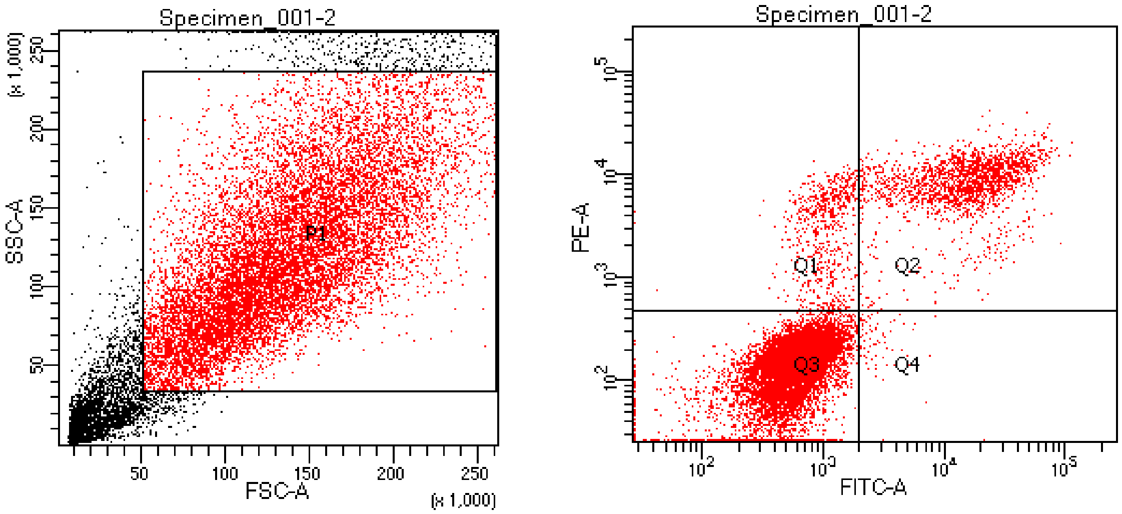

Experiment Name:	20220504-CL
Specimen Name:	Specimen_001
Tube Name:	2
Record Date:	May 4, 2022 2:30:59 PM
$OP:	Administrator
GUID:	d33defa9-0b61-4a69-a623-833...

Population	#Events	%Parent	FITC-A Mean	PE-A Mean
P1	13,251	66.3	3,608	1,746
Q1	####	5.6	1,163	3,453
Q2	####	21.4	17,571	7,763
Q3	####	68.7	595	136
Q4	####	4.3	3,928	293
