# Supplementary material for: LncRNA-AC009948.5 promotes invasion and metastasis of lung adenocarcinoma by binding to miR-186-5p
Source: Front Oncol. 2022 Aug 19;12:949951. doi: 10.3389/fonc.2022.949951 (PMC9437580; doi:10.3389/fonc.2022.949951)
Supplement: Supplementary file 4 [file DataSheet_1.zip › Data Sheet 1/Fig2B/AC009948.5-1/Specimen_001_FITC_05052022090549.pdf]

BD FACSDiva 8.0.1

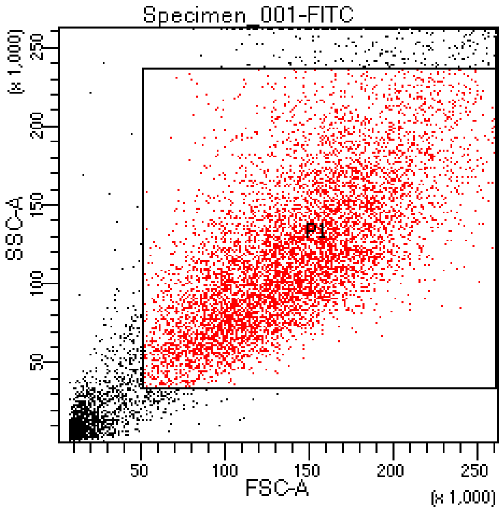

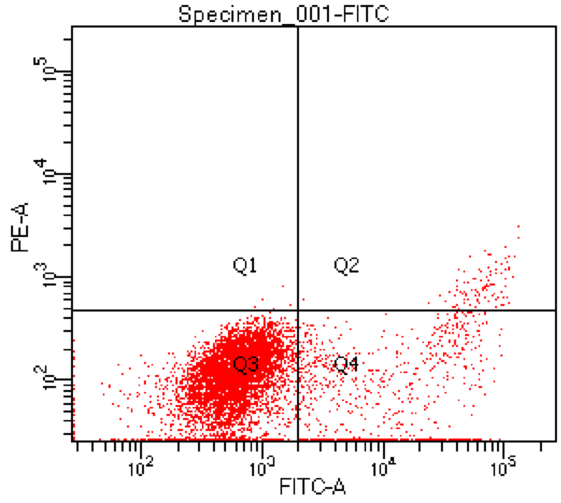

Experiment Name:	20220504-CL
Specimen Name:	Specimen_001
Tube Name:	FITC
Record Date:	May 4, 2022 2:28:41 PM
$OP:	Administrator
GUID:	6dd0e74f-0c02-4462-8fe7-1983...

Population	#Events	%Parent	FITC-A Mean	PE-A Mean
P1	7,162	71.6	3,502	131
Q1	####	0.4	1,471	548
Q2	####	3.4	42,385	944
Q3	####	78.7	641	120
Q4	####	17.5	13,353	104
