# Supplementary material for: LncRNA-AC009948.5 promotes invasion and metastasis of lung adenocarcinoma by binding to miR-186-5p
Source: Front Oncol. 2022 Aug 19;12:949951. doi: 10.3389/fonc.2022.949951 (PMC9437580; doi:10.3389/fonc.2022.949951)
Supplement: Supplementary file 4 [file DataSheet_1.zip › Data Sheet 1/Fig2B/AC009948.5-1/Specimen_001_NC_05052022090535.pdf]

BD FACSDiva 8.0.1

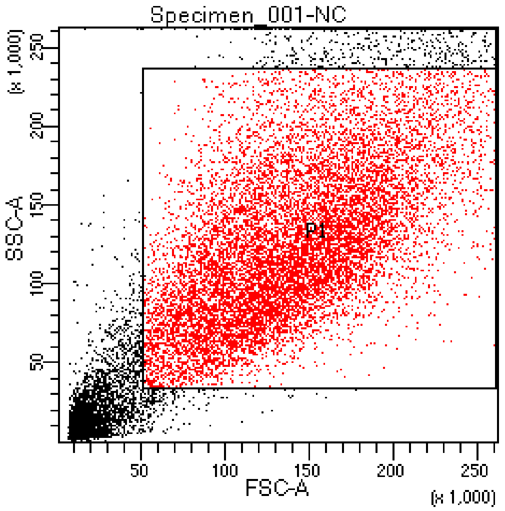

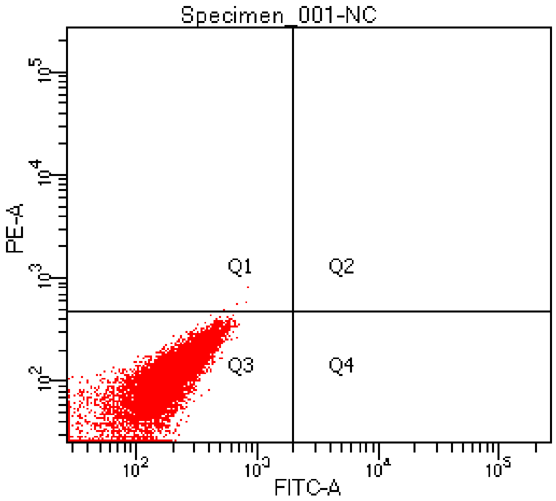

Experiment Name:	20220504-CL
Specimen Name:	Specimen_001
Tube Name:	NC
Record Date:	May 4, 2022 2:28:03 PM
$OP:	Administrator
GUID:	96a767cb-0ff0-43fa-89e2-a0c0...

Population	#Events	%Parent	FITC-A Mean	PE-A Mean
P1	12,528	62.6	193	120
Q1	####	4.2	1,046	681
Q2	####	0.4	2,586	1,612
Q3	####	95.4	190	118
Q4	####	0.0	####	####
