# Supplementary material for: LncRNA-AC009948.5 promotes invasion and metastasis of lung adenocarcinoma by binding to miR-186-5p
Source: Front Oncol. 2022 Aug 19;12:949951. doi: 10.3389/fonc.2022.949951 (PMC9437580; doi:10.3389/fonc.2022.949951)
Supplement: Supplementary file 4 [file DataSheet_1.zip › Data Sheet 1/Fig2B/AC009948.5-1/Specimen_001_PI_05052022090553.pdf]

BD FACSDiva 8.0.1

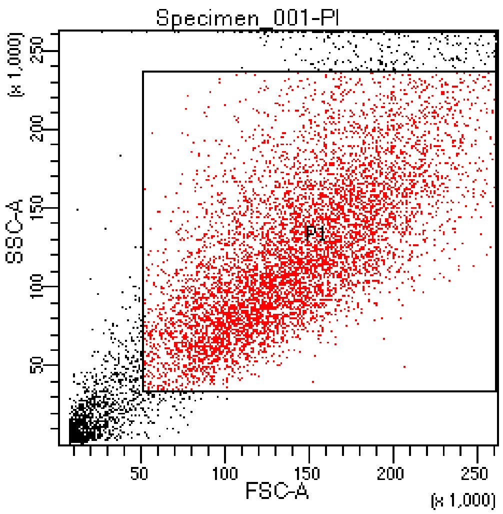

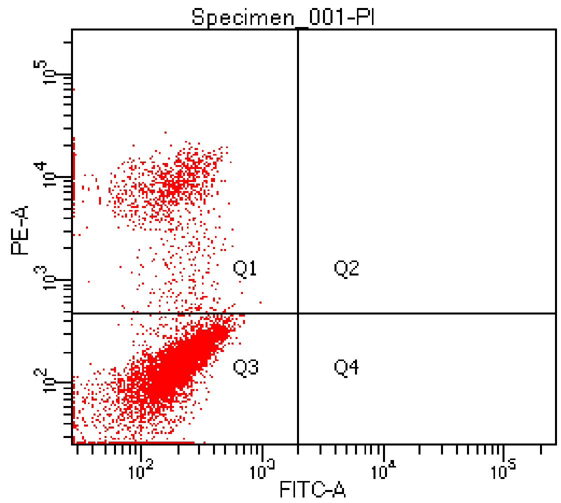

Experiment Name:	20220504-CL
Specimen Name:	Specimen_001
Tube Name:	PI
Record Date:	May 4, 2022 2:29:19 PM
$OP:	Administrator
GUID:	f954d850-f16c-45f6-9915-b252...

Population	#Events	%Parent	FITC-A Mean	PE-A Mean
P1	7,108	71.1	196	1,467
Q1	####	22.6	318	6,359
Q2	####	0.1	35,712	28,253
Q3	####	77.3	194	154
Q4	####	0.0	####	####
