# Supplementary material for: LncRNA-AC009948.5 promotes invasion and metastasis of lung adenocarcinoma by binding to miR-186-5p
Source: Front Oncol. 2022 Aug 19;12:949951. doi: 10.3389/fonc.2022.949951 (PMC9437580; doi:10.3389/fonc.2022.949951)
Supplement: Supplementary file 4 [file DataSheet_1.zip › Data Sheet 1/Fig2B/AC009948.5-2/Scrambled-Specimen_001_1_05052022090341.pdf]

BD FACSDiva 8.0.1

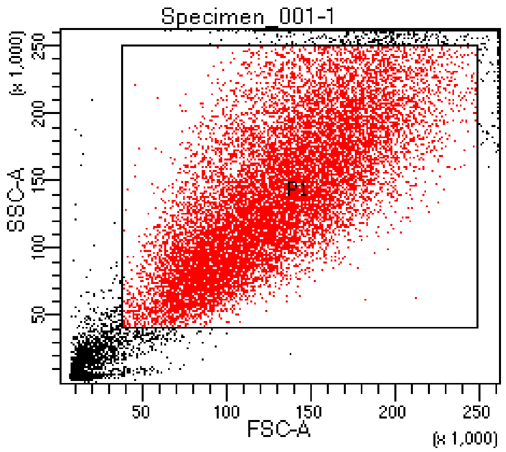

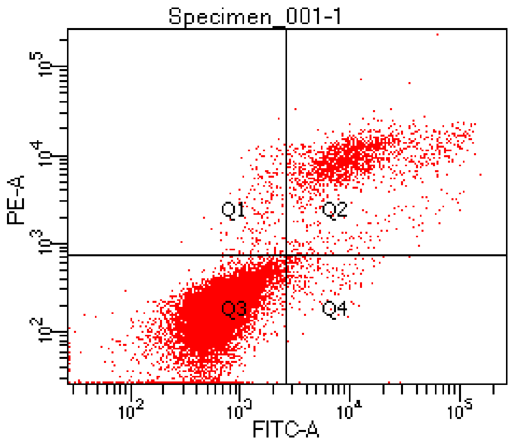

Experiment Name:	20220504-LL
Specimen Name:	Specimen_001
Tube Name:	1
Record Date:	May 4, 2022 2:36:42 PM
$OP:	Administrator
GUID:	ddc5d60e-ae4e-489d-8948-0bd...

Population	#Events	%Parent	FITC-A Mean	PE-A Mean
P1	14,718	73.6	2,724	1,238
Q1	####	1.6	1,658	3,046
Q2	####	11.9	17,535	9,194
Q3	####	82.9	725	193
Q4	####	3.5	5,521	404
