# Supplementary material for: LncRNA-AC009948.5 promotes invasion and metastasis of lung adenocarcinoma by binding to miR-186-5p
Source: Front Oncol. 2022 Aug 19;12:949951. doi: 10.3389/fonc.2022.949951 (PMC9437580; doi:10.3389/fonc.2022.949951)
Supplement: Supplementary file 4 [file DataSheet_1.zip › Data Sheet 1/Fig2B/AC009948.5-2/SiAC009948.5-pecimen_001_2_05052022090346.pdf]

BD FACSDiva 8.0.1

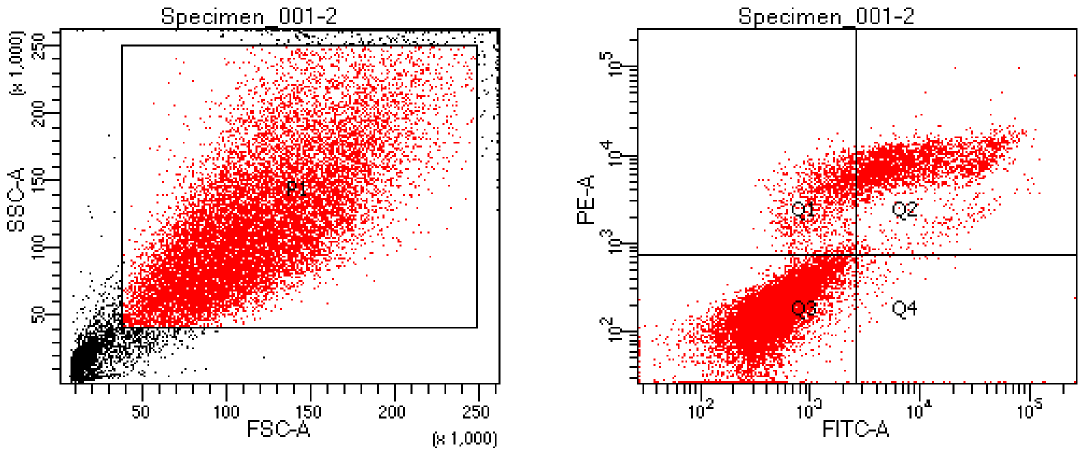

Experiment Name:	20220504-LL
Specimen Name:	Specimen_001
Tube Name:	2
Record Date:	May 4, 2022 2:37:26 PM
$OP:	Administrator
GUID:	a19bbe36-6894-45ae-b1a3-45...

Population	#Events	%Parent	FITC-A Mean	PE-A Mean
P1	15,514	77.6	3,328	1,931
Q1	####	7.6	1,483	3,924
Q2	####	17.7	14,317	7,781
Q3	####	70.7	570	195
Q4	####	4.0	8,916	107
