# Supplementary material for: LncRNA-AC009948.5 promotes invasion and metastasis of lung adenocarcinoma by binding to miR-186-5p
Source: Front Oncol. 2022 Aug 19;12:949951. doi: 10.3389/fonc.2022.949951 (PMC9437580; doi:10.3389/fonc.2022.949951)
Supplement: Supplementary file 4 [file DataSheet_1.zip › Data Sheet 1/Fig2B/AC009948.5-2/Specimen_001_FITC_05052022090337.pdf]

BD FACSDiva 8.0.1

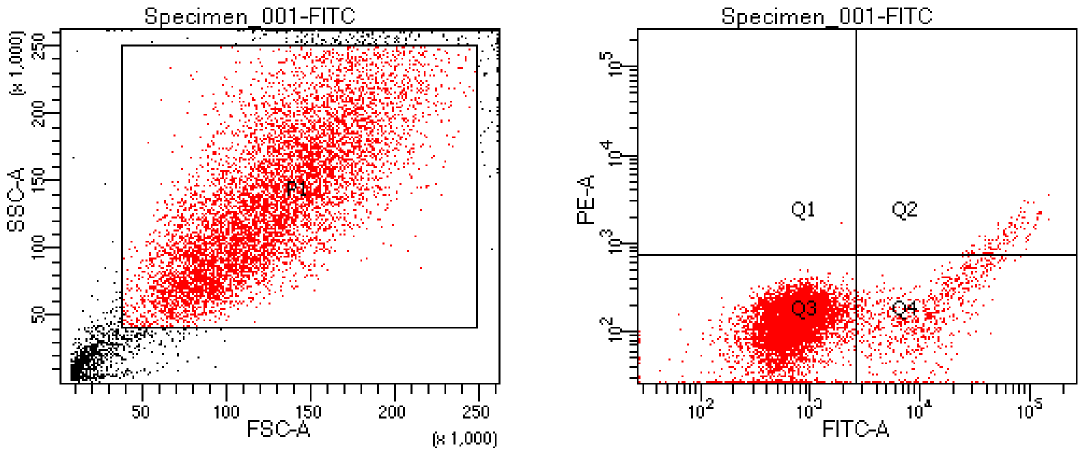

Experiment Name:	20220504-LL
Specimen Name:	Specimen_001
Tube Name:	FITC
Record Date:	May 4, 2022 2:35:49 PM
$OP:	Administrator
GUID:	fe9d97aa-4346-470d-98d0-b14...

Population	#Events	%Parent	FITC-A Mean	PE-A Mean
P1	7,539	75.4	3,007	158
Q1	####	0.1	1,597	1,064
Q2	####	1.5	67,025	1,525
Q3	####	85.0	770	133
Q4	####	13.5	12,030	206
