# Supplementary material for: LncRNA-AC009948.5 promotes invasion and metastasis of lung adenocarcinoma by binding to miR-186-5p
Source: Front Oncol. 2022 Aug 19;12:949951. doi: 10.3389/fonc.2022.949951 (PMC9437580; doi:10.3389/fonc.2022.949951)
Supplement: Supplementary file 4 [file DataSheet_1.zip › Data Sheet 1/Fig2B/AC009948.5-2/Specimen_001_NC_05052022090311.pdf]

BD FACSDiva 8.0.1

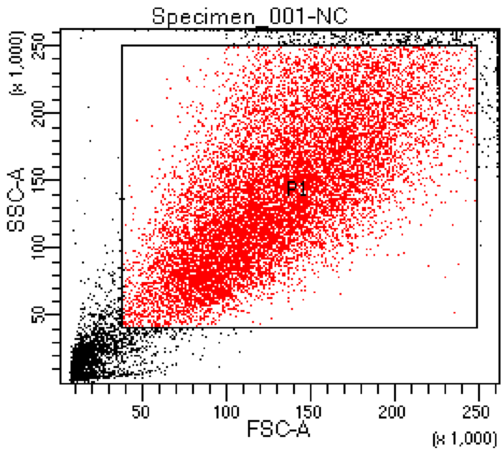

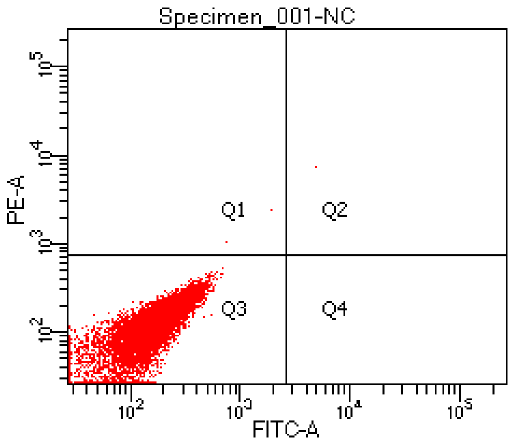

Experiment Name:	20220504-LL
Specimen Name:	Specimen_001
Tube Name:	NC
Record Date:	May 4, 2022 2:34:20 PM
$OP:	Administrator
GUID:	cfc2907c-0788-42f7-b2ce-66c4...

Population	#Events	%Parent	FITC-A Mean	PE-A Mean
P1	13,586	67.9	177	133
Q1	####	0.6	1,338	1,064
Q2	####	0.0	3,440	3,628
Q3	####	99.4	183	137
Q4	####	0.0	####	####
