# Supplementary material for: LncRNA-AC009948.5 promotes invasion and metastasis of lung adenocarcinoma by binding to miR-186-5p
Source: Front Oncol. 2022 Aug 19;12:949951. doi: 10.3389/fonc.2022.949951 (PMC9437580; doi:10.3389/fonc.2022.949951)
Supplement: Supplementary file 4 [file DataSheet_1.zip › Data Sheet 1/Fig2B/AC009948.5-2/Specimen_001_PI_05052022090334.pdf]

BD FACSDiva 8.0.1

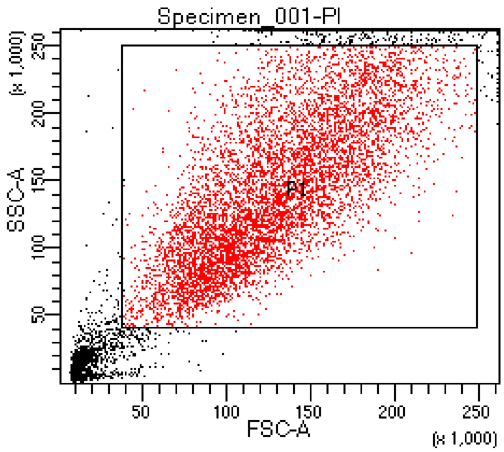

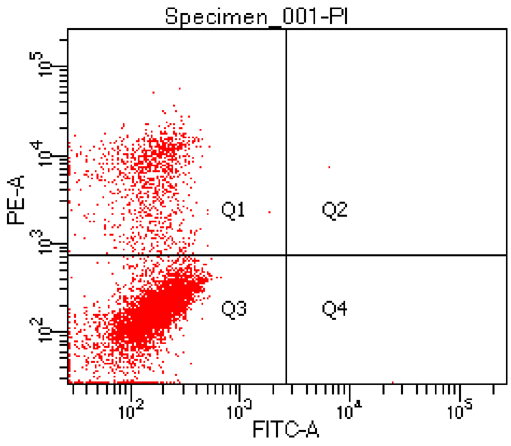

Experiment Name:	20220504-LL
Specimen Name:	Specimen_001
Tube Name:	PI
Record Date:	May 4, 2022 2:35:07 PM
$OP:	Administrator
GUID:	cc2e5467-a5f3-467b-88e8-813...

Population	#Events	%Parent	FITC-A Mean	PE-A Mean
P1	7,027	70.3	179	1,312
Q1	####	16.7	236	7,093
Q2	####	0.0	14,516	104,735
Q3	####	83.3	175	205
Q4	####	0.0	24,165	-2,829
