# Supplementary material for: LncRNA-AC009948.5 promotes invasion and metastasis of lung adenocarcinoma by binding to miR-186-5p
Source: Front Oncol. 2022 Aug 19;12:949951. doi: 10.3389/fonc.2022.949951 (PMC9437580; doi:10.3389/fonc.2022.949951)
Supplement: Supplementary file 4 [file DataSheet_1.zip › Data Sheet 1/Fig2B/AC009948.5-3/Scrambled-Specimen_001_2_06052022161637.pdf]

BD FACSDiva 8.0.1

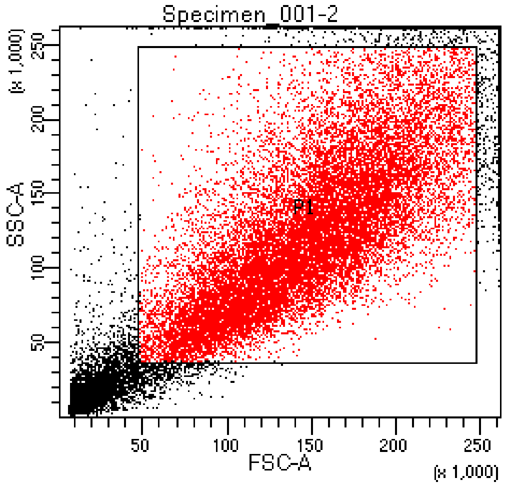

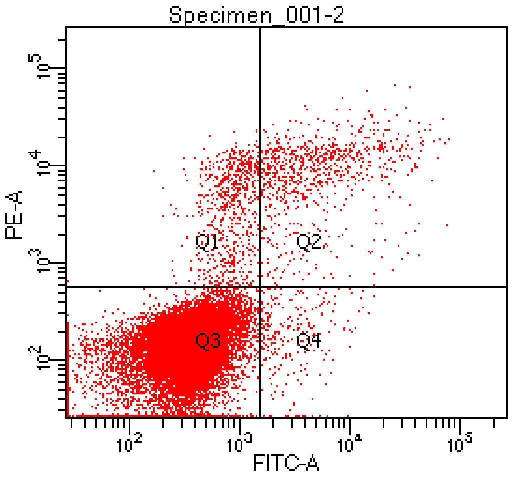

Experiment Name:	20220506-CL
Specimen Name:	Specimen_001
Tube Name:	2
Record Date:	May 6, 2022 3:08:41 PM
$OP:	Administrator
GUID:	9286d33a-6897-45f4-81a2-d00...

Population	#Events	%Parent	FITC-A Mean	PE-A Mean
All Events	30,000	####	1,054	1,113
Q1	1,508	5.0	856	4,941
Q2	2,313	7.7	6,975	9,532
Q3	25,007	83.4	382	144
Q4	1,172	3.9	3,941	252
P1	20,214	67.4	781	788
