# Supplementary material for: LncRNA-AC009948.5 promotes invasion and metastasis of lung adenocarcinoma by binding to miR-186-5p
Source: Front Oncol. 2022 Aug 19;12:949951. doi: 10.3389/fonc.2022.949951 (PMC9437580; doi:10.3389/fonc.2022.949951)
Supplement: Supplementary file 4 [file DataSheet_1.zip › Data Sheet 1/Fig2B/AC009948.5-3/SiAC009948.5-Specimen_001_1_06052022161633.pdf]

BD FACSDiva 8.0.1

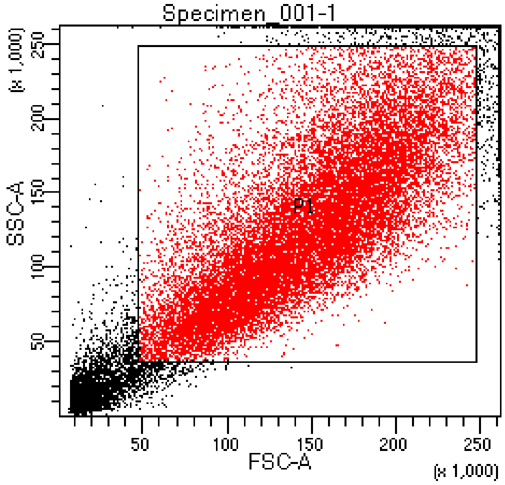

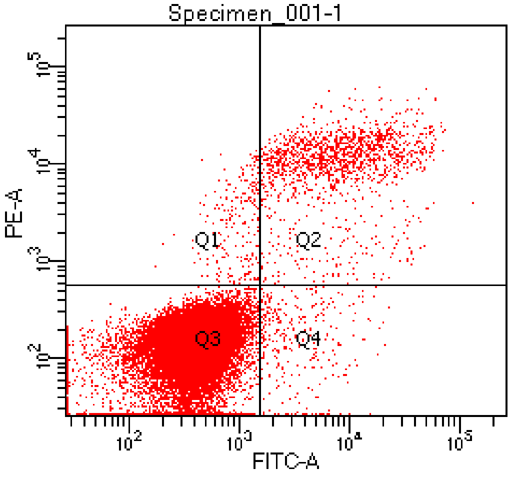

Experiment Name:	20220506-CL
Specimen Name:	Specimen_001
Tube Name:	1
Record Date:	May 6, 2022 3:07:57 PM
$OP:	Administrator
GUID:	5541b5e7-6230-40f3-aa2c-42c...

Population	#Events	%Parent	FITC-A Mean	PE-A Mean
All Events	30,000	####	1,805	1,257
Q1	565	1.9	1,009	3,265
Q2	3,264	10.9	10,584	9,771
Q3	24,419	81.4	447	143
Q4	1,752	5.8	4,638	274
P1	20,965	69.9	1,352	1,044
