# Supplementary material for: LncRNA-AC009948.5 promotes invasion and metastasis of lung adenocarcinoma by binding to miR-186-5p
Source: Front Oncol. 2022 Aug 19;12:949951. doi: 10.3389/fonc.2022.949951 (PMC9437580; doi:10.3389/fonc.2022.949951)
Supplement: Supplementary file 4 [file DataSheet_1.zip › Data Sheet 1/Fig2B/AC009948.5-3/Specimen_001_FITC_06052022161628.pdf]

BD FACSDiva 8.0.1

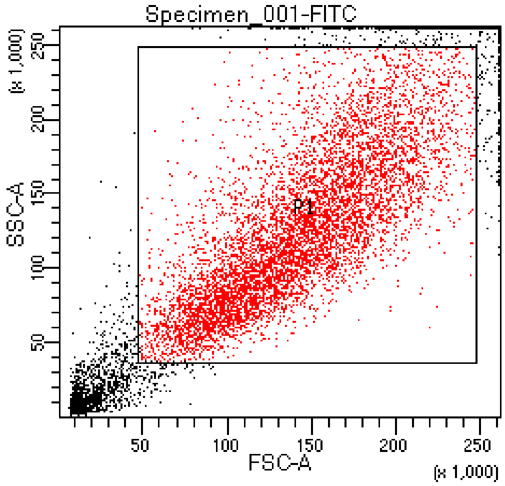

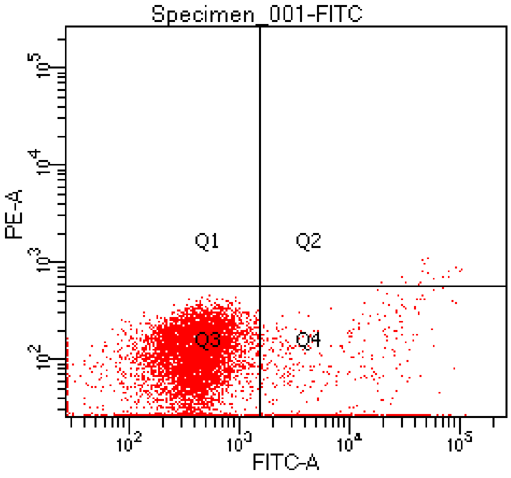

Experiment Name:	20220506-CL
Specimen Name:	Specimen_001
Tube Name:	FITC
Record Date:	May 6, 2022 3:06:59 PM
$OP:	Administrator
GUID:	758f3ef5-062e-4554-b71e-19e0...

Population	#Events	%Parent	FITC-A Mean	PE-A Mean
All Events	10,000	####	2,038	128
Q1	36	0.4	1,232	661
Q2	129	1.3	14,004	927
Q3	8,427	84.3	443	122
Q4	1,408	14.1	10,508	79
P1	7,233	72.3	1,584	115
