# Supplementary material for: LncRNA-AC009948.5 promotes invasion and metastasis of lung adenocarcinoma by binding to miR-186-5p
Source: Front Oncol. 2022 Aug 19;12:949951. doi: 10.3389/fonc.2022.949951 (PMC9437580; doi:10.3389/fonc.2022.949951)
Supplement: Supplementary file 4 [file DataSheet_1.zip › Data Sheet 1/Fig2B/AC009948.5-3/Specimen_001_NC_06052022161600.pdf]

BD FACSDiva 8.0.1

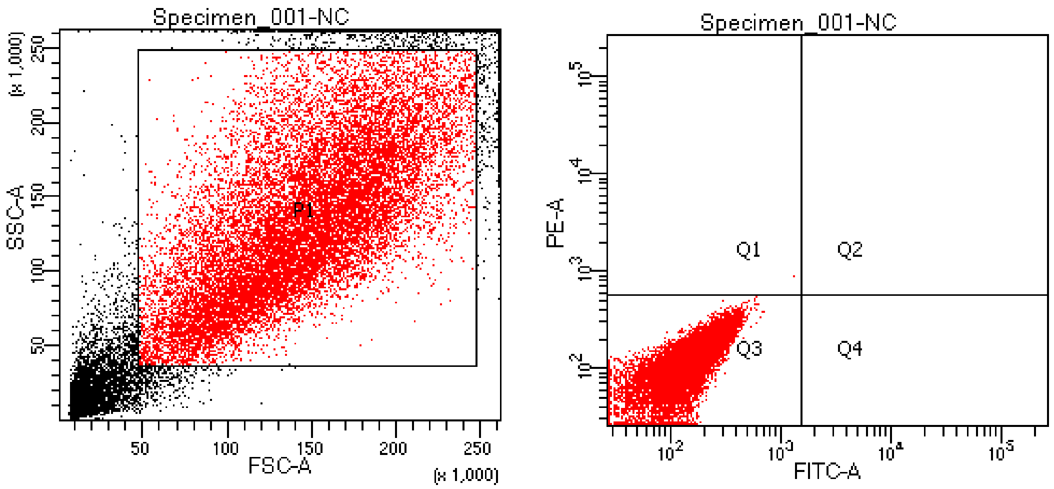

Experiment Name:	20220506-CL
Specimen Name:	Specimen_001
Tube Name:	NC
Record Date:	May 6, 2022 3:05:42 PM
$OP:	Administrator
GUID:	3ef1b28c-168e-41bc-a3a3-c7f8...

Population	#Events	%Parent	FITC-A Mean	PE-A Mean
All Events	30,000	####	173	146
Q1	683	2.3	862	760
Q2	34	0.1	2,597	2,591
Q3	29,283	97.6	154	128
Q4	0	0.0	####	####
P1	18,987	63.3	151	127
