# Supplementary material for: LncRNA-AC009948.5 promotes invasion and metastasis of lung adenocarcinoma by binding to miR-186-5p
Source: Front Oncol. 2022 Aug 19;12:949951. doi: 10.3389/fonc.2022.949951 (PMC9437580; doi:10.3389/fonc.2022.949951)
Supplement: Supplementary file 4 [file DataSheet_1.zip › Data Sheet 1/Fig2B/AC009948.5-3/Specimen_001_PI_06052022161624.pdf]

BD FACSDiva 8.0.1

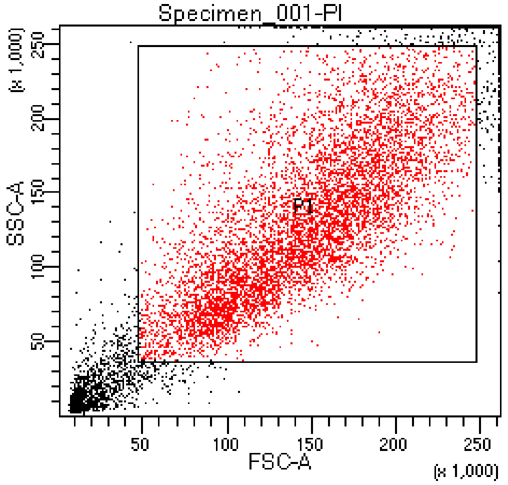

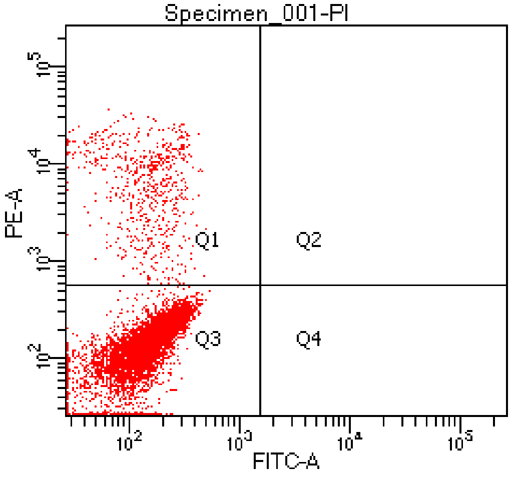

Experiment Name:	20220506-CL
Specimen Name:	Specimen_001
Tube Name:	PI
Record Date:	May 6, 2022 3:06:22 PM
$OP:	Administrator
GUID:	5ef9444b-58a7-4e4c-b0ef-5311...

Population	#Events	%Parent	FITC-A Mean	PE-A Mean
All Events	10,000	####	172	1,266
Q1	1,544	15.4	272	7,259
Q2	3	0.0	1,572	25,086
Q3	8,453	84.5	154	163
Q4	0	0.0	####	####
P1	6,912	69.1	151	974
