# Supplementary figures and images for: LncRNA-AC009948.5 promotes invasion and metastasis of lung adenocarcinoma by binding to miR-186-5p (part 2 of 4)
Source: Front Oncol. 2022 Aug 19;12:949951. doi: 10.3389/fonc.2022.949951 (PMC9437580; doi:10.3389/fonc.2022.949951)

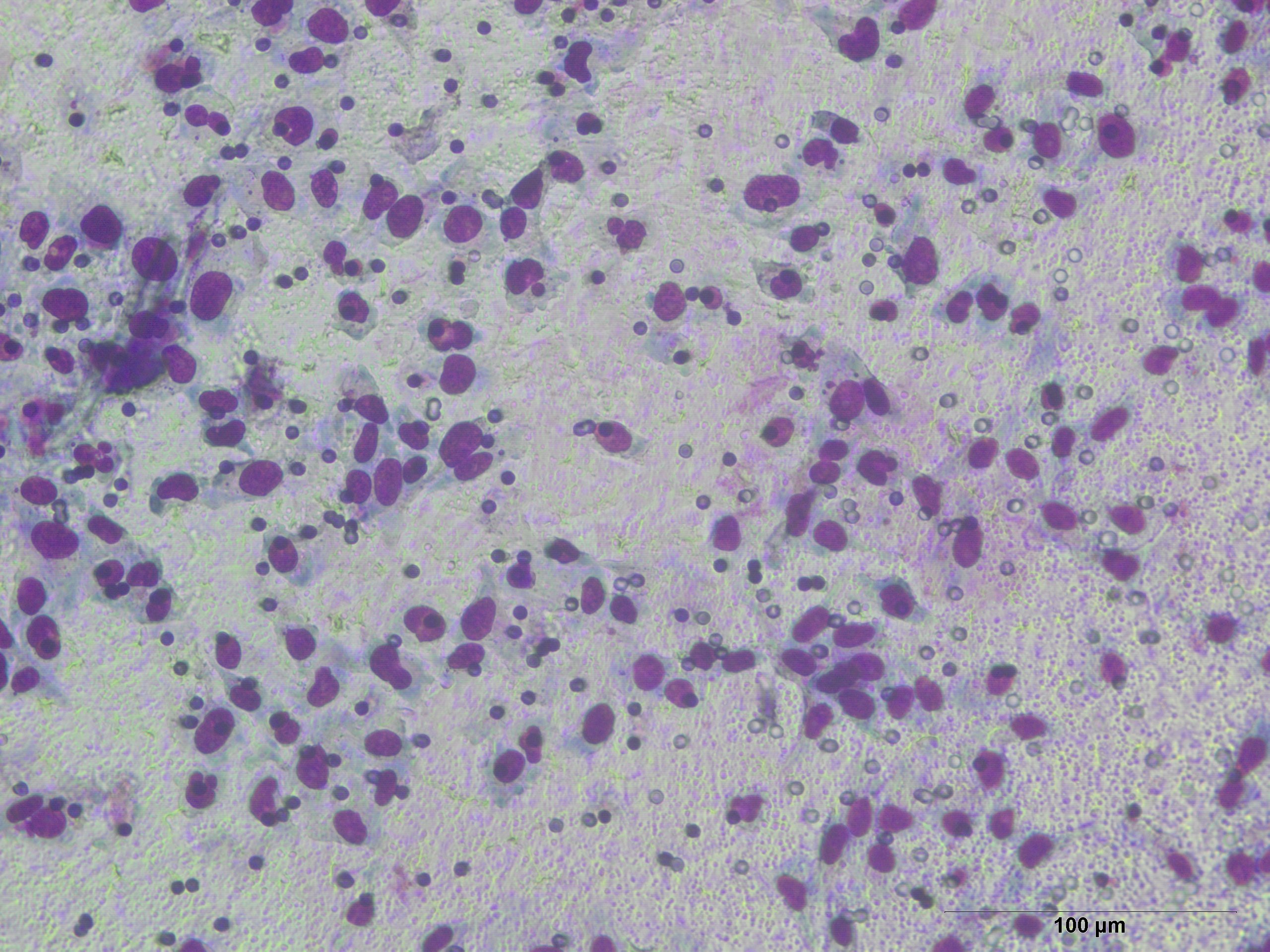

Supplement: Supplementary file 5 [file DataSheet_2.zip › Data Sheet 2/Fig2E/2-AC009948.5-Scrambled-A549-M.jpg]

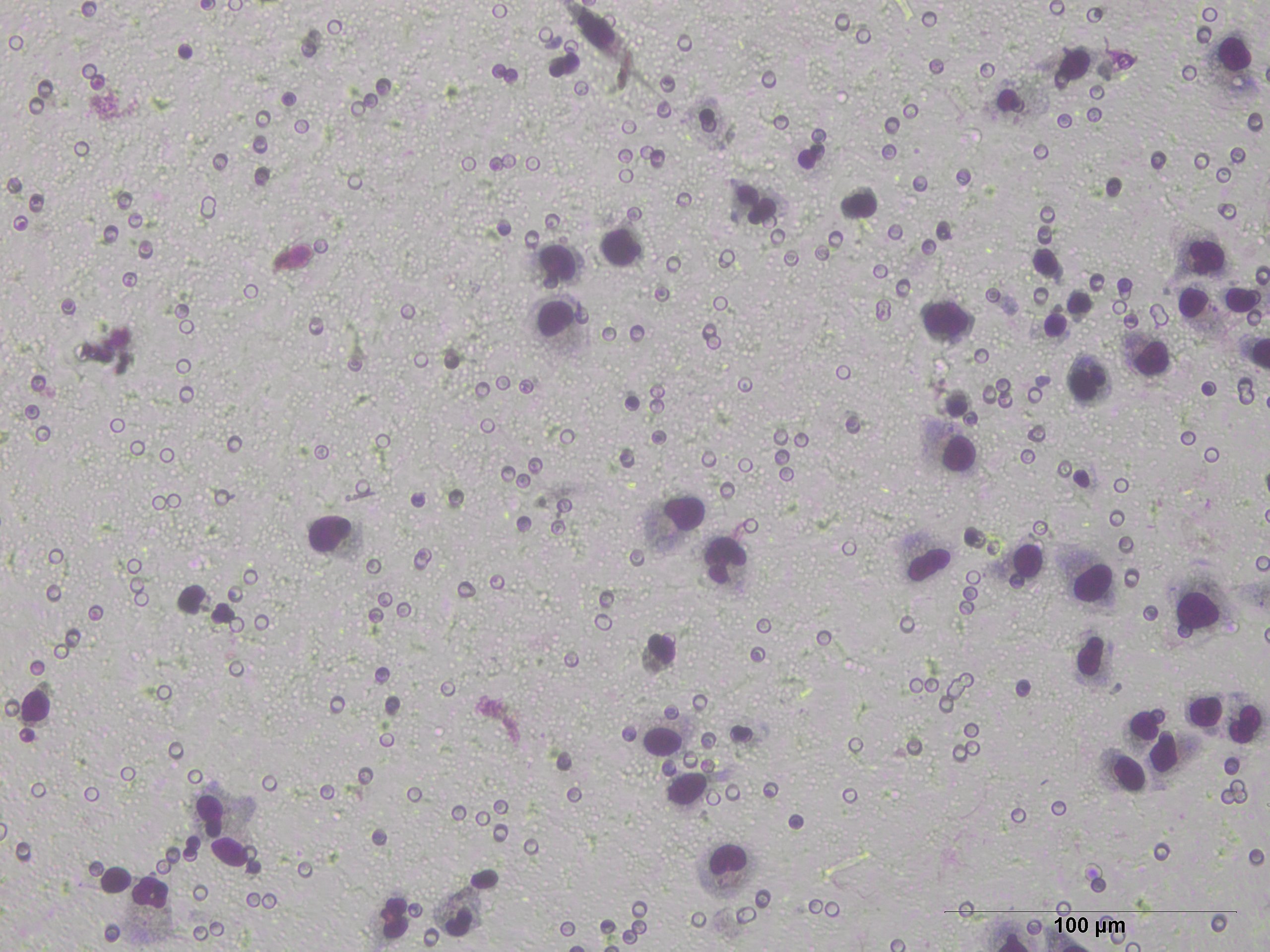

Supplement: Supplementary file 5 [file DataSheet_2.zip › Data Sheet 2/Fig2E/2-AC009948.5-Si-A549-INVASION.jpg]

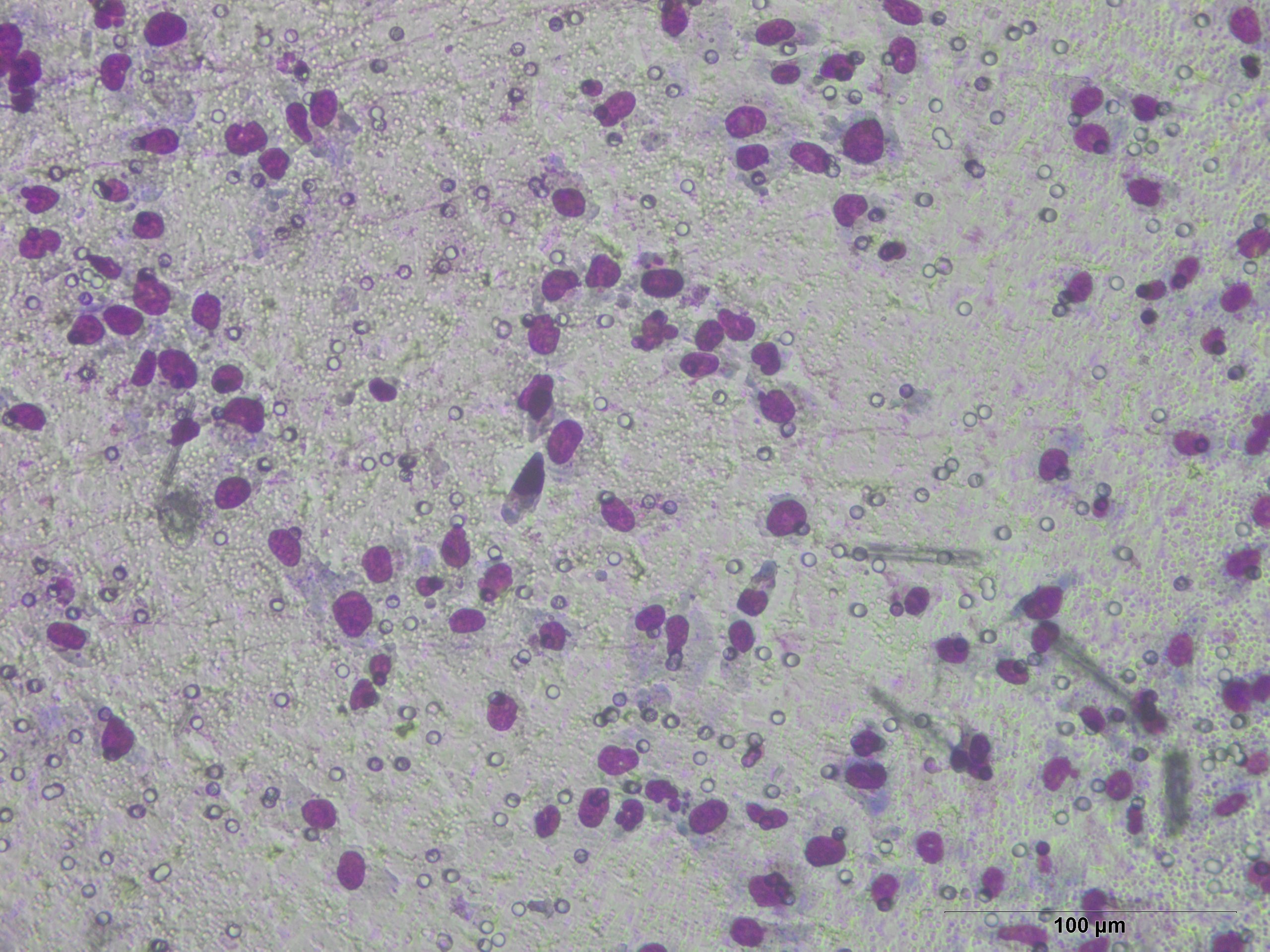

Supplement: Supplementary file 5 [file DataSheet_2.zip › Data Sheet 2/Fig2E/2-AC009948.5-Si-A549-M.jpg]

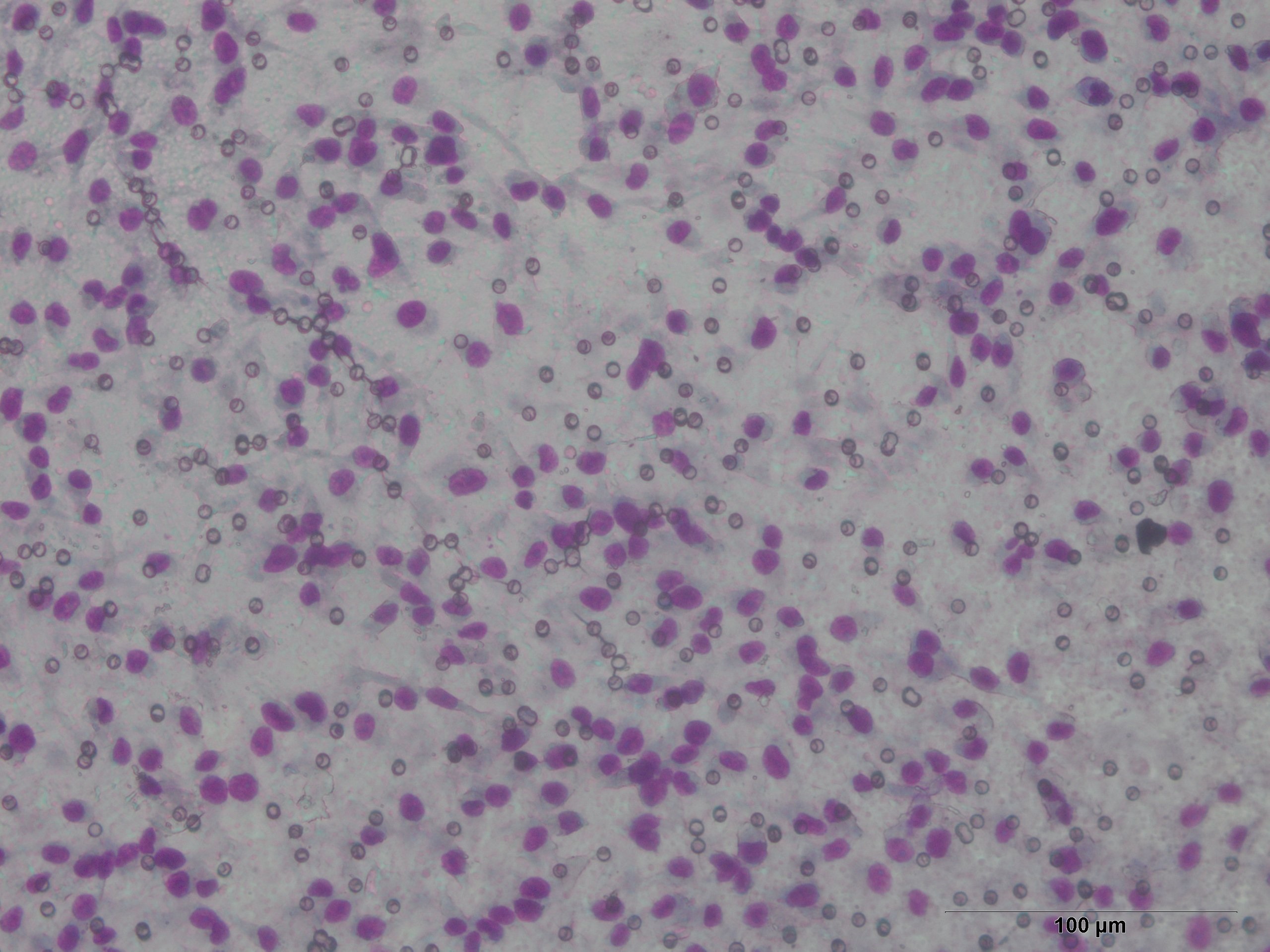

Supplement: Supplementary file 5 [file DataSheet_2.zip › Data Sheet 2/Fig2E/3-AC009948.5-NC-A549-M.jpg]

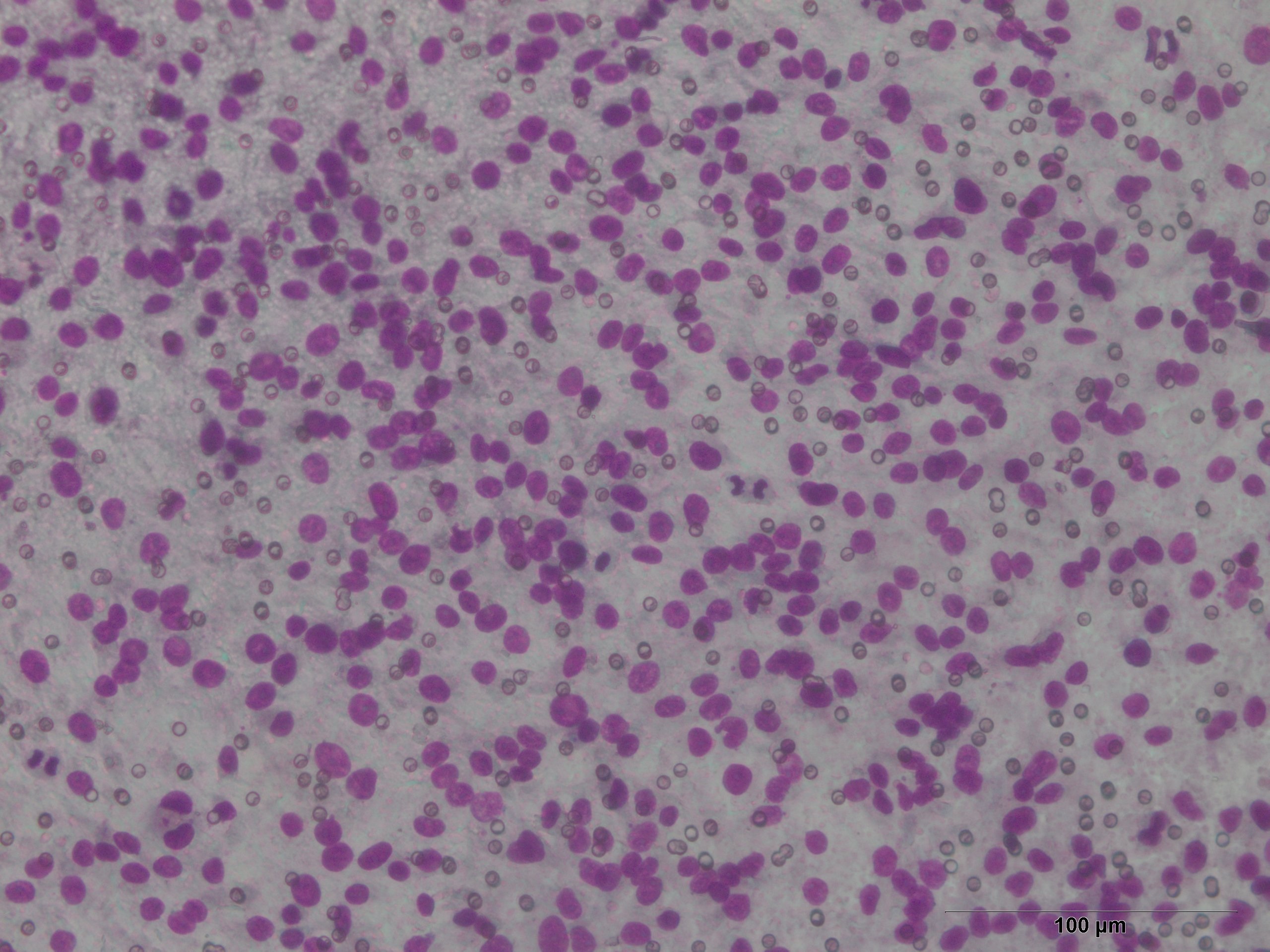

Supplement: Supplementary file 5 [file DataSheet_2.zip › Data Sheet 2/Fig2E/3-AC009948.5-over-A549-M.jpg]

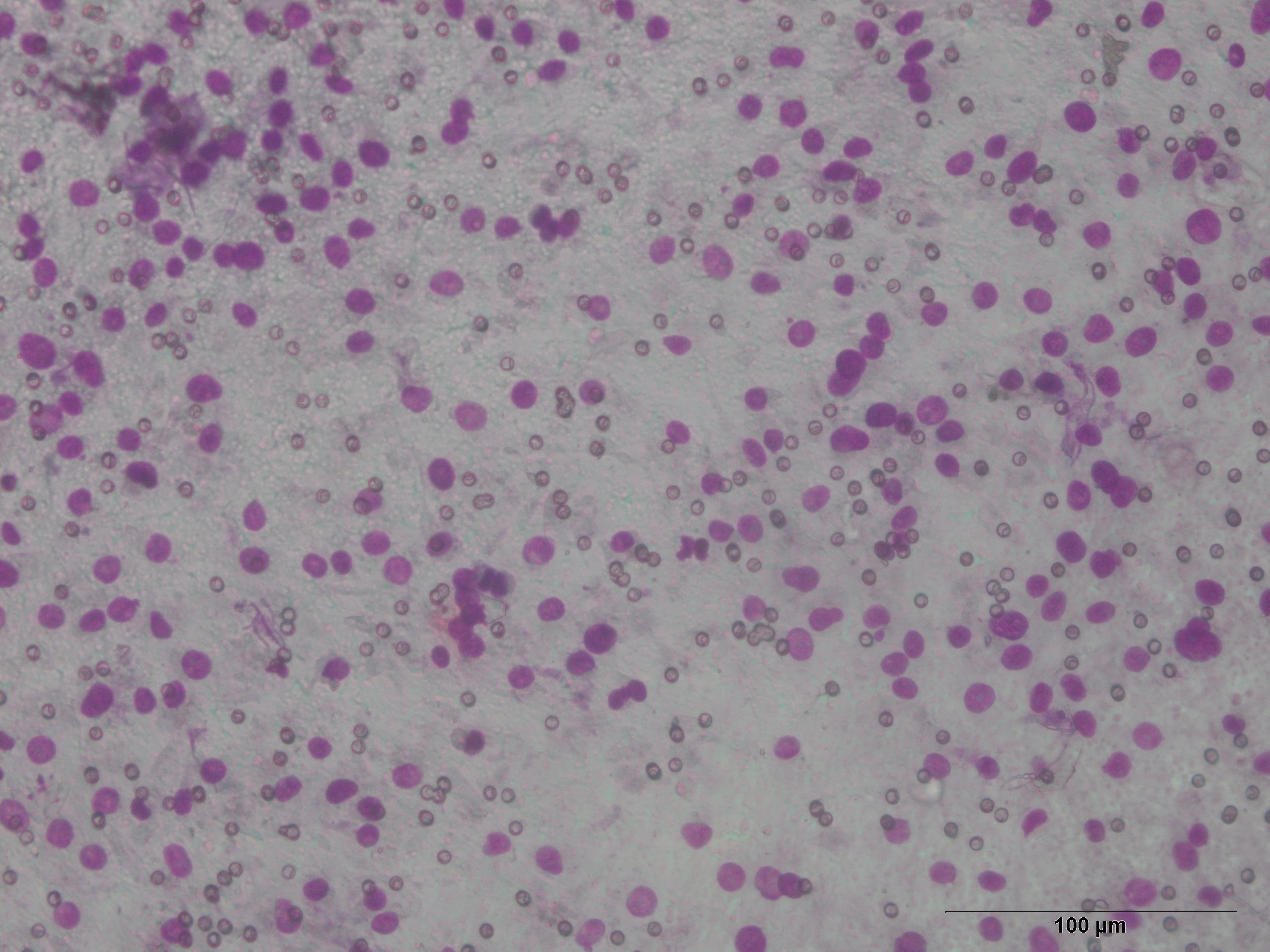

Supplement: Supplementary file 5 [file DataSheet_2.zip › Data Sheet 2/Fig2E/3-AC009948.5-Scrambled-A549-M.jpg]

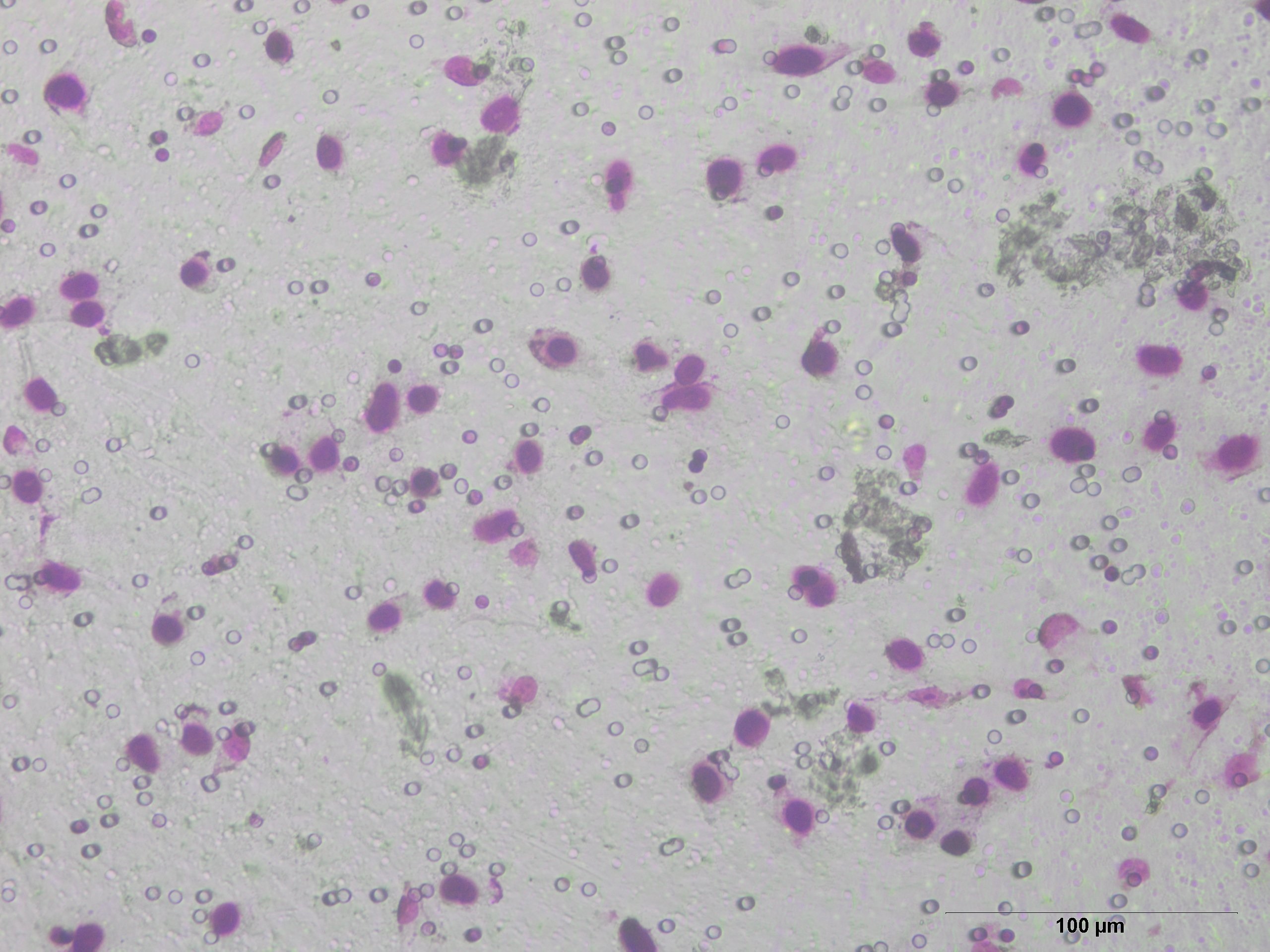

Supplement: Supplementary file 5 [file DataSheet_2.zip › Data Sheet 2/Fig2E/3-AC009948.5-Si-A549-M.jpg]

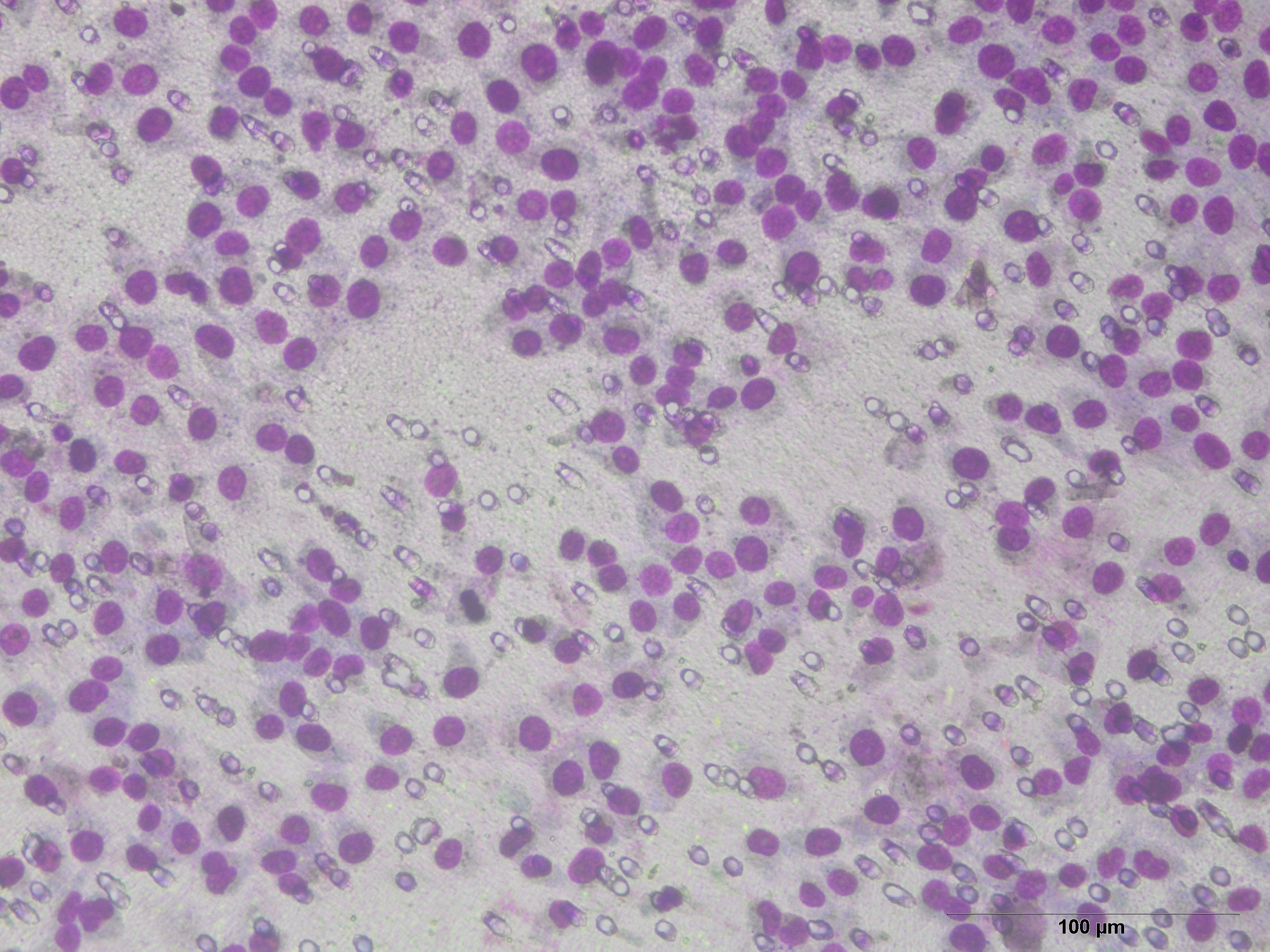

Supplement: Supplementary file 5 [file DataSheet_2.zip › Data Sheet 2/Fig2E/3-NC-AC009948.5-A549-INVASION.jpg]

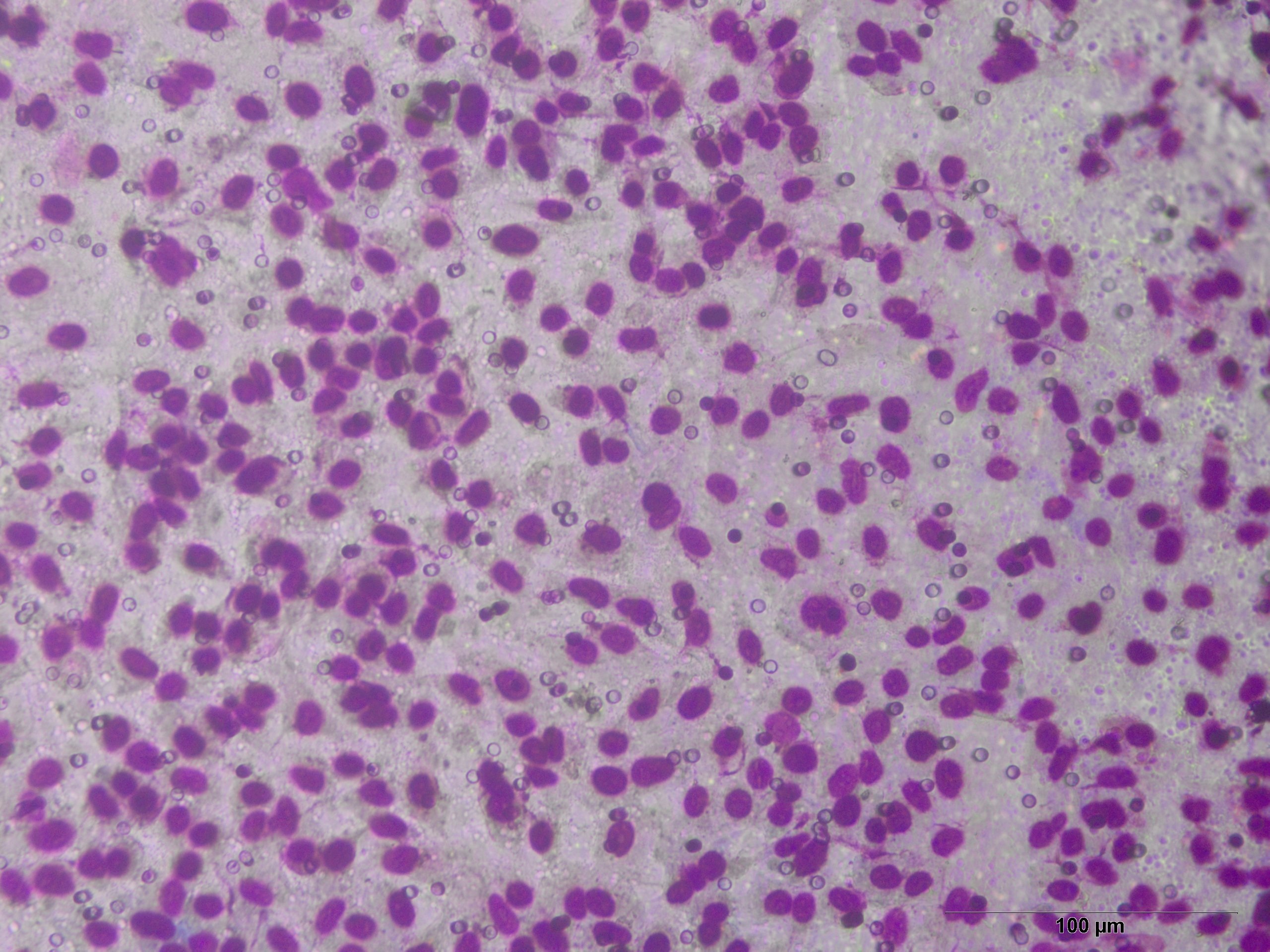

Supplement: Supplementary file 5 [file DataSheet_2.zip › Data Sheet 2/Fig2E/3-over-AC009948.5-INVASION.jpg]

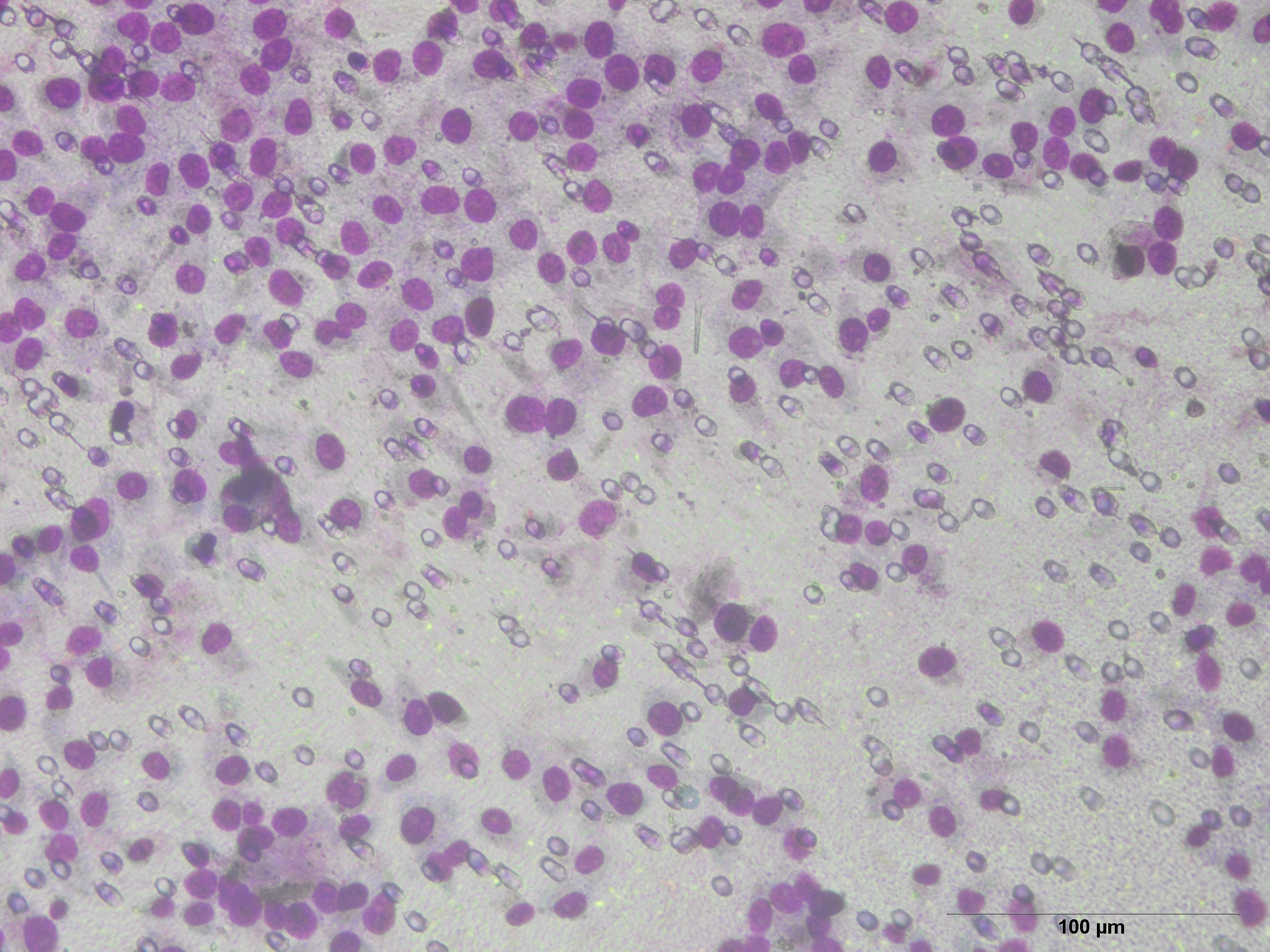

Supplement: Supplementary file 5 [file DataSheet_2.zip › Data Sheet 2/Fig2E/3-Scrambled-AC009948.5-A549-INVASION.jpg]

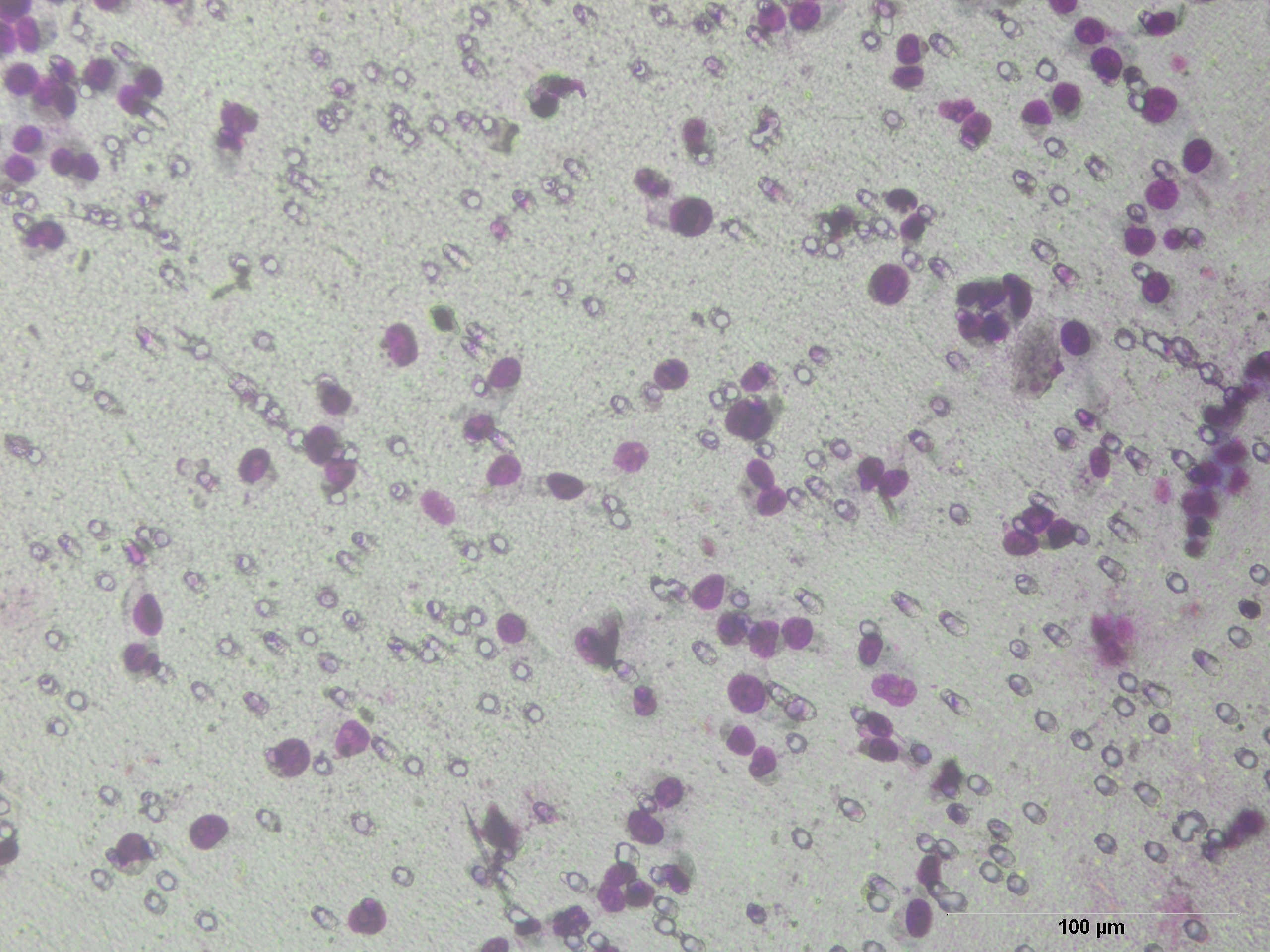

Supplement: Supplementary file 5 [file DataSheet_2.zip › Data Sheet 2/Fig2E/3-Si-AC009948.5-A549-INVASION.jpg]

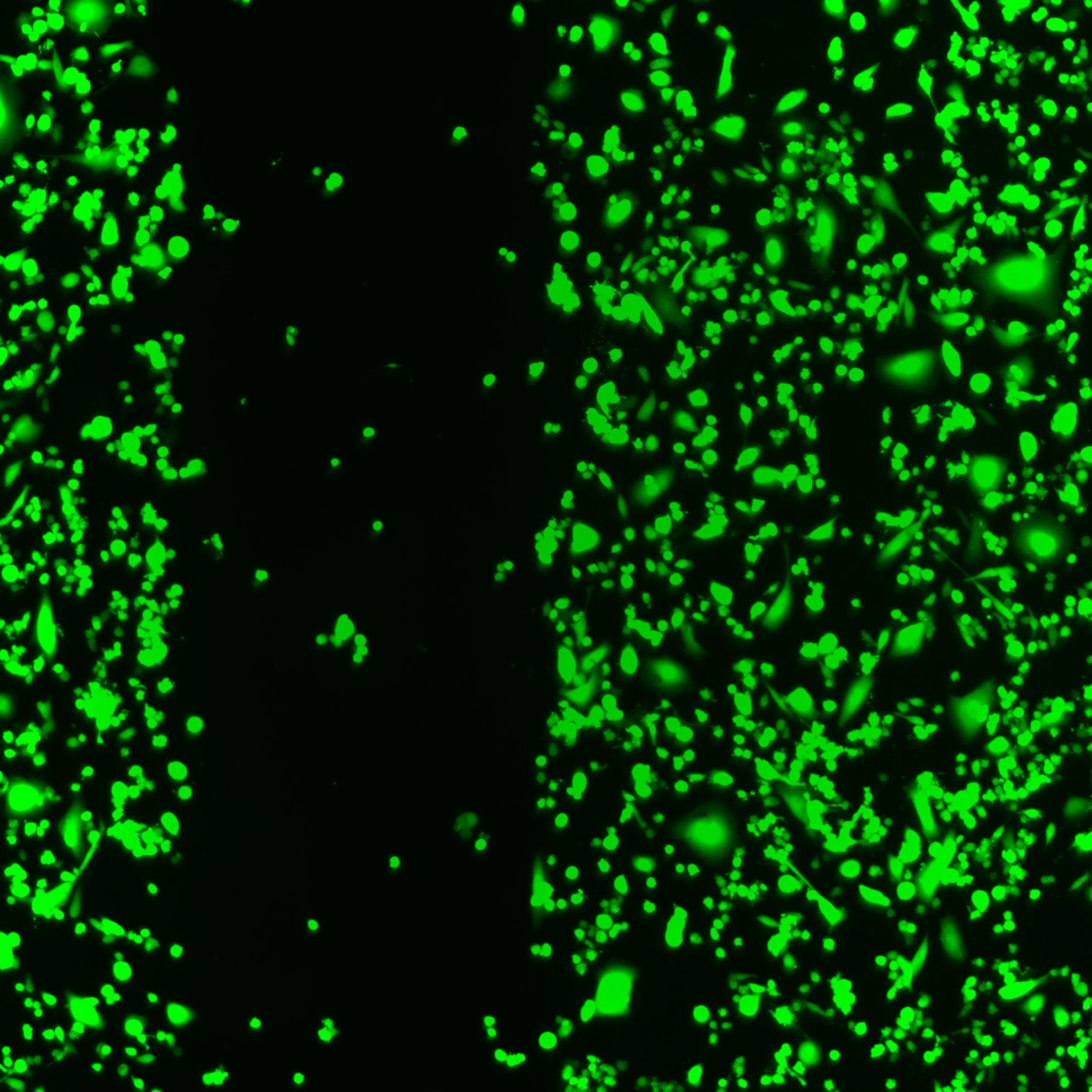

Supplement: Supplementary file 5 [file DataSheet_2.zip › Data Sheet 2/Fig2F/1-0H-NC.jpg]

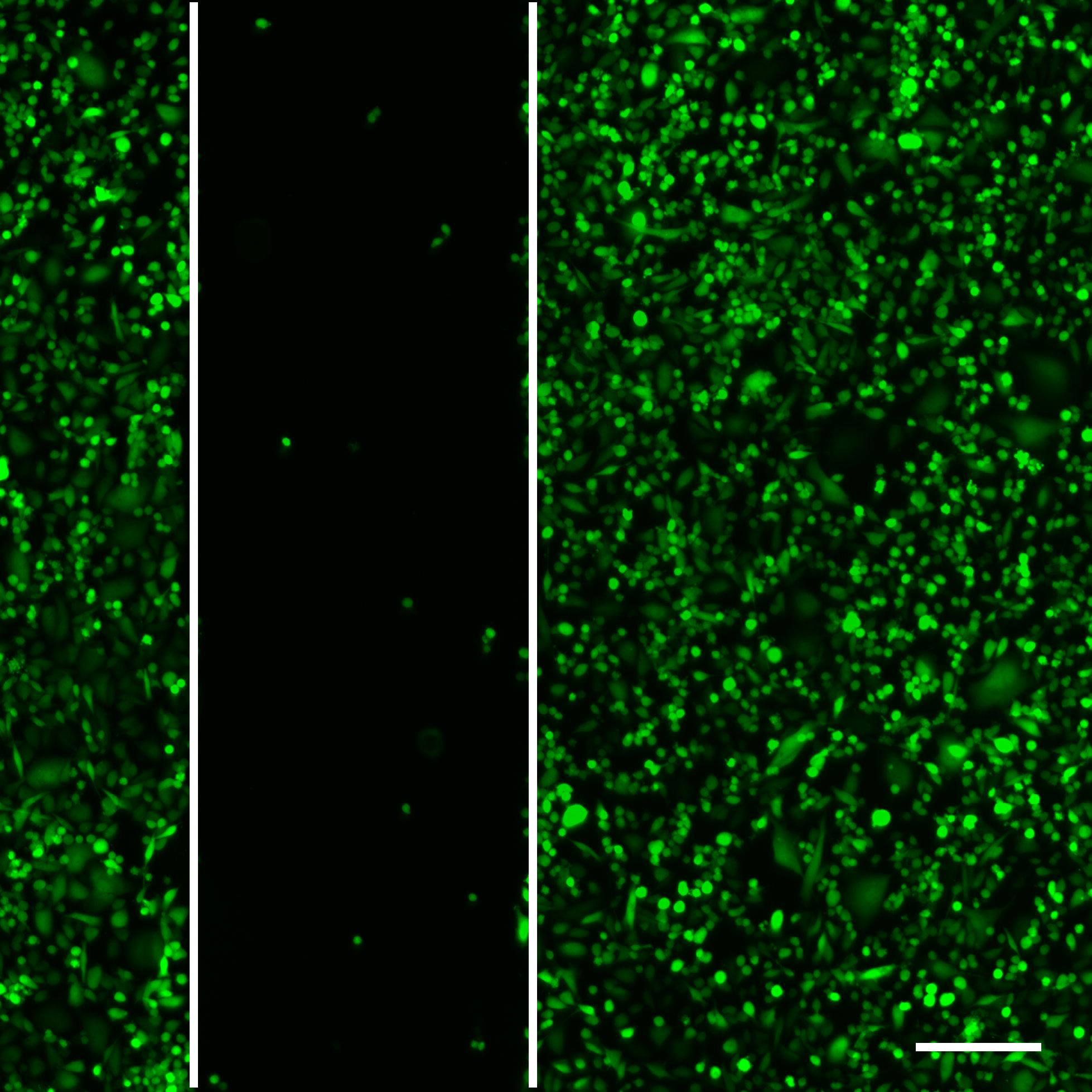

Supplement: Supplementary file 5 [file DataSheet_2.zip › Data Sheet 2/Fig2F/1-0H-over-AC009948.5.jpg]

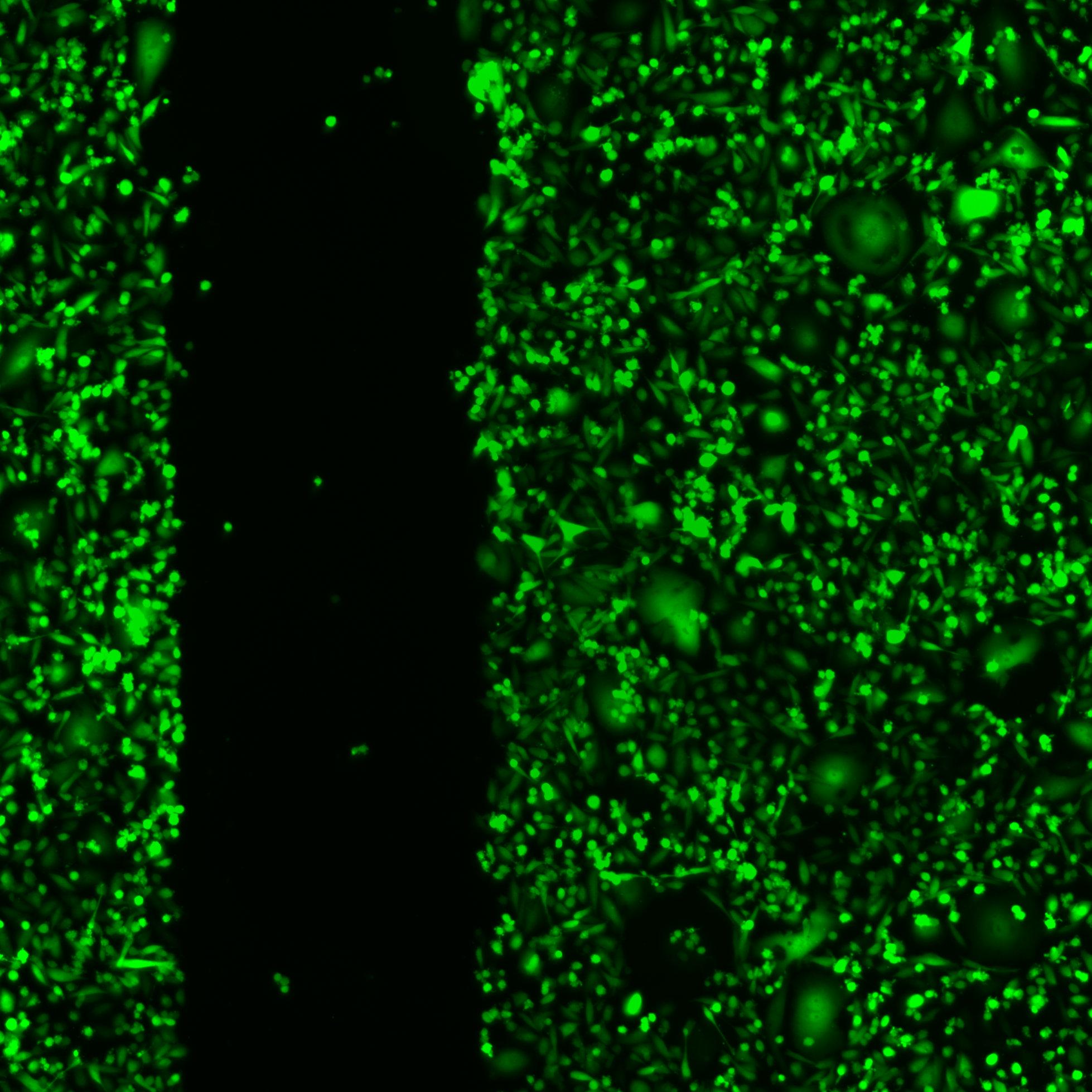

Supplement: Supplementary file 5 [file DataSheet_2.zip › Data Sheet 2/Fig2F/1-0H-scrambled.jpg]

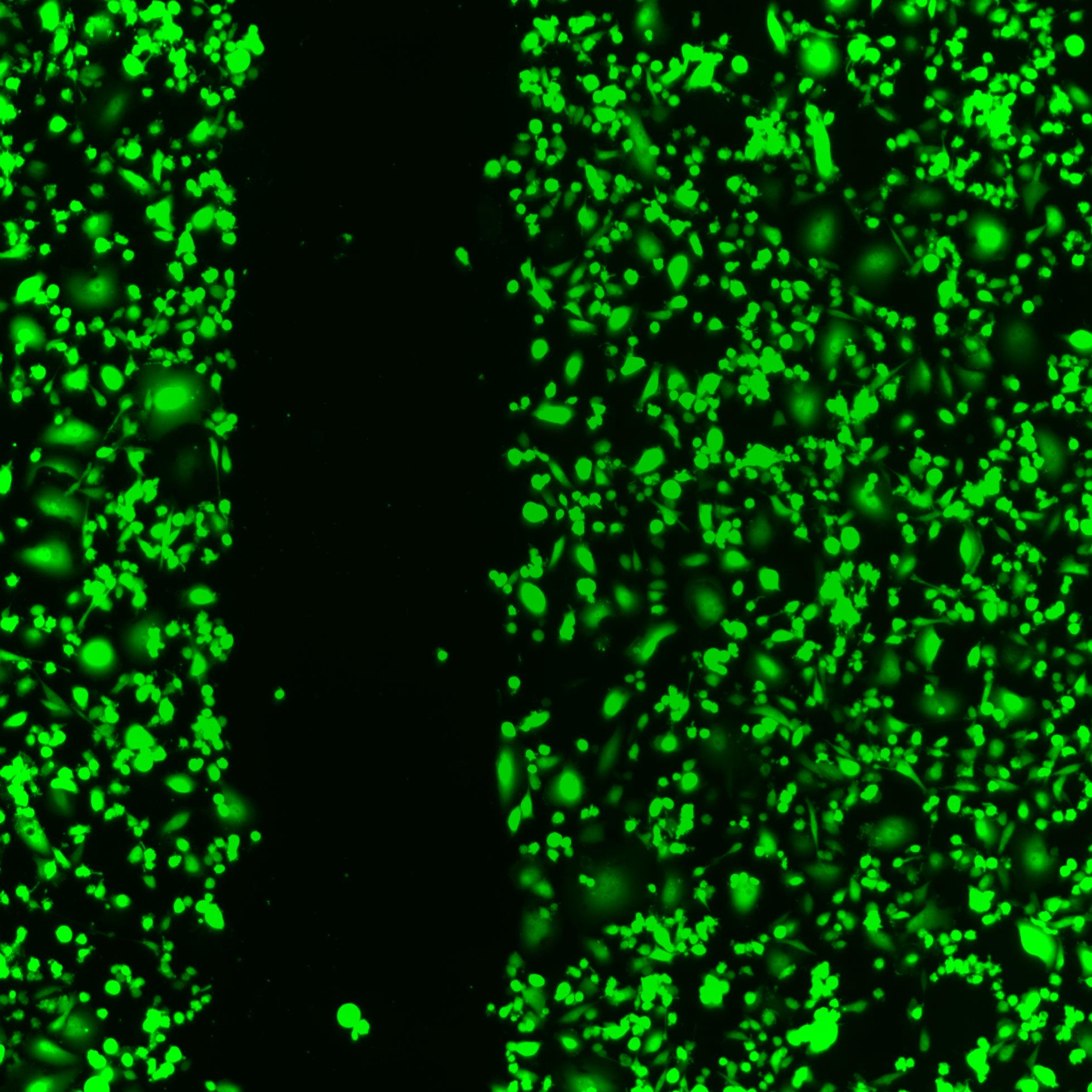

Supplement: Supplementary file 5 [file DataSheet_2.zip › Data Sheet 2/Fig2F/1-0H-SiAC009948.5.jpg]

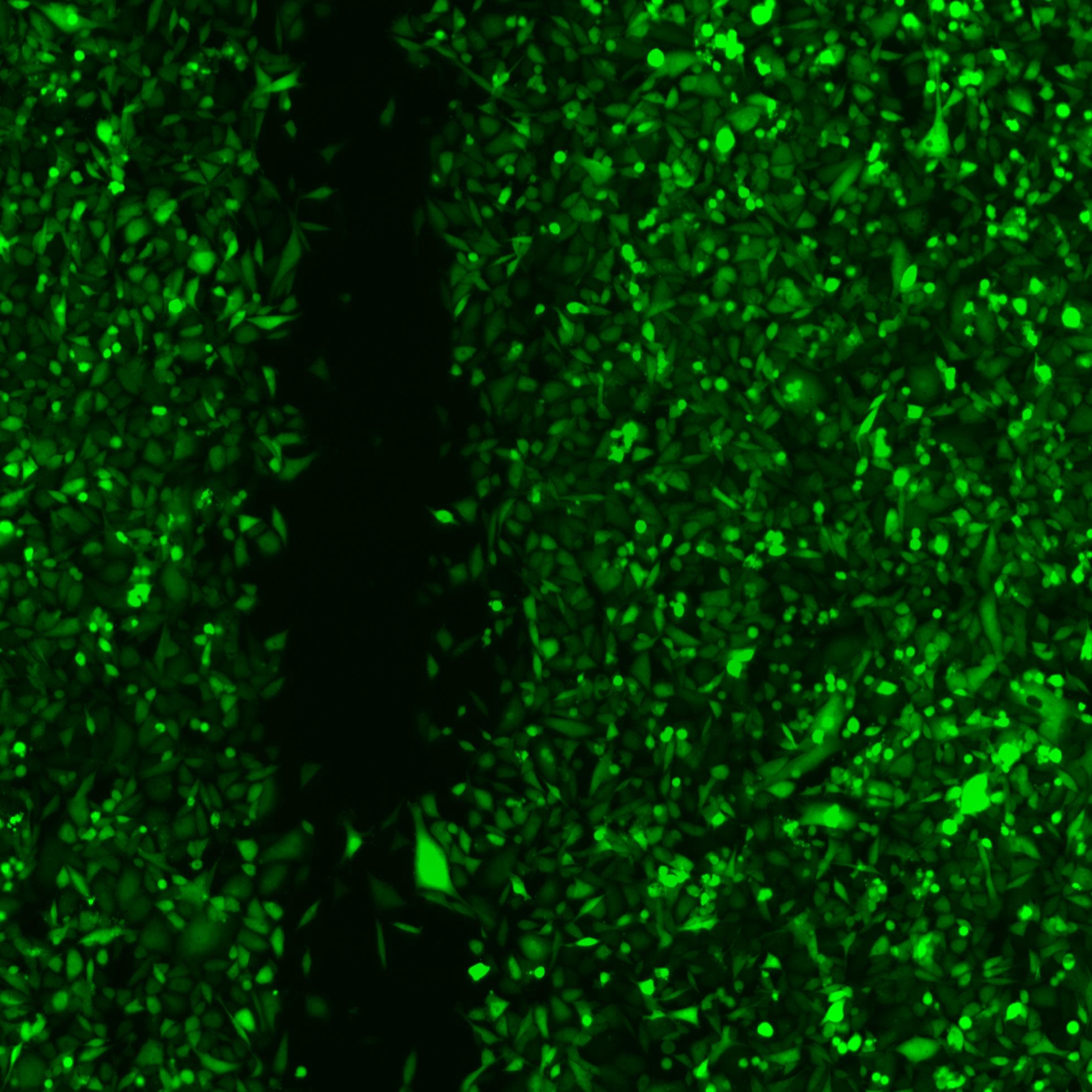

Supplement: Supplementary file 5 [file DataSheet_2.zip › Data Sheet 2/Fig2F/1-24H-NC.jpg]

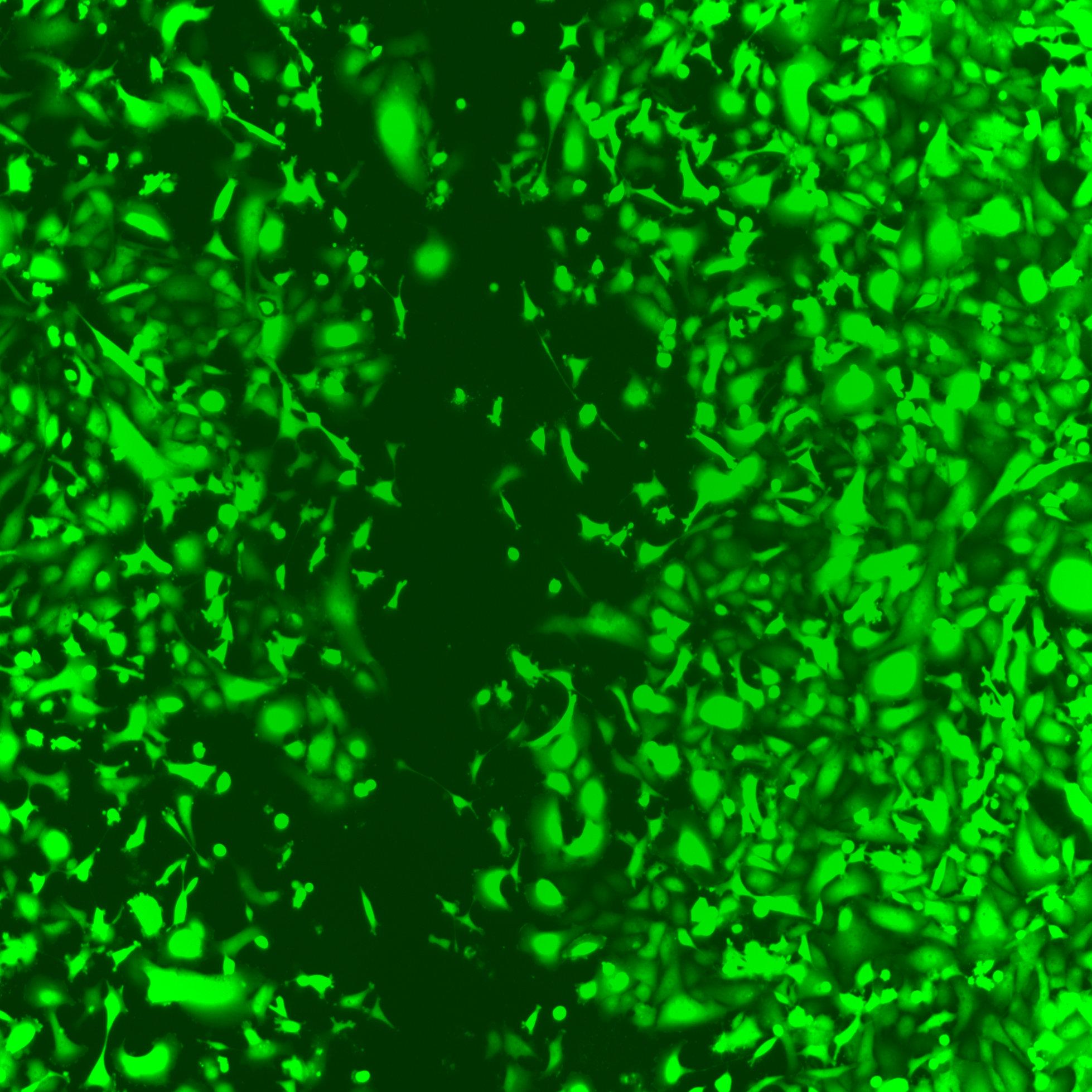

Supplement: Supplementary file 5 [file DataSheet_2.zip › Data Sheet 2/Fig2F/1-24H-over-AC009948.5.jpg]

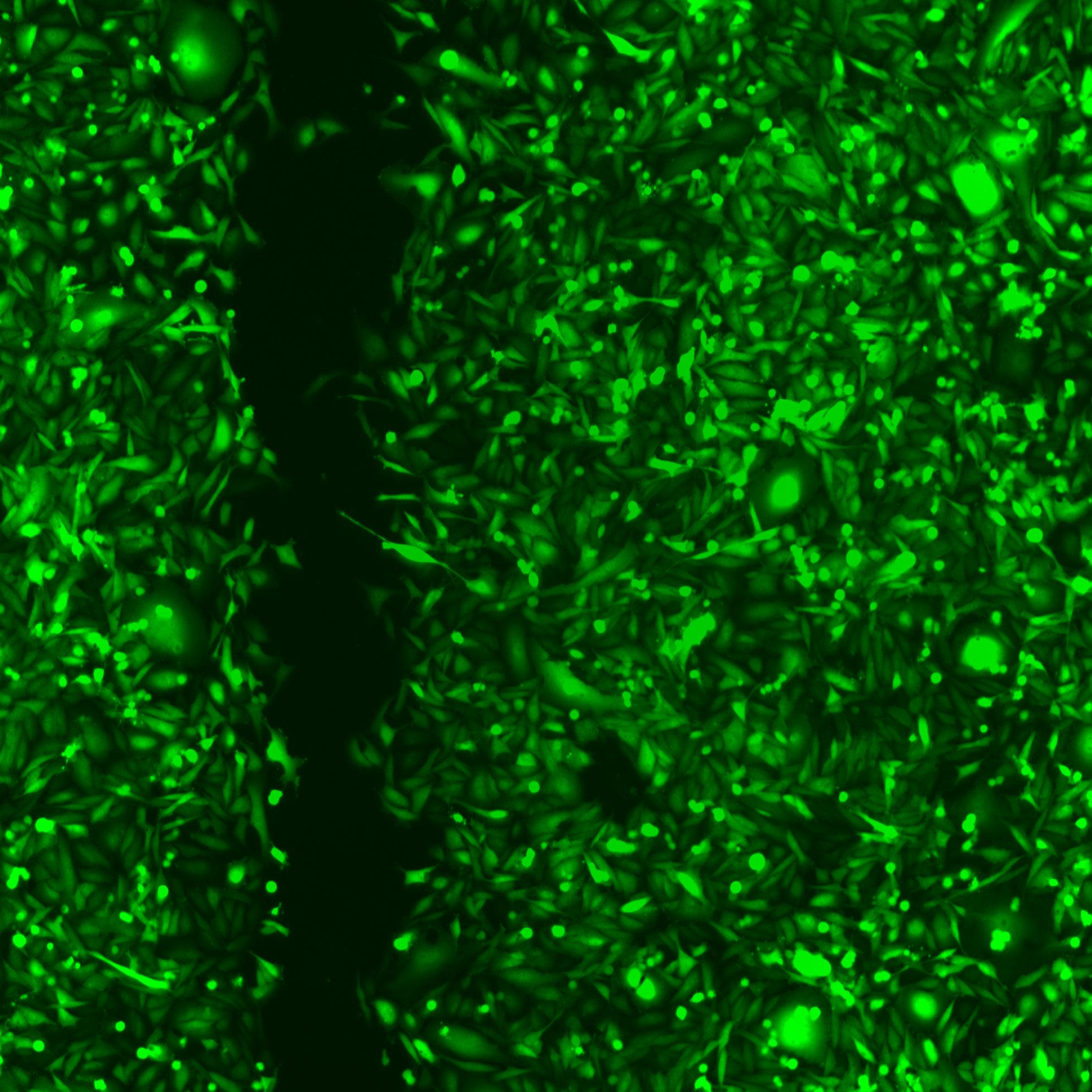

Supplement: Supplementary file 5 [file DataSheet_2.zip › Data Sheet 2/Fig2F/1-24H-Scrambled.jpg]

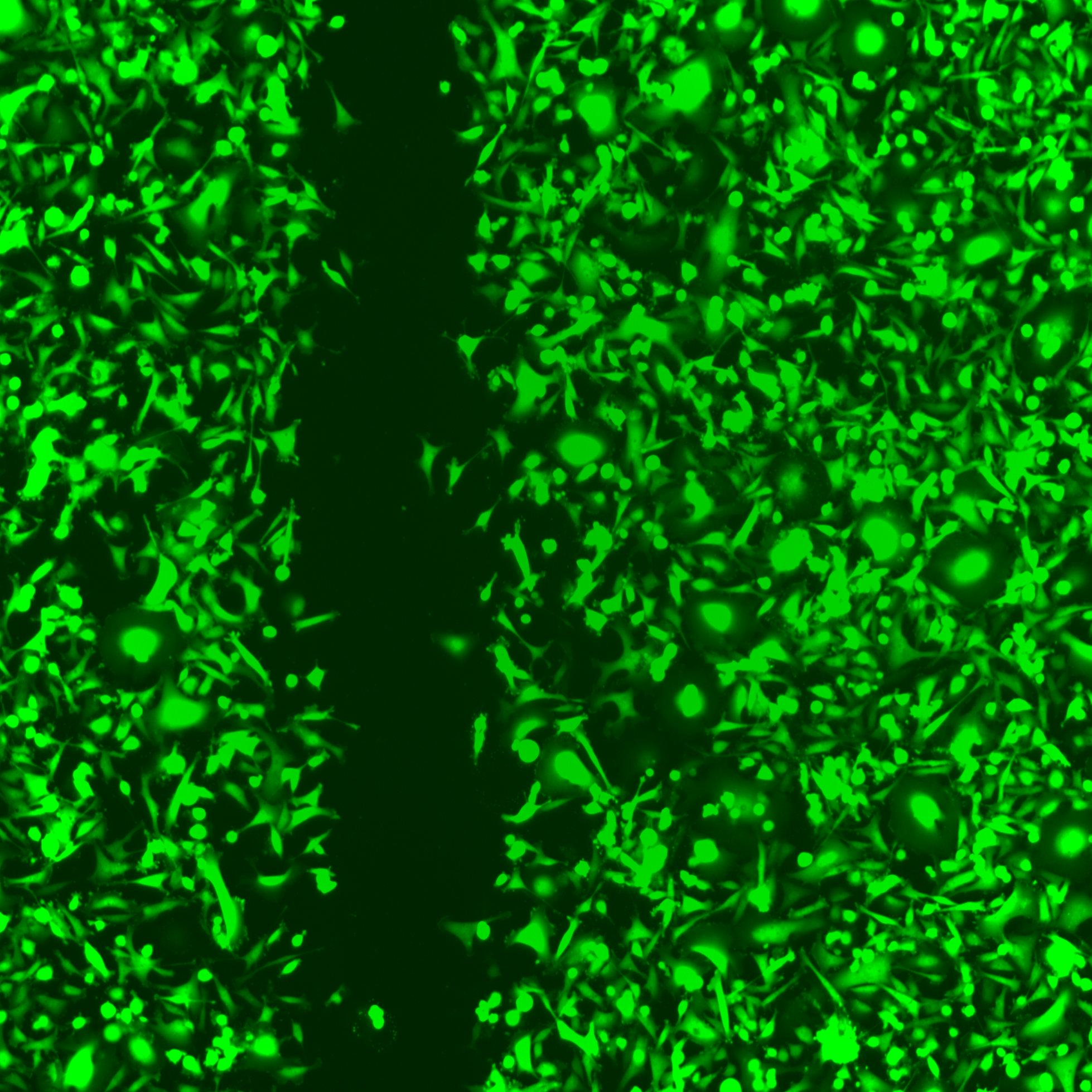

Supplement: Supplementary file 5 [file DataSheet_2.zip › Data Sheet 2/Fig2F/1-24H-SiAC009948.5.jpg]

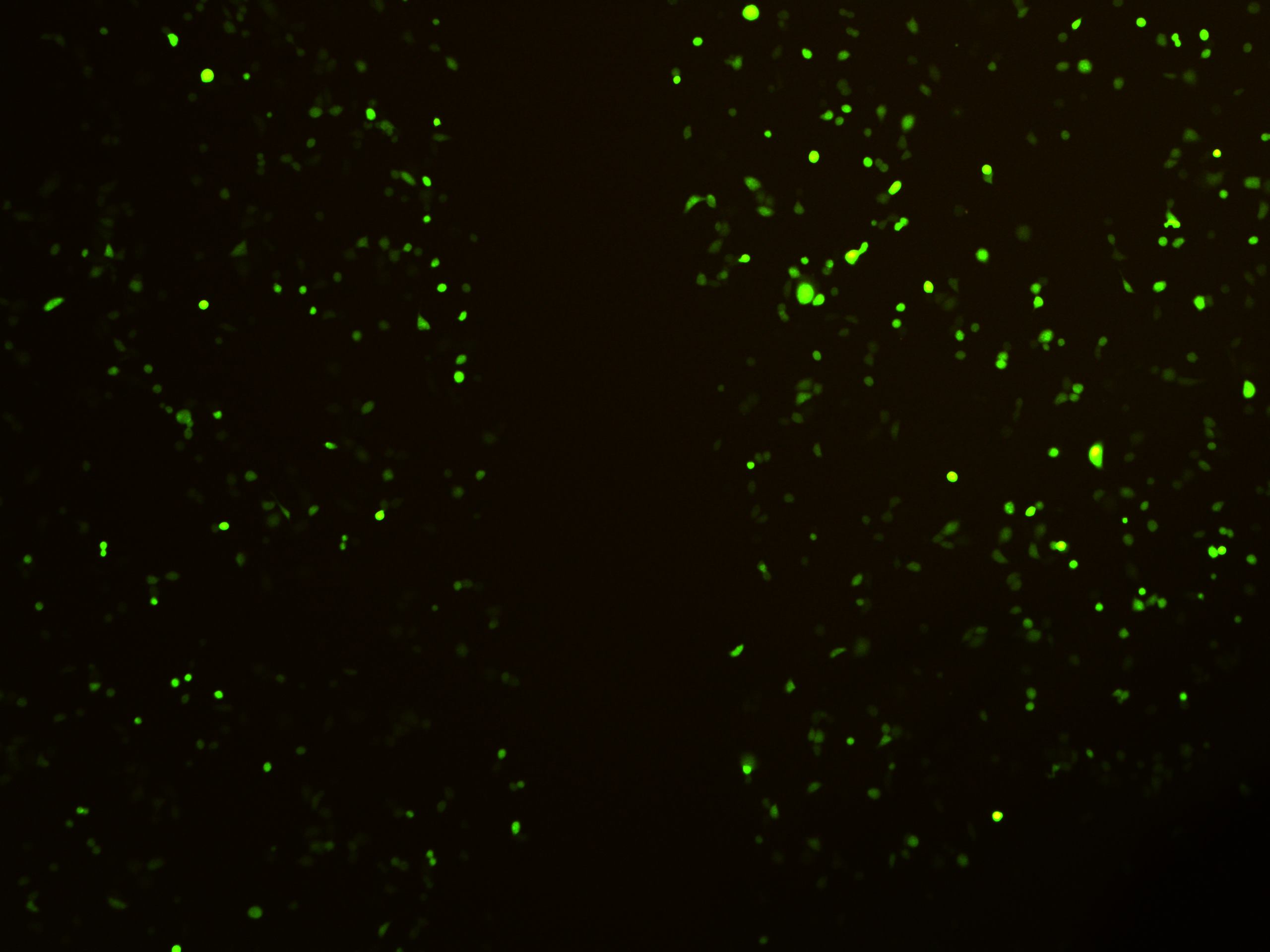

Supplement: Supplementary file 5 [file DataSheet_2.zip › Data Sheet 2/Fig2F/2-0H-NC.jpg]

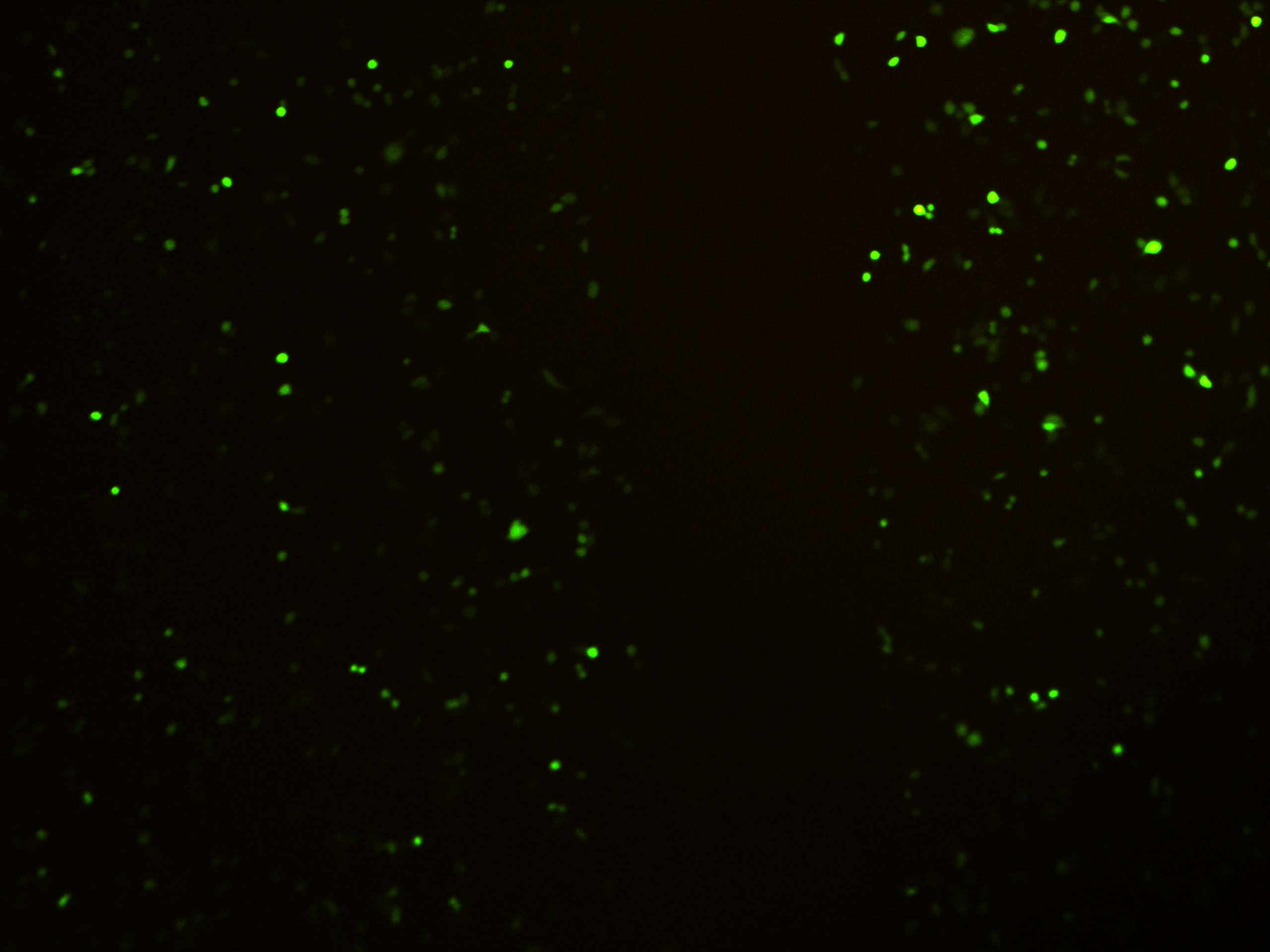

Supplement: Supplementary file 5 [file DataSheet_2.zip › Data Sheet 2/Fig2F/2-0H-over-AC009948.5.jpg]

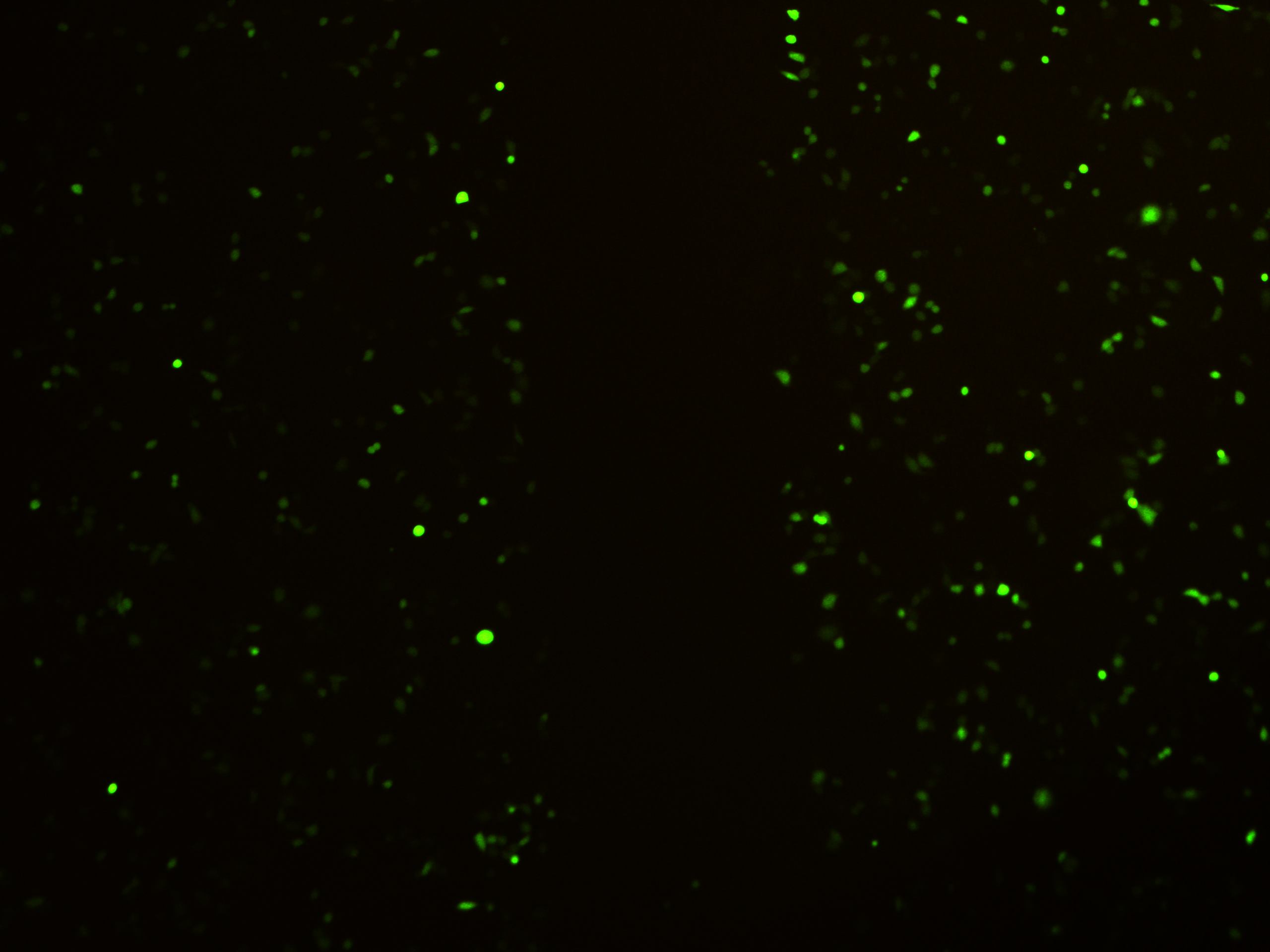

Supplement: Supplementary file 5 [file DataSheet_2.zip › Data Sheet 2/Fig2F/2-0H-scrambled.jpg]

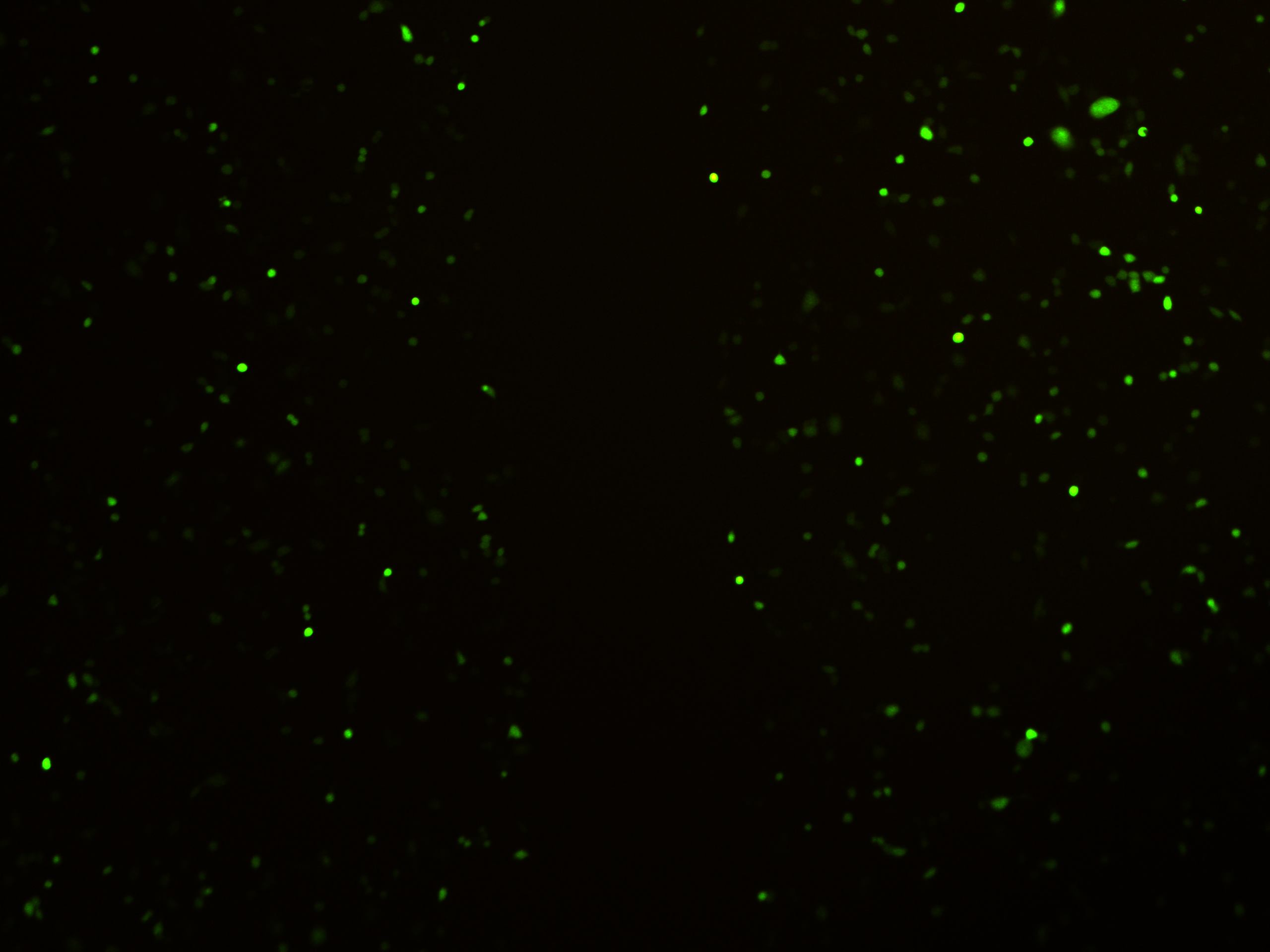

Supplement: Supplementary file 5 [file DataSheet_2.zip › Data Sheet 2/Fig2F/2-0H-SiAC009948.5.jpg]

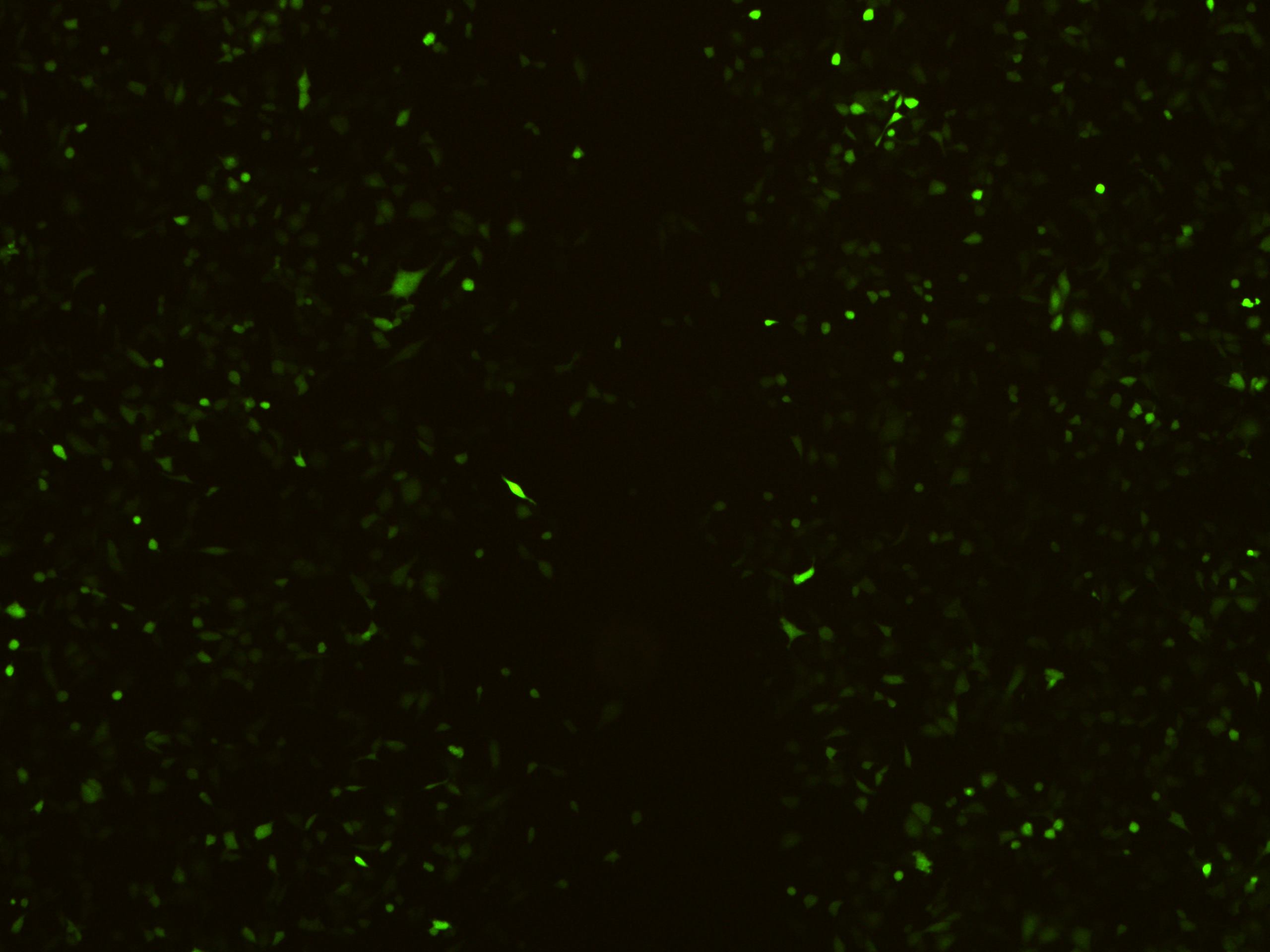

Supplement: Supplementary file 5 [file DataSheet_2.zip › Data Sheet 2/Fig2F/2-24H-NC.jpg]

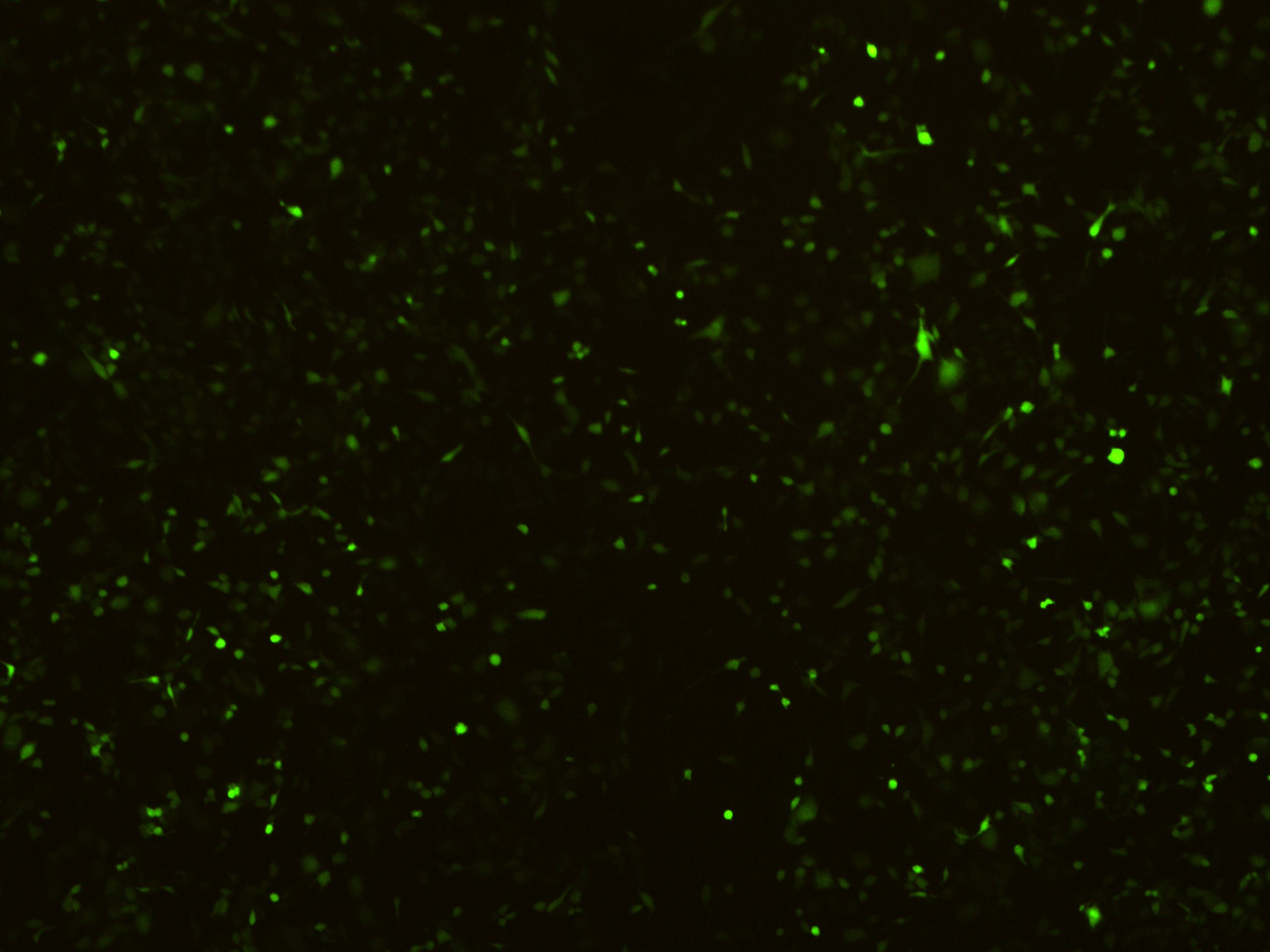

Supplement: Supplementary file 5 [file DataSheet_2.zip › Data Sheet 2/Fig2F/2-24H-over-AC009948.5.jpg]

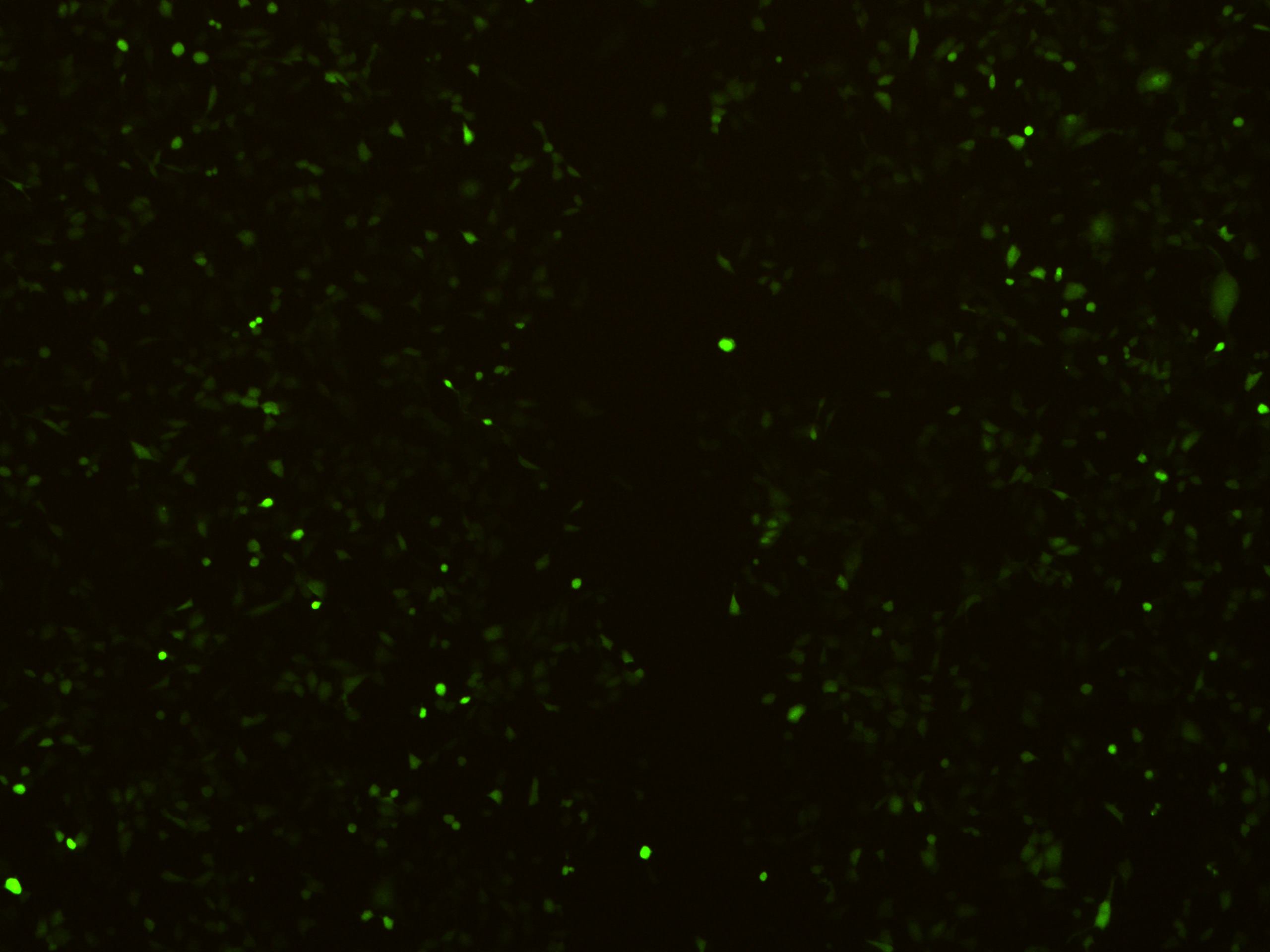

Supplement: Supplementary file 5 [file DataSheet_2.zip › Data Sheet 2/Fig2F/2-24H-Scrambled.jpg]

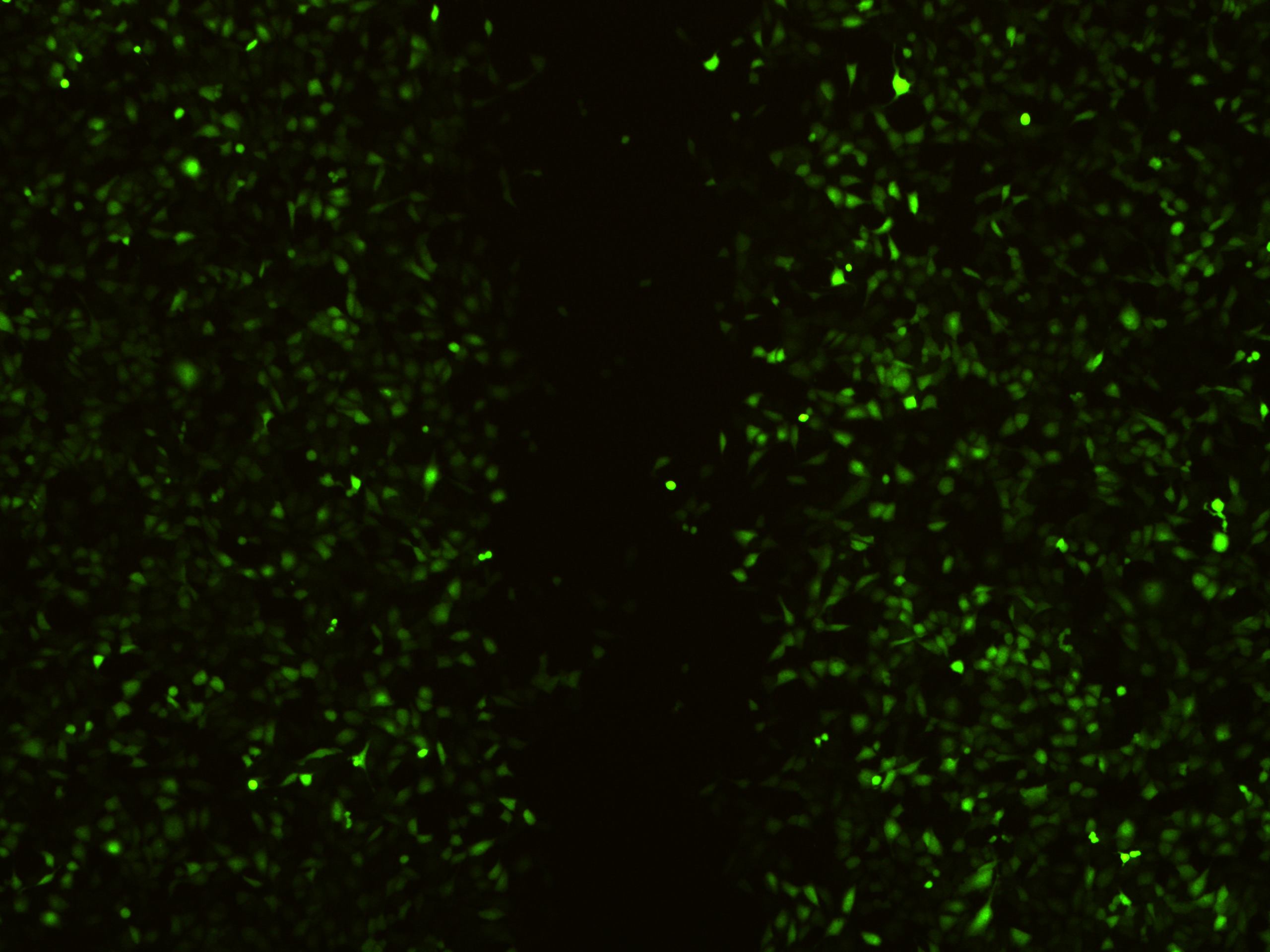

Supplement: Supplementary file 5 [file DataSheet_2.zip › Data Sheet 2/Fig2F/2-24H-SiAC009948.5.jpg]

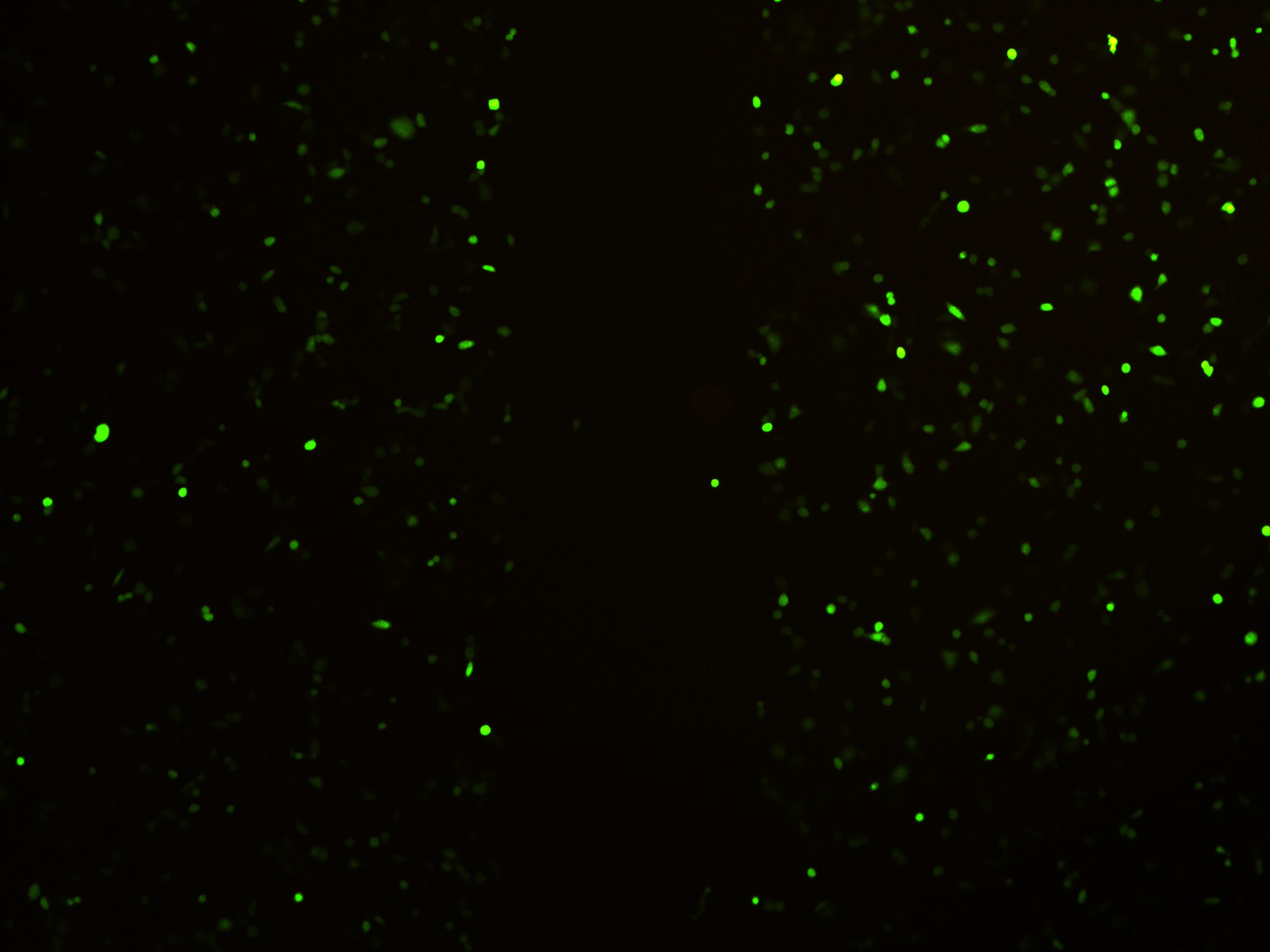

Supplement: Supplementary file 5 [file DataSheet_2.zip › Data Sheet 2/Fig2F/3-0H-NC.jpg]

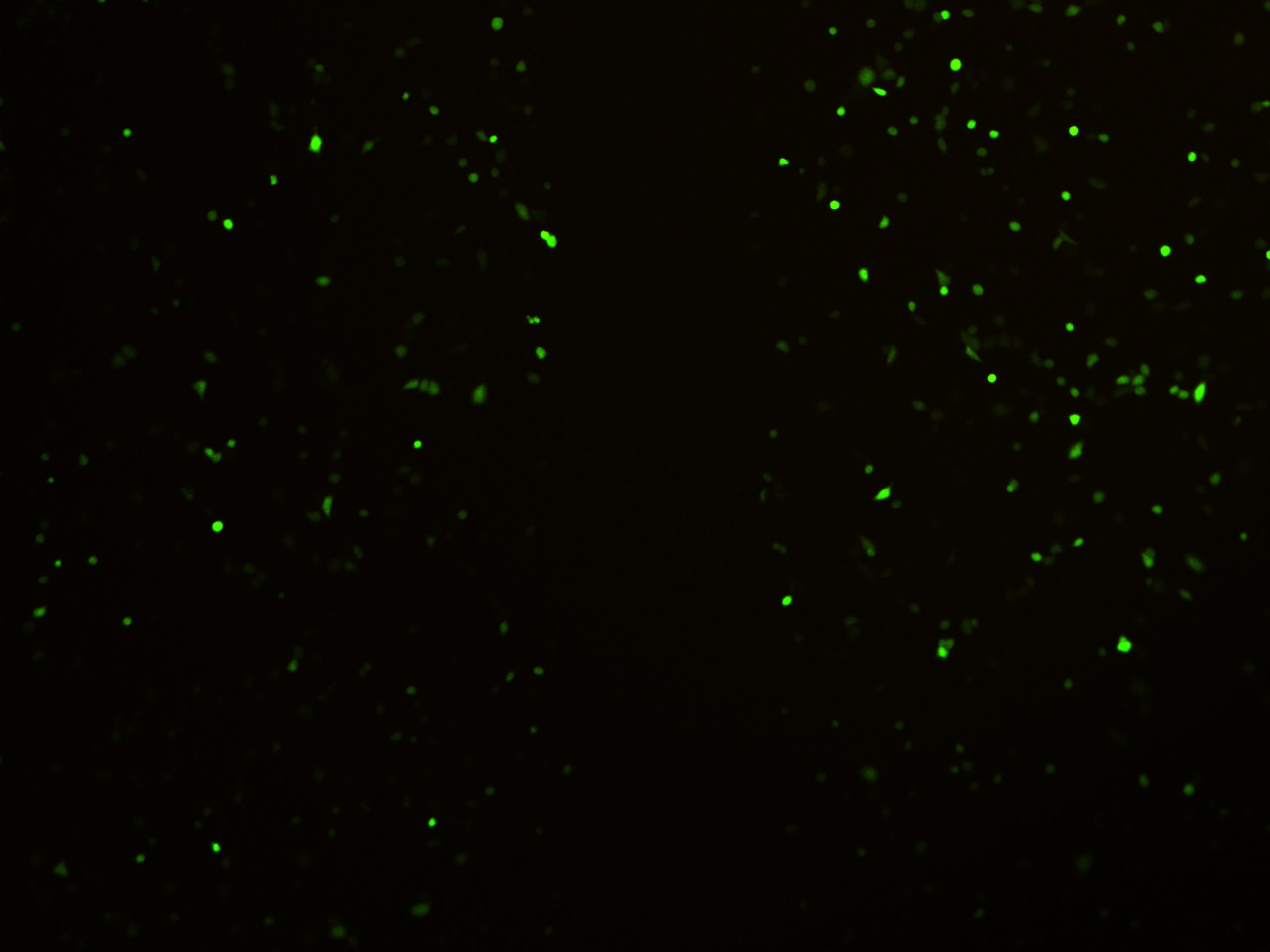

Supplement: Supplementary file 5 [file DataSheet_2.zip › Data Sheet 2/Fig2F/3-0H-over-AC009948.5.jpg]

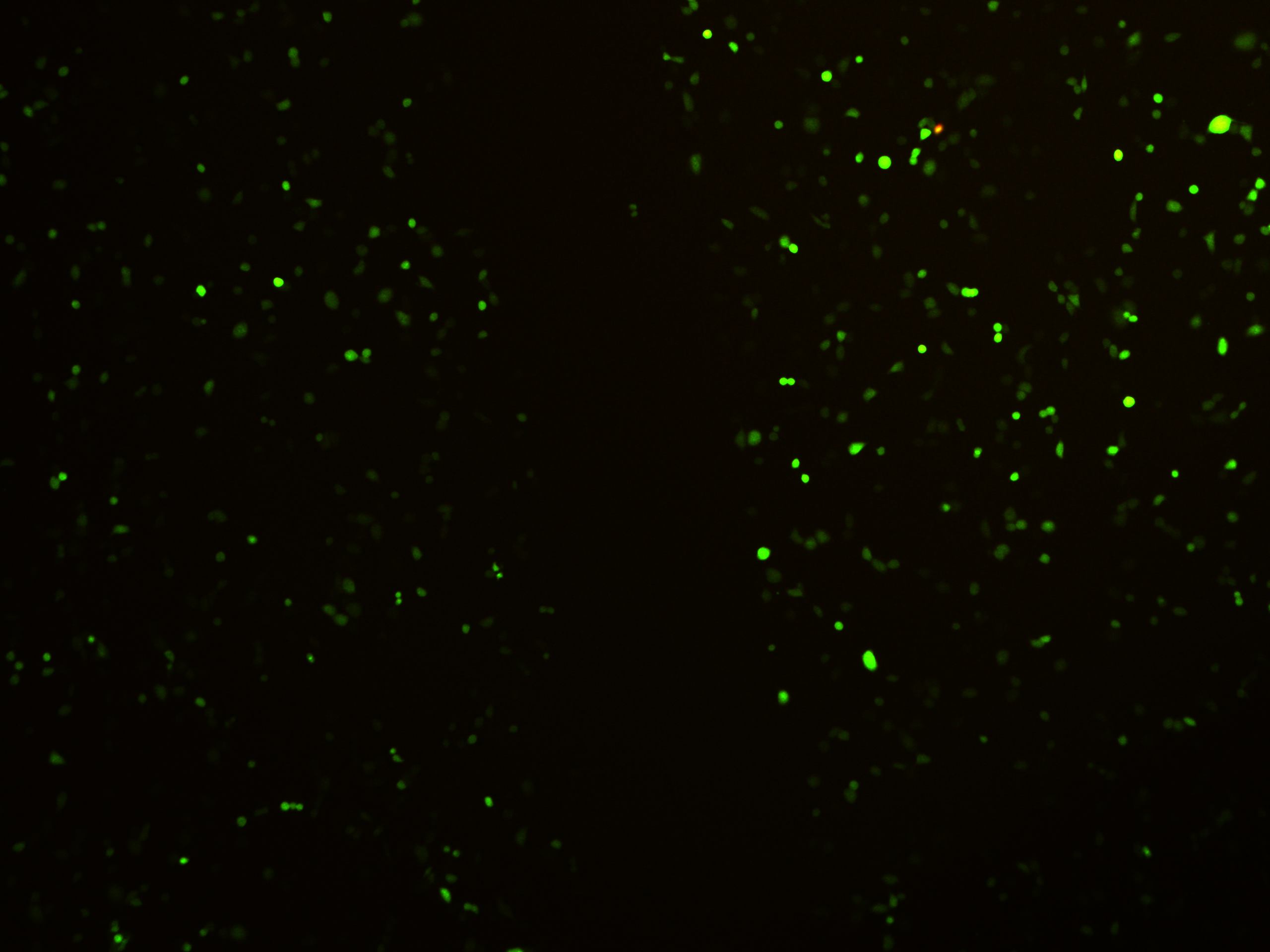

Supplement: Supplementary file 5 [file DataSheet_2.zip › Data Sheet 2/Fig2F/3-0H-scrambled.jpg]

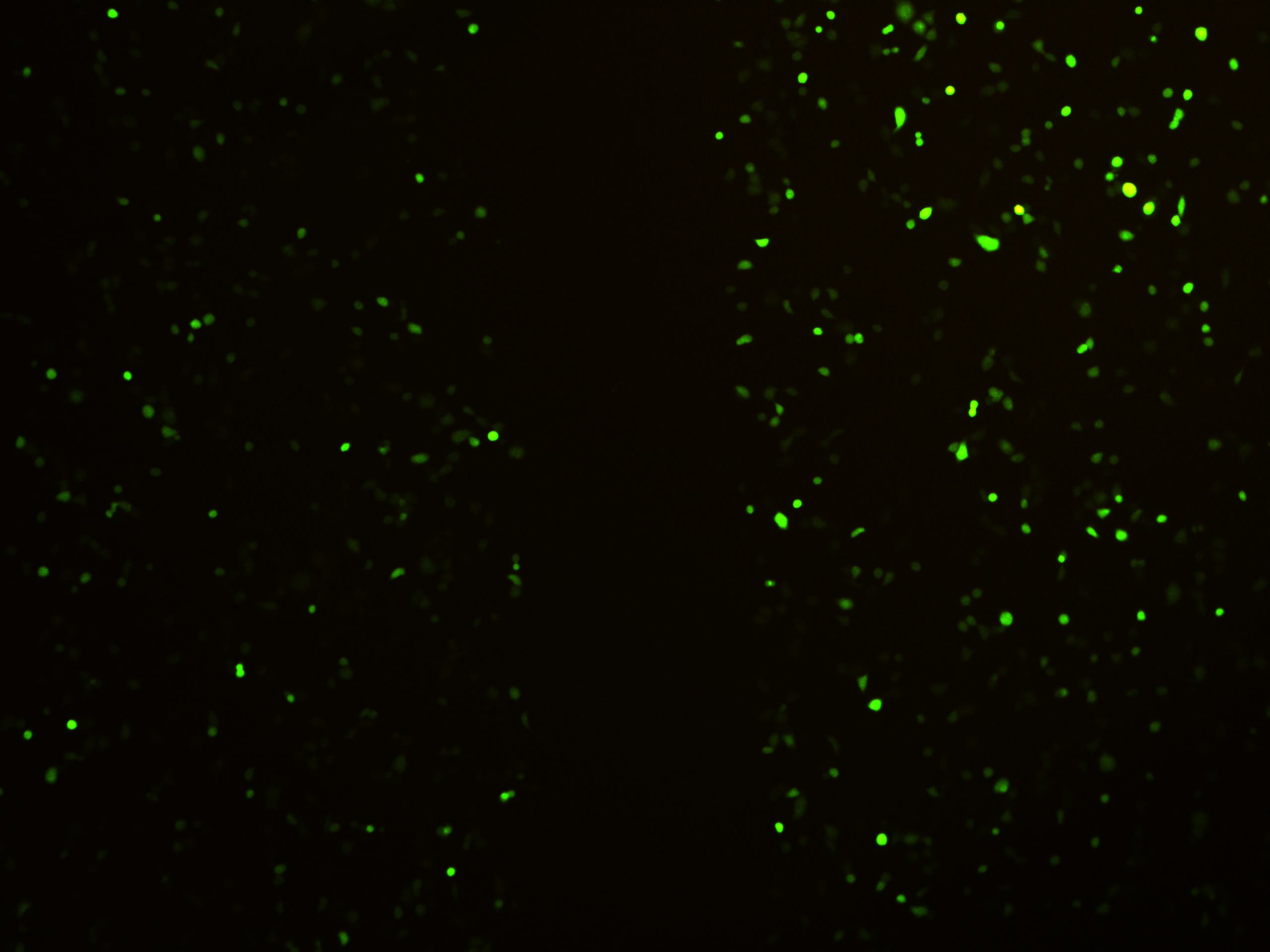

Supplement: Supplementary file 5 [file DataSheet_2.zip › Data Sheet 2/Fig2F/3-0H-SiAC009948.5.jpg]

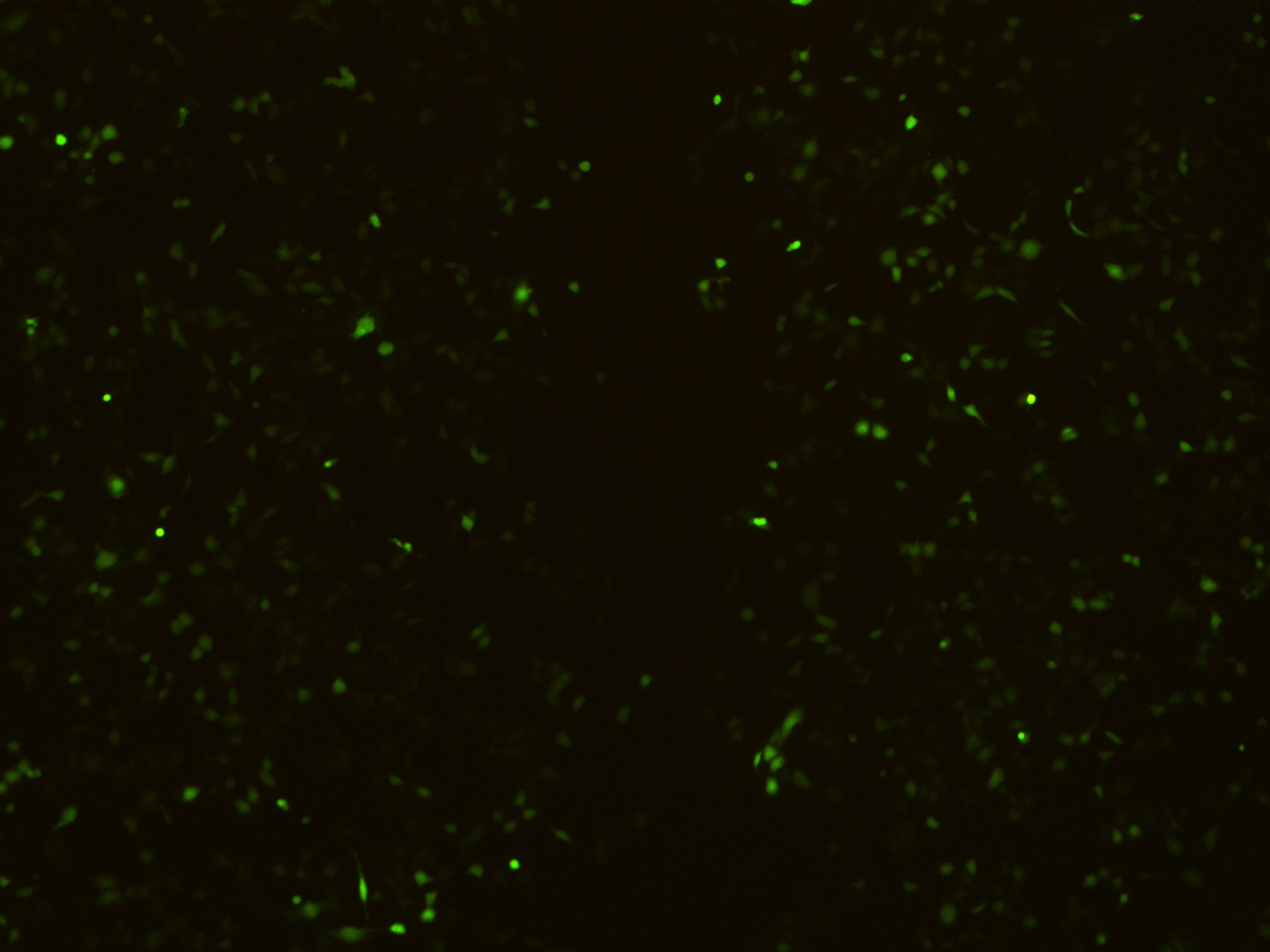

Supplement: Supplementary file 5 [file DataSheet_2.zip › Data Sheet 2/Fig2F/3-24H-NC.jpg]

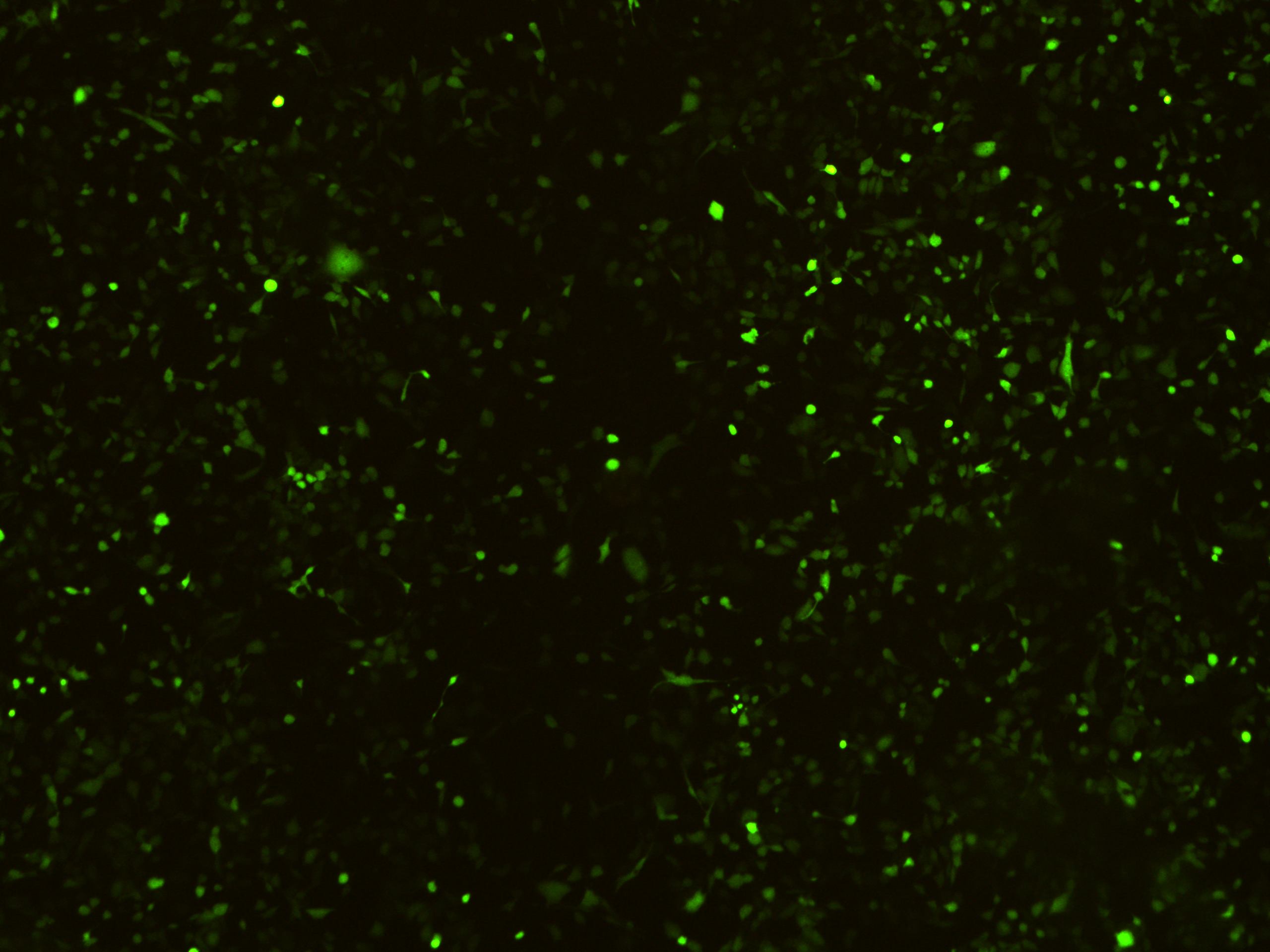

Supplement: Supplementary file 5 [file DataSheet_2.zip › Data Sheet 2/Fig2F/3-24H-over-AC009948.5.jpg]

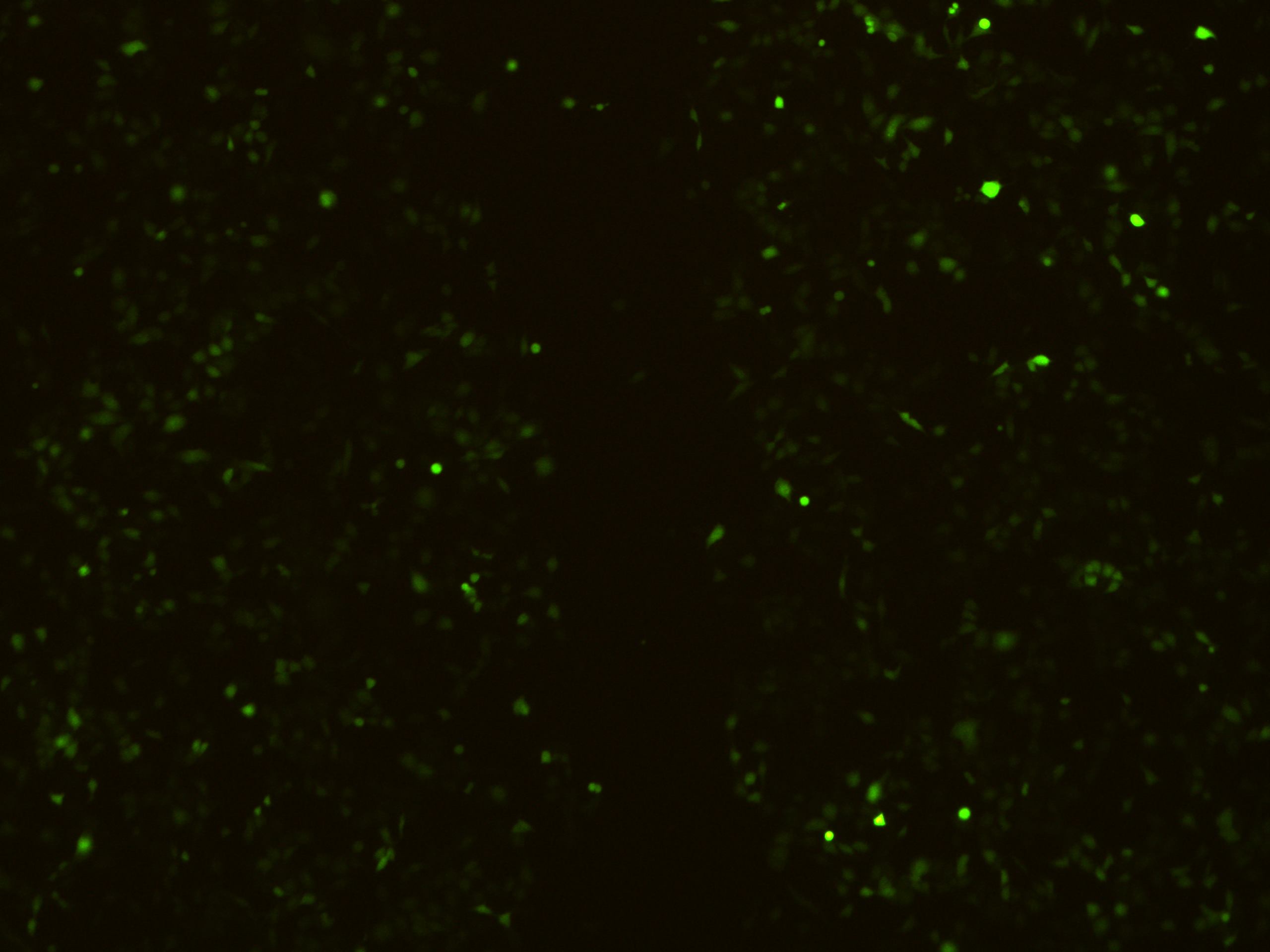

Supplement: Supplementary file 5 [file DataSheet_2.zip › Data Sheet 2/Fig2F/3-24H-Scrambled.jpg]

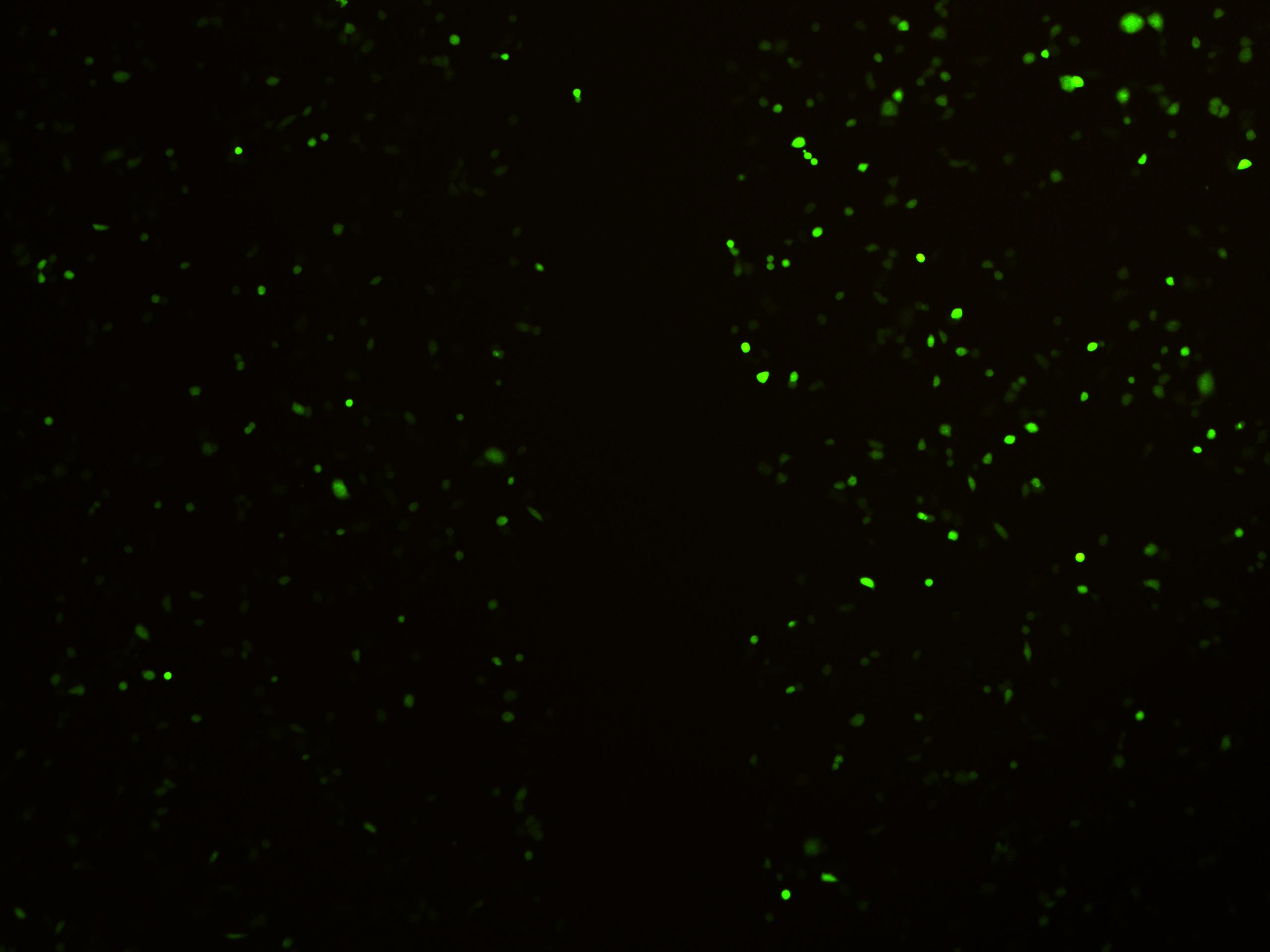

Supplement: Supplementary file 5 [file DataSheet_2.zip › Data Sheet 2/Fig2F/3-24H-SiAC009948.5.jpg]

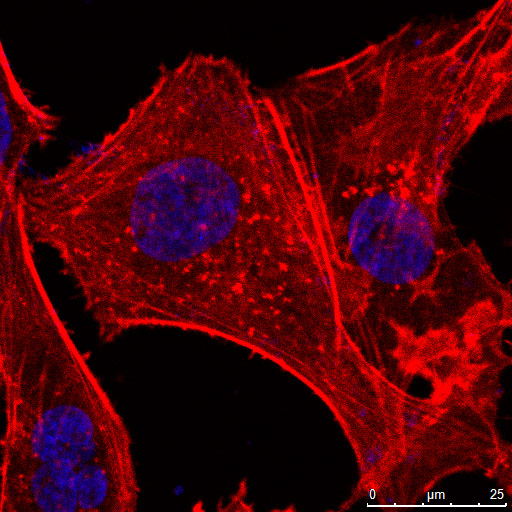

Supplement: Supplementary file 5 [file DataSheet_2.zip › Data Sheet 2/Fig5B/1-AC009948.5-CO-factin-_z0.jpg]

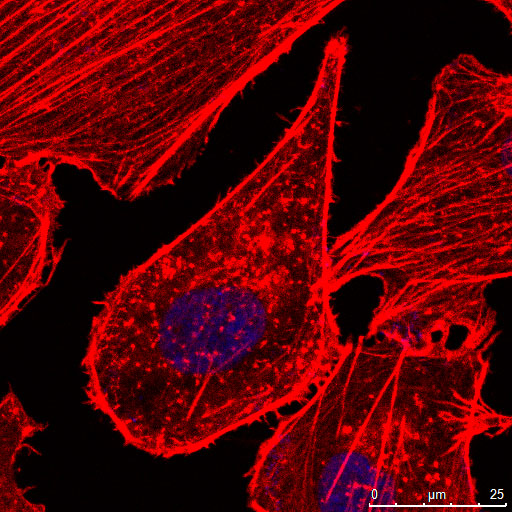

Supplement: Supplementary file 5 [file DataSheet_2.zip › Data Sheet 2/Fig5B/1-AC009948.5-factin-con-Series007_z0.jpg]

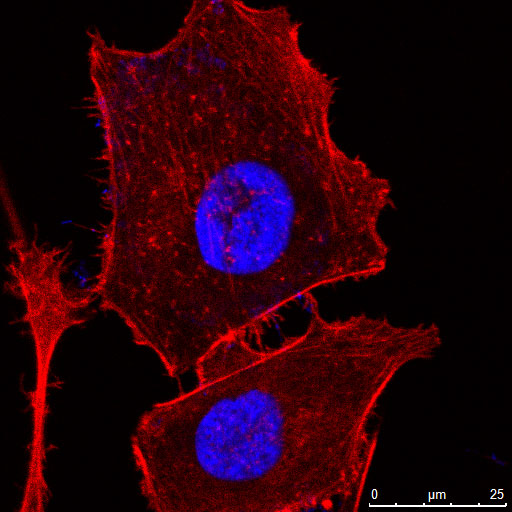

Supplement: Supplementary file 5 [file DataSheet_2.zip › Data Sheet 2/Fig5B/1-AC009948.5-over-miR-186-Series074_z0.jpg]

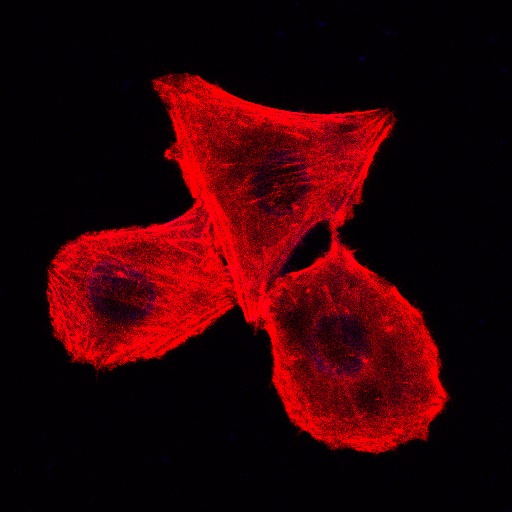

Supplement: Supplementary file 5 [file DataSheet_2.zip › Data Sheet 2/Fig5B/1-AC009948.5-Scrambled-Series023_z0.jpg]

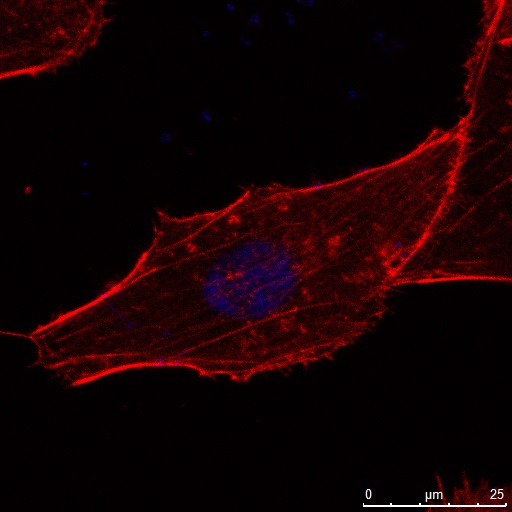

Supplement: Supplementary file 5 [file DataSheet_2.zip › Data Sheet 2/Fig5B/1-SiAC009948.5-factin-_Series038_z0.jpg]

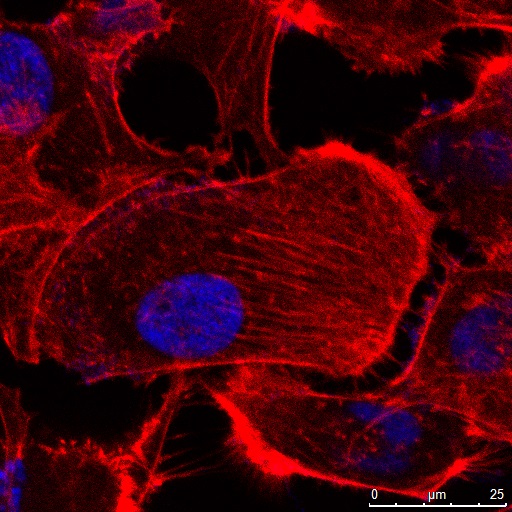

Supplement: Supplementary file 5 [file DataSheet_2.zip › Data Sheet 2/Fig5B/2-AC009948.5-CO_Series034_z0.jpg]

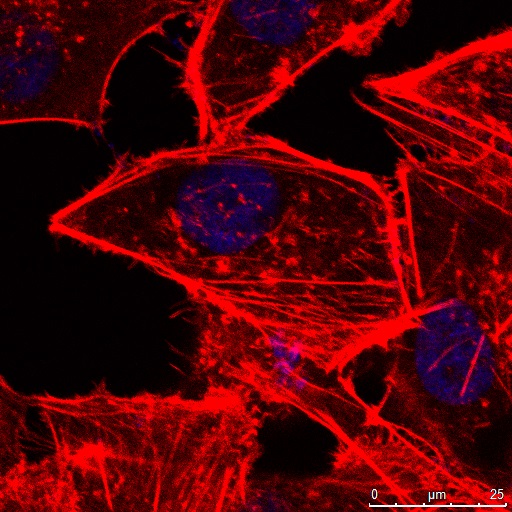

Supplement: Supplementary file 5 [file DataSheet_2.zip › Data Sheet 2/Fig5B/2-AC009948.5-factin-con-_Series056_z0.jpg]

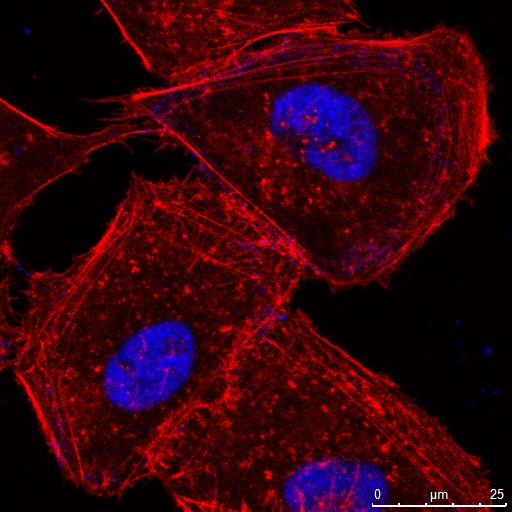

Supplement: Supplementary file 5 [file DataSheet_2.zip › Data Sheet 2/Fig5B/2-AC009948.5-over-miR-186-_Series028_z0.jpg]

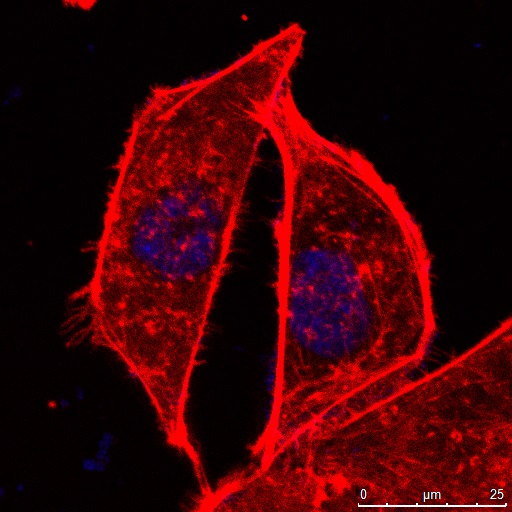

Supplement: Supplementary file 5 [file DataSheet_2.zip › Data Sheet 2/Fig5B/2-AC009948.5-Scrambled-Series060_z0.jpg]

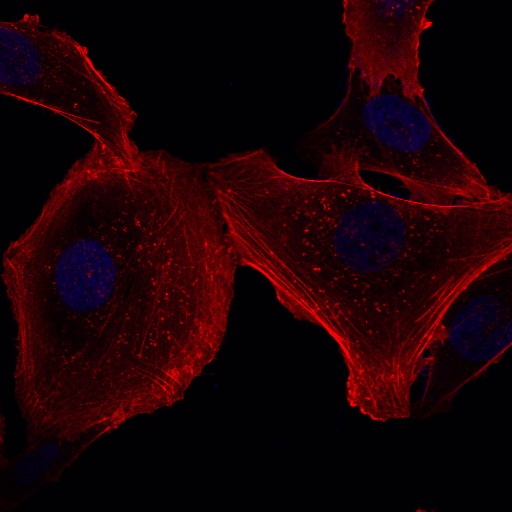

Supplement: Supplementary file 5 [file DataSheet_2.zip › Data Sheet 2/Fig5B/2-SiAC009948.5-Experiment001_Series152_z0.jpg]

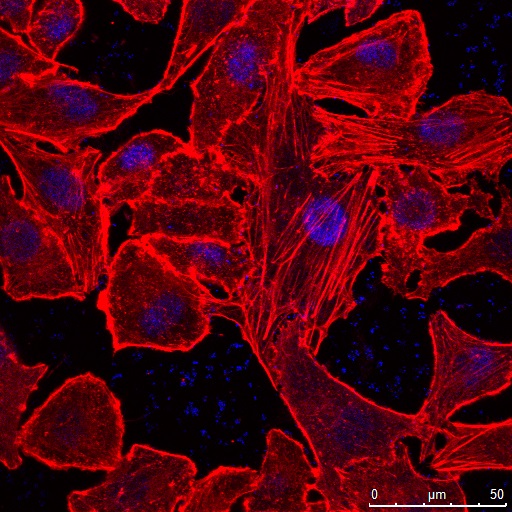

Supplement: Supplementary file 5 [file DataSheet_2.zip › Data Sheet 2/Fig5B/3-AC009948.5-CO_Series027_z0.jpg]

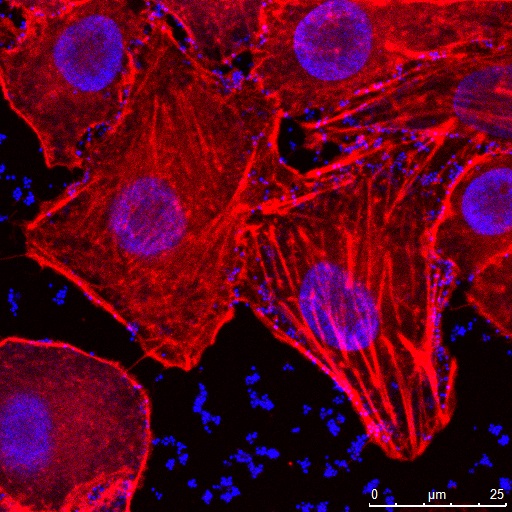

Supplement: Supplementary file 5 [file DataSheet_2.zip › Data Sheet 2/Fig5B/3-AC009948.5-factin-con-_Series008_z0.jpg]

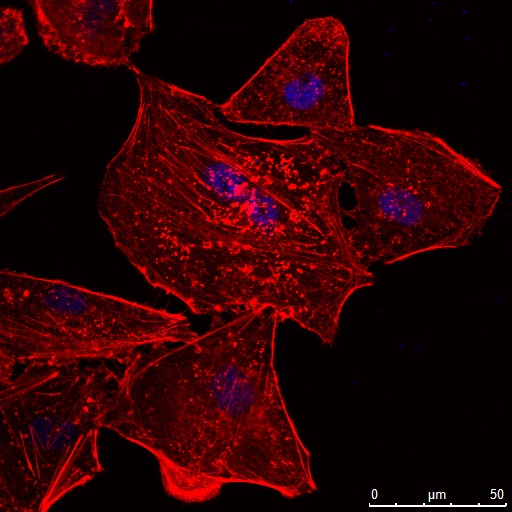

Supplement: Supplementary file 5 [file DataSheet_2.zip › Data Sheet 2/Fig5B/3-AC009948.5-over-miR-186_Series041_z0.jpg]

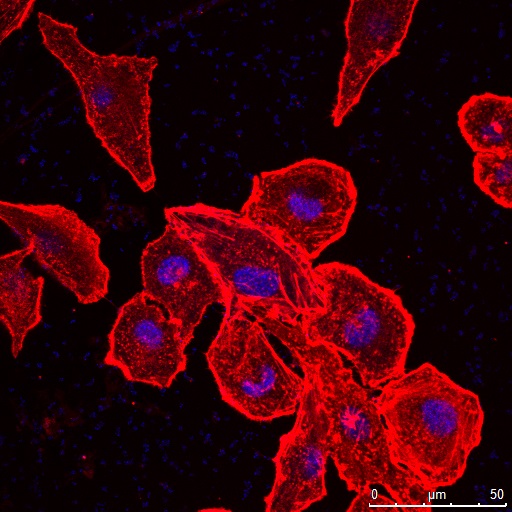

Supplement: Supplementary file 5 [file DataSheet_2.zip › Data Sheet 2/Fig5B/3-AC009948.5-Scrambled-Series009_z0.jpg]

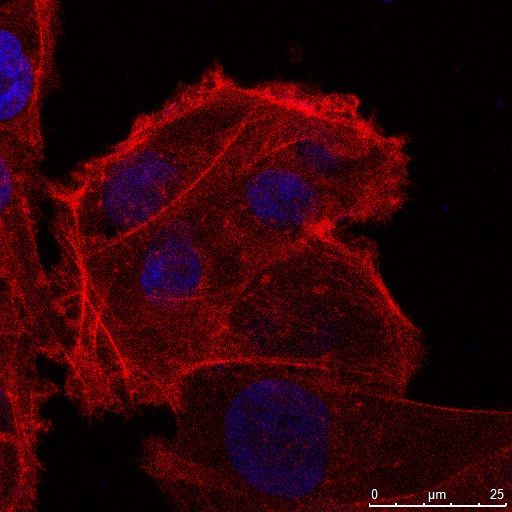

Supplement: Supplementary file 5 [file DataSheet_2.zip › Data Sheet 2/Fig5B/3-SiAC009948.5-factin-_Series016_z0.jpg]

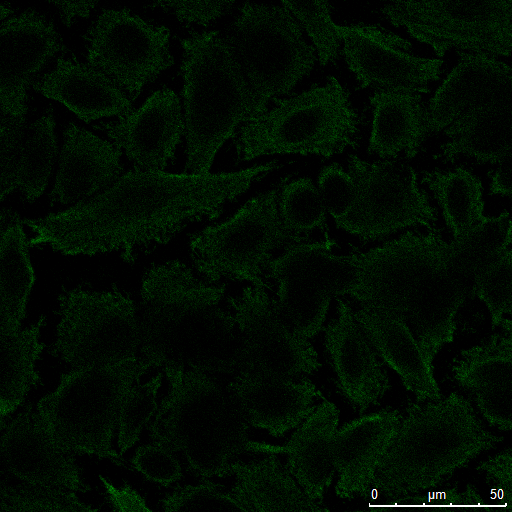

Supplement: Supplementary file 6 [file DataSheet_3.zip › Data Sheet 3/Fig4A/AC009948.5-LNCExperiment001_Series014_z0_ch01 (1).tif]

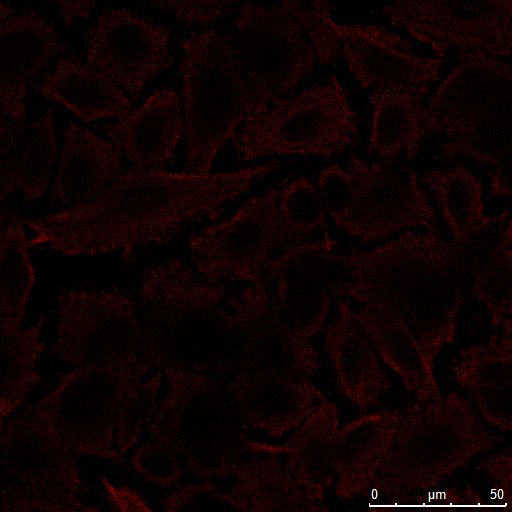

Supplement: Supplementary file 6 [file DataSheet_3.zip › Data Sheet 3/Fig4A/AC009948.5-LNCExperiment001_Series014_z0_ch01 (2).tif]

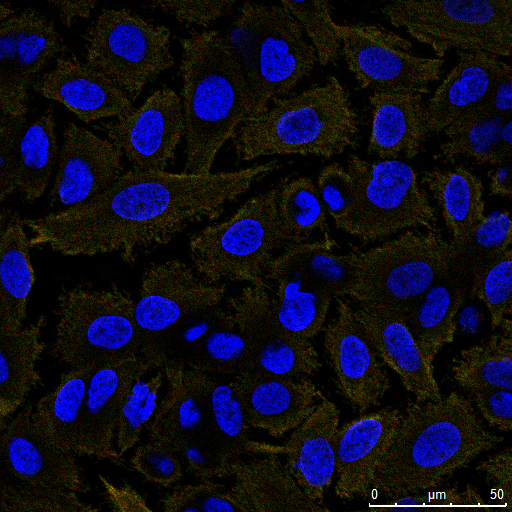

Supplement: Supplementary file 6 [file DataSheet_3.zip › Data Sheet 3/Fig4A/AC009948.5-LNCExperiment001_Series014_z0_ch01 (3).tif]

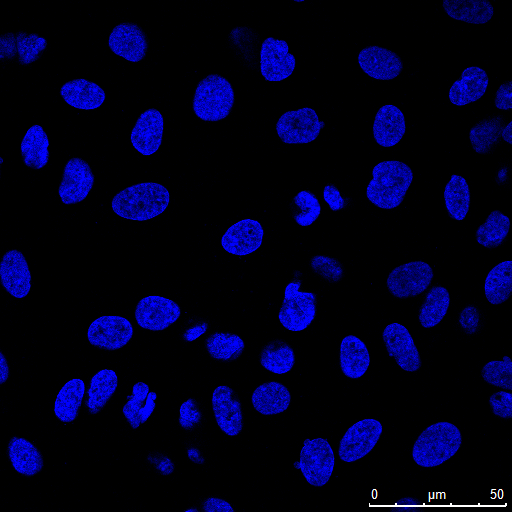

Supplement: Supplementary file 6 [file DataSheet_3.zip › Data Sheet 3/Fig4A/AC009948.5-LNCExperiment001_Series014_z0_ch01 (4).tif]

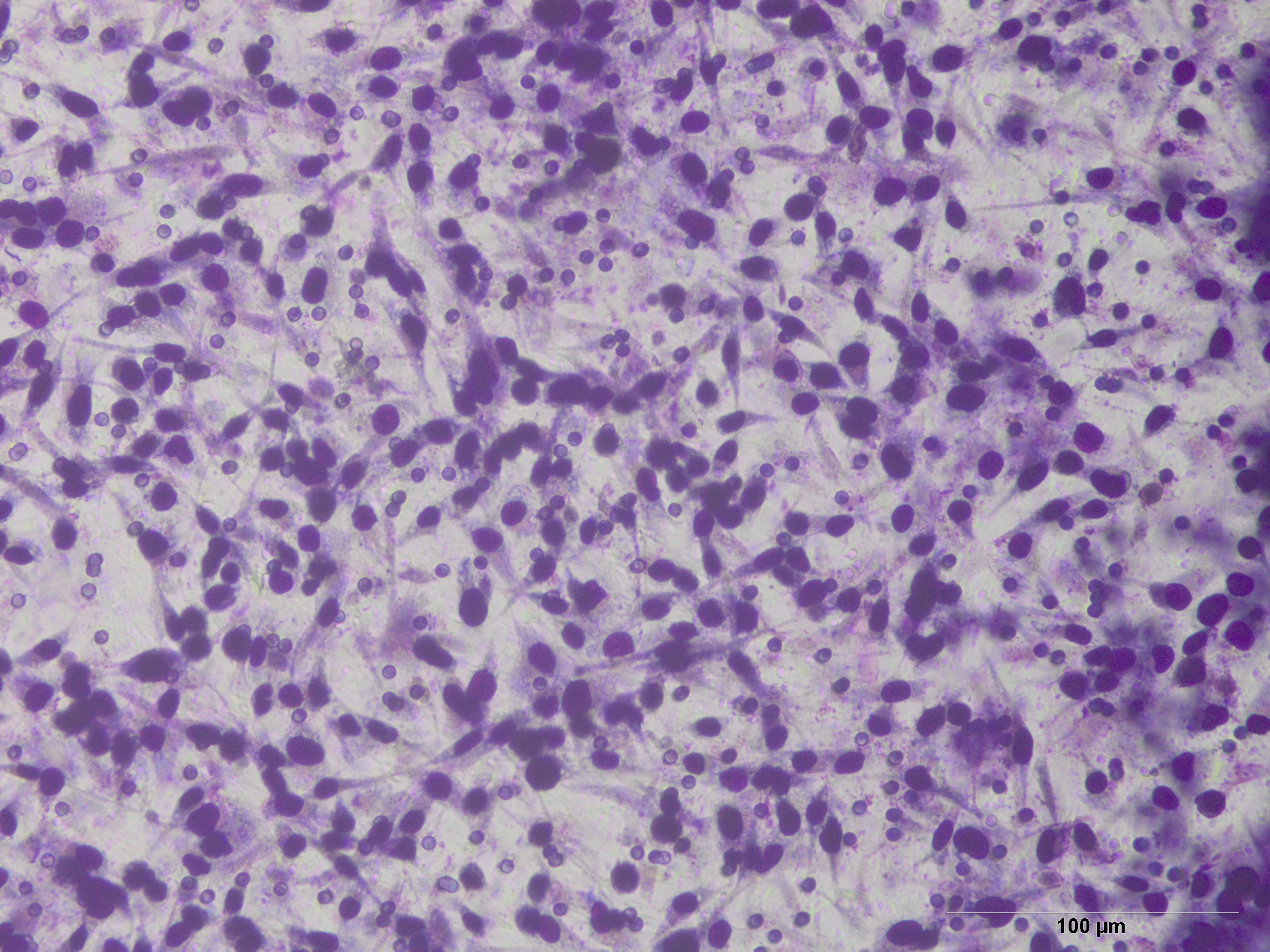

Supplement: Supplementary file 6 [file DataSheet_3.zip › Data Sheet 3/Fig4C/1-AC009948,5-sh-con-M.jpg]

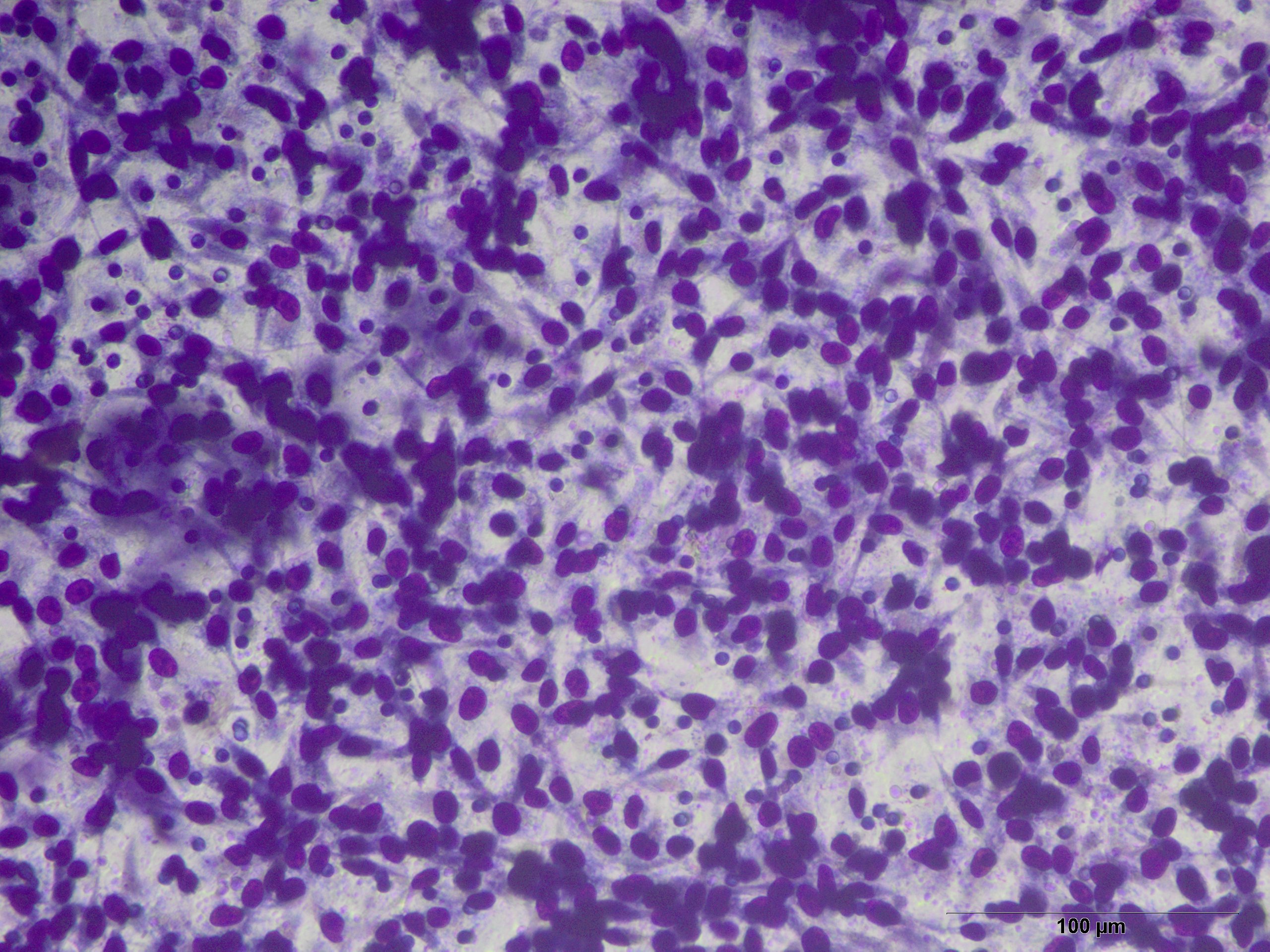

Supplement: Supplementary file 6 [file DataSheet_3.zip › Data Sheet 3/Fig4C/1-AC009948.5-con-M.jpg]

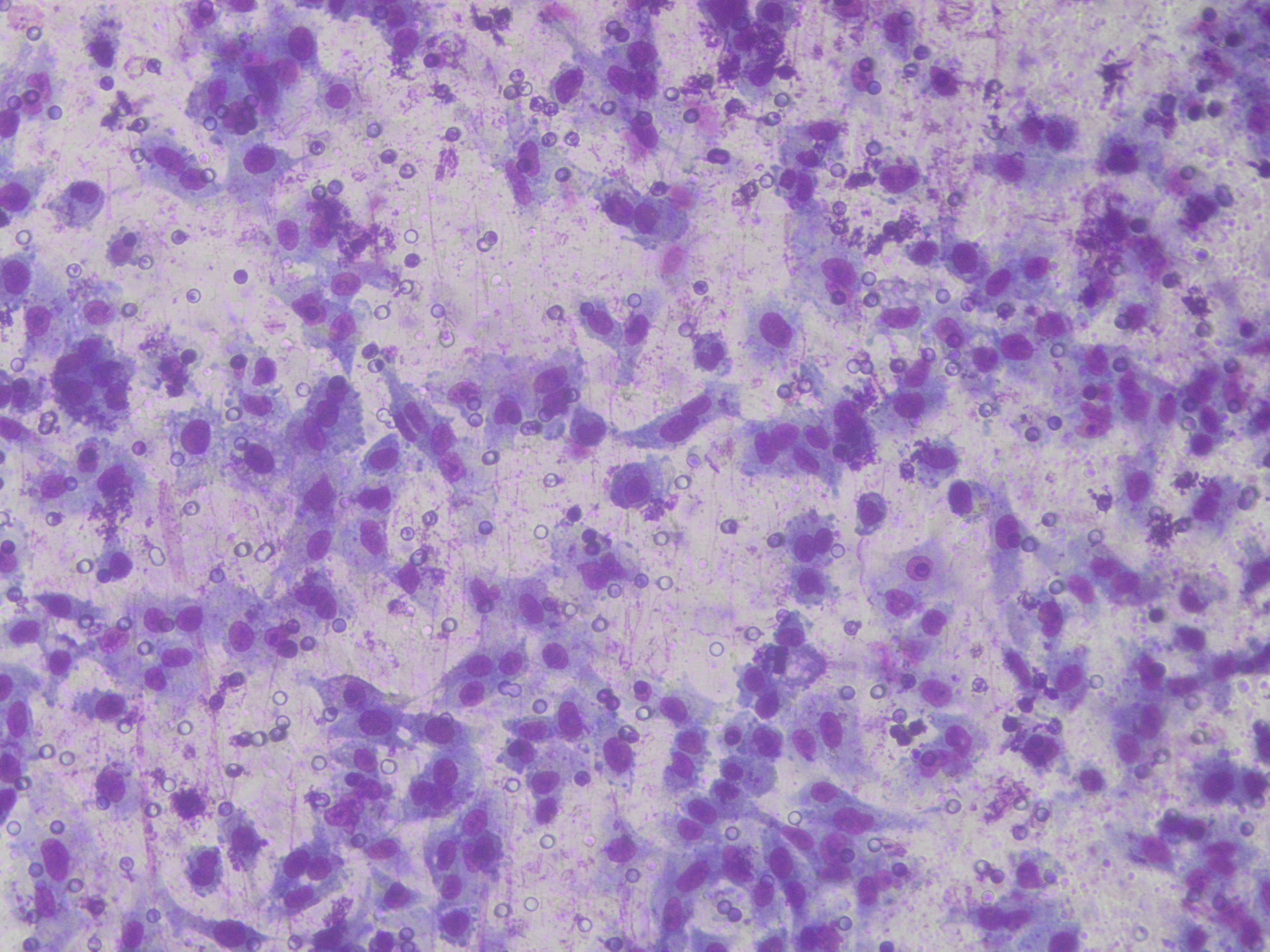

Supplement: Supplementary file 6 [file DataSheet_3.zip › Data Sheet 3/Fig4C/1-AC009948.5-COTRANS-INVASION-.jpg]

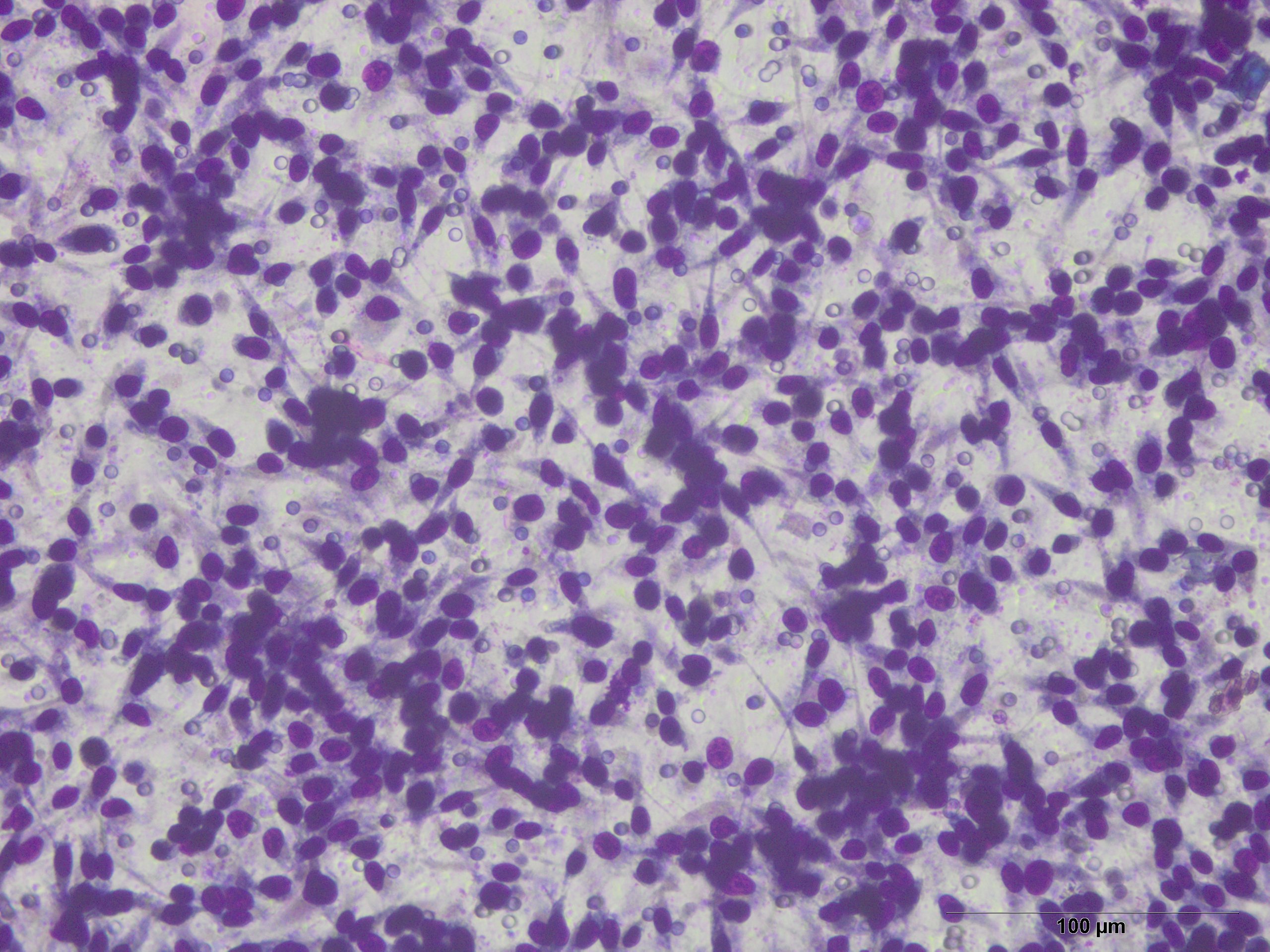

Supplement: Supplementary file 6 [file DataSheet_3.zip › Data Sheet 3/Fig4C/1-AC009948.5-COTRANS-M.jpg]

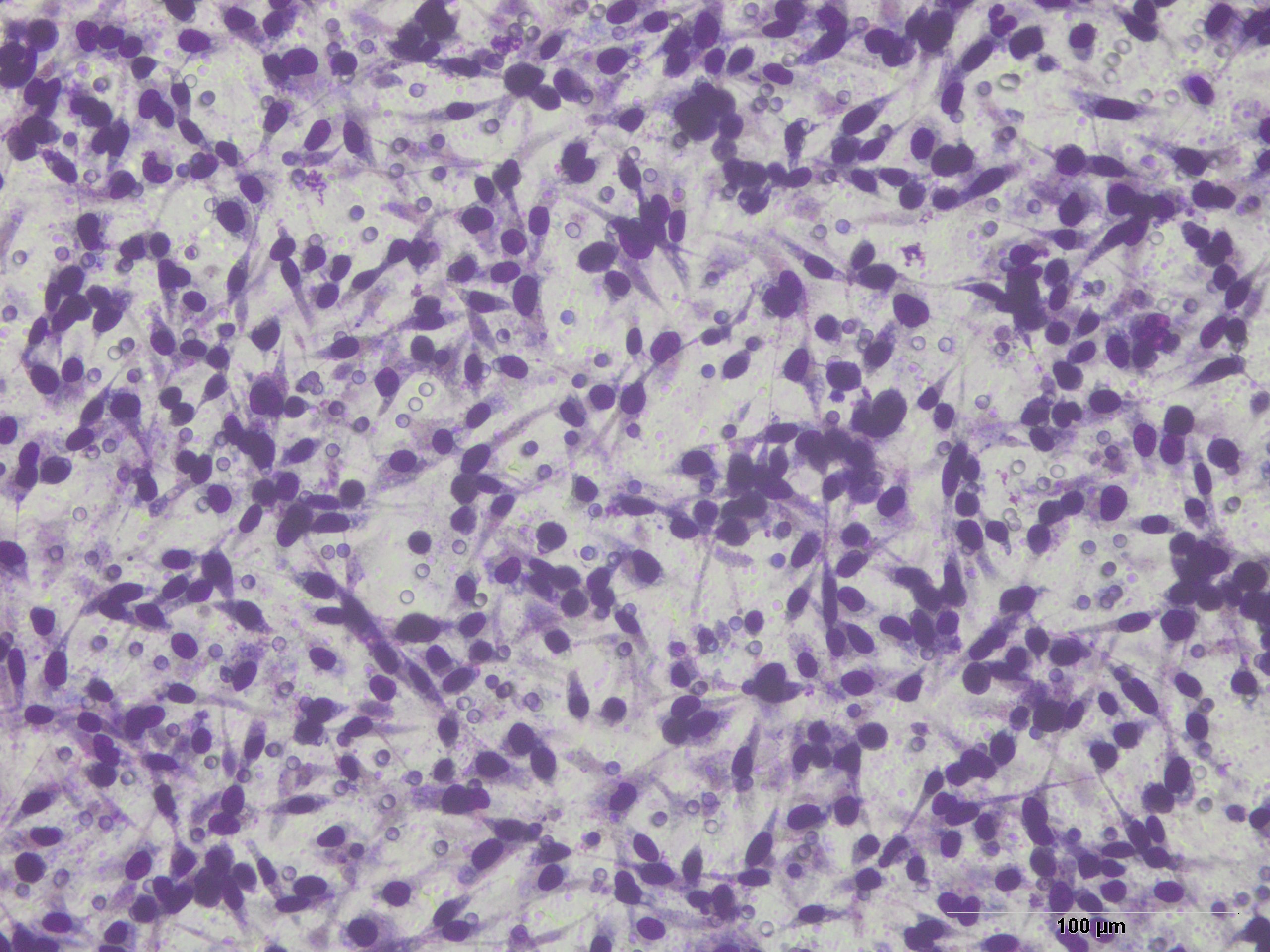

Supplement: Supplementary file 6 [file DataSheet_3.zip › Data Sheet 3/Fig4C/1-AC009948.5-OVER-186-M.jpg]

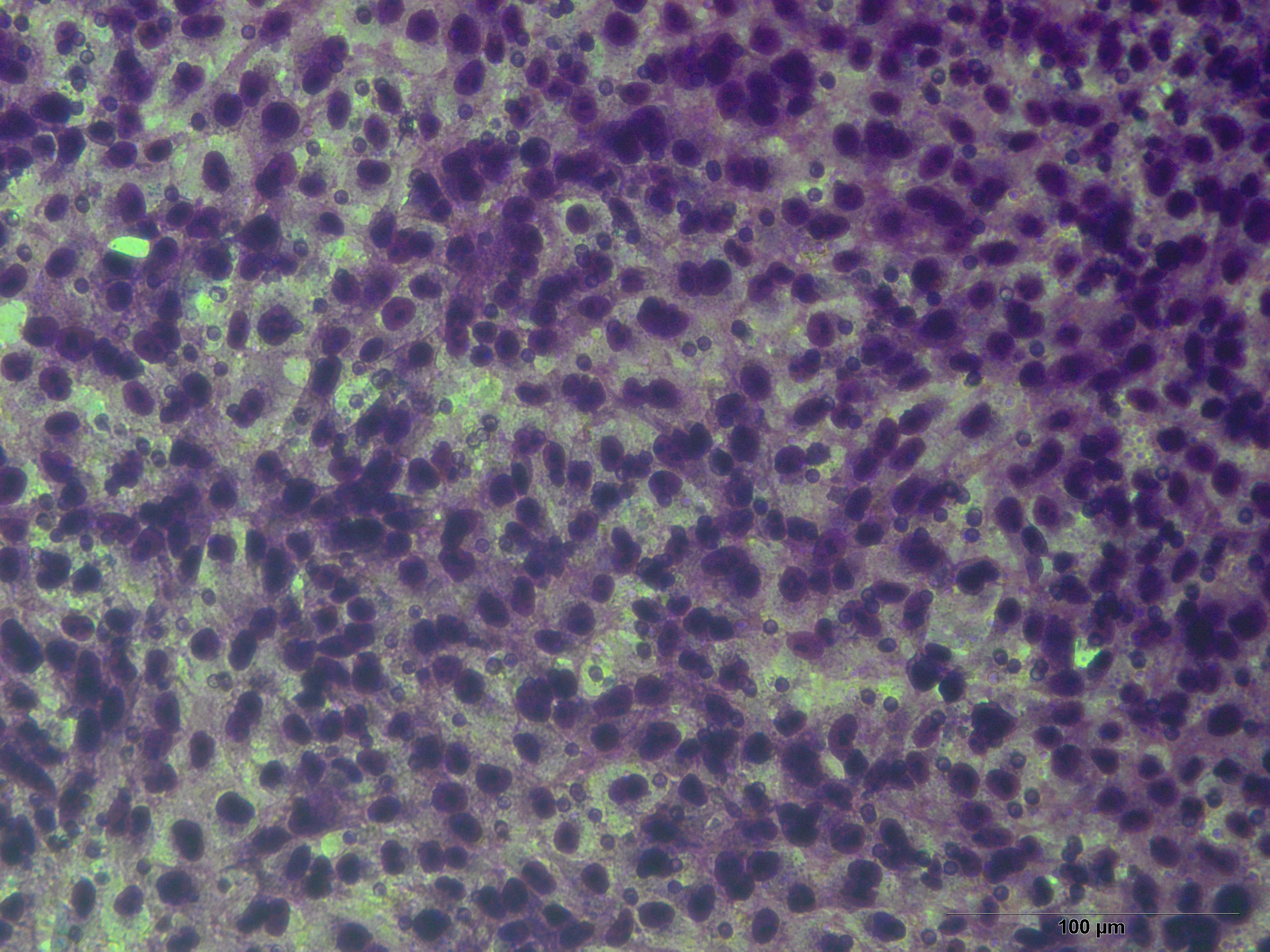

Supplement: Supplementary file 6 [file DataSheet_3.zip › Data Sheet 3/Fig4C/1-AC009948.5-sh-miR-186-M.jpg]

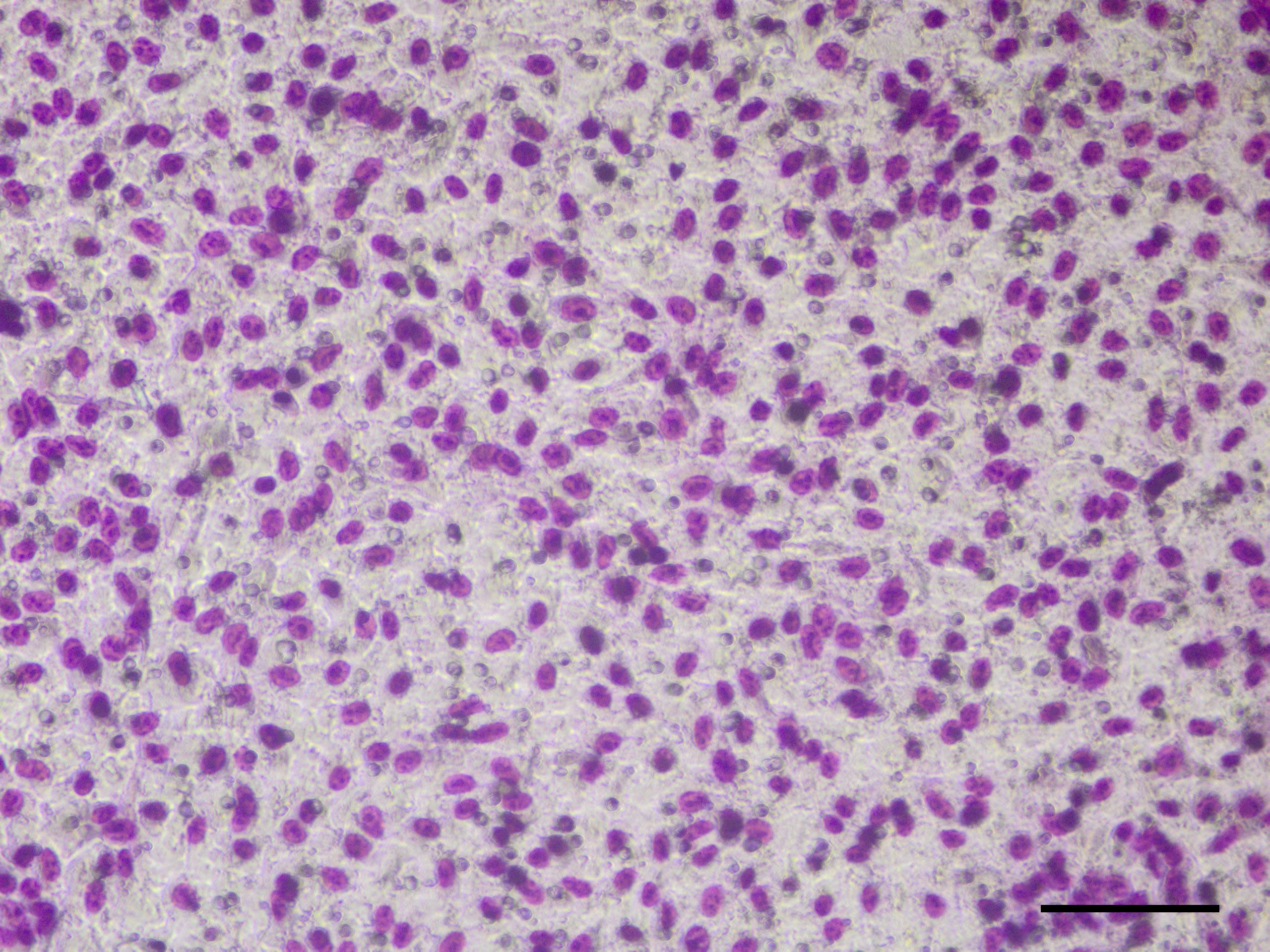

Supplement: Supplementary file 6 [file DataSheet_3.zip › Data Sheet 3/Fig4C/1-con-INVASION.jpg]

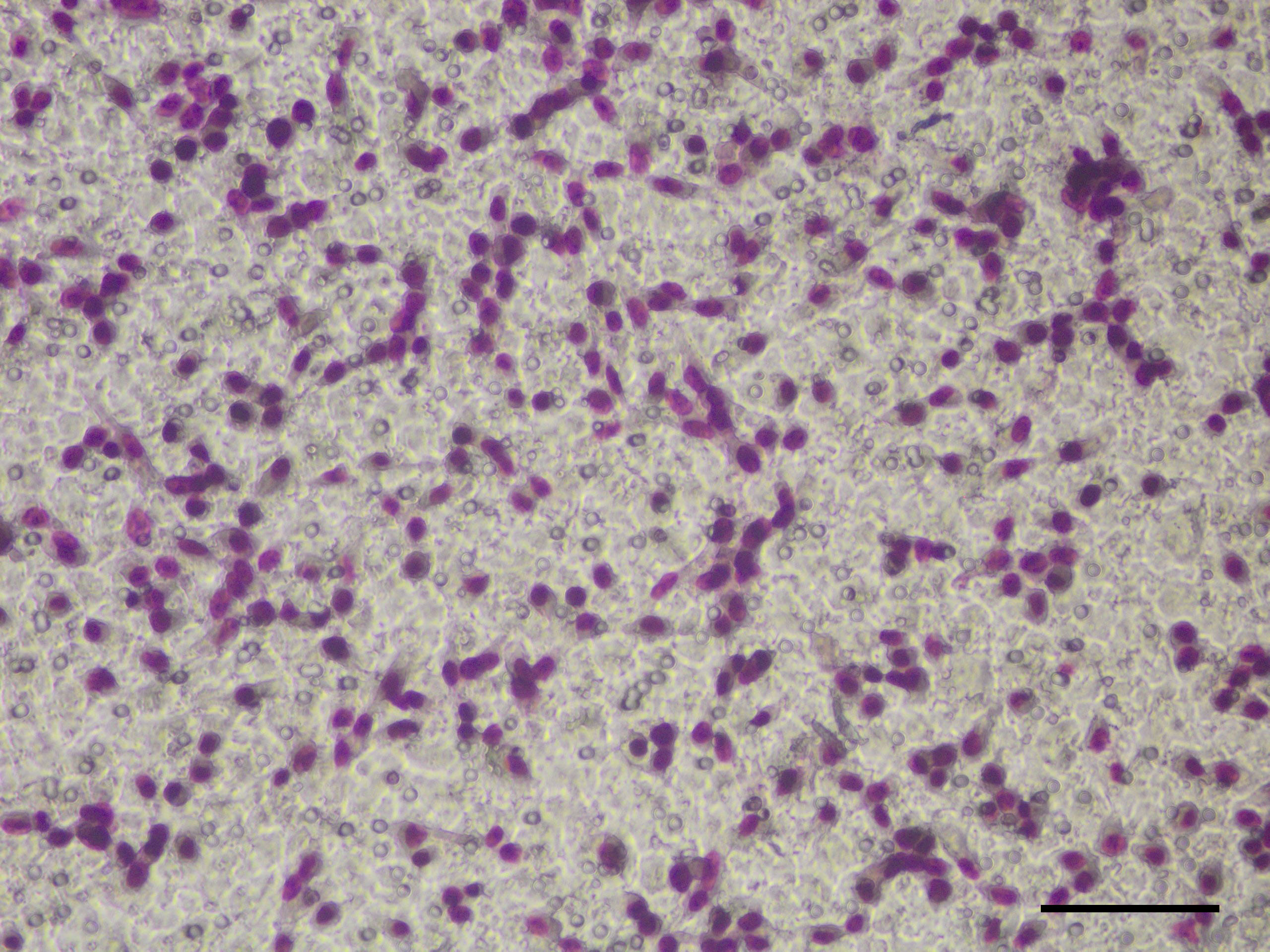

Supplement: Supplementary file 6 [file DataSheet_3.zip › Data Sheet 3/Fig4C/1-over-miR-186-5p-INVASION.jpg]

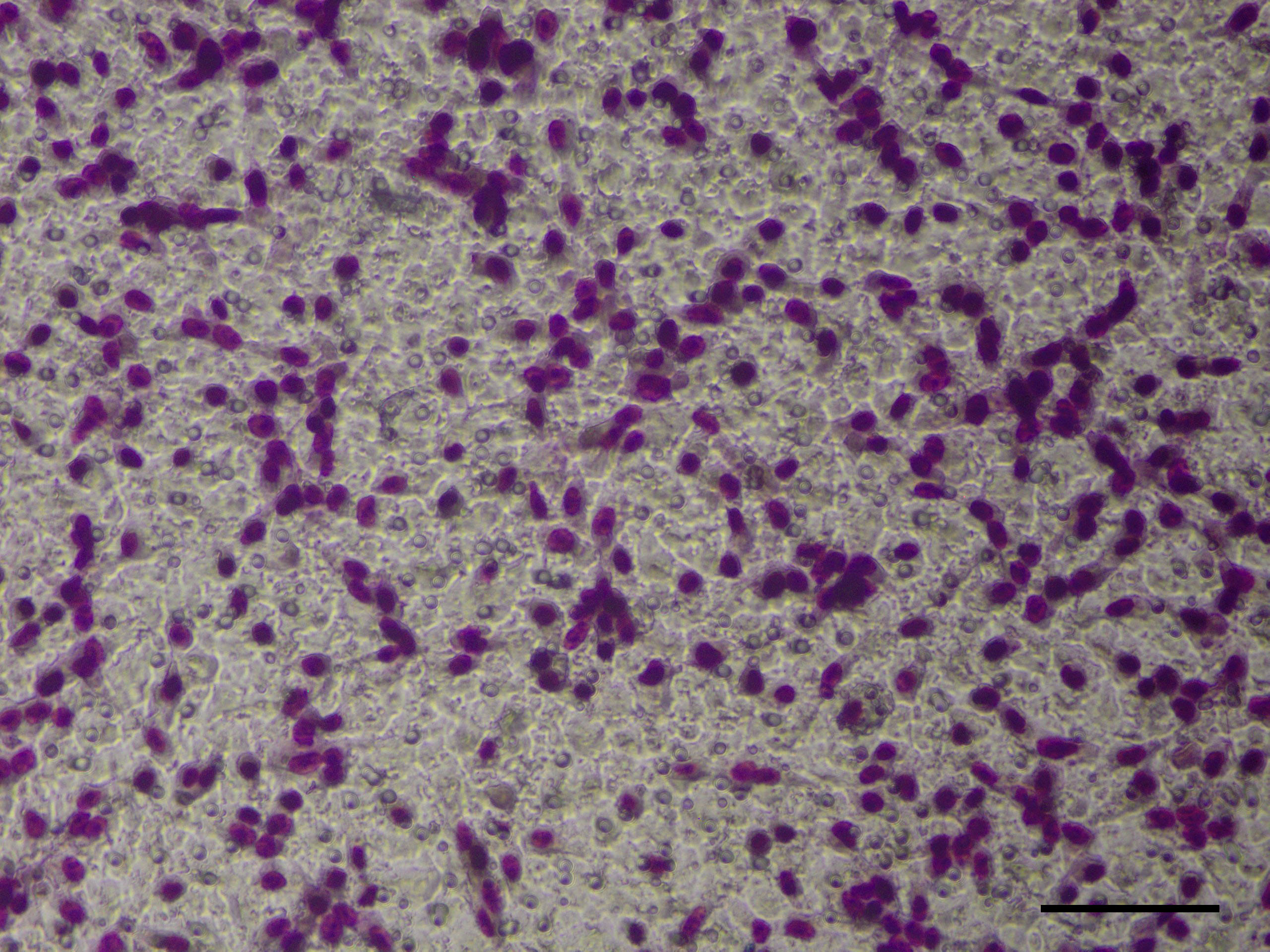

Supplement: Supplementary file 6 [file DataSheet_3.zip › Data Sheet 3/Fig4C/1-sh-con-INVASION.jpg]

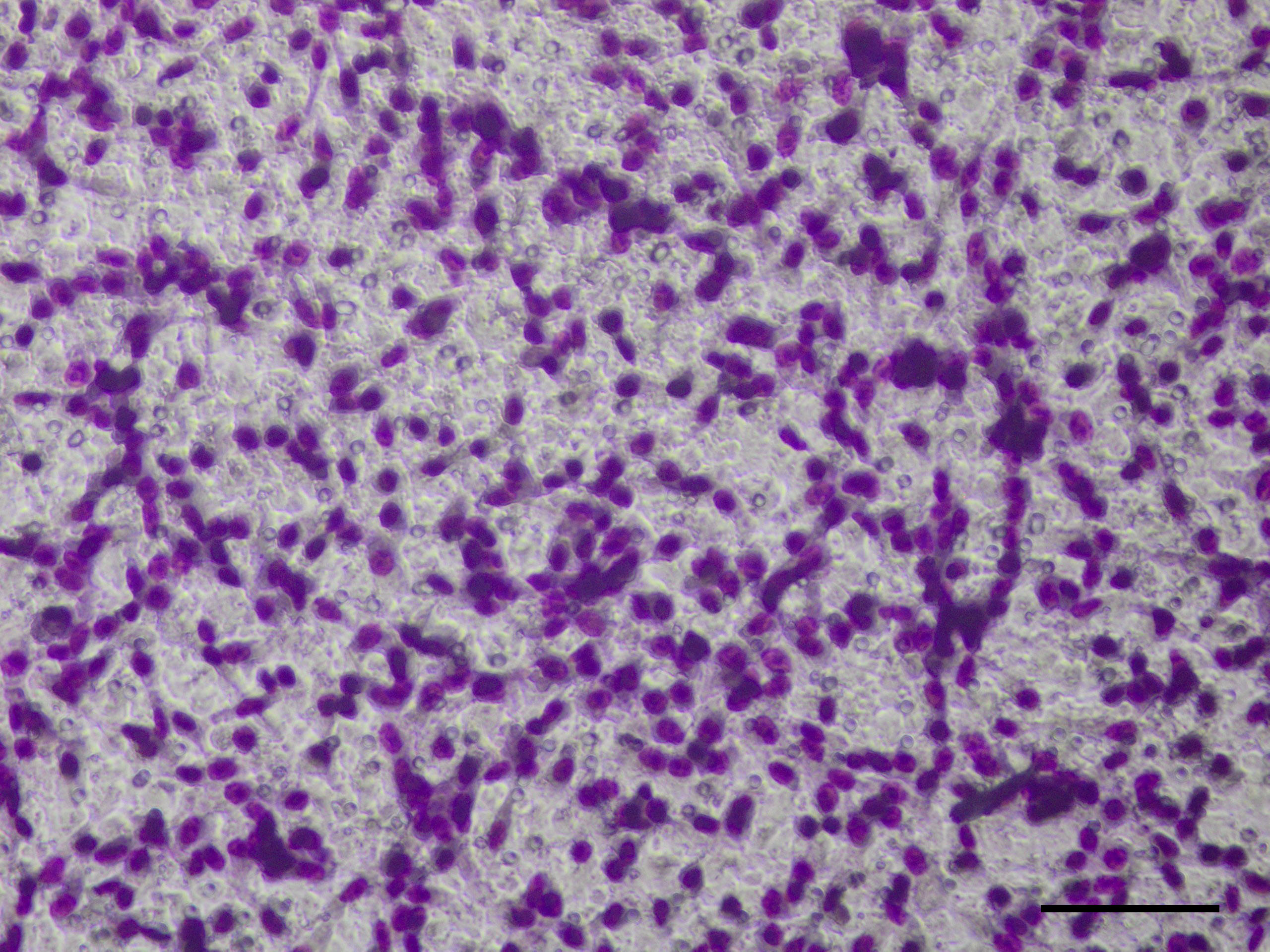

Supplement: Supplementary file 6 [file DataSheet_3.zip › Data Sheet 3/Fig4C/1-sh-miR-186-5p-INVASION.jpg]

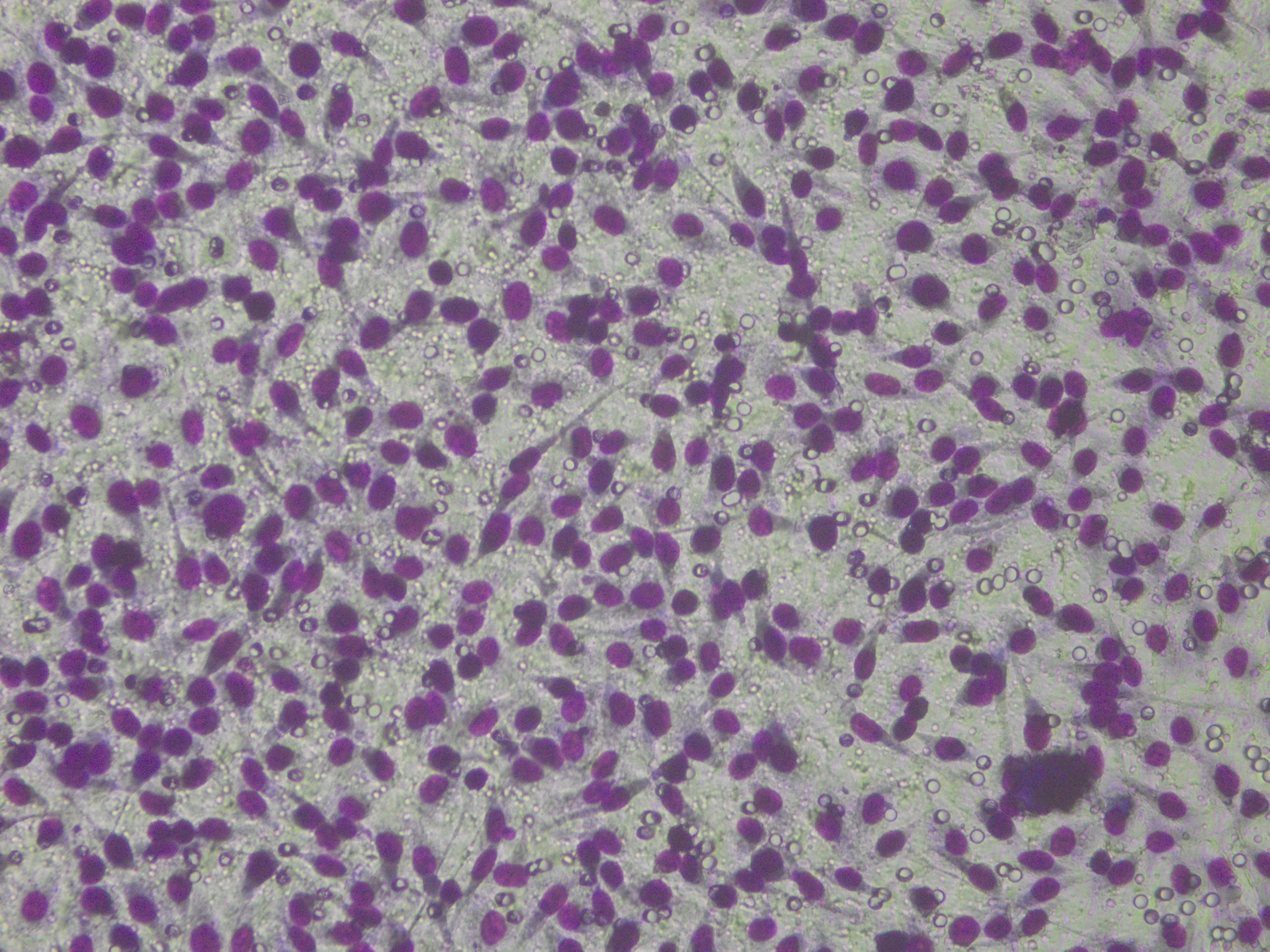

Supplement: Supplementary file 6 [file DataSheet_3.zip › Data Sheet 3/Fig4C/2-AC009948,5-sh-con-M.jpg]

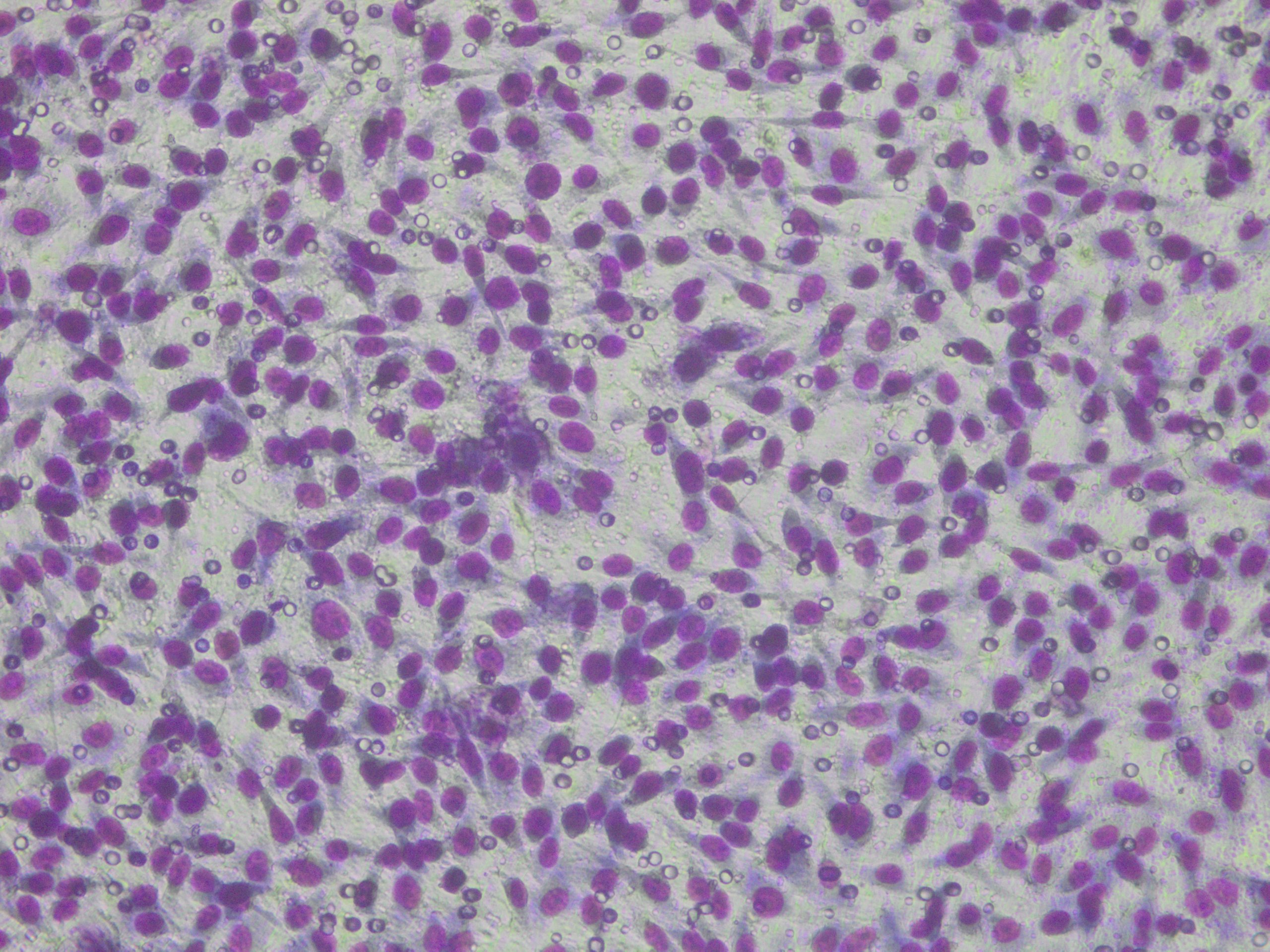

Supplement: Supplementary file 6 [file DataSheet_3.zip › Data Sheet 3/Fig4C/2-AC009948.5-con-M.jpg]

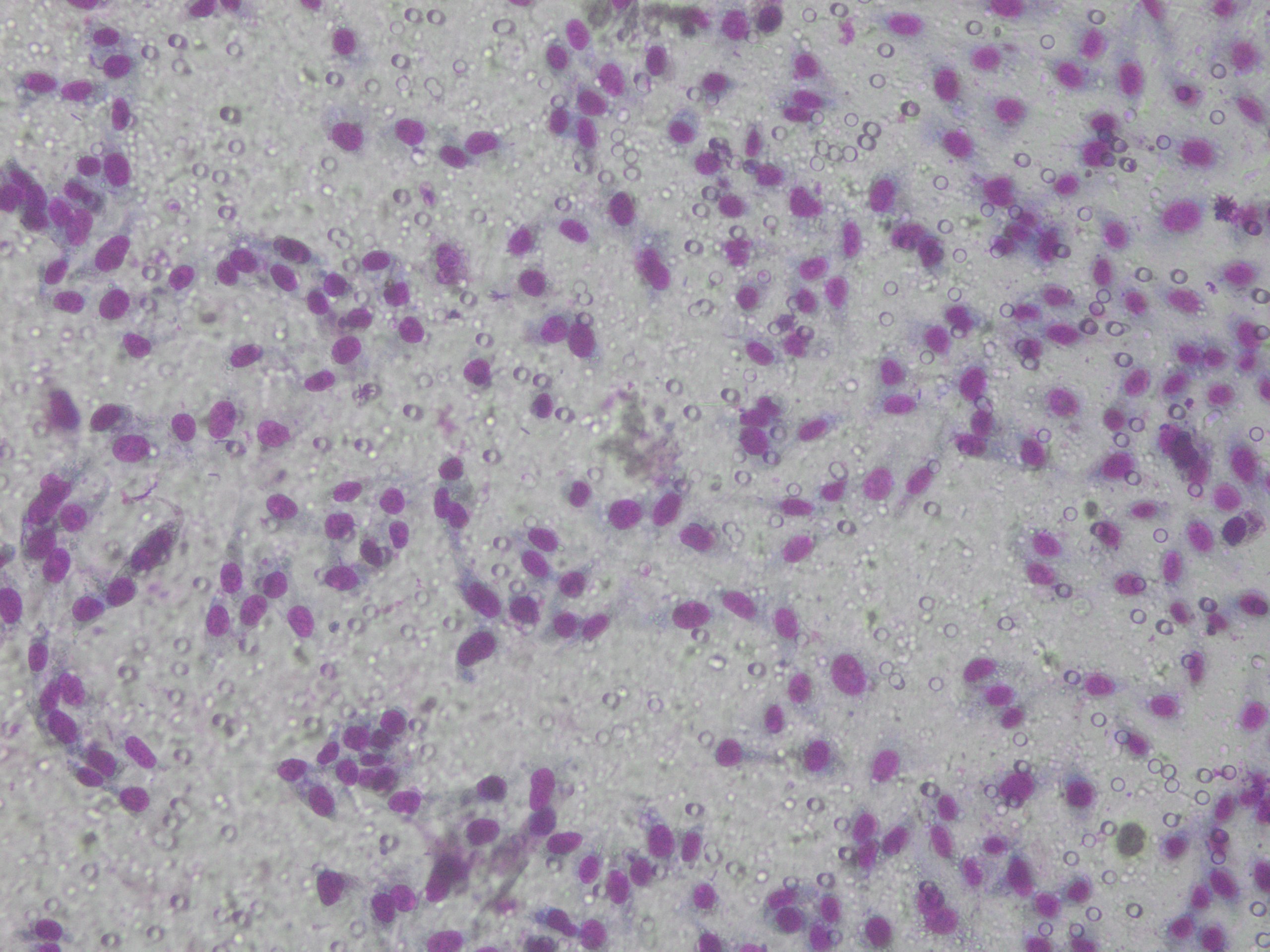

Supplement: Supplementary file 6 [file DataSheet_3.zip › Data Sheet 3/Fig4C/2-AC009948.5-COTRANS-INVASION-.jpg]

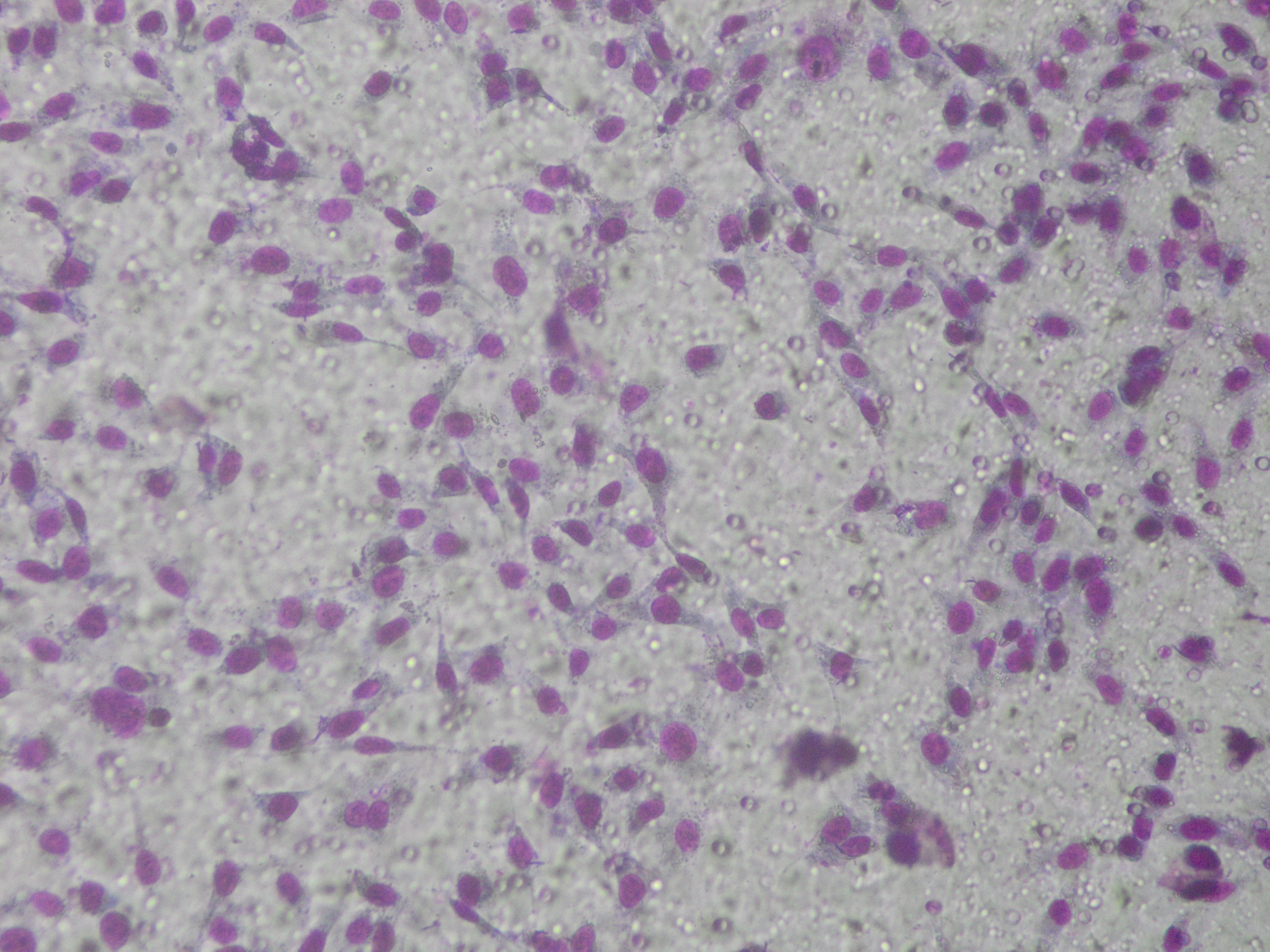

Supplement: Supplementary file 6 [file DataSheet_3.zip › Data Sheet 3/Fig4C/2-AC009948.5-OVER-186-M.jpg]

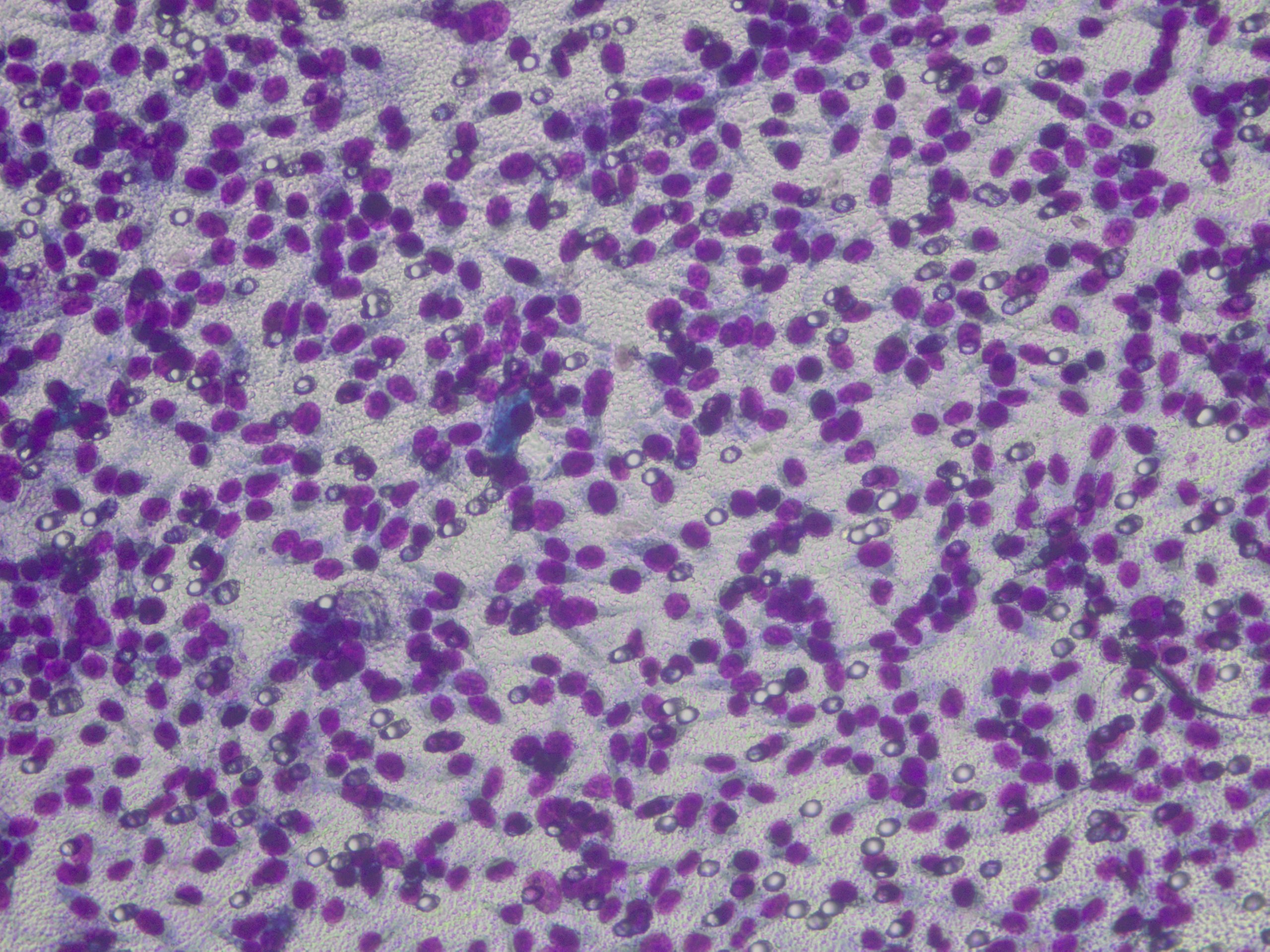

Supplement: Supplementary file 6 [file DataSheet_3.zip › Data Sheet 3/Fig4C/2-AC009948.5-sh-miR-186-M.jpg]

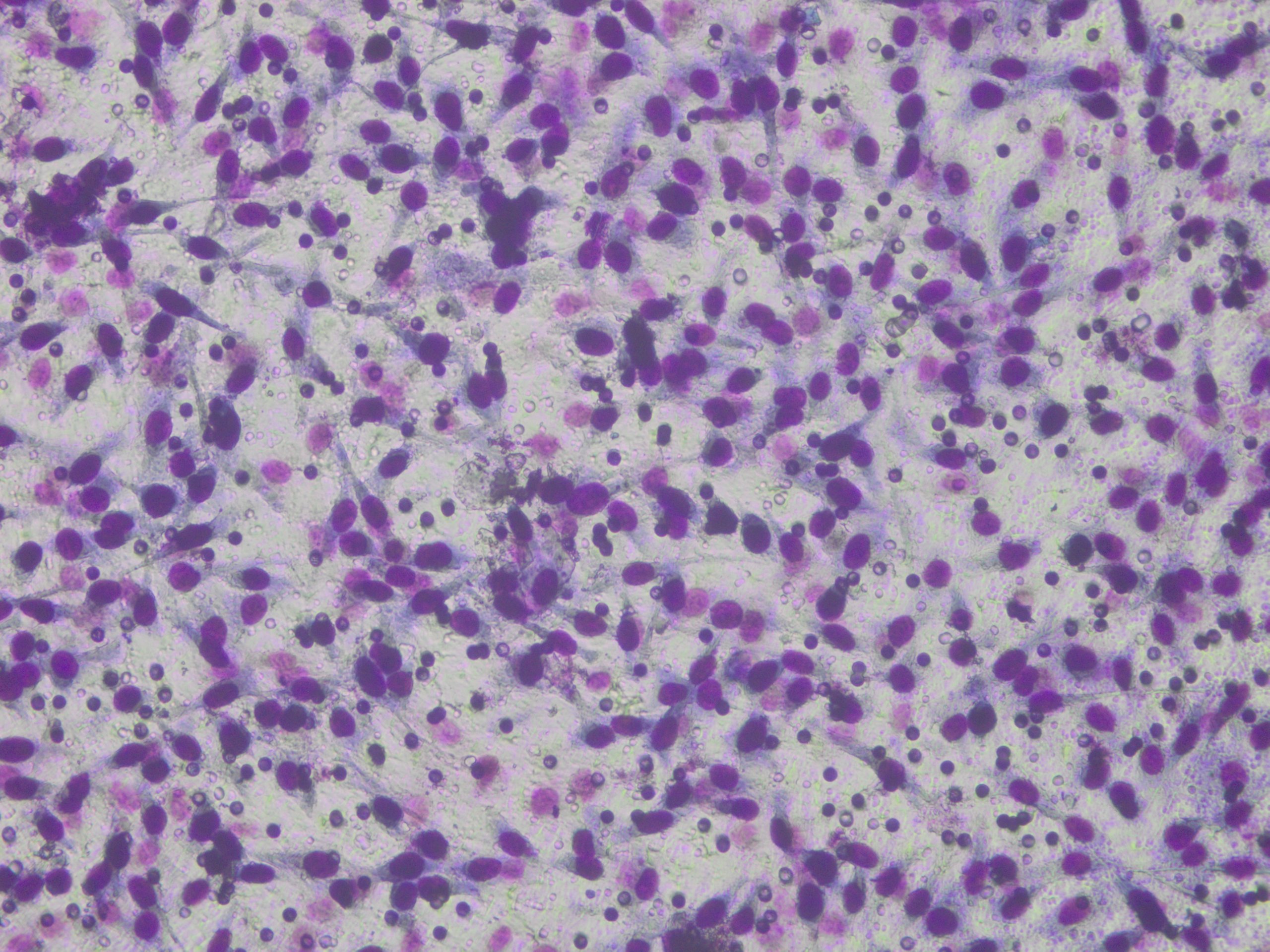

Supplement: Supplementary file 6 [file DataSheet_3.zip › Data Sheet 3/Fig4C/2-AC009958.5-COTRANS-M.jpg]

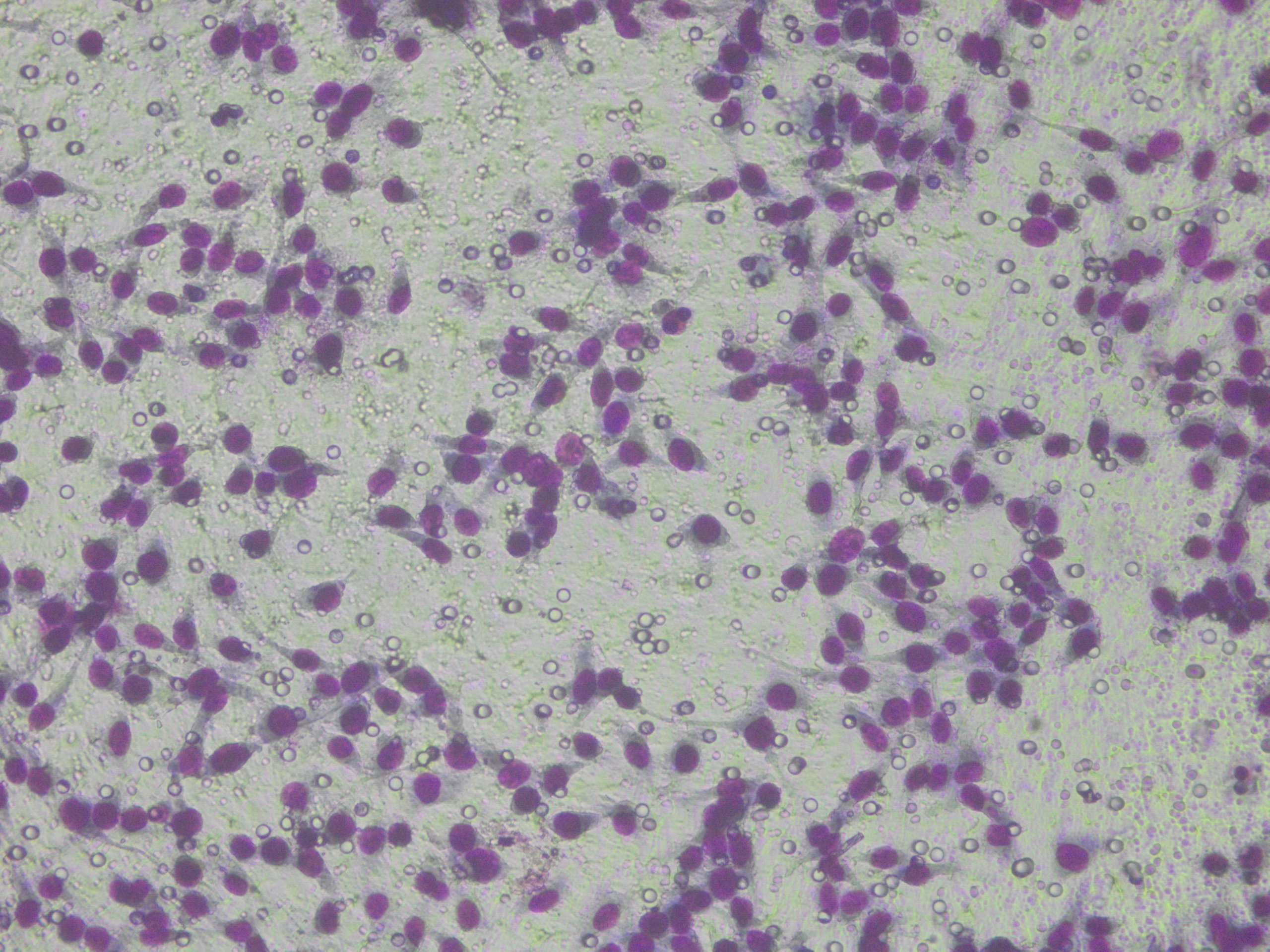

Supplement: Supplementary file 6 [file DataSheet_3.zip › Data Sheet 3/Fig4C/2-con-INVASION.jpg]

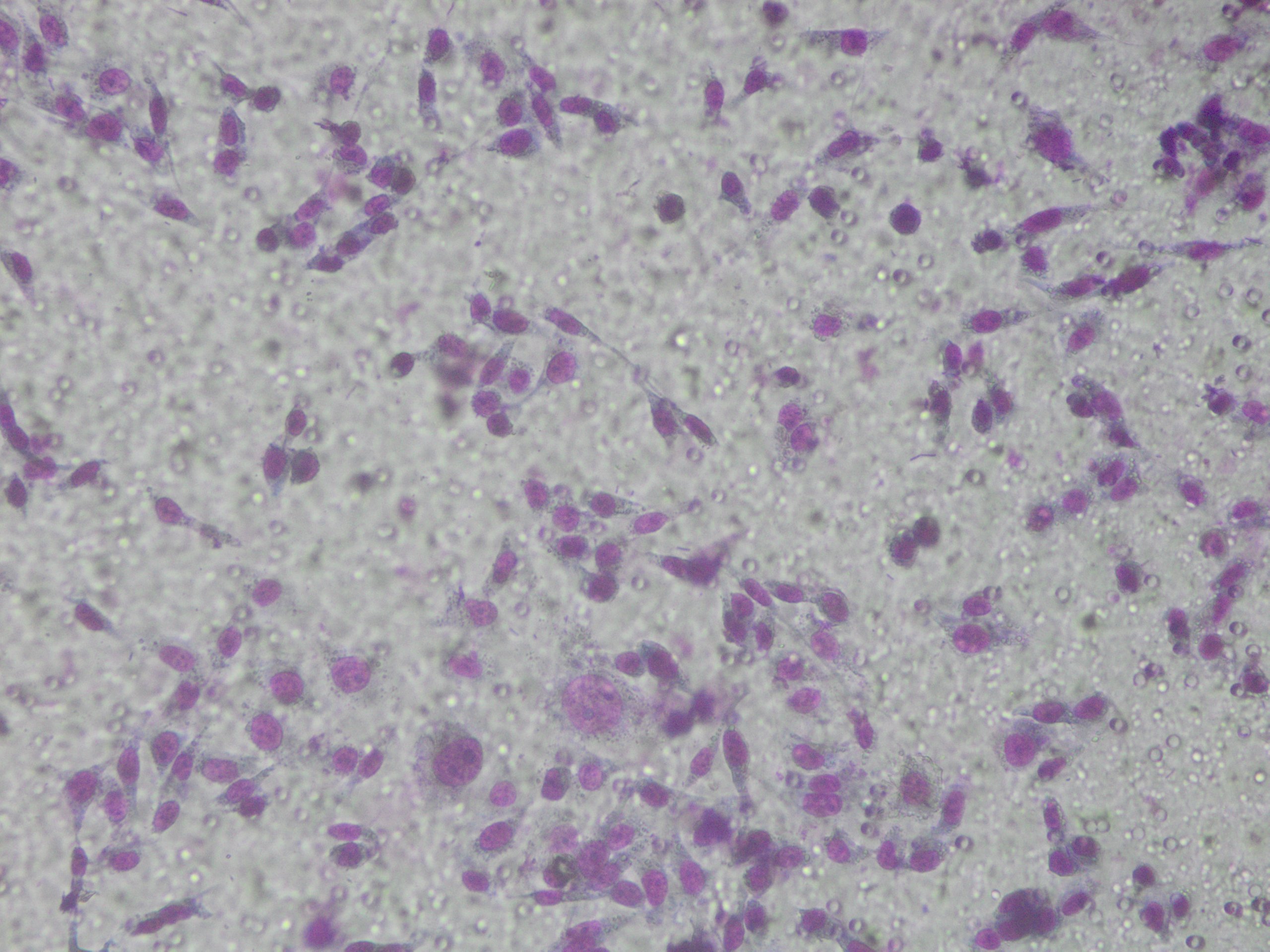

Supplement: Supplementary file 6 [file DataSheet_3.zip › Data Sheet 3/Fig4C/2-over-miR-186-5p-INVASION.jpg]

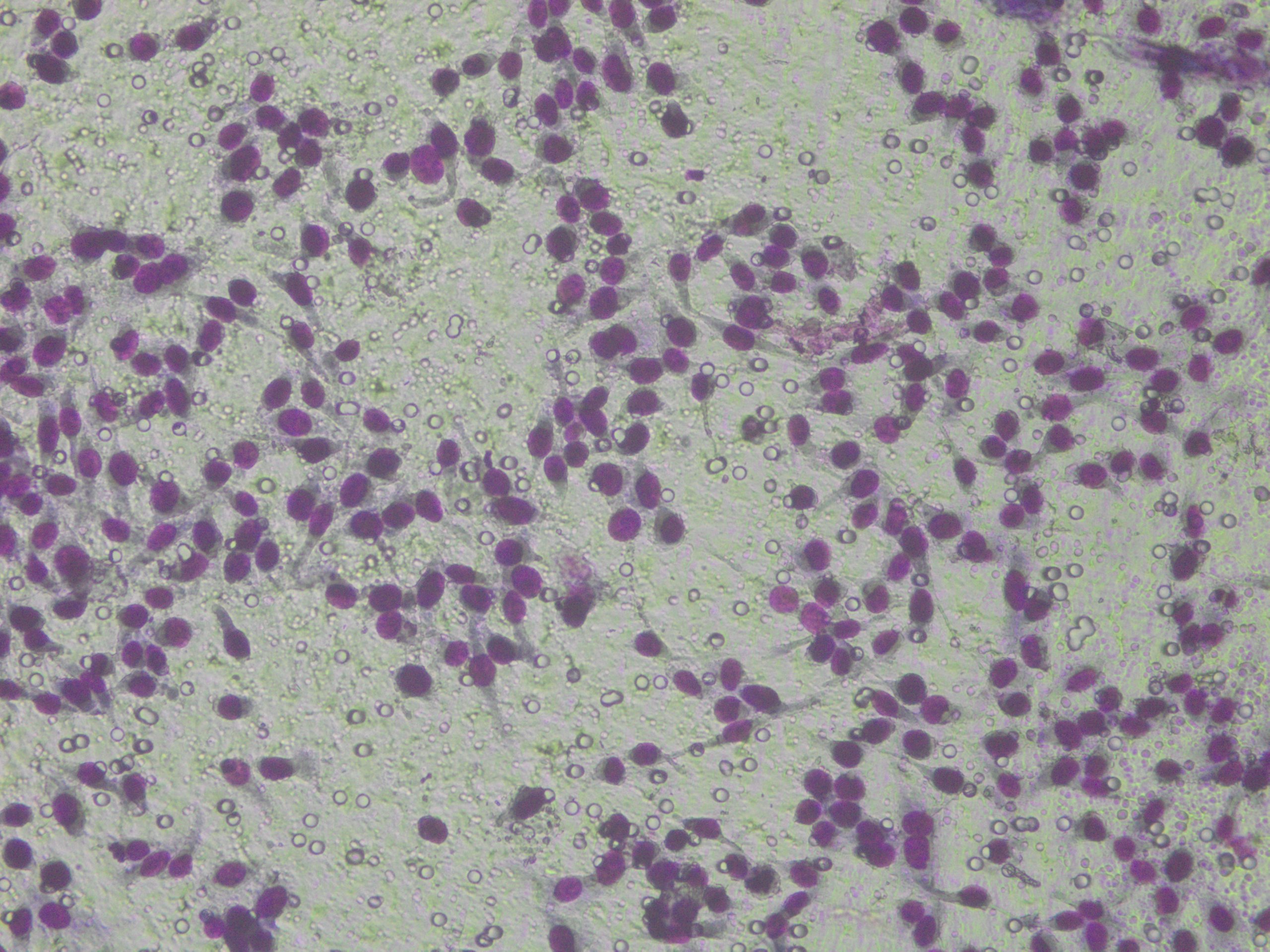

Supplement: Supplementary file 6 [file DataSheet_3.zip › Data Sheet 3/Fig4C/2-sh-con-INVASION.jpg]

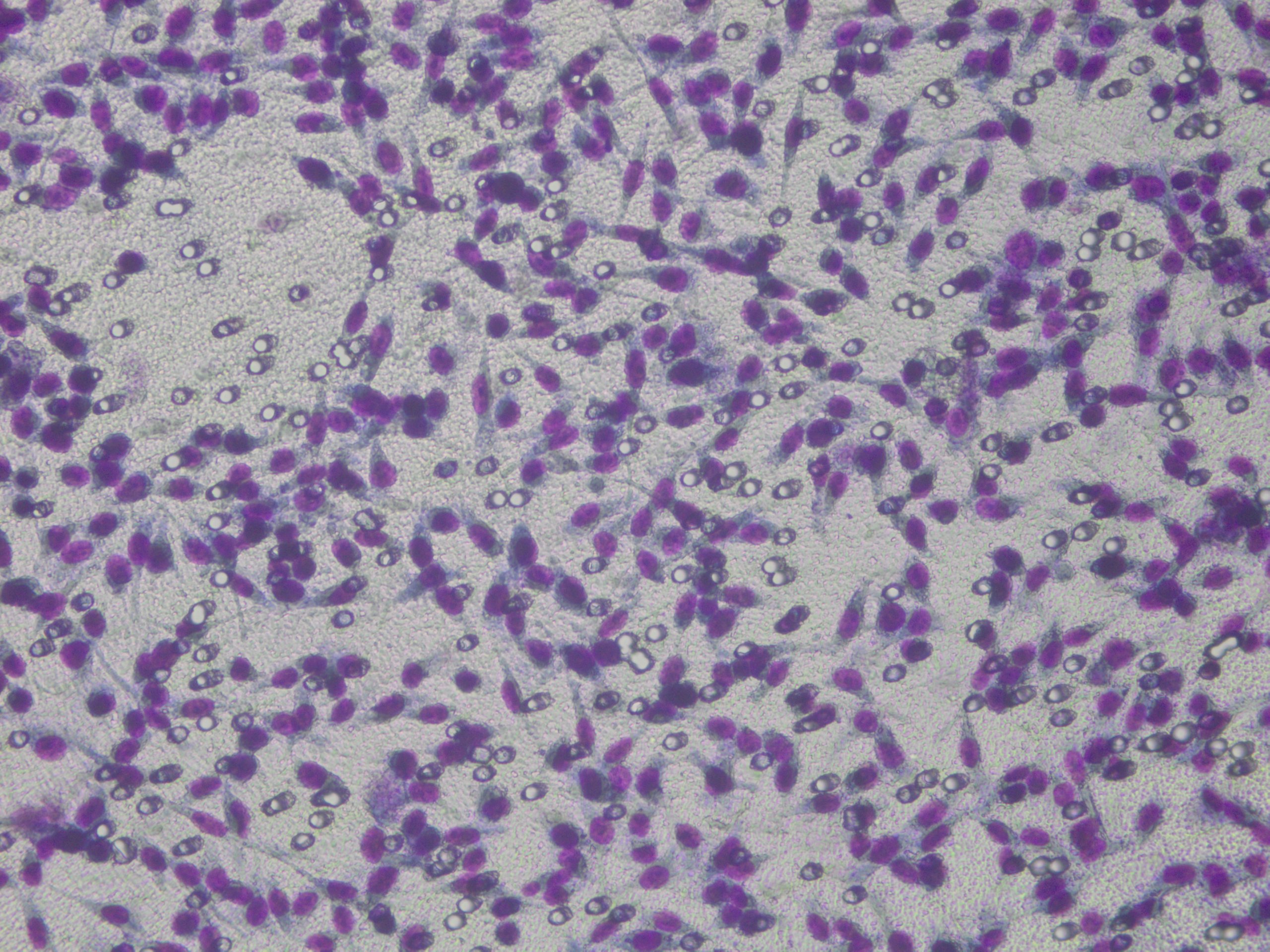

Supplement: Supplementary file 6 [file DataSheet_3.zip › Data Sheet 3/Fig4C/2-sh-miR-186-5p-INVASION.jpg]

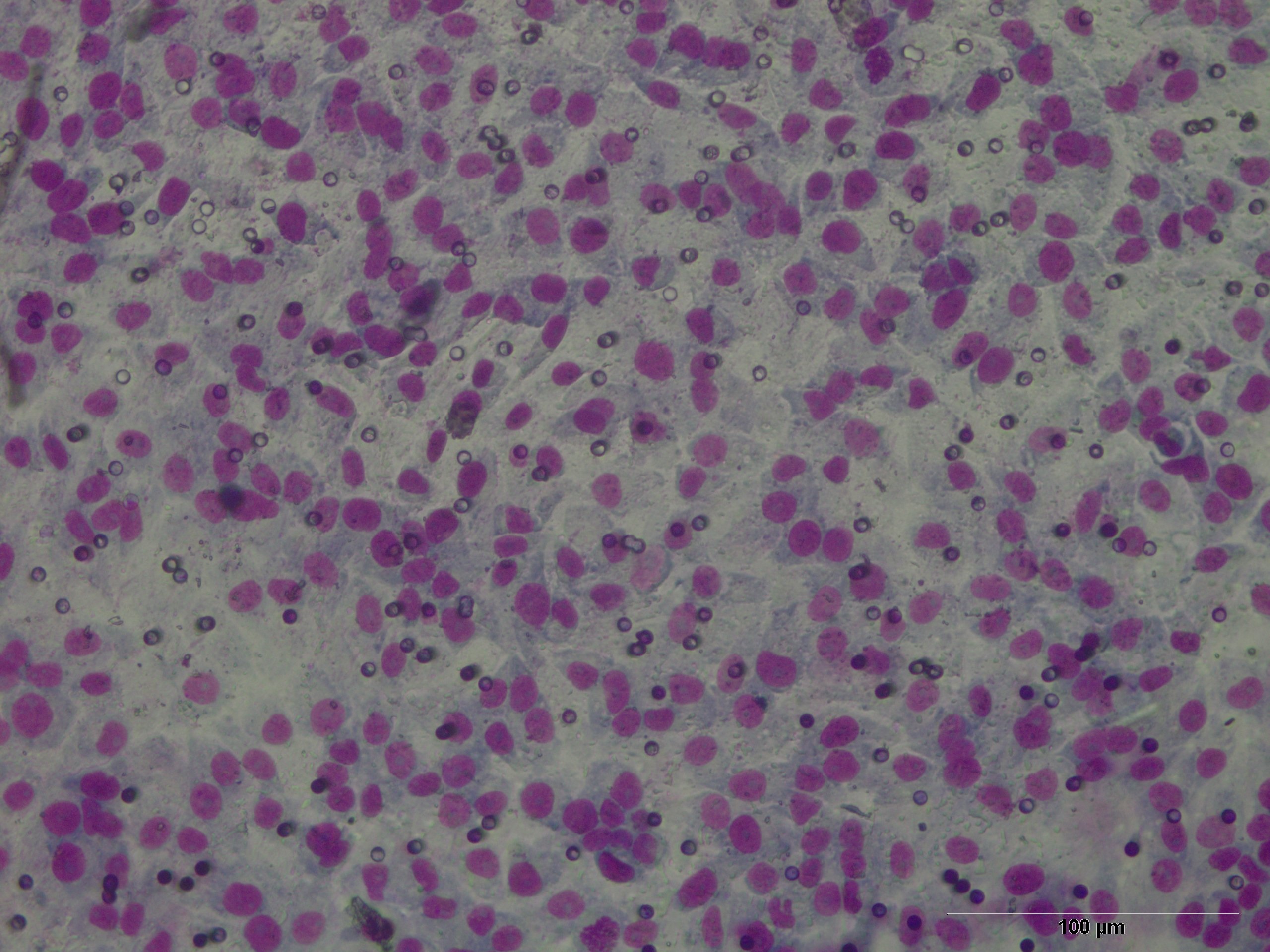

Supplement: Supplementary file 6 [file DataSheet_3.zip › Data Sheet 3/Fig4C/3-AC009948,5-sh-con-M.jpg]

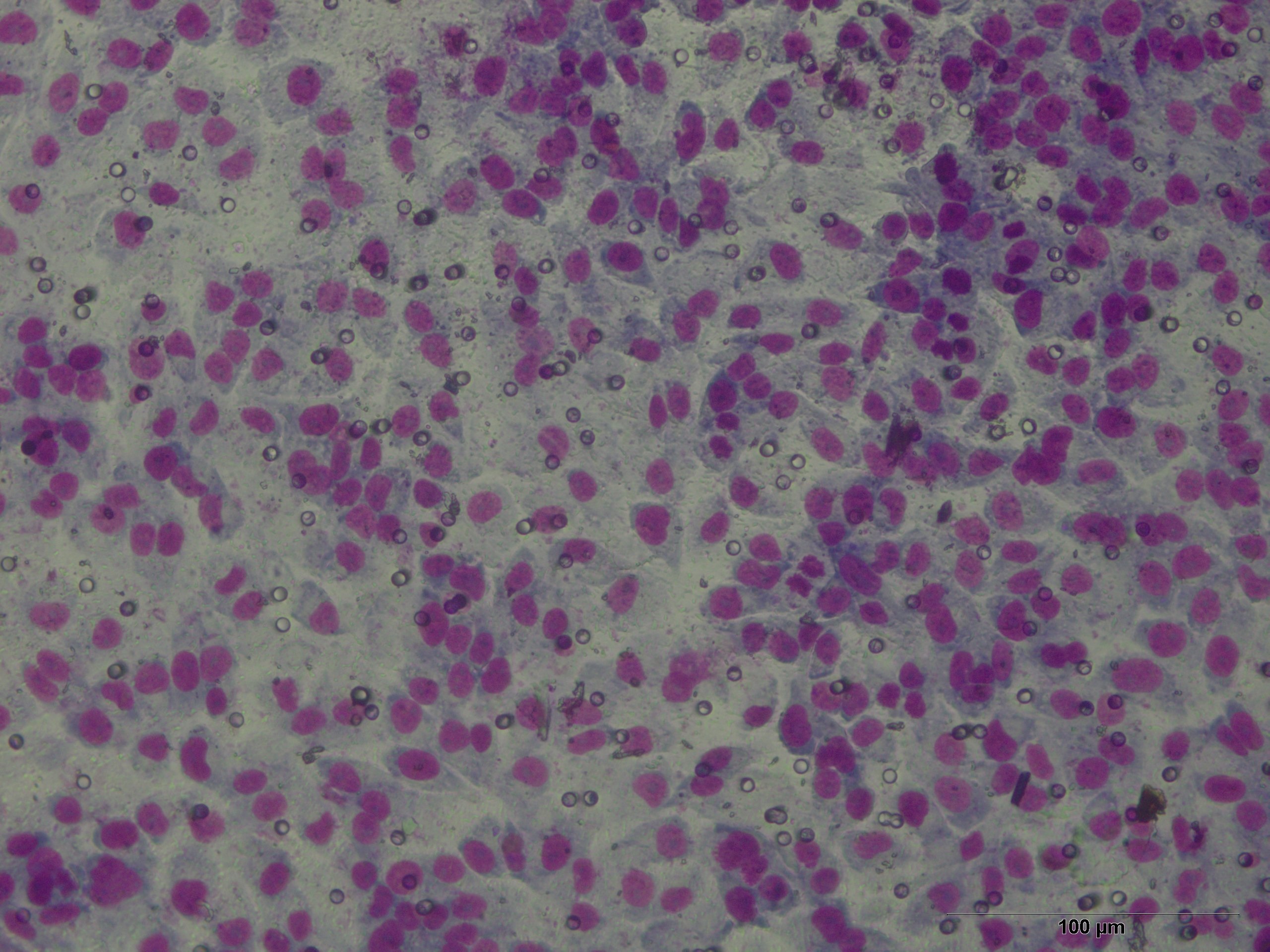

Supplement: Supplementary file 6 [file DataSheet_3.zip › Data Sheet 3/Fig4C/3-AC009948.5-con-M.jpg]

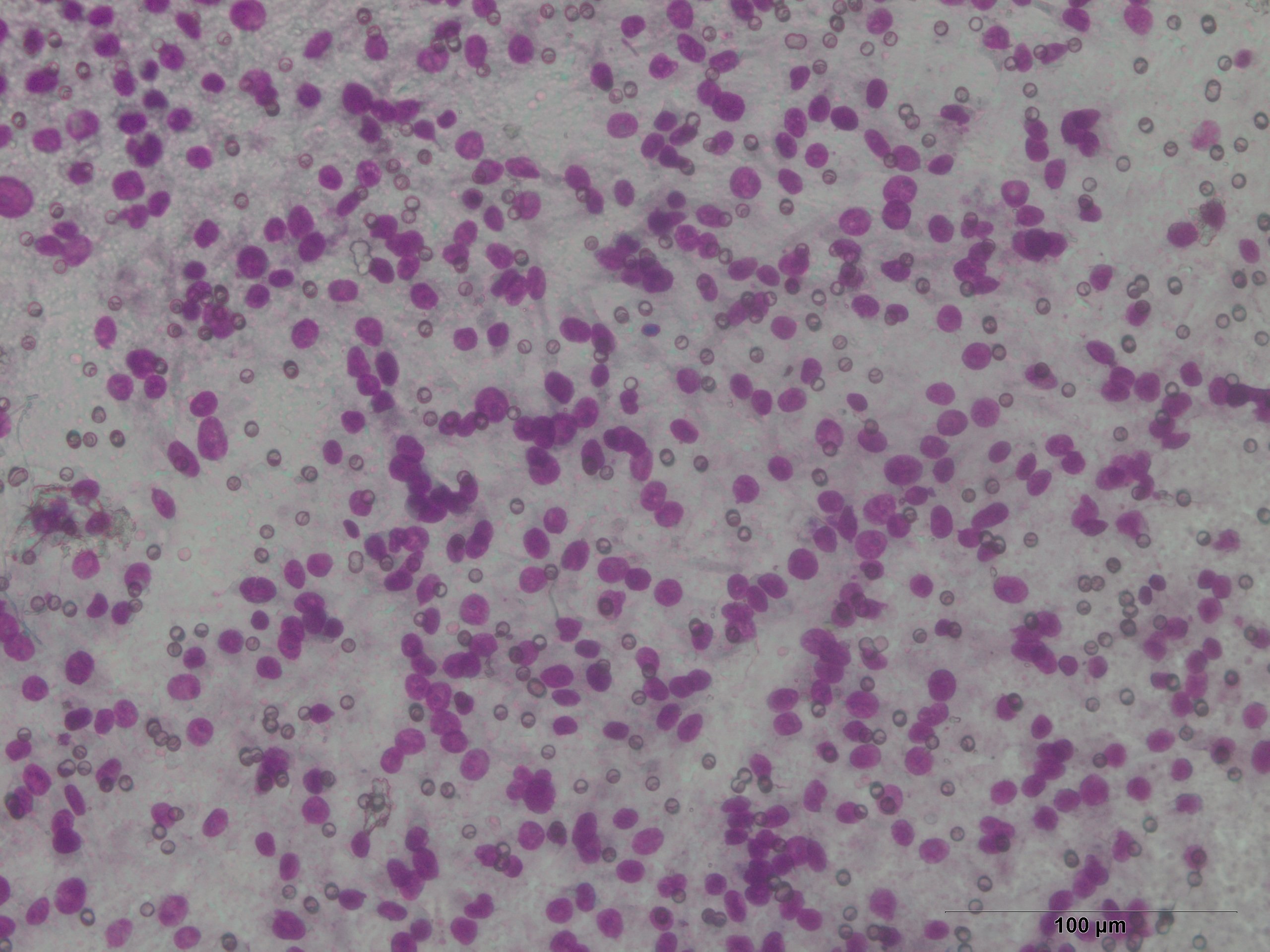

Supplement: Supplementary file 6 [file DataSheet_3.zip › Data Sheet 3/Fig4C/3-AC009948.5-COTRANS-INVASION-.jpg]

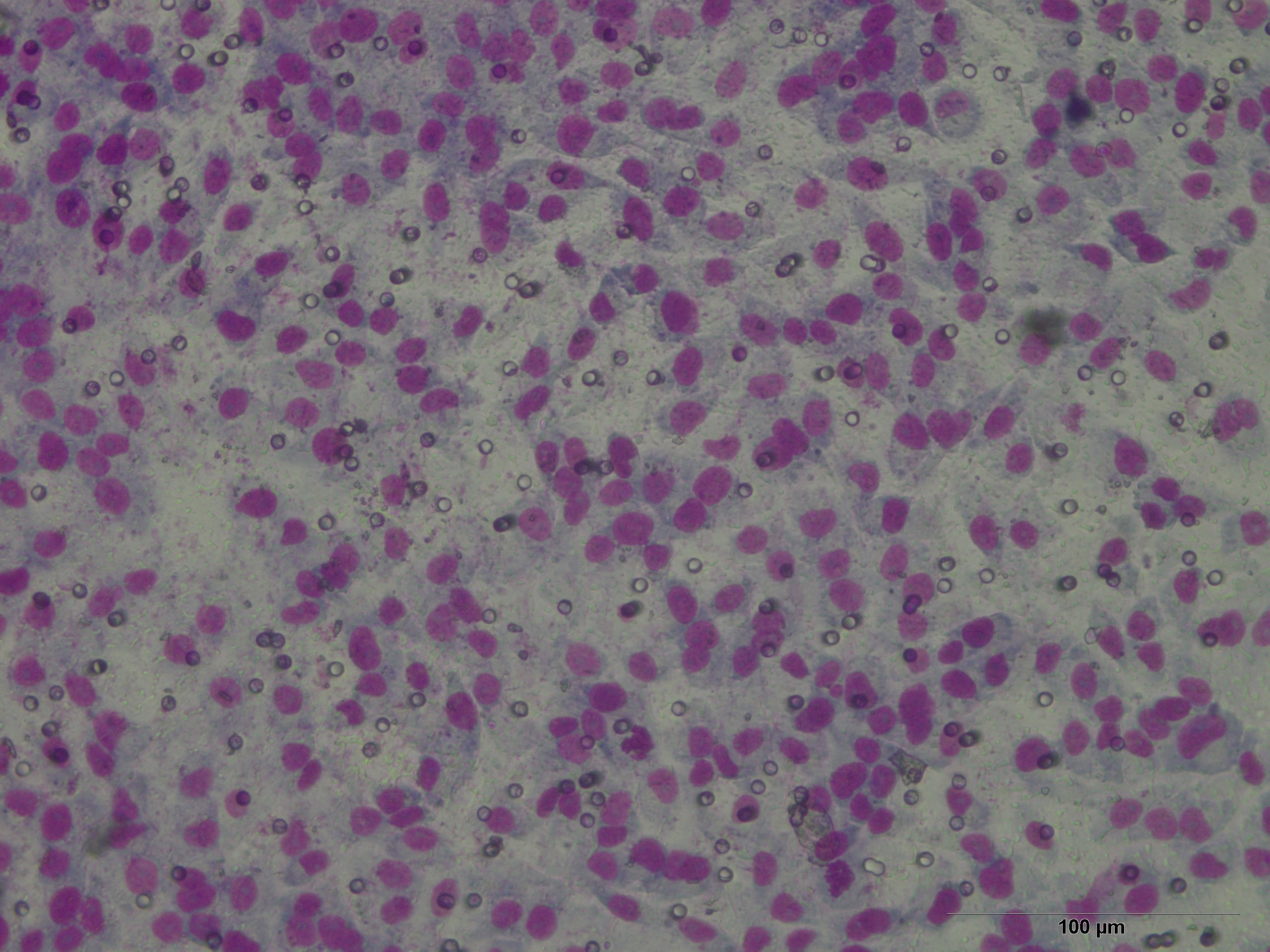

Supplement: Supplementary file 6 [file DataSheet_3.zip › Data Sheet 3/Fig4C/3-AC009948.5-COTRANS-M.jpg]

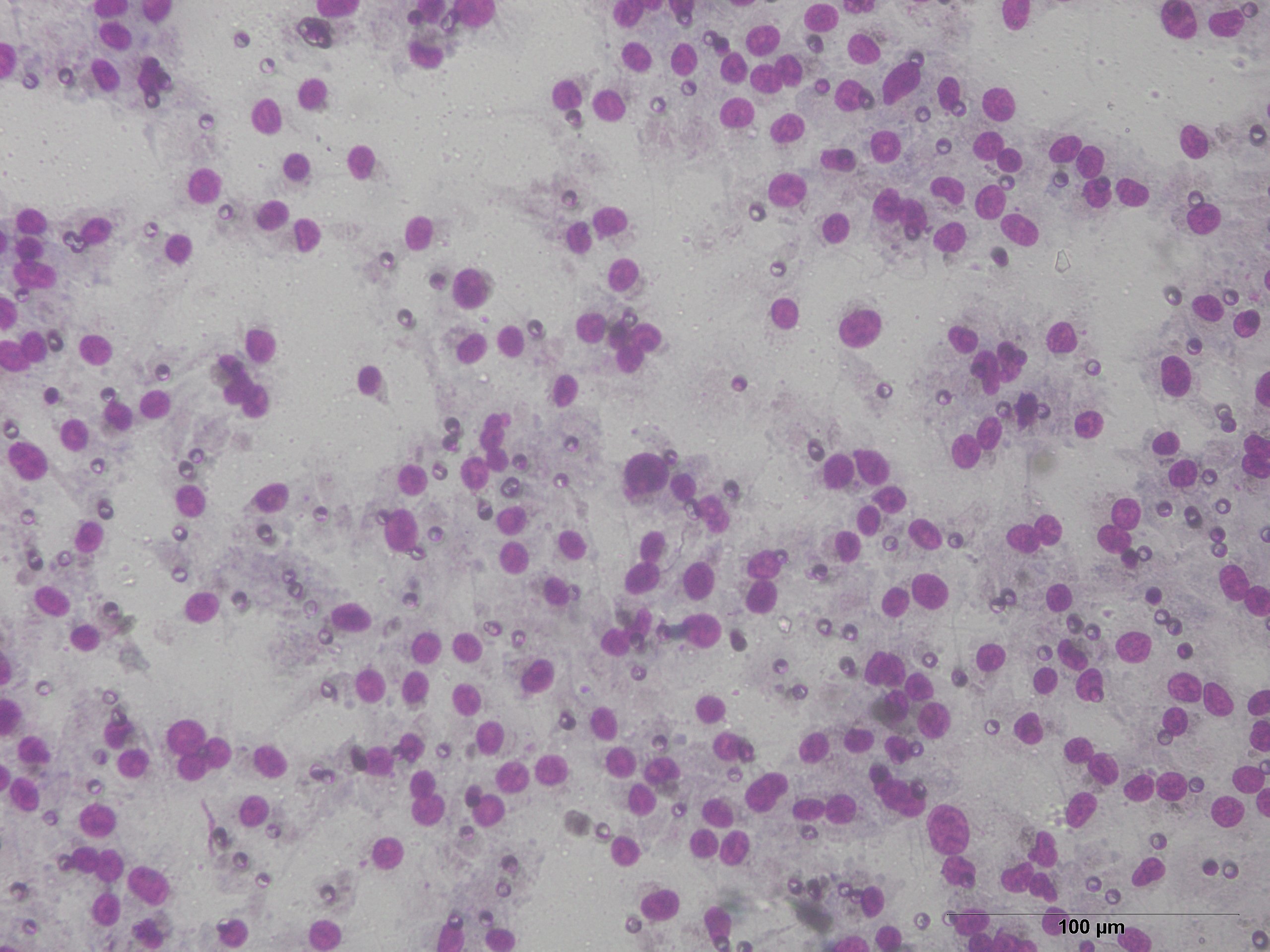

Supplement: Supplementary file 6 [file DataSheet_3.zip › Data Sheet 3/Fig4C/3-AC009948.5-OVER-186-M.jpg]

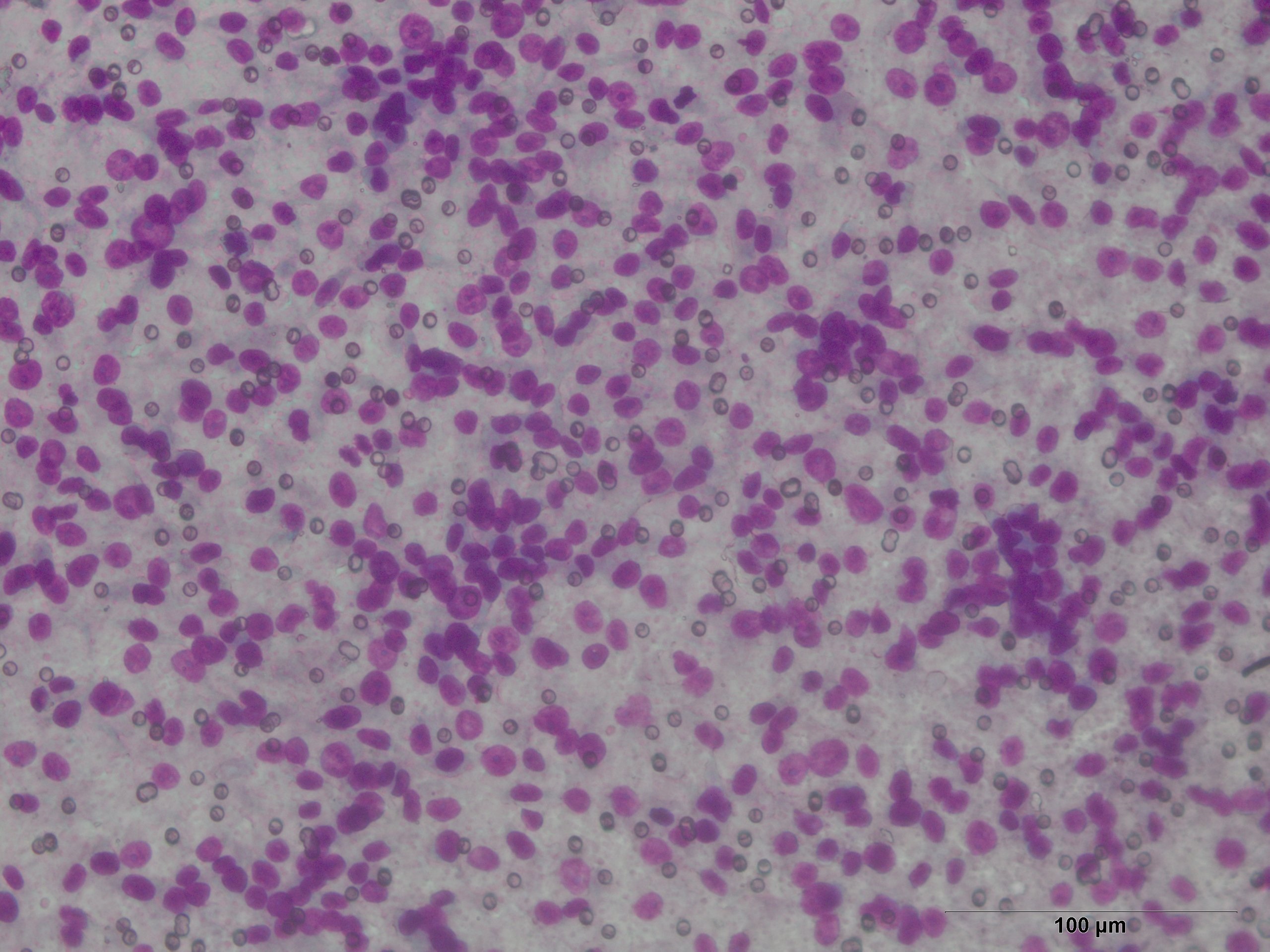

Supplement: Supplementary file 6 [file DataSheet_3.zip › Data Sheet 3/Fig4C/3-AC009948.5-sh-miR-186-M.jpg]

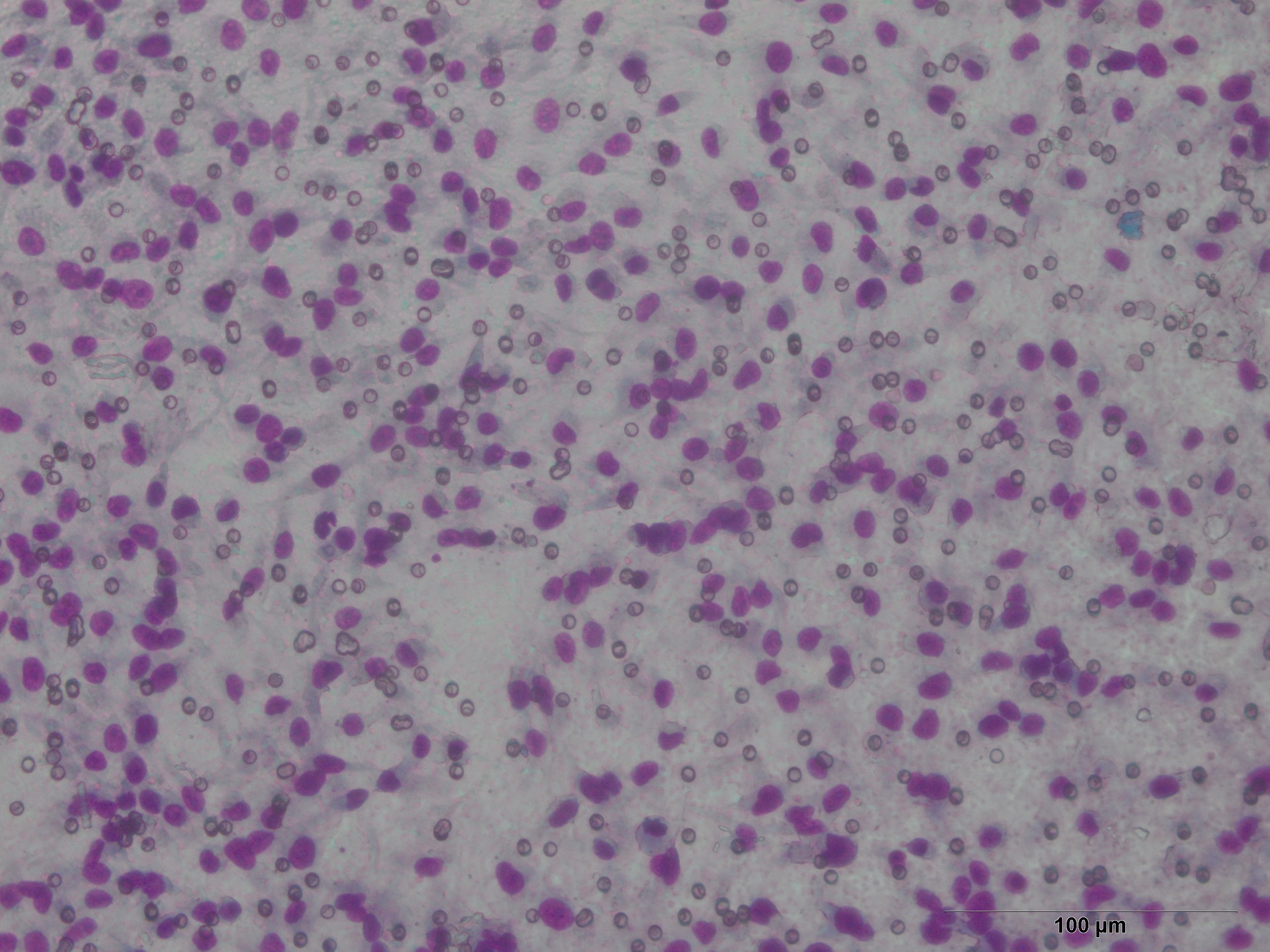

Supplement: Supplementary file 6 [file DataSheet_3.zip › Data Sheet 3/Fig4C/3-con-INVASION.jpg]

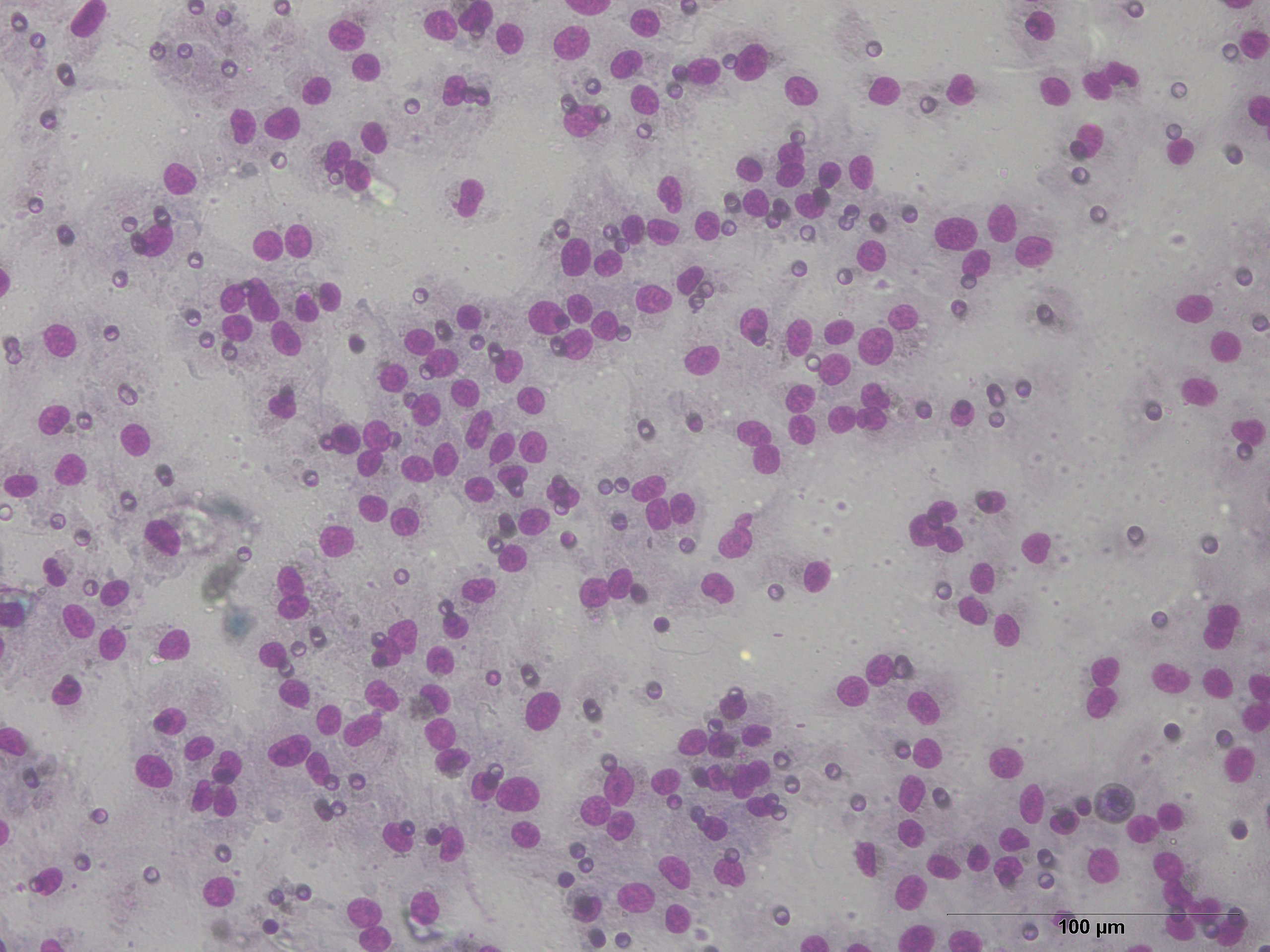

Supplement: Supplementary file 6 [file DataSheet_3.zip › Data Sheet 3/Fig4C/3-over-miR-186-5p-INVASION.jpg]

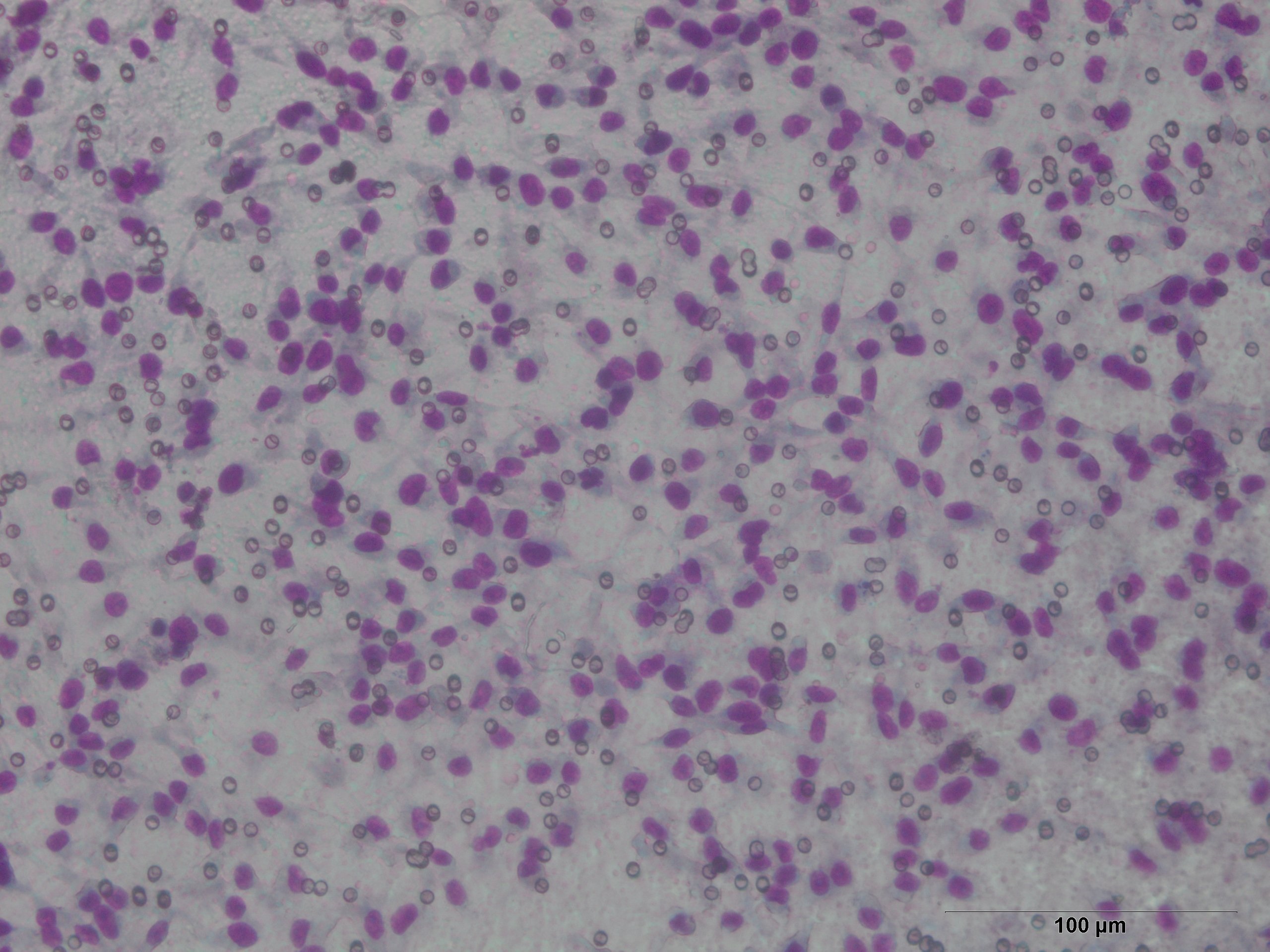

Supplement: Supplementary file 6 [file DataSheet_3.zip › Data Sheet 3/Fig4C/3-sh-con-INVASION.jpg]

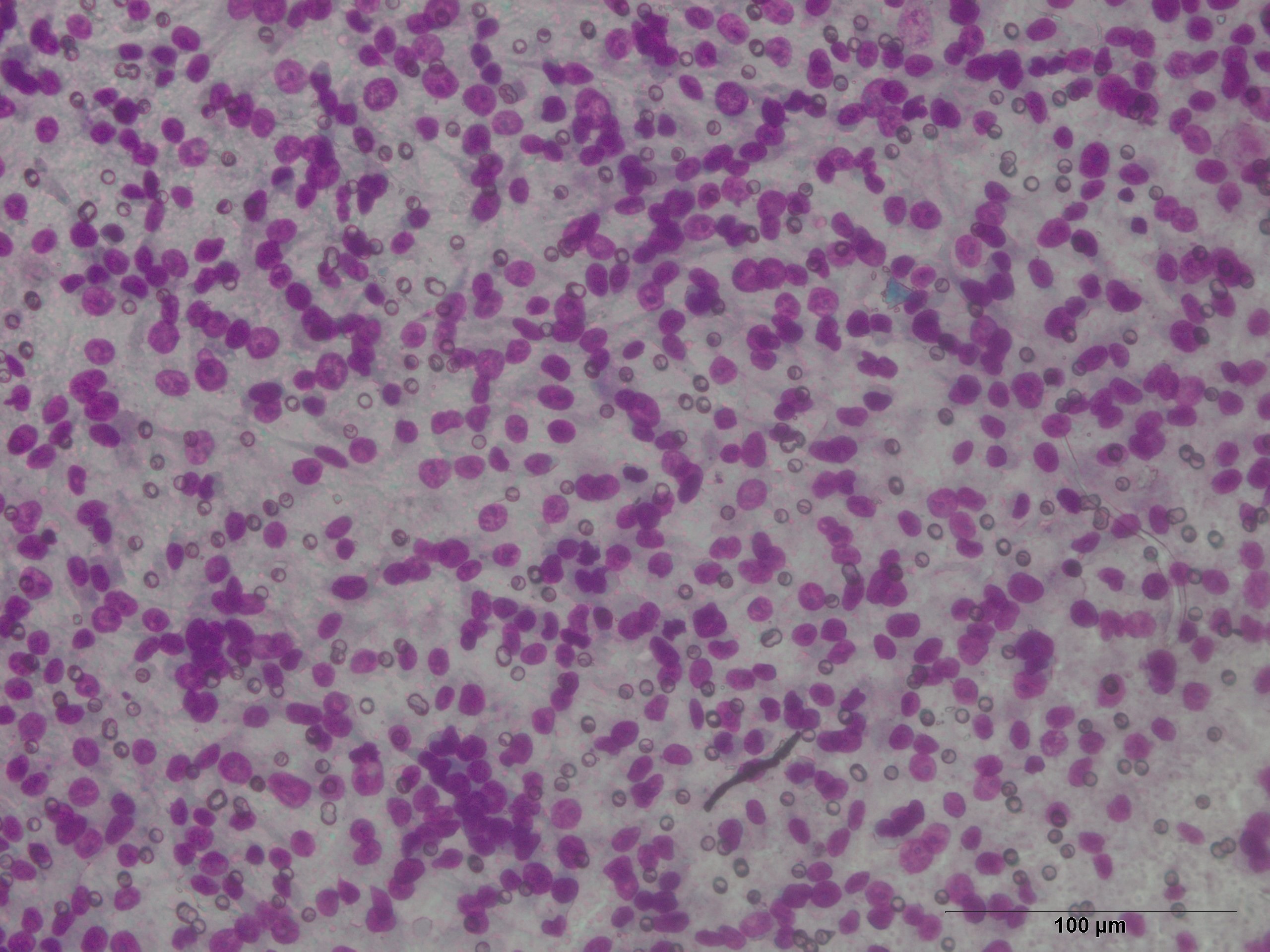

Supplement: Supplementary file 6 [file DataSheet_3.zip › Data Sheet 3/Fig4C/3-sh-miR-186-5p-INVASION.jpg]

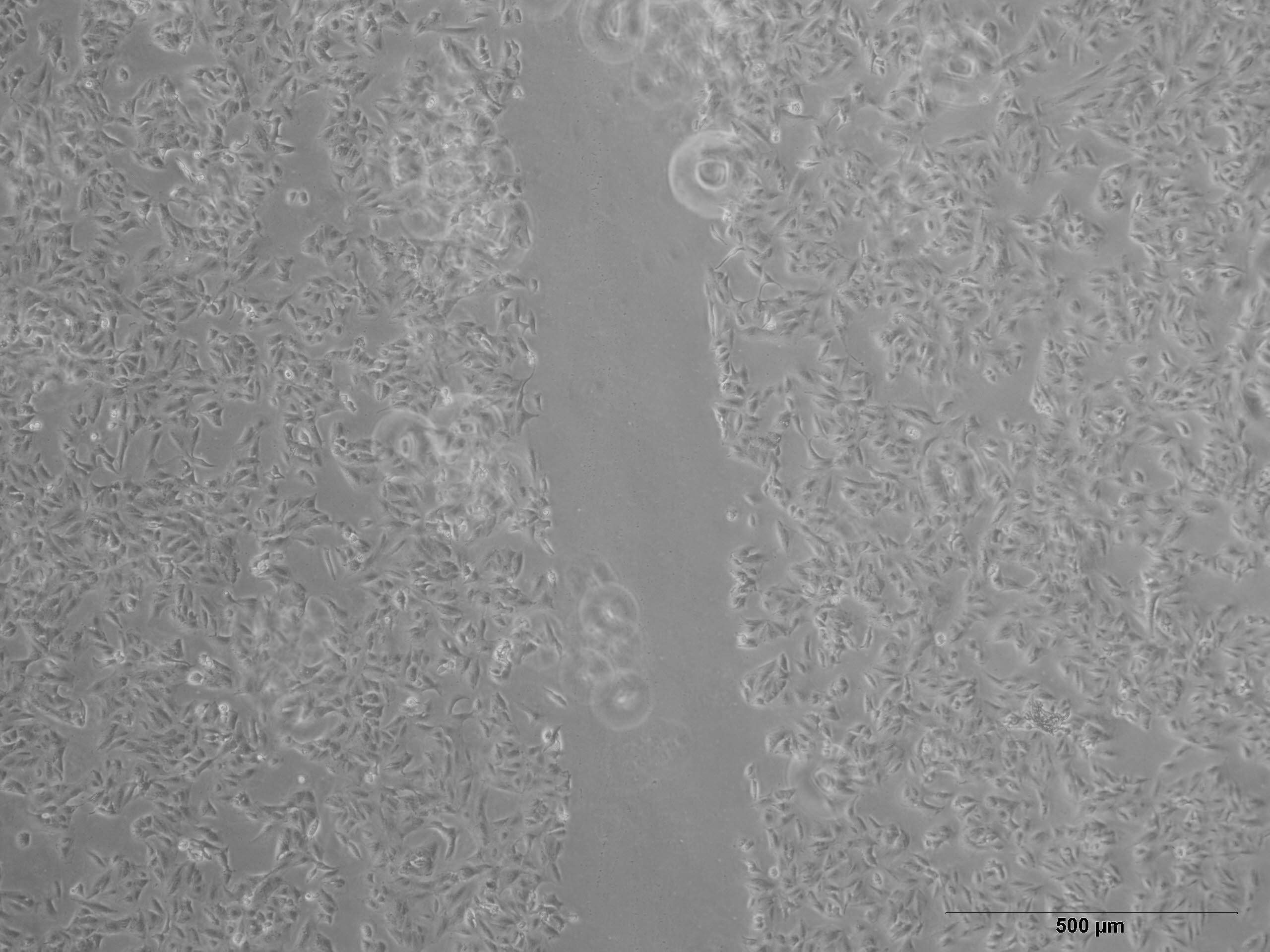

Supplement: Supplementary file 7 [file DataSheet_4.zip › Data Sheet 4/Fig4D/1-AC009948.5-CON-0H.jpg]

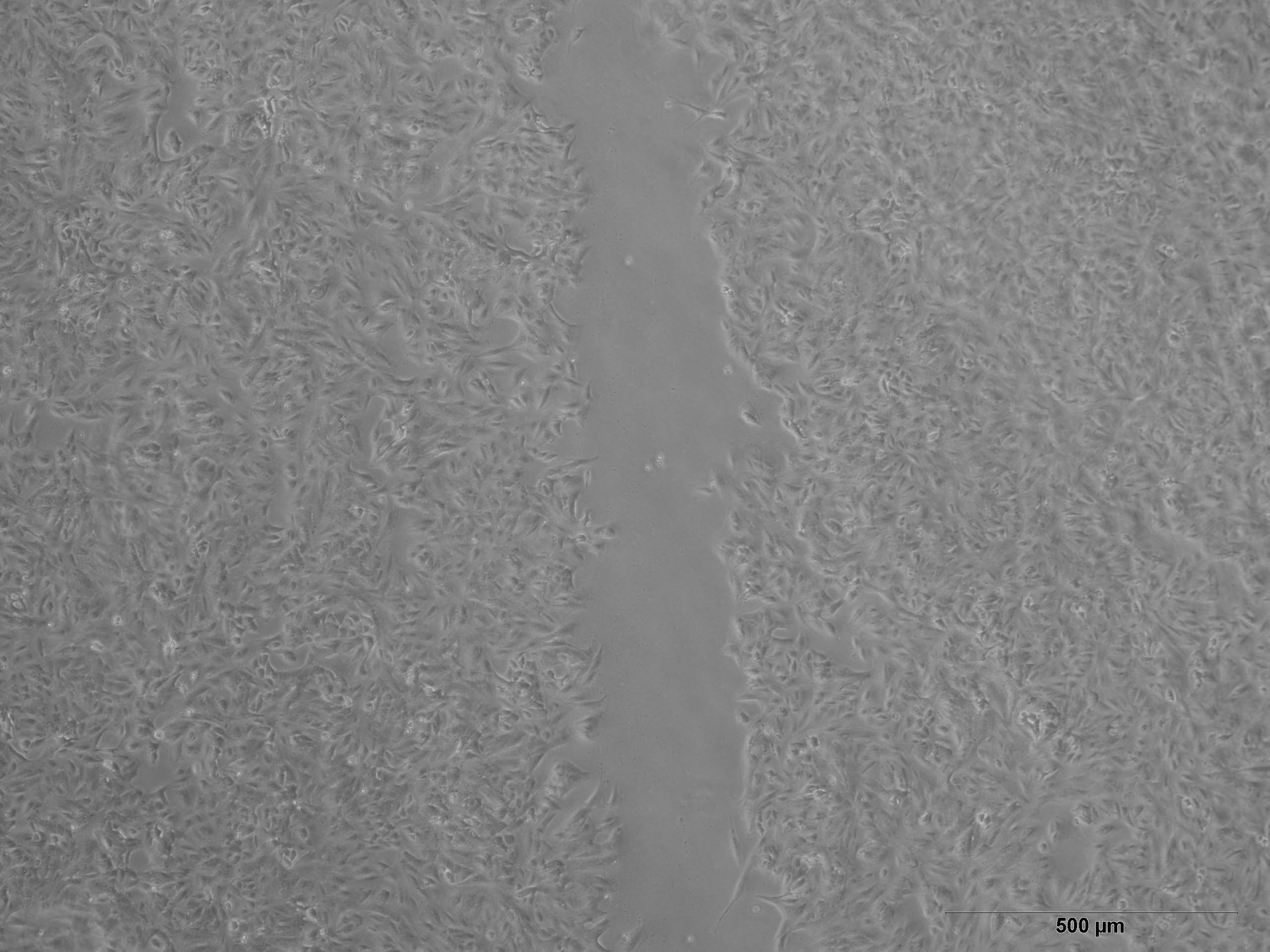

Supplement: Supplementary file 7 [file DataSheet_4.zip › Data Sheet 4/Fig4D/1-AC009948.5-CON-24h.jpg]

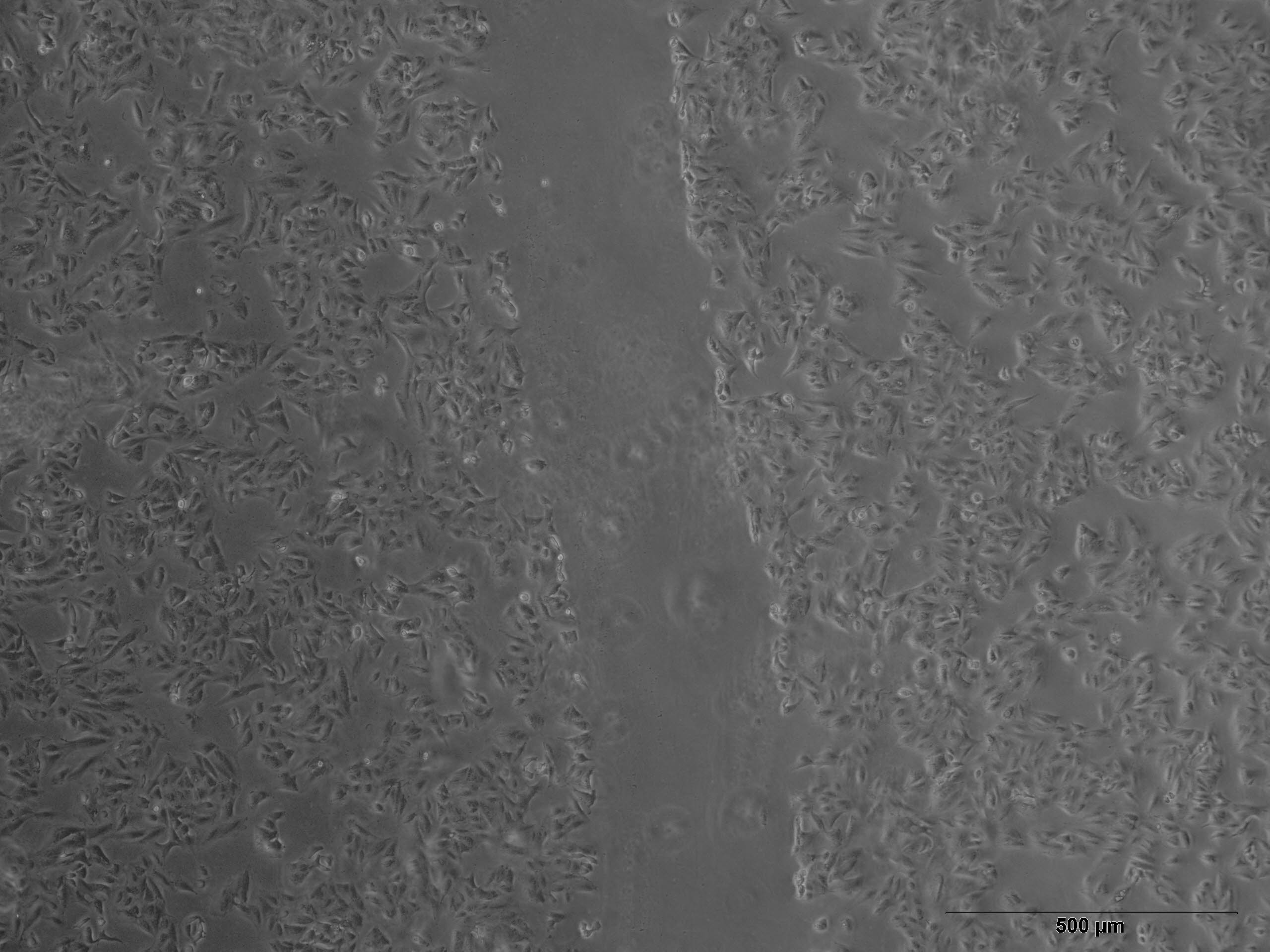

Supplement: Supplementary file 7 [file DataSheet_4.zip › Data Sheet 4/Fig4D/1-AC009948.5-COTRANS-0h.jpg]

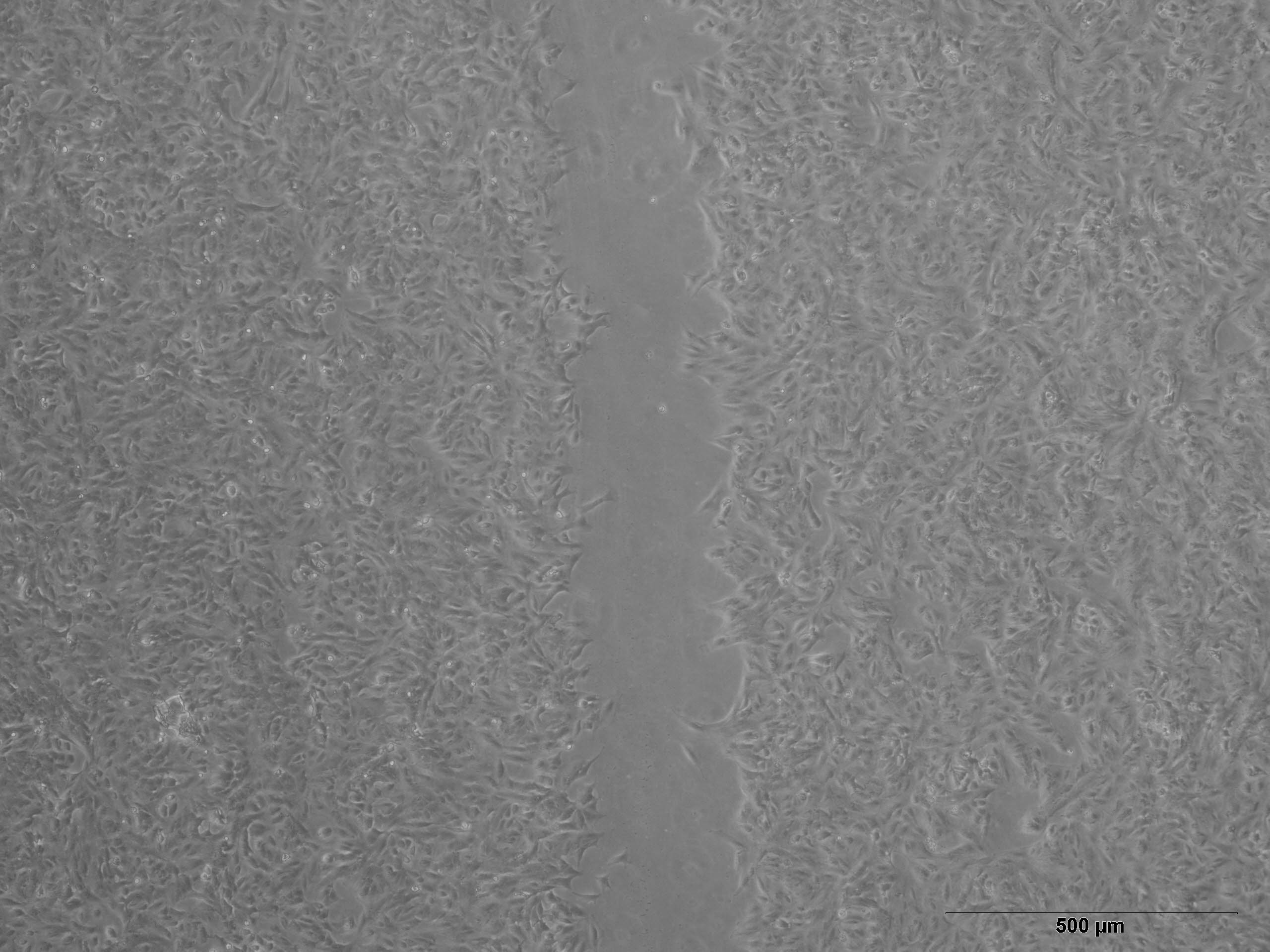

Supplement: Supplementary file 7 [file DataSheet_4.zip › Data Sheet 4/Fig4D/1-AC009948.5-COTRANS-24H.jpg]

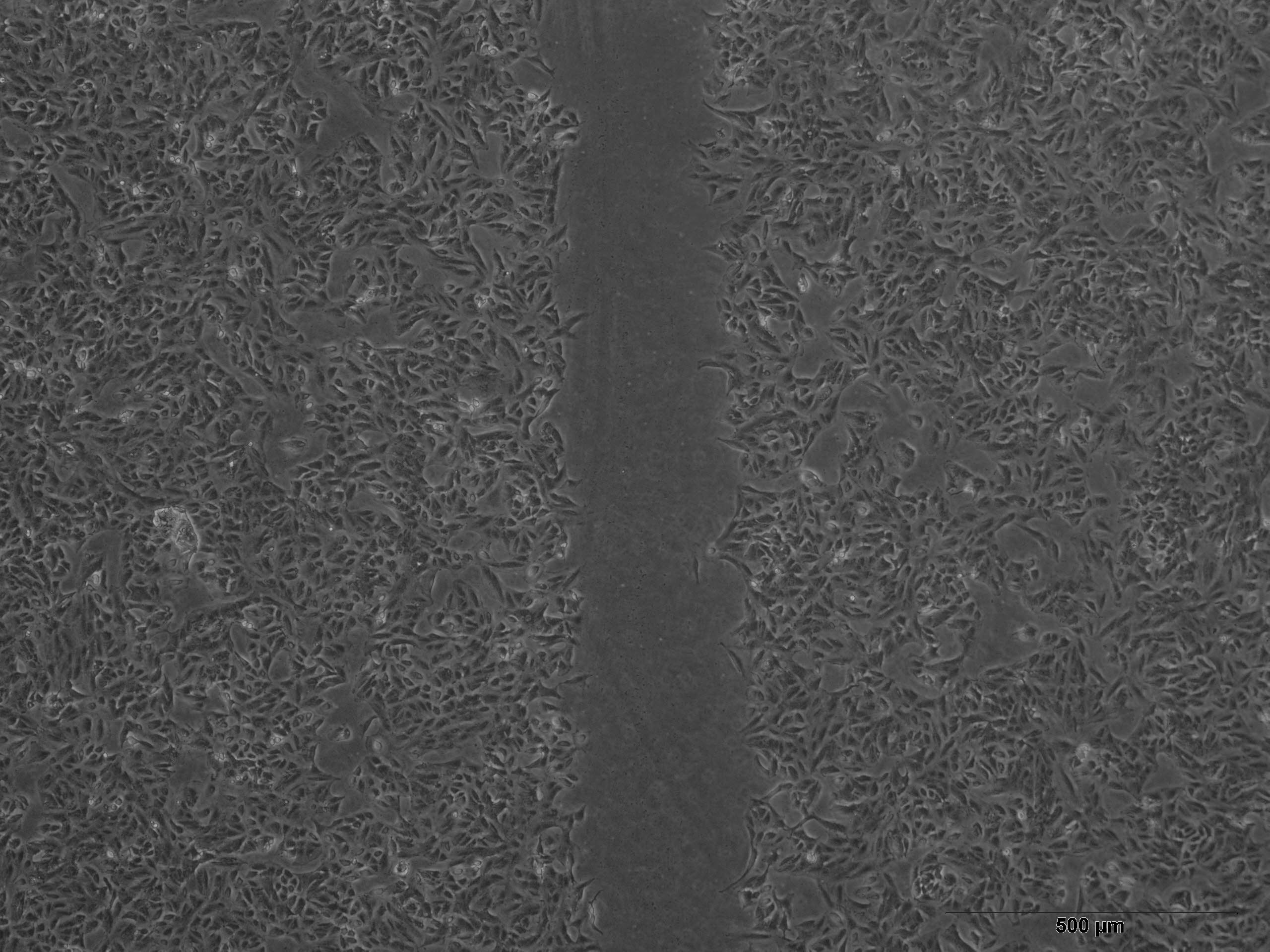

Supplement: Supplementary file 7 [file DataSheet_4.zip › Data Sheet 4/Fig4D/1-AC009948.5-over-186-24h.jpg]

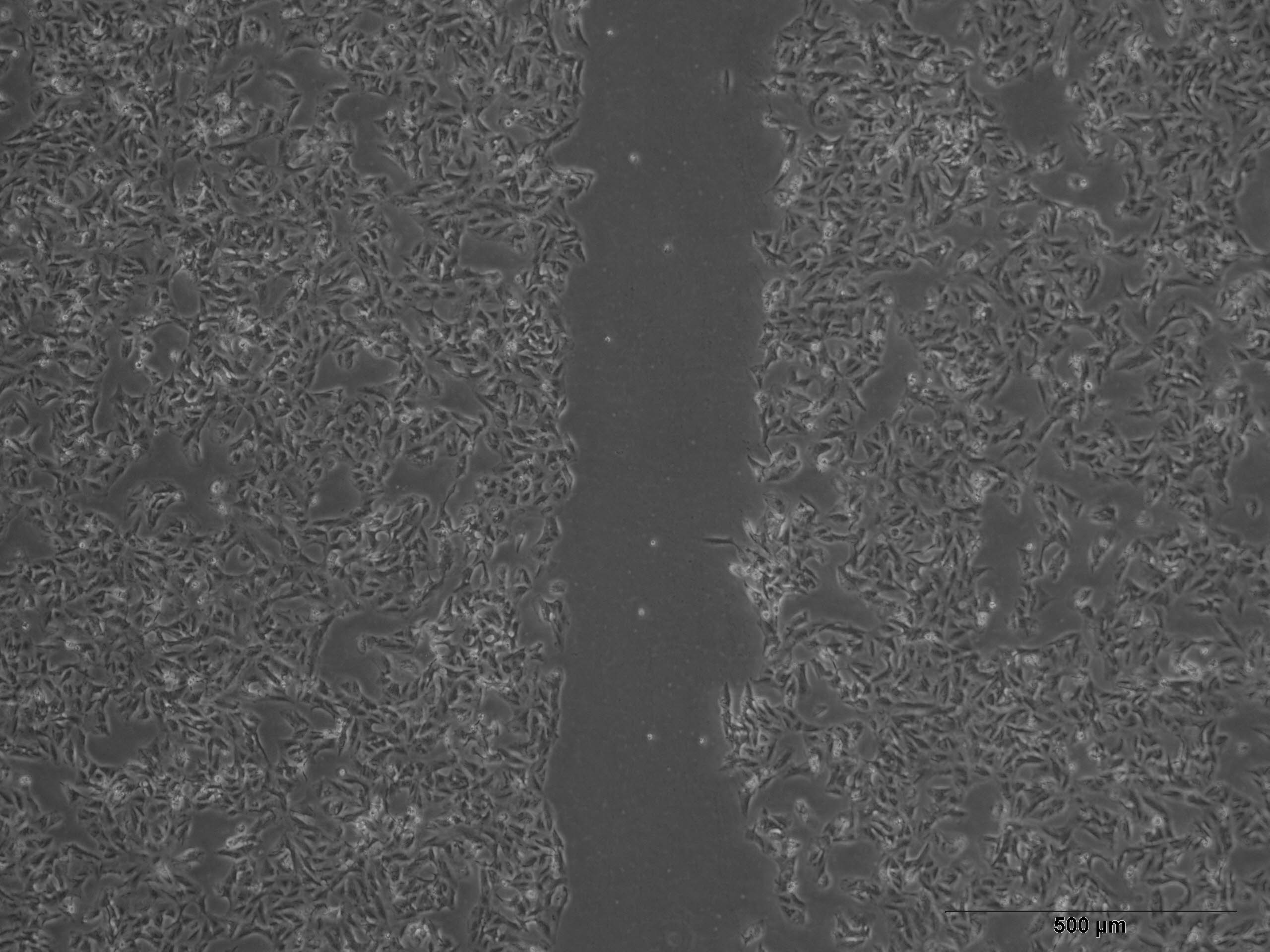

Supplement: Supplementary file 7 [file DataSheet_4.zip › Data Sheet 4/Fig4D/1-AC009948.5-Sh-186-0H.jpg]

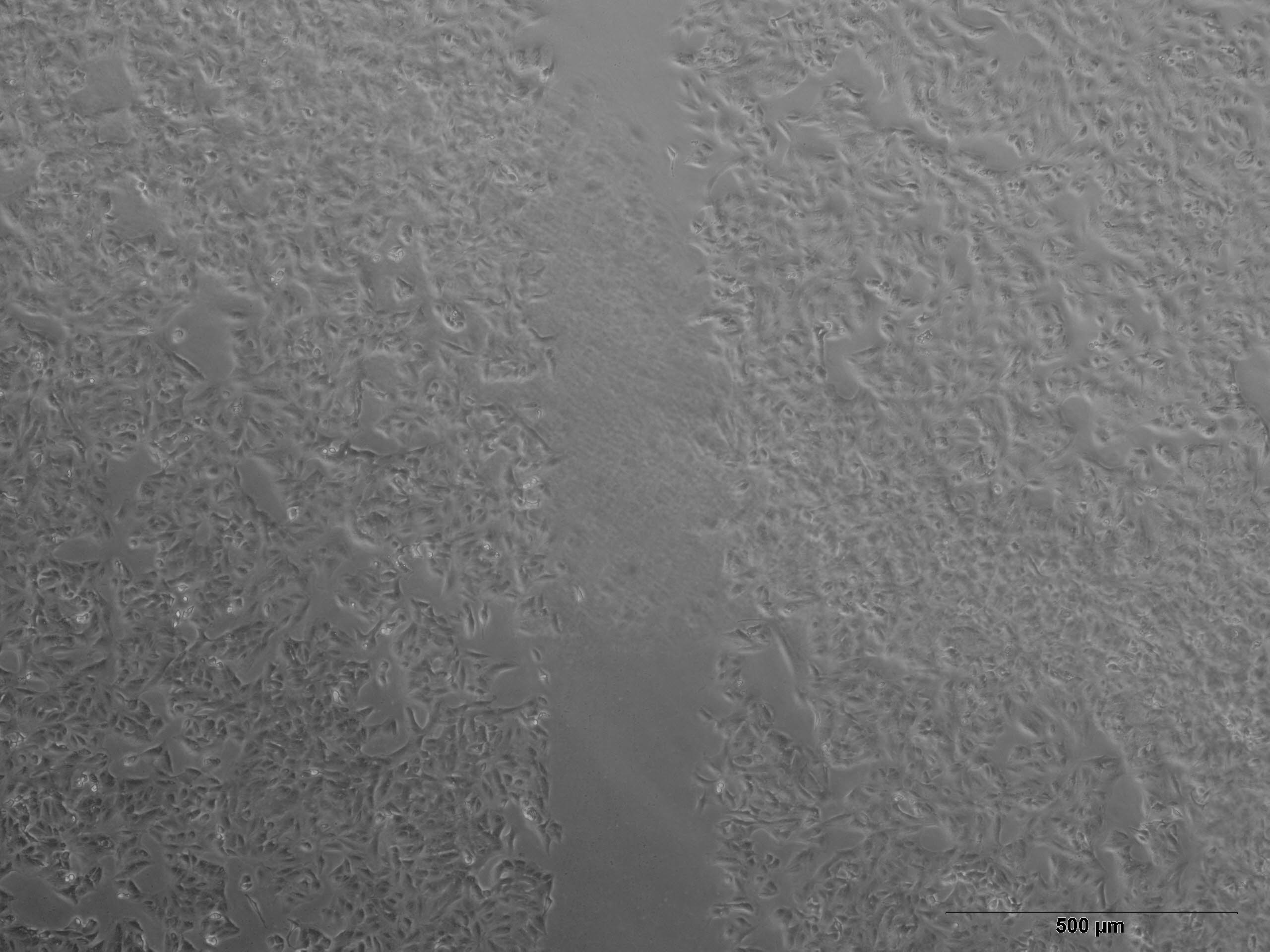

Supplement: Supplementary file 7 [file DataSheet_4.zip › Data Sheet 4/Fig4D/1-AC009948.5-sh-con-0H.jpg]

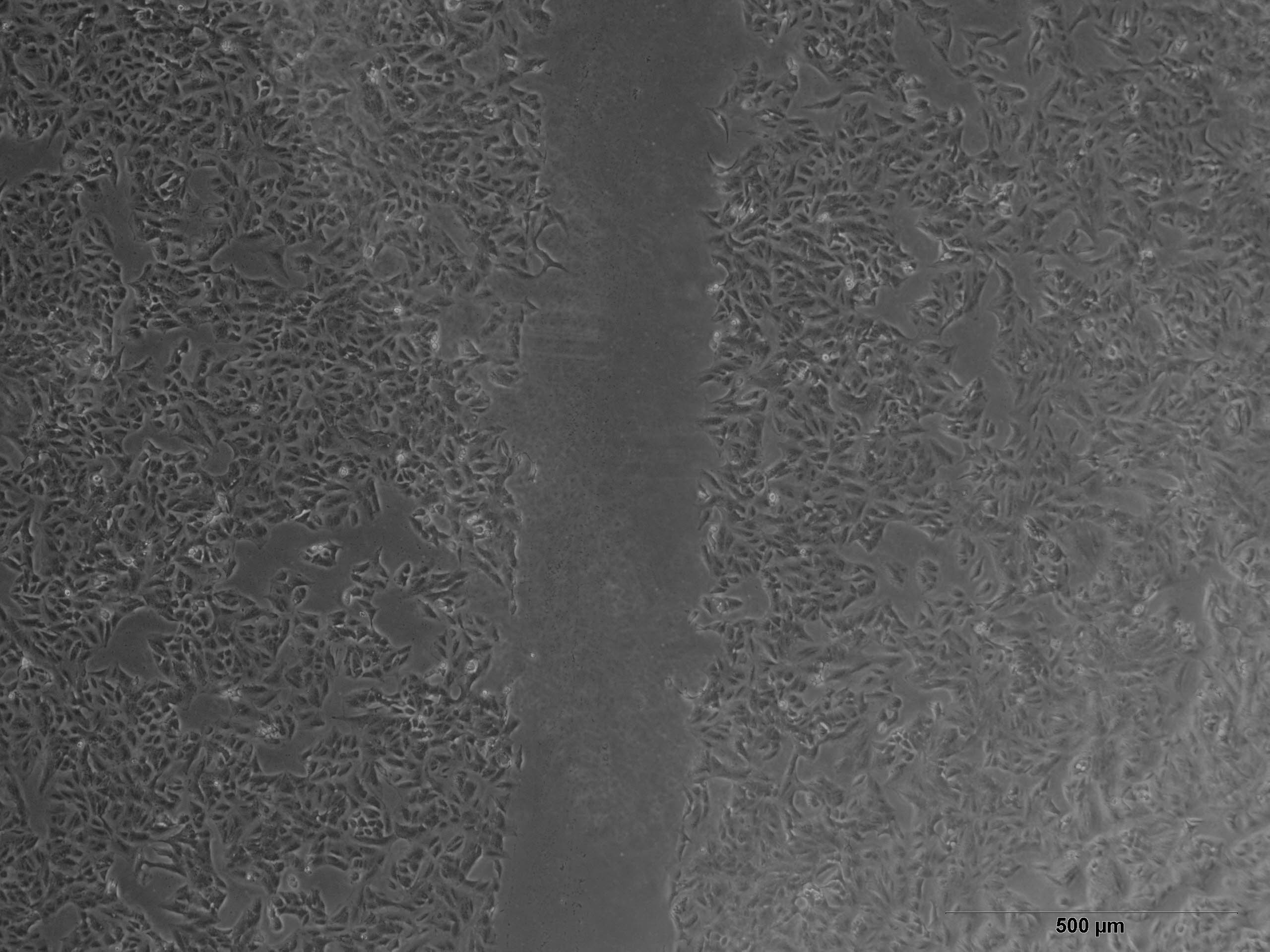

Supplement: Supplementary file 7 [file DataSheet_4.zip › Data Sheet 4/Fig4D/1-AC009948.5-sh-con-24H.jpg]

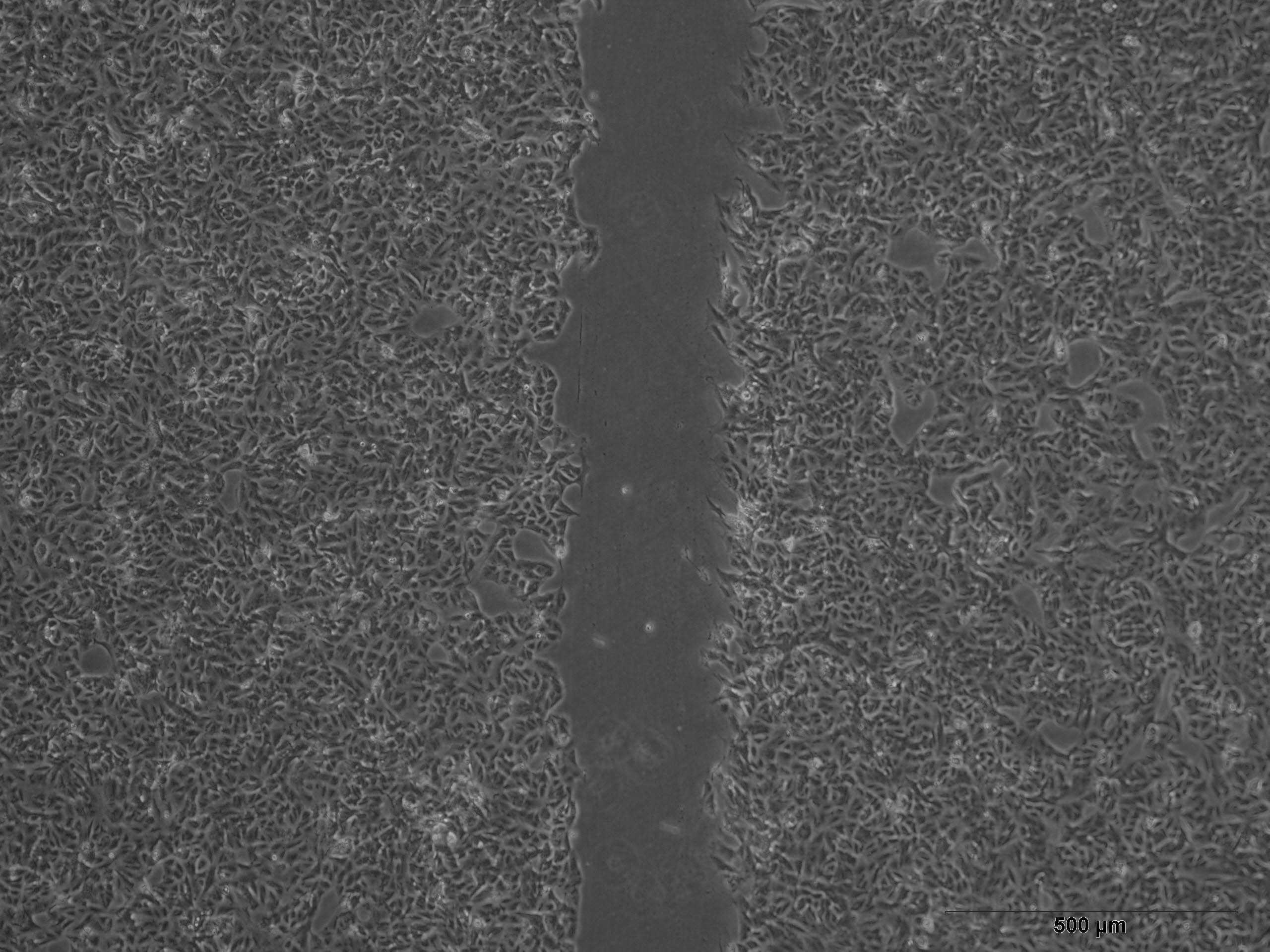

Supplement: Supplementary file 7 [file DataSheet_4.zip › Data Sheet 4/Fig4D/1-AC009948.5-sh186-24h.jpg]

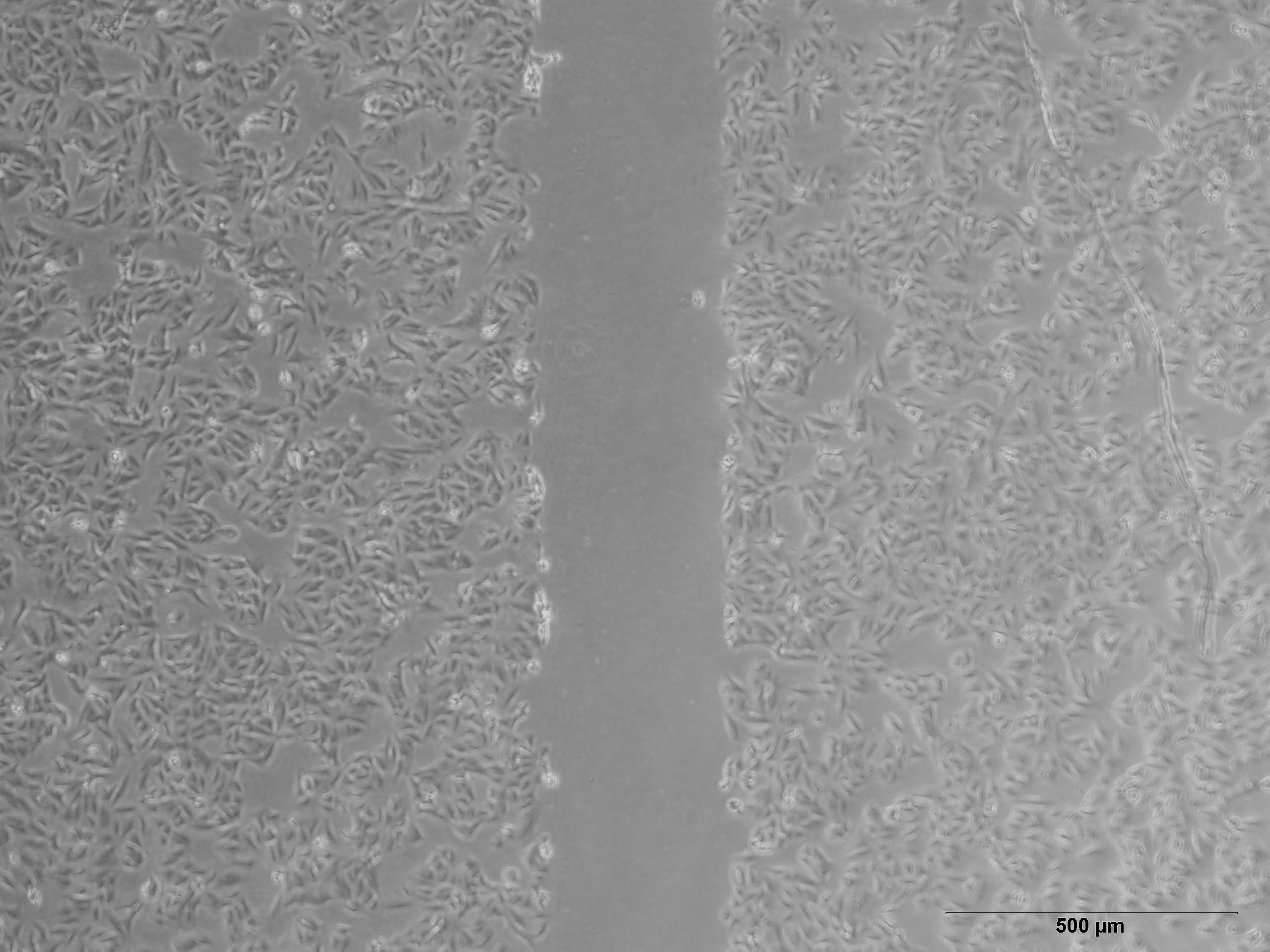

Supplement: Supplementary file 7 [file DataSheet_4.zip › Data Sheet 4/Fig4D/1-AC009958.5-over-186-0h.jpg]

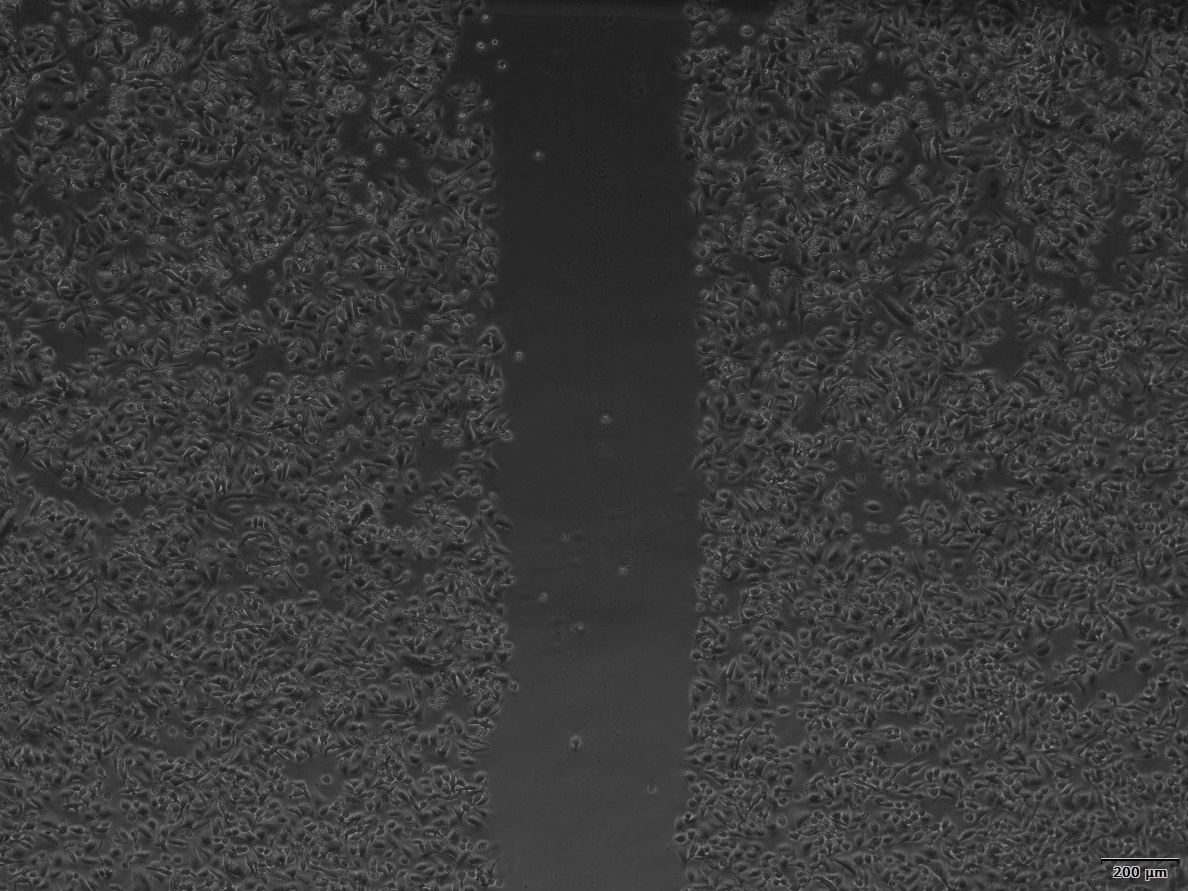

Supplement: Supplementary file 7 [file DataSheet_4.zip › Data Sheet 4/Fig4D/2-AC009948.5-CON-0H.jpg]

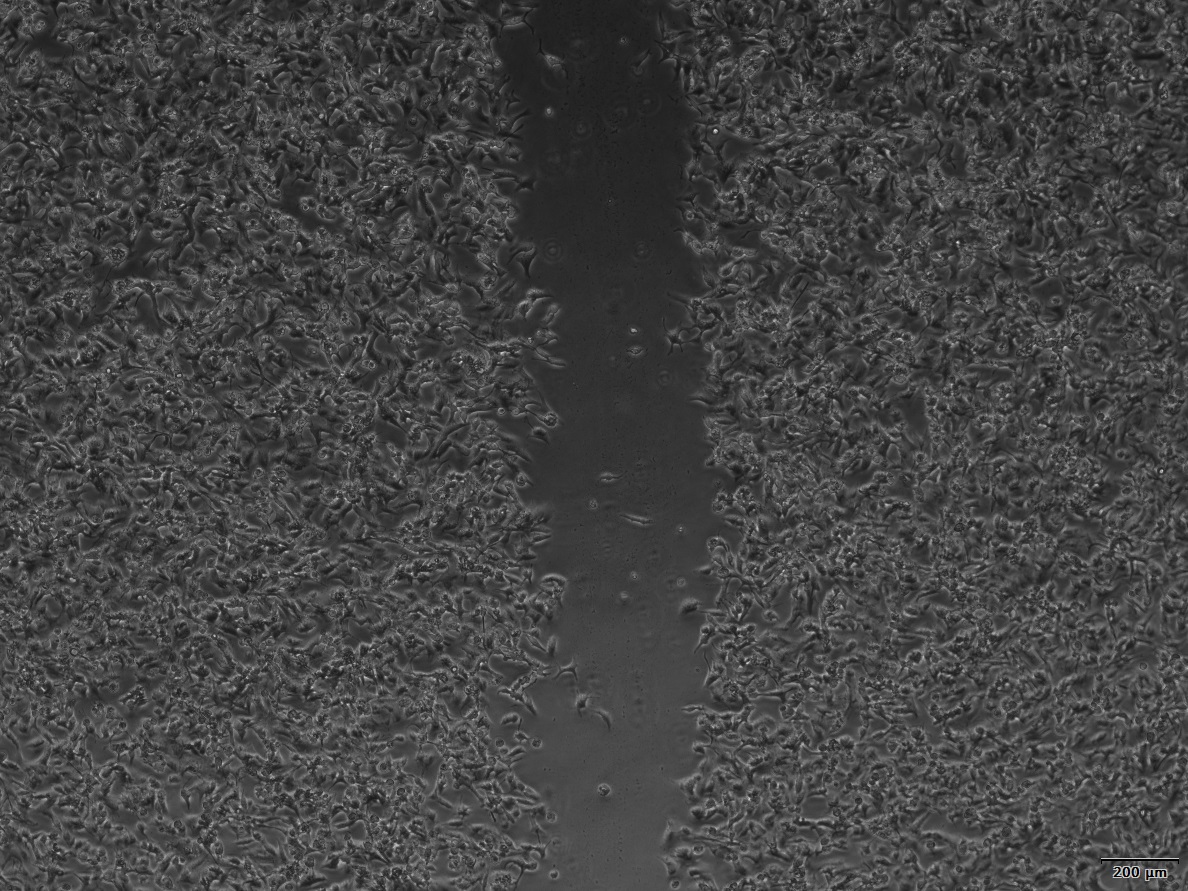

Supplement: Supplementary file 7 [file DataSheet_4.zip › Data Sheet 4/Fig4D/2-AC009948.5-CON-24h.jpg]

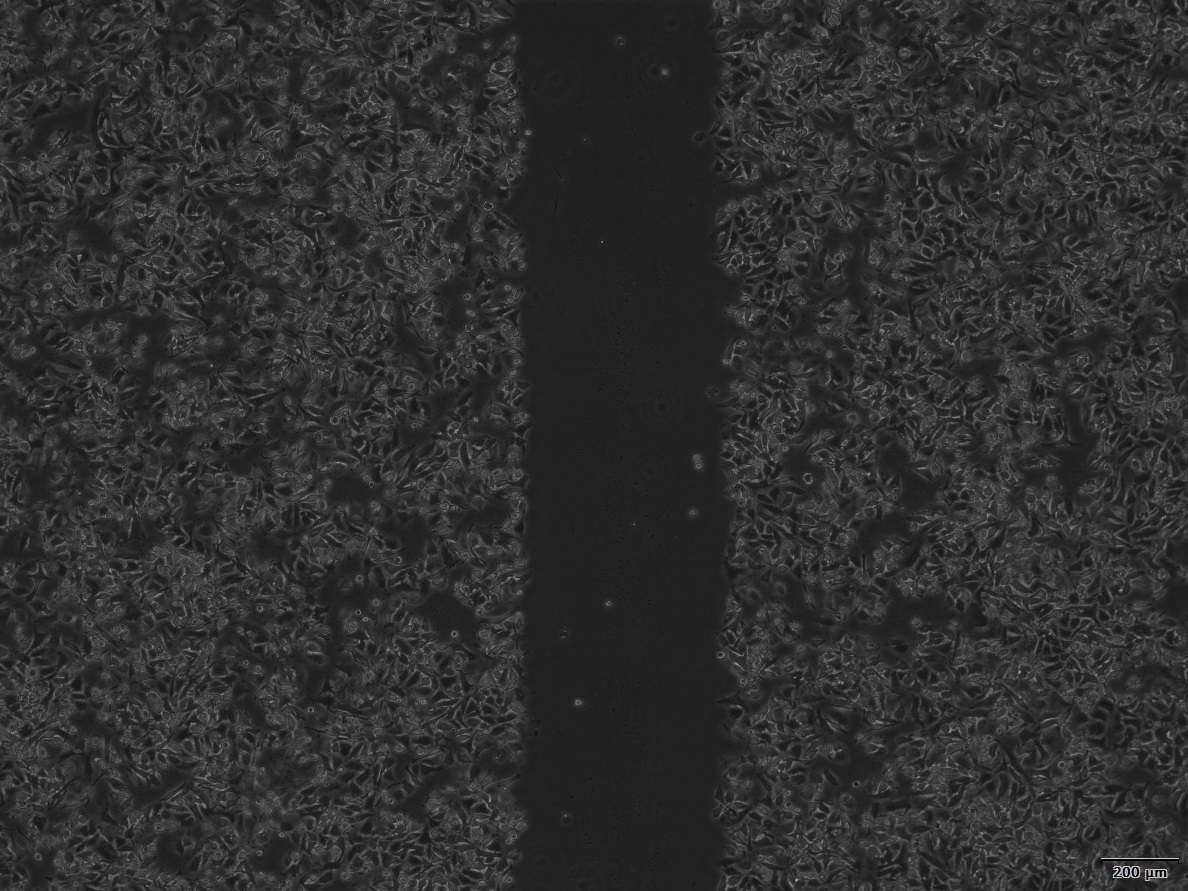

Supplement: Supplementary file 7 [file DataSheet_4.zip › Data Sheet 4/Fig4D/2-AC009948.5-COTRANS-0h.jpg]

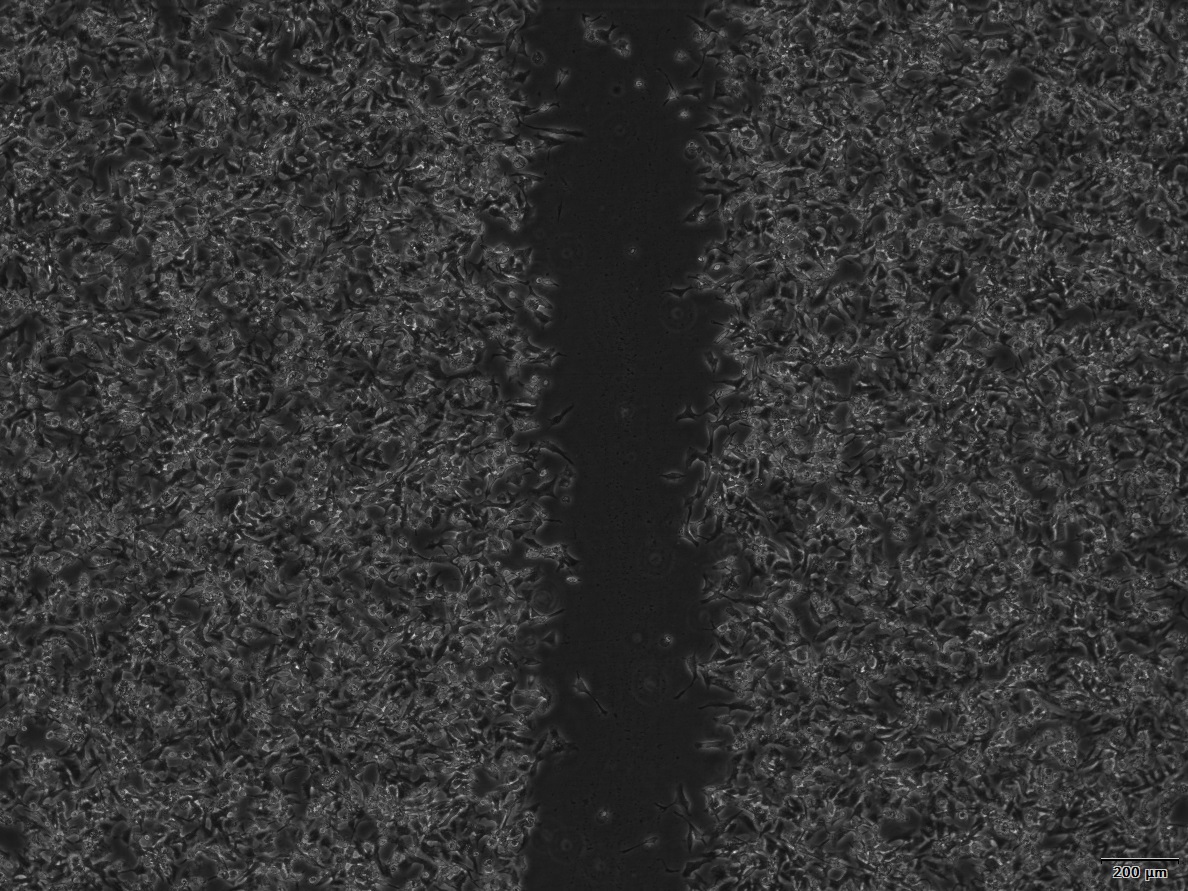

Supplement: Supplementary file 7 [file DataSheet_4.zip › Data Sheet 4/Fig4D/2-AC009948.5-COTRANS-24H.jpg]

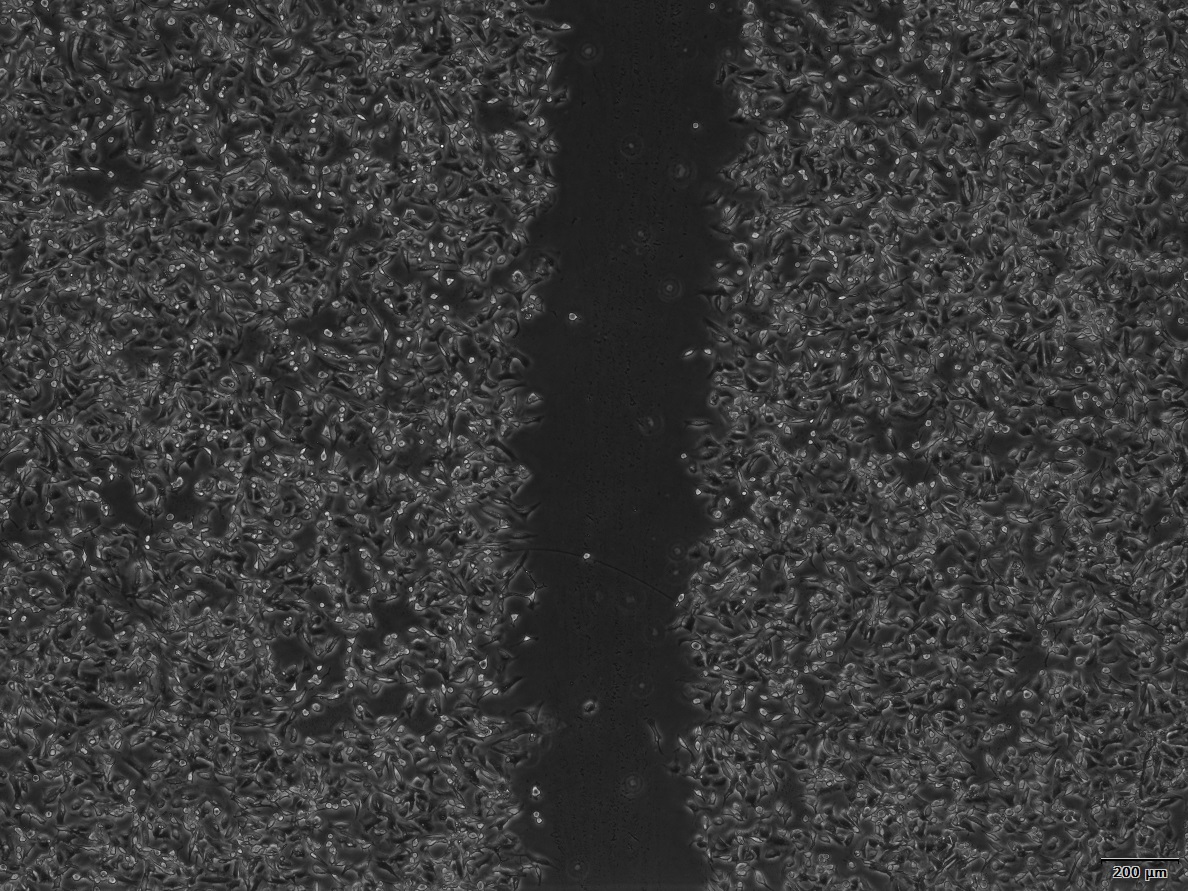

Supplement: Supplementary file 7 [file DataSheet_4.zip › Data Sheet 4/Fig4D/2-AC009948.5-over-186-24h.jpg]

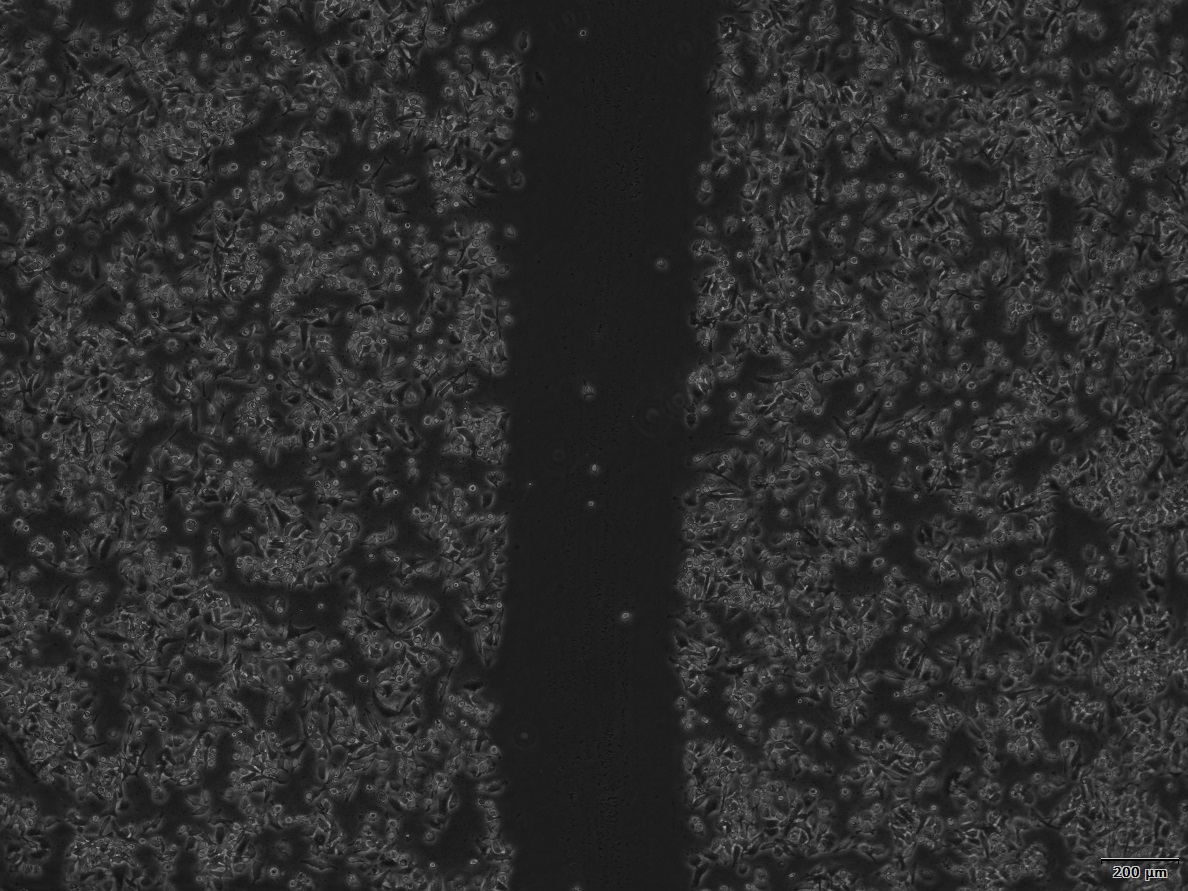

Supplement: Supplementary file 7 [file DataSheet_4.zip › Data Sheet 4/Fig4D/2-AC009948.5-Sh-186-0H.jpg]
