# Supplementary material for: LncRNA-AC009948.5 promotes invasion and metastasis of lung adenocarcinoma by binding to miR-186-5p
Source: Front Oncol. 2022 Aug 19;12:949951. doi: 10.3389/fonc.2022.949951 (PMC9437580; doi:10.3389/fonc.2022.949951)
Supplement: Supplementary file 7 [file DataSheet_4.zip › Data Sheet 4/FigS1B/AC009948.5-1/Scrambled-1.pdf]

BD FACSDiva 8.0.1

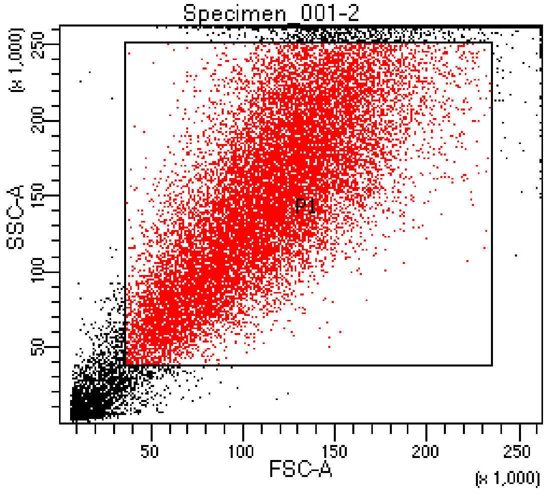

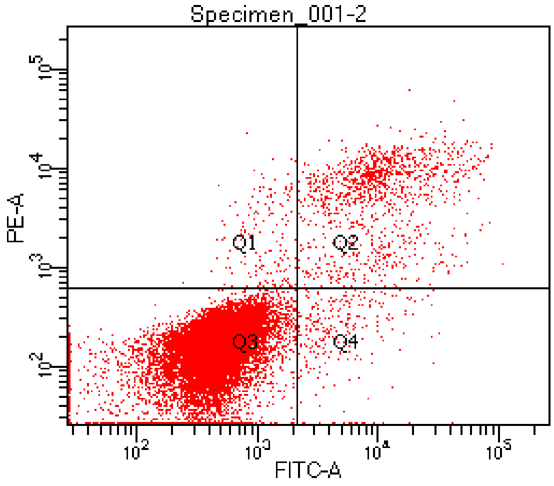

Experiment Name:	20220512-CL-02
Specimen Name:	Specimen_001
Tube Name:	2
Record Date:	May 12, 2022 3:04:41 PM
$OP:	Administrator
GUID:	48dd0018-245b-4856-9aff-bcda...

Population	#Events	%Parent	FITC-A Mean	PE-A Mean
P1	19,051	63.5	1,446	613
Q1	####	2.9	1,472	1,472
Q2	####	10.7	11,978	6,650
Q3	####	82.9	580	195
Q4	####	3.5	5,110	313
