# Supplementary material for: LncRNA-AC009948.5 promotes invasion and metastasis of lung adenocarcinoma by binding to miR-186-5p
Source: Front Oncol. 2022 Aug 19;12:949951. doi: 10.3389/fonc.2022.949951 (PMC9437580; doi:10.3389/fonc.2022.949951)
Supplement: Supplementary file 7 [file DataSheet_4.zip › Data Sheet 4/FigS1B/AC009948.5-1/SiAC009948.5-1.pdf]

BD FACSDiva 8.0.1

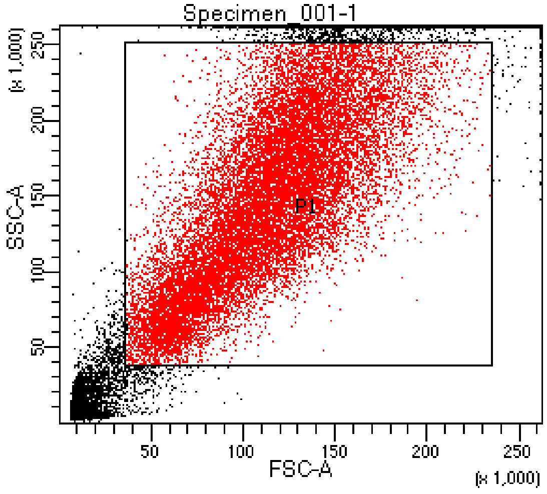

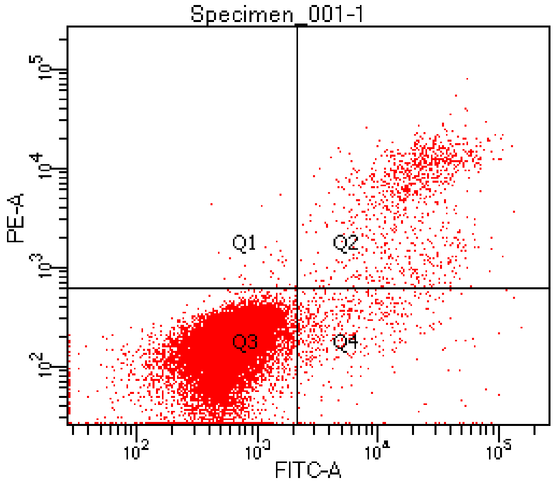

Experiment Name:	20220512-CL-02
Specimen Name:	Specimen_001
Tube Name:	1
Record Date:	May 12, 2022 3:03:55 PM
$OP:	Administrator
GUID:	f2f76568-1c2a-4fd1-b487-3573f...

Population	#Events	%Parent	FITC-A Mean	PE-A Mean
P1	20,071	66.9	2,064	537
Q1	####	1.1	1,587	1,013
Q2	####	8.7	20,379	6,999
Q3	####	82.8	704	192
Q4	####	7.4	7,528	290
