# Supplementary material for: LncRNA-AC009948.5 promotes invasion and metastasis of lung adenocarcinoma by binding to miR-186-5p
Source: Front Oncol. 2022 Aug 19;12:949951. doi: 10.3389/fonc.2022.949951 (PMC9437580; doi:10.3389/fonc.2022.949951)
Supplement: Supplementary file 7 [file DataSheet_4.zip › Data Sheet 4/FigS1B/AC009948.5-1/Specimen_001_FITC_12052022161346.pdf]

BD FACSDiva 8.0.1

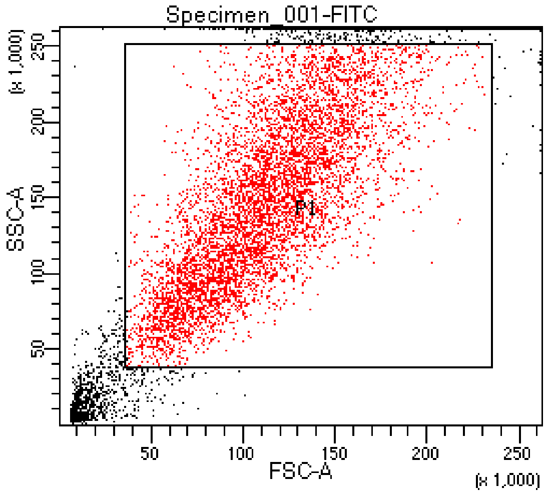

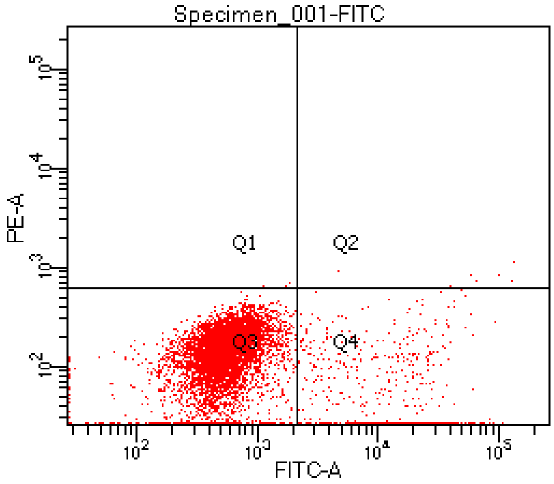

Experiment Name:	20220512-CL-02
Specimen Name:	Specimen_001
Tube Name:	FITC
Record Date:	May 12, 2022 3:01:53 PM
$OP:	Administrator
GUID:	25fed34a-3310-4798-bd4c-653...

Population	#Events	%Parent	FITC-A Mean	PE-A Mean
P1	6,557	65.6	2,187	134
Q1	####	0.8	1,709	729
Q2	####	2.4	7,324	904
Q3	####	82.2	676	171
Q4	####	14.5	14,955	90
