# Supplementary material for: LncRNA-AC009948.5 promotes invasion and metastasis of lung adenocarcinoma by binding to miR-186-5p
Source: Front Oncol. 2022 Aug 19;12:949951. doi: 10.3389/fonc.2022.949951 (PMC9437580; doi:10.3389/fonc.2022.949951)
Supplement: Supplementary file 7 [file DataSheet_4.zip › Data Sheet 4/FigS1B/AC009948.5-1/Specimen_001_nc_12052022161337.pdf]

BD FACSDiva 8.0.1

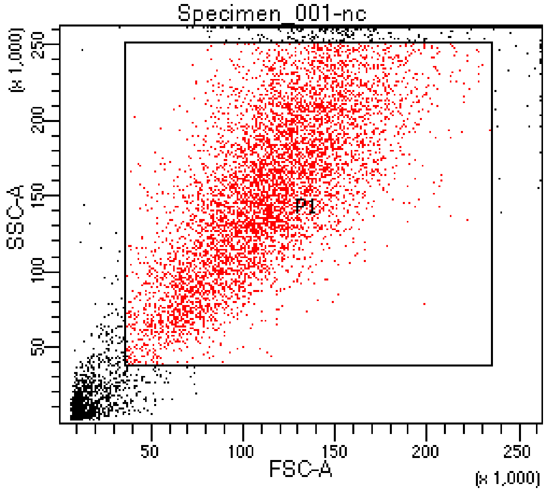

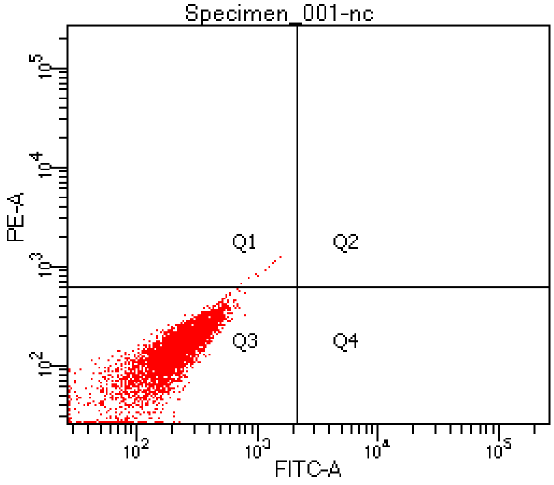

Experiment Name:	20220512-CL-02
Specimen Name:	Specimen_001
Tube Name:	nc
Record Date:	May 12, 2022 3:01:08 PM
$OP:	Administrator
GUID:	f9285af1-3330-4985-b318-93b1...

Population	#Events	%Parent	FITC-A Mean	PE-A Mean
P1	5,918	59.2	225	155
Q1	####	4.7	1,195	844
Q2	####	0.5	2,632	1,815
Q3	####	94.8	264	181
Q4	####	0.0	####	####
