# Supplementary material for: LncRNA-AC009948.5 promotes invasion and metastasis of lung adenocarcinoma by binding to miR-186-5p
Source: Front Oncol. 2022 Aug 19;12:949951. doi: 10.3389/fonc.2022.949951 (PMC9437580; doi:10.3389/fonc.2022.949951)
Supplement: Supplementary file 7 [file DataSheet_4.zip › Data Sheet 4/FigS1B/AC009948.5-1/Specimen_001_PI_12052022161350.pdf]

BD FACSDiva 8.0.1

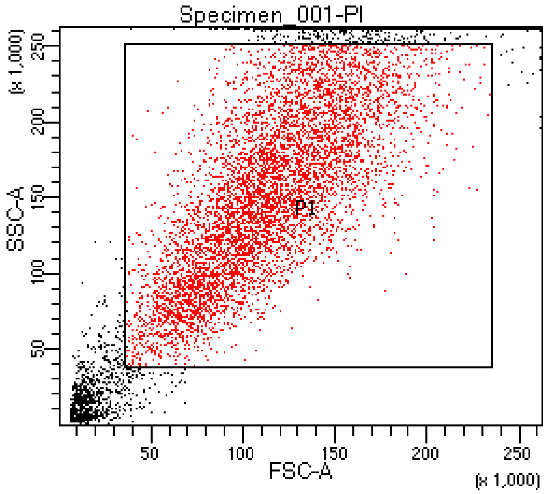

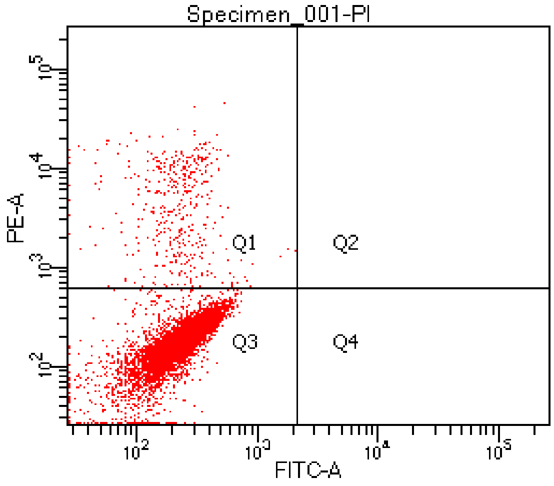

Experiment Name:	20220512-CL-02
Specimen Name:	Specimen_001
Tube Name:	PI
Record Date:	May 12, 2022 3:02:39 PM
$OP:	Administrator
GUID:	a6f6914e-6b7c-4812-abad-e83...

Population	#Events	%Parent	FITC-A Mean	PE-A Mean
P1	6,377	63.8	238	618
Q1	####	13.0	570	5,186
Q2	####	0.2	2,654	4,050
Q3	####	86.8	270	219
Q4	####	0.0	####	####
