# Supplementary material for: LncRNA-AC009948.5 promotes invasion and metastasis of lung adenocarcinoma by binding to miR-186-5p
Source: Front Oncol. 2022 Aug 19;12:949951. doi: 10.3389/fonc.2022.949951 (PMC9437580; doi:10.3389/fonc.2022.949951)
Supplement: Supplementary file 7 [file DataSheet_4.zip › Data Sheet 4/FigS1B/AC009948.5-2-3/Scrambled-2.pdf]

BD FACSDiva 8.0.1

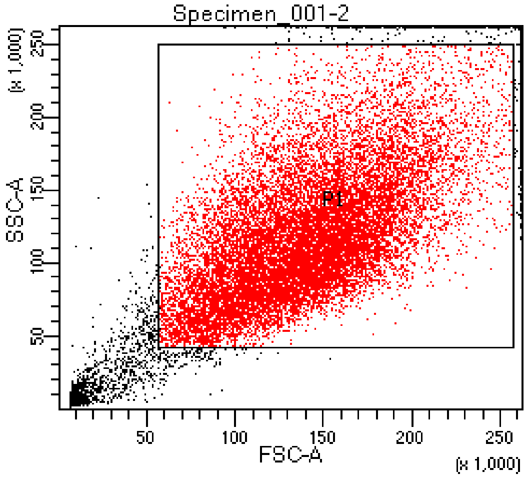

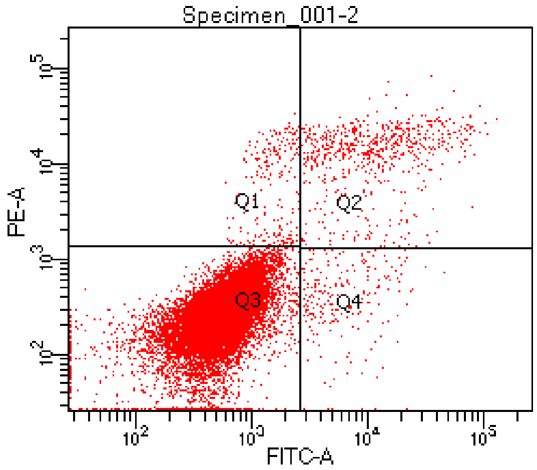

Experiment Name:	20220516-CL
Specimen Name:	Specimen_001
Tube Name:	2
Record Date:	May 16, 2022 2:27:15 PM
$OP:	Administrator
GUID:	4c77ee03-13e4-43b1-a825-520...

Population	#Events	%Parent	FITC-A Mean	PE-A Mean
All Events	20,000	####	1,508	1,110
Q1	356	1.8	1,562	8,639
Q2	958	4.8	15,997	14,046
Q3	18,272	91.4	621	297
Q4	414	2.1	7,109	584
P1	17,392	87.0	1,333	968
