# Supplementary material for: LncRNA-AC009948.5 promotes invasion and metastasis of lung adenocarcinoma by binding to miR-186-5p
Source: Front Oncol. 2022 Aug 19;12:949951. doi: 10.3389/fonc.2022.949951 (PMC9437580; doi:10.3389/fonc.2022.949951)
Supplement: Supplementary file 7 [file DataSheet_4.zip › Data Sheet 4/FigS1B/AC009948.5-2-3/Scrambled-3.pdf]

BD FACSDiva 8.0.1

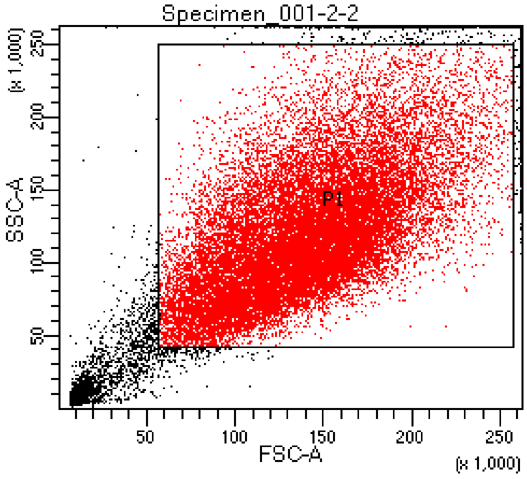

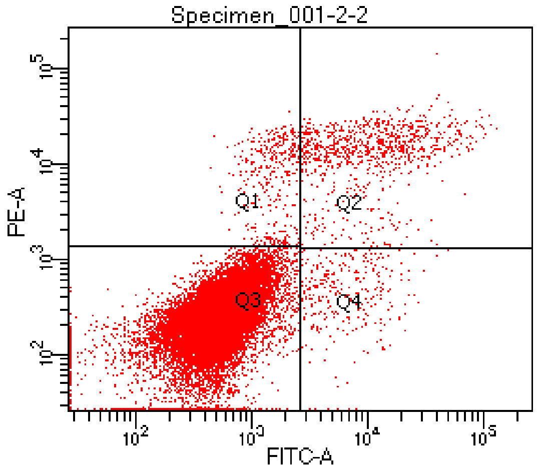

Experiment Name:	20220516-CL
Specimen Name:	Specimen_001
Tube Name:	2-2
Record Date:	May 16, 2022 2:29:43 PM
$OP:	Administrator
GUID:	bd744a92-1bdf-4598-8c18-a65...

Population	#Events	%Parent	FITC-A Mean	PE-A Mean
All Events	30,000	####	1,424	1,109
Q1	616	2.1	1,548	9,033
Q2	1,358	4.5	15,270	14,188
Q3	27,414	91.4	611	293
Q4	612	2.0	6,954	639
P1	25,938	86.5	1,239	967
