# Supplementary material for: LncRNA-AC009948.5 promotes invasion and metastasis of lung adenocarcinoma by binding to miR-186-5p
Source: Front Oncol. 2022 Aug 19;12:949951. doi: 10.3389/fonc.2022.949951 (PMC9437580; doi:10.3389/fonc.2022.949951)
Supplement: Supplementary file 7 [file DataSheet_4.zip › Data Sheet 4/FigS1B/AC009948.5-2-3/SiAC009948.5-2.pdf]

BD FACSDiva 8.0.1

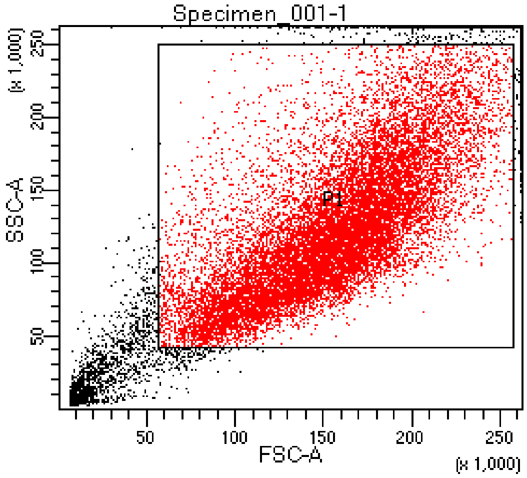

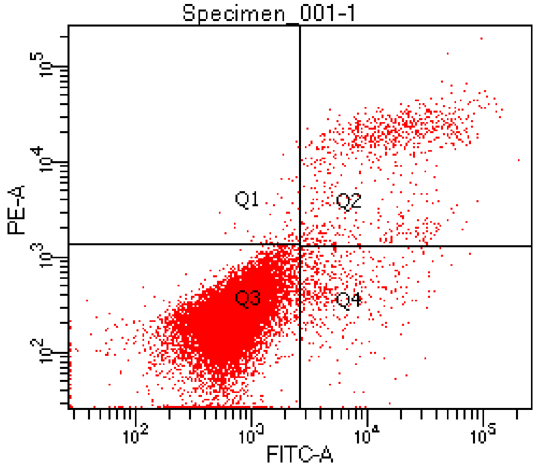

Experiment Name:	20220516-CL
Specimen Name:	Specimen_001
Tube Name:	1
Record Date:	May 16, 2022 2:26:40 PM
$OP:	Administrator
GUID:	9941ddb8-5509-4ff4-a3b5-5ec0...

Population	#Events	%Parent	FITC-A Mean	PE-A Mean
All Events	20,000	####	2,691	1,343
Q1	95	0.5	1,795	3,645
Q2	1,344	6.7	21,832	15,539
Q3	17,355	86.8	808	283
Q4	1,206	6.0	8,531	605
P1	16,664	83.3	2,063	1,103
