# Supplementary material for: LncRNA-AC009948.5 promotes invasion and metastasis of lung adenocarcinoma by binding to miR-186-5p
Source: Front Oncol. 2022 Aug 19;12:949951. doi: 10.3389/fonc.2022.949951 (PMC9437580; doi:10.3389/fonc.2022.949951)
Supplement: Supplementary file 7 [file DataSheet_4.zip › Data Sheet 4/FigS1B/AC009948.5-2-3/SiAC009948.5-3.pdf]

BD FACSDiva 8.0.1

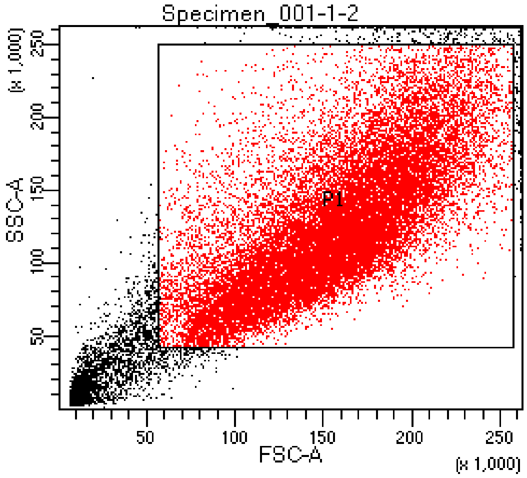

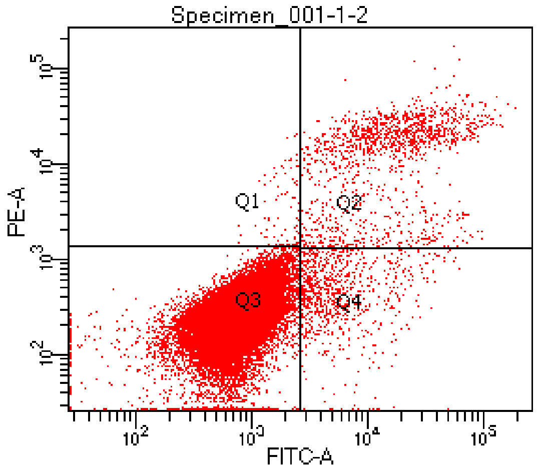

Experiment Name:	20220516-CL
Specimen Name:	Specimen_001
Tube Name:	1-2
Record Date:	May 16, 2022 2:29:08 PM
$OP:	Administrator
GUID:	4c95f11f-9b49-4681-b7e6-bea1...

Population	#Events	%Parent	FITC-A Mean	PE-A Mean
All Events	30,000	####	3,067	1,485
Q1	144	0.5	1,818	5,784
Q2	2,316	7.7	22,081	15,256
Q3	25,221	84.1	846	277
Q4	2,319	7.7	8,316	598
P1	24,449	81.5	2,337	1,240
