# Supplementary material for: LncRNA-AC009948.5 promotes invasion and metastasis of lung adenocarcinoma by binding to miR-186-5p
Source: Front Oncol. 2022 Aug 19;12:949951. doi: 10.3389/fonc.2022.949951 (PMC9437580; doi:10.3389/fonc.2022.949951)
Supplement: Supplementary file 7 [file DataSheet_4.zip › Data Sheet 4/FigS1B/AC009948.5-2-3/Specimen_001_FITC_16052022164938.pdf]

BD FACSDiva 8.0.1

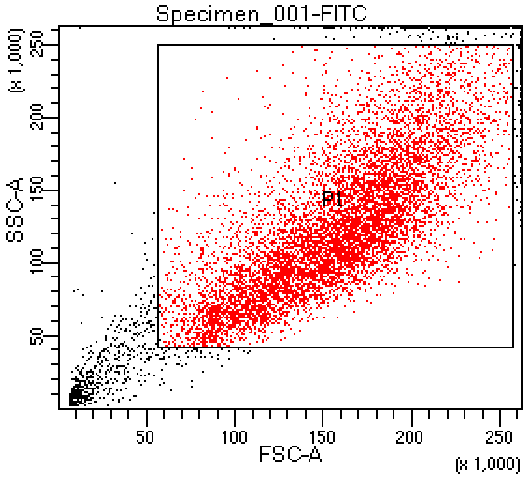

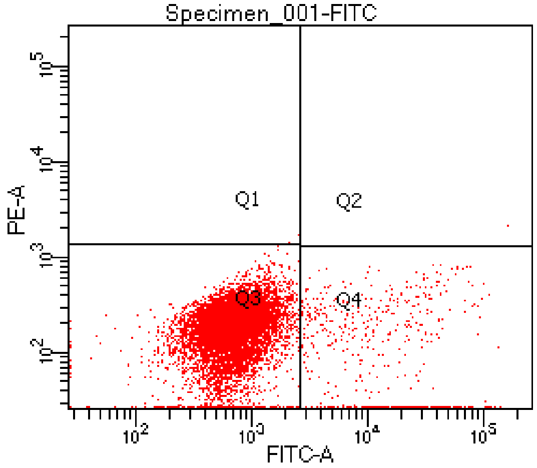

Experiment Name:	20220516-CL
Specimen Name:	Specimen_001
Tube Name:	FITC
Record Date:	May 16, 2022 2:25:26 PM
$OP:	Administrator
GUID:	74b175e6-0522-4f67-a6c9-798...

Population	#Events	%Parent	FITC-A Mean	PE-A Mean
All Events	10,000	####	2,582	224
Q1	3	0.0	2,323	1,517
Q2	11	0.1	64,598	25,278
Q3	9,019	90.2	798	197
Q4	967	9.7	18,515	183
P1	8,656	86.6	2,025	189
