# Supplementary material for: LncRNA-AC009948.5 promotes invasion and metastasis of lung adenocarcinoma by binding to miR-186-5p
Source: Front Oncol. 2022 Aug 19;12:949951. doi: 10.3389/fonc.2022.949951 (PMC9437580; doi:10.3389/fonc.2022.949951)
Supplement: Supplementary file 7 [file DataSheet_4.zip › Data Sheet 4/FigS1B/AC009948.5-2-3/Specimen_001_NC-2_16052022164956.pdf]

BD FACSDiva 8.0.1

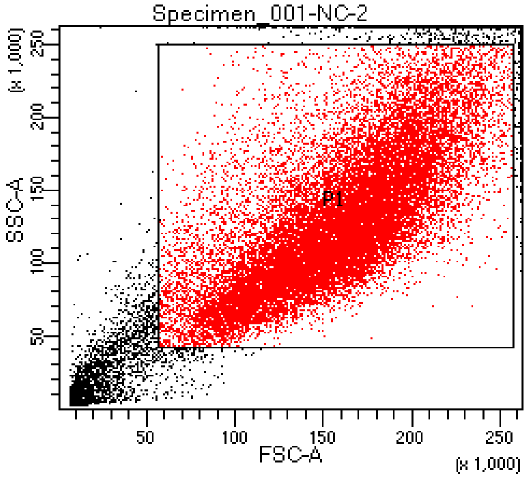

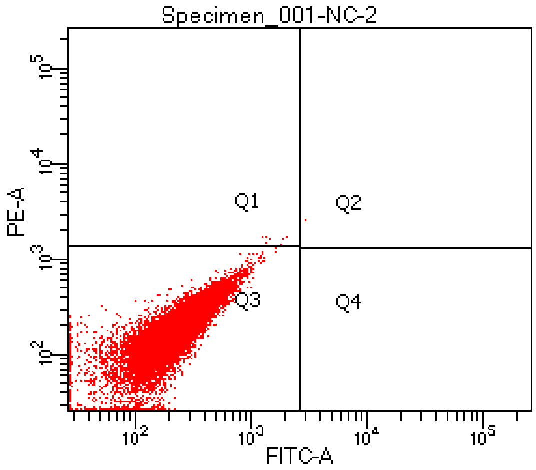

Experiment Name:	20220516-CL
Specimen Name:	Specimen_001
Tube Name:	NC-2
Record Date:	May 16, 2022 2:28:17 PM
$OP:	Administrator
GUID:	177f46f3-86ef-4989-8c13-64a6...

Population	#Events	%Parent	FITC-A Mean	PE-A Mean
All Events	30,000	####	280	239
Q1	60	0.2	1,775	1,625
Q2	10	0.0	27,926	35,621
Q3	29,930	99.8	267	224
Q4	0	0.0	####	####
P1	24,275	80.9	255	213
