# Supplementary material for: LncRNA-AC009948.5 promotes invasion and metastasis of lung adenocarcinoma by binding to miR-186-5p
Source: Front Oncol. 2022 Aug 19;12:949951. doi: 10.3389/fonc.2022.949951 (PMC9437580; doi:10.3389/fonc.2022.949951)
Supplement: Supplementary file 7 [file DataSheet_4.zip › Data Sheet 4/FigS1B/AC009948.5-2-3/Specimen_001_NC_16052022164923.pdf]

BD FACSDiva 8.0.1

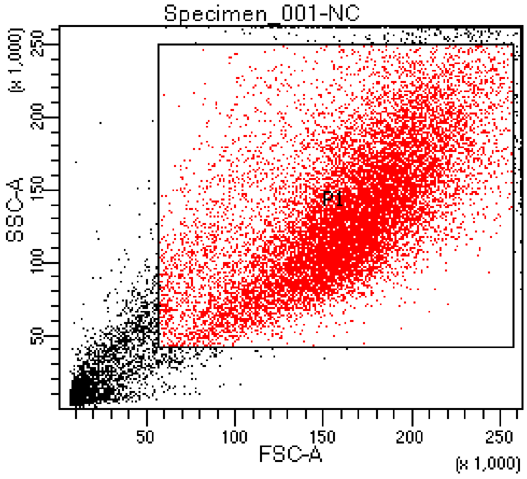

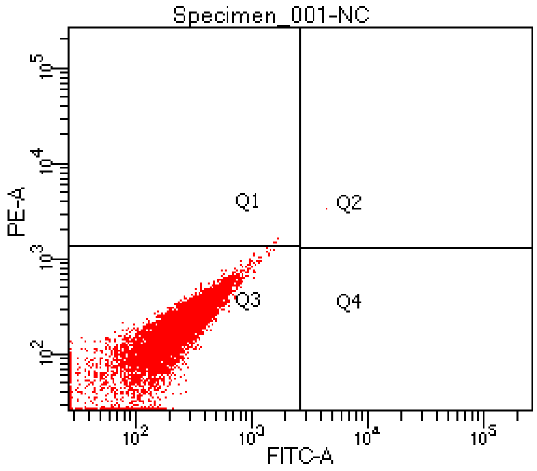

Experiment Name:	20220516-CL
Specimen Name:	Specimen_001
Tube Name:	NC
Record Date:	May 16, 2022 2:24:54 PM
$OP:	Administrator
GUID:	16ada050-4a33-455f-95d5-60c...

Population	#Events	%Parent	FITC-A Mean	PE-A Mean
All Events	20,000	####	281	233
Q1	112	0.6	1,945	1,675
Q2	25	0.1	4,301	4,301
Q3	19,863	99.3	267	220
Q4	0	0.0	####	####
P1	14,301	71.5	249	205
