# Supplementary material for: LncRNA-AC009948.5 promotes invasion and metastasis of lung adenocarcinoma by binding to miR-186-5p
Source: Front Oncol. 2022 Aug 19;12:949951. doi: 10.3389/fonc.2022.949951 (PMC9437580; doi:10.3389/fonc.2022.949951)
Supplement: Supplementary file 7 [file DataSheet_4.zip › Data Sheet 4/FigS1B/AC009948.5-2-3/Specimen_001_PI_16052022164943.pdf]

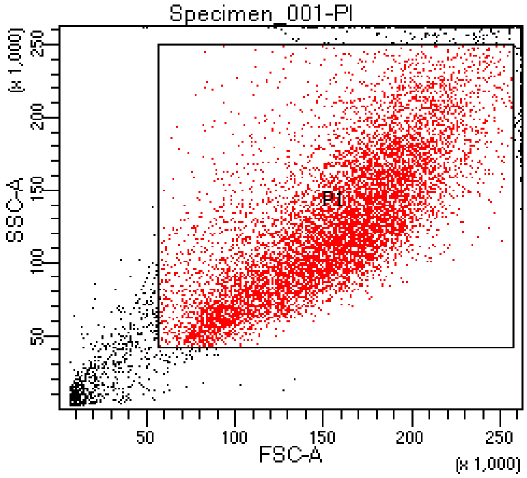

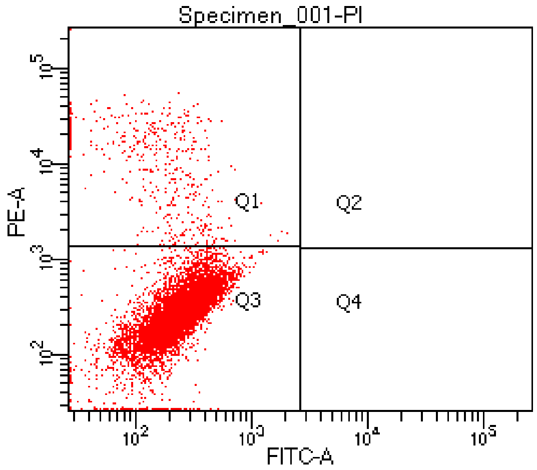

Experiment Name:	20220516-CL
Specimen Name:	Specimen_001
Tube Name:	PI
Record Date:	May 16, 2022 2:26:00 PM
$OP:	Administrator
GUID:	34f2bd5b-6ac2-4928-b2a2-8e3...

Population	#Events	%Parent	FITC-A Mean	PE-A Mean
All Events	10,000	####	260	1,291
Q1	731	7.3	237	13,535
Q2	3	0.0	10,381	12,366
Q3	9,266	92.7	259	322
Q4	0	0.0	####	####
P1	8,575	85.8	252	1,096
