# Supplementary figures and images for: LncRNA-AC009948.5 promotes invasion and metastasis of lung adenocarcinoma by binding to miR-186-5p (part 3 of 4)
Source: Front Oncol. 2022 Aug 19;12:949951. doi: 10.3389/fonc.2022.949951 (PMC9437580; doi:10.3389/fonc.2022.949951)

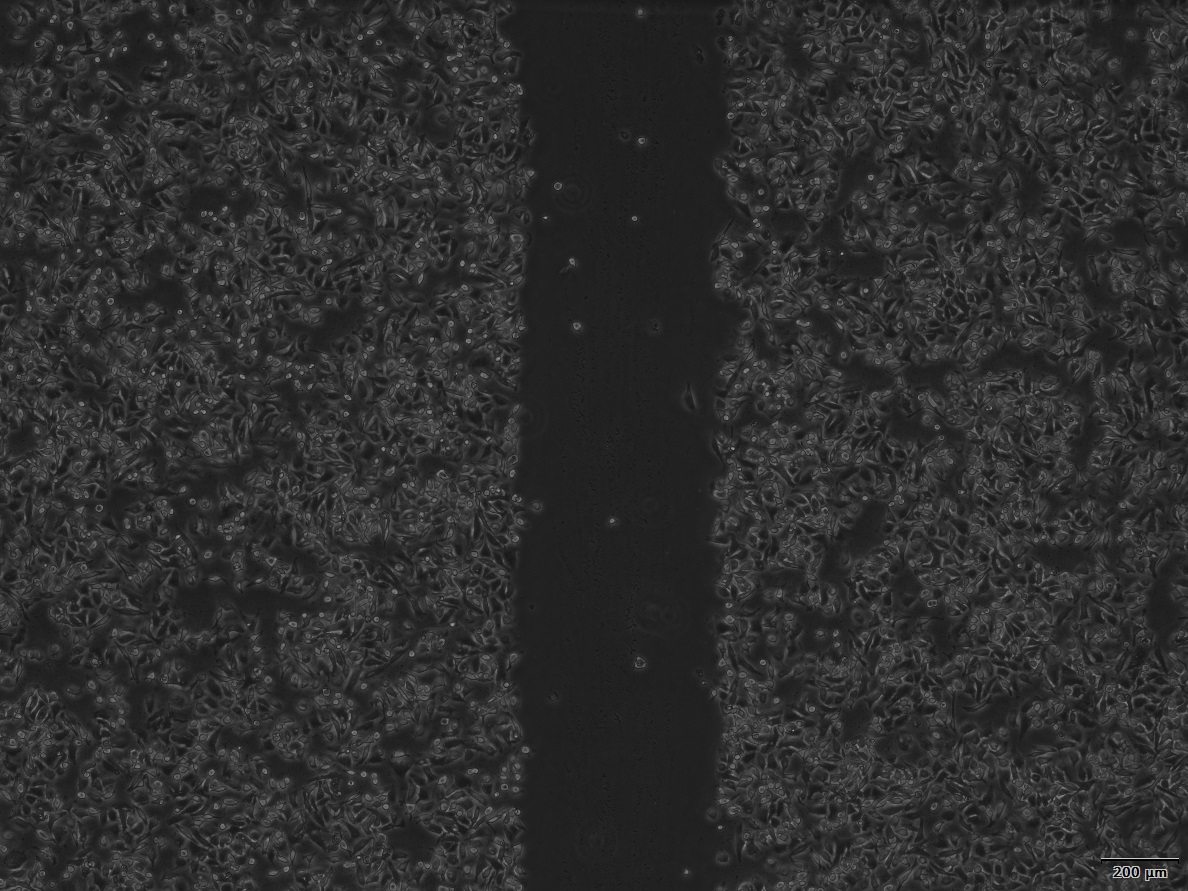

Supplement: Supplementary file 7 [file DataSheet_4.zip › Data Sheet 4/Fig4D/2-AC009948.5-sh-con-0H.jpg]

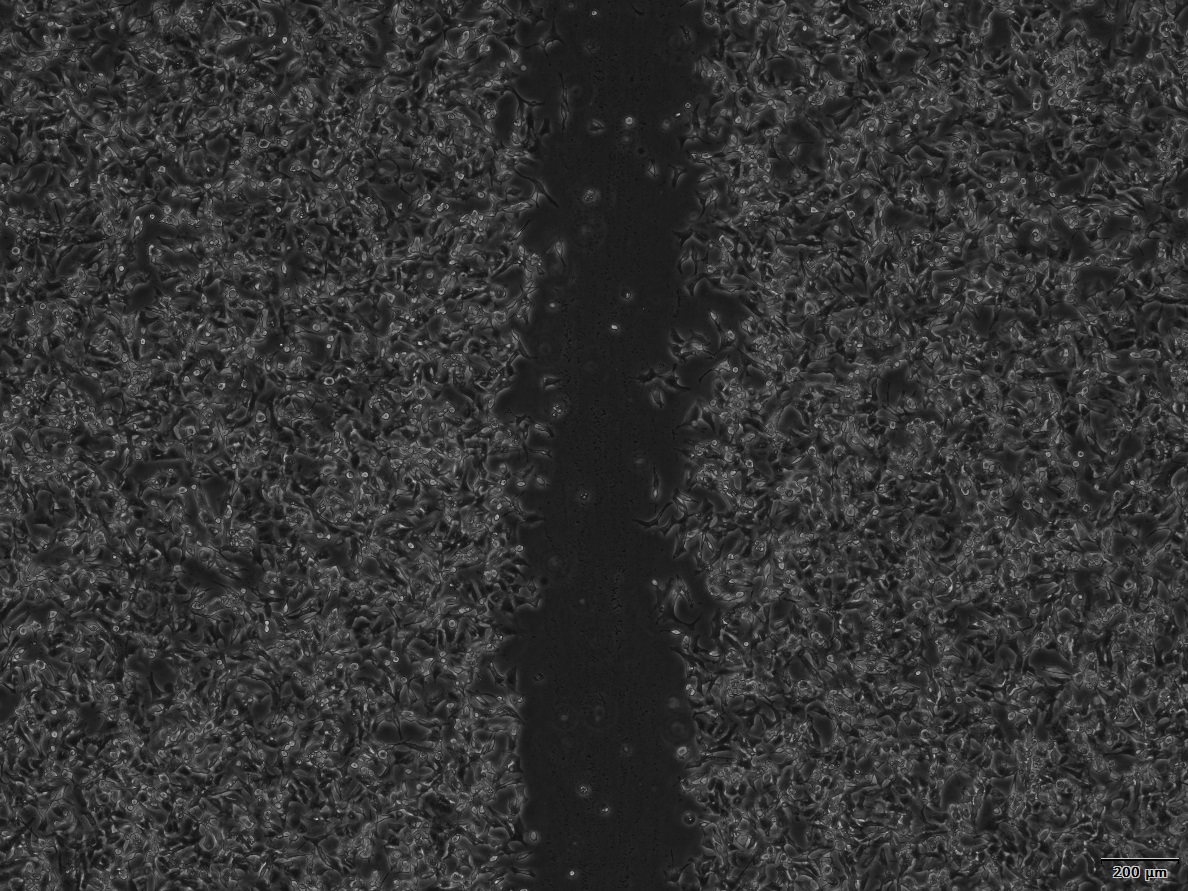

Supplement: Supplementary file 7 [file DataSheet_4.zip › Data Sheet 4/Fig4D/2-AC009948.5-sh-con-24H.jpg]

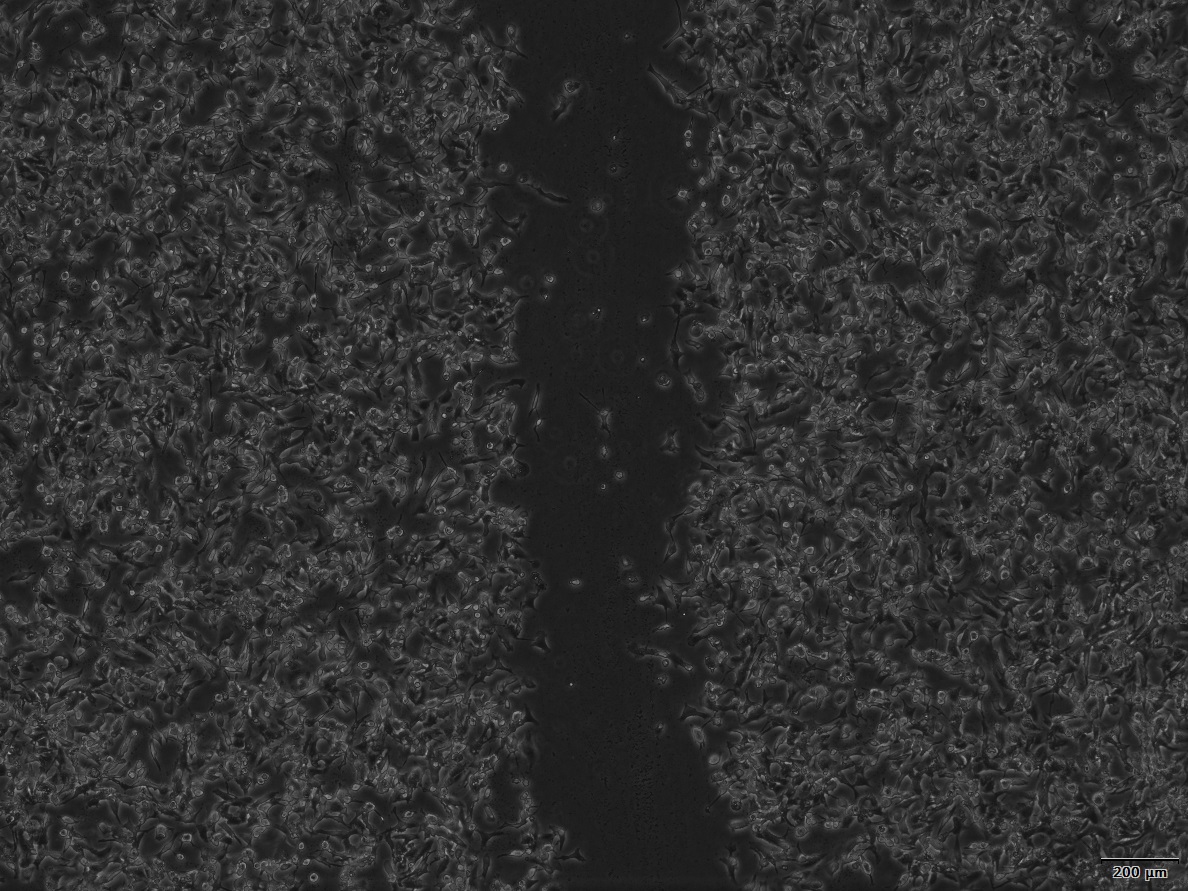

Supplement: Supplementary file 7 [file DataSheet_4.zip › Data Sheet 4/Fig4D/2-AC009948.5-sh186-24h.jpg]

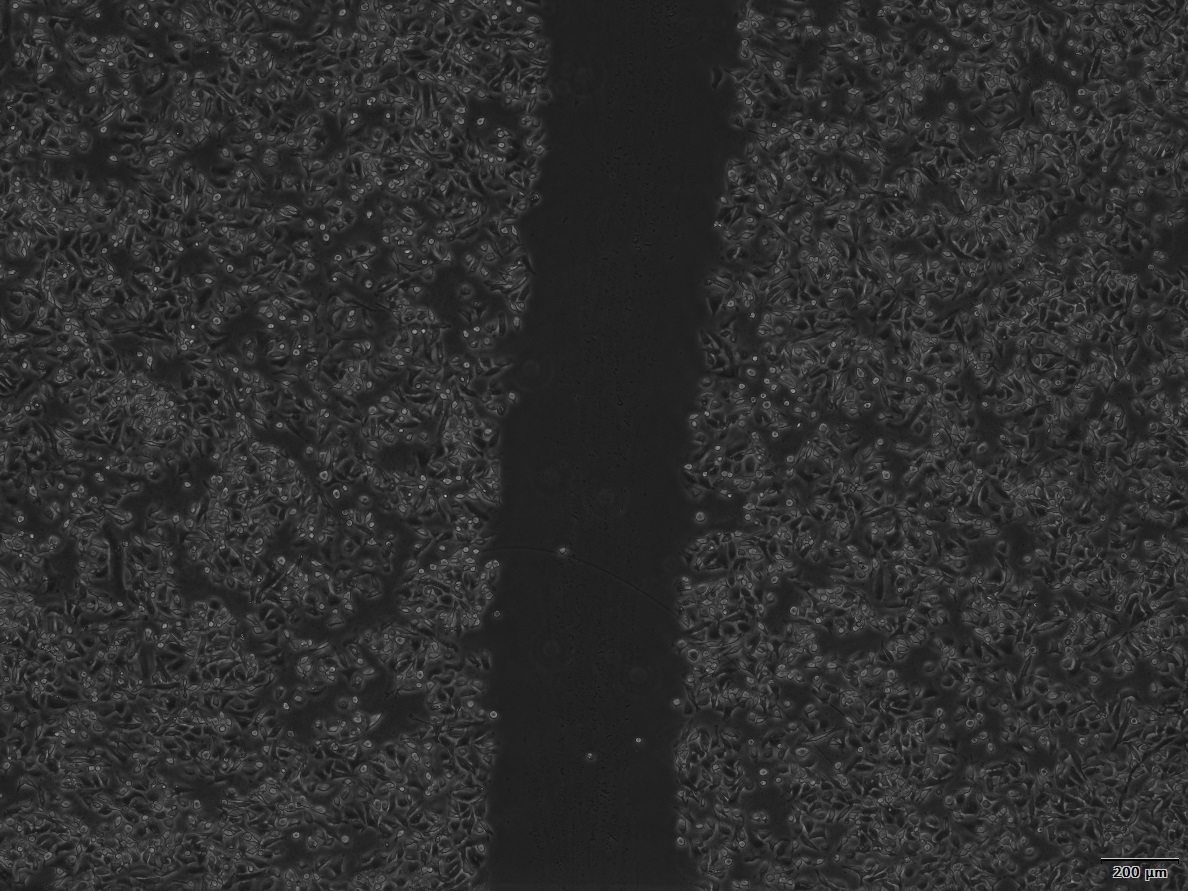

Supplement: Supplementary file 7 [file DataSheet_4.zip › Data Sheet 4/Fig4D/2-AC009958.5-over-186-0h.jpg]

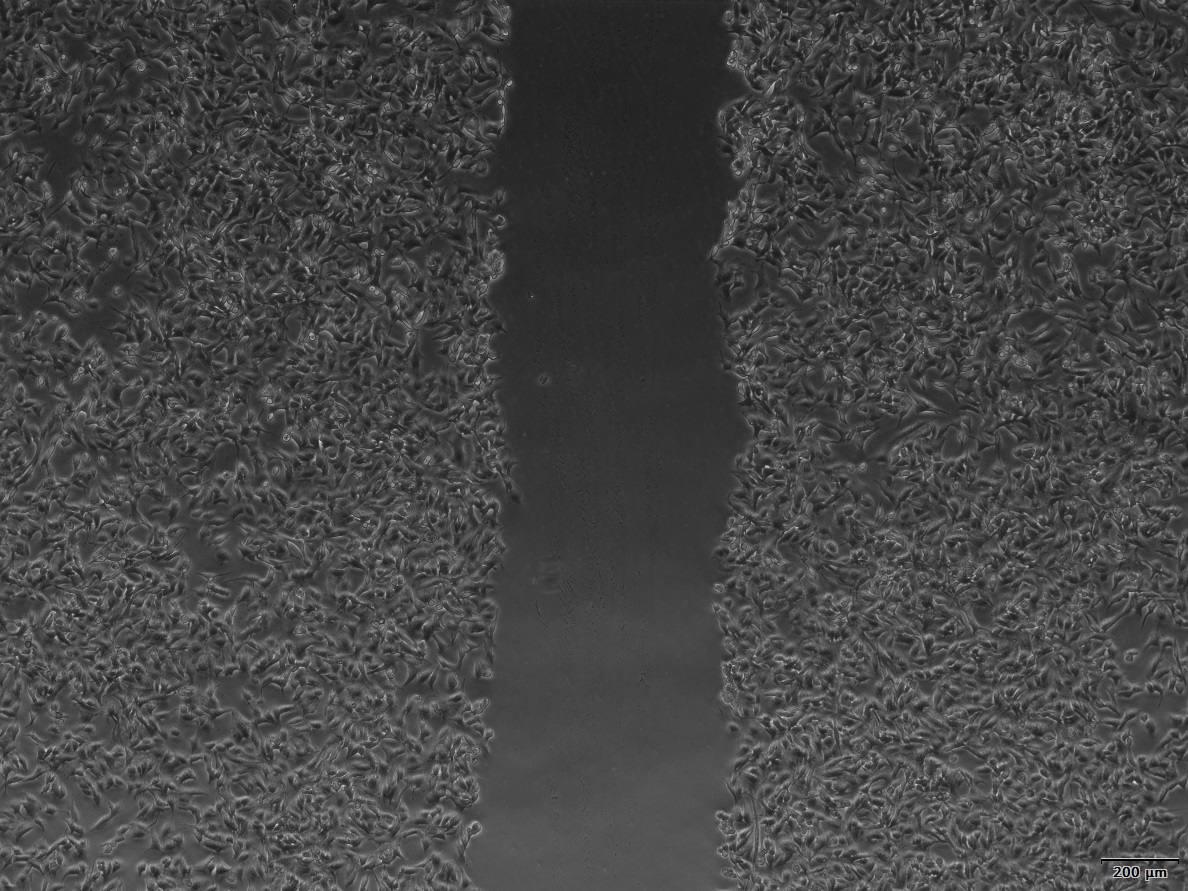

Supplement: Supplementary file 7 [file DataSheet_4.zip › Data Sheet 4/Fig4D/3-AC009948.5-CON-0H.jpg]

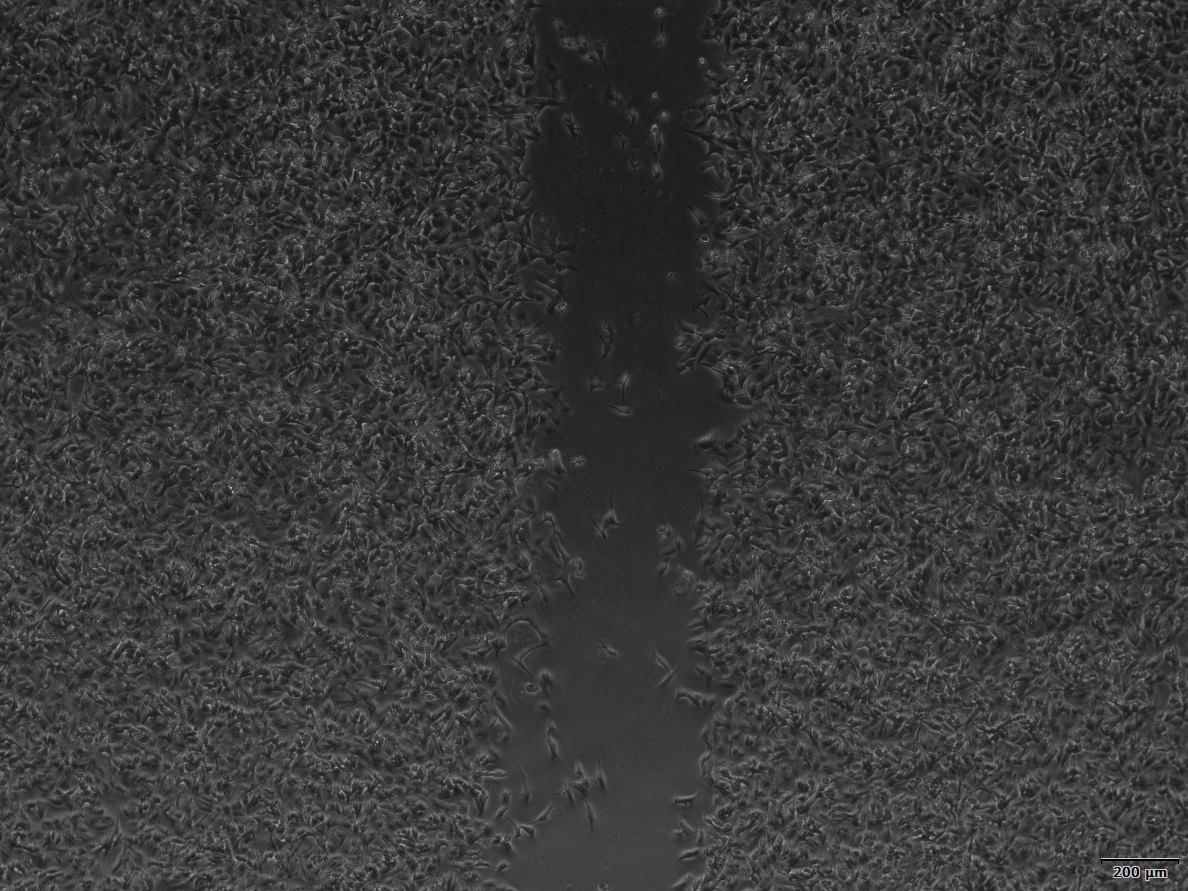

Supplement: Supplementary file 7 [file DataSheet_4.zip › Data Sheet 4/Fig4D/3-AC009948.5-CON-24h.jpg]

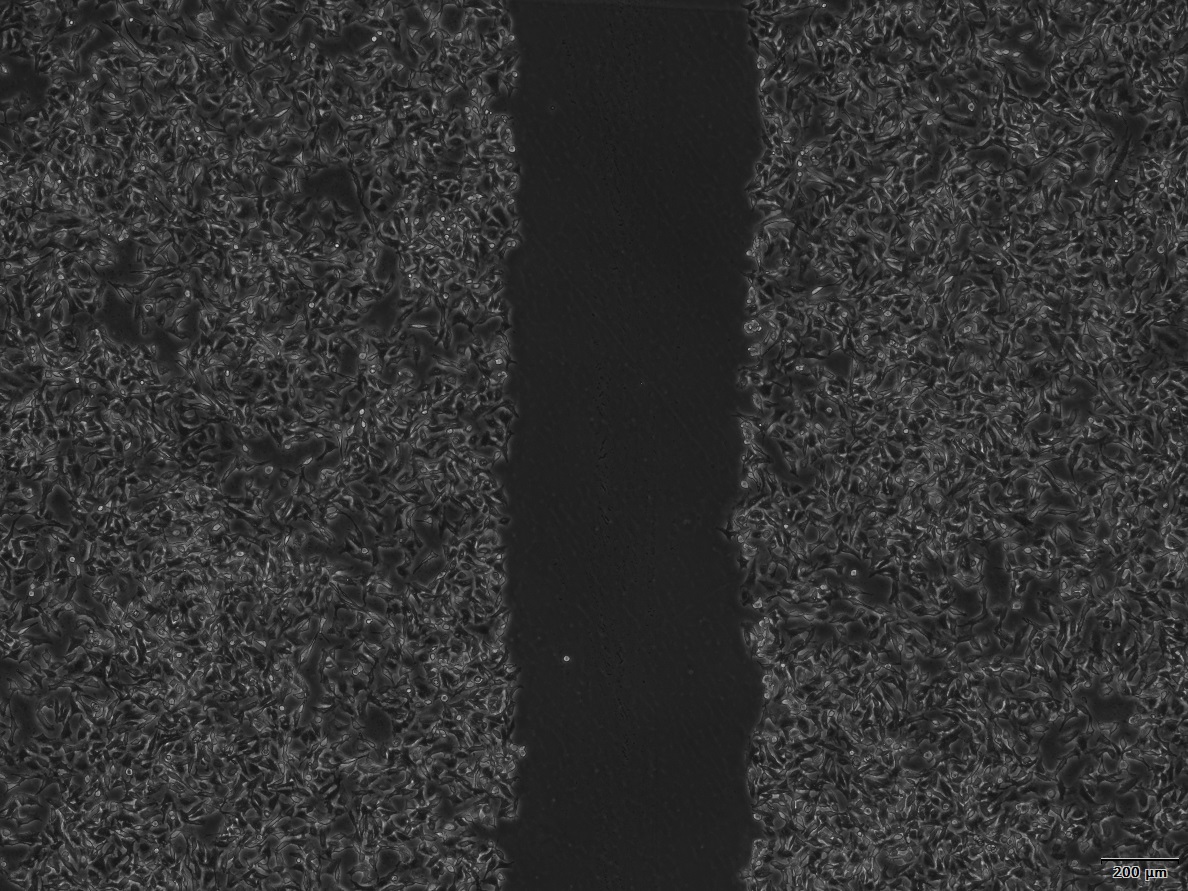

Supplement: Supplementary file 7 [file DataSheet_4.zip › Data Sheet 4/Fig4D/3-AC009948.5-COTRANS-0h.jpg]

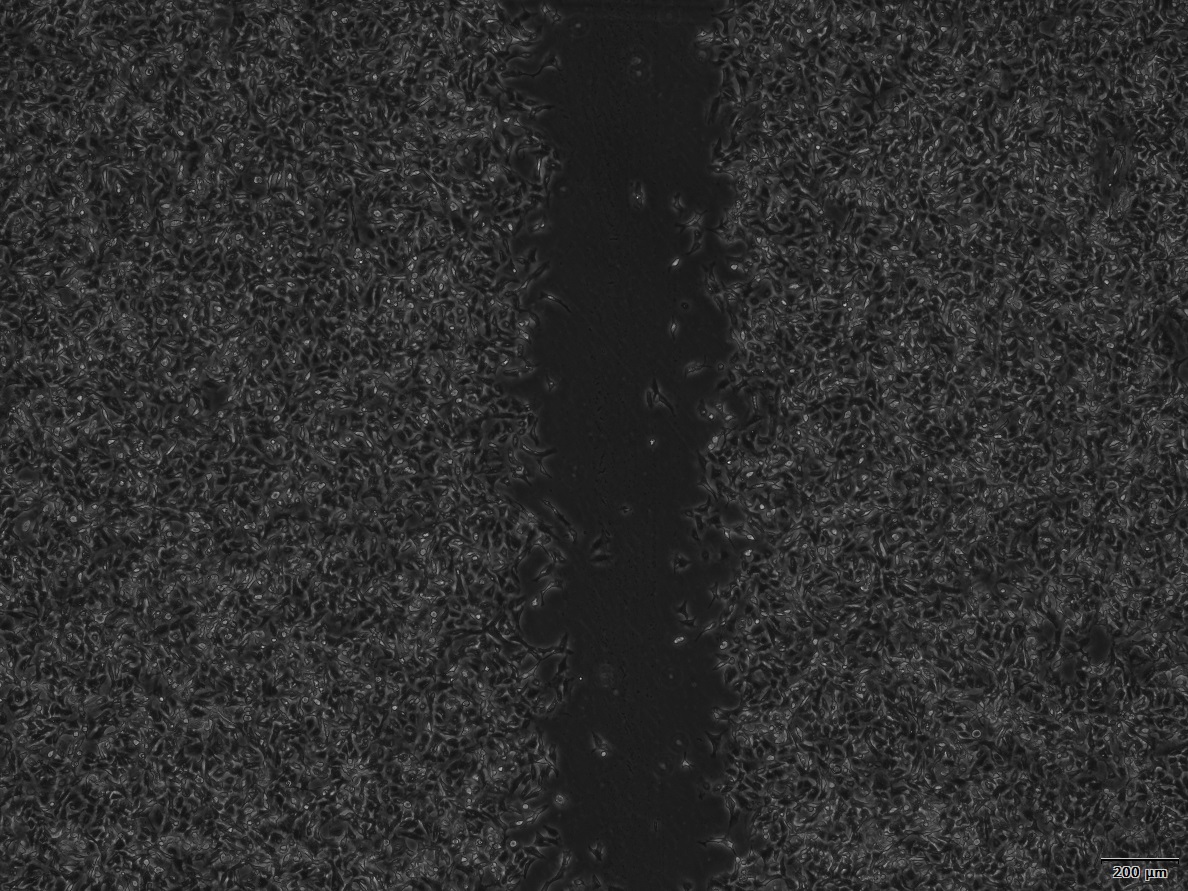

Supplement: Supplementary file 7 [file DataSheet_4.zip › Data Sheet 4/Fig4D/3-AC009948.5-COTRANS-24H.jpg]

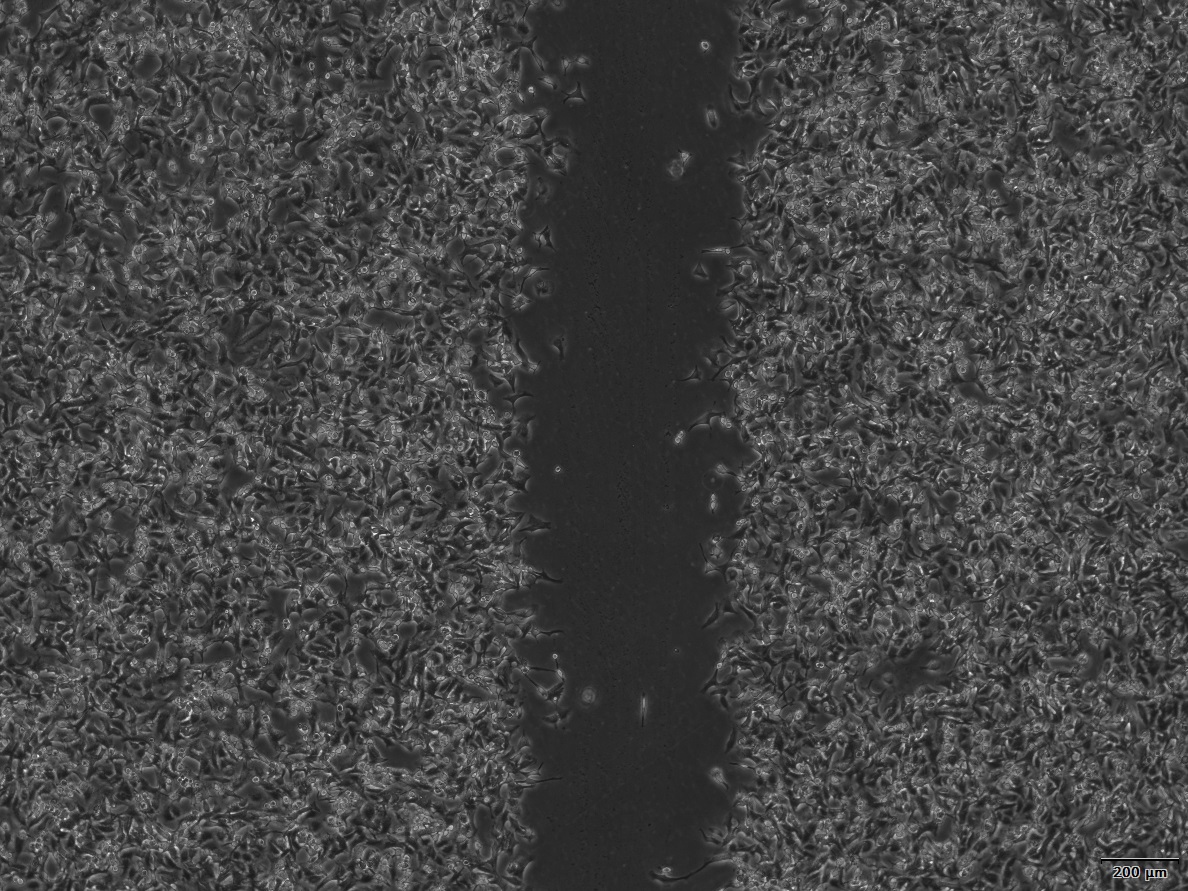

Supplement: Supplementary file 7 [file DataSheet_4.zip › Data Sheet 4/Fig4D/3-AC009948.5-over-186-24h.jpg]

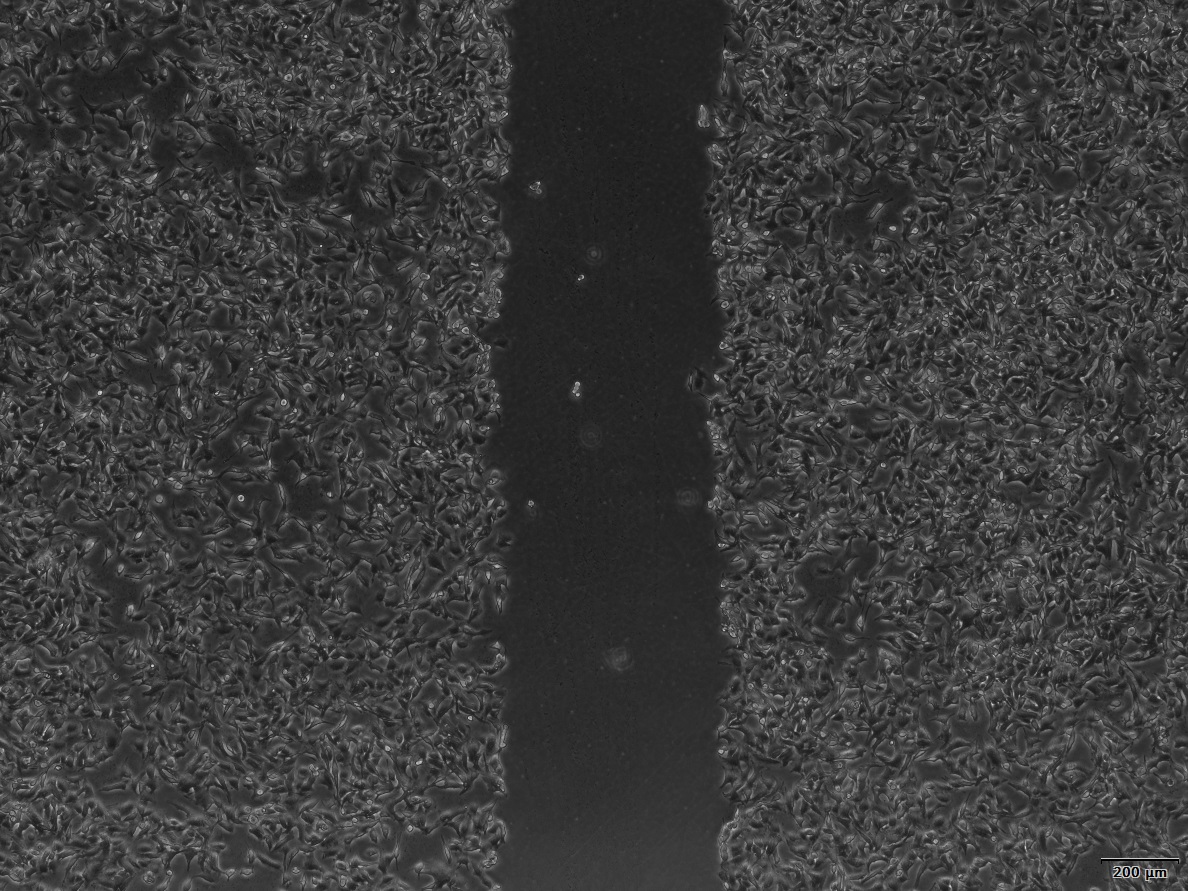

Supplement: Supplementary file 7 [file DataSheet_4.zip › Data Sheet 4/Fig4D/3-AC009948.5-Sh-186-0H.jpg]

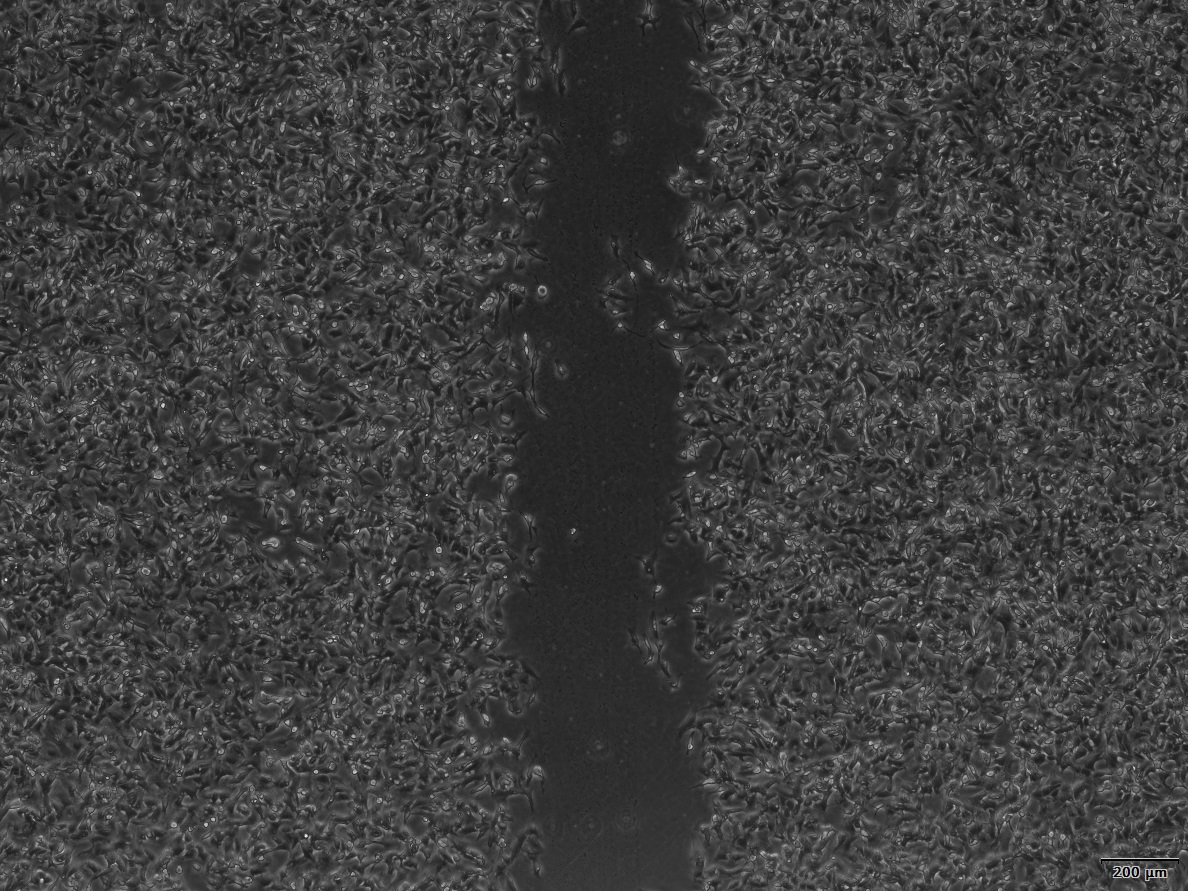

Supplement: Supplementary file 7 [file DataSheet_4.zip › Data Sheet 4/Fig4D/3-AC009948.5-Sh-186-24H.jpg]

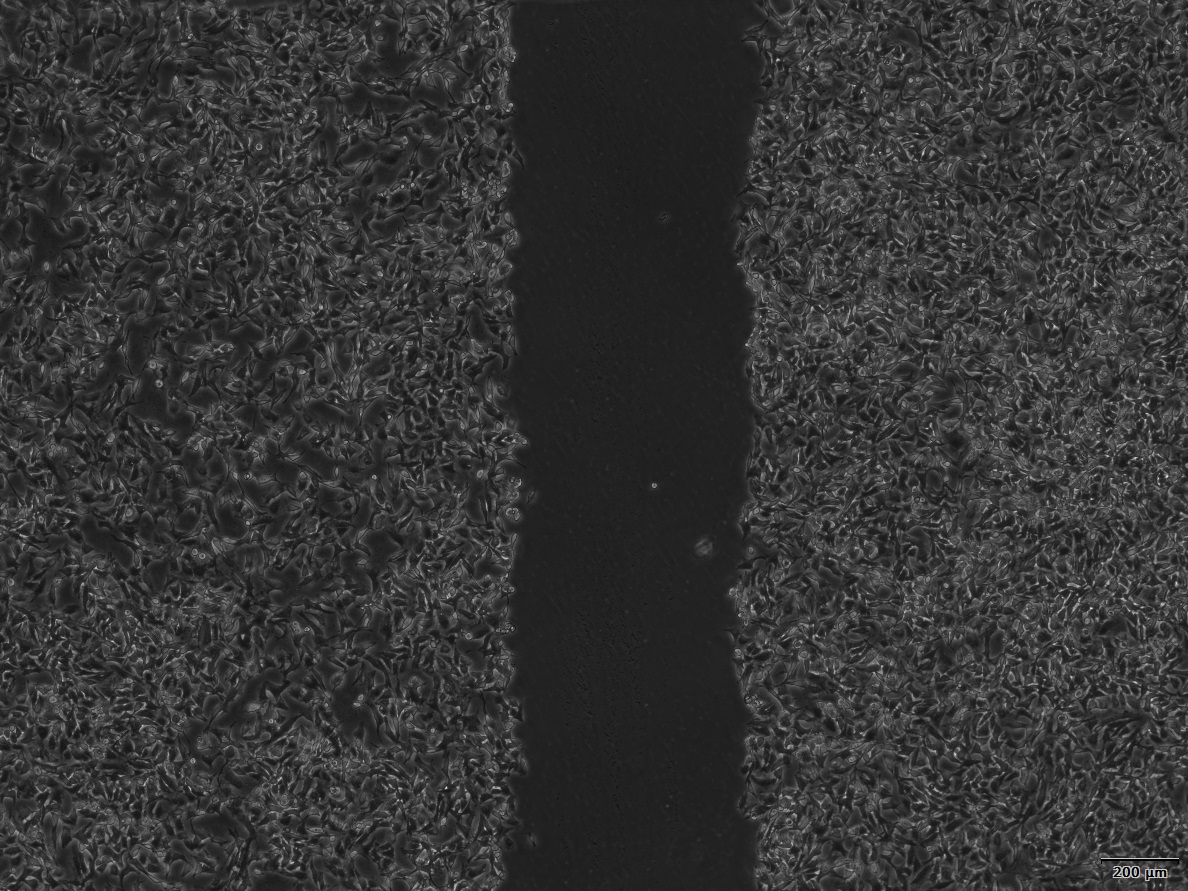

Supplement: Supplementary file 7 [file DataSheet_4.zip › Data Sheet 4/Fig4D/3-AC009948.5-sh-con-0H.jpg]

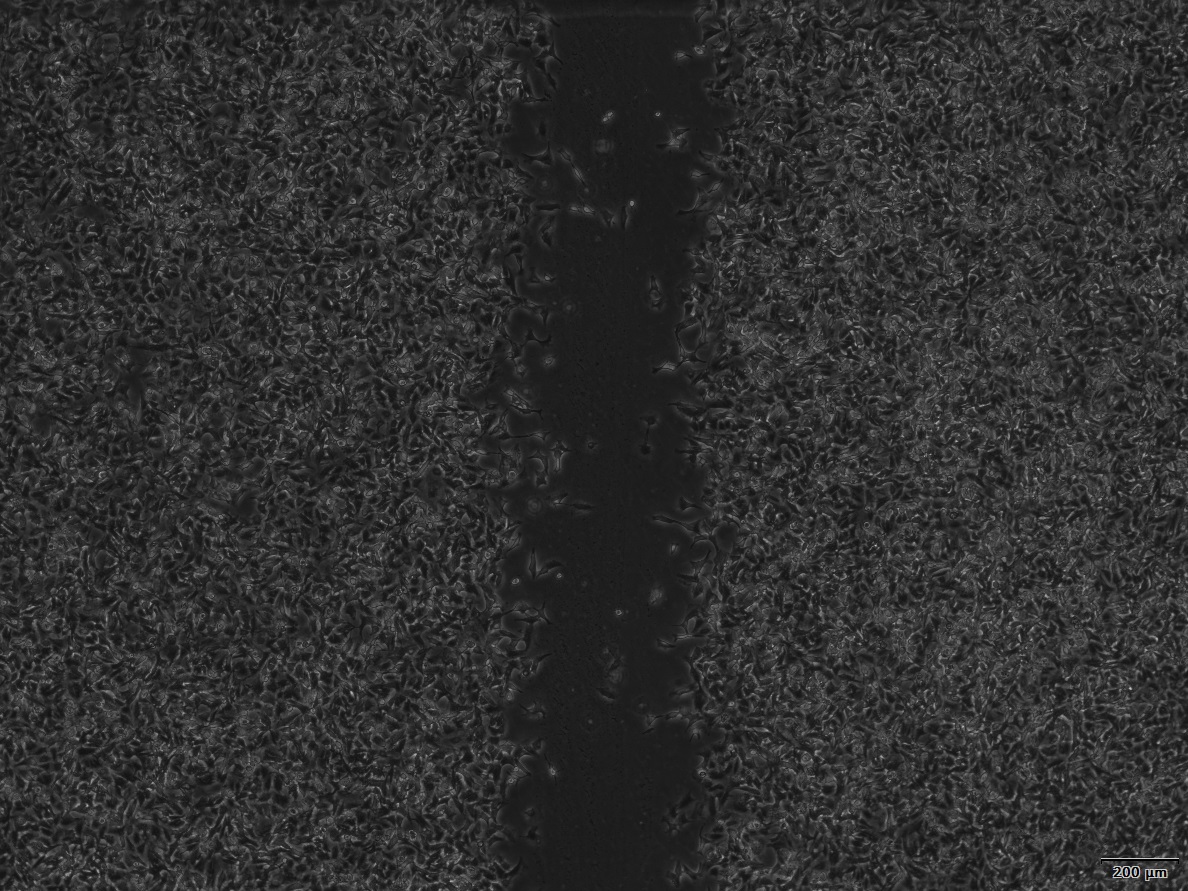

Supplement: Supplementary file 7 [file DataSheet_4.zip › Data Sheet 4/Fig4D/3-AC009948.5-sh-con-24H.jpg]

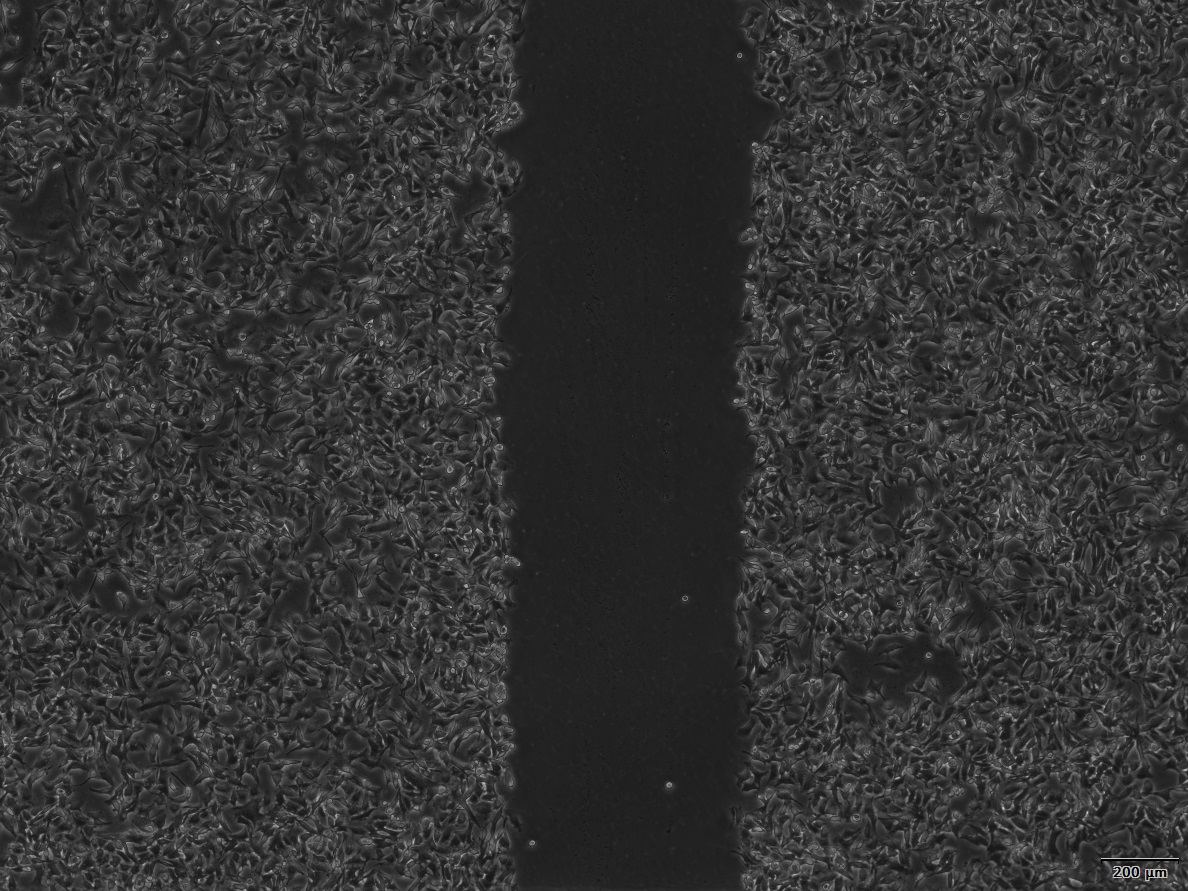

Supplement: Supplementary file 7 [file DataSheet_4.zip › Data Sheet 4/Fig4D/3-AC009958.5-over-186-0h.jpg]

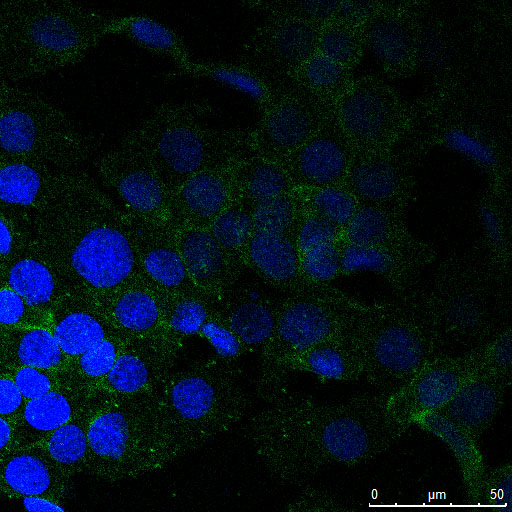

Supplement: Supplementary file 7 [file DataSheet_4.zip › Data Sheet 4/Fig4E/Ecadherin/1-AC009948.5-CO-ecadherin.jpg]

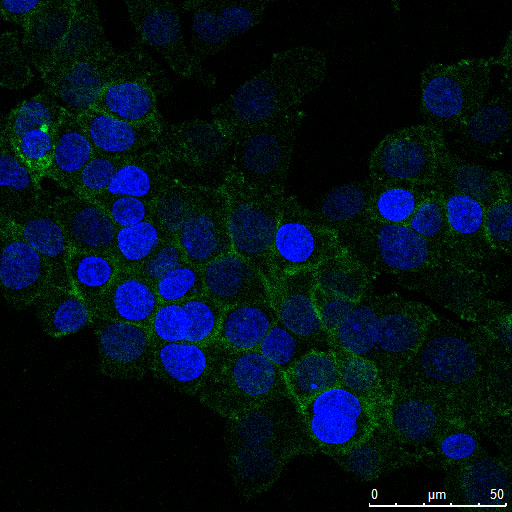

Supplement: Supplementary file 7 [file DataSheet_4.zip › Data Sheet 4/Fig4E/Ecadherin/1-AC009948.5-con-ecadherin.jpg]

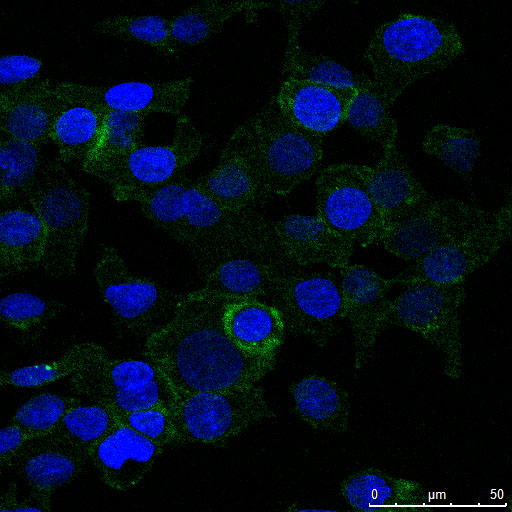

Supplement: Supplementary file 7 [file DataSheet_4.zip › Data Sheet 4/Fig4E/Ecadherin/1-AC009948.5-ecadherin-scrambled.jpg]

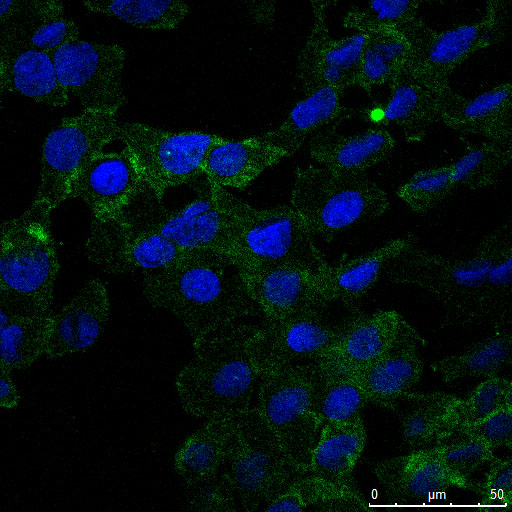

Supplement: Supplementary file 7 [file DataSheet_4.zip › Data Sheet 4/Fig4E/Ecadherin/1-AC009948.5-over-186-ecadherin.jpg]

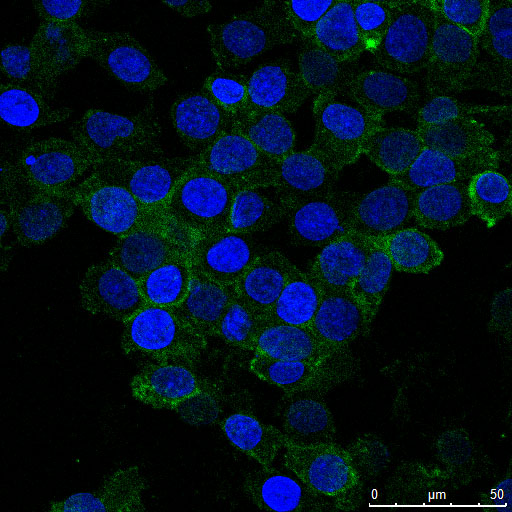

Supplement: Supplementary file 7 [file DataSheet_4.zip › Data Sheet 4/Fig4E/Ecadherin/1-siAC009948.5-ecadherin.jpg]

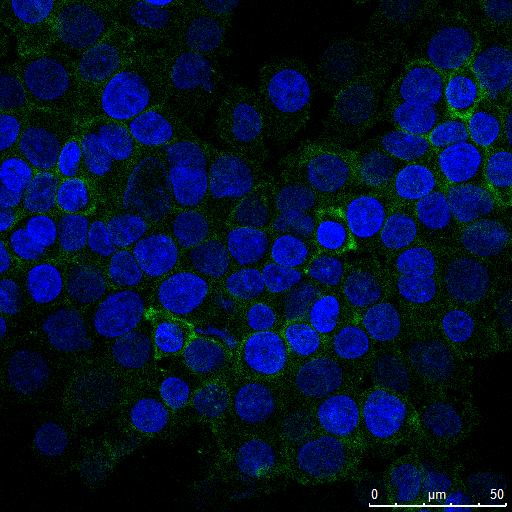

Supplement: Supplementary file 7 [file DataSheet_4.zip › Data Sheet 4/Fig4E/Ecadherin/2-AC009948.5-CO-ecadherin.jpg]

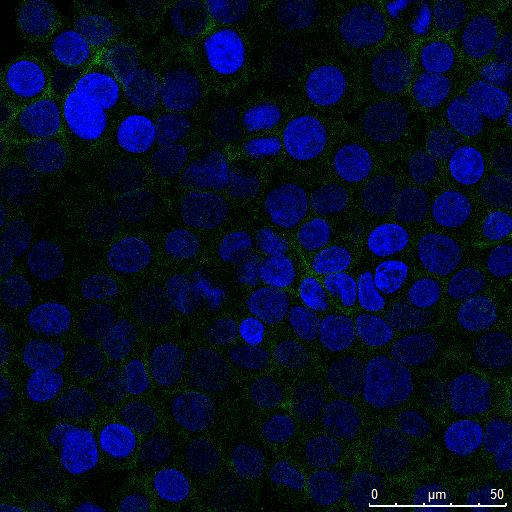

Supplement: Supplementary file 7 [file DataSheet_4.zip › Data Sheet 4/Fig4E/Ecadherin/2-AC009948.5-con-ecadherin.jpg]

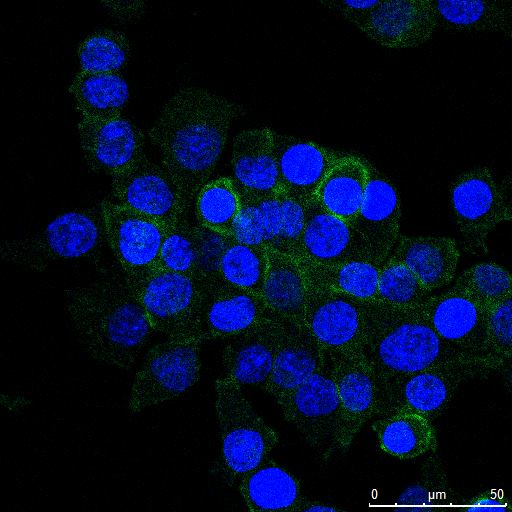

Supplement: Supplementary file 7 [file DataSheet_4.zip › Data Sheet 4/Fig4E/Ecadherin/2-AC009948.5-ecadherin-scrambled.jpg]

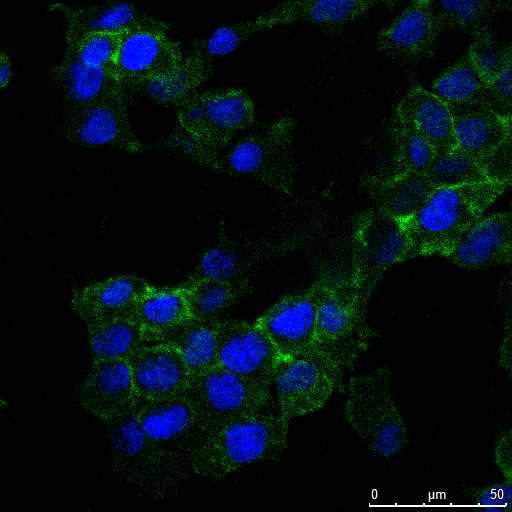

Supplement: Supplementary file 7 [file DataSheet_4.zip › Data Sheet 4/Fig4E/Ecadherin/2-AC009948.5-over-186-ecadherin.jpg]

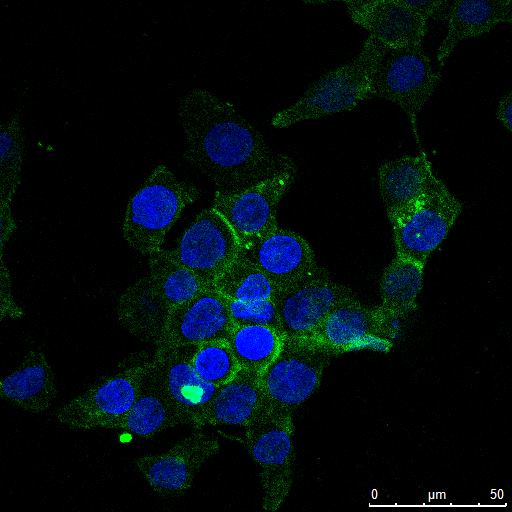

Supplement: Supplementary file 7 [file DataSheet_4.zip › Data Sheet 4/Fig4E/Ecadherin/2-siAC009948.5-ecadherin.jpg]

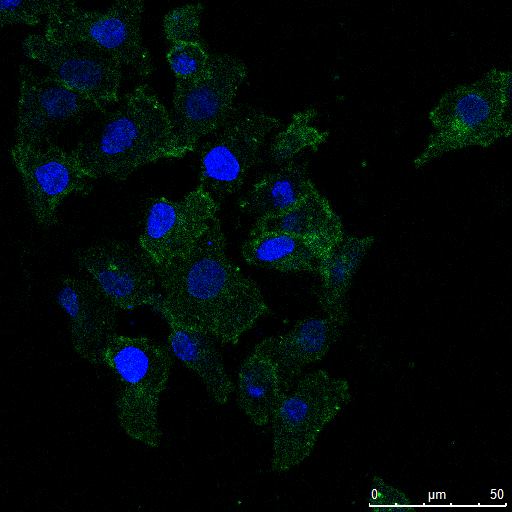

Supplement: Supplementary file 7 [file DataSheet_4.zip › Data Sheet 4/Fig4E/Ecadherin/3-AC009948.5-CO-ecadherin.jpg]

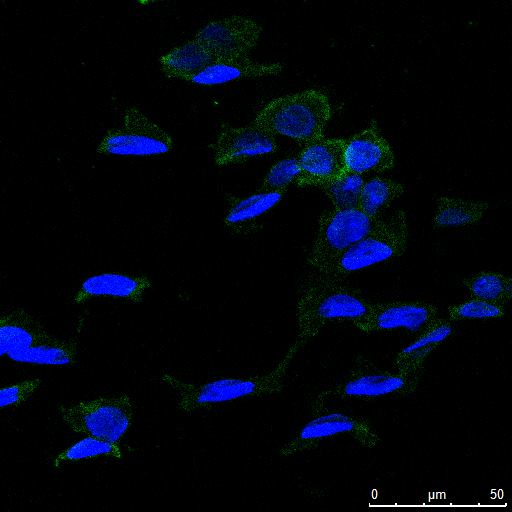

Supplement: Supplementary file 7 [file DataSheet_4.zip › Data Sheet 4/Fig4E/Ecadherin/3-AC009948.5-con-ecadherin.jpg]

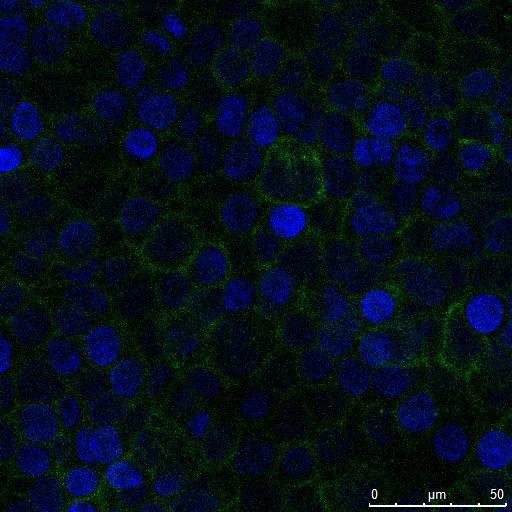

Supplement: Supplementary file 7 [file DataSheet_4.zip › Data Sheet 4/Fig4E/Ecadherin/3-AC009948.5-ecadherin-scrambled.jpg]

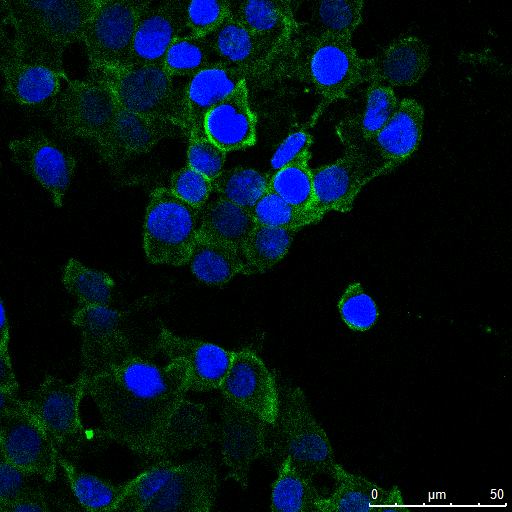

Supplement: Supplementary file 7 [file DataSheet_4.zip › Data Sheet 4/Fig4E/Ecadherin/3-AC009948.5-over-186-ecadherin.jpg]

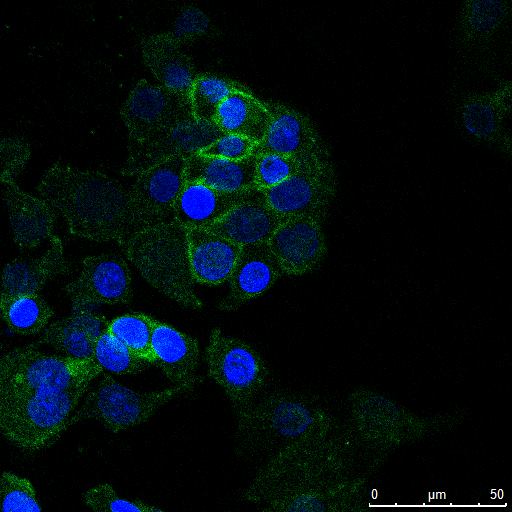

Supplement: Supplementary file 7 [file DataSheet_4.zip › Data Sheet 4/Fig4E/Ecadherin/3-siAC009948.5-ecadherin.jpg]

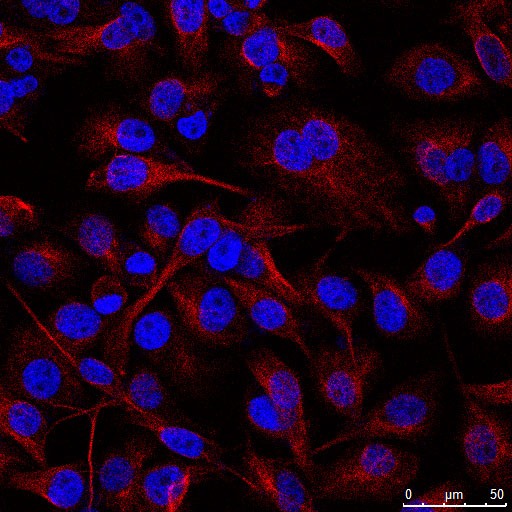

Supplement: Supplementary file 7 [file DataSheet_4.zip › Data Sheet 4/Fig4E/Vimentin/1-AC009948.5-CO-vimentin.jpg]

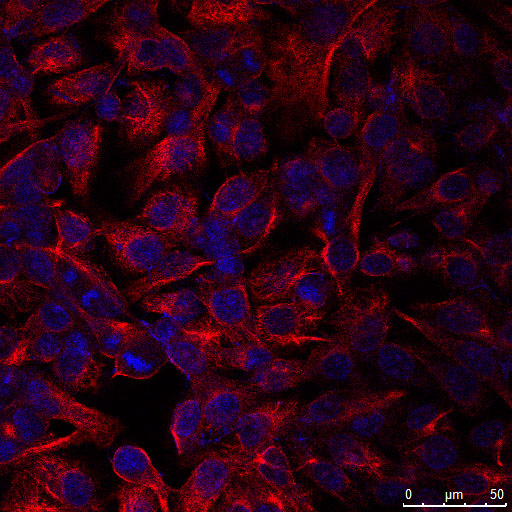

Supplement: Supplementary file 7 [file DataSheet_4.zip › Data Sheet 4/Fig4E/Vimentin/1-AC009948.5-con-vimentin-Series035_z0.jpg]

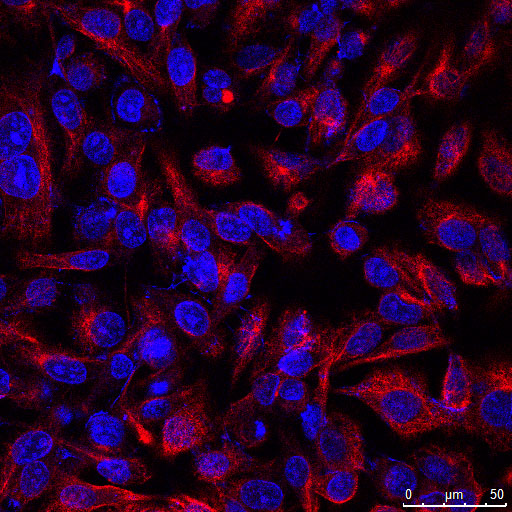

Supplement: Supplementary file 7 [file DataSheet_4.zip › Data Sheet 4/Fig4E/Vimentin/1-AC009948.5-scrambled-vimentin_Series029_z0.jpg]

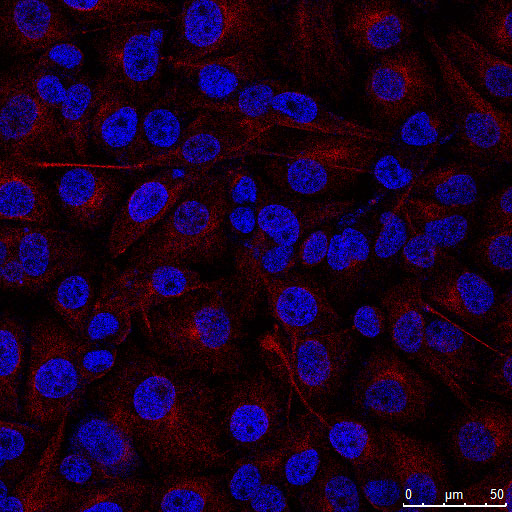

Supplement: Supplementary file 7 [file DataSheet_4.zip › Data Sheet 4/Fig4E/Vimentin/1-over-186-vimentin.jpg]

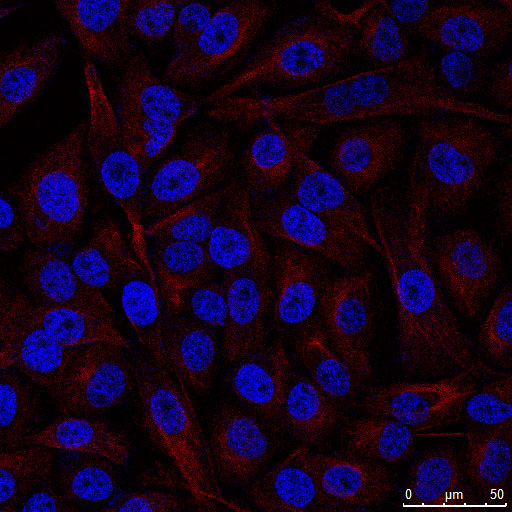

Supplement: Supplementary file 7 [file DataSheet_4.zip › Data Sheet 4/Fig4E/Vimentin/1-SiAC009948.5-vimentin-Series005_z0 -.jpg]

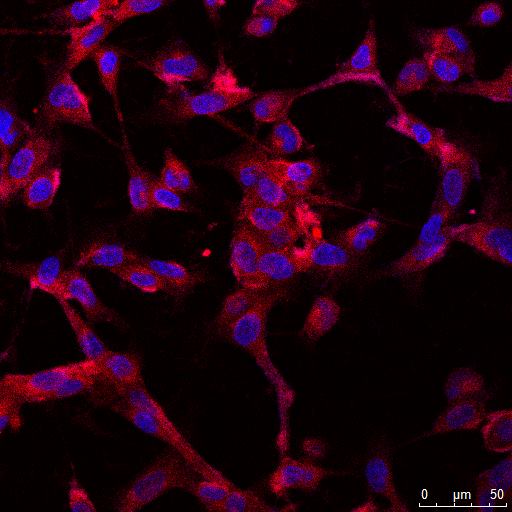

Supplement: Supplementary file 7 [file DataSheet_4.zip › Data Sheet 4/Fig4E/Vimentin/2-AC009948.5-CO-vimentin.jpg]

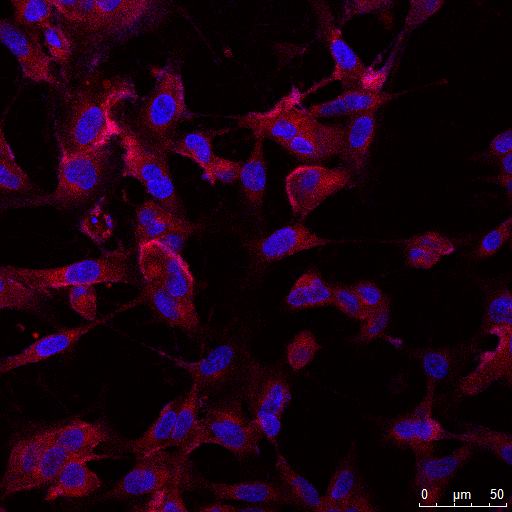

Supplement: Supplementary file 7 [file DataSheet_4.zip › Data Sheet 4/Fig4E/Vimentin/2-AC009948.5-con-vimentin-Series035_z0.jpg]

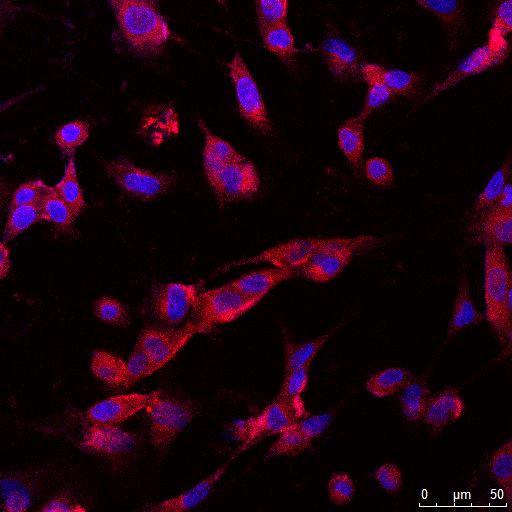

Supplement: Supplementary file 7 [file DataSheet_4.zip › Data Sheet 4/Fig4E/Vimentin/2-AC009948.5-scrambled-vimentin_Series029_z0.jpg]

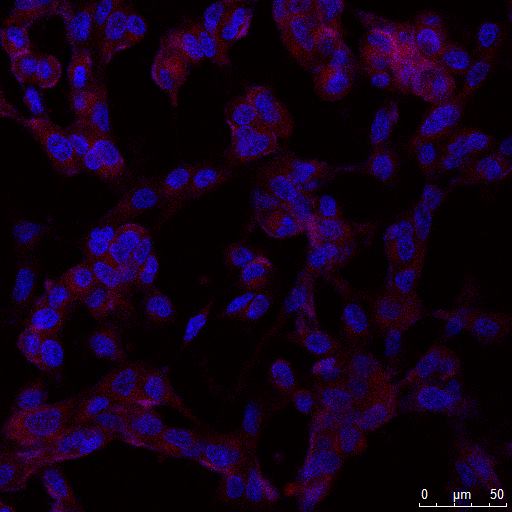

Supplement: Supplementary file 7 [file DataSheet_4.zip › Data Sheet 4/Fig4E/Vimentin/2-over-186-vimentin.jpg]

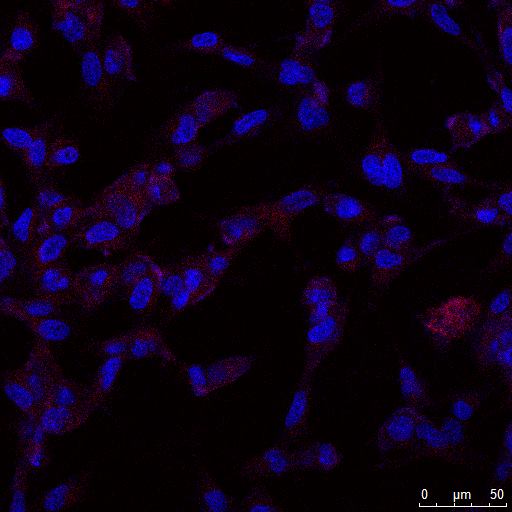

Supplement: Supplementary file 7 [file DataSheet_4.zip › Data Sheet 4/Fig4E/Vimentin/2-SiAC009948.5-vimentin-Series005_z0 -.jpg]

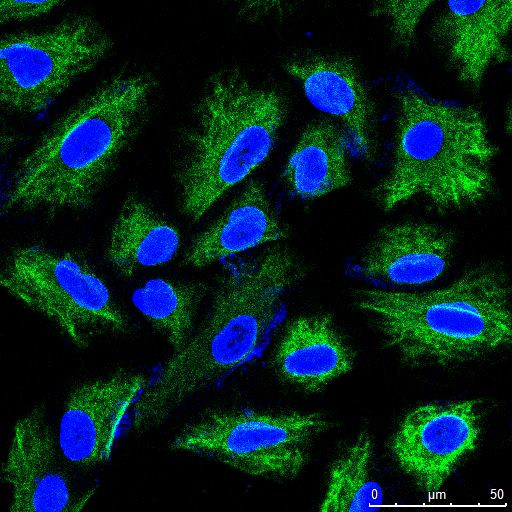

Supplement: Supplementary file 7 [file DataSheet_4.zip › Data Sheet 4/Fig4E/Vimentin/3-AC009948.5-CO-vimentin.jpg]

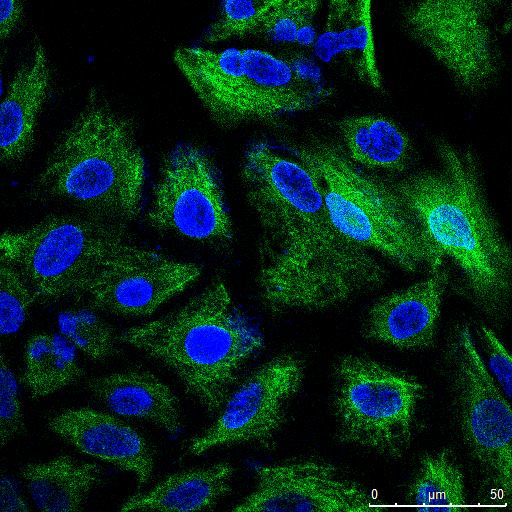

Supplement: Supplementary file 7 [file DataSheet_4.zip › Data Sheet 4/Fig4E/Vimentin/3-AC009948.5-con-vimentin-Series035_z0.jpg]

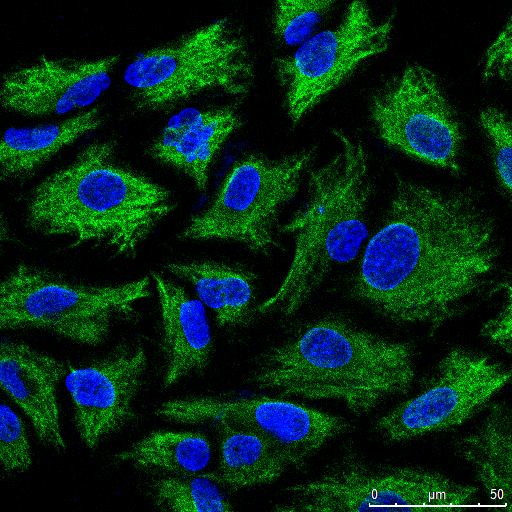

Supplement: Supplementary file 7 [file DataSheet_4.zip › Data Sheet 4/Fig4E/Vimentin/3-AC009948.5-scrambled-vimentin_Series029_z0.jpg]

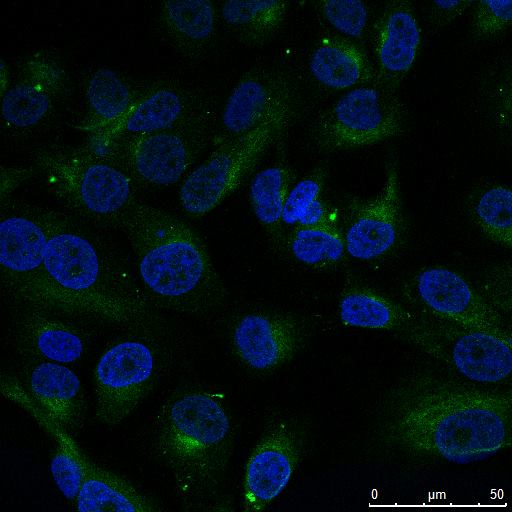

Supplement: Supplementary file 7 [file DataSheet_4.zip › Data Sheet 4/Fig4E/Vimentin/3-over-186-vimentin.jpg]

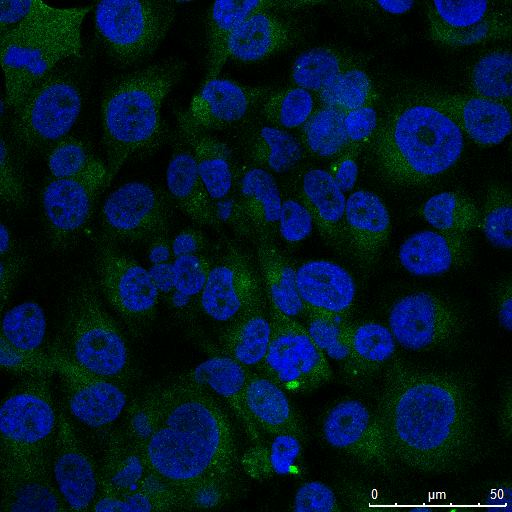

Supplement: Supplementary file 7 [file DataSheet_4.zip › Data Sheet 4/Fig4E/Vimentin/3-SiAC009948.5-vimentin-Series005_z0 -.jpg]

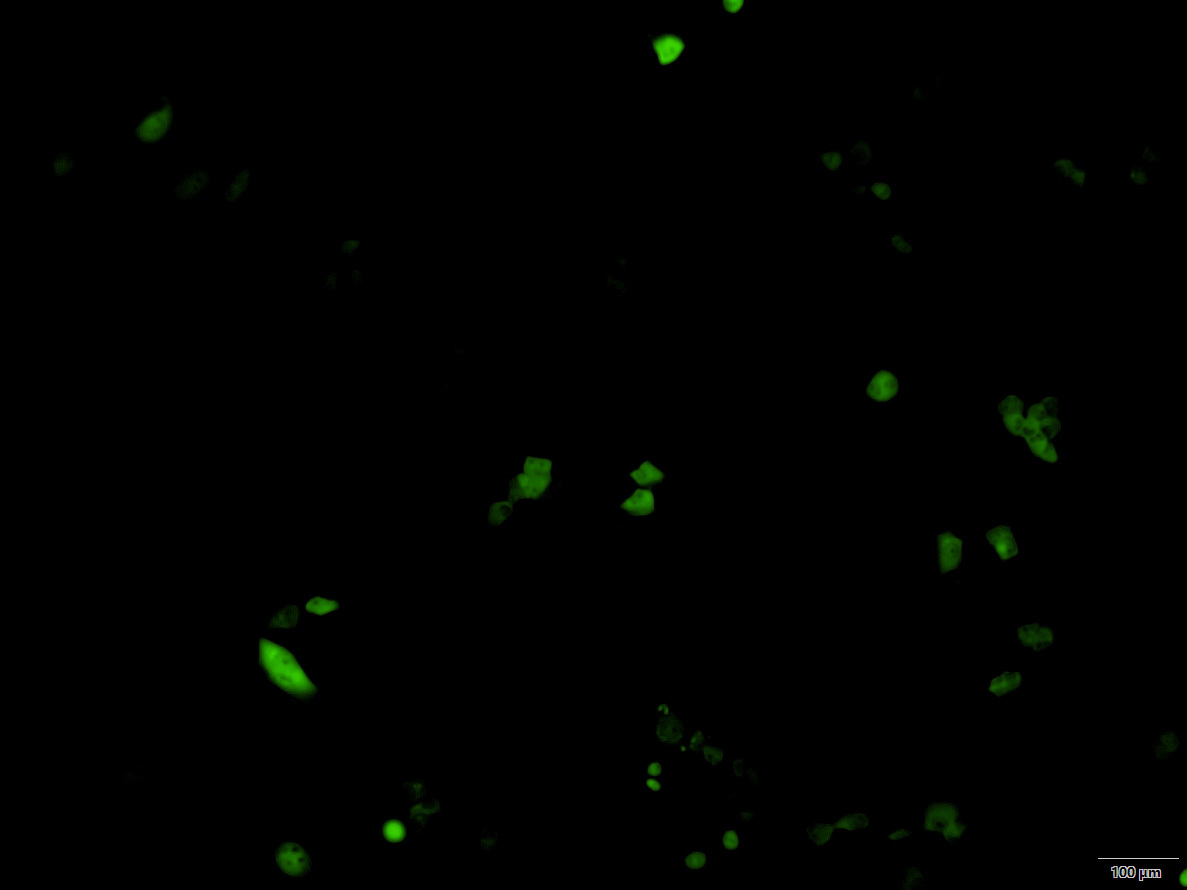

Supplement: Supplementary file 8 [file DataSheet_5.zip › Data Sheet 5/FigS1C/1-NC-day1-AC009948.5.jpg]

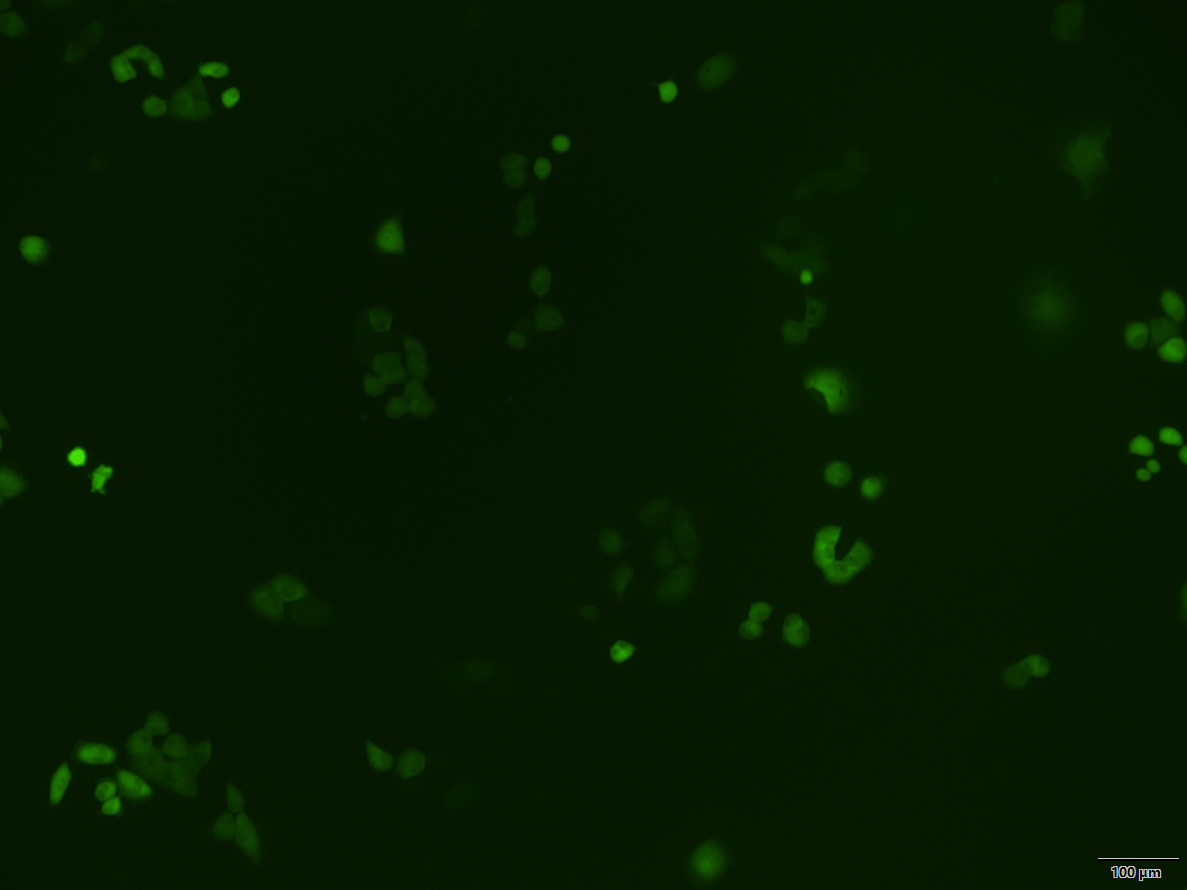

Supplement: Supplementary file 8 [file DataSheet_5.zip › Data Sheet 5/FigS1C/1-NC-day2-AC009948.5.jpg]

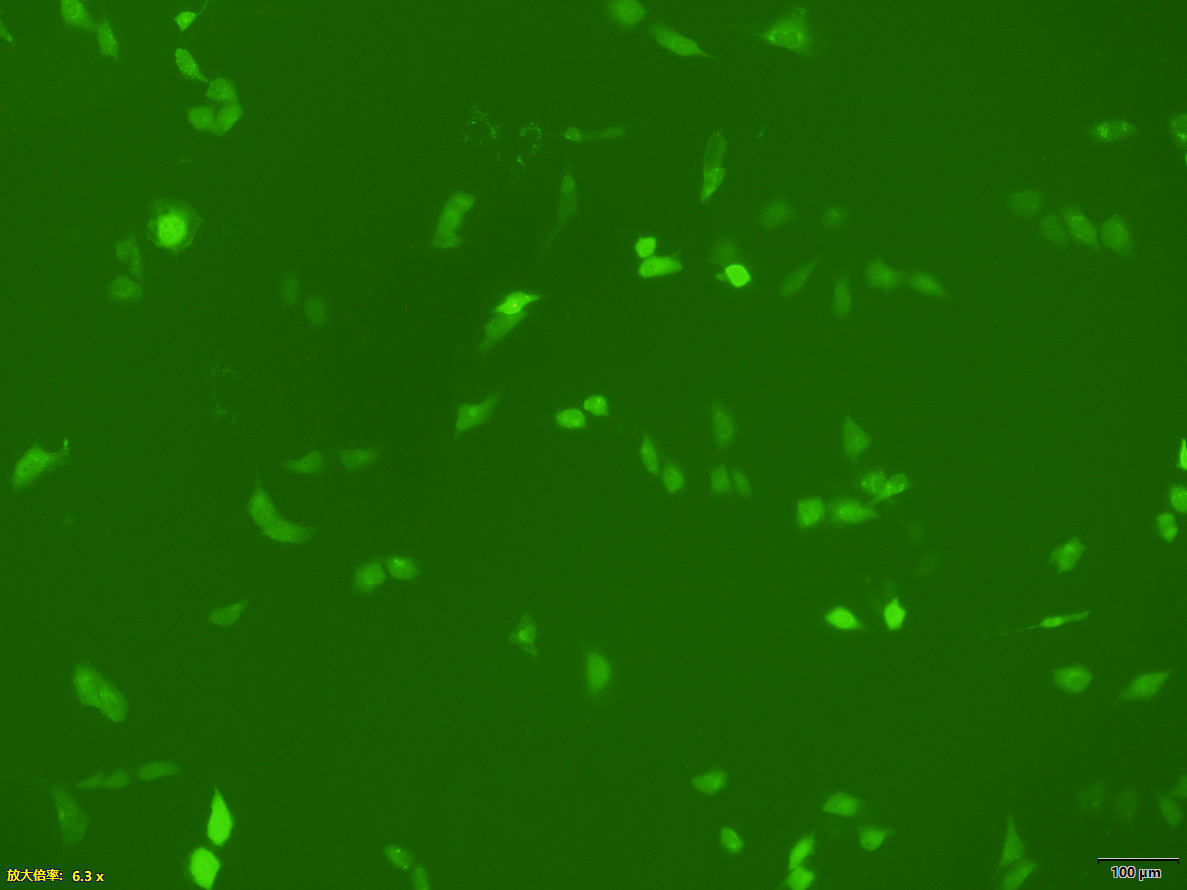

Supplement: Supplementary file 8 [file DataSheet_5.zip › Data Sheet 5/FigS1C/1-NC-day3-AC009948.5.jpg]

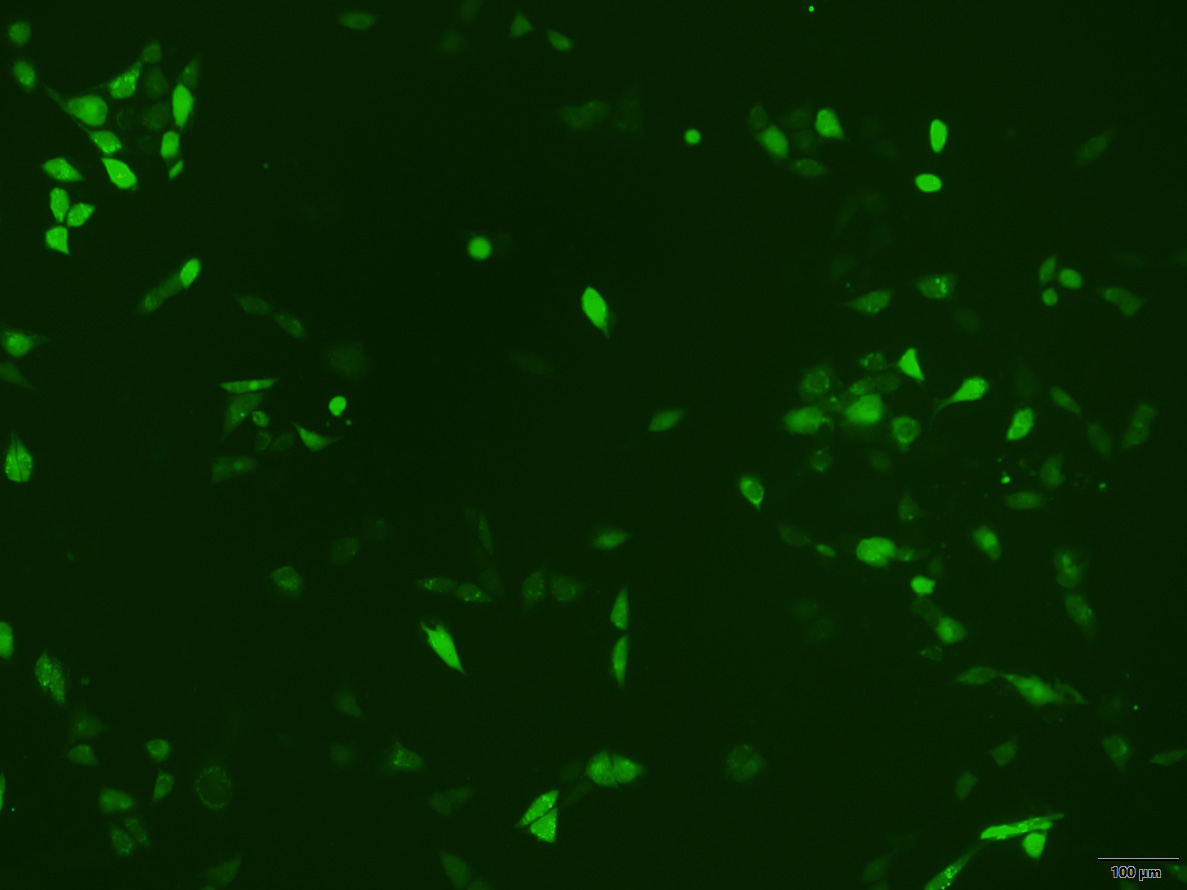

Supplement: Supplementary file 8 [file DataSheet_5.zip › Data Sheet 5/FigS1C/1-NC-day4-AC009948.5.jpg]

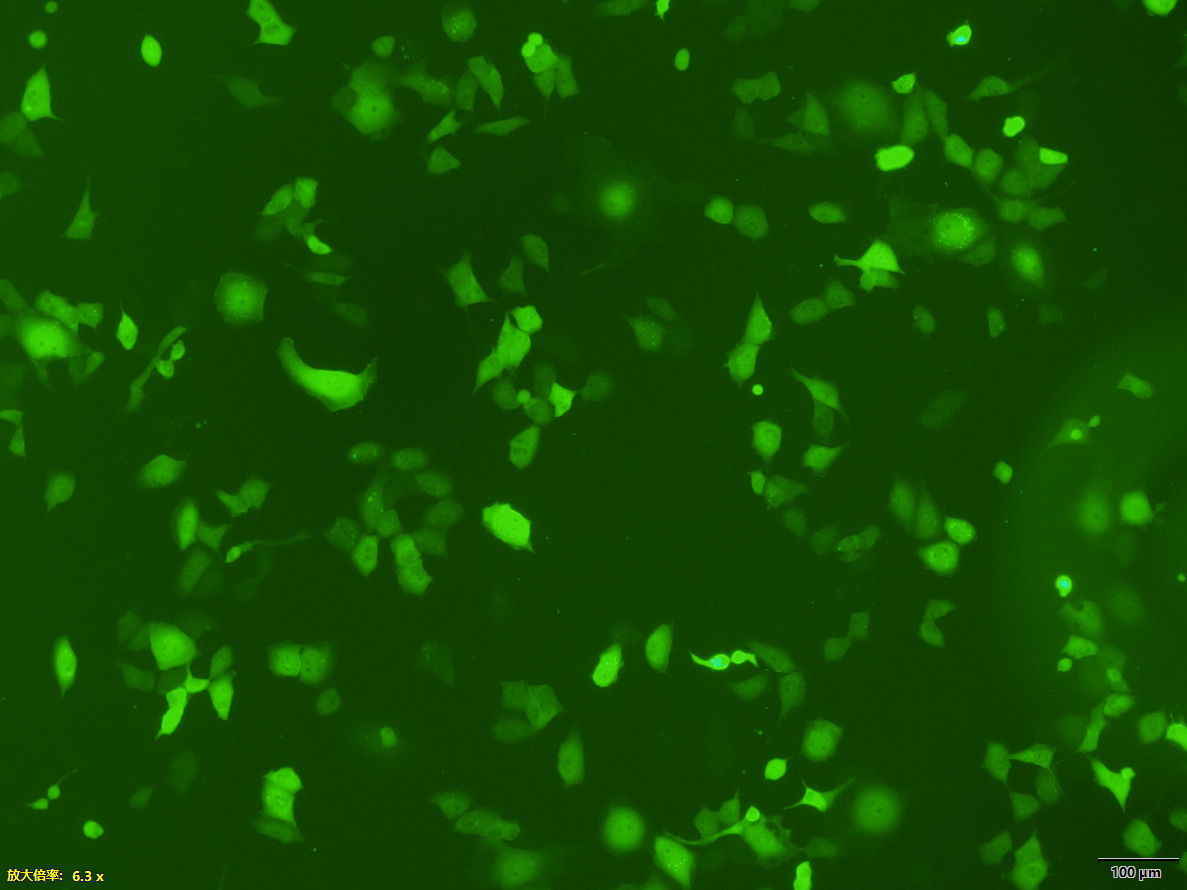

Supplement: Supplementary file 8 [file DataSheet_5.zip › Data Sheet 5/FigS1C/1-NC-day5-AC009948.5.jpg]

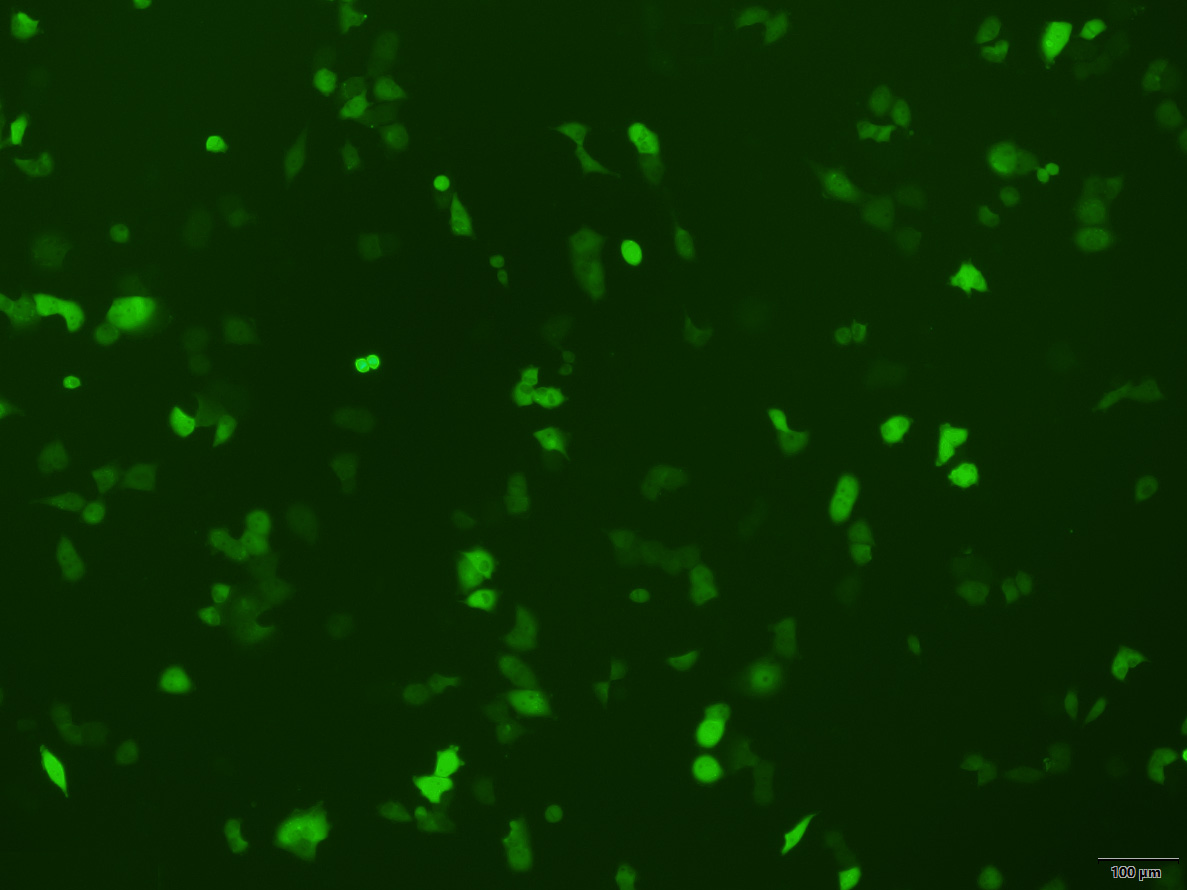

Supplement: Supplementary file 8 [file DataSheet_5.zip › Data Sheet 5/FigS1C/1-over-AC009948.5-day1.jpg]

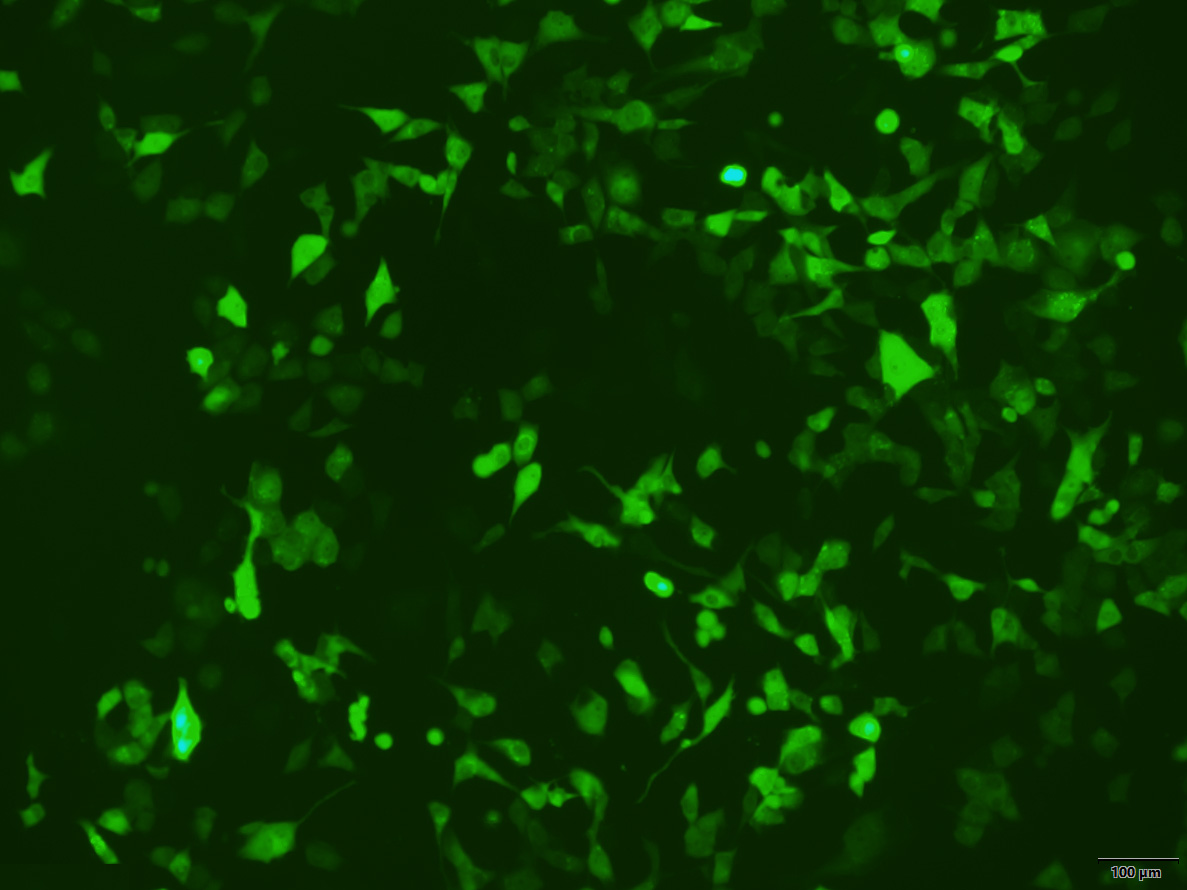

Supplement: Supplementary file 8 [file DataSheet_5.zip › Data Sheet 5/FigS1C/1-over-AC009948.5-day2.jpg]

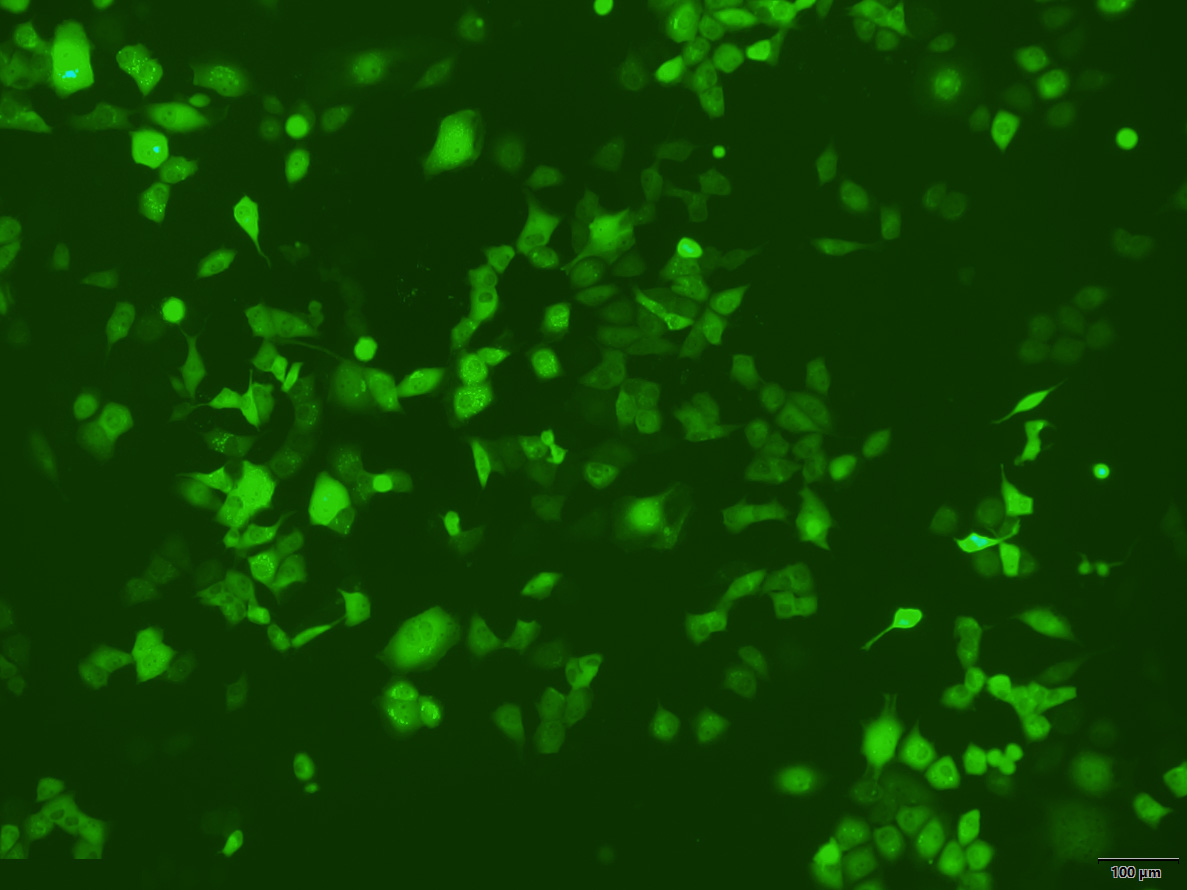

Supplement: Supplementary file 8 [file DataSheet_5.zip › Data Sheet 5/FigS1C/1-over-AC009948.5-day3.jpg]

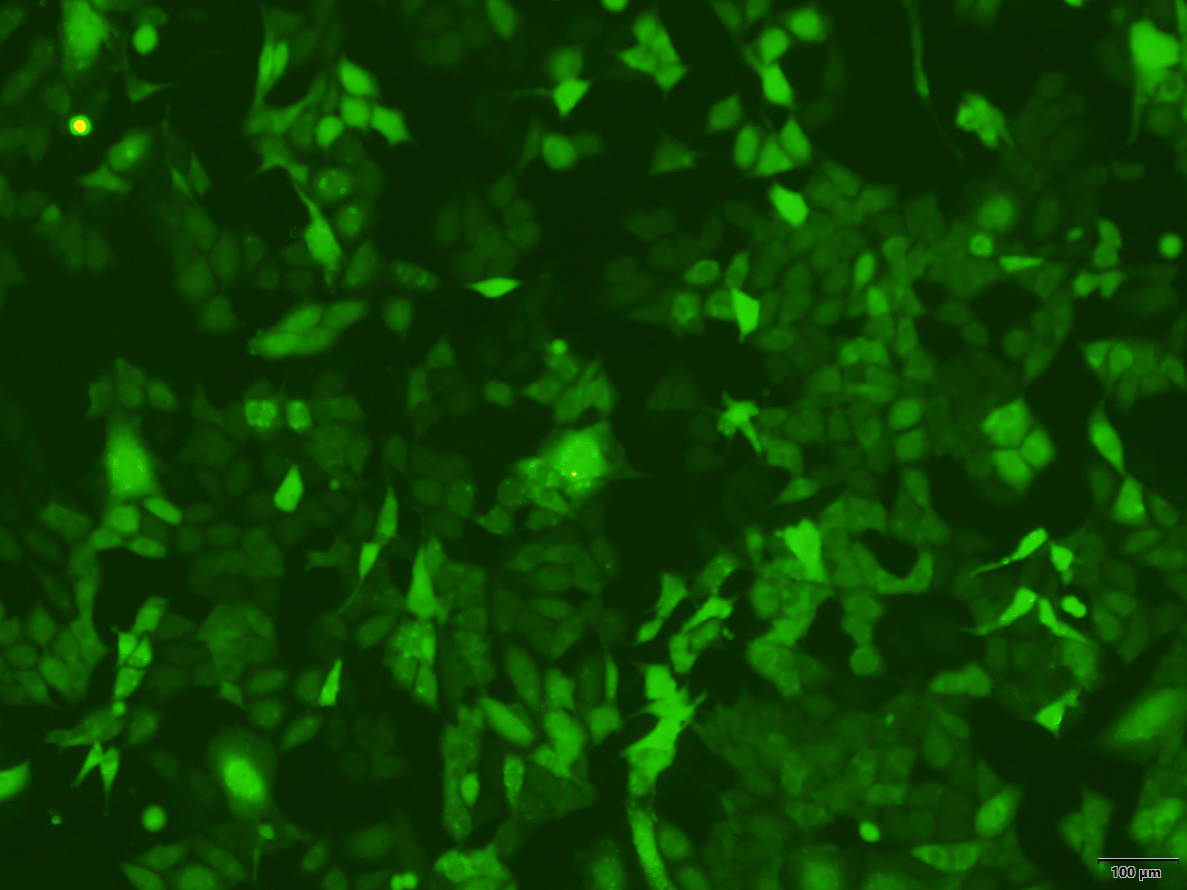

Supplement: Supplementary file 8 [file DataSheet_5.zip › Data Sheet 5/FigS1C/1-over-AC009948.5-day4.jpg]

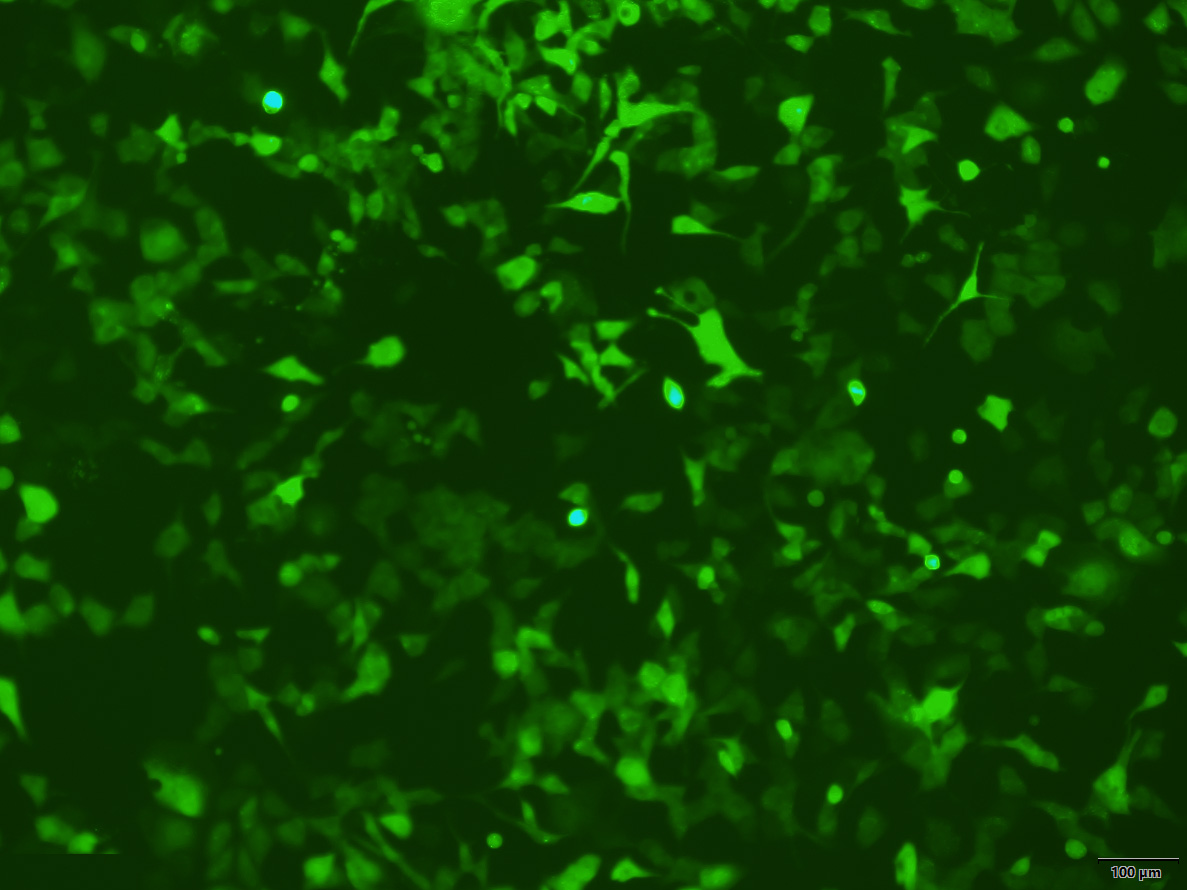

Supplement: Supplementary file 8 [file DataSheet_5.zip › Data Sheet 5/FigS1C/1-over-AC009948.5-day5.jpg]

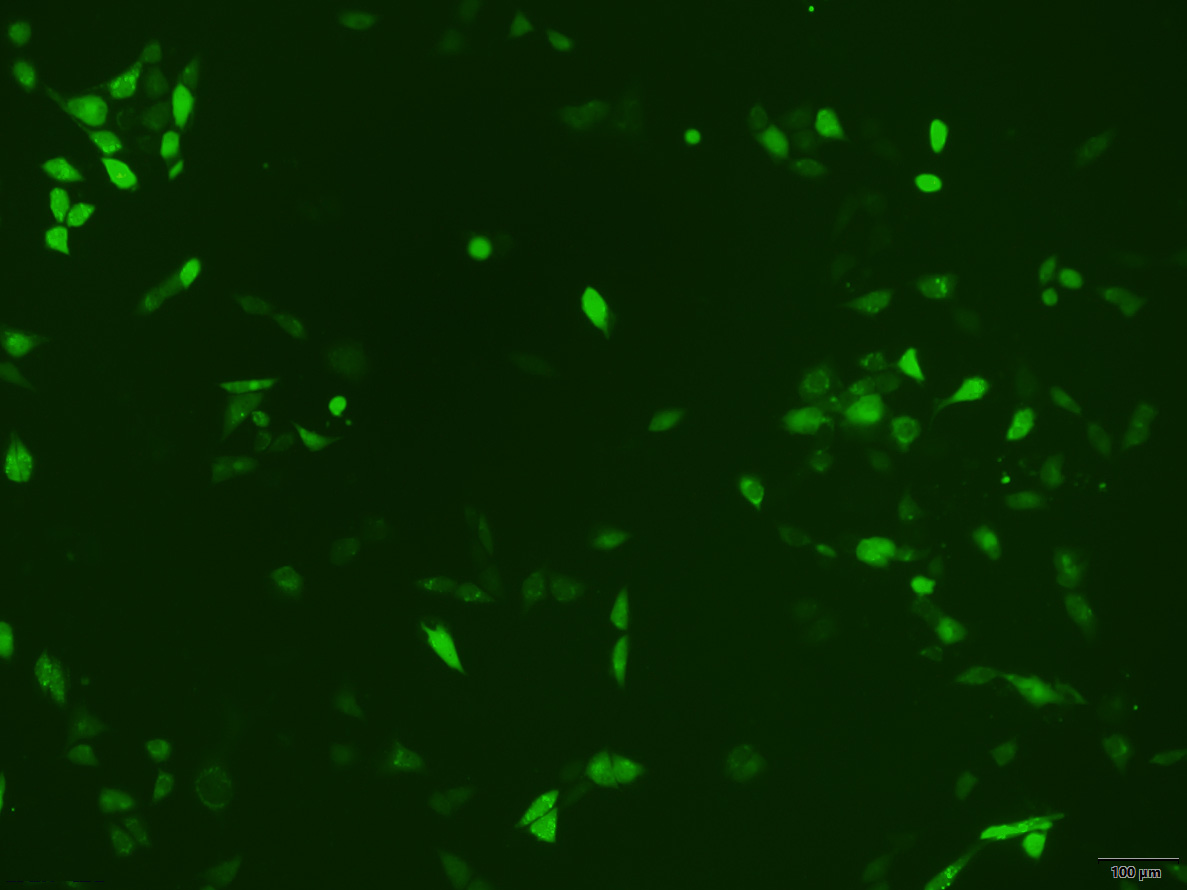

Supplement: Supplementary file 8 [file DataSheet_5.zip › Data Sheet 5/FigS1C/1-Scrambled-day1.jpg]

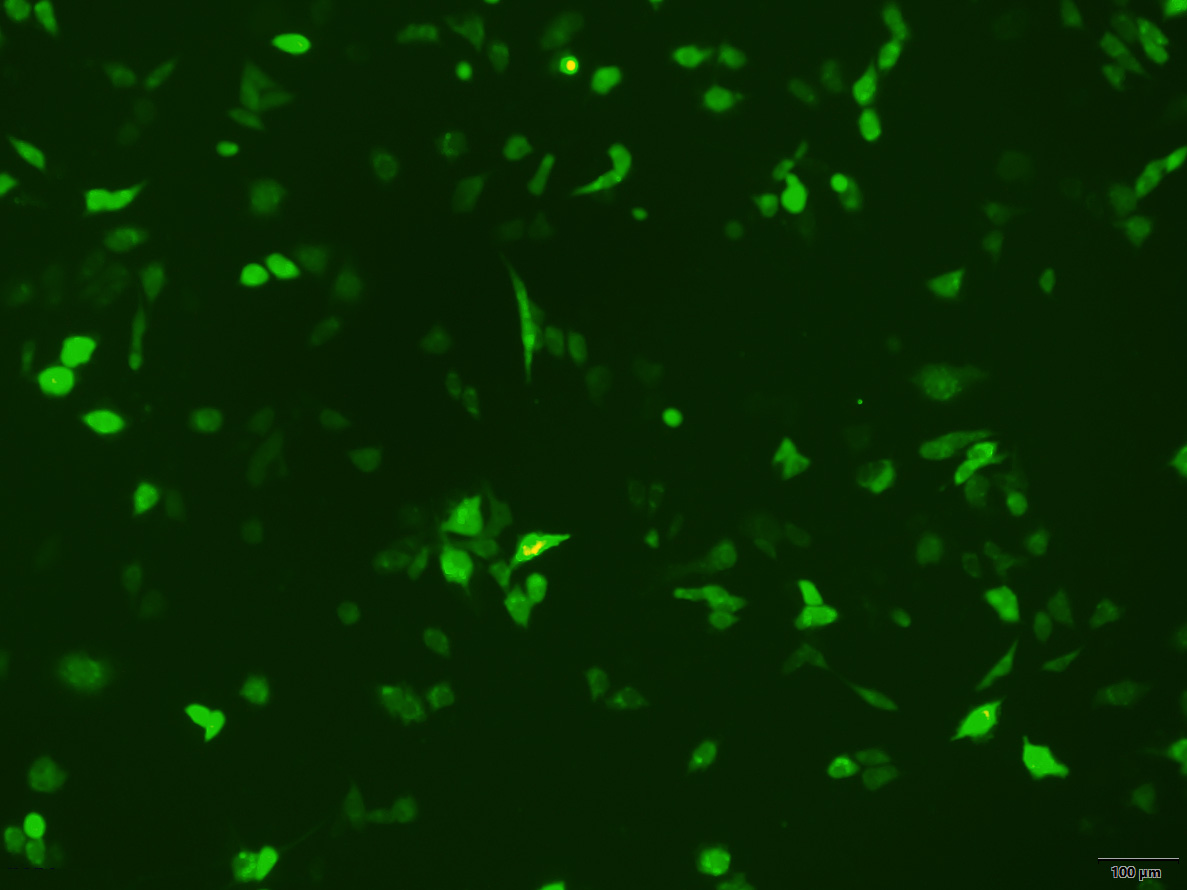

Supplement: Supplementary file 8 [file DataSheet_5.zip › Data Sheet 5/FigS1C/1-Scrambled-day2.jpg]

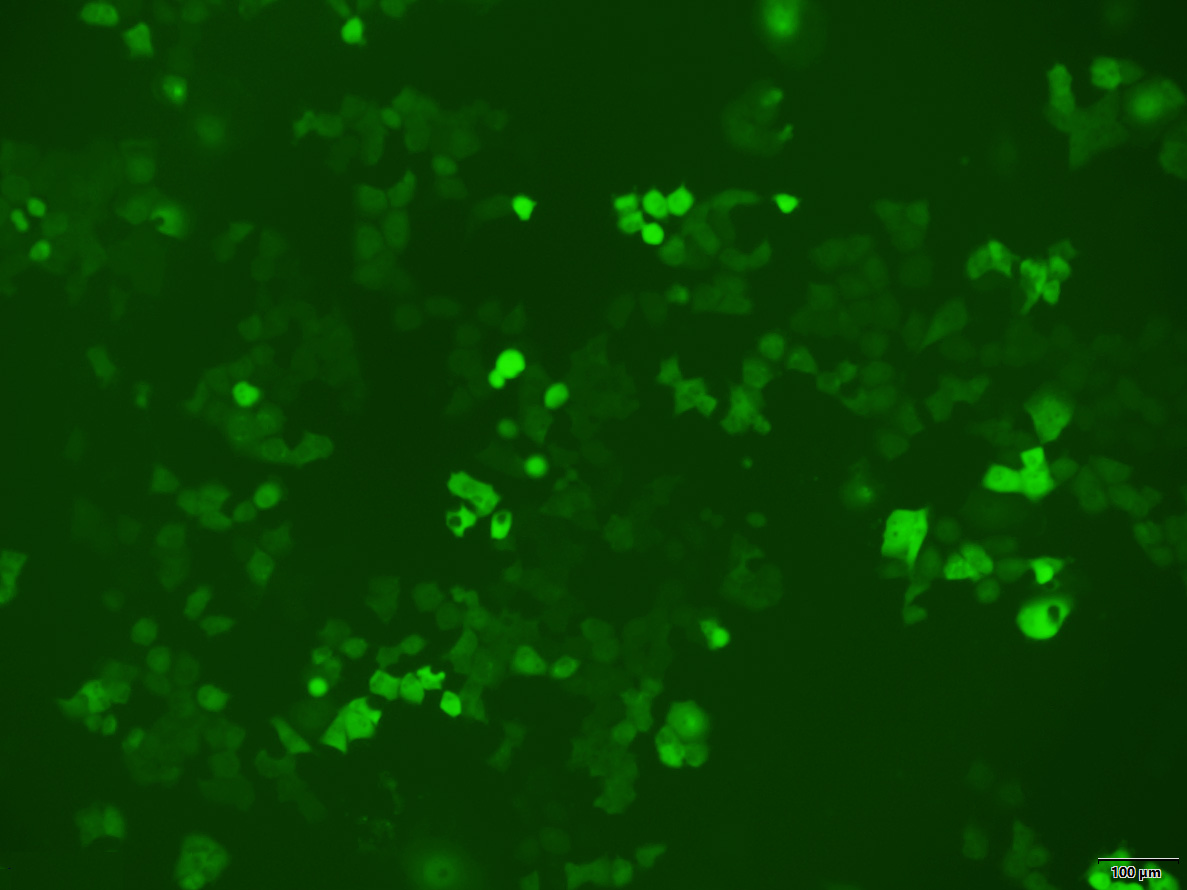

Supplement: Supplementary file 8 [file DataSheet_5.zip › Data Sheet 5/FigS1C/1-Scrambled-day3.jpg]

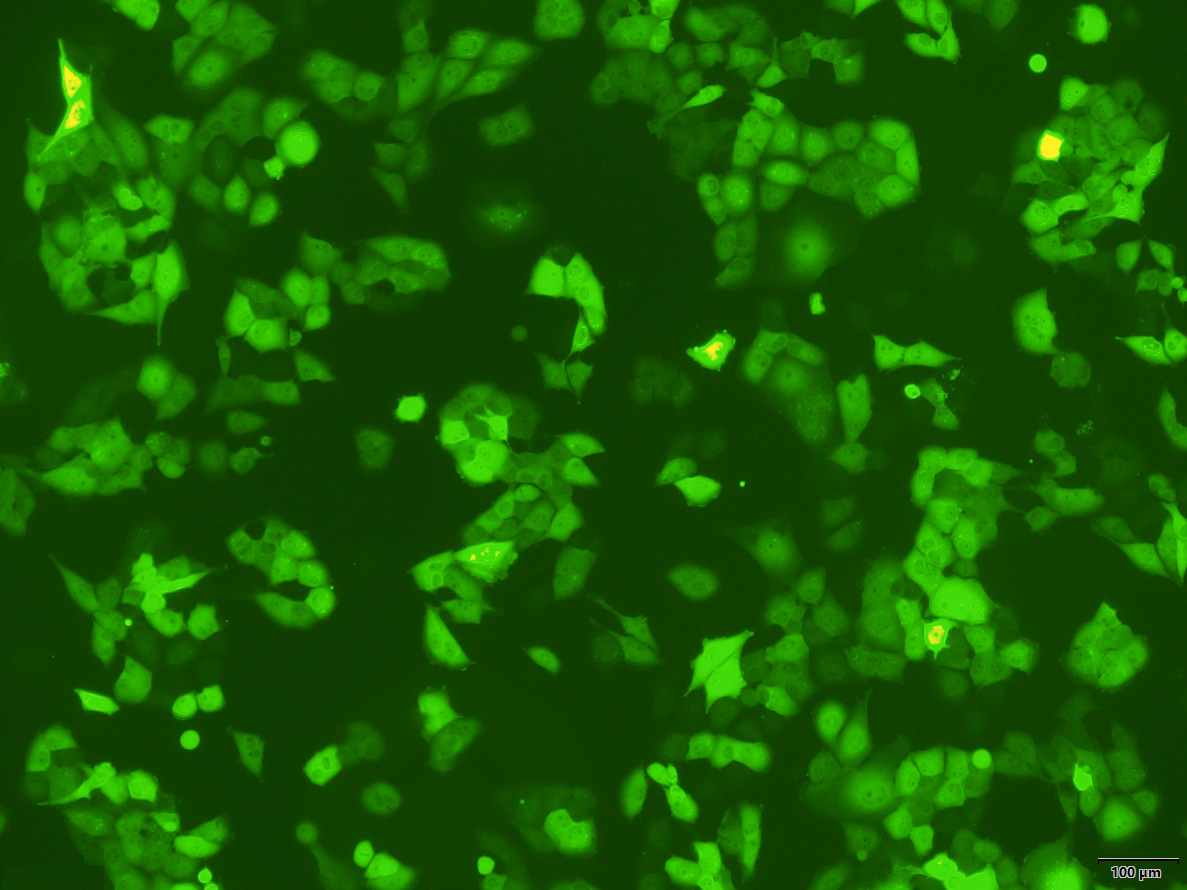

Supplement: Supplementary file 8 [file DataSheet_5.zip › Data Sheet 5/FigS1C/1-Scrambled-day4.jpg]

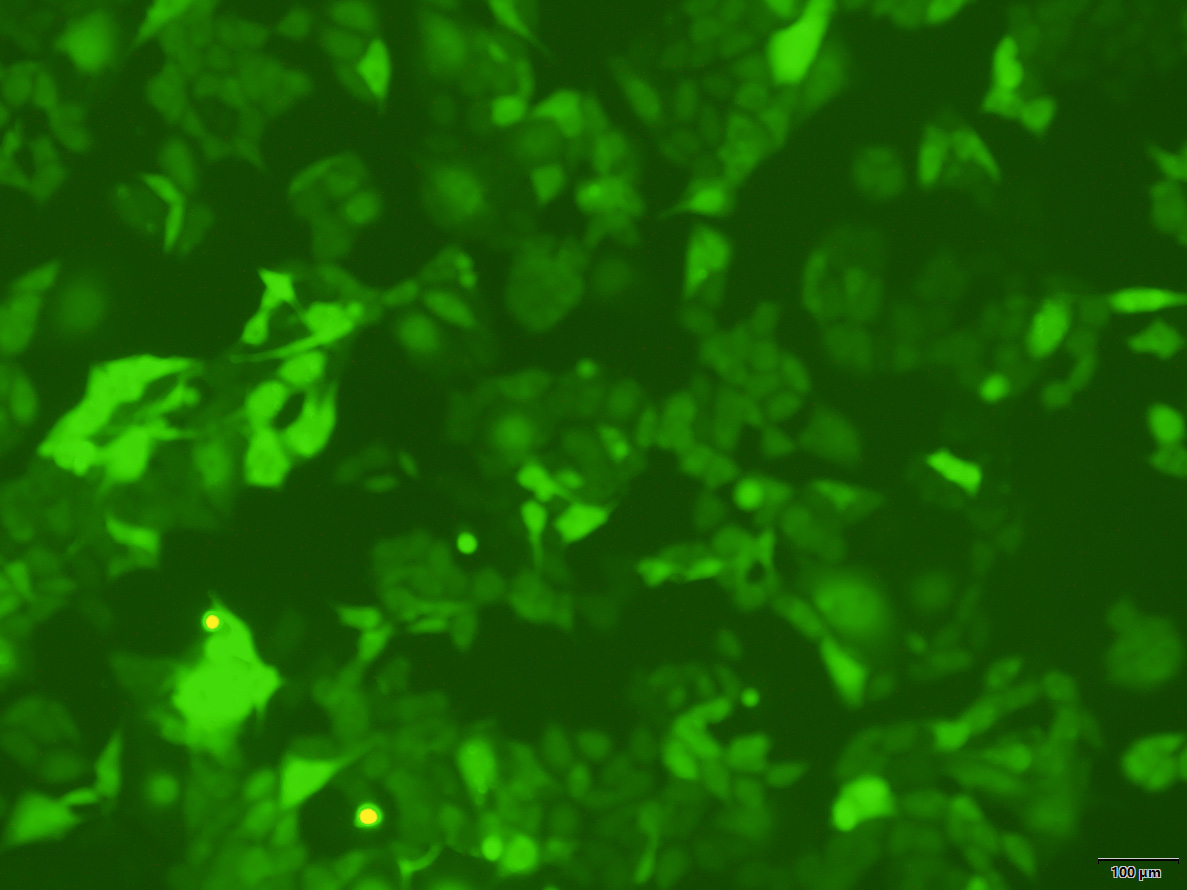

Supplement: Supplementary file 8 [file DataSheet_5.zip › Data Sheet 5/FigS1C/1-Scrambled-day5.jpg]

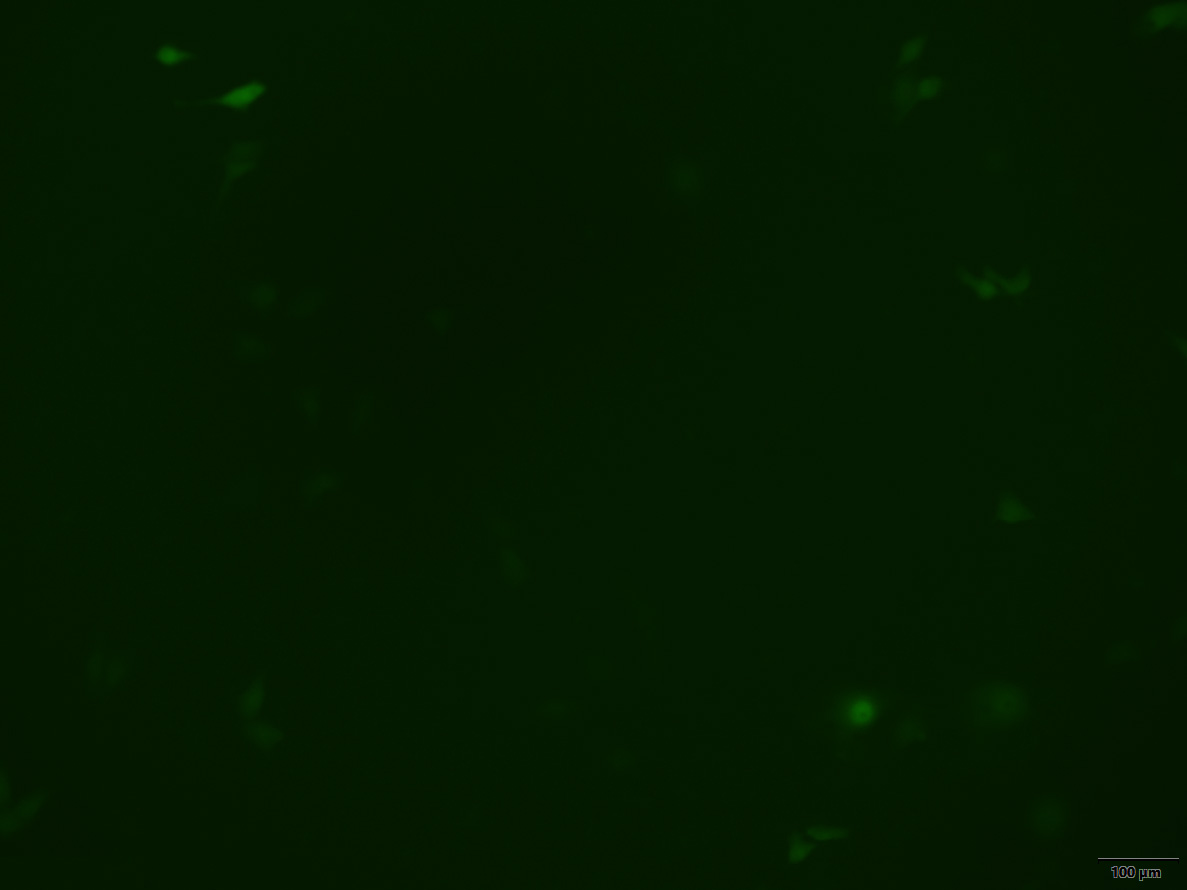

Supplement: Supplementary file 8 [file DataSheet_5.zip › Data Sheet 5/FigS1C/1-SiAC009948.5-day1.jpg]

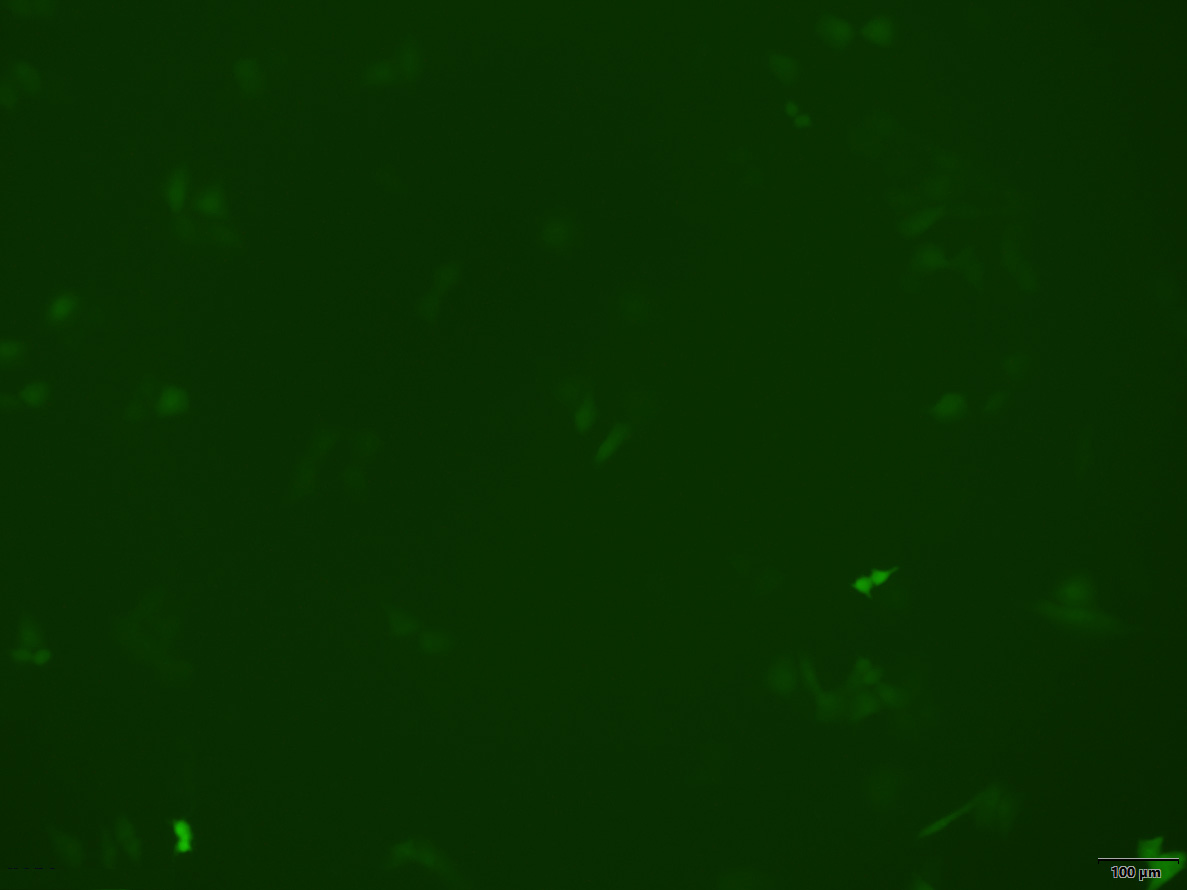

Supplement: Supplementary file 8 [file DataSheet_5.zip › Data Sheet 5/FigS1C/1-SiAC009948.5-day2.jpg]

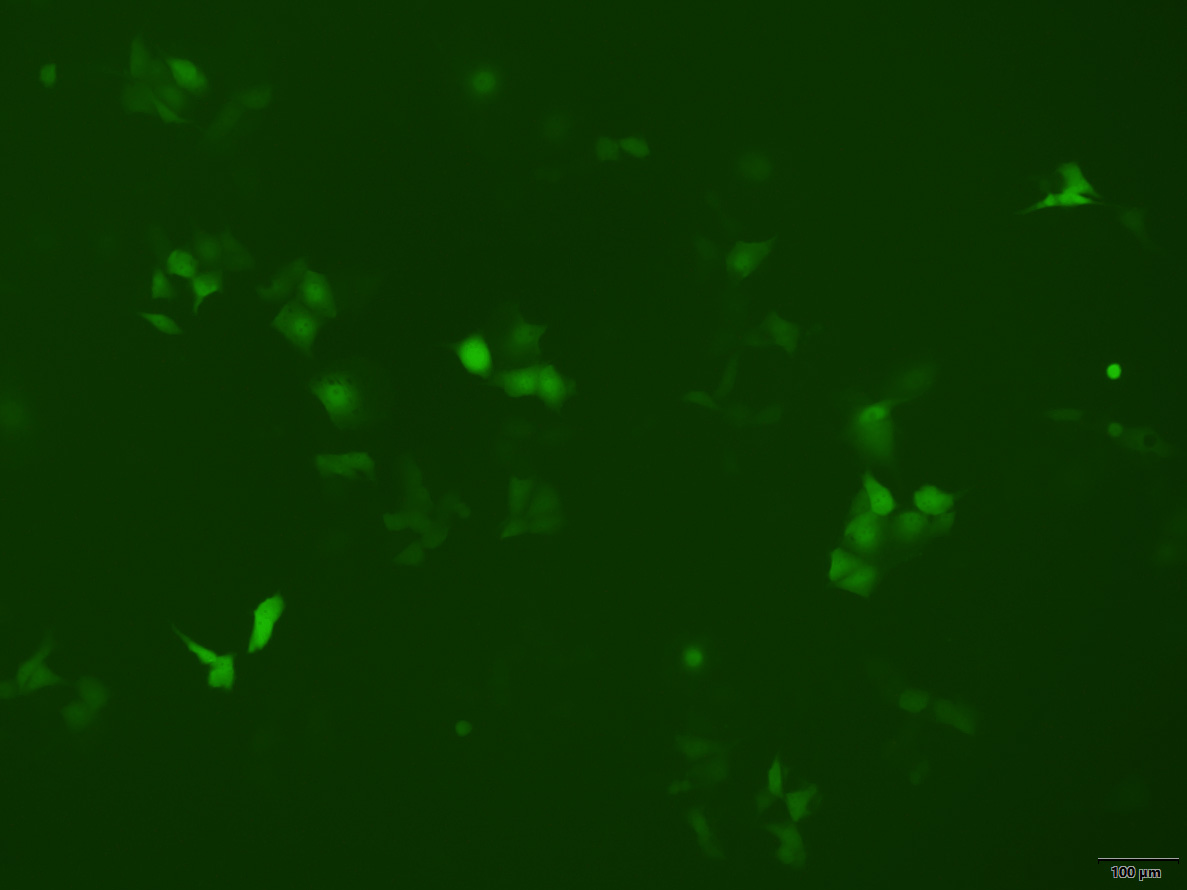

Supplement: Supplementary file 8 [file DataSheet_5.zip › Data Sheet 5/FigS1C/1-SiAC009948.5-day3.jpg]

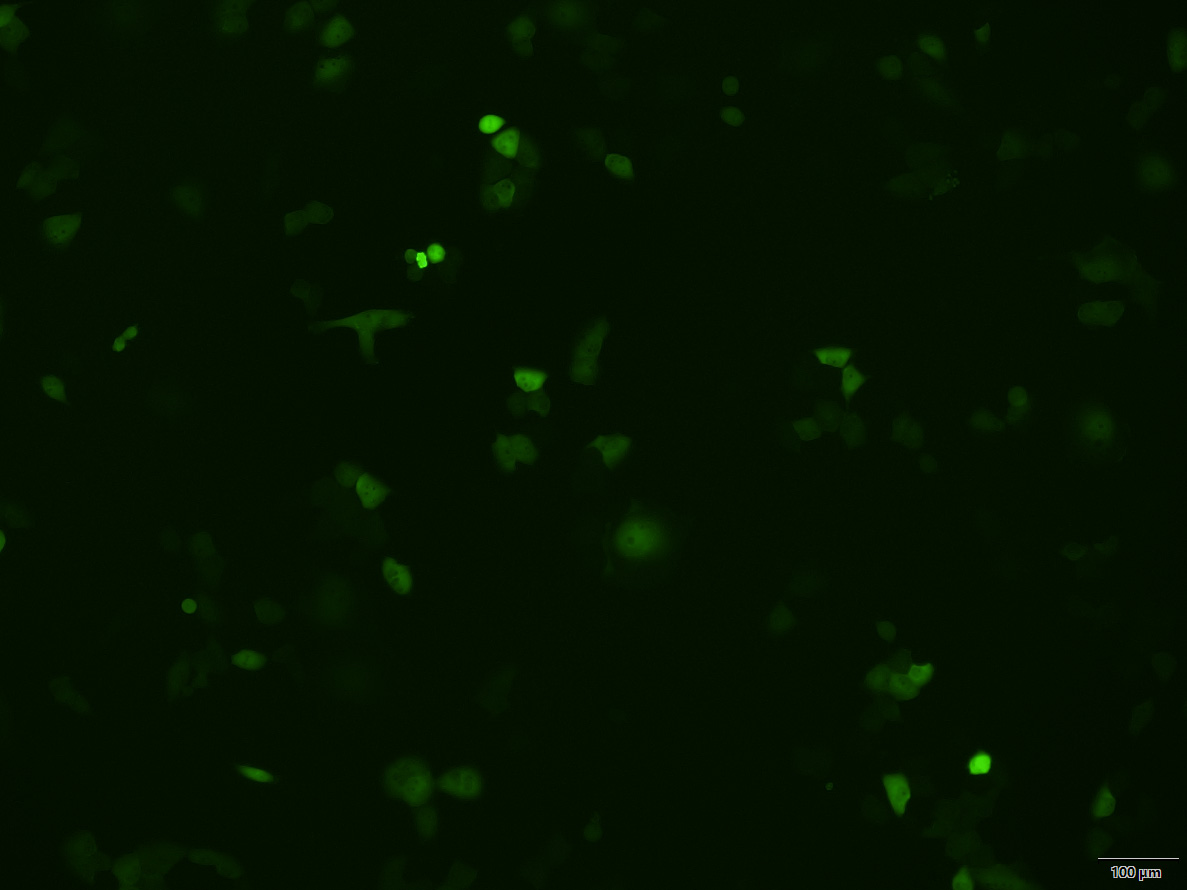

Supplement: Supplementary file 8 [file DataSheet_5.zip › Data Sheet 5/FigS1C/1-SiAC009948.5-day4.jpg]

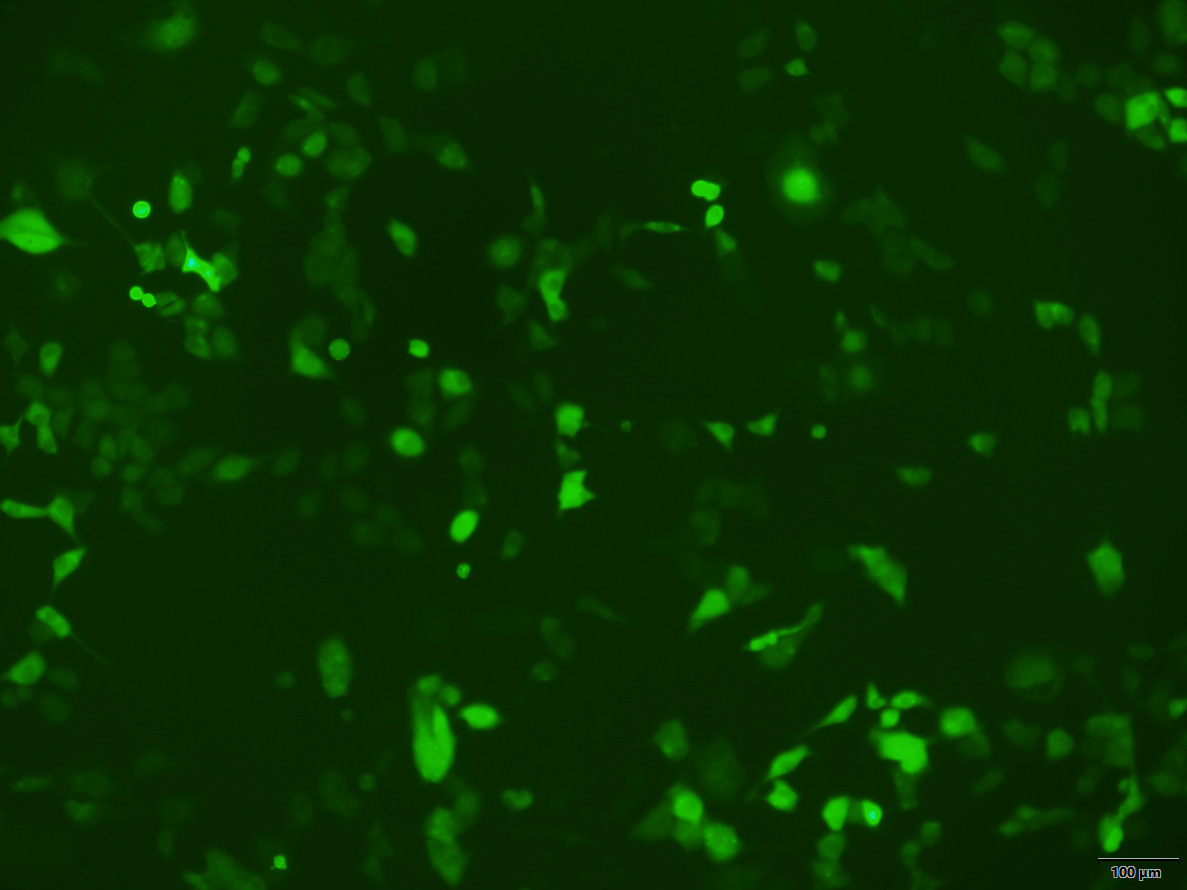

Supplement: Supplementary file 8 [file DataSheet_5.zip › Data Sheet 5/FigS1C/1-SiAC009948.5-day5.jpg]

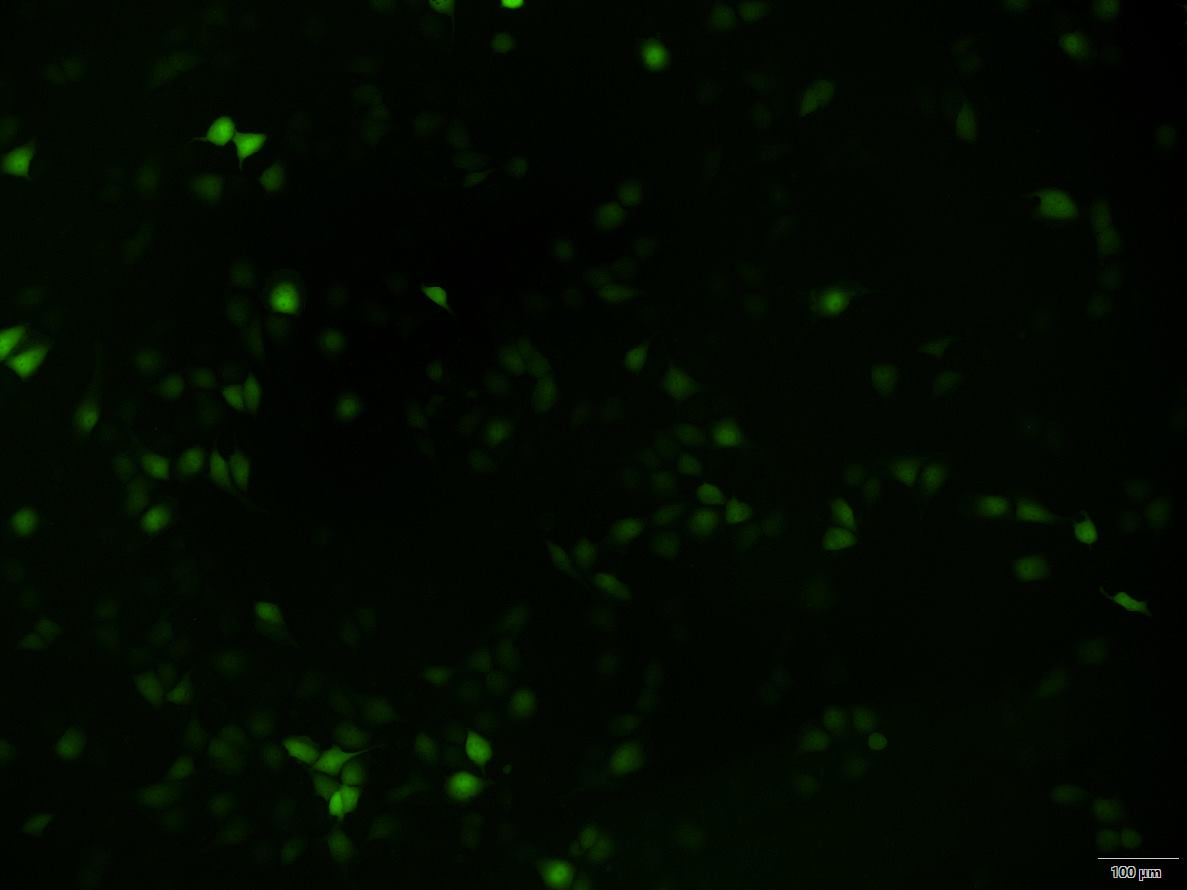

Supplement: Supplementary file 8 [file DataSheet_5.zip › Data Sheet 5/FigS1C/2-NC-day1-AC009948.5.jpg]

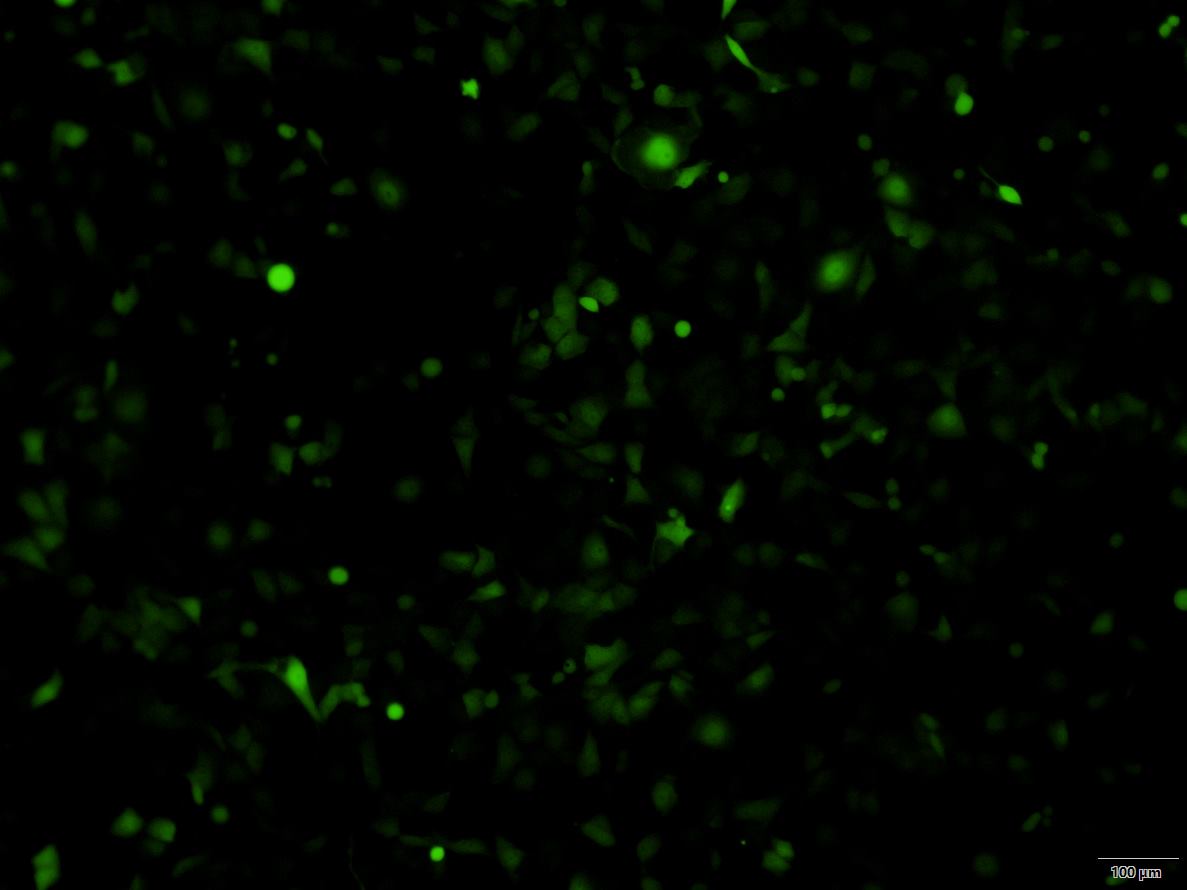

Supplement: Supplementary file 8 [file DataSheet_5.zip › Data Sheet 5/FigS1C/2-NC-day2-AC009948.5.jpg]

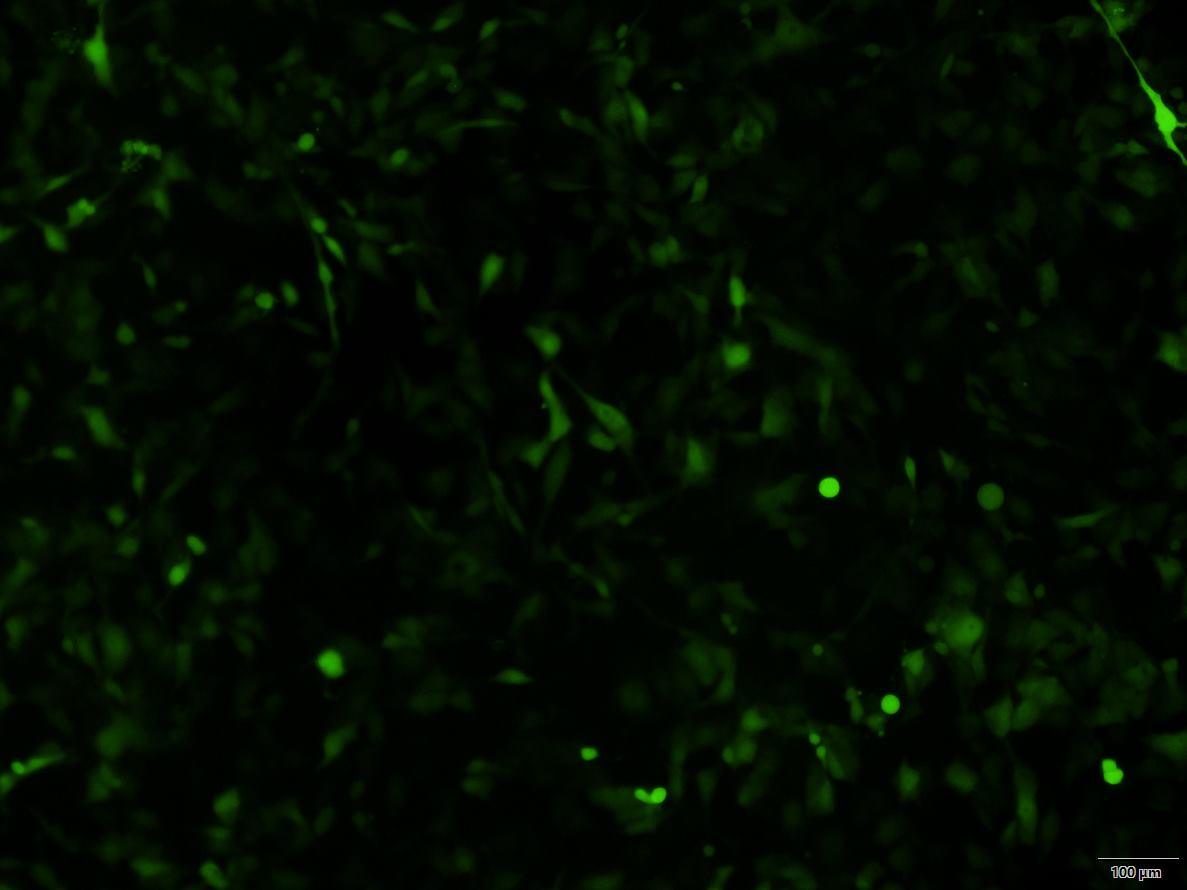

Supplement: Supplementary file 8 [file DataSheet_5.zip › Data Sheet 5/FigS1C/2-NC-day3-AC009948.5.jpg]

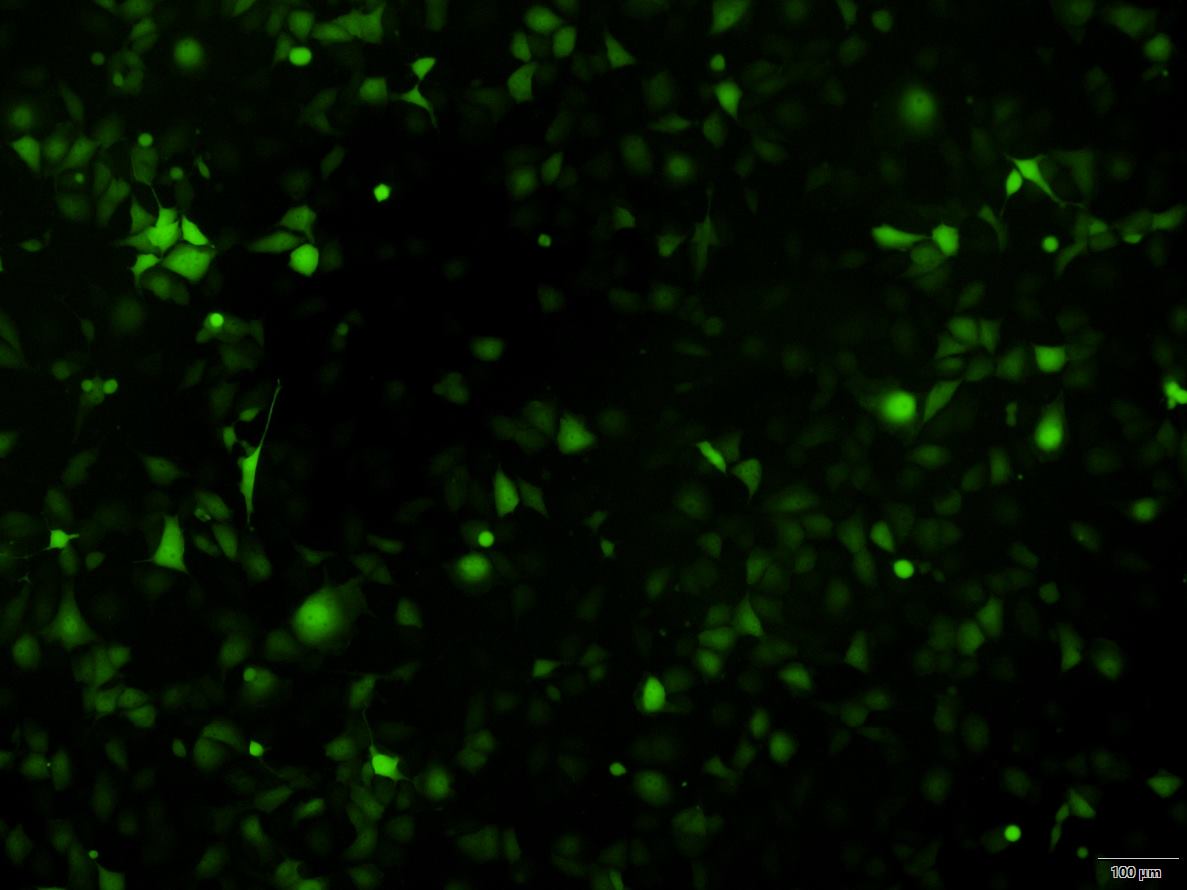

Supplement: Supplementary file 8 [file DataSheet_5.zip › Data Sheet 5/FigS1C/2-NC-day4-AC009948.5.jpg]

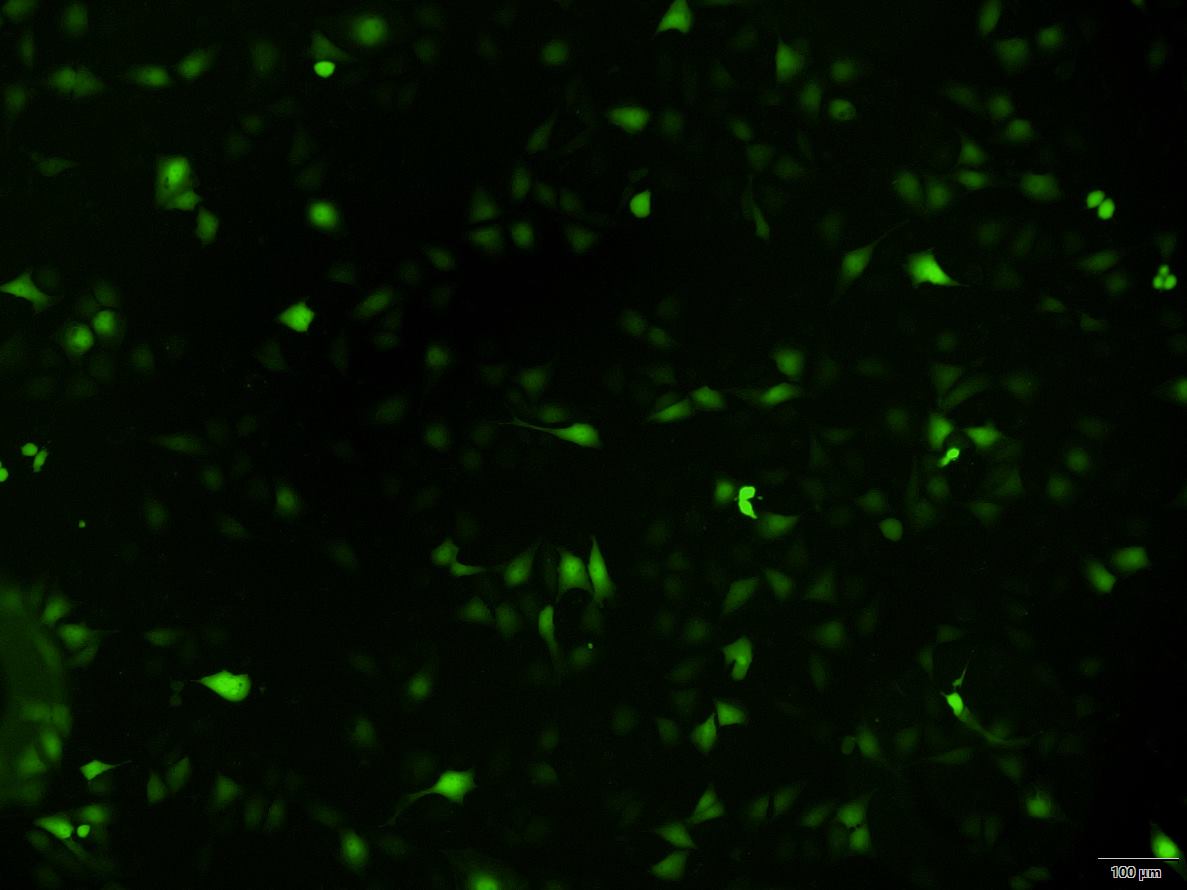

Supplement: Supplementary file 8 [file DataSheet_5.zip › Data Sheet 5/FigS1C/2-NC-day5-AC009948.5.jpg]

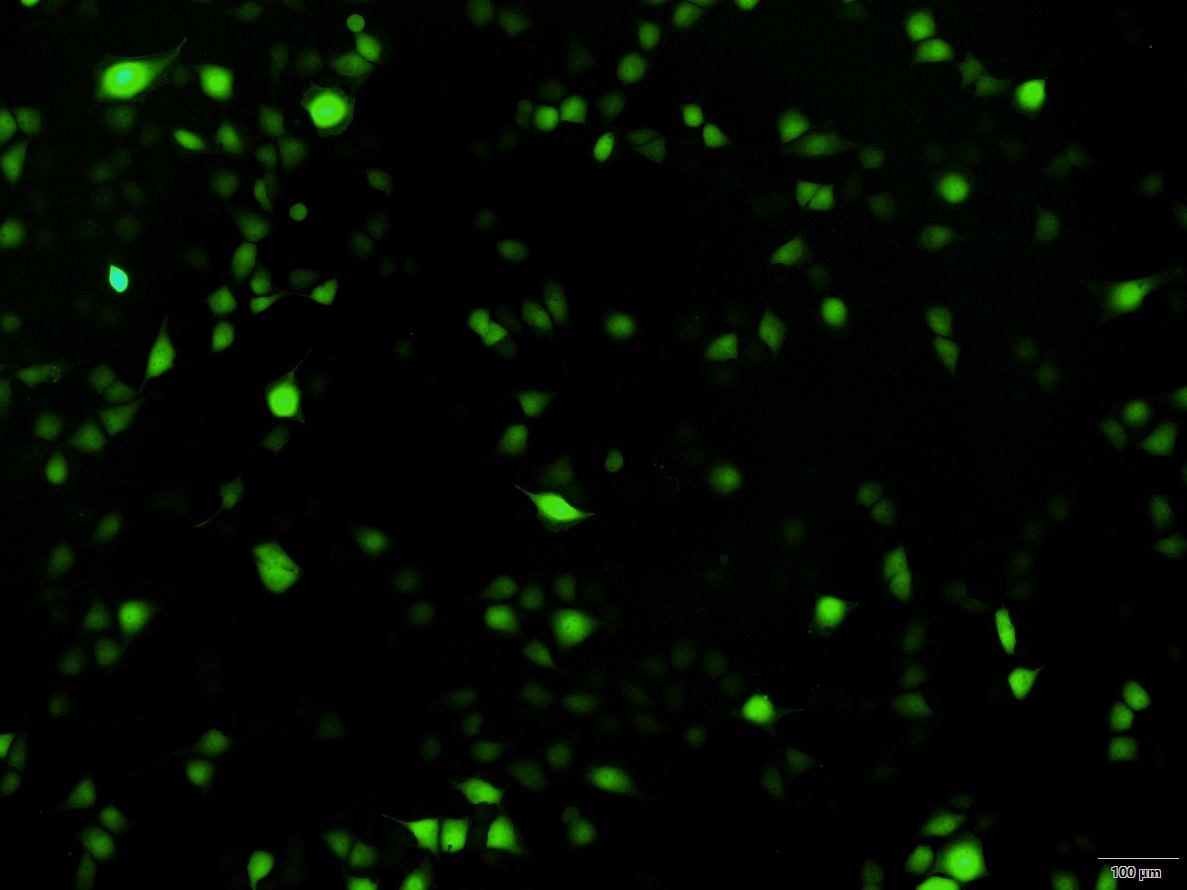

Supplement: Supplementary file 8 [file DataSheet_5.zip › Data Sheet 5/FigS1C/2-over-AC009948.5-day1.jpg]

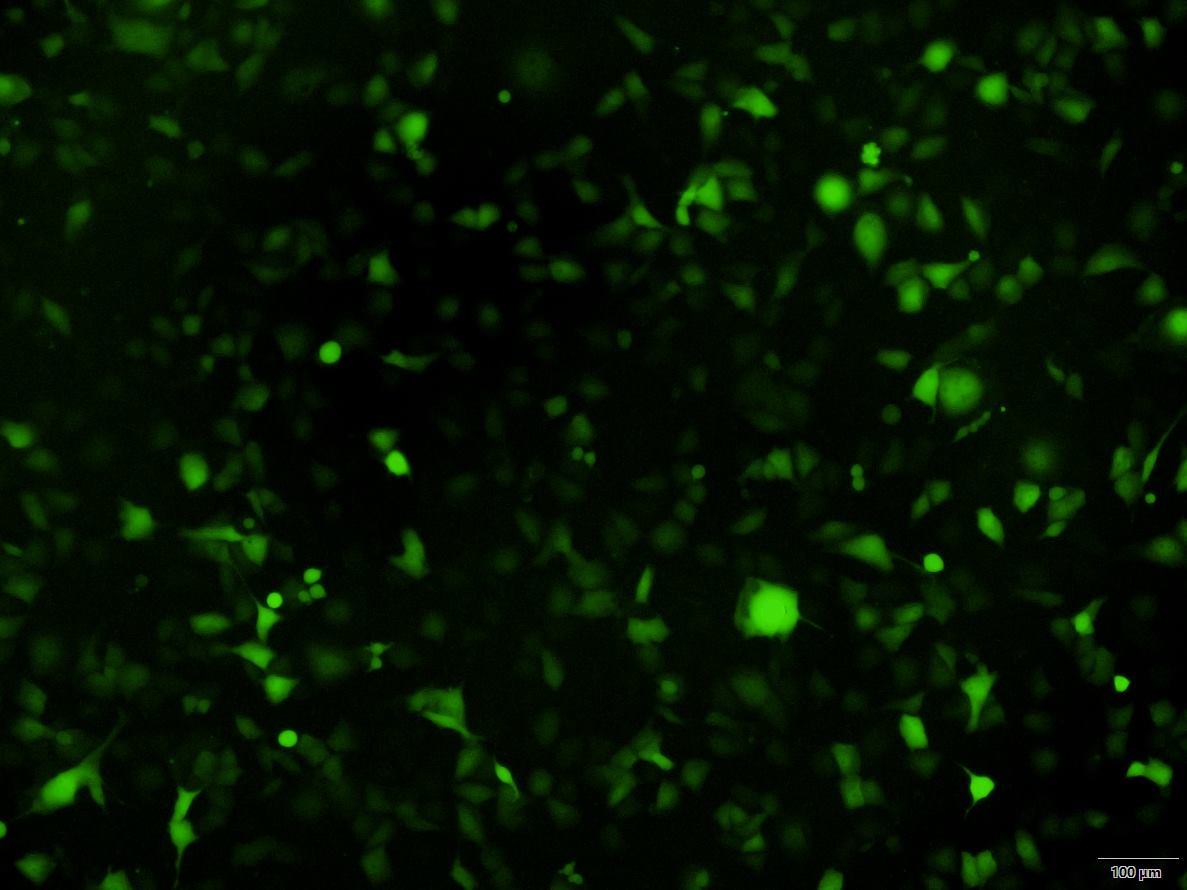

Supplement: Supplementary file 8 [file DataSheet_5.zip › Data Sheet 5/FigS1C/2-over-AC009948.5-day2.jpg]

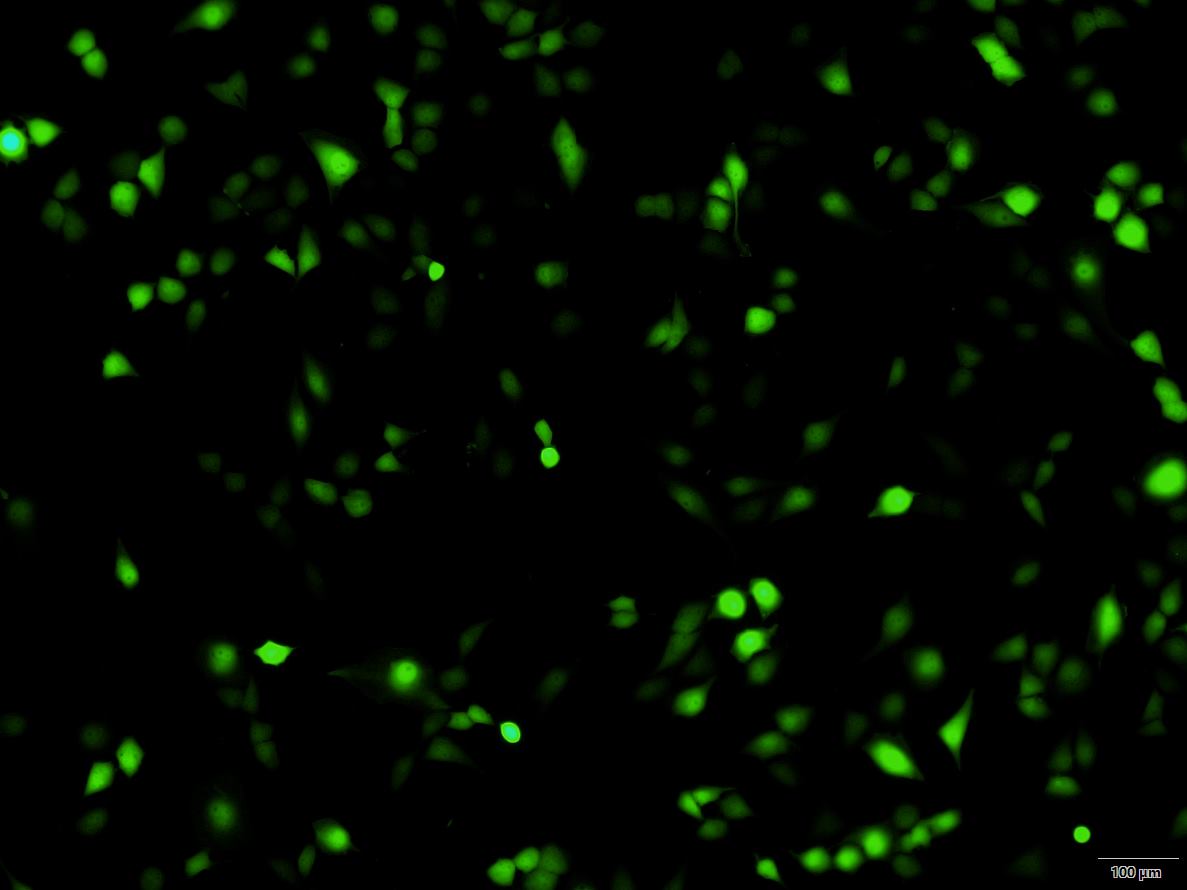

Supplement: Supplementary file 8 [file DataSheet_5.zip › Data Sheet 5/FigS1C/2-over-AC009948.5-day3.jpg]

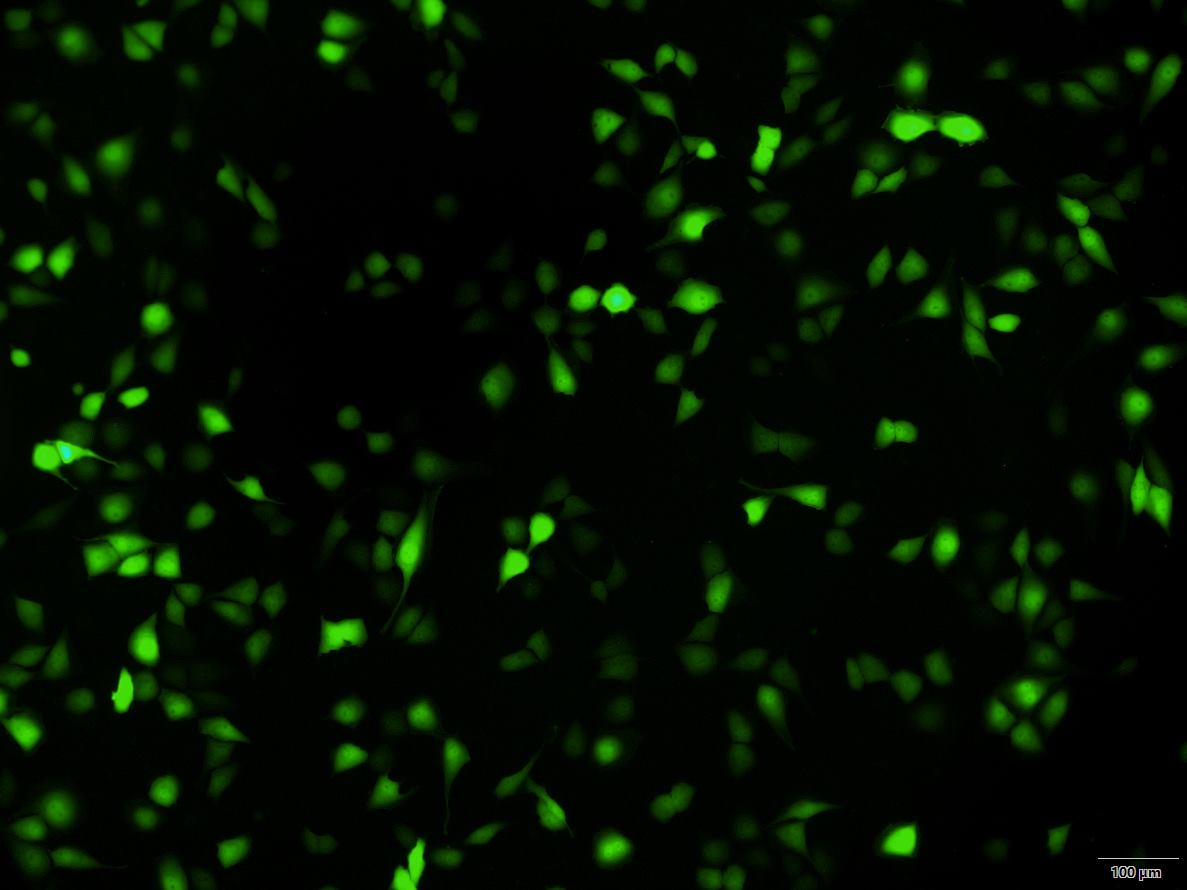

Supplement: Supplementary file 8 [file DataSheet_5.zip › Data Sheet 5/FigS1C/2-over-AC009948.5-day4.jpg]

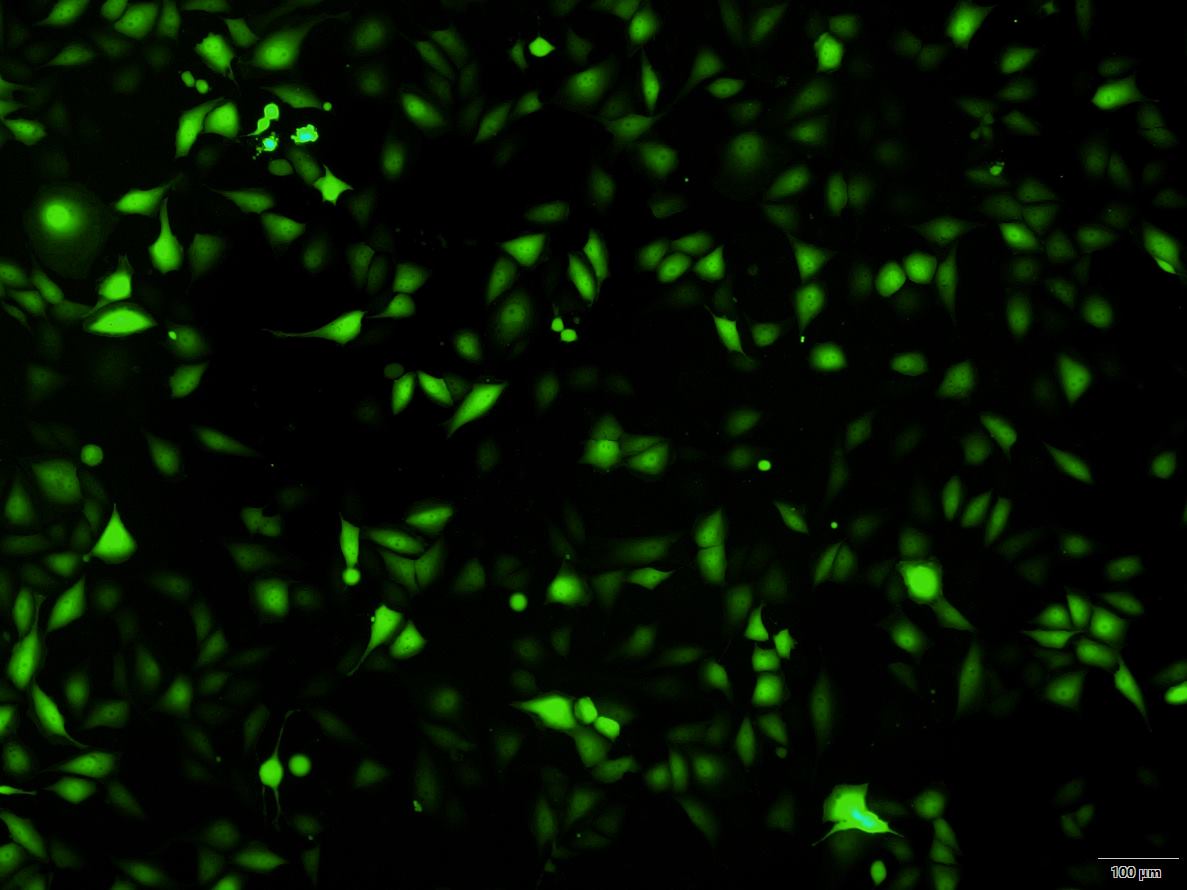

Supplement: Supplementary file 8 [file DataSheet_5.zip › Data Sheet 5/FigS1C/2-over-AC009948.5-day5.jpg]

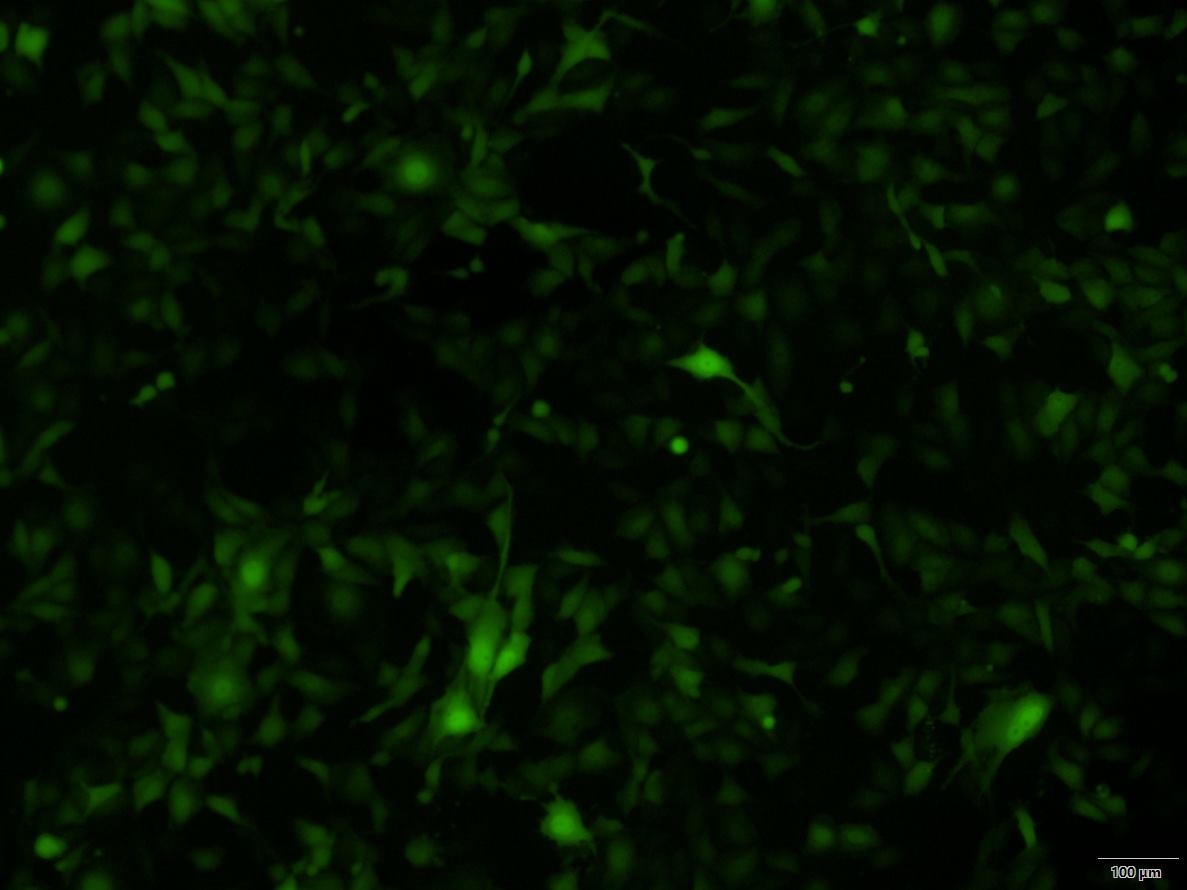

Supplement: Supplementary file 8 [file DataSheet_5.zip › Data Sheet 5/FigS1C/2-Scrambled-day1.jpg]

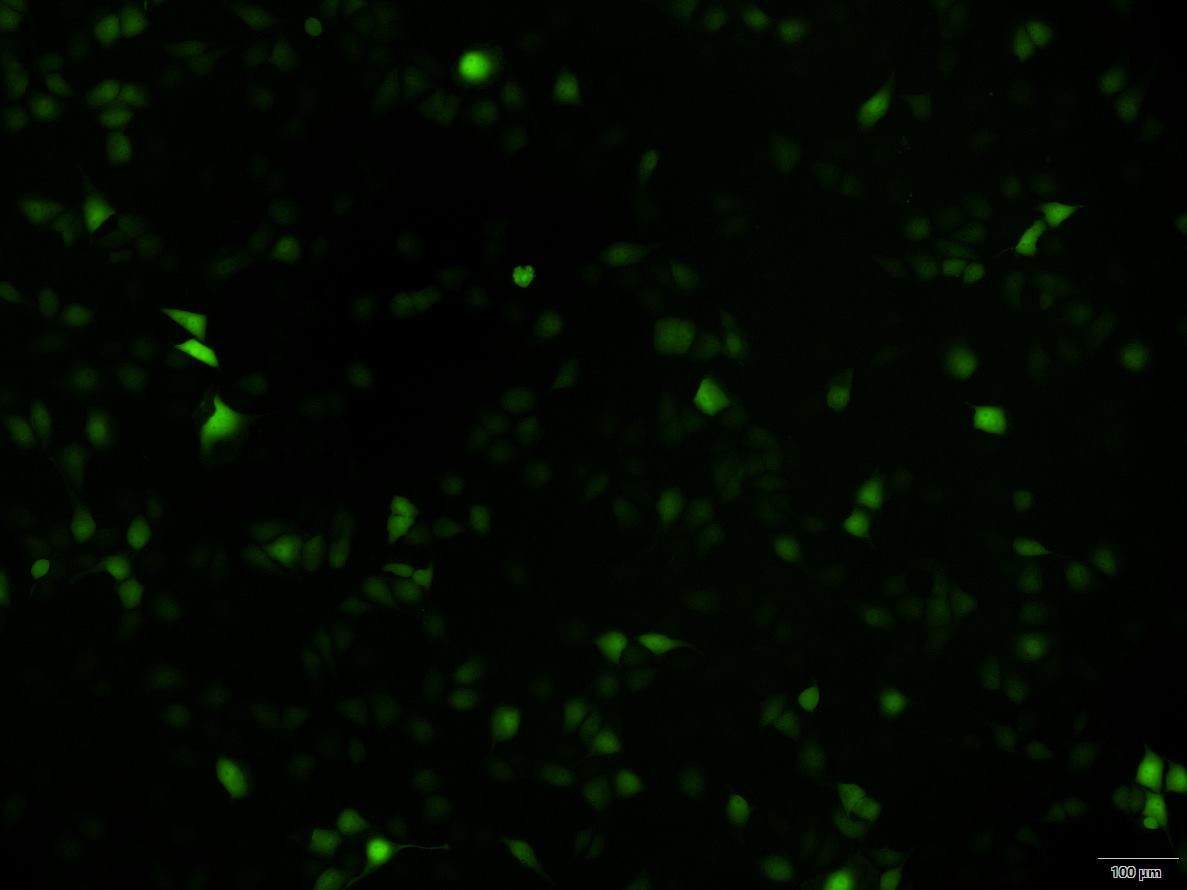

Supplement: Supplementary file 8 [file DataSheet_5.zip › Data Sheet 5/FigS1C/2-Scrambled-day2.jpg]

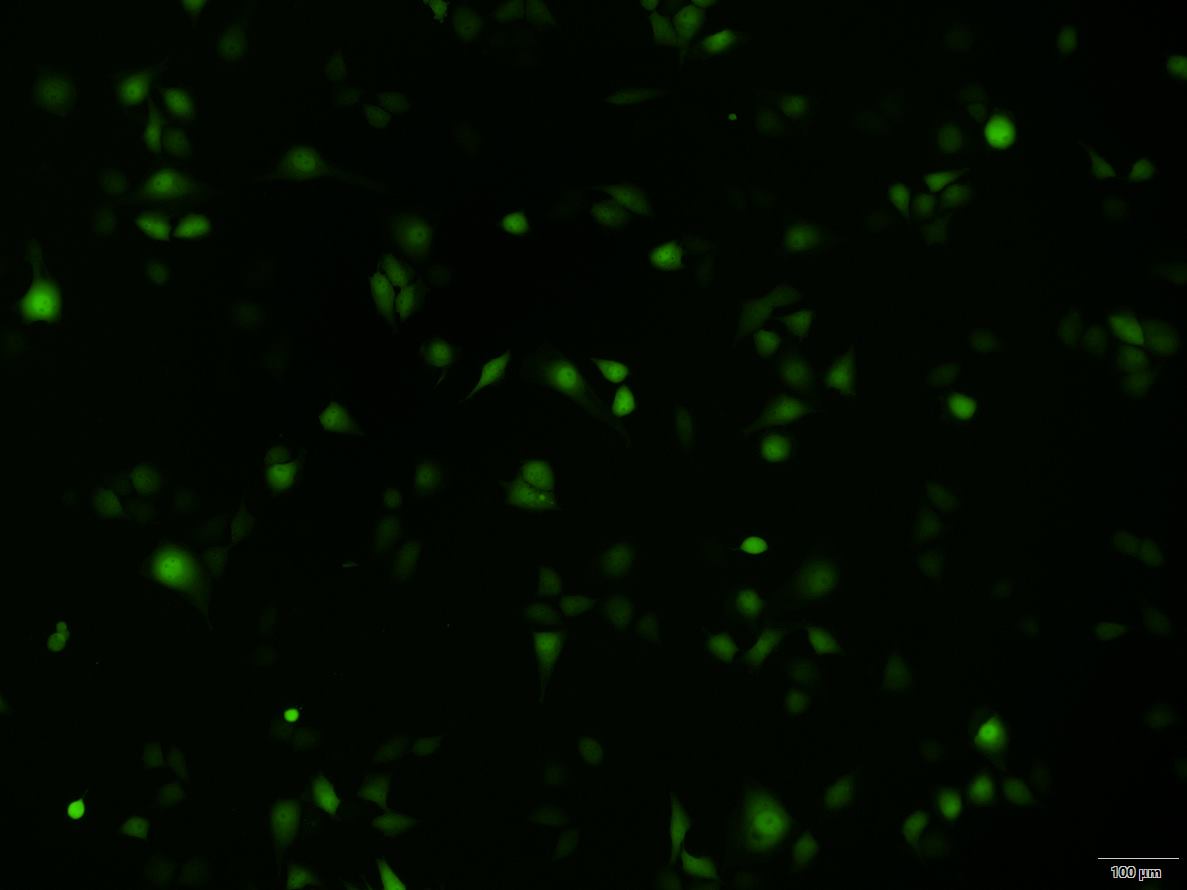

Supplement: Supplementary file 8 [file DataSheet_5.zip › Data Sheet 5/FigS1C/2-Scrambled-day3.jpg]

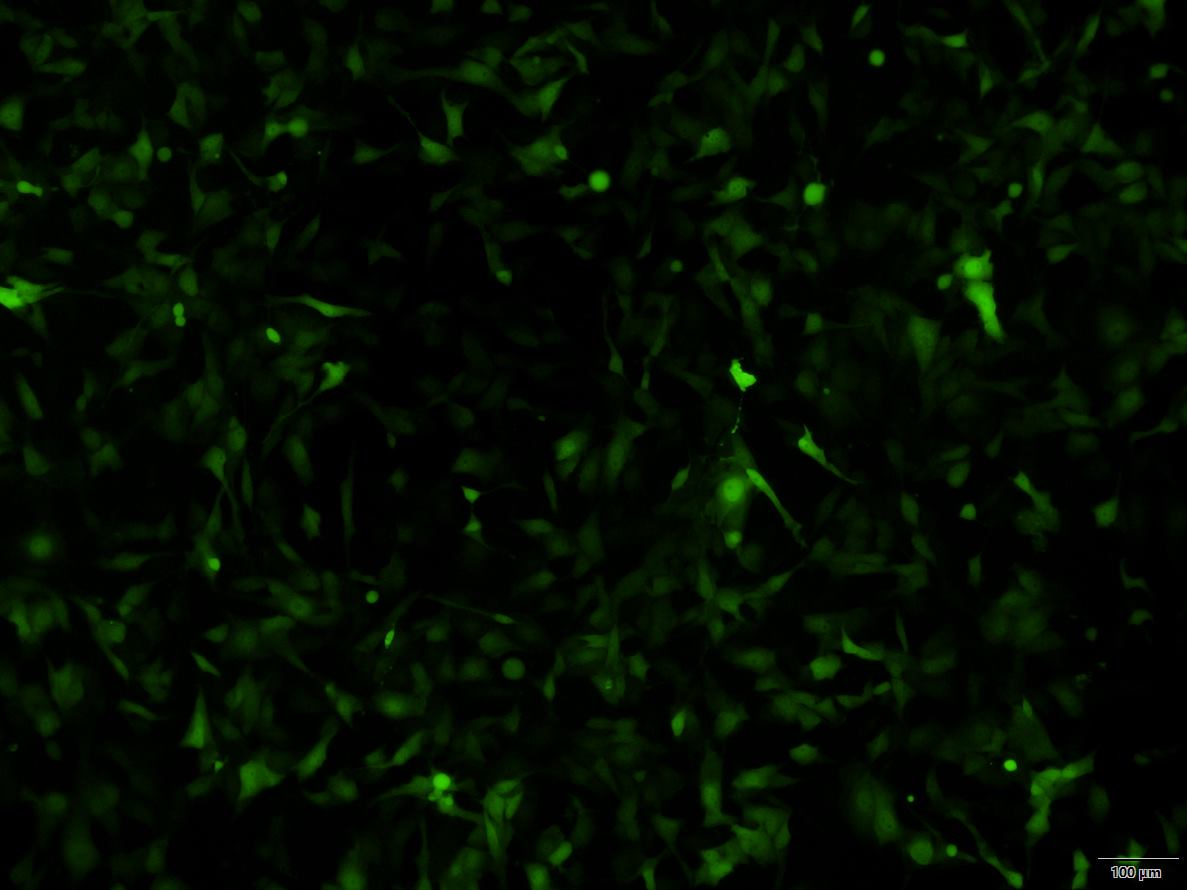

Supplement: Supplementary file 8 [file DataSheet_5.zip › Data Sheet 5/FigS1C/2-Scrambled-day4.jpg]

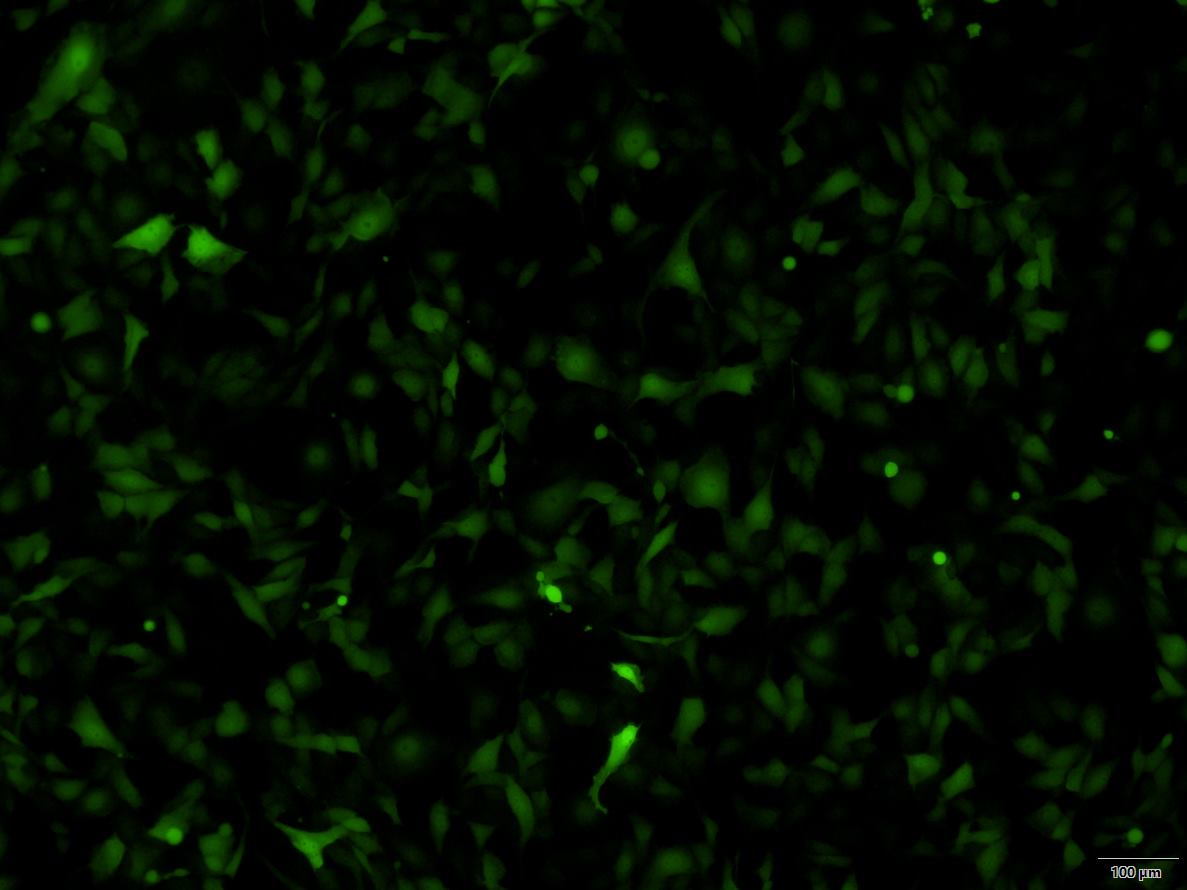

Supplement: Supplementary file 8 [file DataSheet_5.zip › Data Sheet 5/FigS1C/2-Scrambled-day5.jpg]

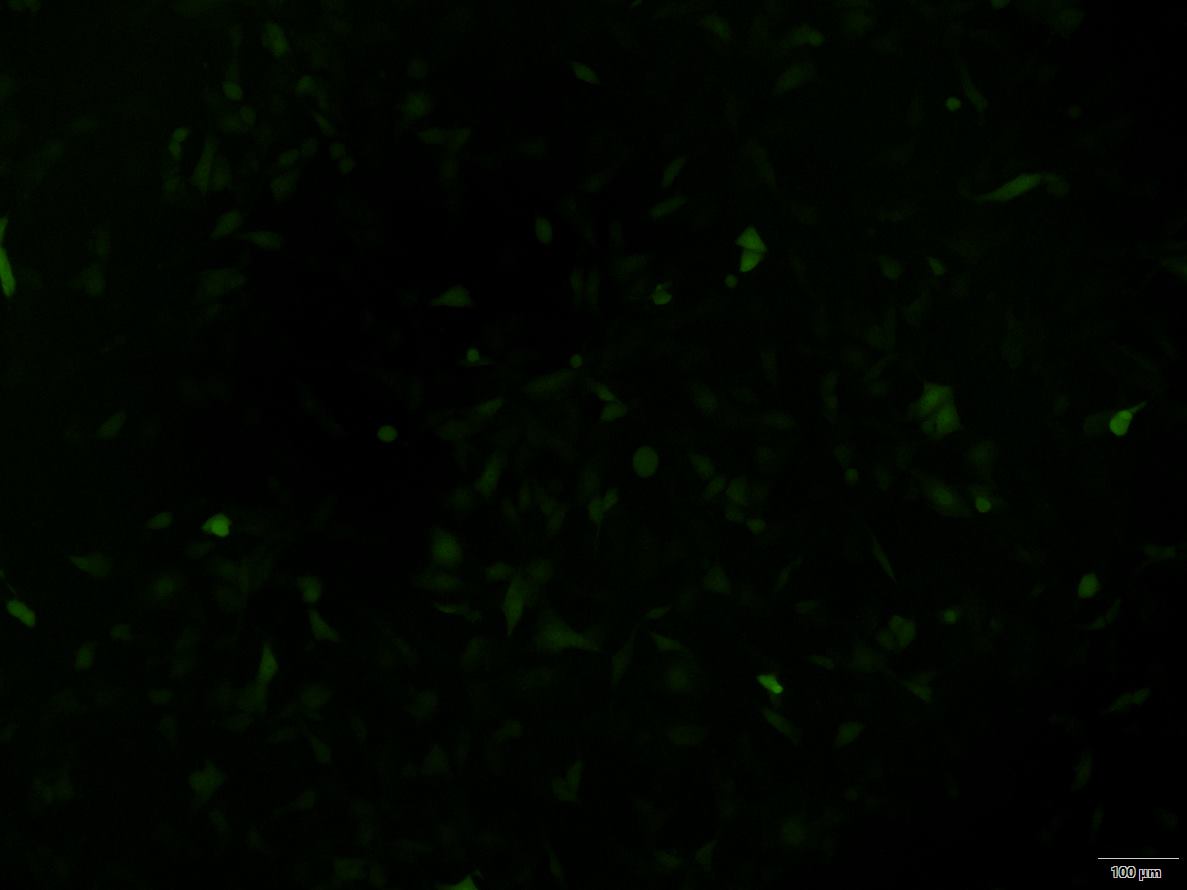

Supplement: Supplementary file 8 [file DataSheet_5.zip › Data Sheet 5/FigS1C/2-SiAC009948.5-day1.jpg]

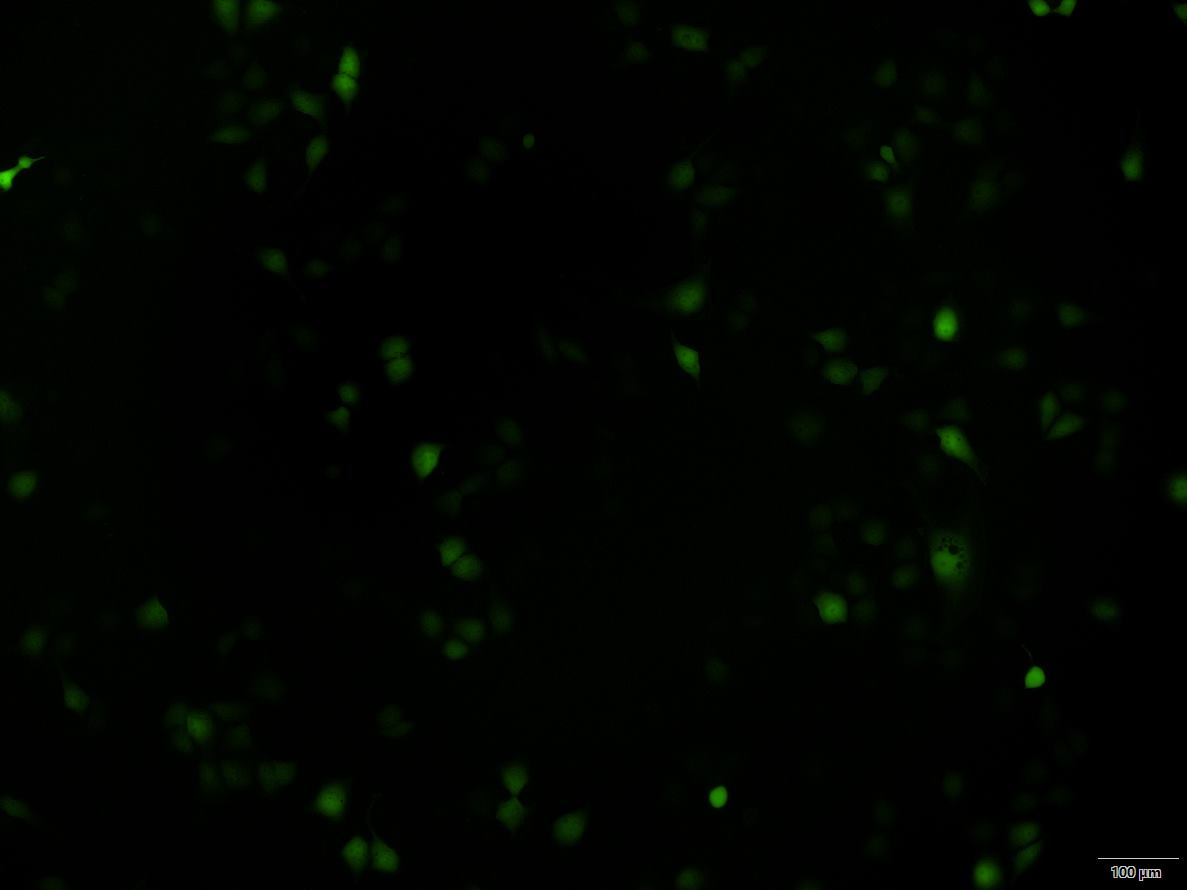

Supplement: Supplementary file 8 [file DataSheet_5.zip › Data Sheet 5/FigS1C/2-SiAC009948.5-day2.jpg]

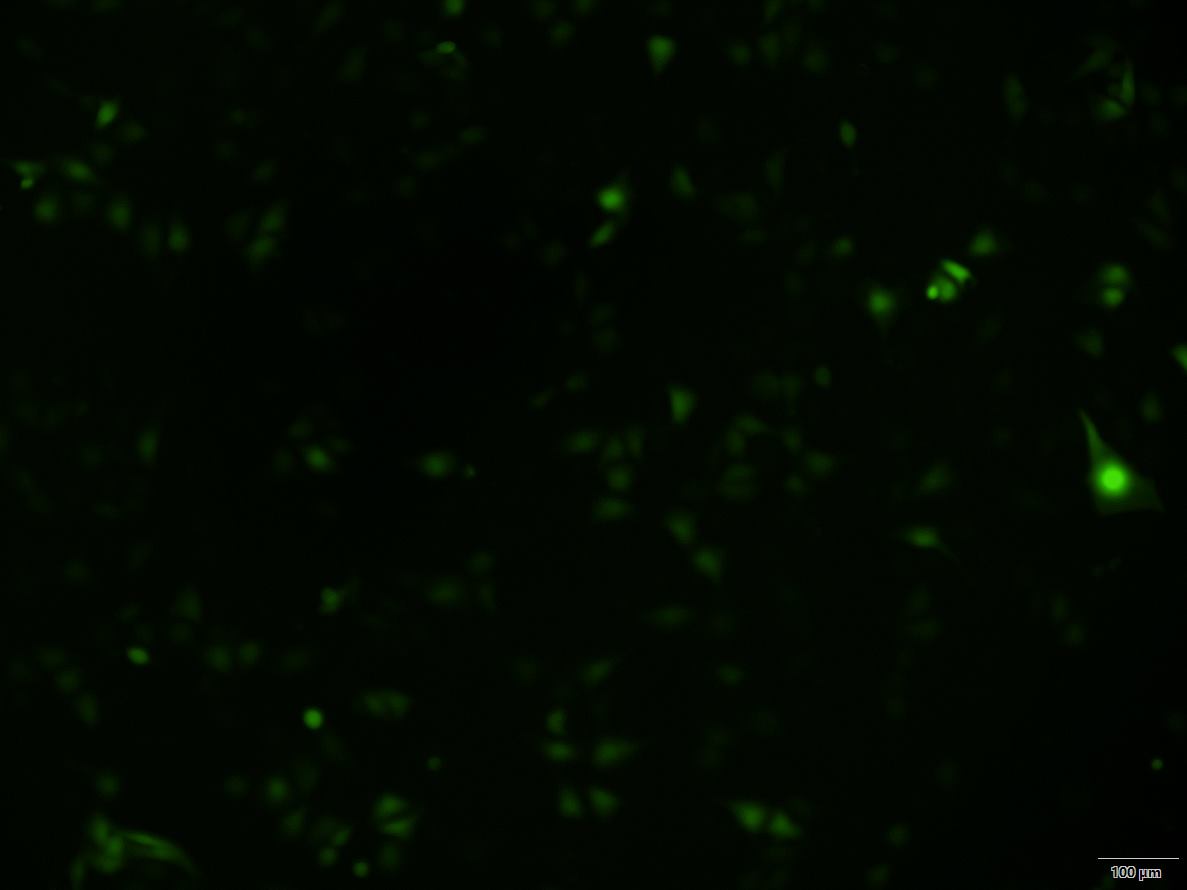

Supplement: Supplementary file 8 [file DataSheet_5.zip › Data Sheet 5/FigS1C/2-SiAC009948.5-day3.jpg]

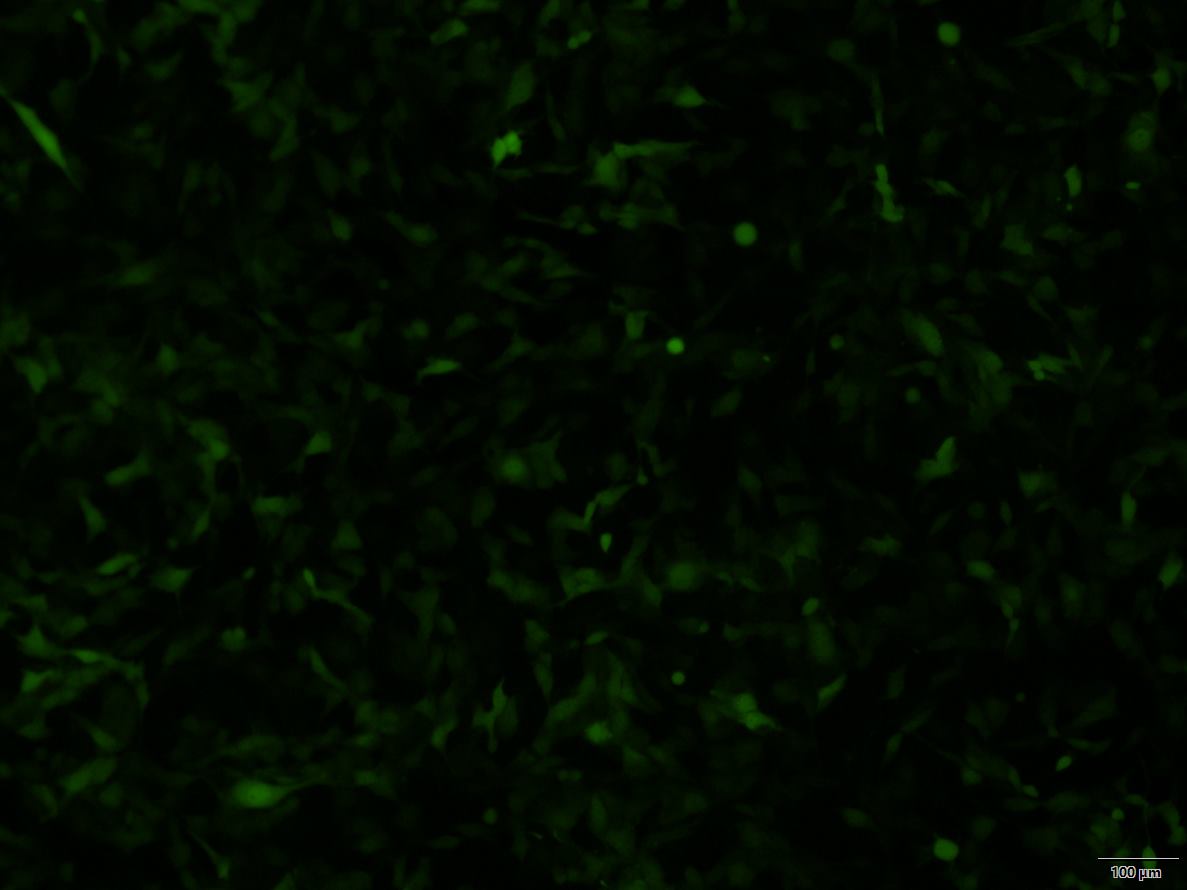

Supplement: Supplementary file 8 [file DataSheet_5.zip › Data Sheet 5/FigS1C/2-SiAC009948.5-day4.jpg]

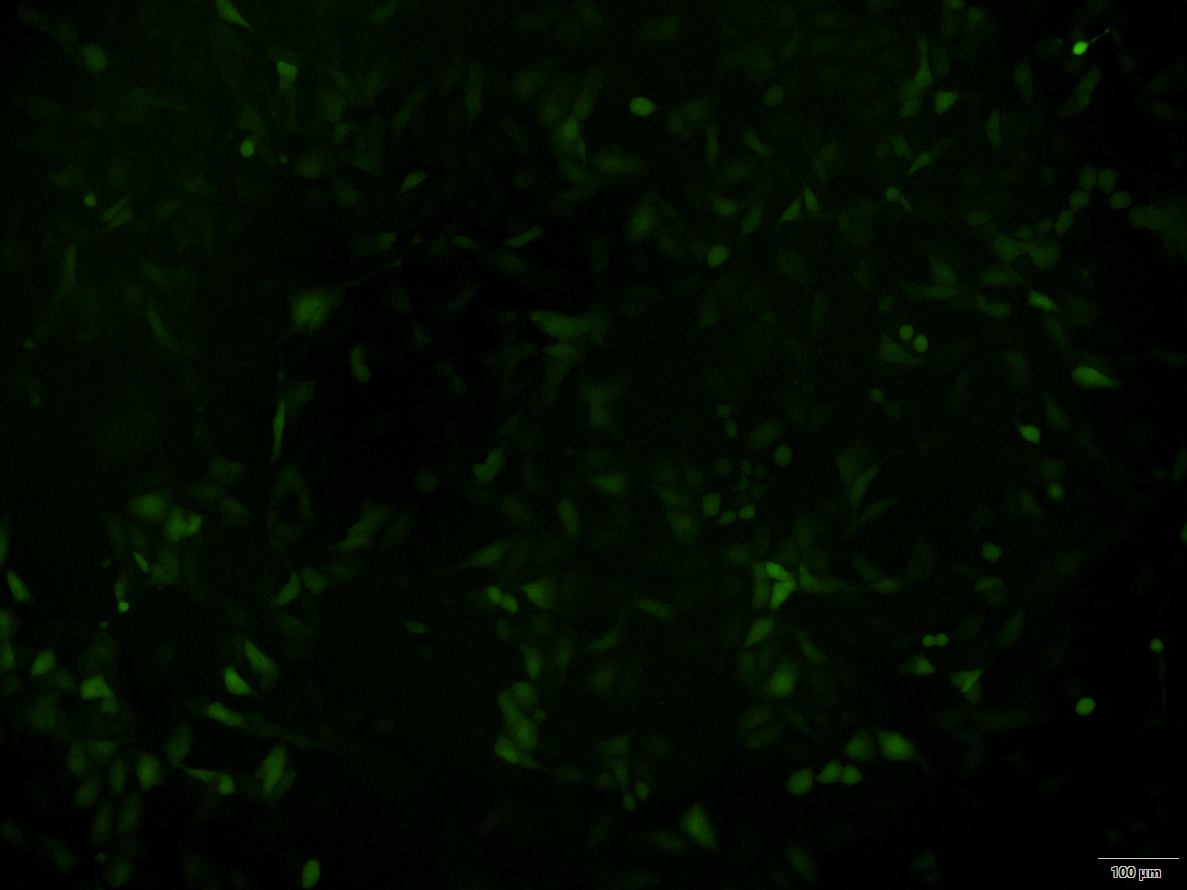

Supplement: Supplementary file 8 [file DataSheet_5.zip › Data Sheet 5/FigS1C/2-SiAC009948.5-day5.jpg]

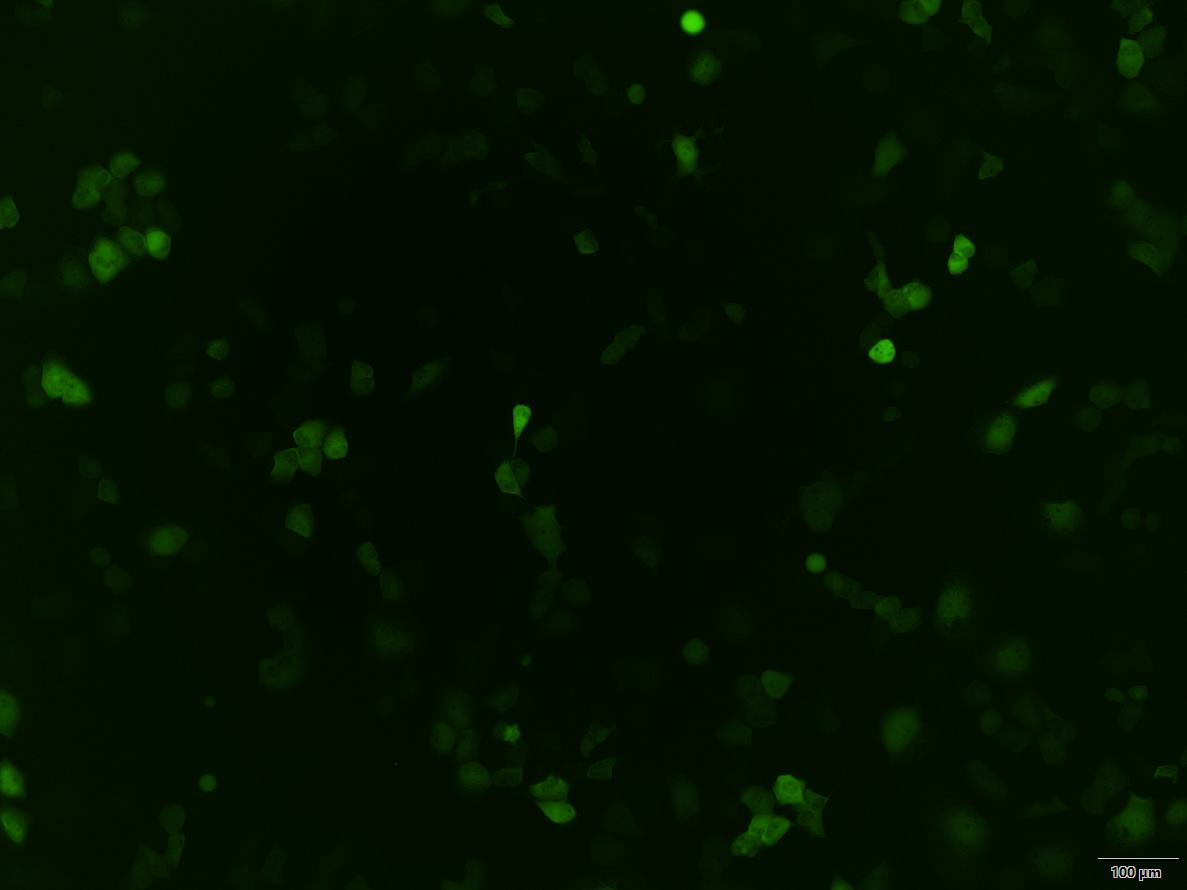

Supplement: Supplementary file 8 [file DataSheet_5.zip › Data Sheet 5/FigS1C/3-NC-day1-AC009948.5.jpg]

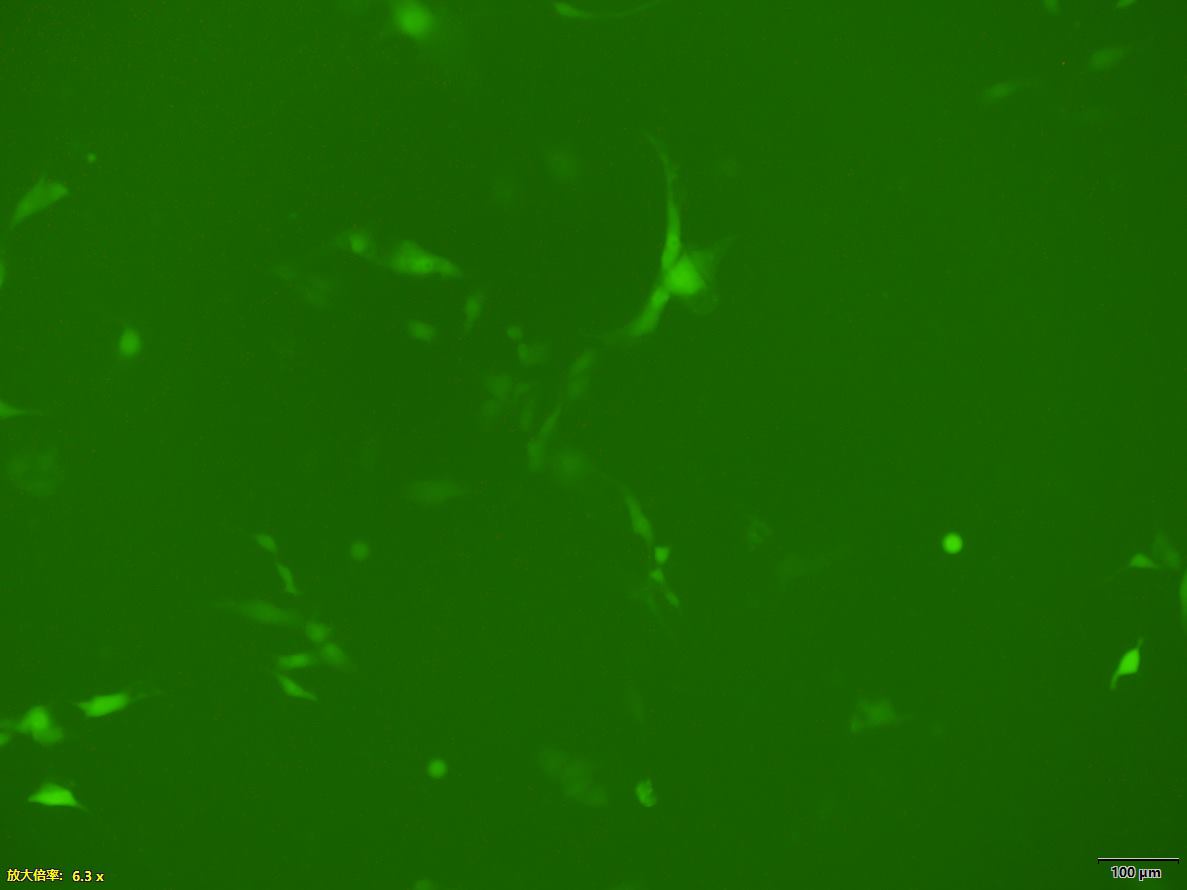

Supplement: Supplementary file 8 [file DataSheet_5.zip › Data Sheet 5/FigS1C/3-NC-day2-AC009948.5.jpg]

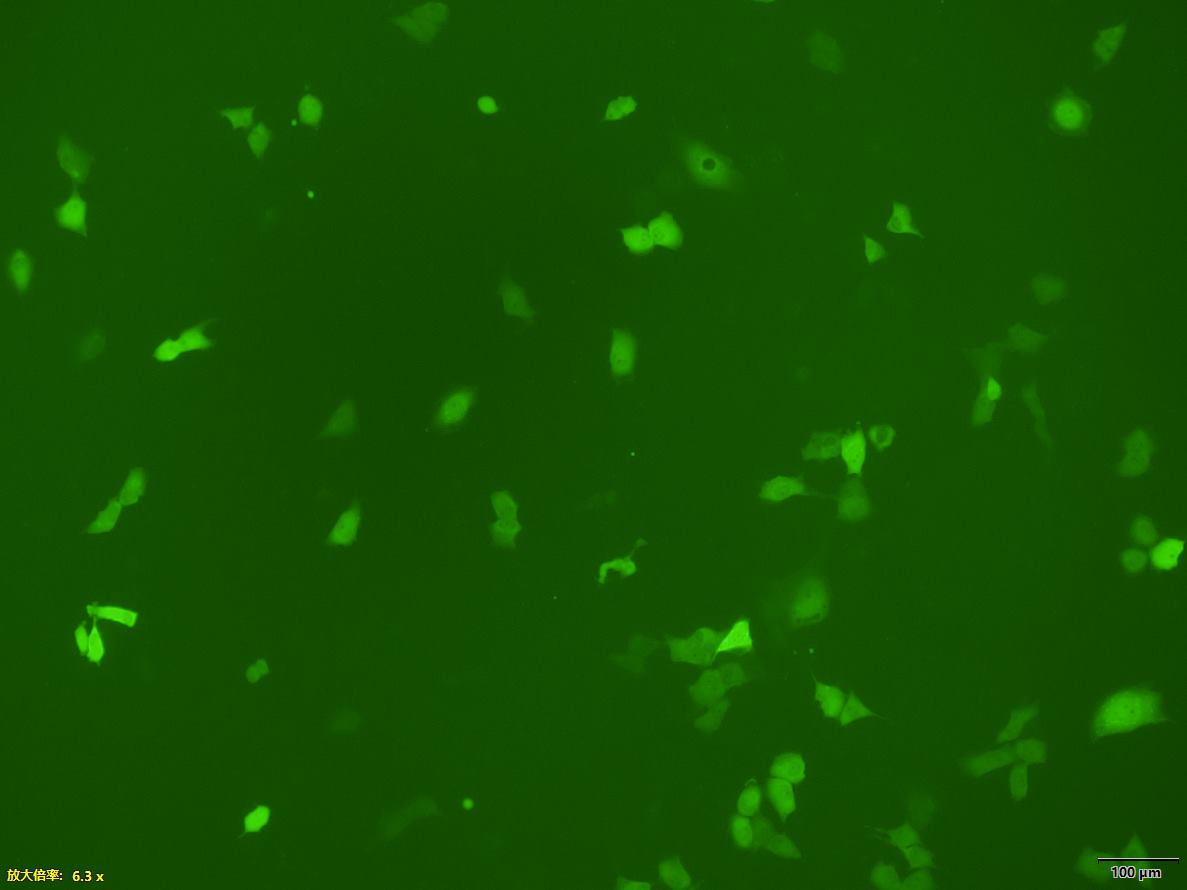

Supplement: Supplementary file 8 [file DataSheet_5.zip › Data Sheet 5/FigS1C/3-NC-day3-AC009948.5.jpg]

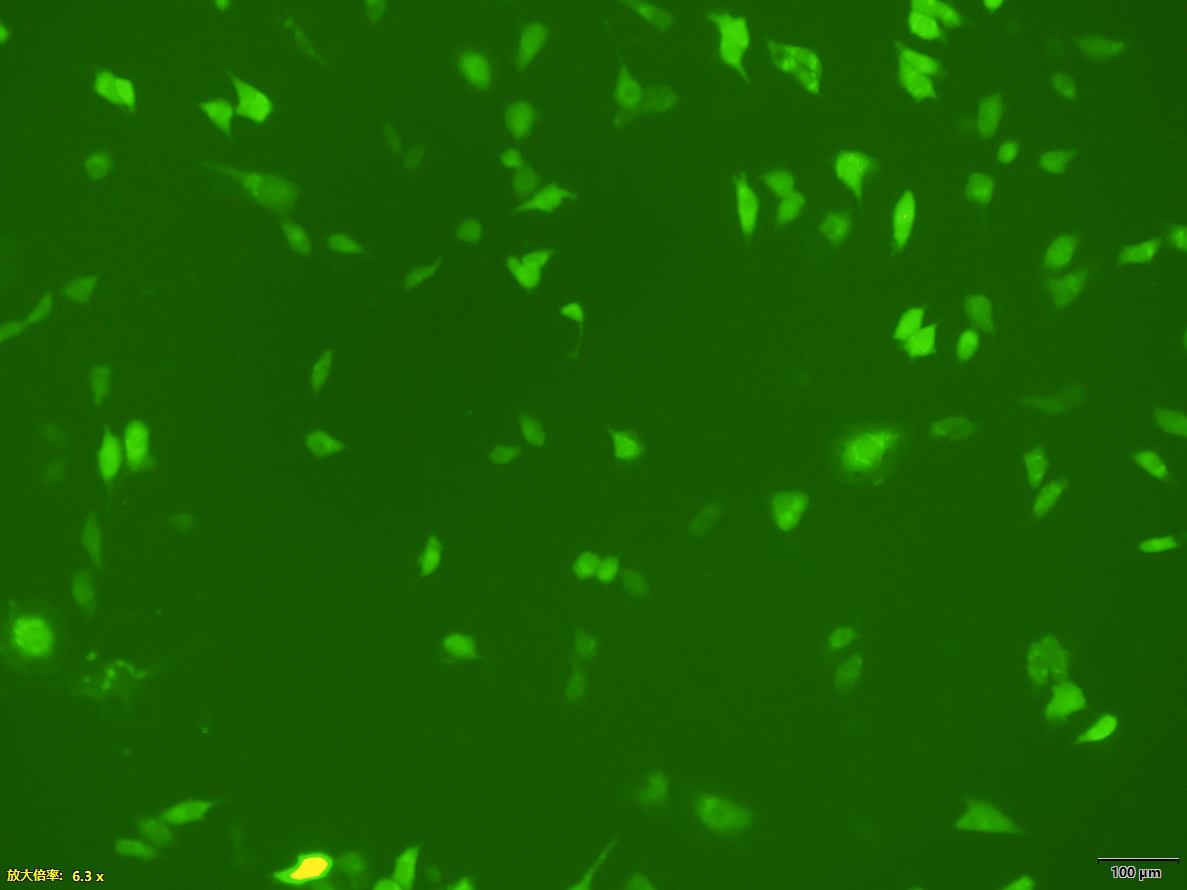

Supplement: Supplementary file 8 [file DataSheet_5.zip › Data Sheet 5/FigS1C/3-NC-day4-AC009948.5.jpg]

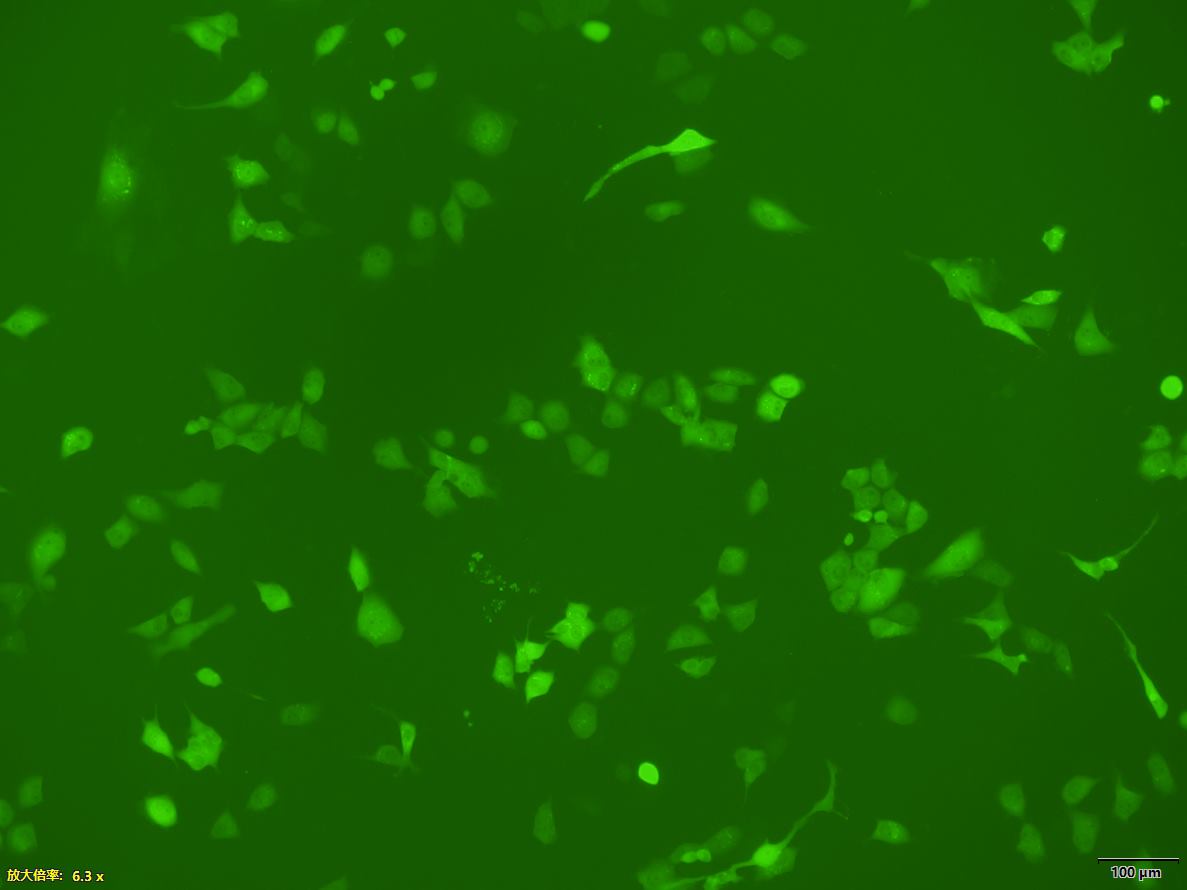

Supplement: Supplementary file 8 [file DataSheet_5.zip › Data Sheet 5/FigS1C/3-NC-day5-AC009948.5.jpg]

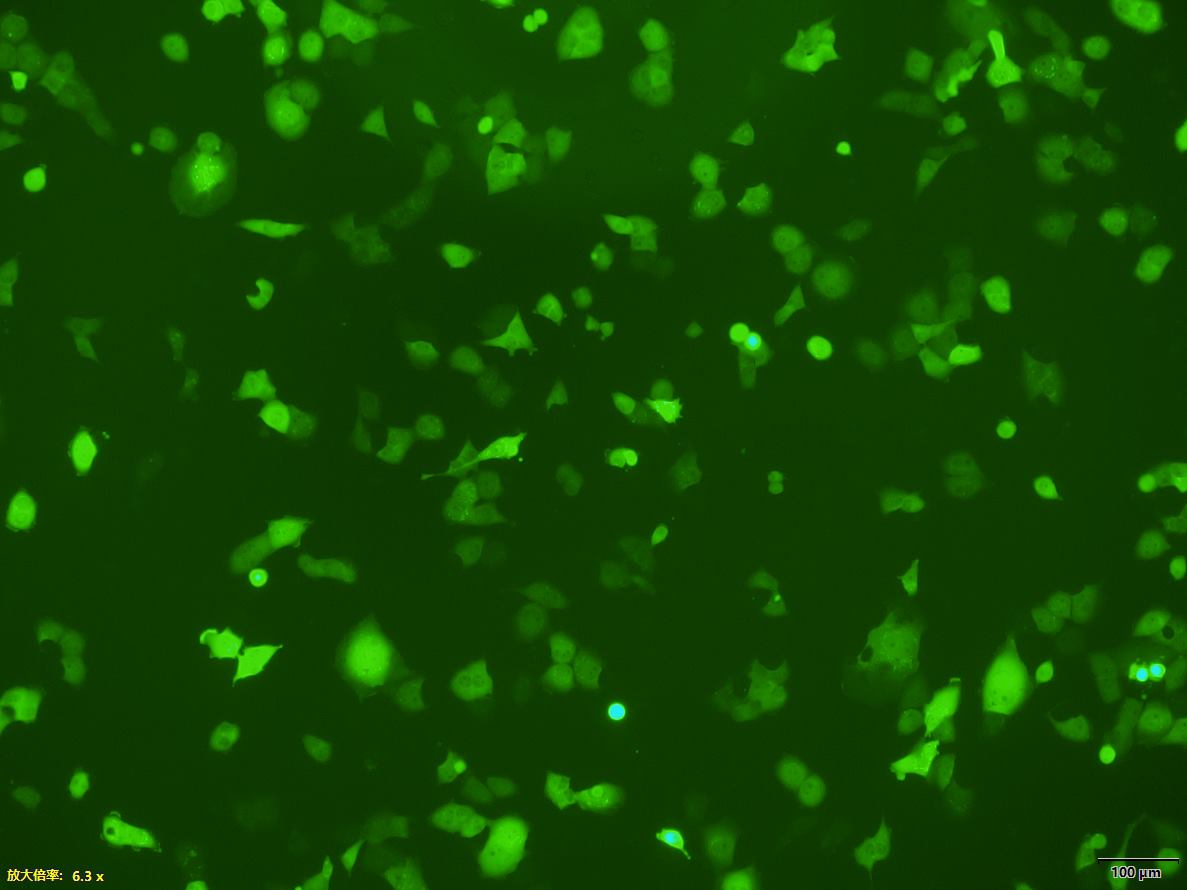

Supplement: Supplementary file 8 [file DataSheet_5.zip › Data Sheet 5/FigS1C/3-over-AC009948.5-day1.jpg]

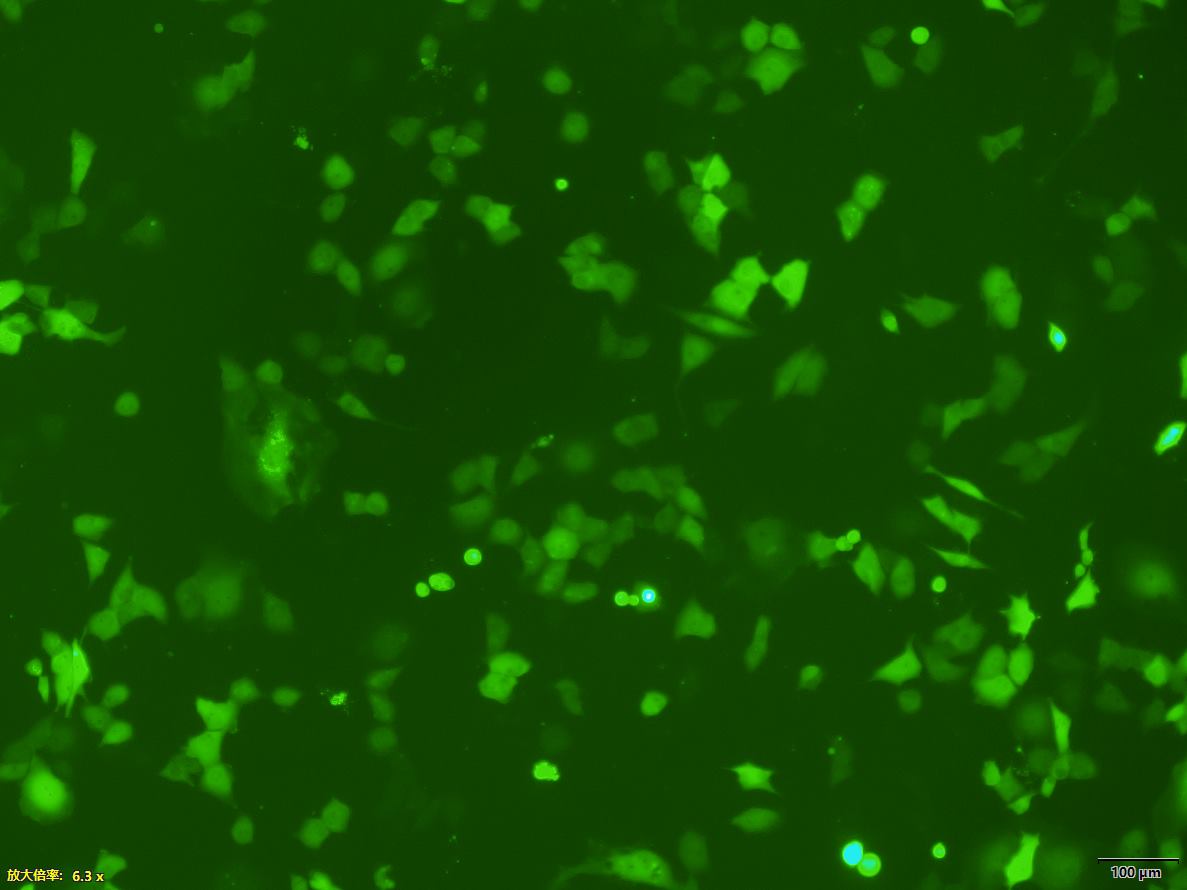

Supplement: Supplementary file 8 [file DataSheet_5.zip › Data Sheet 5/FigS1C/3-over-AC009948.5-day2.jpg]

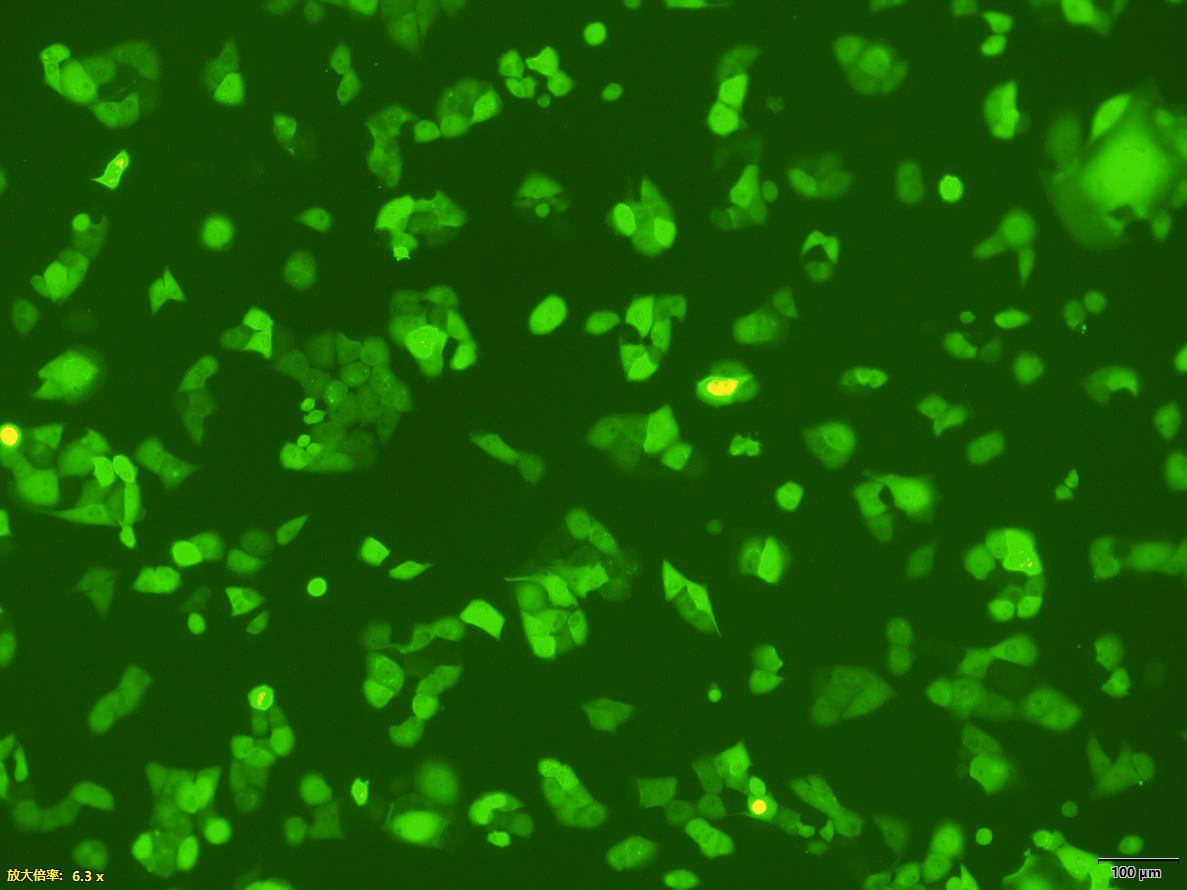

Supplement: Supplementary file 8 [file DataSheet_5.zip › Data Sheet 5/FigS1C/3-over-AC009948.5-day3.jpg]

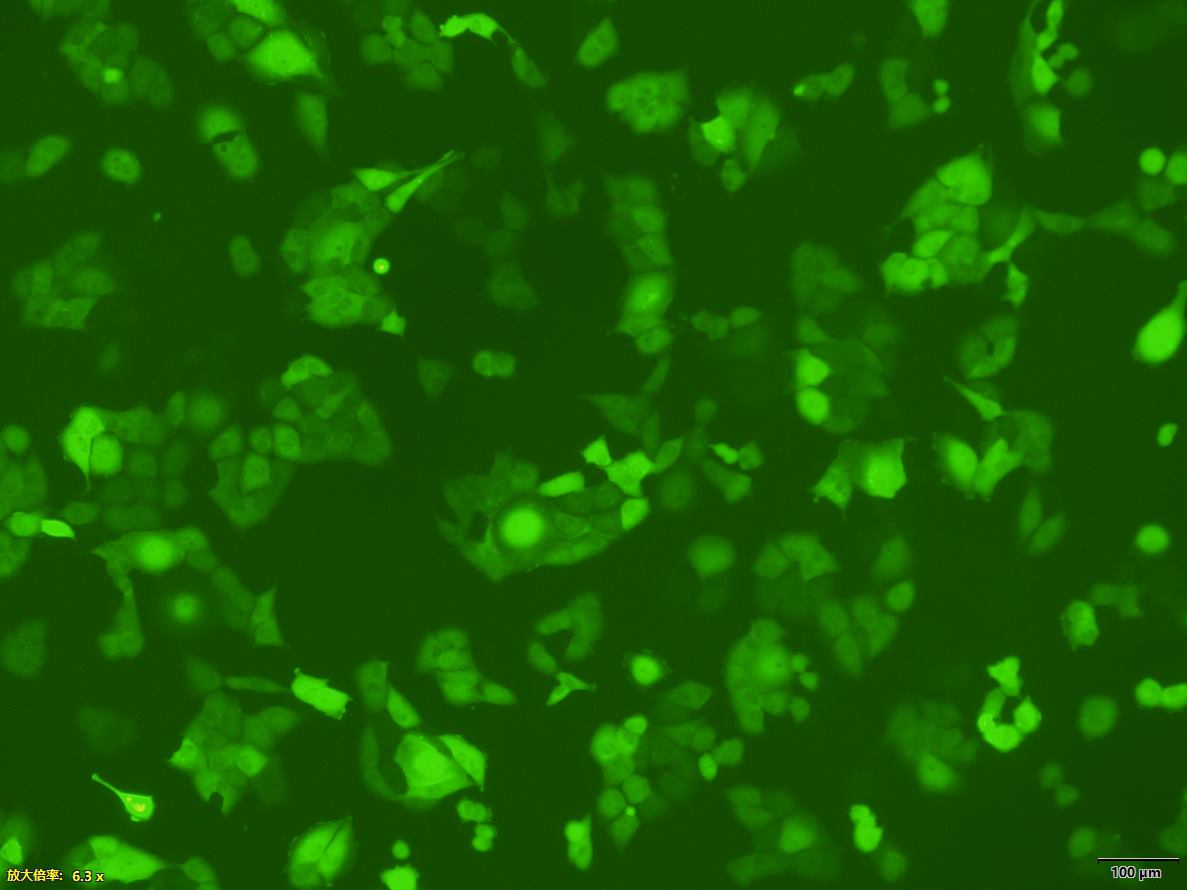

Supplement: Supplementary file 8 [file DataSheet_5.zip › Data Sheet 5/FigS1C/3-over-AC009948.5-day4.jpg]

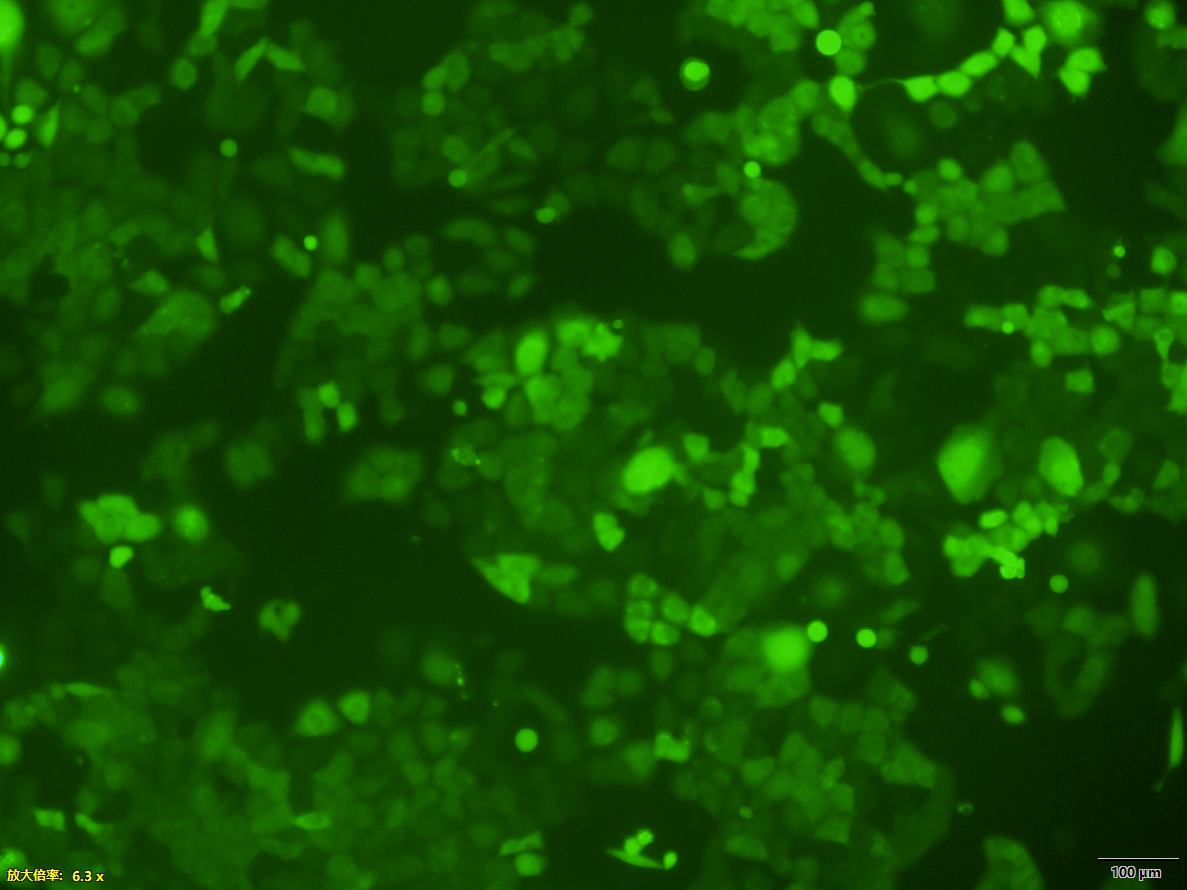

Supplement: Supplementary file 8 [file DataSheet_5.zip › Data Sheet 5/FigS1C/3-over-AC009948.5-day5.jpg]

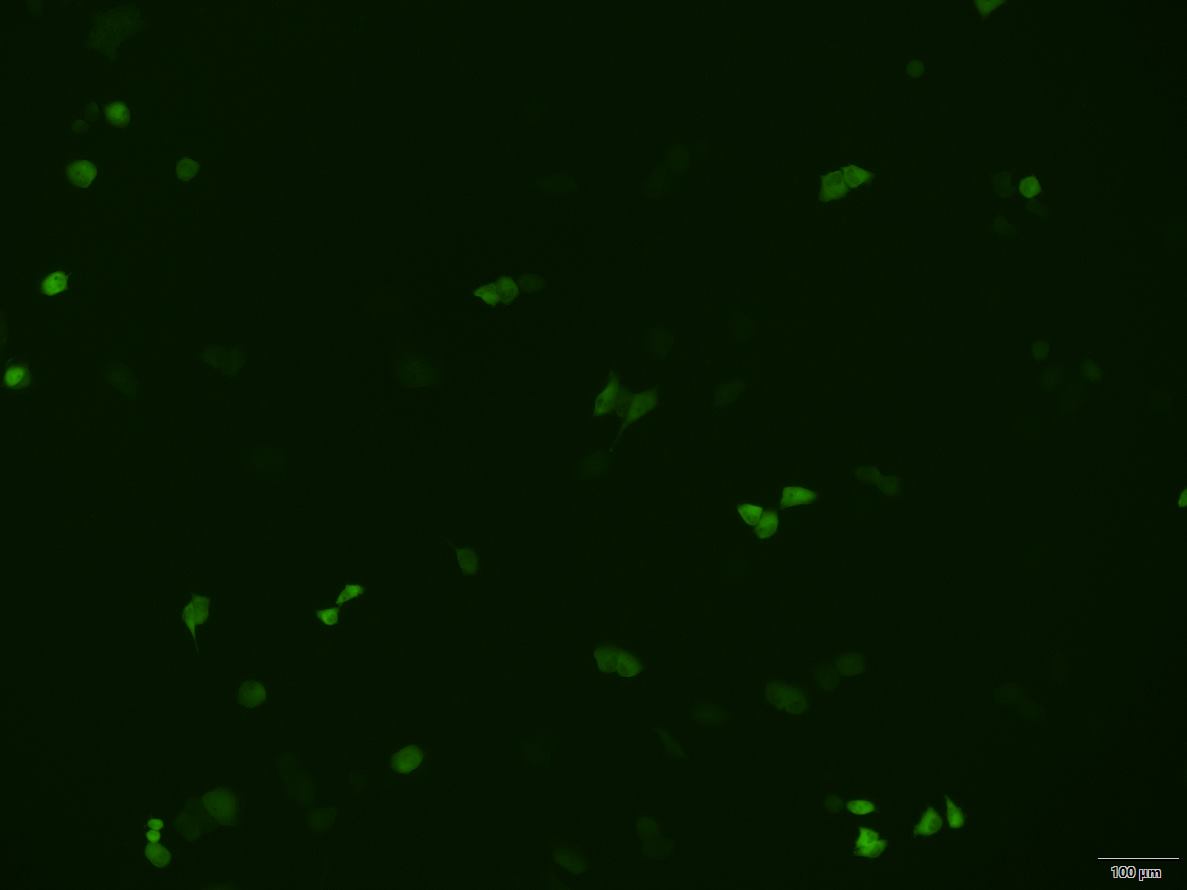

Supplement: Supplementary file 8 [file DataSheet_5.zip › Data Sheet 5/FigS1C/3-Scrambled-day1.jpg]

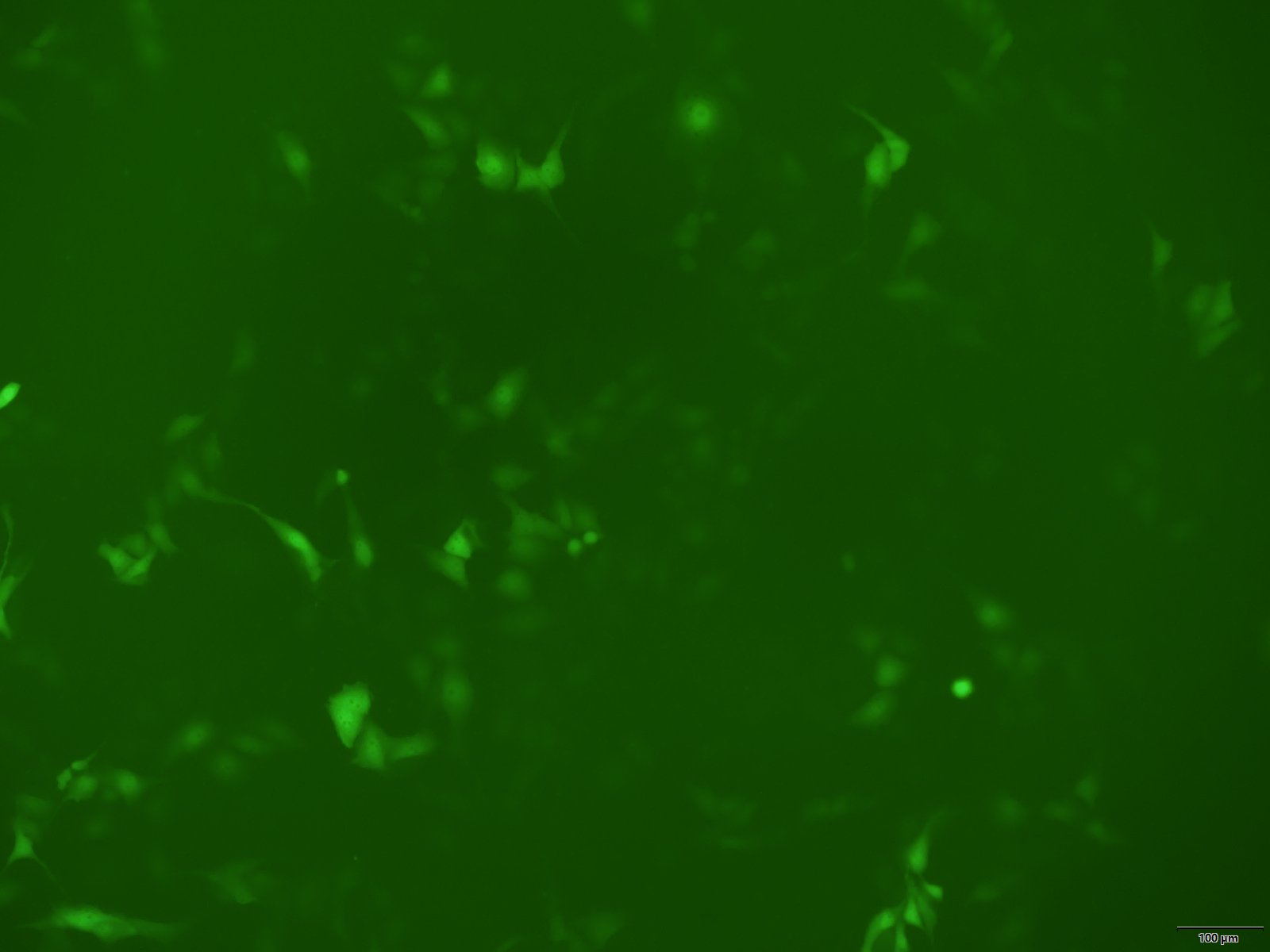

Supplement: Supplementary file 8 [file DataSheet_5.zip › Data Sheet 5/FigS1C/3-Scrambled-day2.jpg]

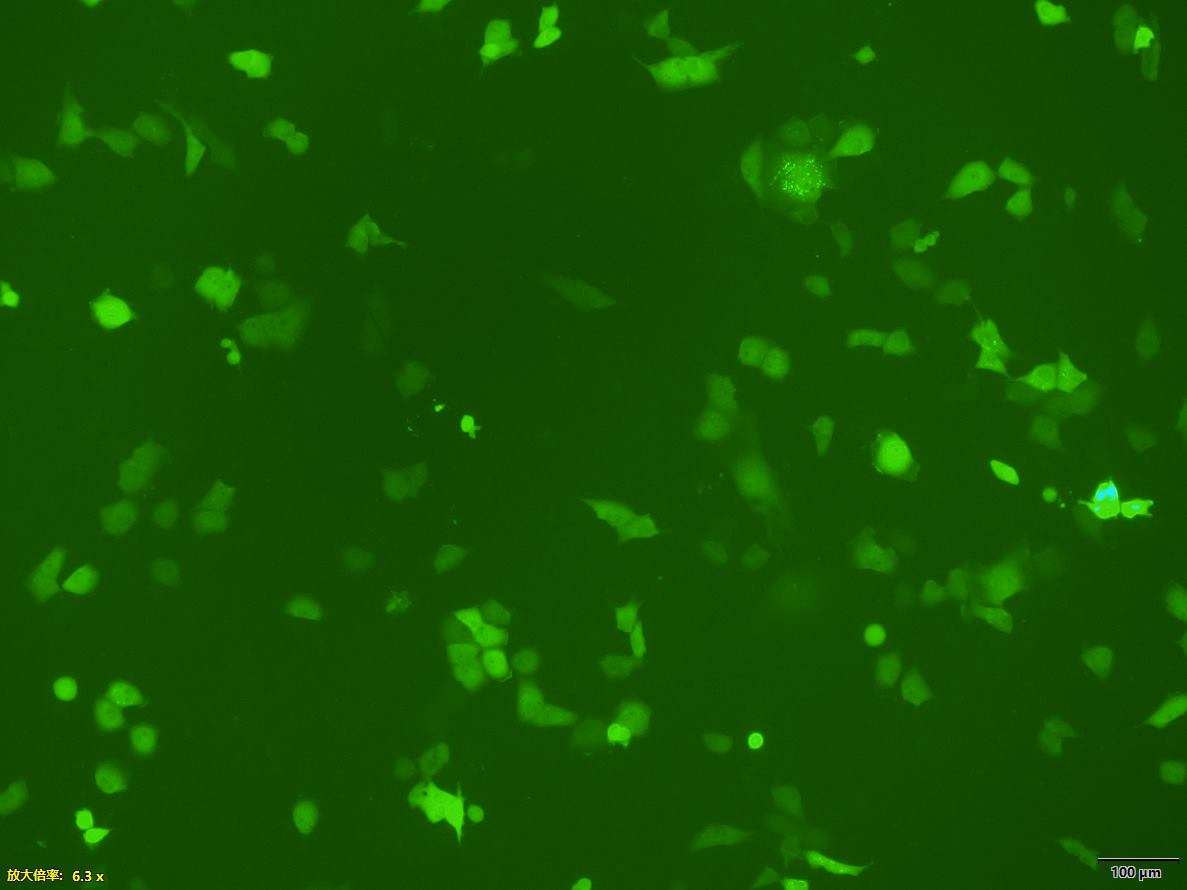

Supplement: Supplementary file 8 [file DataSheet_5.zip › Data Sheet 5/FigS1C/3-Scrambled-day3.jpg]

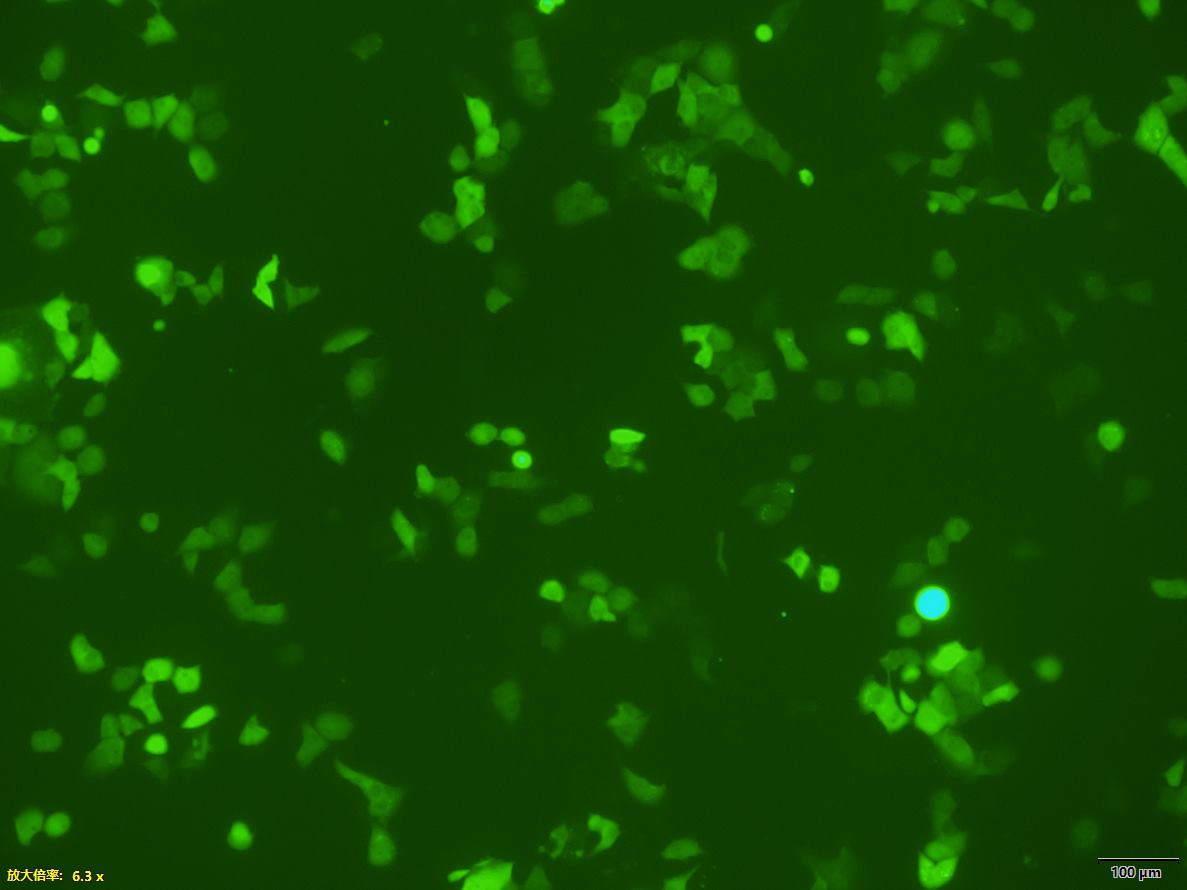

Supplement: Supplementary file 8 [file DataSheet_5.zip › Data Sheet 5/FigS1C/3-Scrambled-day4.jpg]

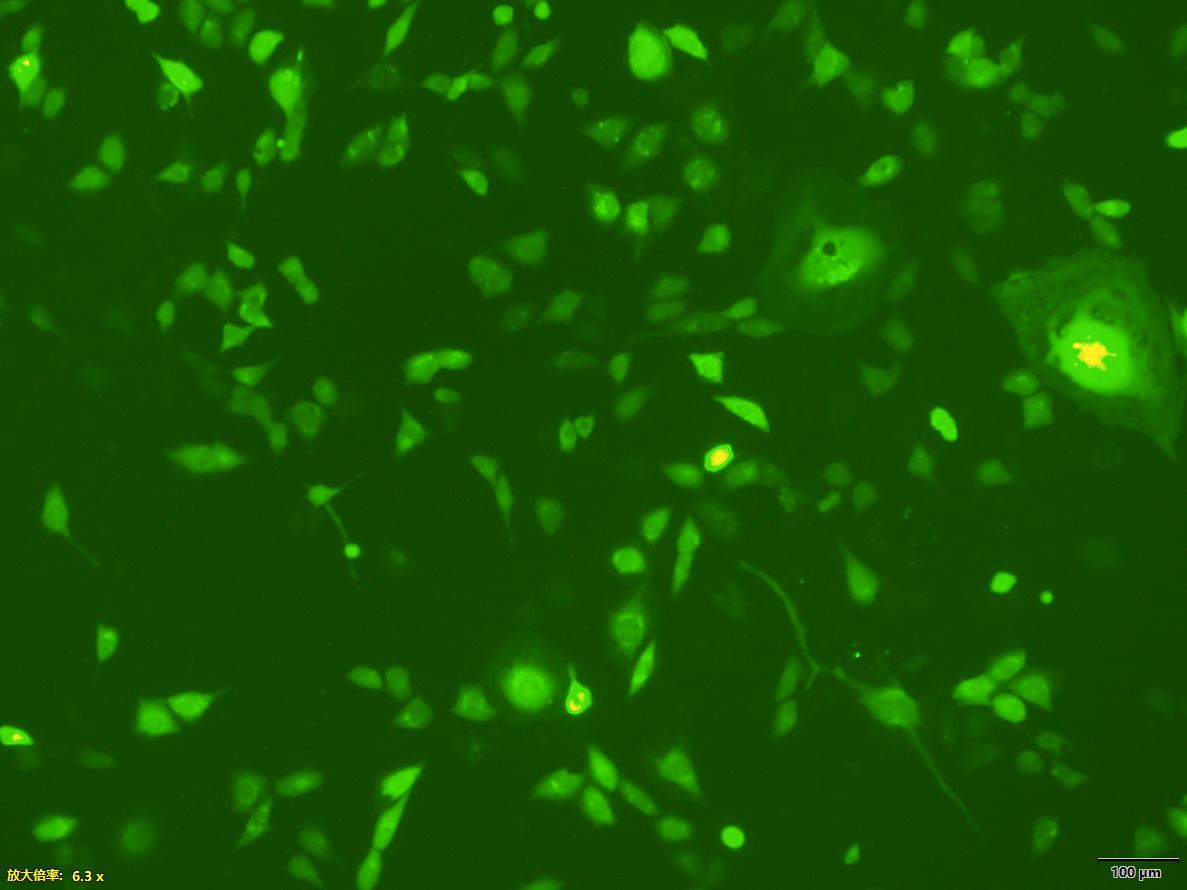

Supplement: Supplementary file 8 [file DataSheet_5.zip › Data Sheet 5/FigS1C/3-Scrambled-day5.jpg]

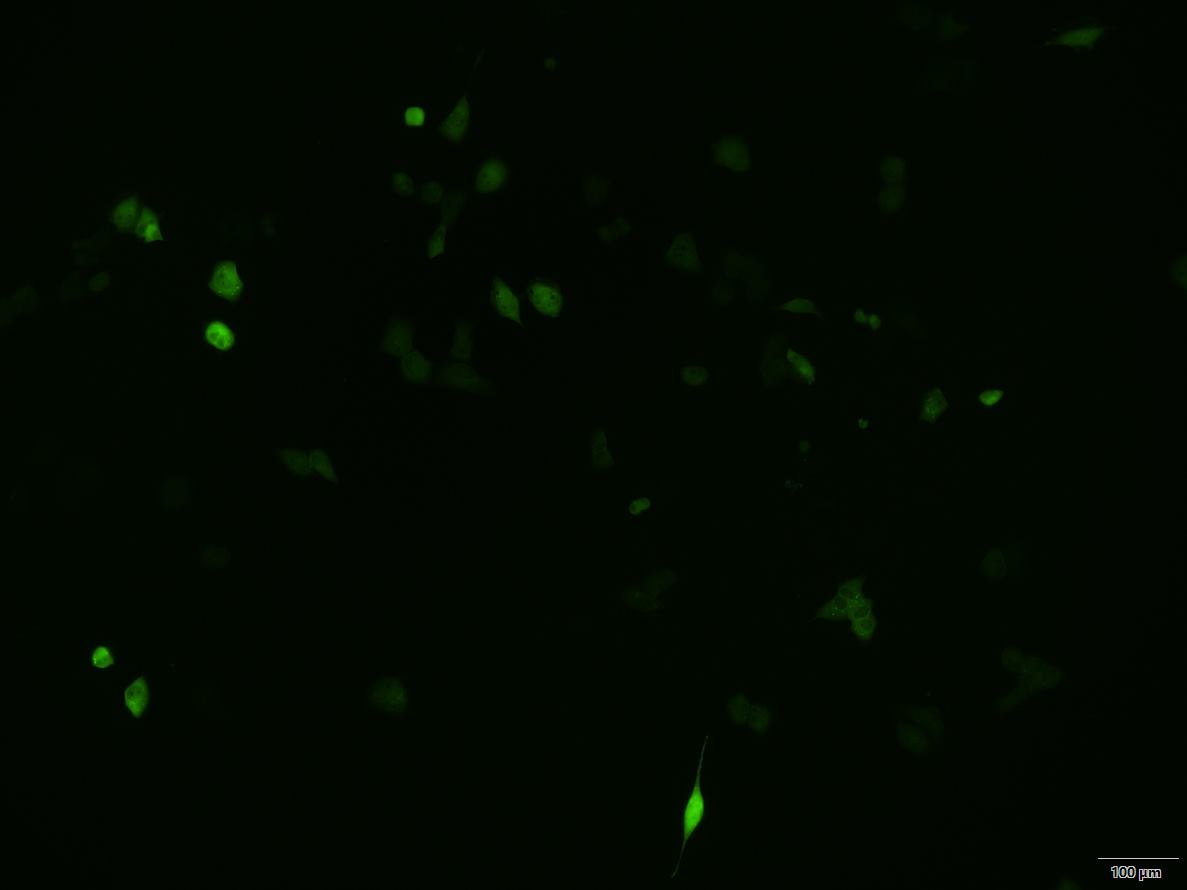

Supplement: Supplementary file 8 [file DataSheet_5.zip › Data Sheet 5/FigS1C/3-SiAC009948.5-day1.jpg]
