# Supplementary figures and images for: LncRNA-AC009948.5 promotes invasion and metastasis of lung adenocarcinoma by binding to miR-186-5p (part 4 of 4)
Source: Front Oncol. 2022 Aug 19;12:949951. doi: 10.3389/fonc.2022.949951 (PMC9437580; doi:10.3389/fonc.2022.949951)

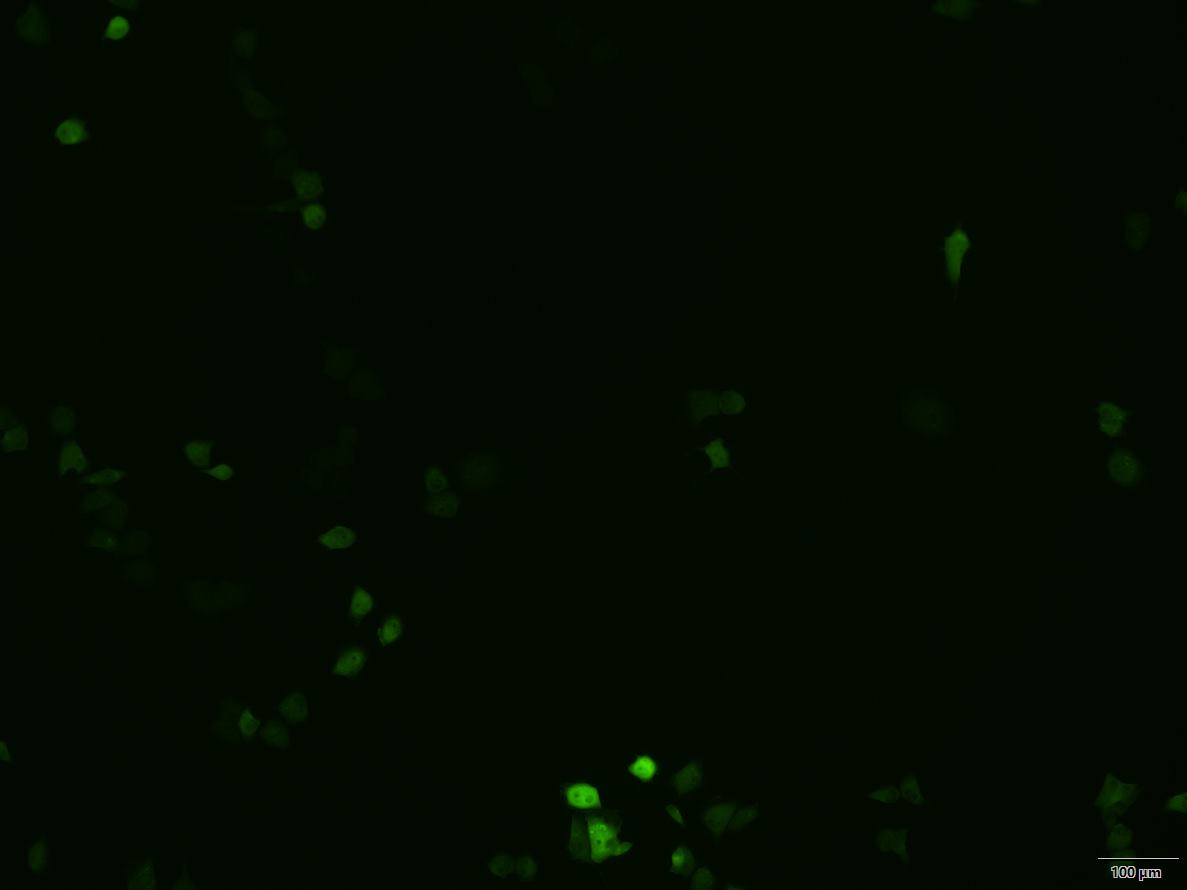

Supplement: Supplementary file 8 [file DataSheet_5.zip › Data Sheet 5/FigS1C/3-SiAC009948.5-day2.jpg]

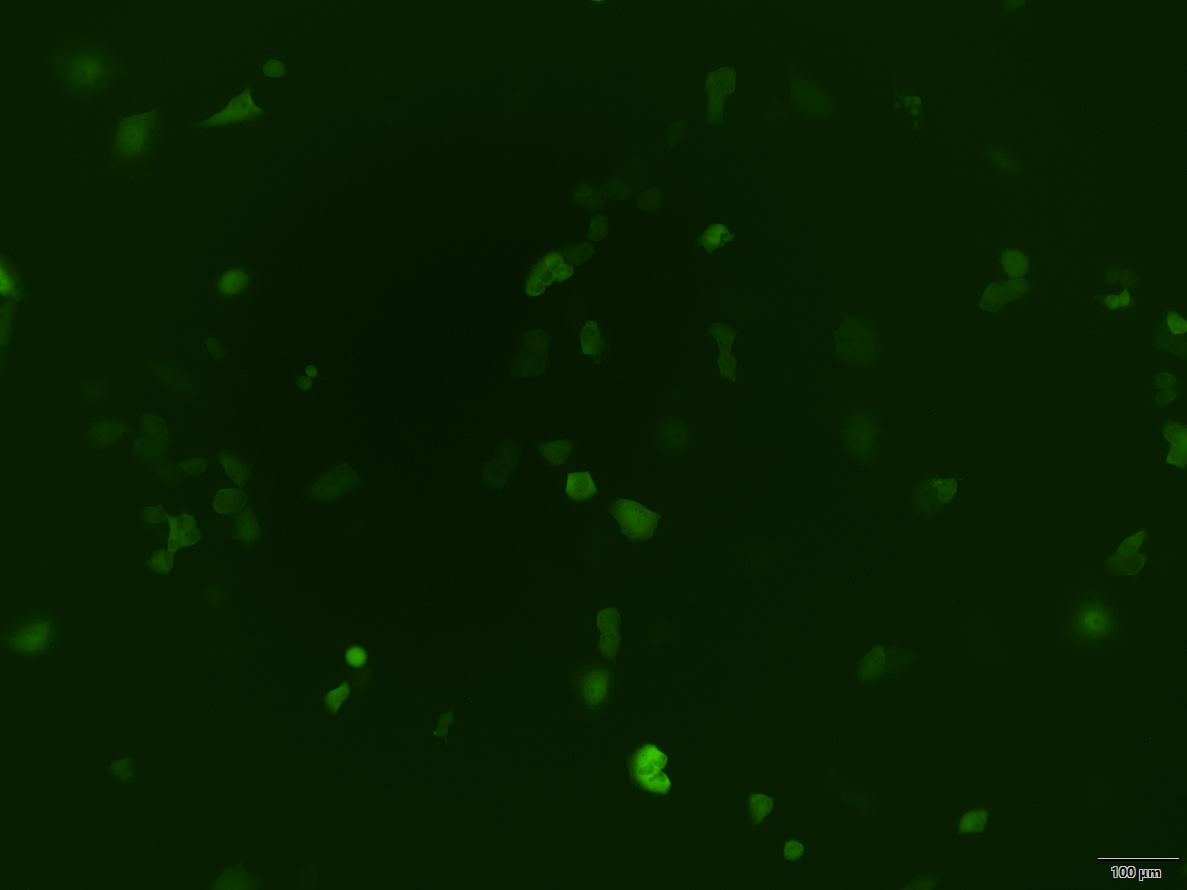

Supplement: Supplementary file 8 [file DataSheet_5.zip › Data Sheet 5/FigS1C/3-SiAC009948.5-day3.jpg]

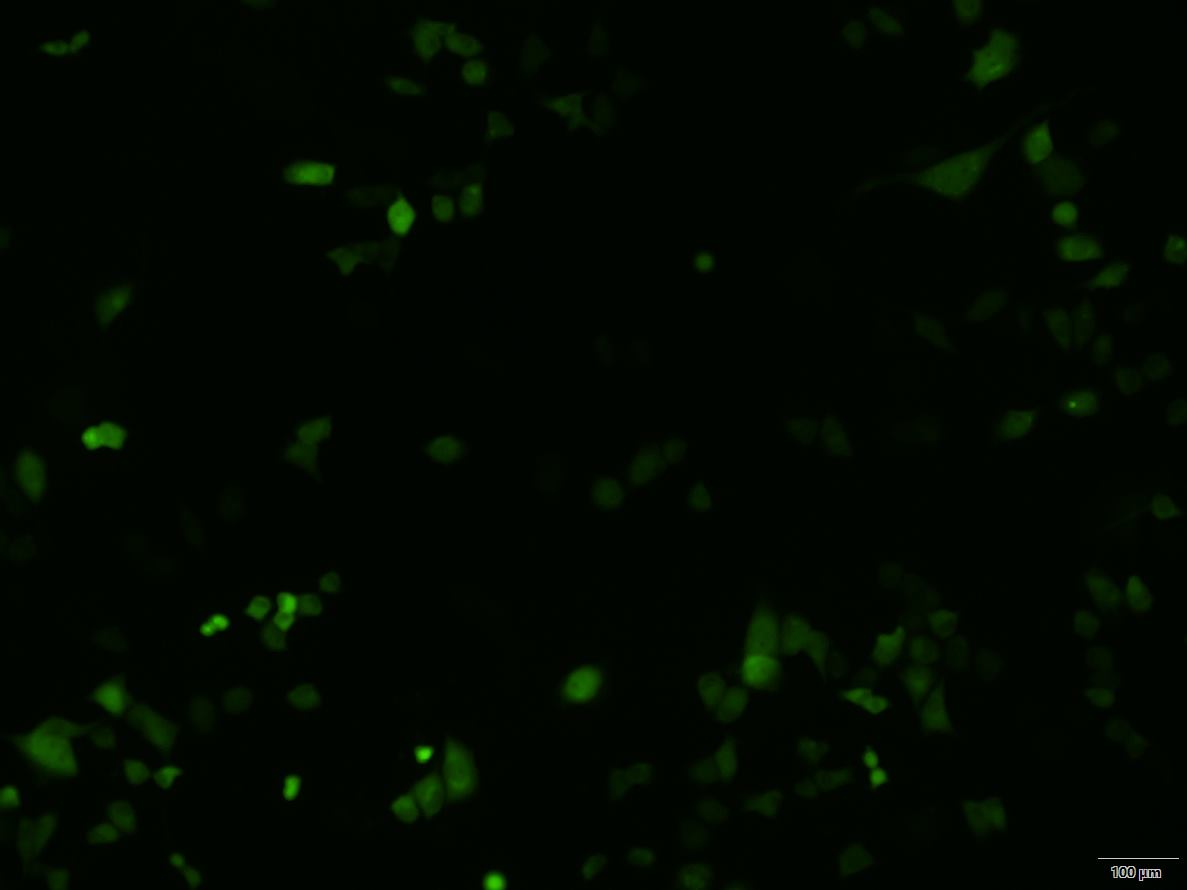

Supplement: Supplementary file 8 [file DataSheet_5.zip › Data Sheet 5/FigS1C/3-SiAC009948.5-day4.jpg]

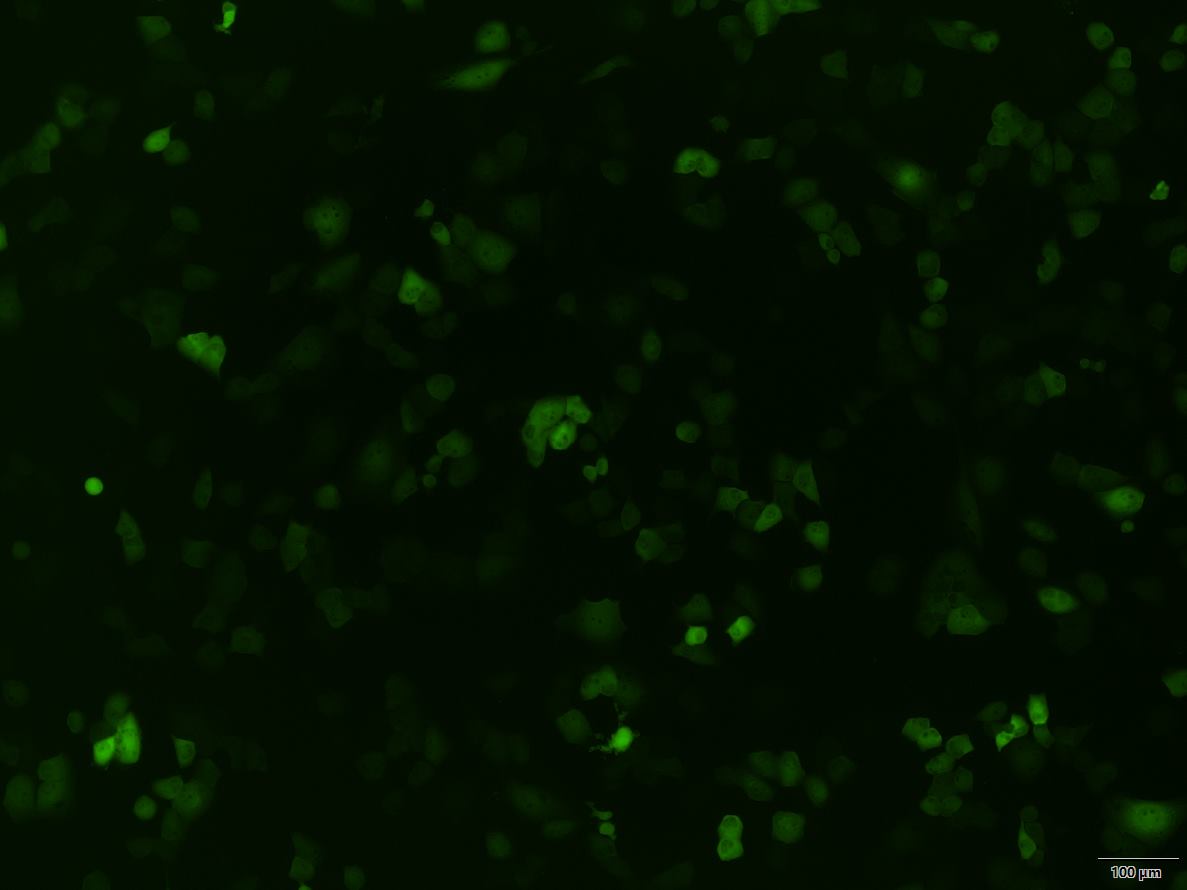

Supplement: Supplementary file 8 [file DataSheet_5.zip › Data Sheet 5/FigS1C/3-SiAC009948.5-day5.jpg]

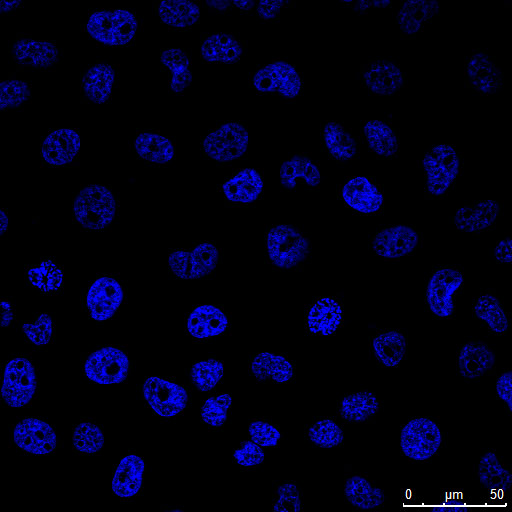

Supplement: Supplementary file 8 [file DataSheet_5.zip › Data Sheet 5/FigS1D/1-1-1-NC-EDU-H1299_Series007_z0_ch01.jpg]

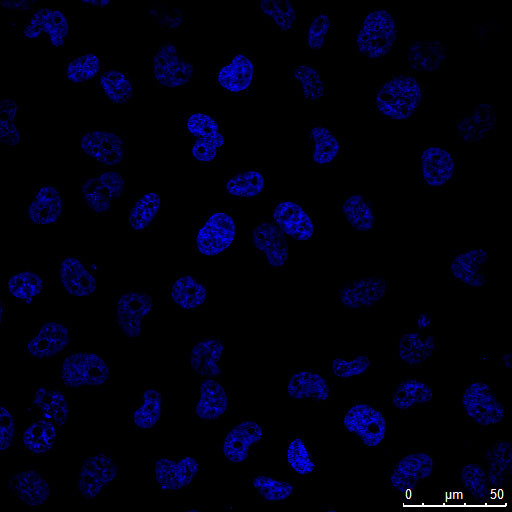

Supplement: Supplementary file 8 [file DataSheet_5.zip › Data Sheet 5/FigS1D/1-1-1-over-AC009948.5-EDU-H1299_Series051_z0_ch01.jpg]

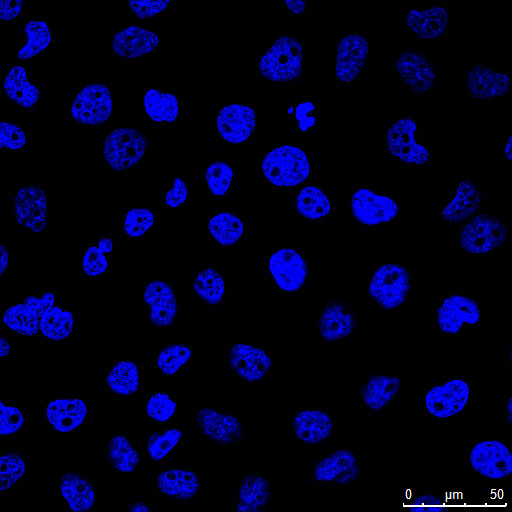

Supplement: Supplementary file 8 [file DataSheet_5.zip › Data Sheet 5/FigS1D/1-1-1-Scrambled-EDU-H1299_Series013_z0_ch01.jpg]

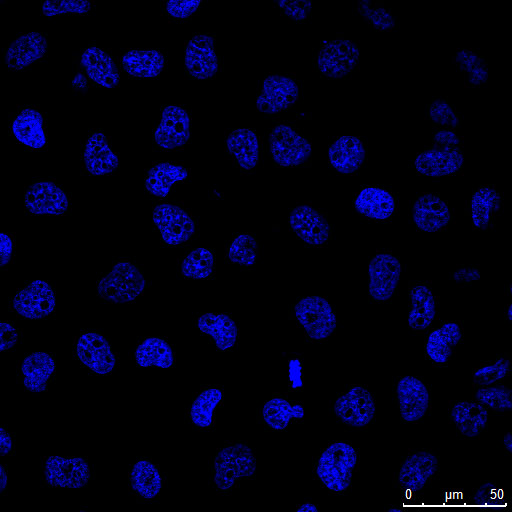

Supplement: Supplementary file 8 [file DataSheet_5.zip › Data Sheet 5/FigS1D/1-1-1-SiAC009948.5-EDU-H1299_Series034_z0_ch01.jpg]

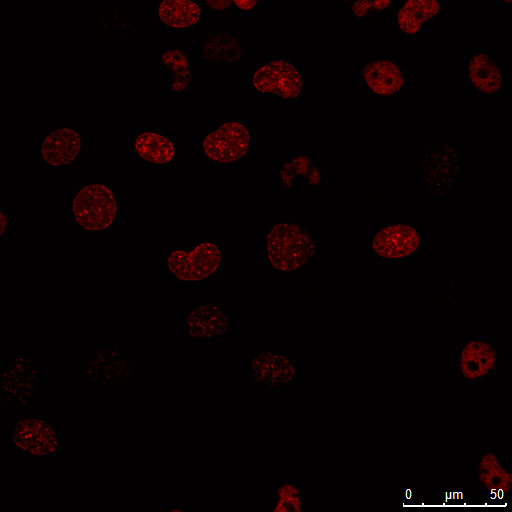

Supplement: Supplementary file 8 [file DataSheet_5.zip › Data Sheet 5/FigS1D/1-1-NC-EDU-H1299_Series007_z0_ch00.jpg]

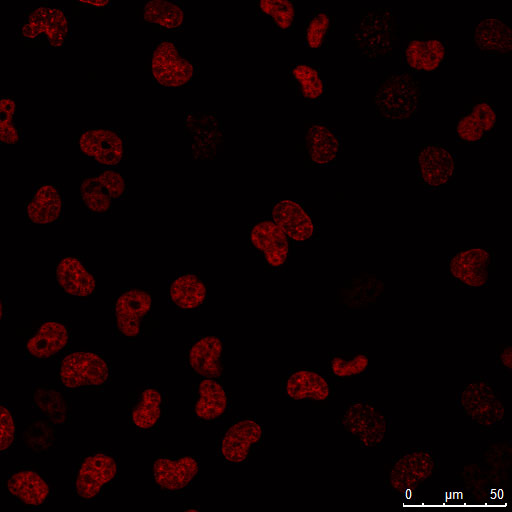

Supplement: Supplementary file 8 [file DataSheet_5.zip › Data Sheet 5/FigS1D/1-1-over-AC009948.5-EDU-H1299_Series051_z0_ch00.jpg]

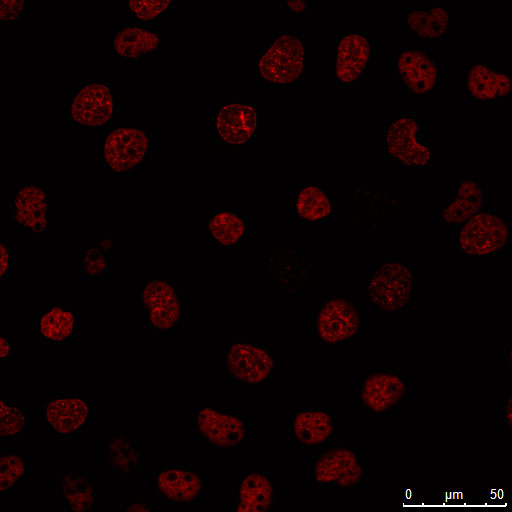

Supplement: Supplementary file 8 [file DataSheet_5.zip › Data Sheet 5/FigS1D/1-1-Scrambled-EDU-H1299_Series013_z0_ch00.jpg]

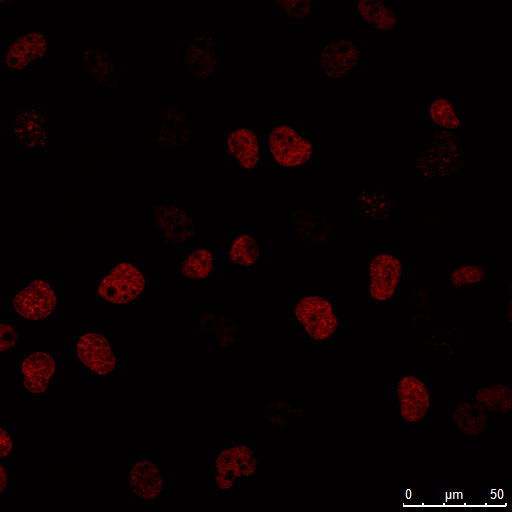

Supplement: Supplementary file 8 [file DataSheet_5.zip › Data Sheet 5/FigS1D/1-1-SiAC009948.5-EDU-H1299_Series034_z0_ch00.jpg]

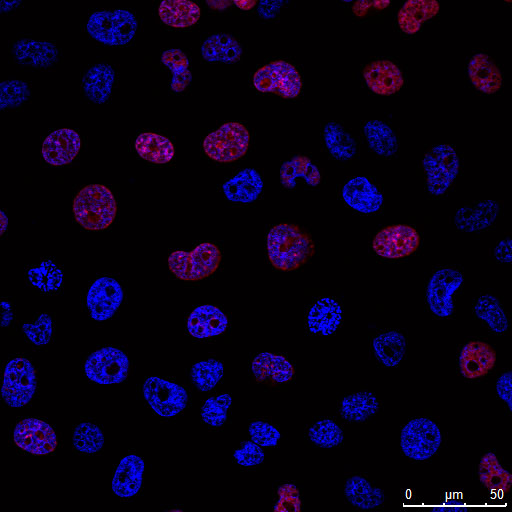

Supplement: Supplementary file 8 [file DataSheet_5.zip › Data Sheet 5/FigS1D/1-NC-EDU-H1299_Series007_z0.jpg]

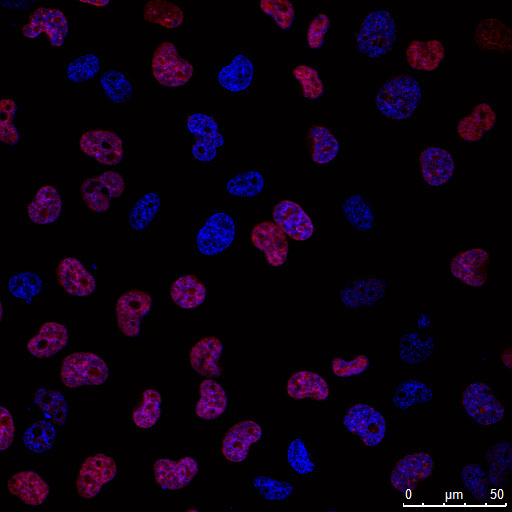

Supplement: Supplementary file 8 [file DataSheet_5.zip › Data Sheet 5/FigS1D/1-overAC009948.5-EDU-H1299_Series051_z0.jpg]

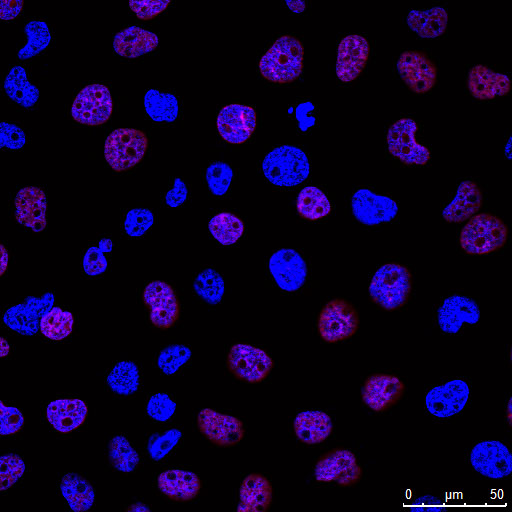

Supplement: Supplementary file 8 [file DataSheet_5.zip › Data Sheet 5/FigS1D/1-Scrambled-EDU-H1299_Series013_z0.jpg]

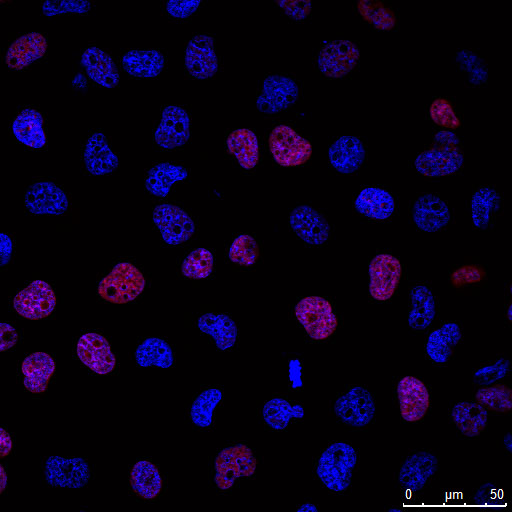

Supplement: Supplementary file 8 [file DataSheet_5.zip › Data Sheet 5/FigS1D/1-SiAC009948.5-EDU-H1299_Series034_z0.jpg]

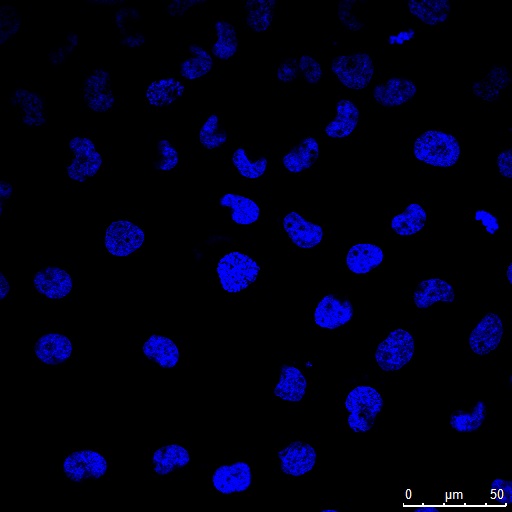

Supplement: Supplementary file 8 [file DataSheet_5.zip › Data Sheet 5/FigS1D/2-1-1-NC-EDU-H1299_Series018_z0_ch01.jpg]

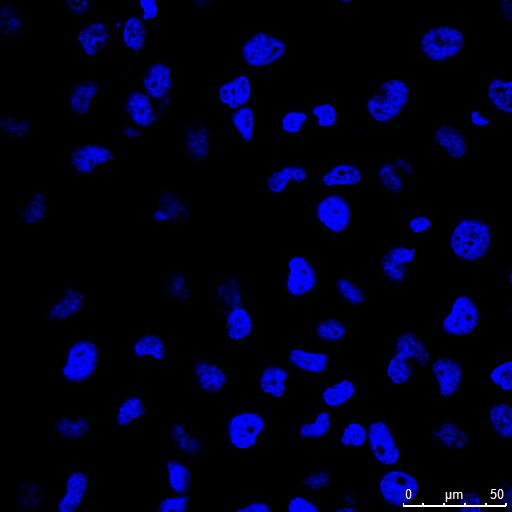

Supplement: Supplementary file 8 [file DataSheet_5.zip › Data Sheet 5/FigS1D/2-1-1-over-AC009948.5-EDU-H1299_Series024_z0_ch01.jpg]

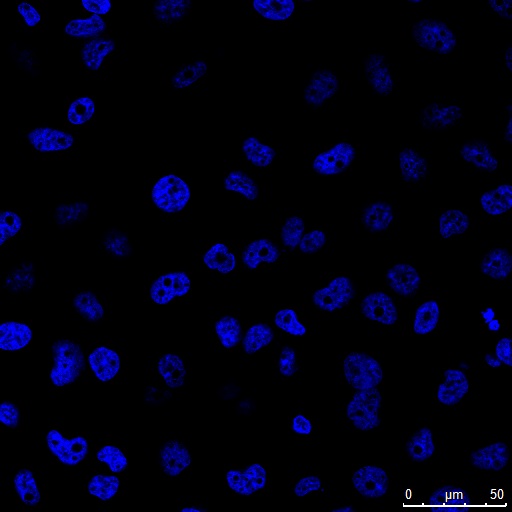

Supplement: Supplementary file 8 [file DataSheet_5.zip › Data Sheet 5/FigS1D/2-1-1-Scrambled-EDU-H1299_Series045_z0_ch01.jpg]

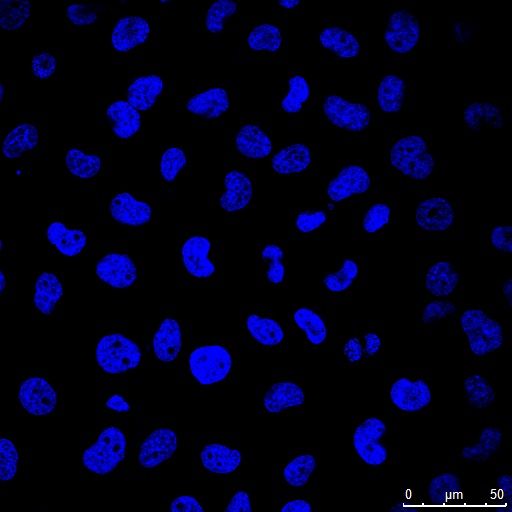

Supplement: Supplementary file 8 [file DataSheet_5.zip › Data Sheet 5/FigS1D/2-1-1-SiAC009948.5-EDU-H1299_Series028_z0_ch01.jpg]

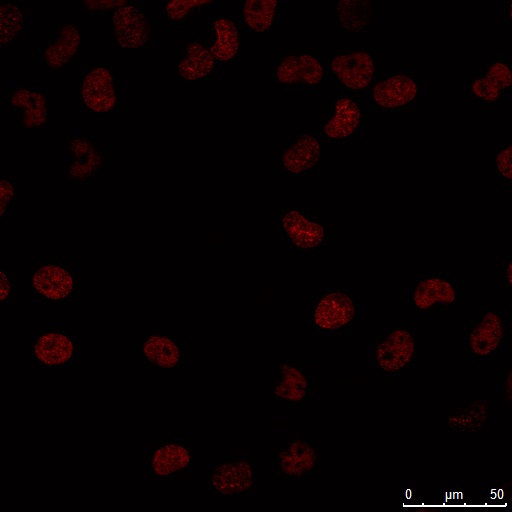

Supplement: Supplementary file 8 [file DataSheet_5.zip › Data Sheet 5/FigS1D/2-1-NC-EDU-H1299_Series018_z0_ch00.jpg]

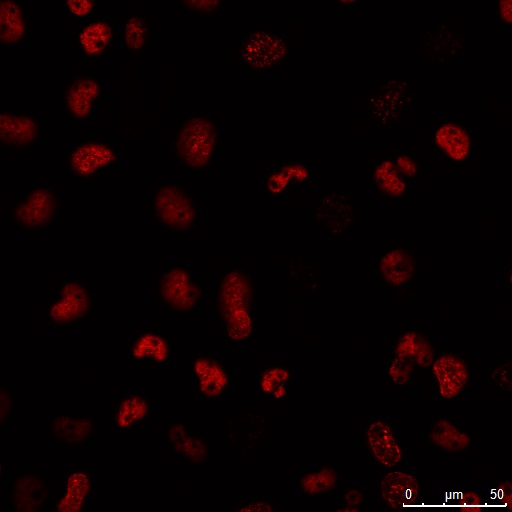

Supplement: Supplementary file 8 [file DataSheet_5.zip › Data Sheet 5/FigS1D/2-1-over-AC009948.5-EDU-H1299_Series024_z0_ch00.jpg]

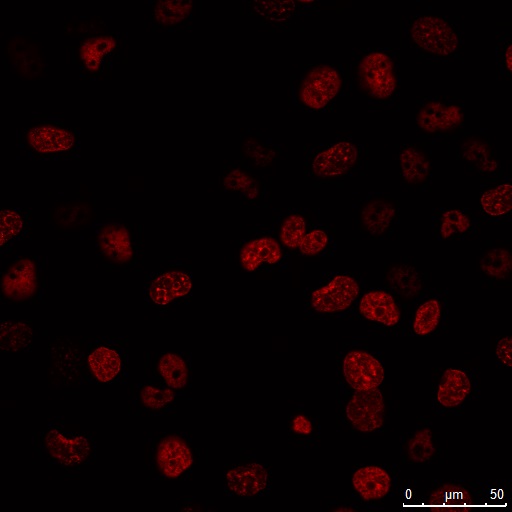

Supplement: Supplementary file 8 [file DataSheet_5.zip › Data Sheet 5/FigS1D/2-1-Scrambled-EDU-H1299_Series045_z0_ch00.jpg]

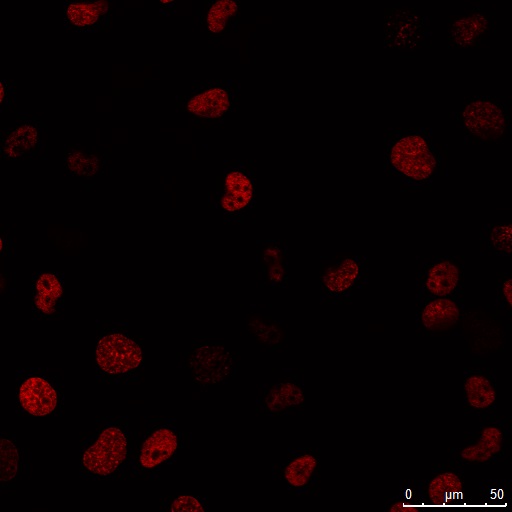

Supplement: Supplementary file 8 [file DataSheet_5.zip › Data Sheet 5/FigS1D/2-1-SiAC009948.5-EDU-H1299_Series028_z0_ch00.jpg]

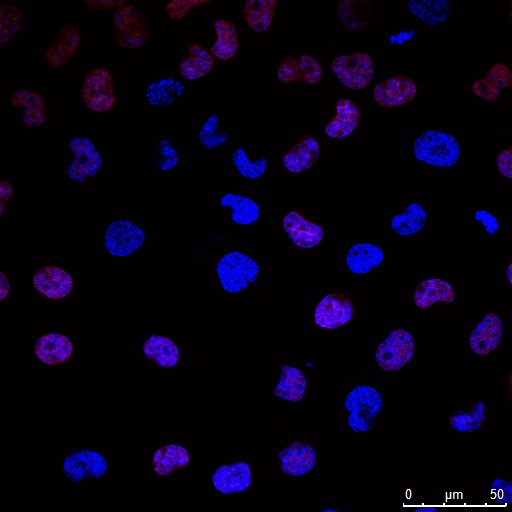

Supplement: Supplementary file 8 [file DataSheet_5.zip › Data Sheet 5/FigS1D/2-NC-EDU-H1299_Series018_z0.jpg]

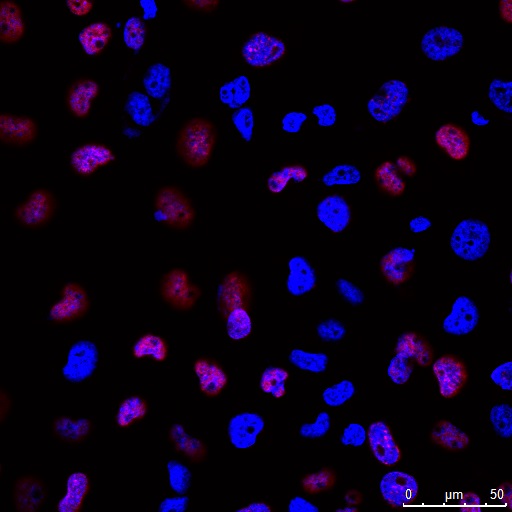

Supplement: Supplementary file 8 [file DataSheet_5.zip › Data Sheet 5/FigS1D/2-over-AC009948.5-EDU-H1299_Series024_z0.jpg]

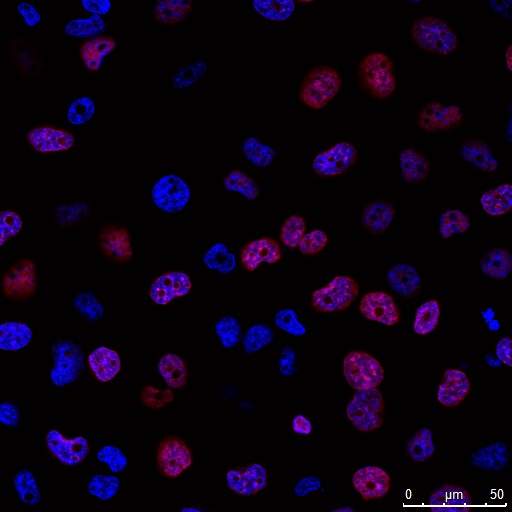

Supplement: Supplementary file 8 [file DataSheet_5.zip › Data Sheet 5/FigS1D/2-Scrambled-EDU-H1299_Series045_z0.jpg]

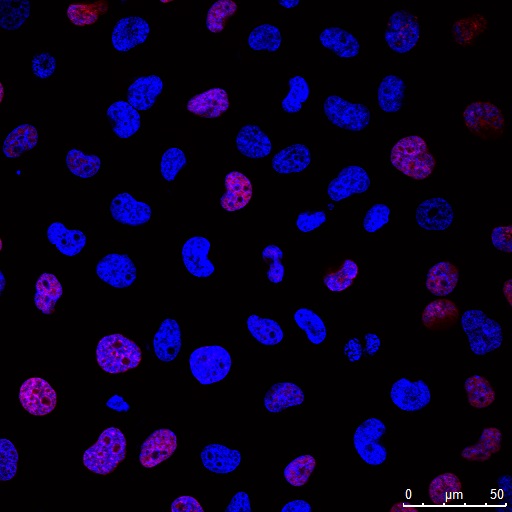

Supplement: Supplementary file 8 [file DataSheet_5.zip › Data Sheet 5/FigS1D/2-SiAC009948.5-EDU-H1299_Series028_z0.jpg]

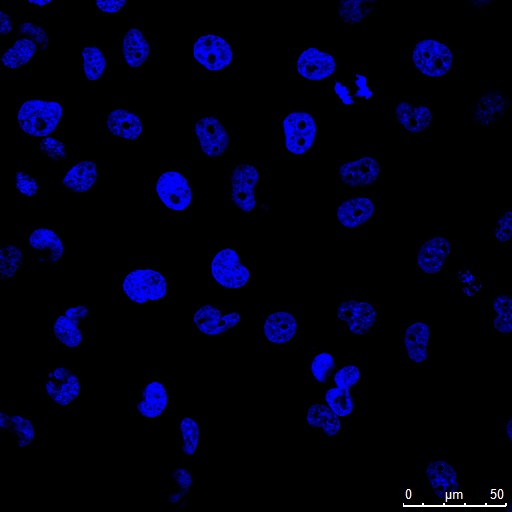

Supplement: Supplementary file 8 [file DataSheet_5.zip › Data Sheet 5/FigS1D/3-1-1-NC-EDU-H1299_Series063_z0_ch00.jpg]

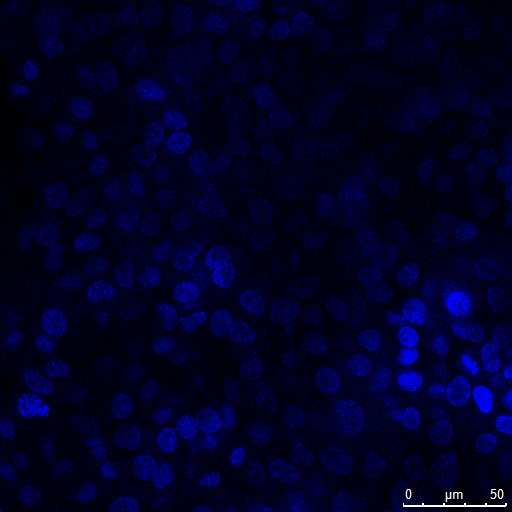

Supplement: Supplementary file 8 [file DataSheet_5.zip › Data Sheet 5/FigS1D/3-1-1-over-AC009948.5-edu-H1299-CON313_Series074_z0_ch00.jpg]

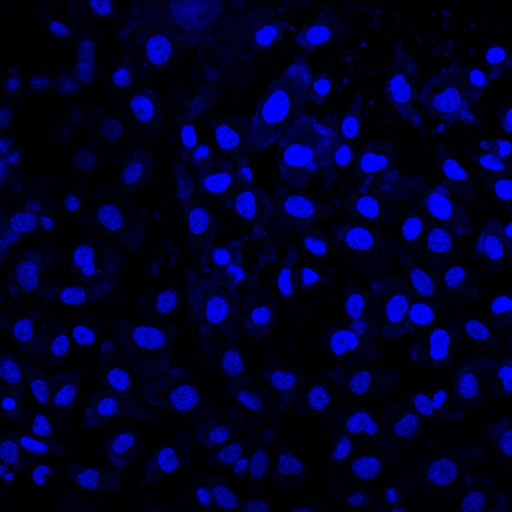

Supplement: Supplementary file 8 [file DataSheet_5.zip › Data Sheet 5/FigS1D/3-1-1-Scrambled-edu-H1299-con_Series064_z0_ch01.jpg]

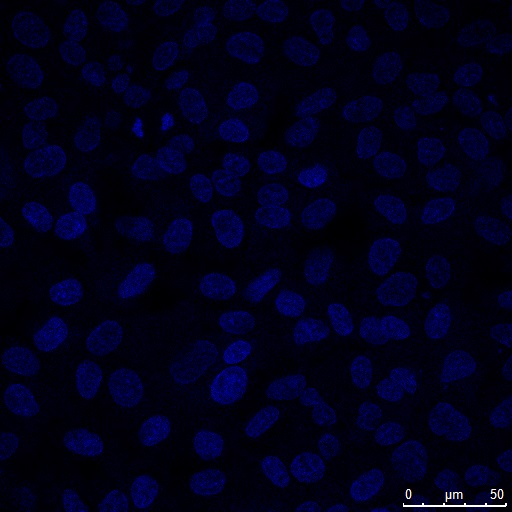

Supplement: Supplementary file 8 [file DataSheet_5.zip › Data Sheet 5/FigS1D/3-1-1SiAC009948.5-edu-H1299-CON313_Series081_z0_ch00.jpg]

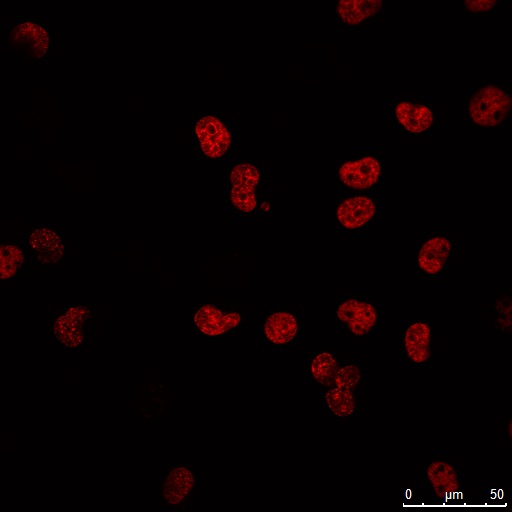

Supplement: Supplementary file 8 [file DataSheet_5.zip › Data Sheet 5/FigS1D/3-1-NC-EDU-H1299_Series063_z0_ch00.jpg]

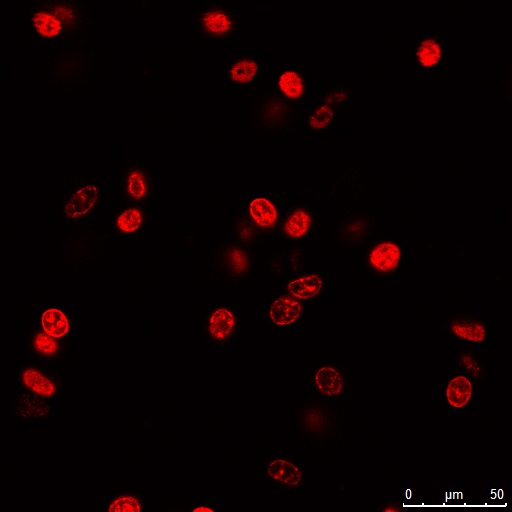

Supplement: Supplementary file 8 [file DataSheet_5.zip › Data Sheet 5/FigS1D/3-1-over-AC009948.5-edu-H1299-CON313_Series074_z0_ch00.jpg]

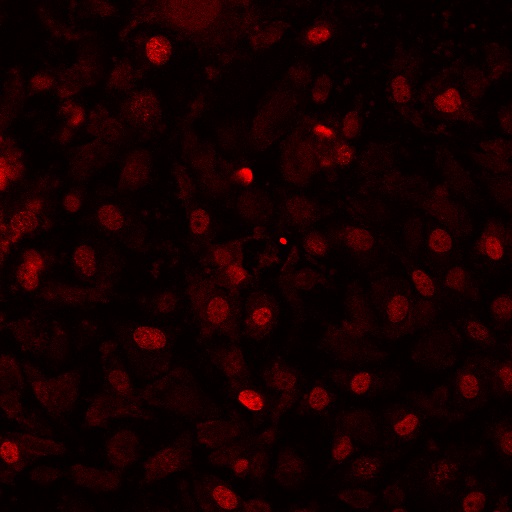

Supplement: Supplementary file 8 [file DataSheet_5.zip › Data Sheet 5/FigS1D/3-1-Scrambled-edu-H1299-con_Series064_z0_ch01.jpg]

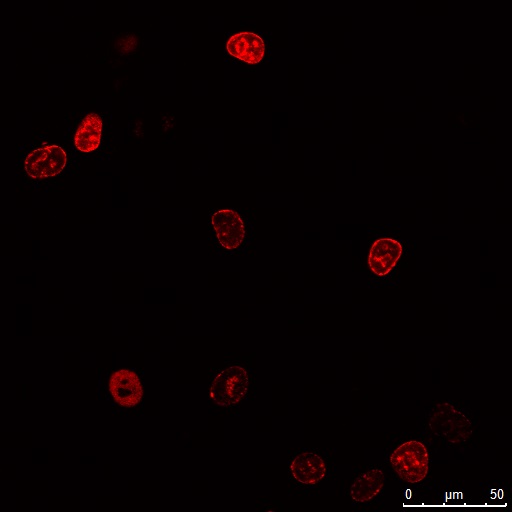

Supplement: Supplementary file 8 [file DataSheet_5.zip › Data Sheet 5/FigS1D/3-1-SiAC009948.5-edu-H1299-CON313_Series081_z0_ch00.jpg]

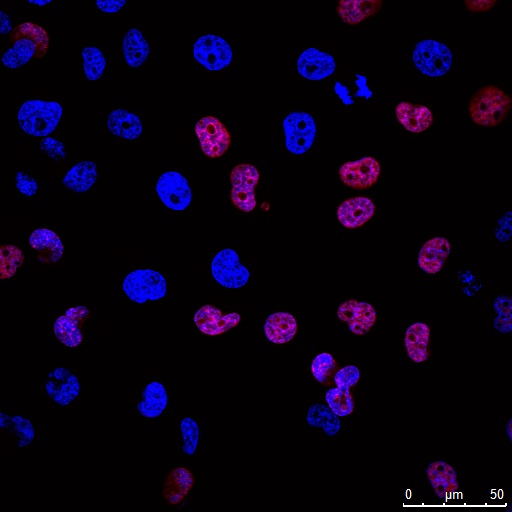

Supplement: Supplementary file 8 [file DataSheet_5.zip › Data Sheet 5/FigS1D/3-NC-EDU-H1299_Series063_z0_ch00.jpg]

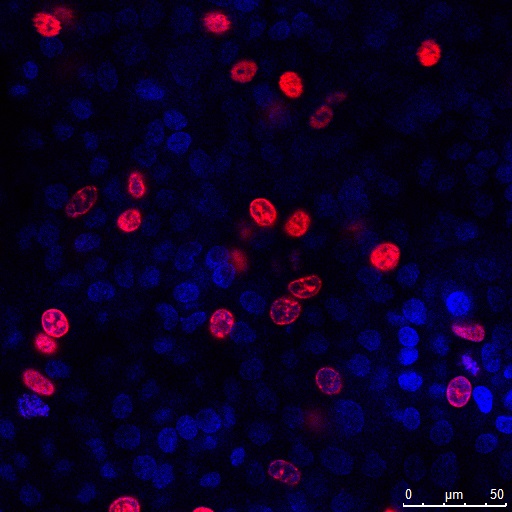

Supplement: Supplementary file 8 [file DataSheet_5.zip › Data Sheet 5/FigS1D/3-over-AC009948.5-edu-H1299-CON313_Series074_z0_ch00.jpg]

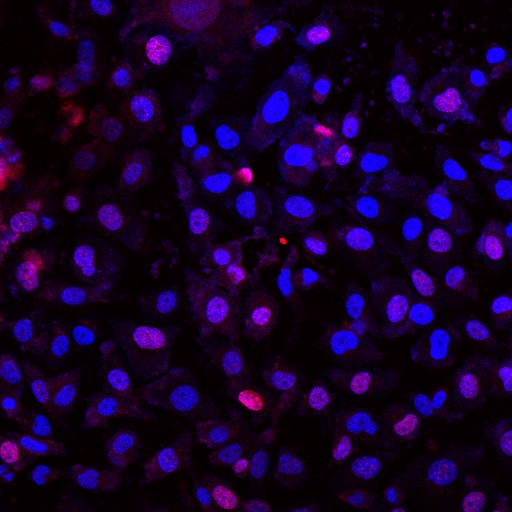

Supplement: Supplementary file 8 [file DataSheet_5.zip › Data Sheet 5/FigS1D/3-Scrambled-edu-H1299-con_Series064_z0_ch01.jpg]

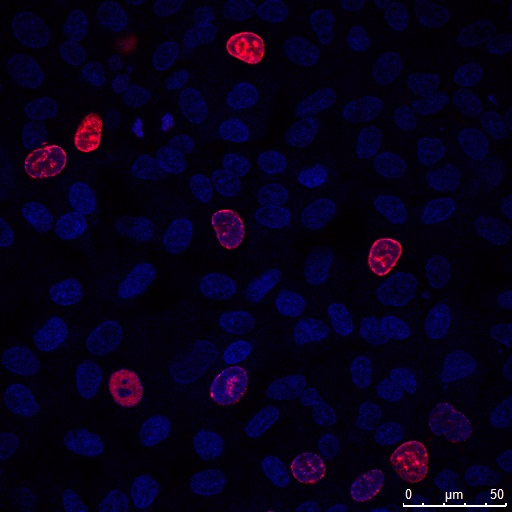

Supplement: Supplementary file 8 [file DataSheet_5.zip › Data Sheet 5/FigS1D/3-SiAC009948.5-edu-H1299-CON313_Series081_z0_ch00.jpg]

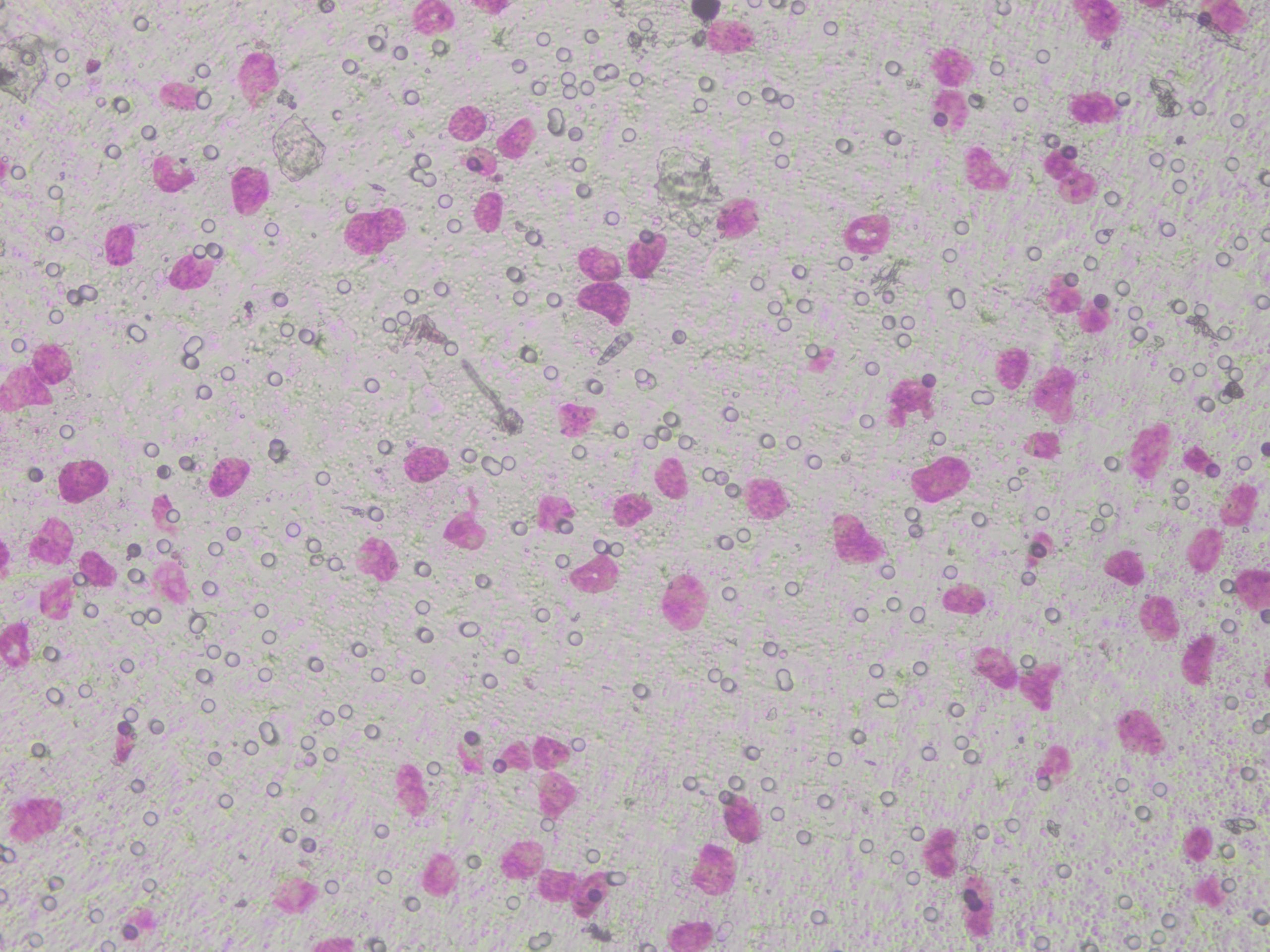

Supplement: Supplementary file 9 [file DataSheet_6.zip › Data Sheet 6/FigS1E/1-NC-INVASION.jpg]

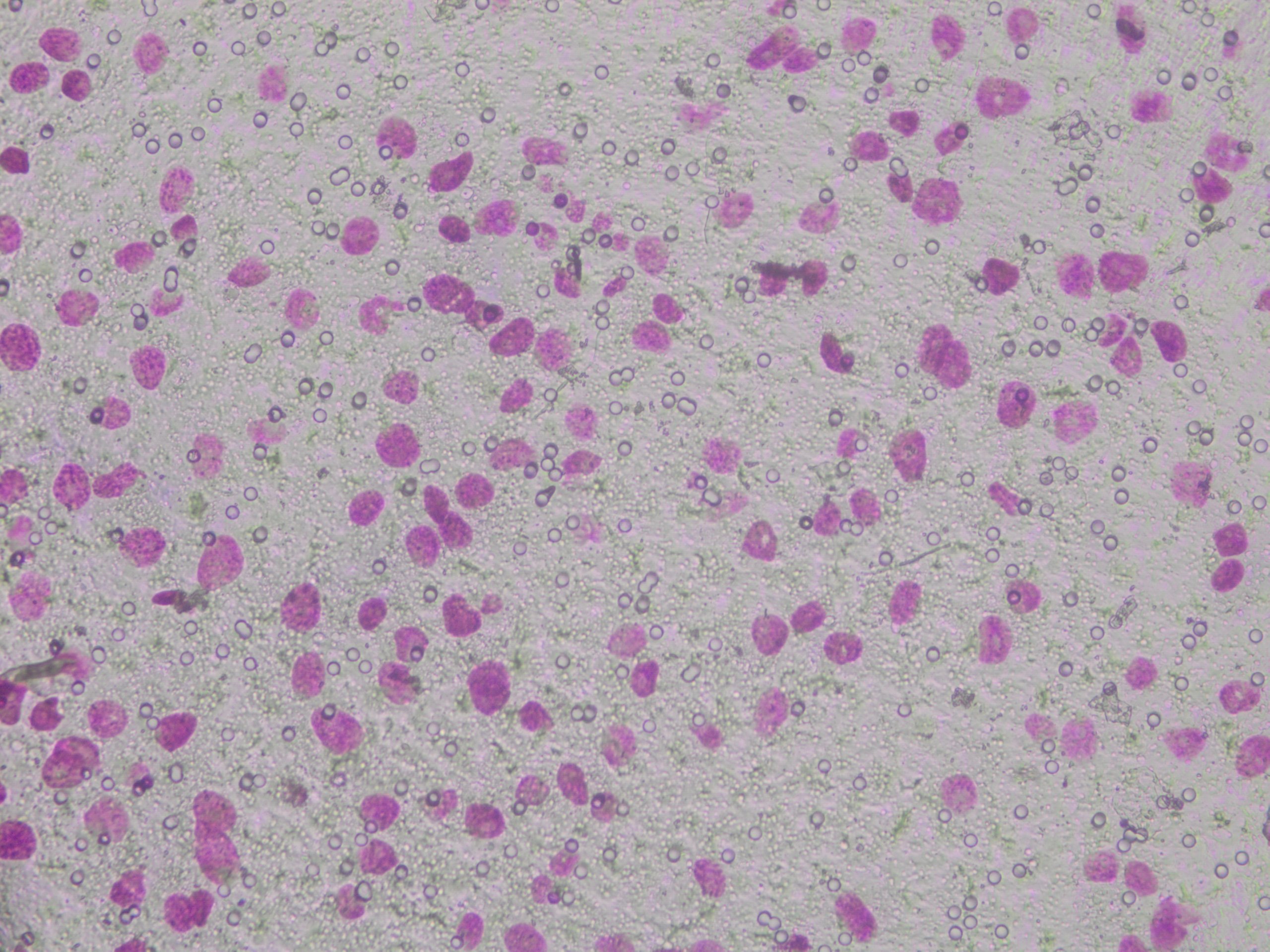

Supplement: Supplementary file 9 [file DataSheet_6.zip › Data Sheet 6/FigS1E/1-NC-M.jpg]

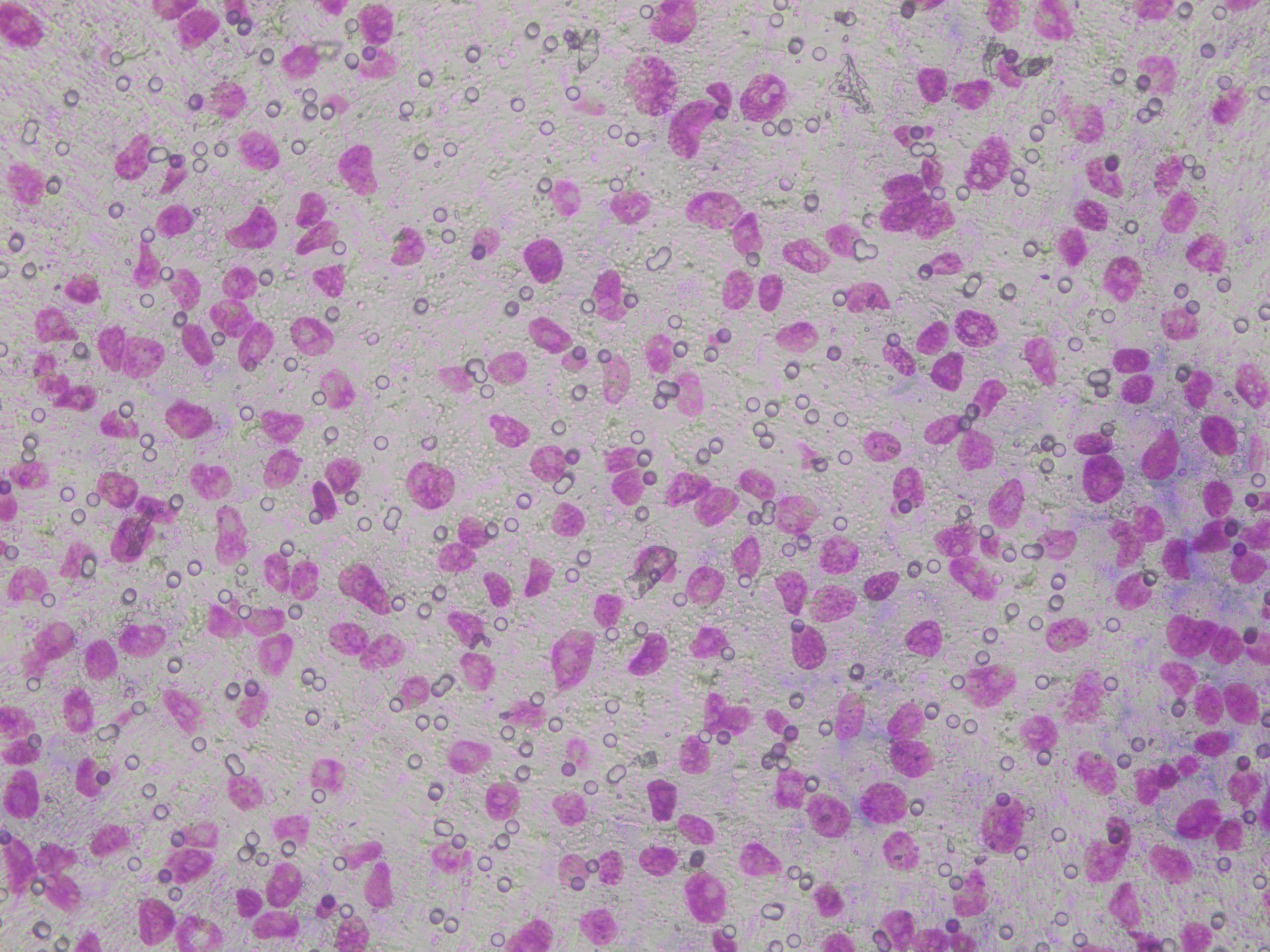

Supplement: Supplementary file 9 [file DataSheet_6.zip › Data Sheet 6/FigS1E/1-over-AC009948.5-INVASION.jpg]

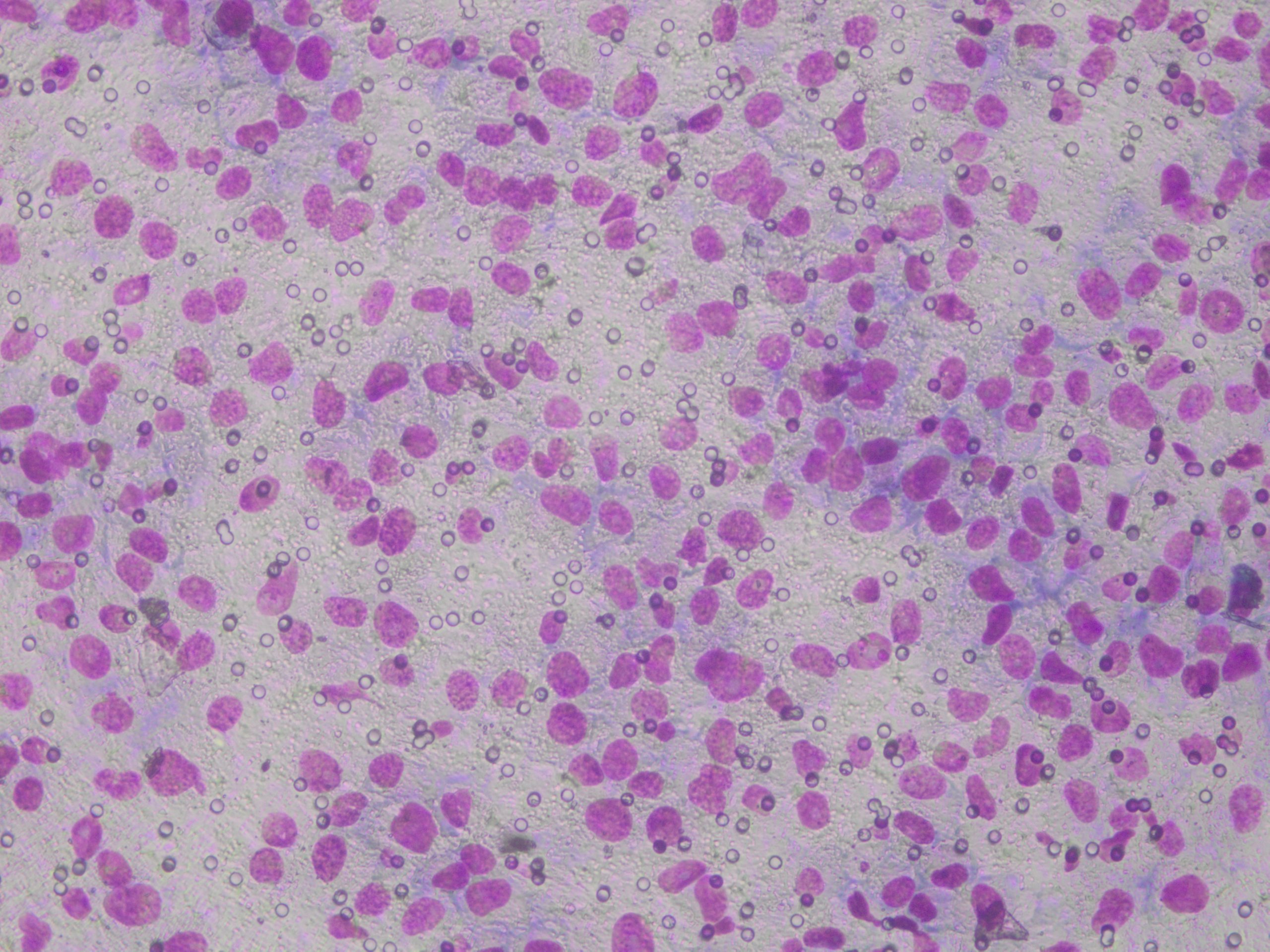

Supplement: Supplementary file 9 [file DataSheet_6.zip › Data Sheet 6/FigS1E/1-over-AC009948.5-M.jpg]

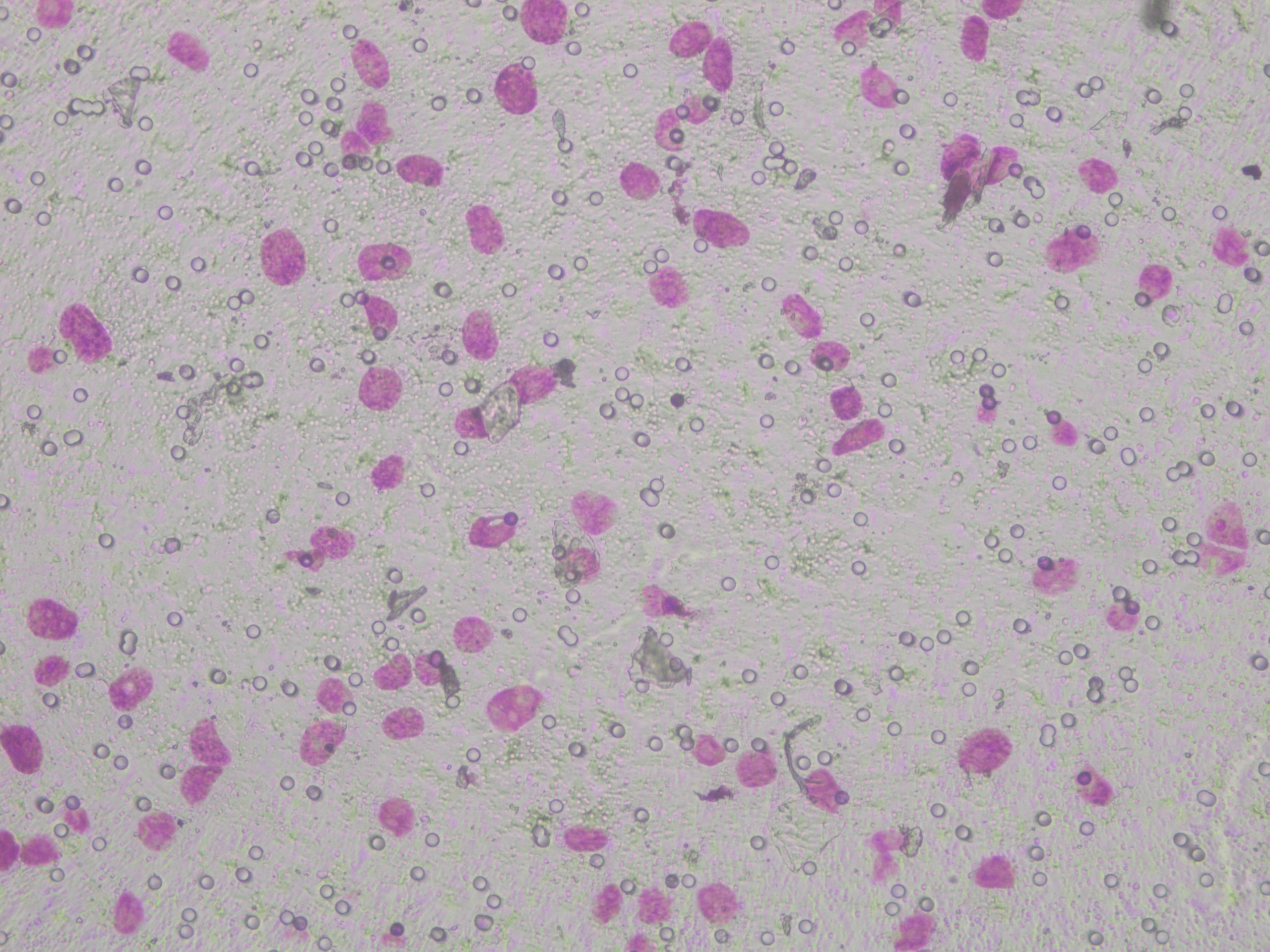

Supplement: Supplementary file 9 [file DataSheet_6.zip › Data Sheet 6/FigS1E/1-Scrambled-INVASION.jpg]

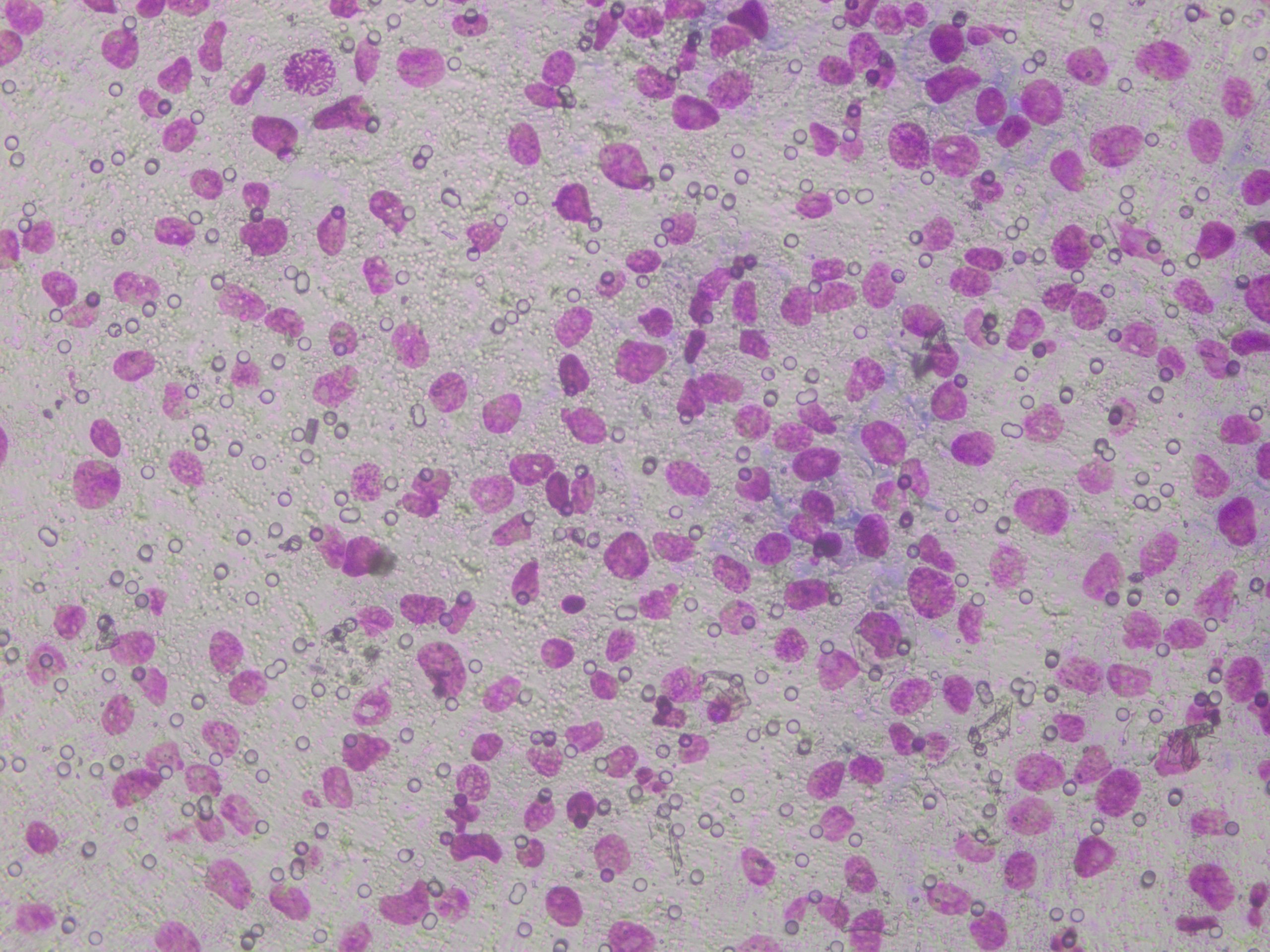

Supplement: Supplementary file 9 [file DataSheet_6.zip › Data Sheet 6/FigS1E/1-Scrambled-M.jpg]

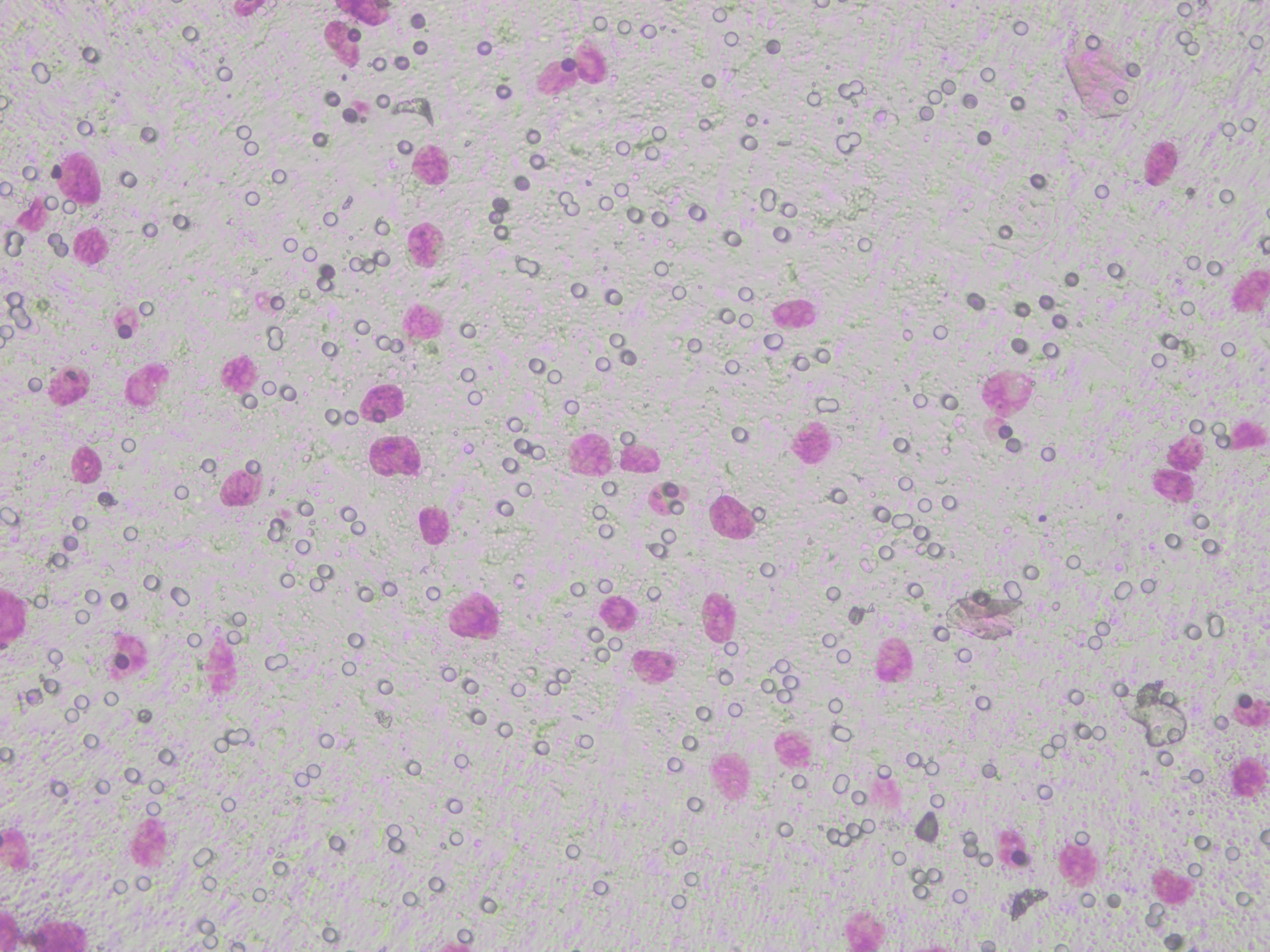

Supplement: Supplementary file 9 [file DataSheet_6.zip › Data Sheet 6/FigS1E/1-SiAC009948.5-INVASION.jpg]

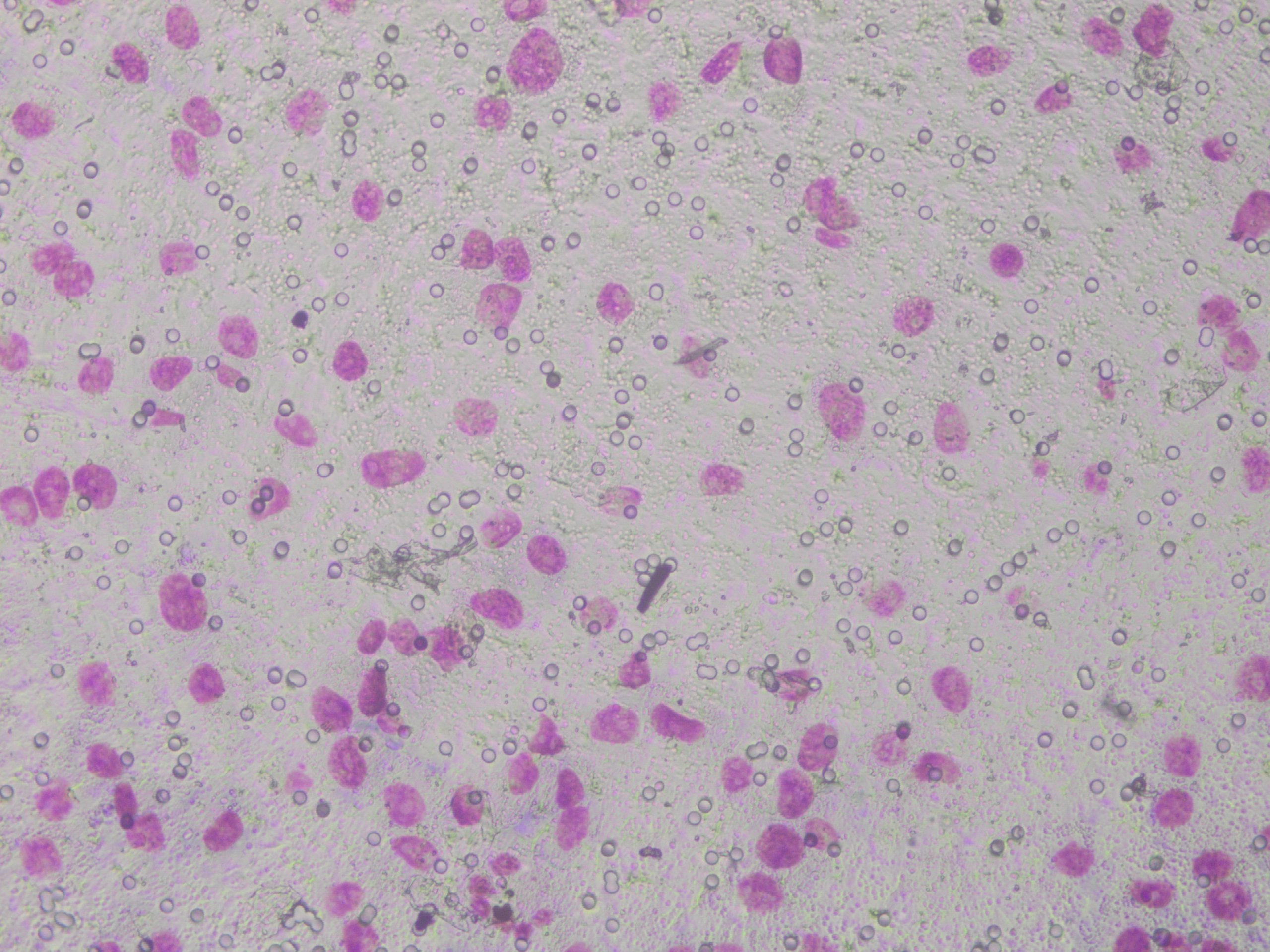

Supplement: Supplementary file 9 [file DataSheet_6.zip › Data Sheet 6/FigS1E/1-SiAC009948.5-M.jpg]

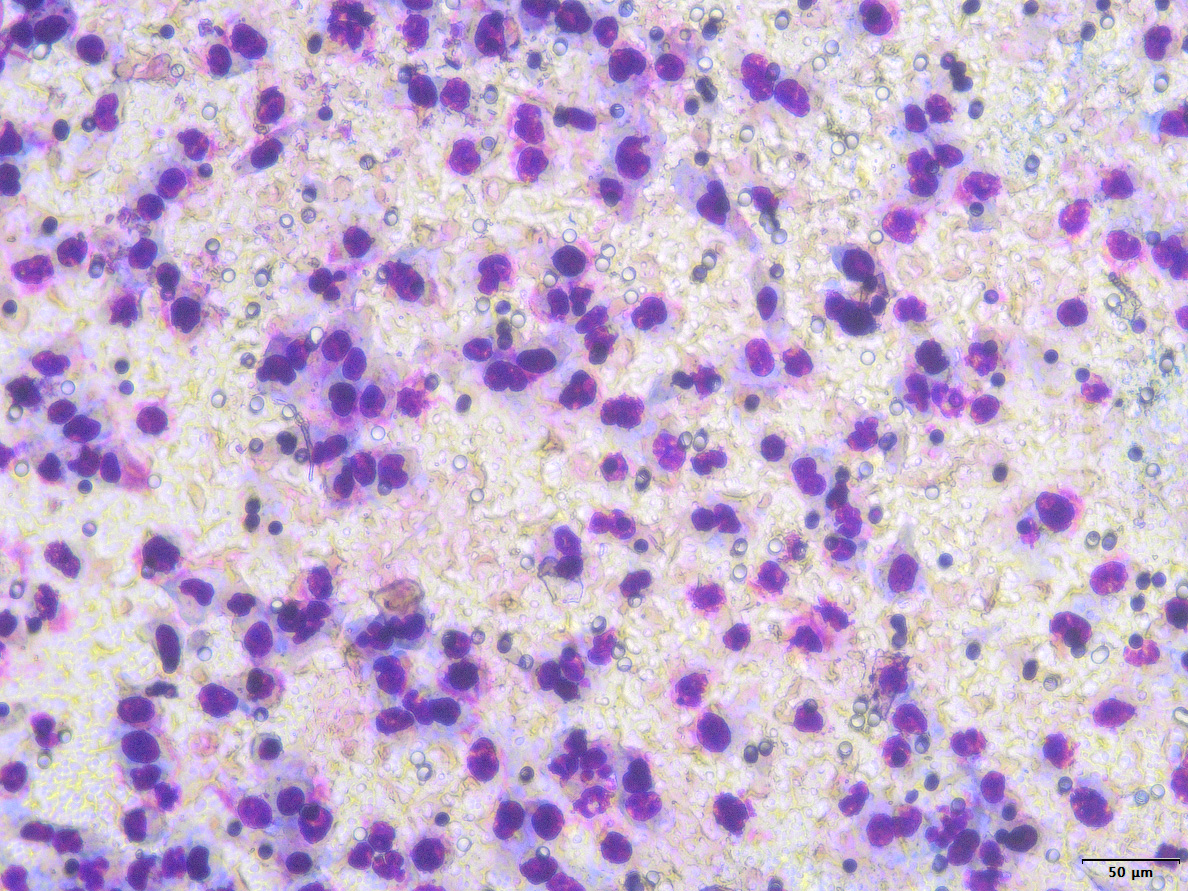

Supplement: Supplementary file 9 [file DataSheet_6.zip › Data Sheet 6/FigS1E/2-NC-INVASION.jpg]

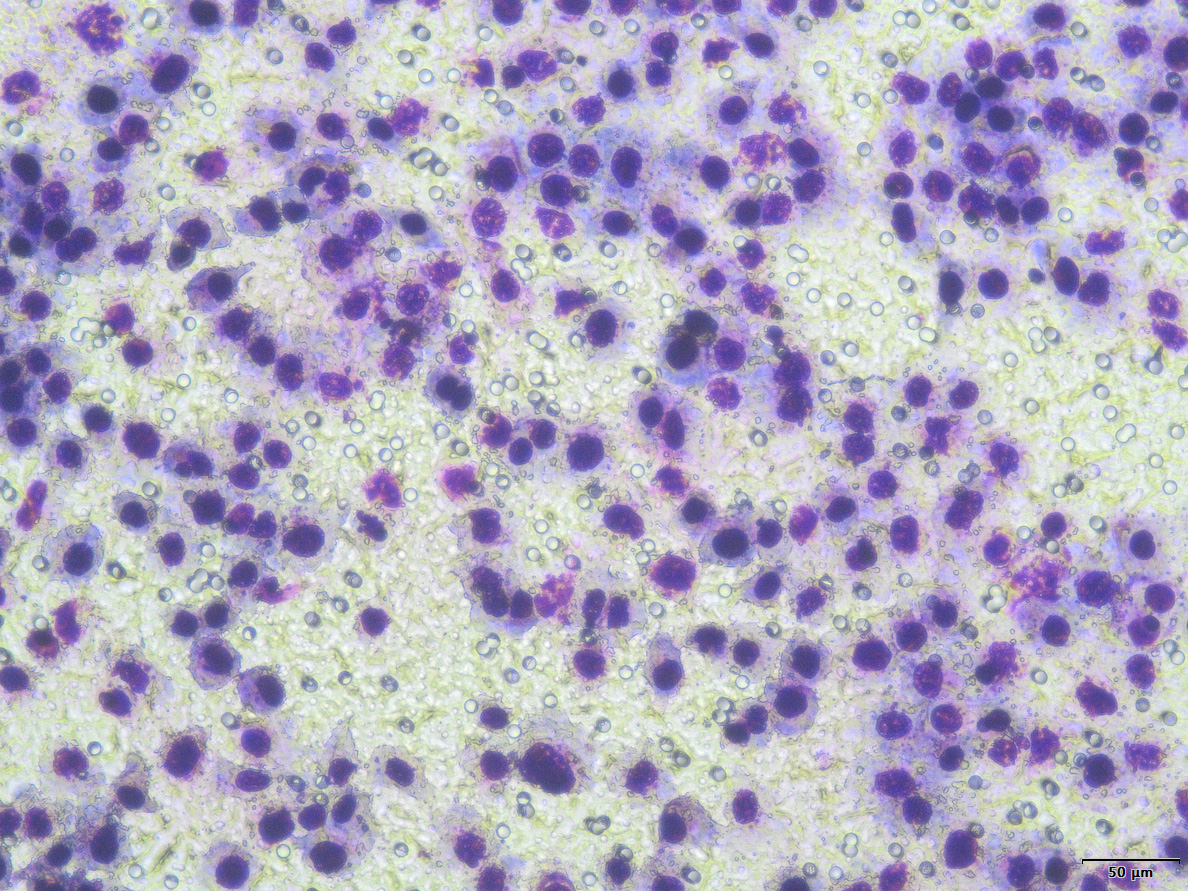

Supplement: Supplementary file 9 [file DataSheet_6.zip › Data Sheet 6/FigS1E/2-NC-M.jpg]

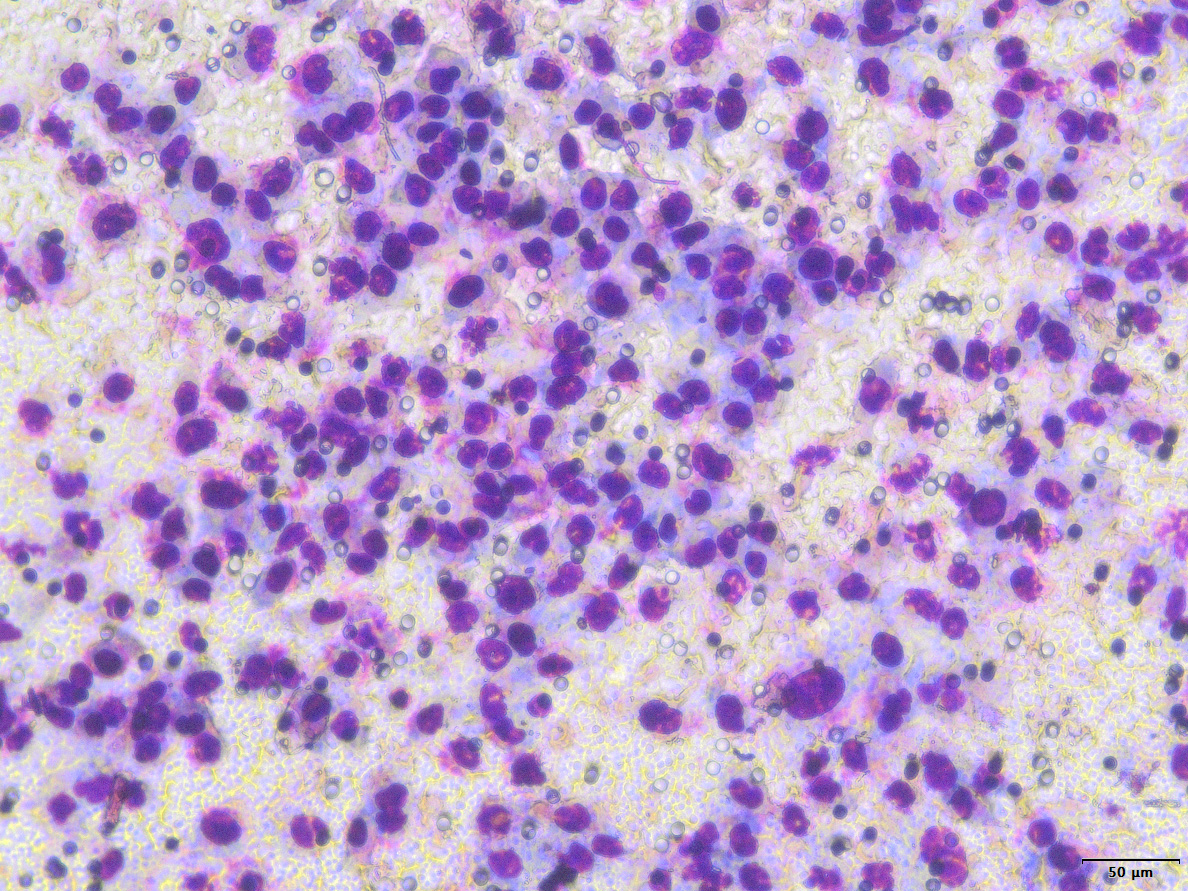

Supplement: Supplementary file 9 [file DataSheet_6.zip › Data Sheet 6/FigS1E/2-over-AC009948.5-INVASION.jpg]

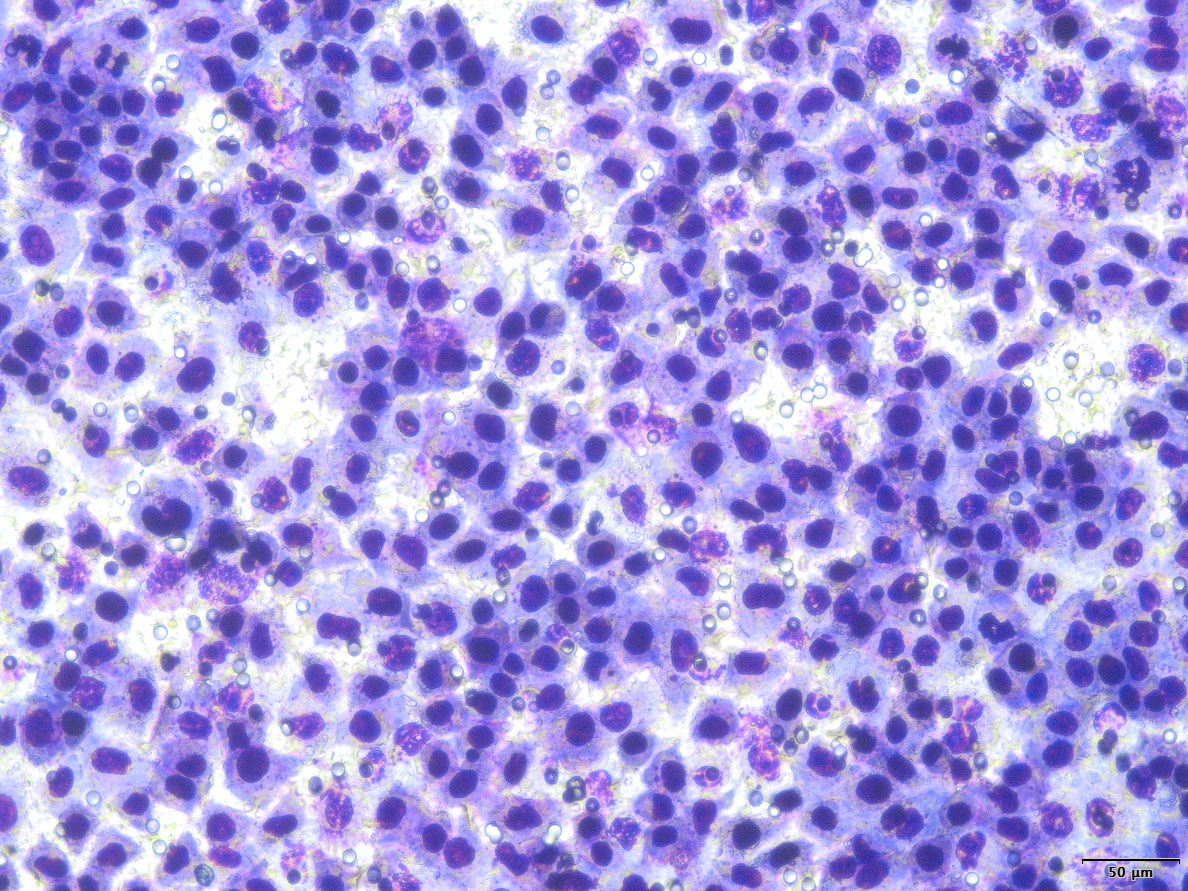

Supplement: Supplementary file 9 [file DataSheet_6.zip › Data Sheet 6/FigS1E/2-over-AC009948.5-M.jpg]

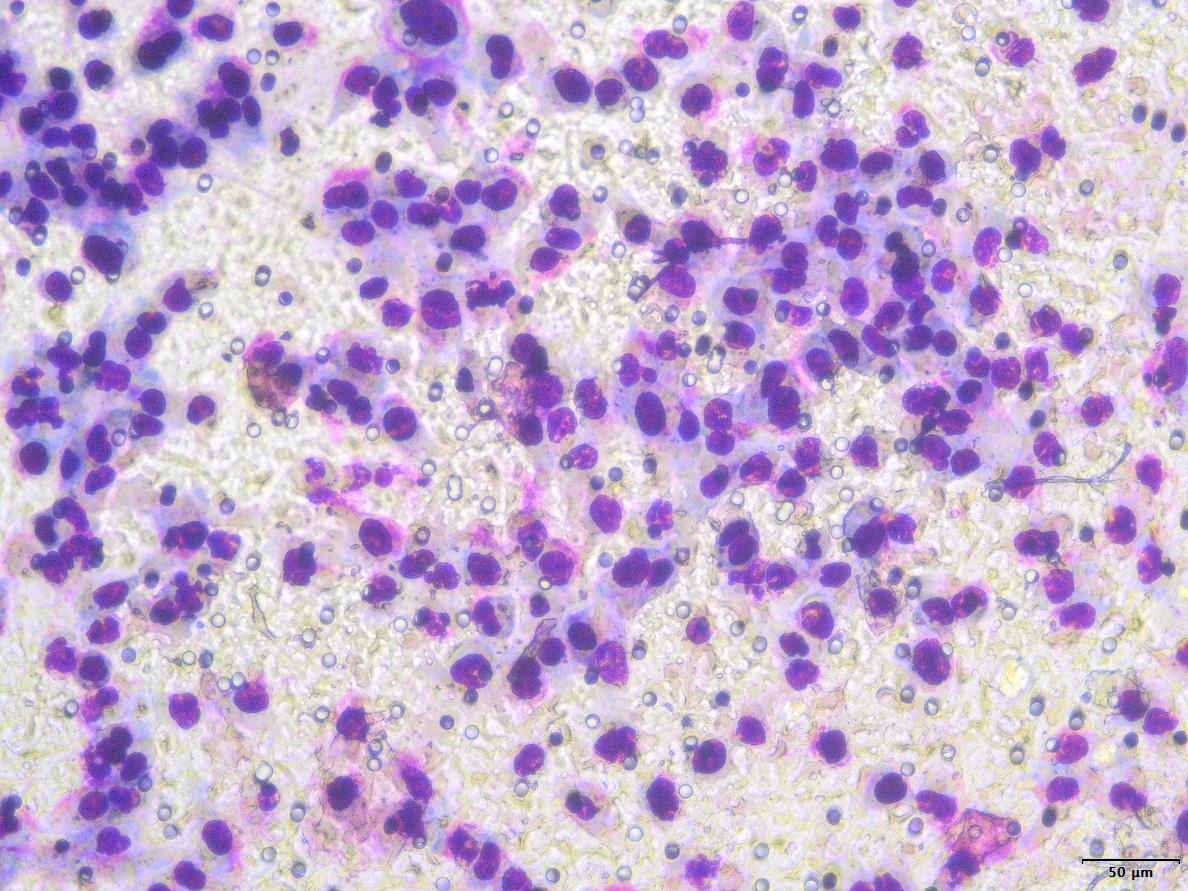

Supplement: Supplementary file 9 [file DataSheet_6.zip › Data Sheet 6/FigS1E/2-Scrambled-INVASION.jpg]

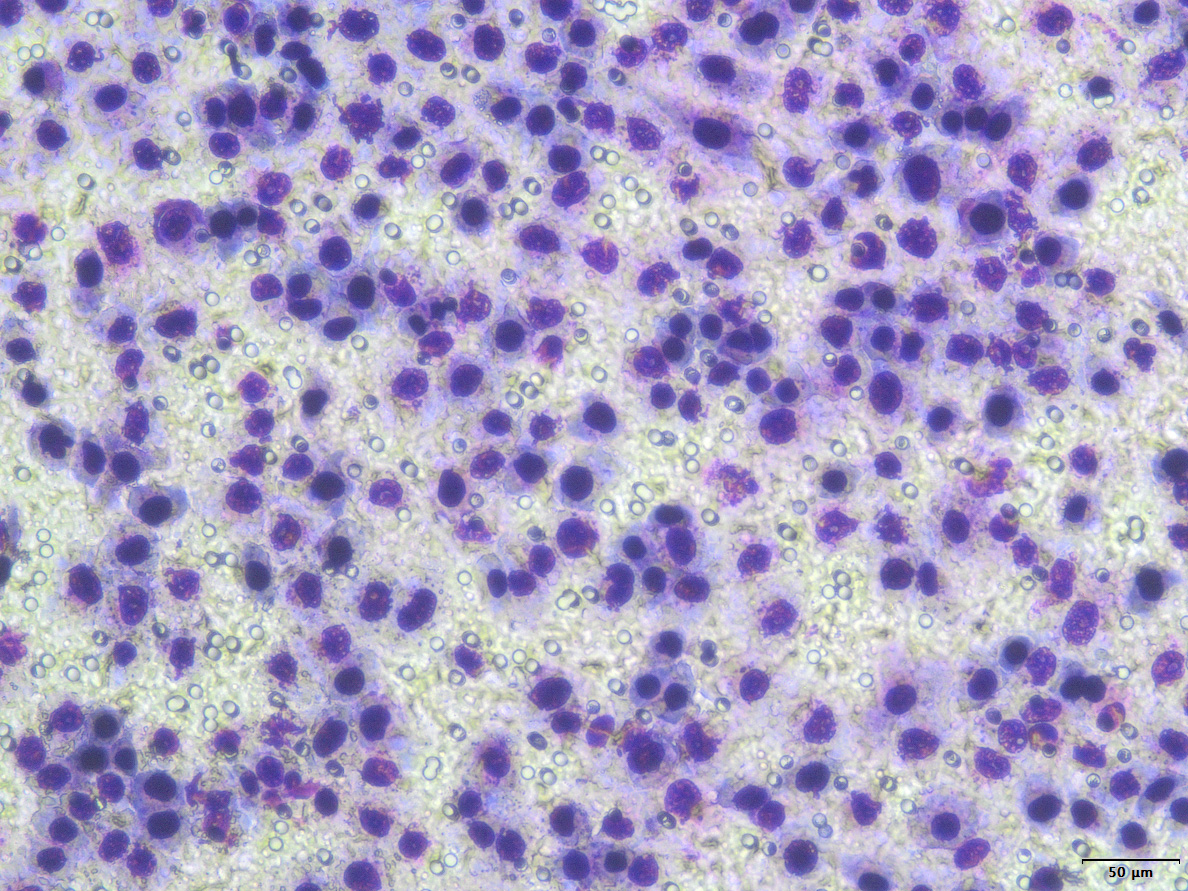

Supplement: Supplementary file 9 [file DataSheet_6.zip › Data Sheet 6/FigS1E/2-Scrambled-M.jpg]

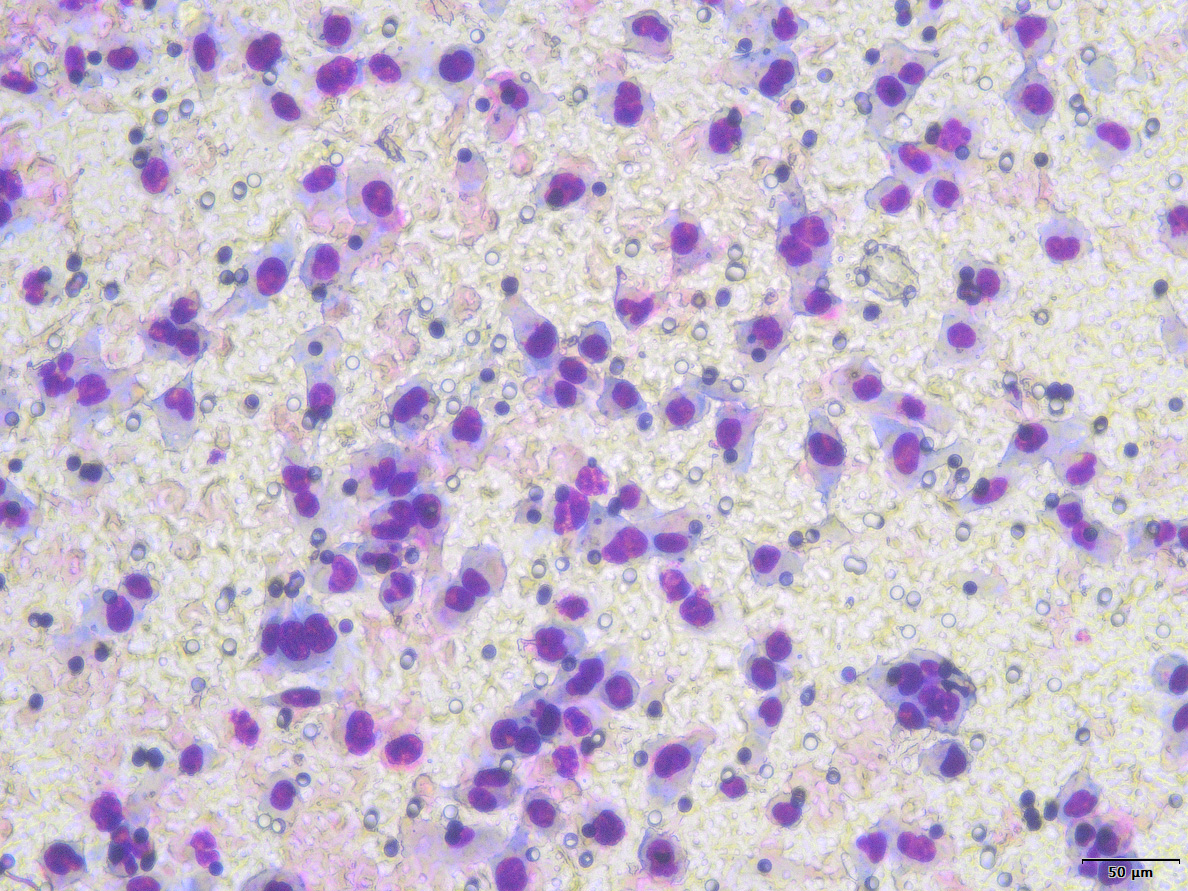

Supplement: Supplementary file 9 [file DataSheet_6.zip › Data Sheet 6/FigS1E/2-SIAC009948.5-INVASION.jpg]

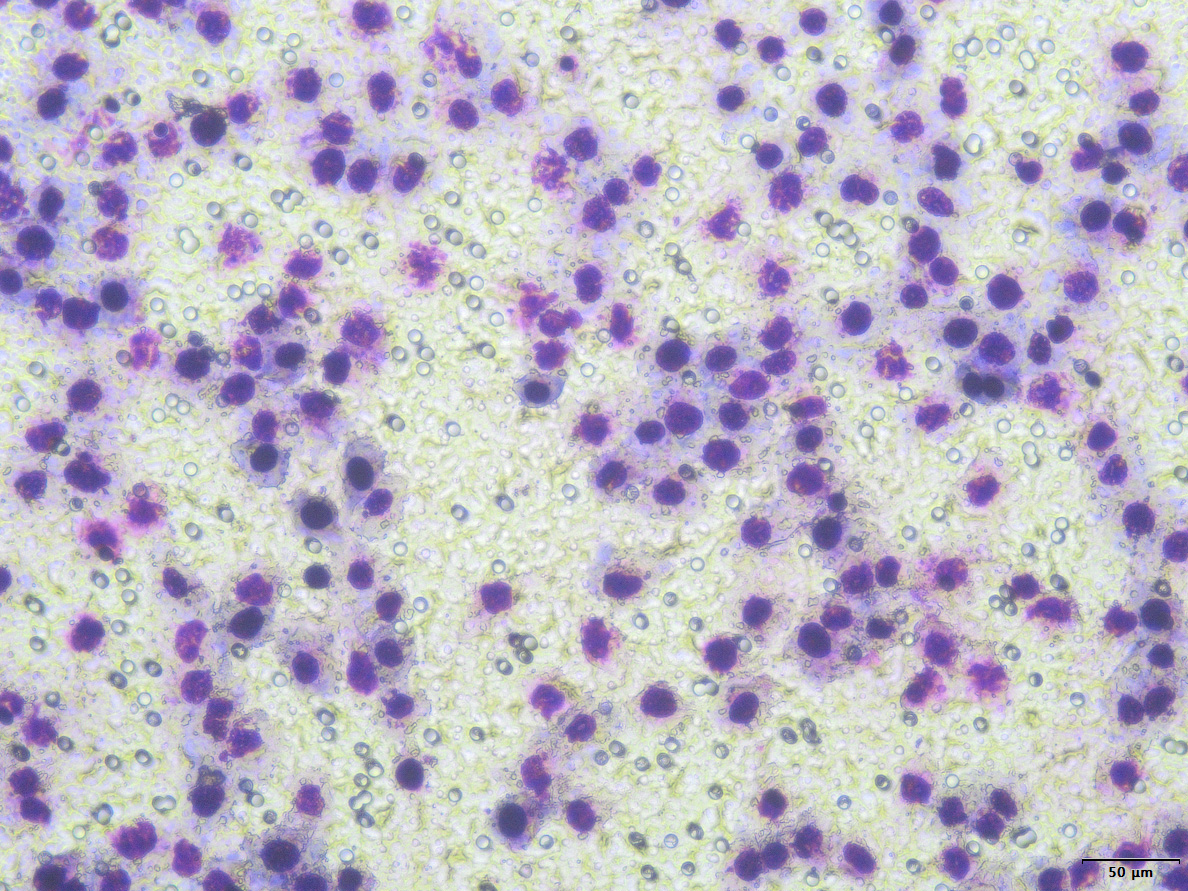

Supplement: Supplementary file 9 [file DataSheet_6.zip › Data Sheet 6/FigS1E/2-SIAC009948.5-M.jpg]

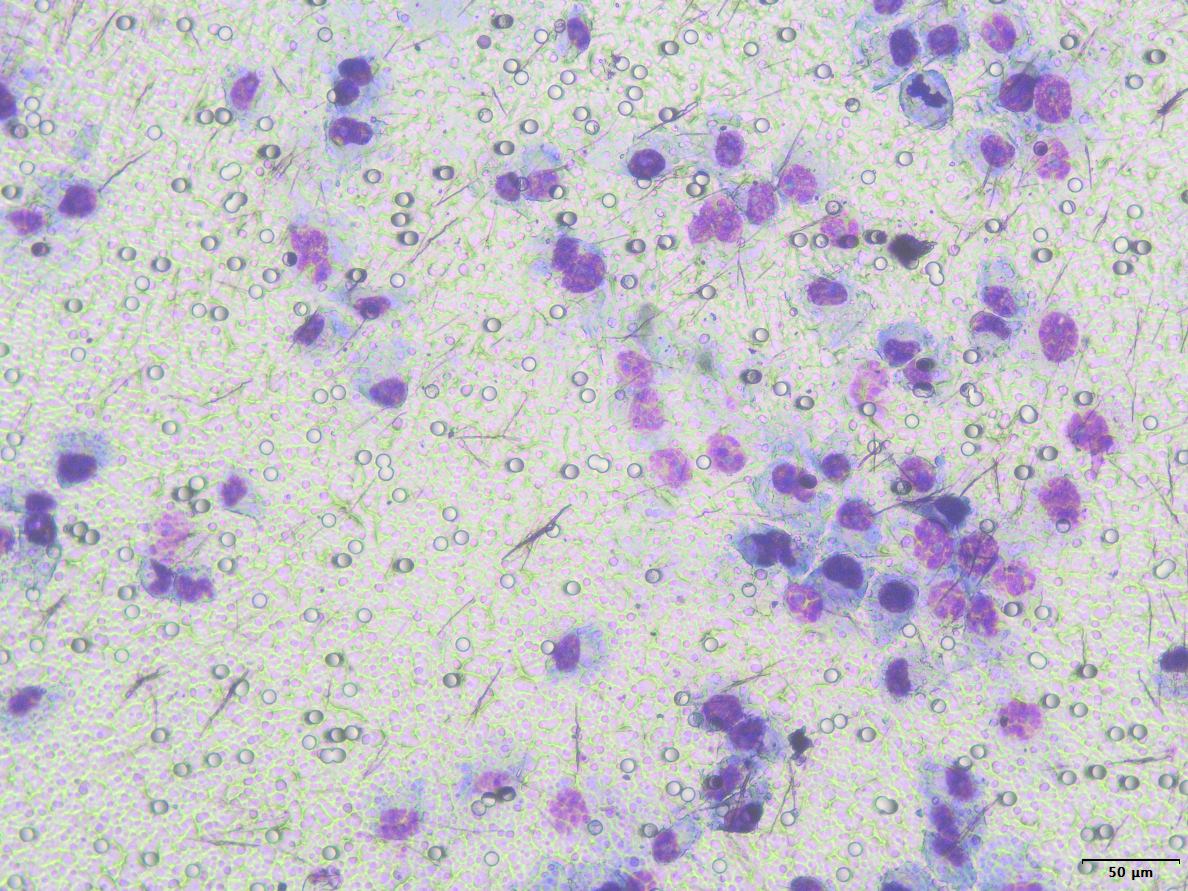

Supplement: Supplementary file 9 [file DataSheet_6.zip › Data Sheet 6/FigS1E/3-NC-INVASION.jpg]

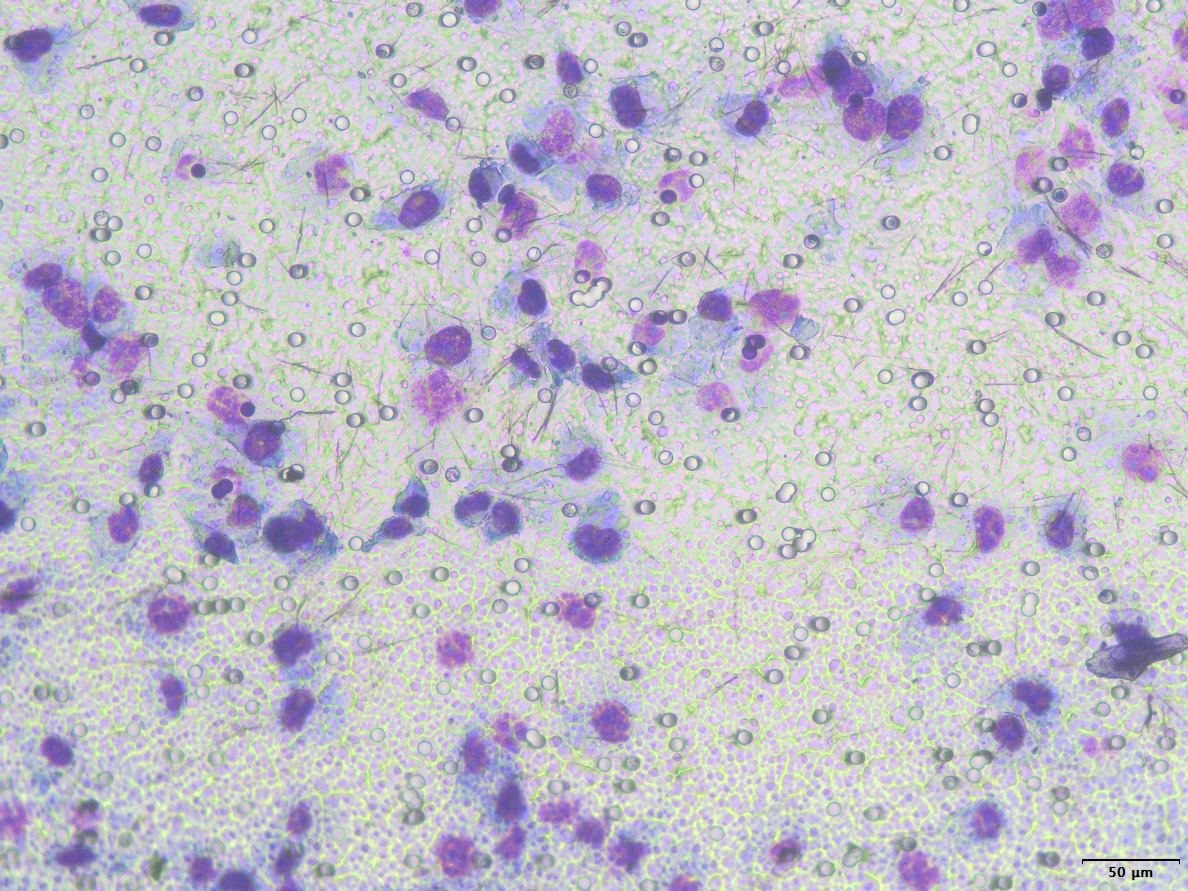

Supplement: Supplementary file 9 [file DataSheet_6.zip › Data Sheet 6/FigS1E/3-NC-M.jpg]

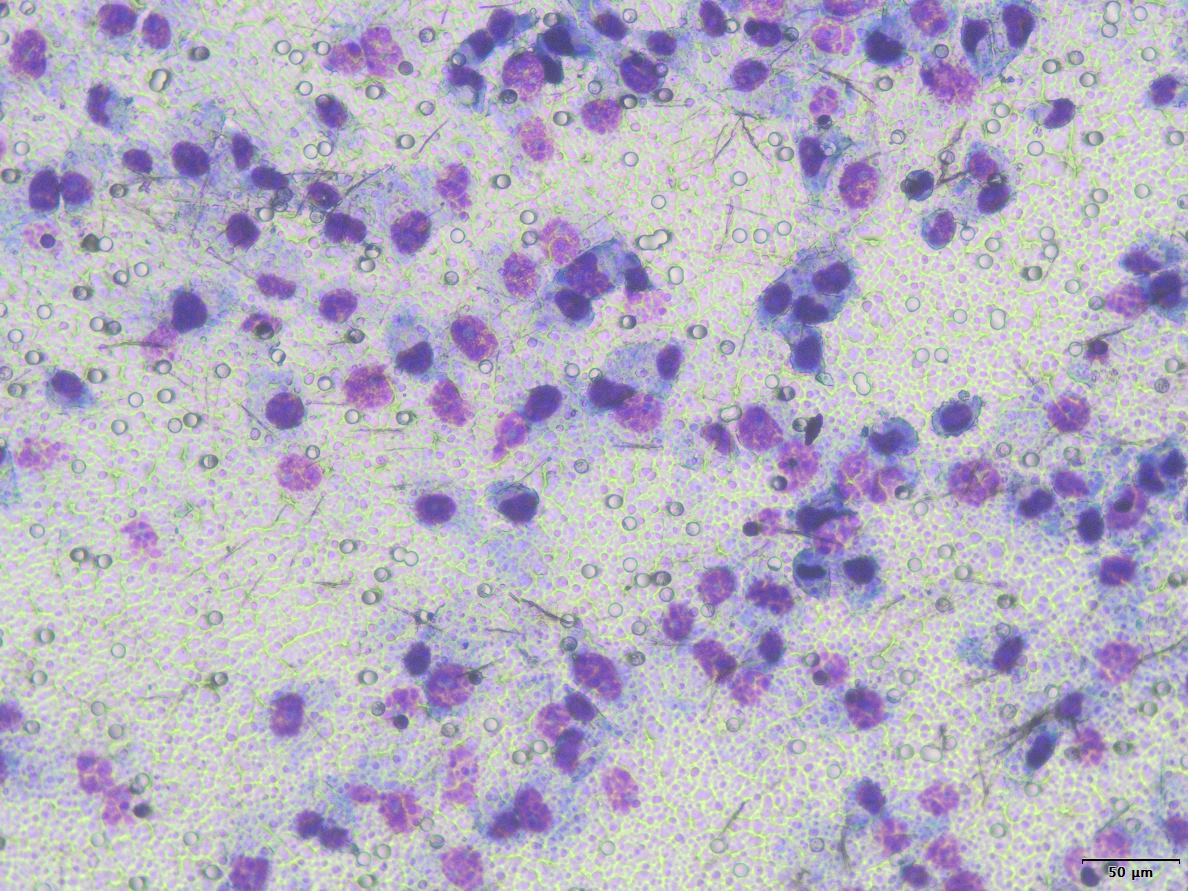

Supplement: Supplementary file 9 [file DataSheet_6.zip › Data Sheet 6/FigS1E/3-over-AC009948.5-INVASION.jpg]

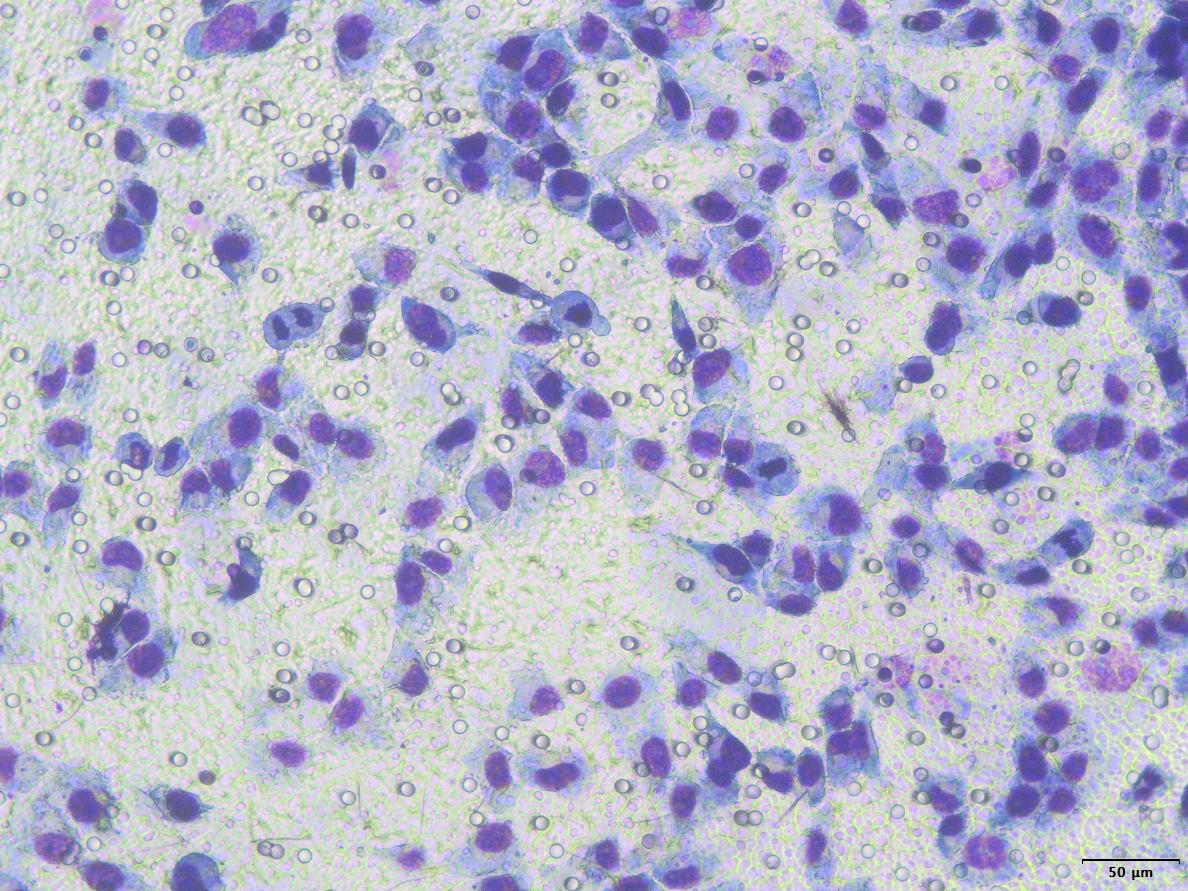

Supplement: Supplementary file 9 [file DataSheet_6.zip › Data Sheet 6/FigS1E/3-over-AC009948.5-M.jpg]

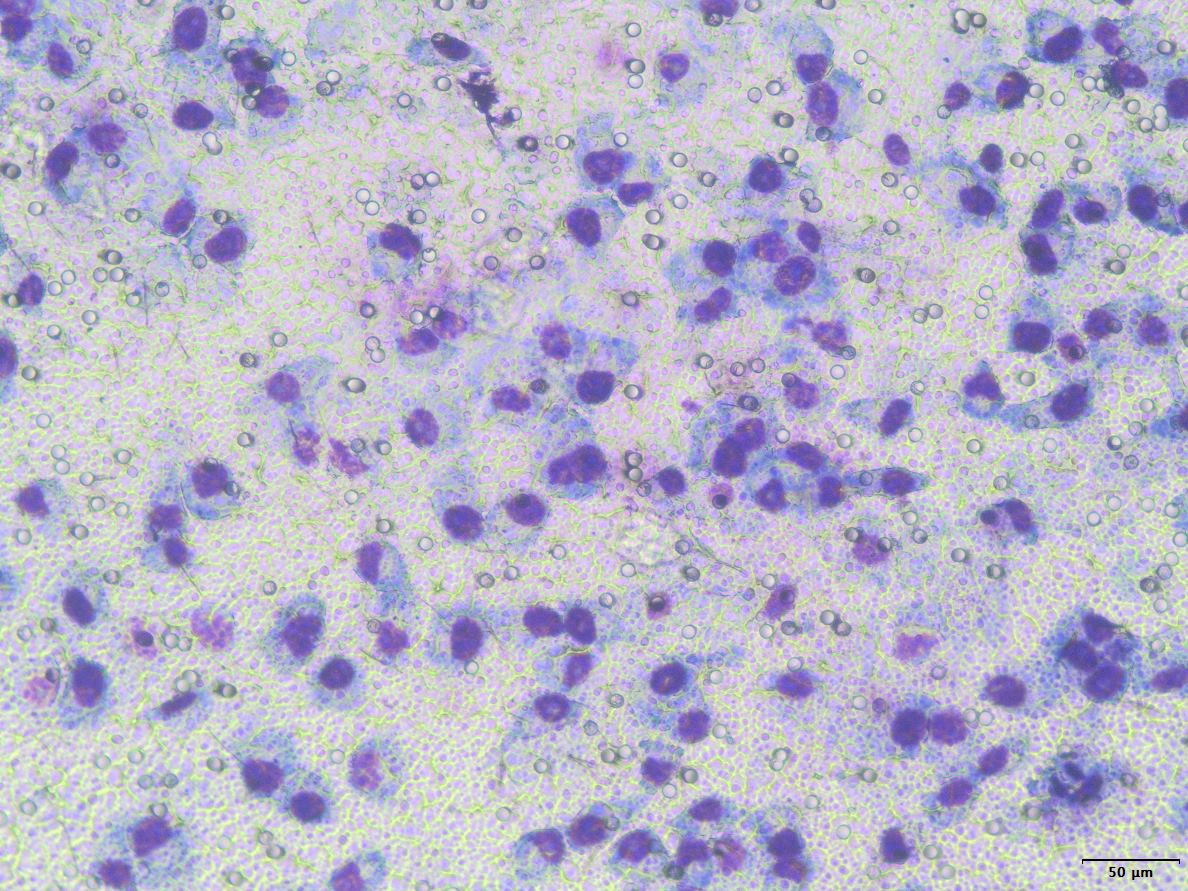

Supplement: Supplementary file 9 [file DataSheet_6.zip › Data Sheet 6/FigS1E/3-Scrambled-INVASION.jpg]

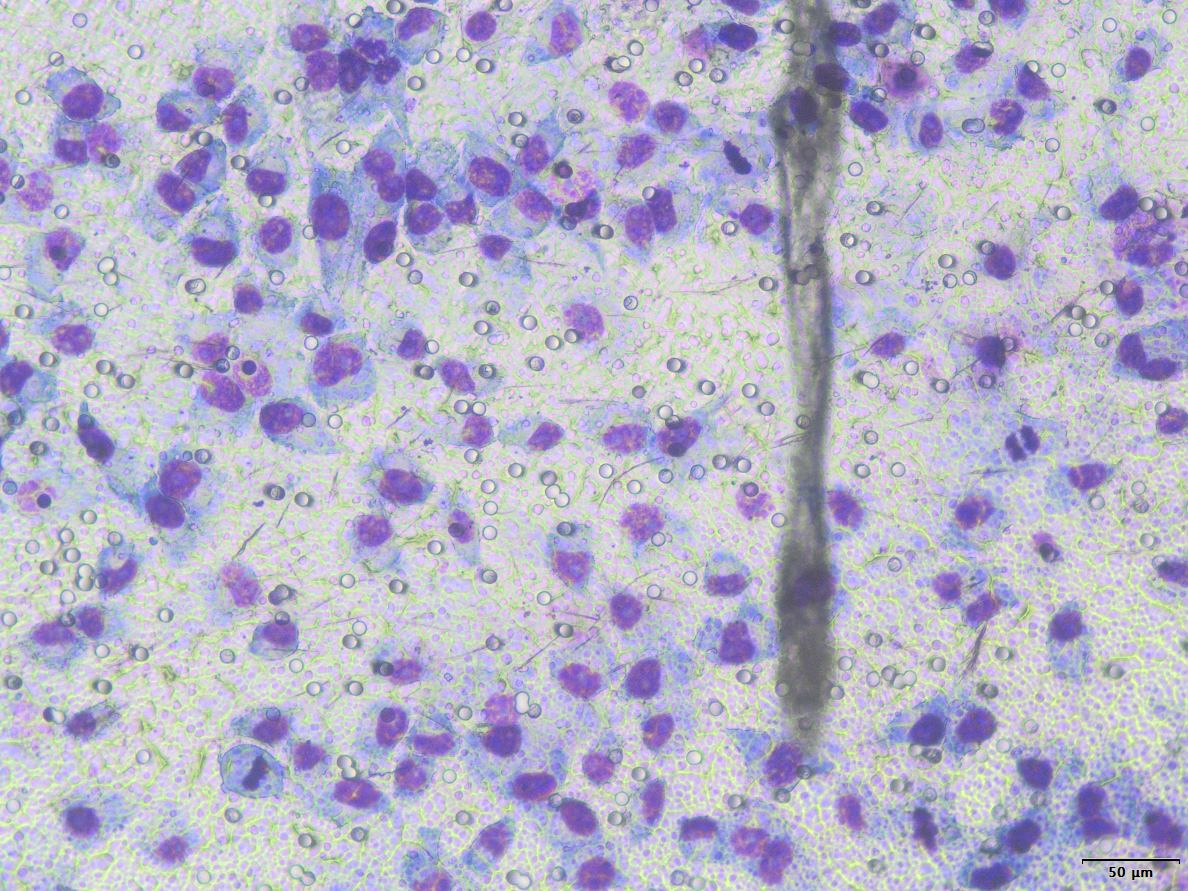

Supplement: Supplementary file 9 [file DataSheet_6.zip › Data Sheet 6/FigS1E/3-Scrambled-M.jpg]

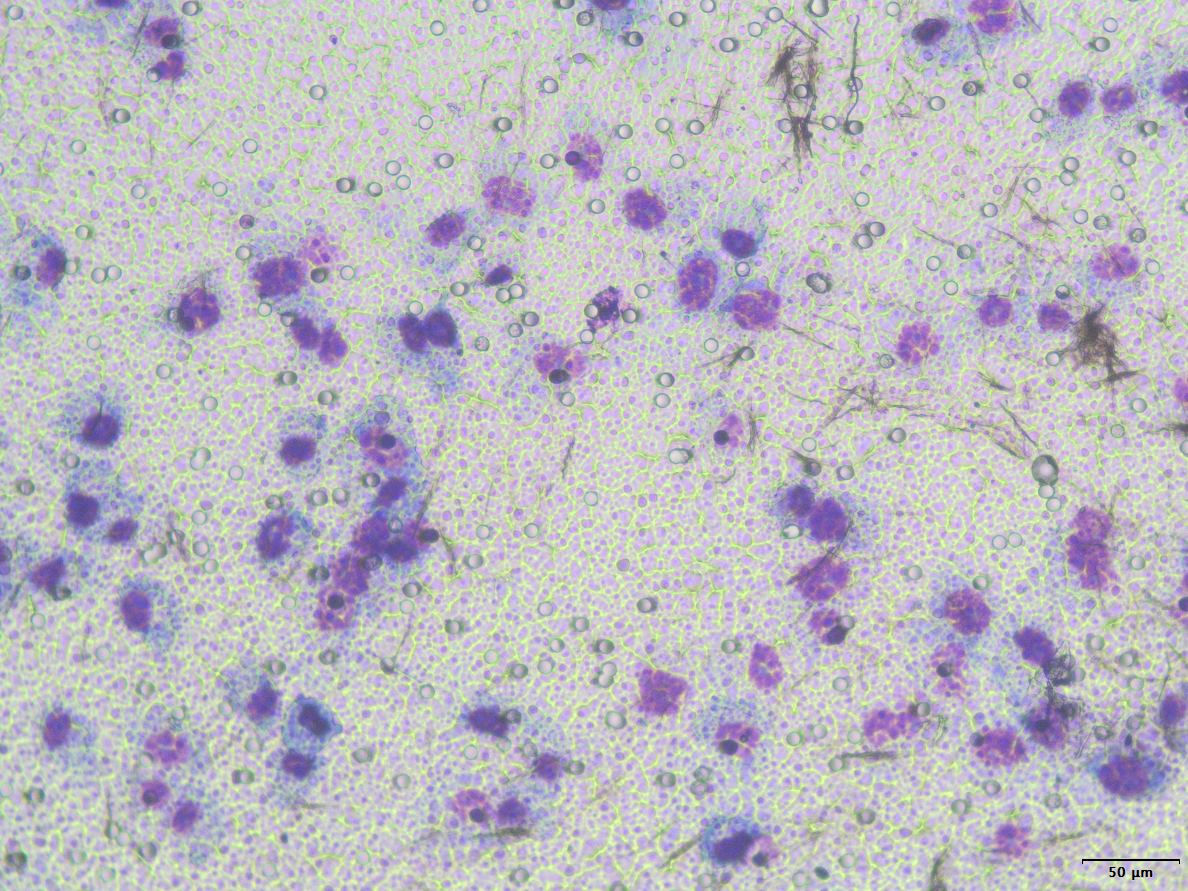

Supplement: Supplementary file 9 [file DataSheet_6.zip › Data Sheet 6/FigS1E/3-SiAC009948.5-INVASION.jpg]

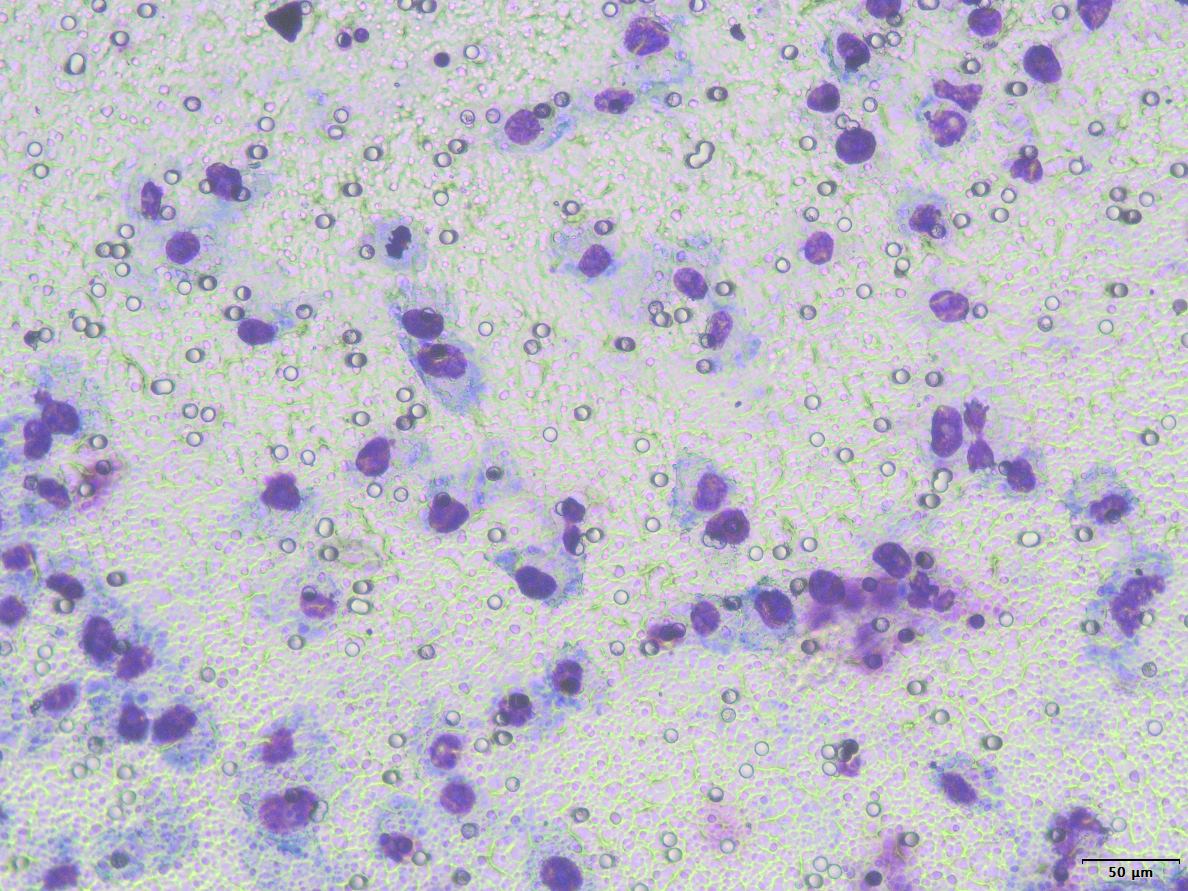

Supplement: Supplementary file 9 [file DataSheet_6.zip › Data Sheet 6/FigS1E/3-SiAC009948.5-M.jpg]

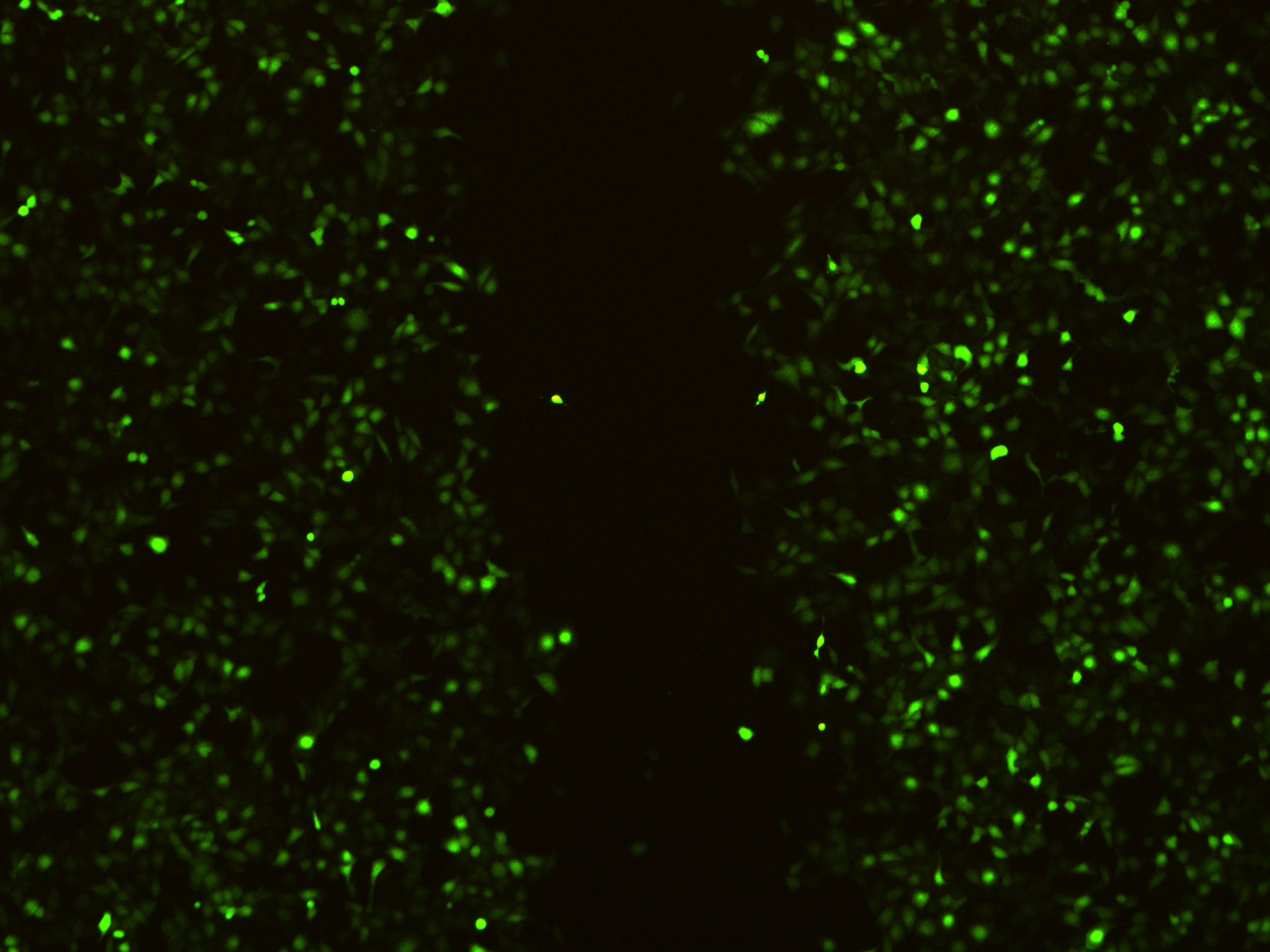

Supplement: Supplementary file 9 [file DataSheet_6.zip › Data Sheet 6/FigS1F/1-NC-0H.jpg]

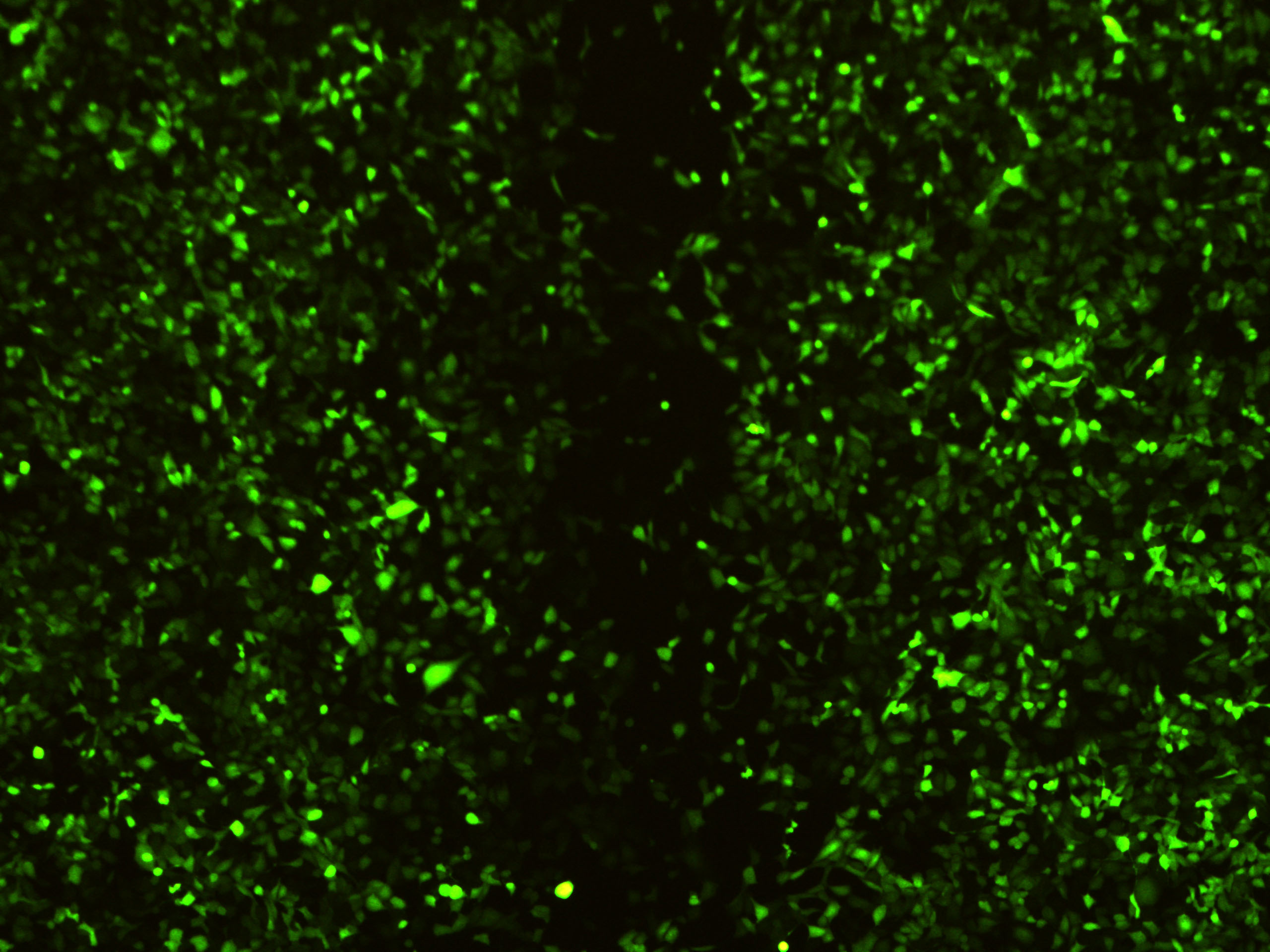

Supplement: Supplementary file 9 [file DataSheet_6.zip › Data Sheet 6/FigS1F/1-NC-24H.jpg]

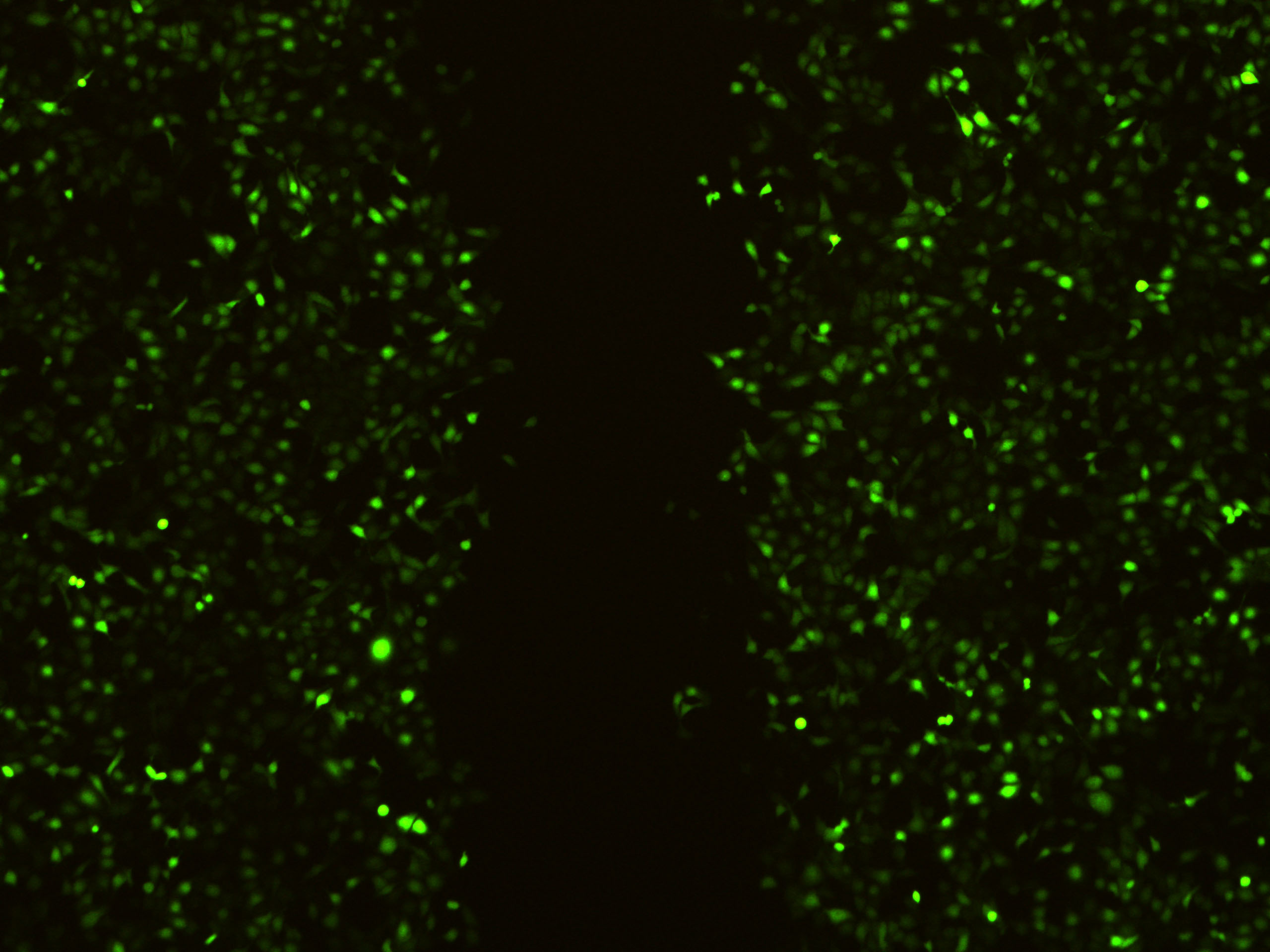

Supplement: Supplementary file 9 [file DataSheet_6.zip › Data Sheet 6/FigS1F/1-over-AC009948.5-0H.jpg]

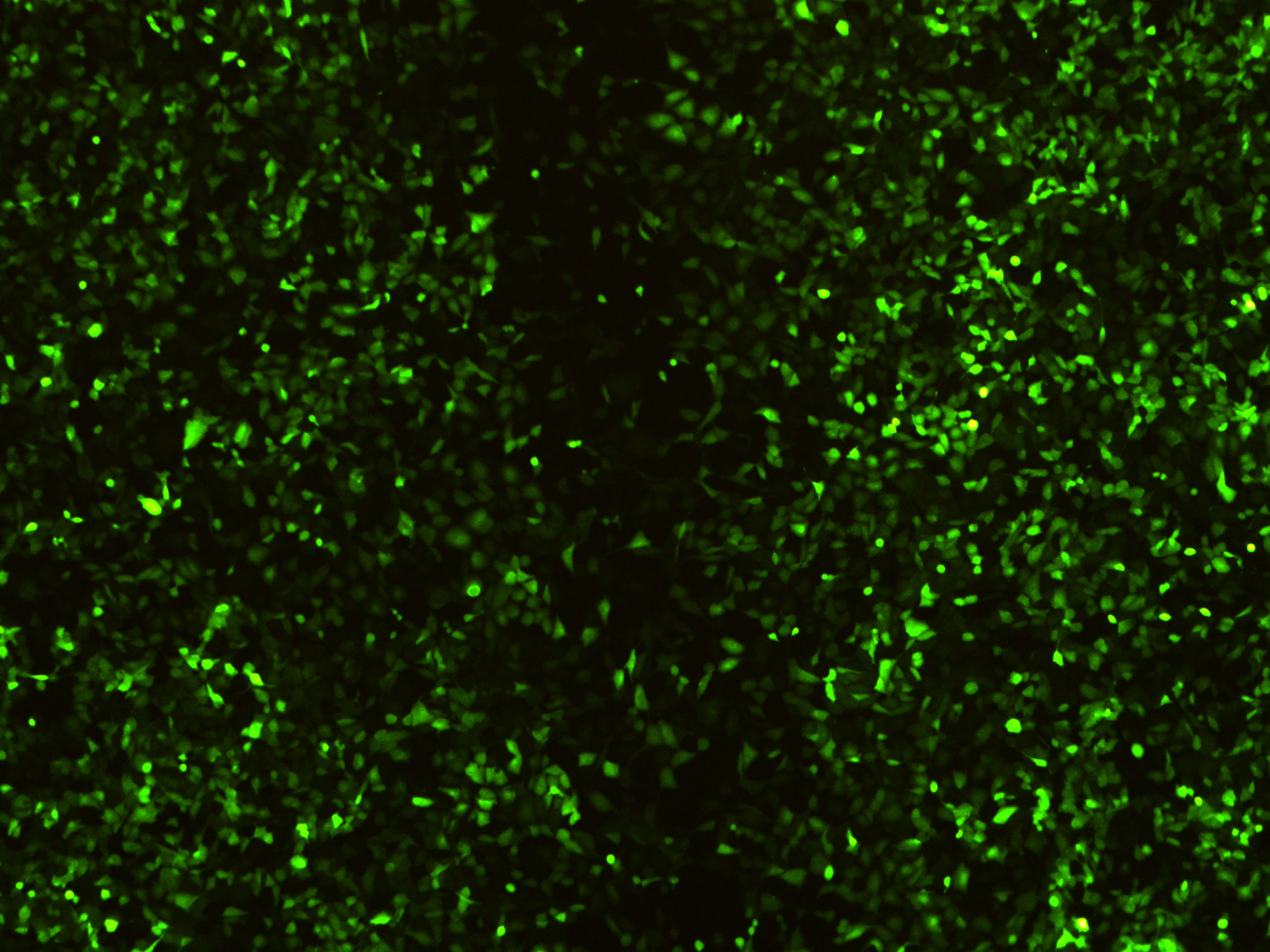

Supplement: Supplementary file 9 [file DataSheet_6.zip › Data Sheet 6/FigS1F/1-over-AC009948.5-24H.jpg]

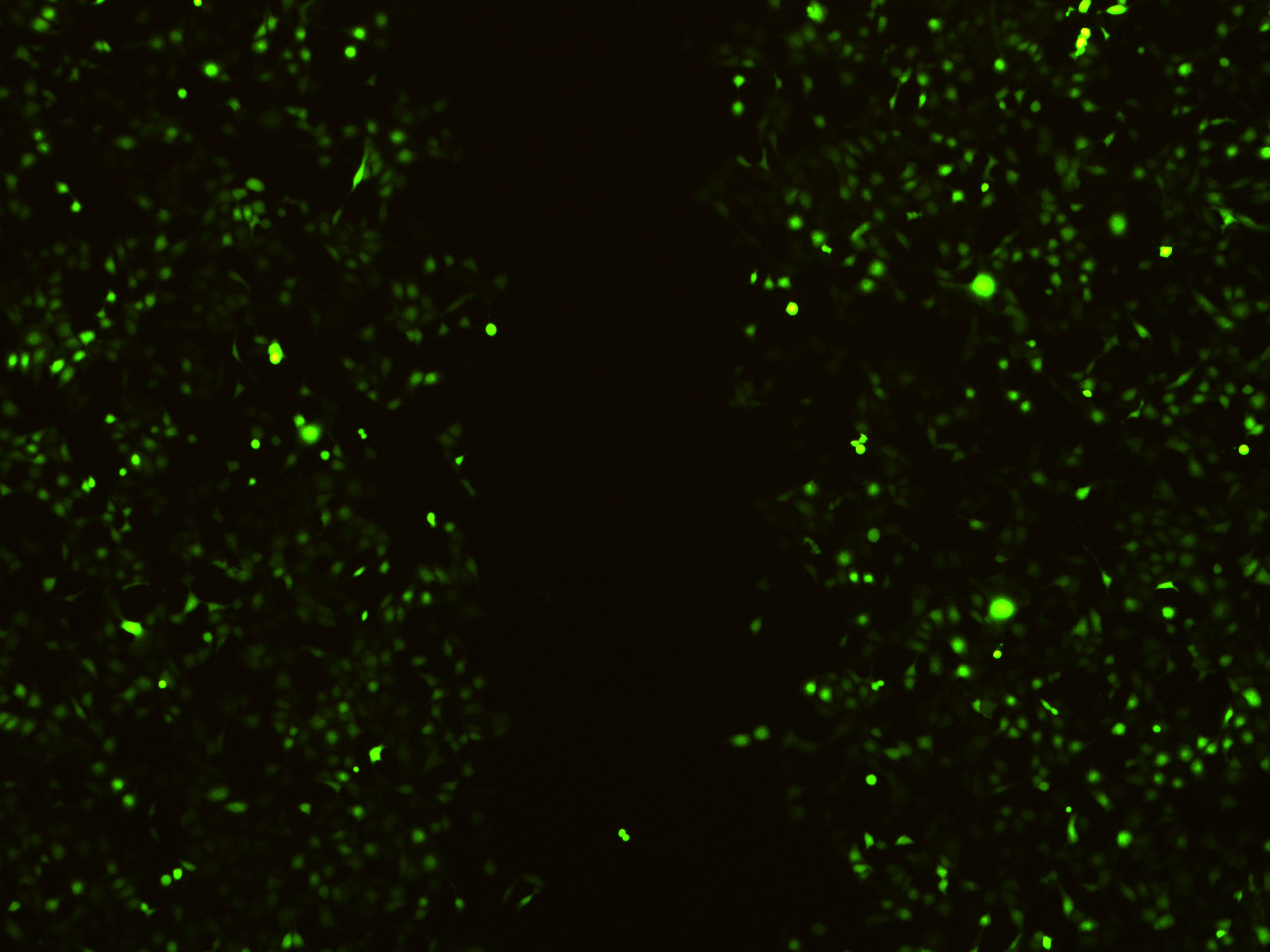

Supplement: Supplementary file 9 [file DataSheet_6.zip › Data Sheet 6/FigS1F/1-scrambled-0H.jpg]

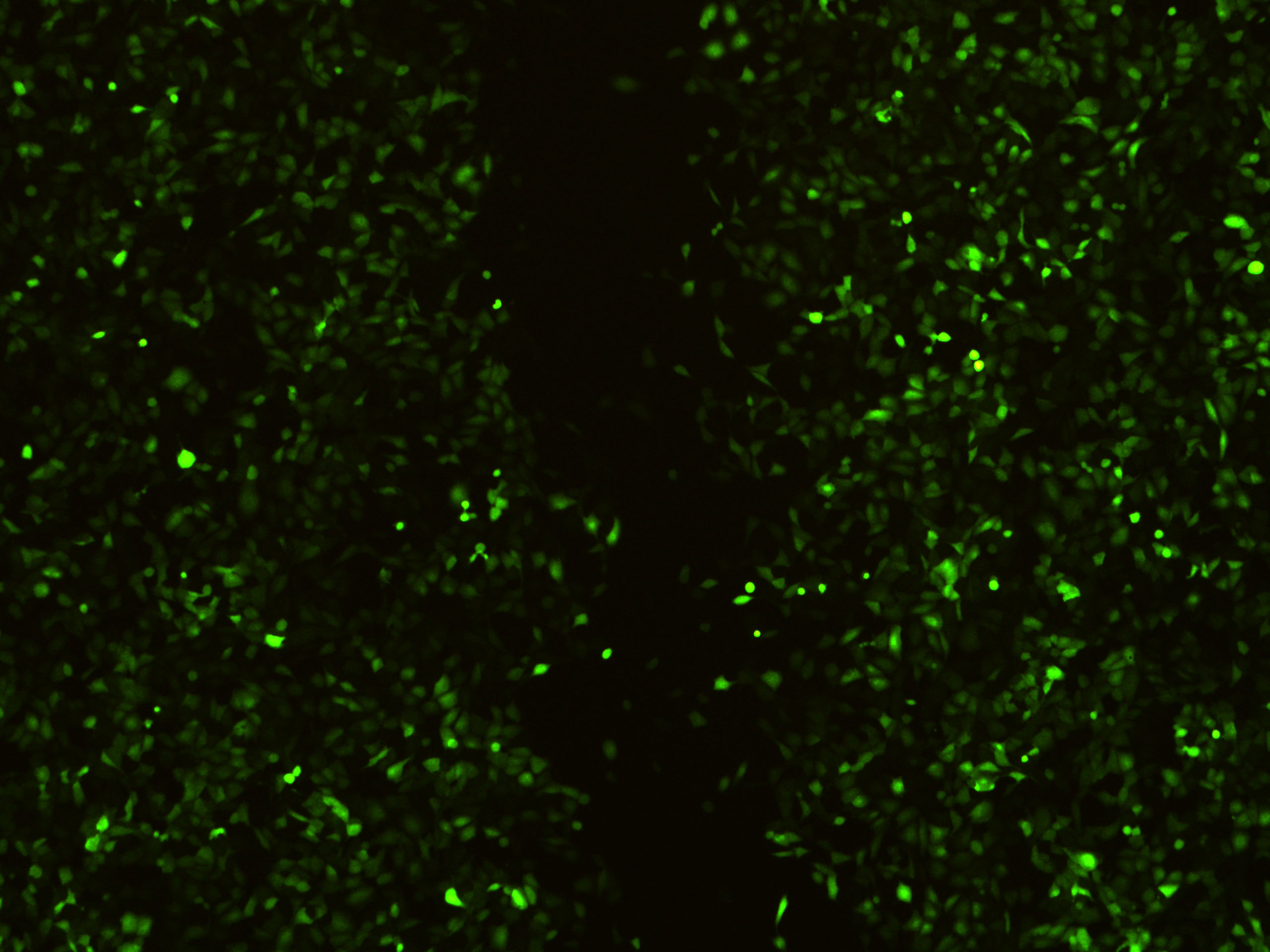

Supplement: Supplementary file 9 [file DataSheet_6.zip › Data Sheet 6/FigS1F/1-scrambled-24H.jpg]

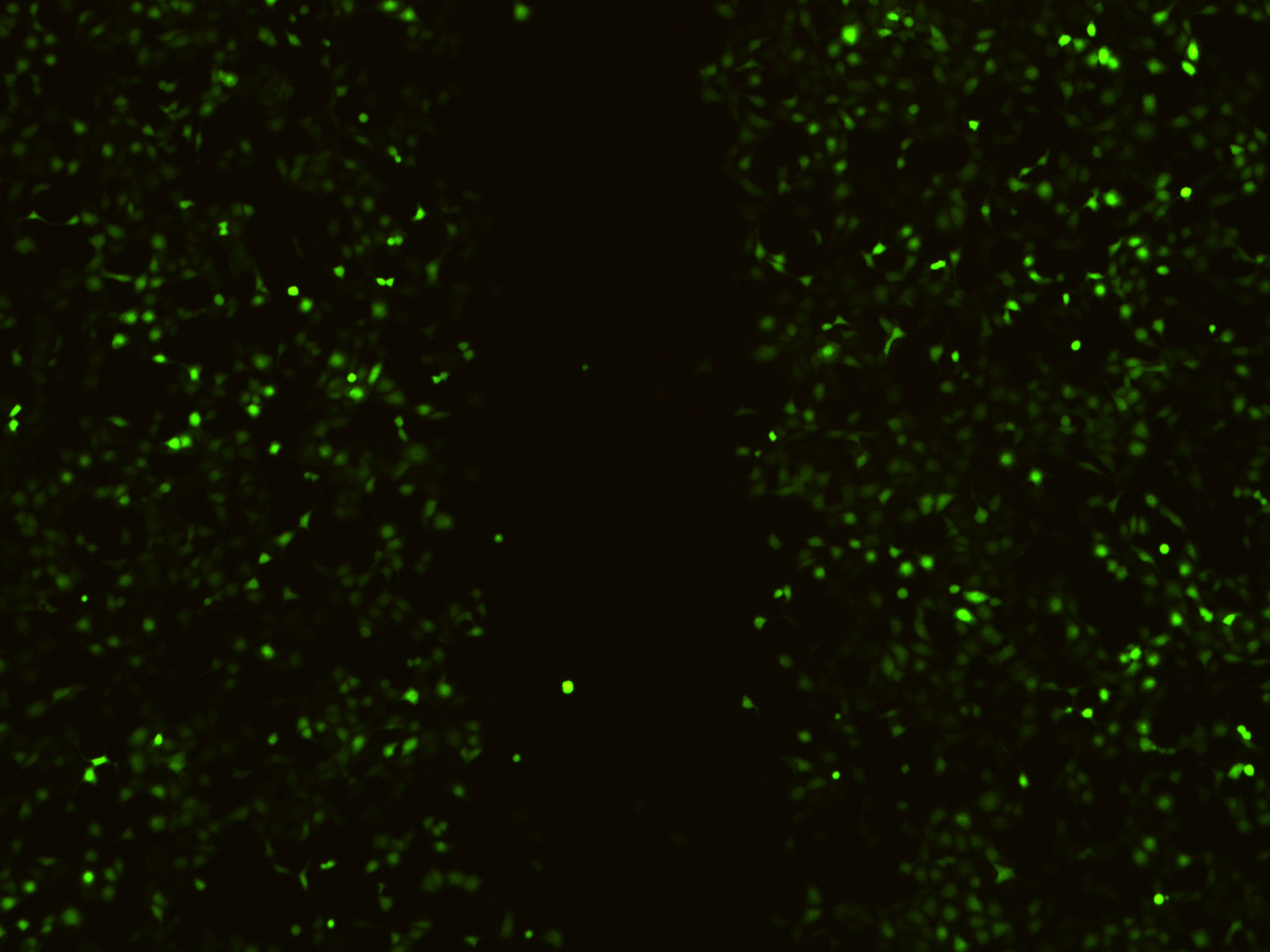

Supplement: Supplementary file 9 [file DataSheet_6.zip › Data Sheet 6/FigS1F/1-SiAC009948.5-0H.jpg]

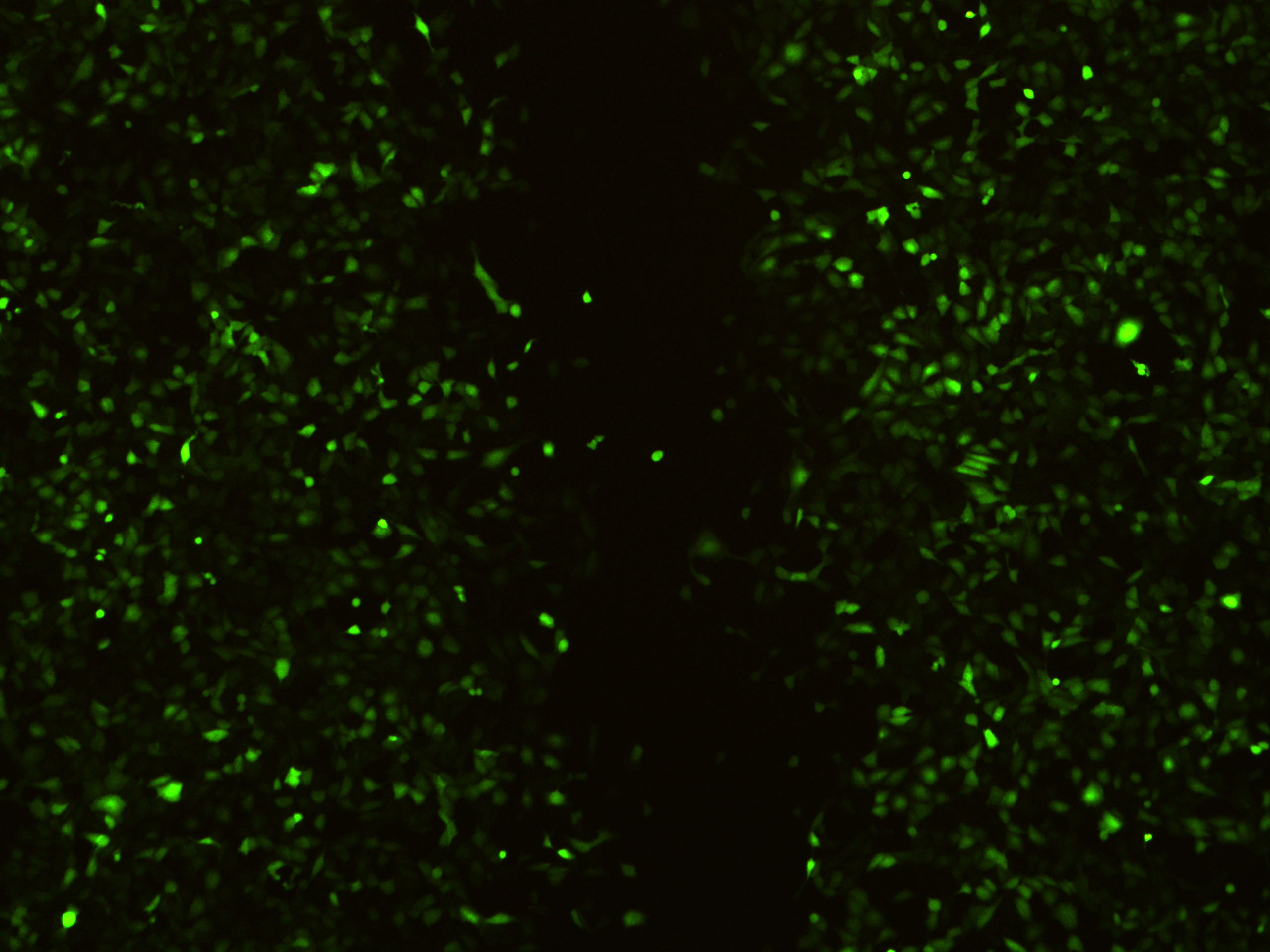

Supplement: Supplementary file 9 [file DataSheet_6.zip › Data Sheet 6/FigS1F/1-siAC009948.5-24H.jpg]

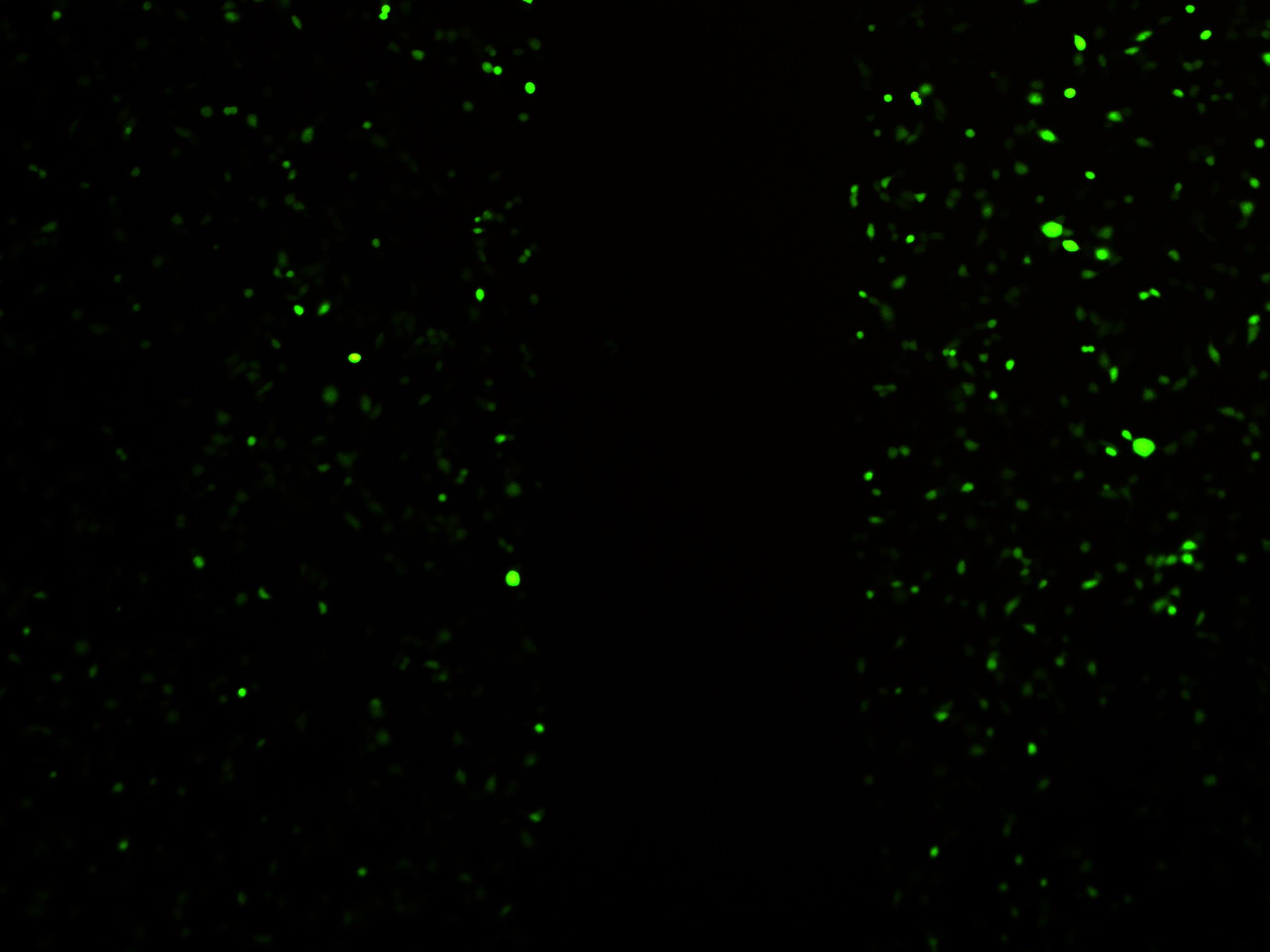

Supplement: Supplementary file 9 [file DataSheet_6.zip › Data Sheet 6/FigS1F/2-NC-0H.jpg]

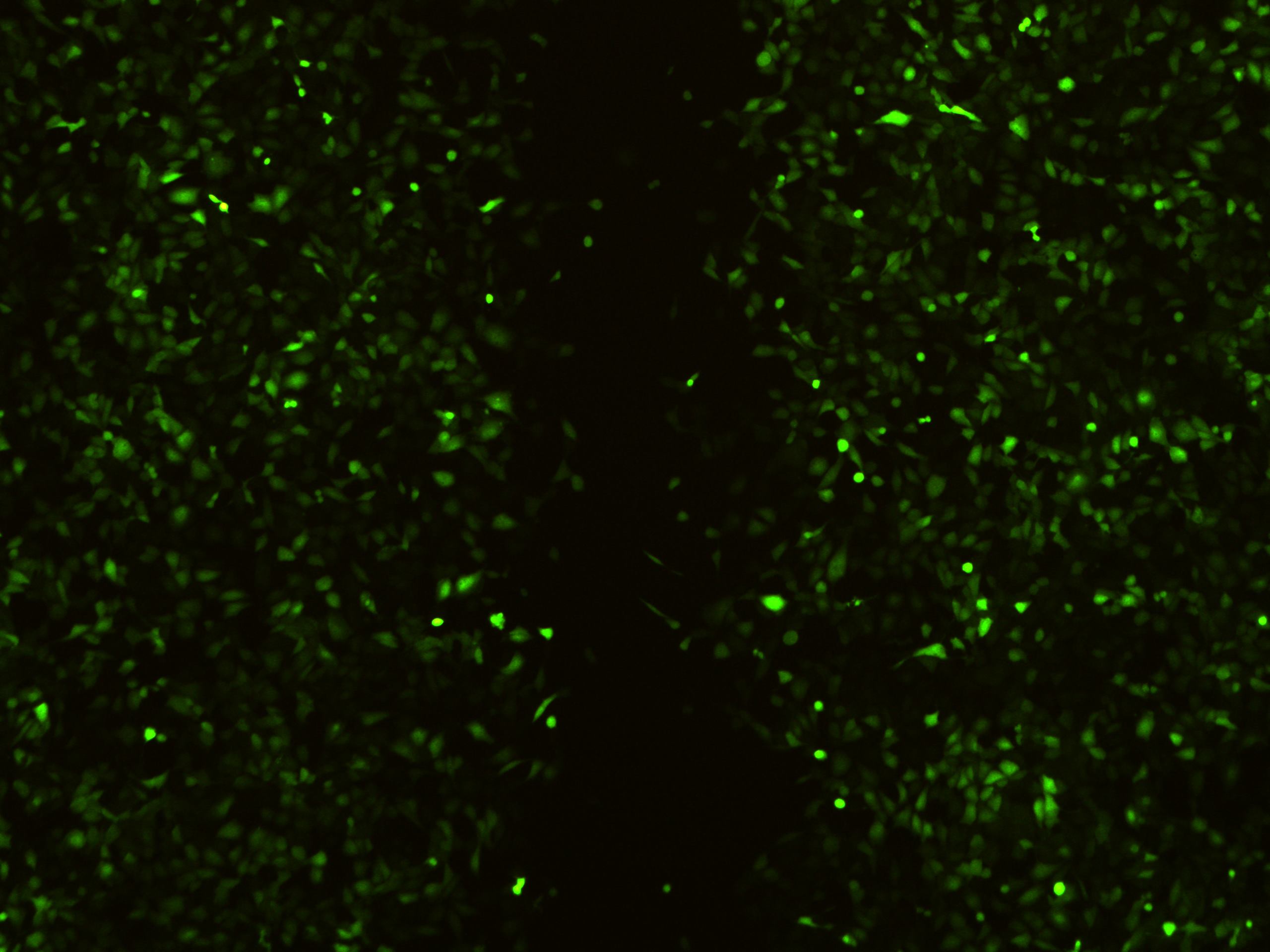

Supplement: Supplementary file 9 [file DataSheet_6.zip › Data Sheet 6/FigS1F/2-NC-24H.jpg]

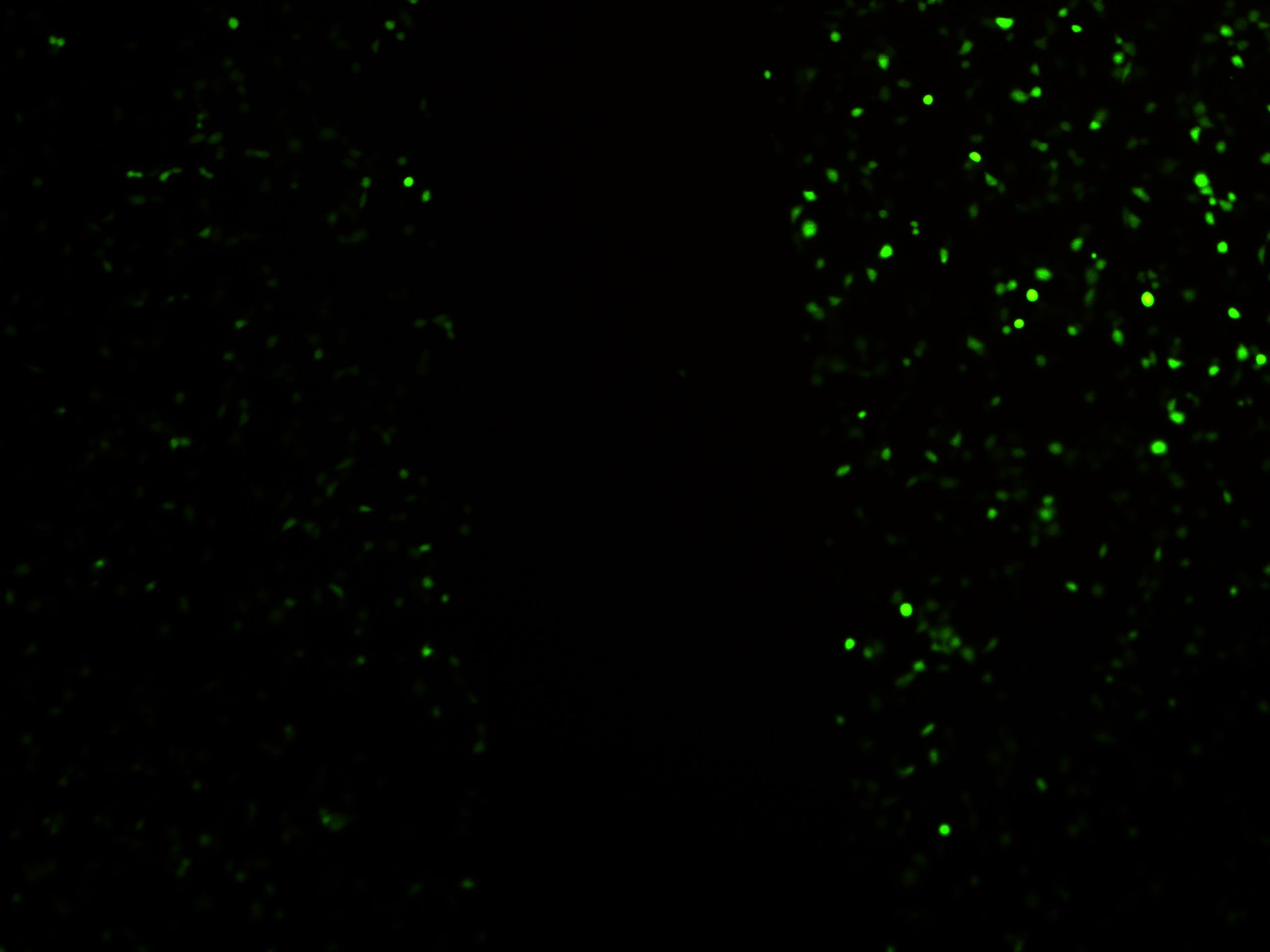

Supplement: Supplementary file 9 [file DataSheet_6.zip › Data Sheet 6/FigS1F/2-over-AC009948.5-0H.jpg]

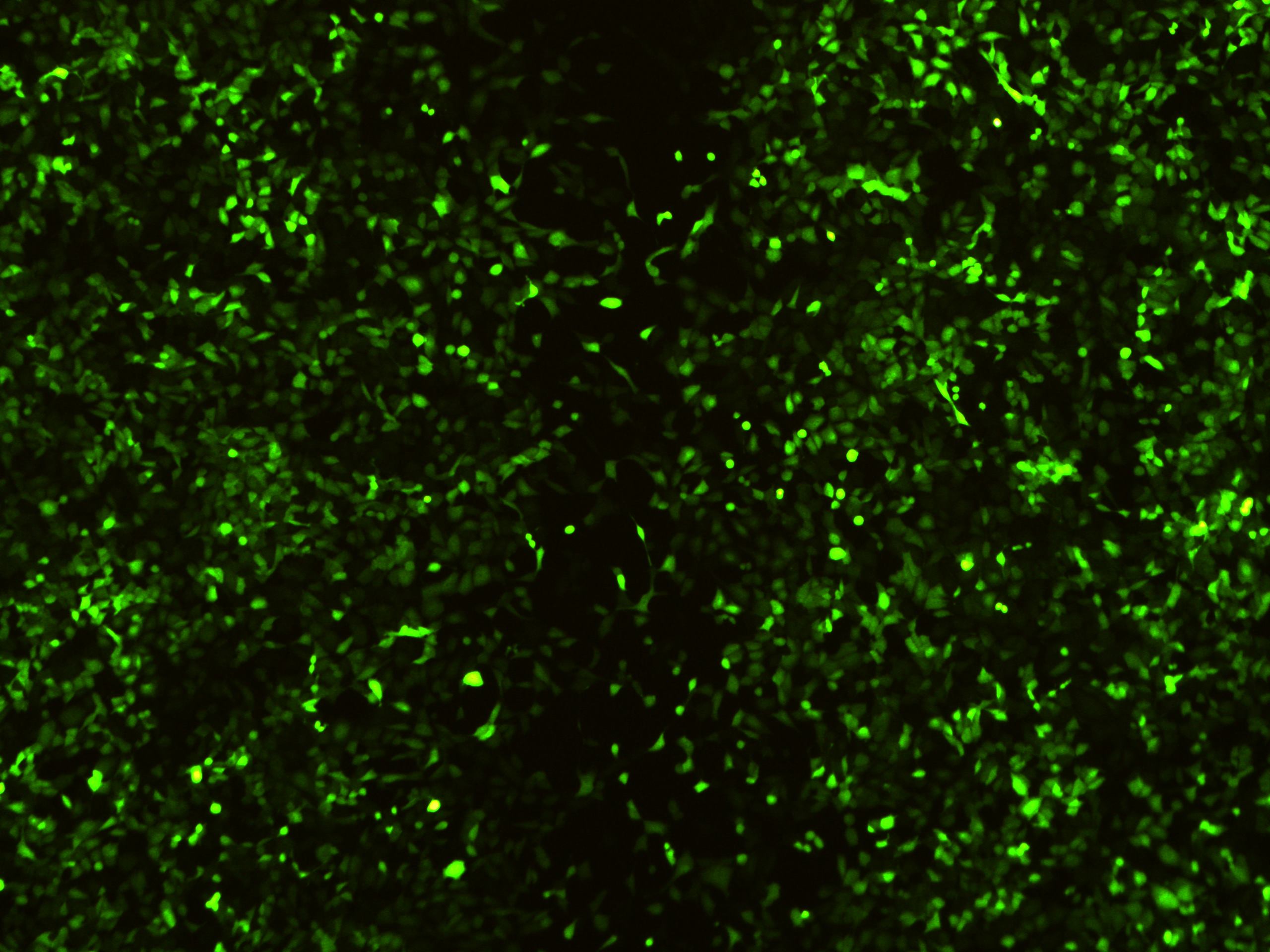

Supplement: Supplementary file 9 [file DataSheet_6.zip › Data Sheet 6/FigS1F/2-over-AC009948.5-24H.jpg]

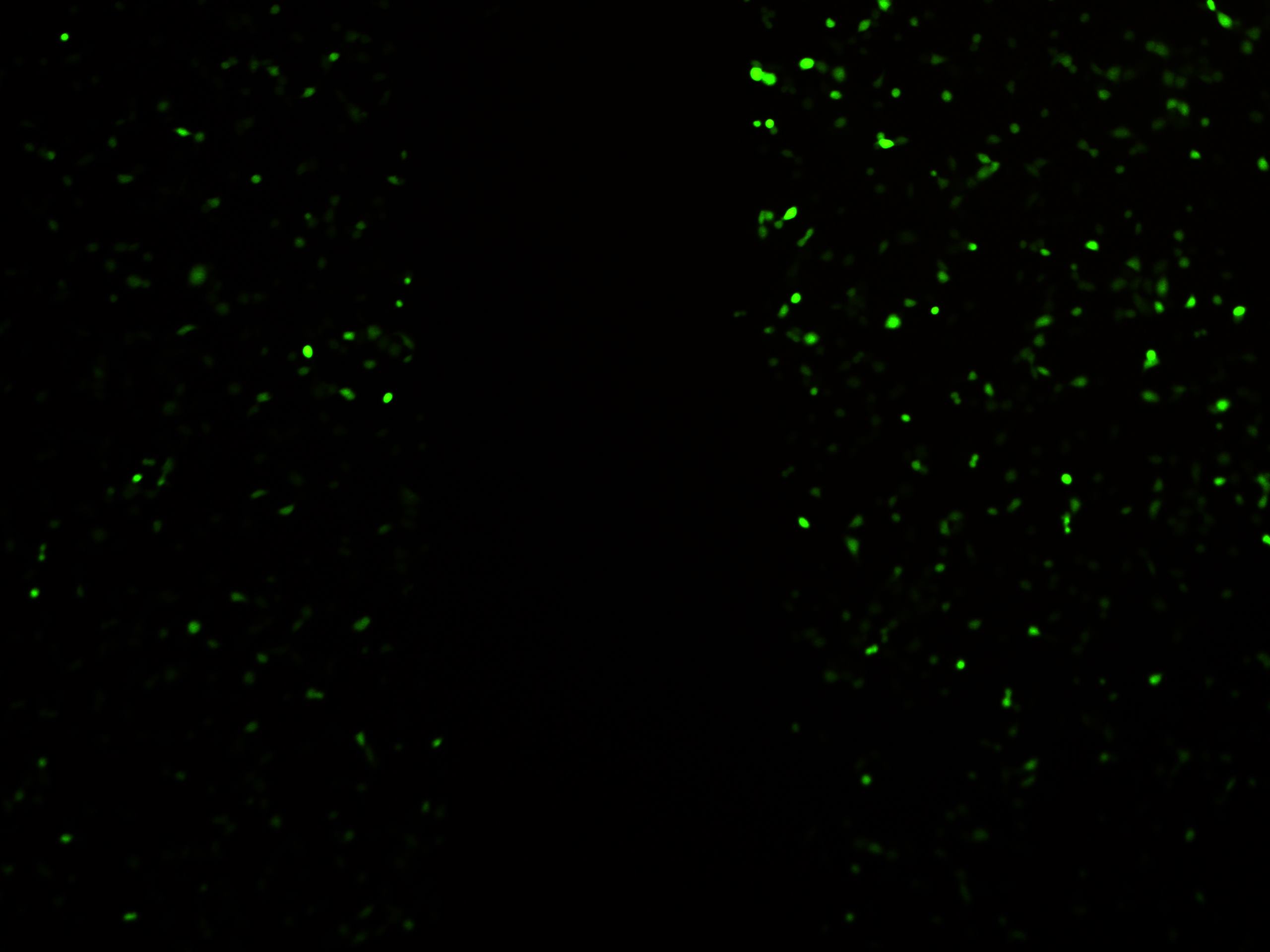

Supplement: Supplementary file 9 [file DataSheet_6.zip › Data Sheet 6/FigS1F/2-scrambled-0H.jpg]

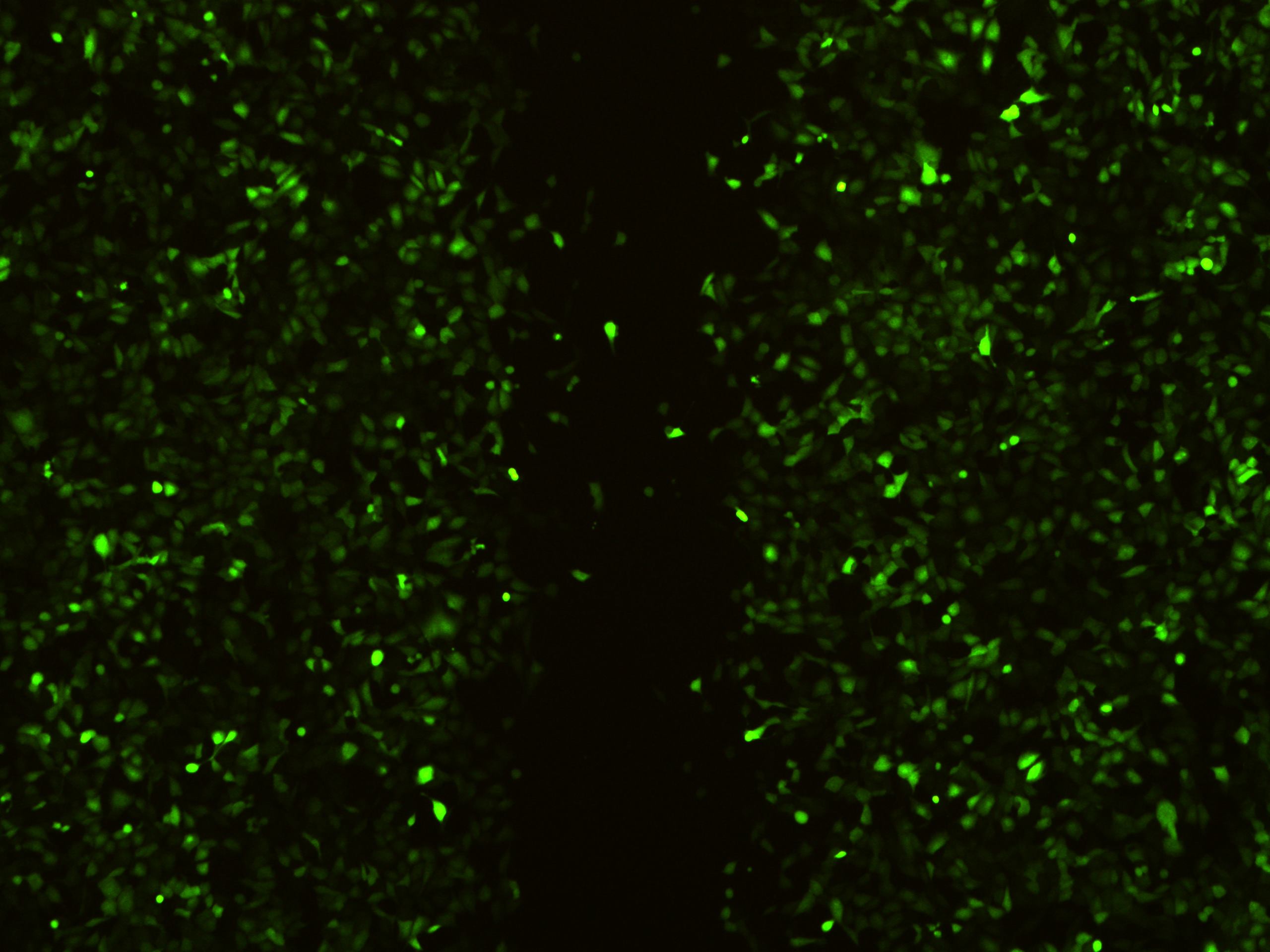

Supplement: Supplementary file 9 [file DataSheet_6.zip › Data Sheet 6/FigS1F/2-scrambled-24H.jpg]

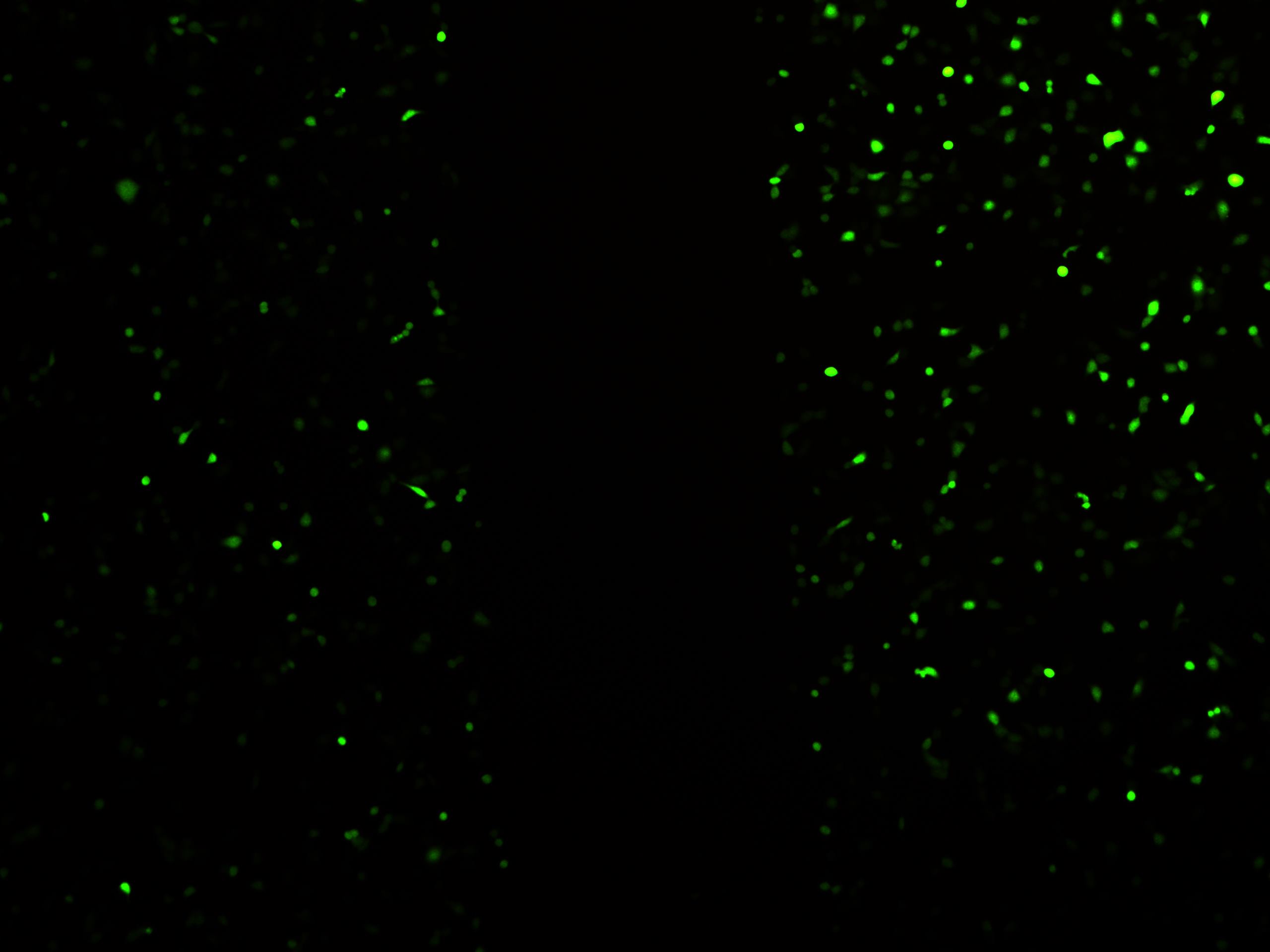

Supplement: Supplementary file 9 [file DataSheet_6.zip › Data Sheet 6/FigS1F/2-SiAC009948.5-0H.jpg]

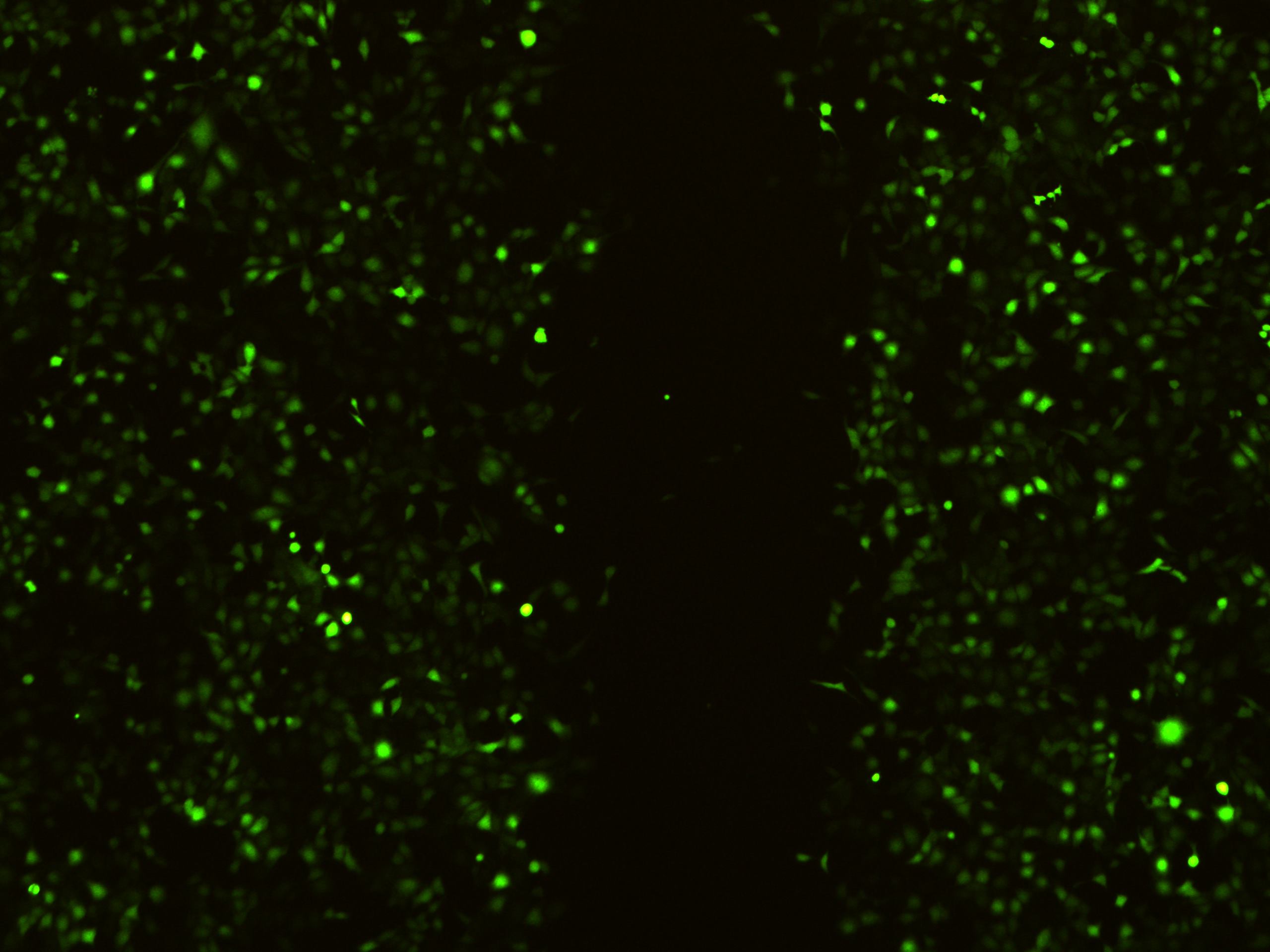

Supplement: Supplementary file 9 [file DataSheet_6.zip › Data Sheet 6/FigS1F/2-siAC009948.5-24H.jpg]

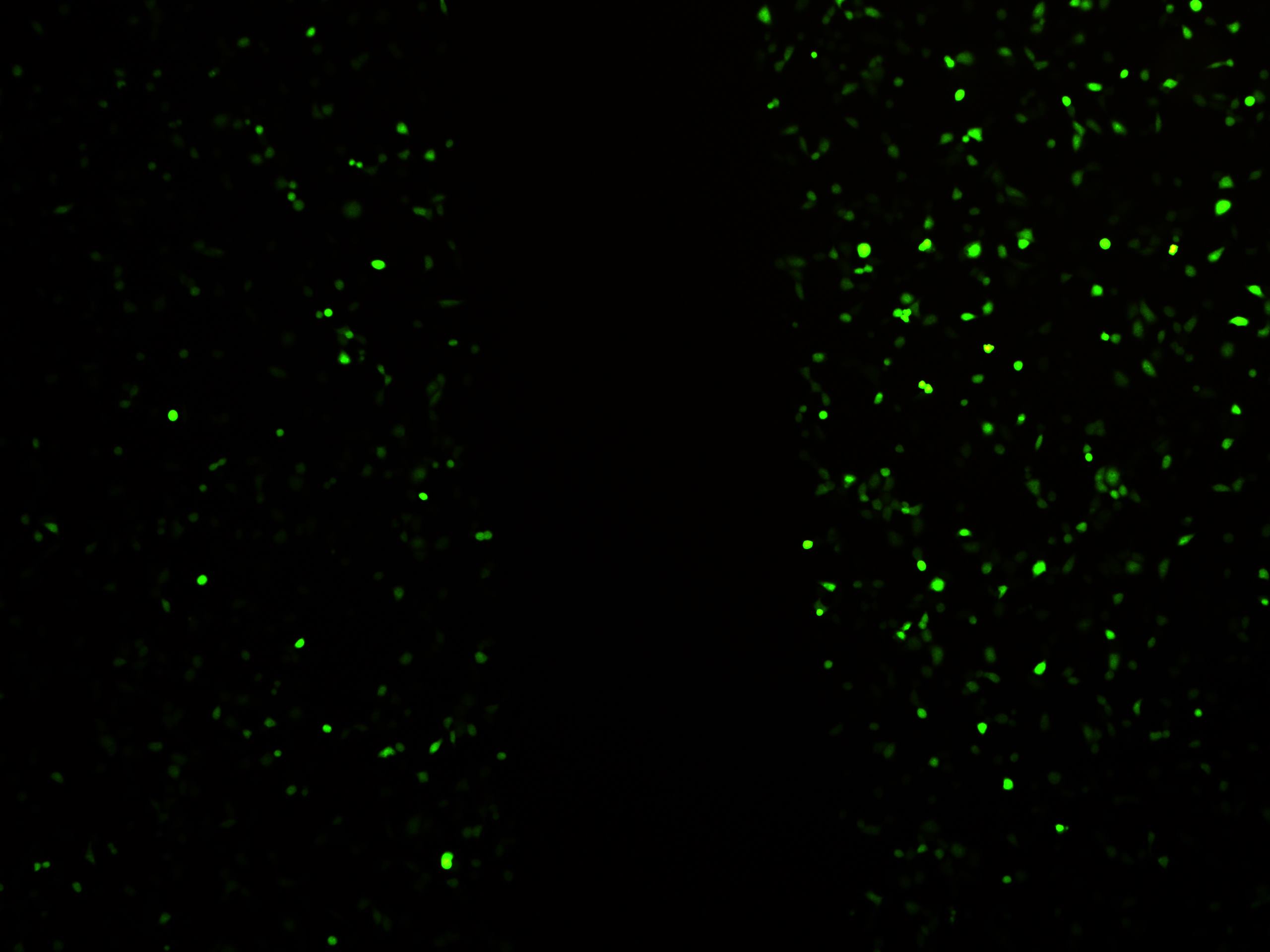

Supplement: Supplementary file 9 [file DataSheet_6.zip › Data Sheet 6/FigS1F/3-NC-0H.jpg]

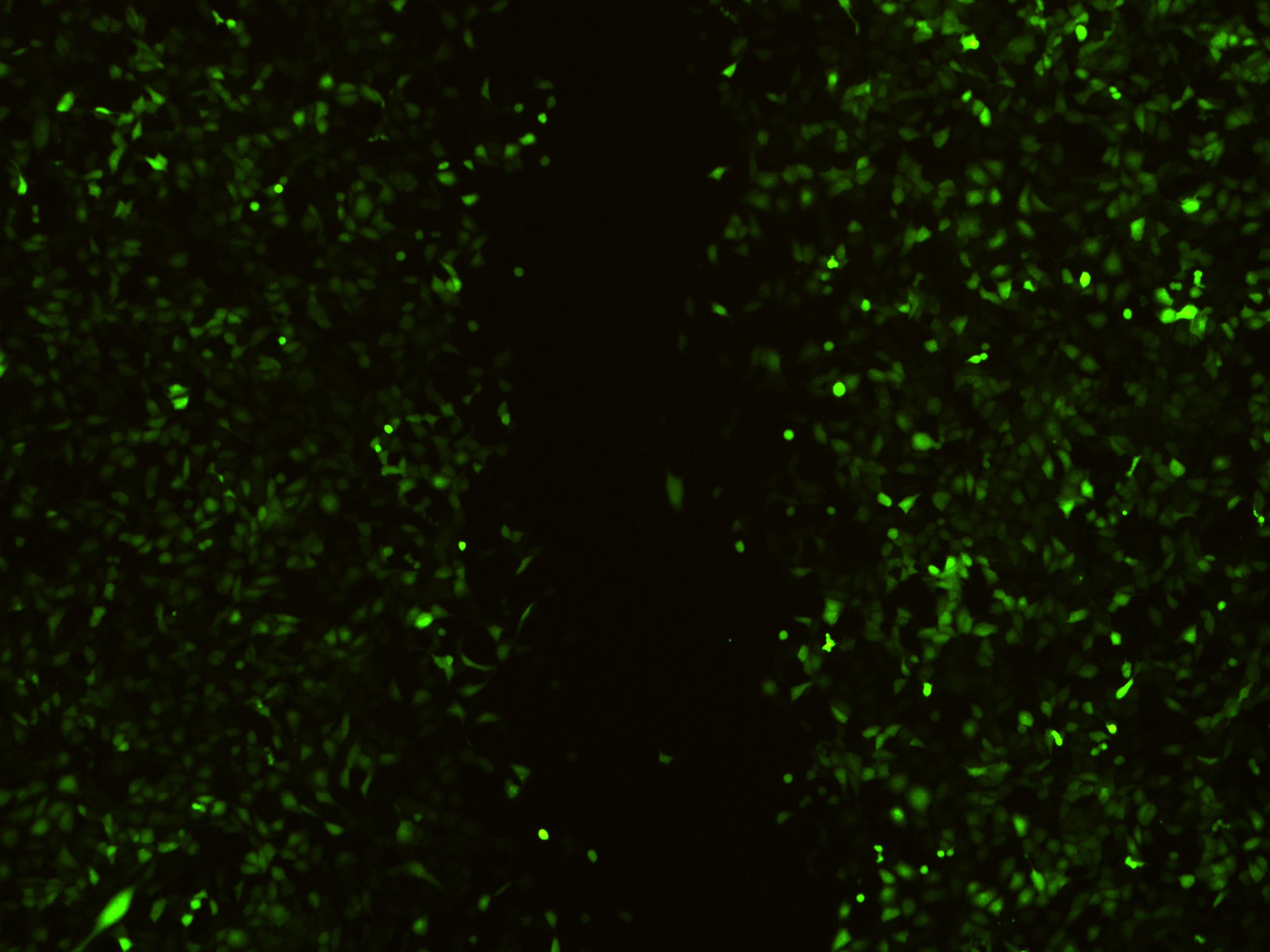

Supplement: Supplementary file 9 [file DataSheet_6.zip › Data Sheet 6/FigS1F/3-NC-24H.jpg]

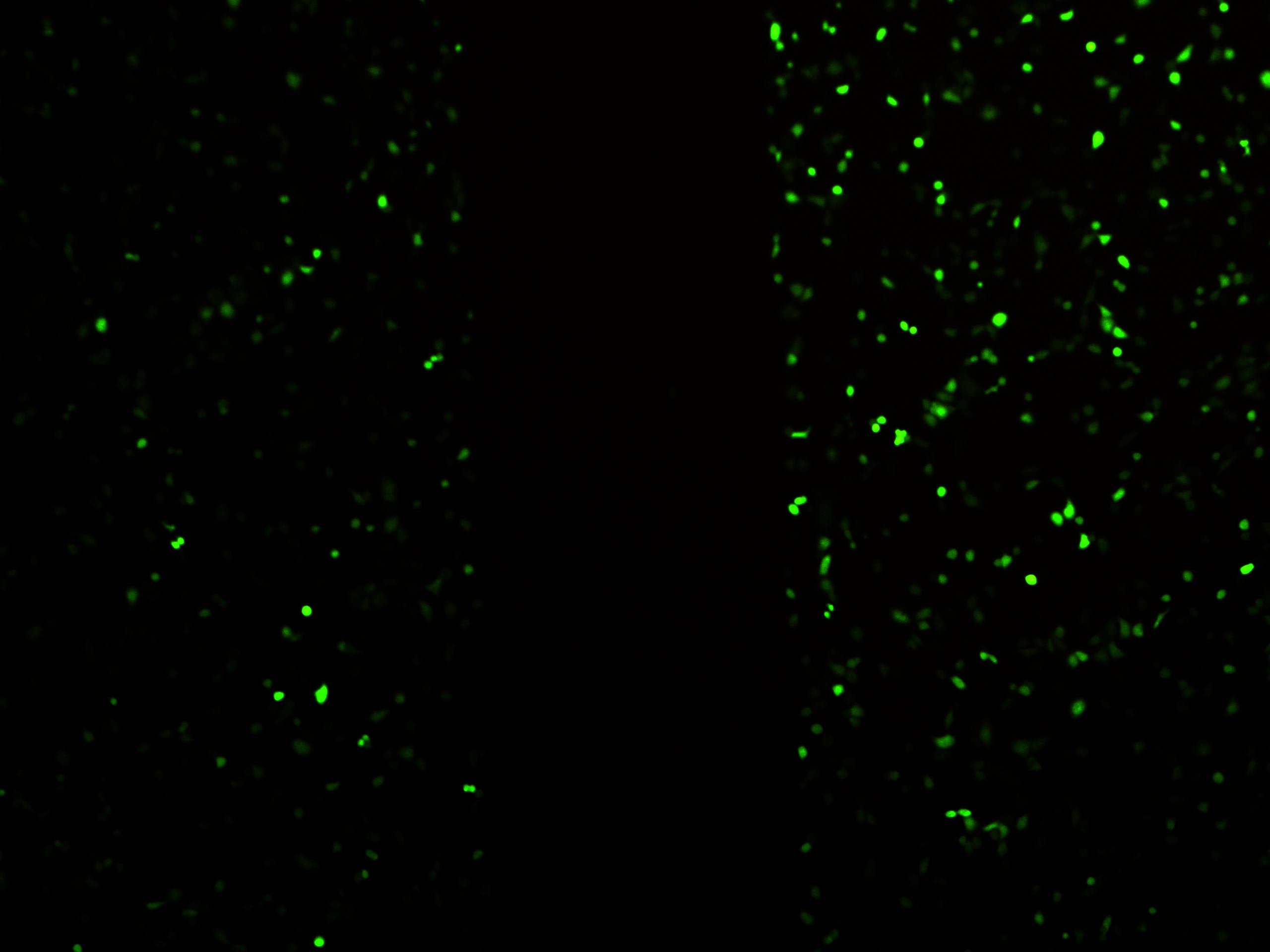

Supplement: Supplementary file 9 [file DataSheet_6.zip › Data Sheet 6/FigS1F/3-over-AC009948.5-0H.jpg]

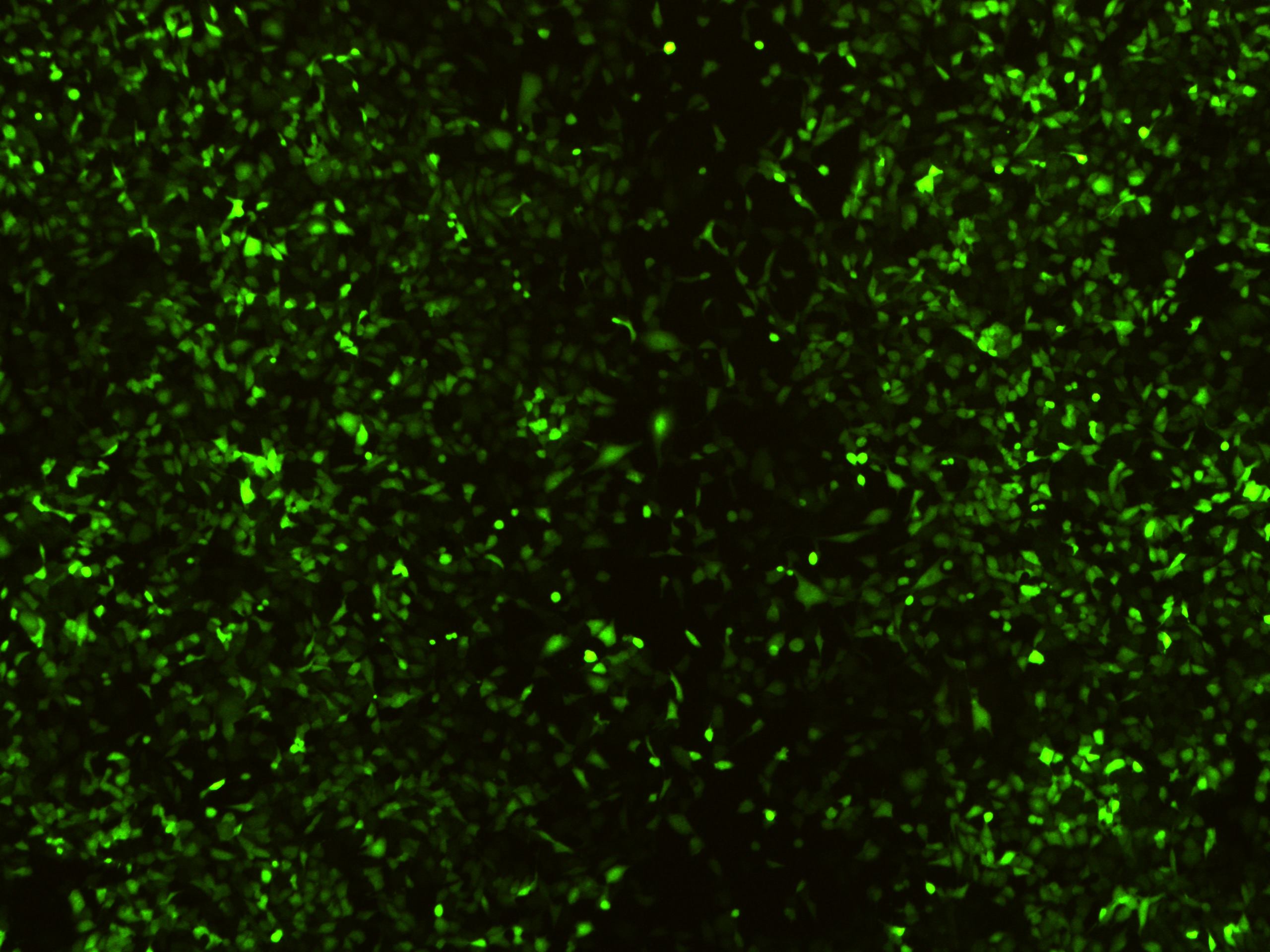

Supplement: Supplementary file 9 [file DataSheet_6.zip › Data Sheet 6/FigS1F/3-over-AC009948.5-24H.jpg]

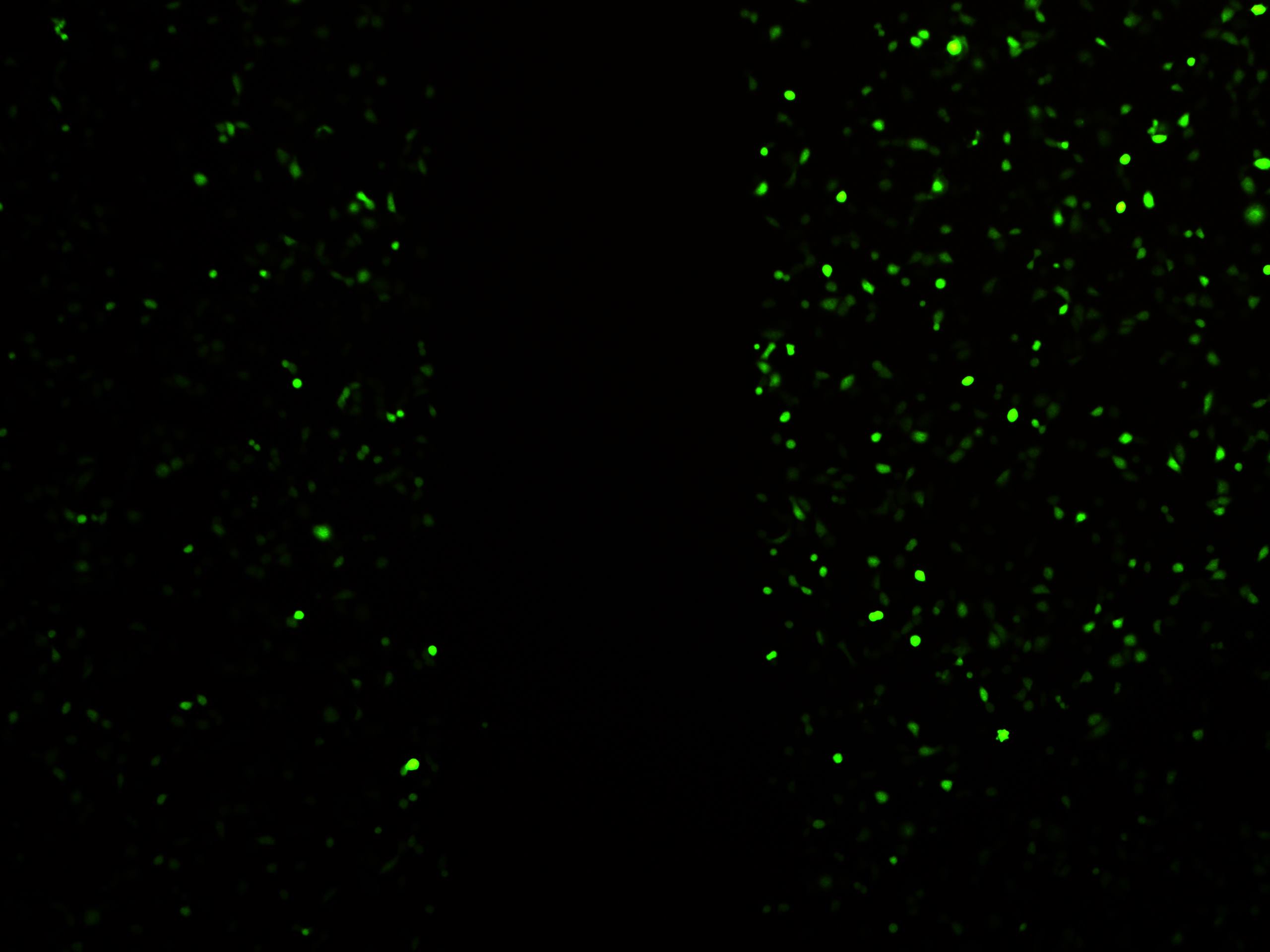

Supplement: Supplementary file 9 [file DataSheet_6.zip › Data Sheet 6/FigS1F/3-scrambled-0H.jpg]

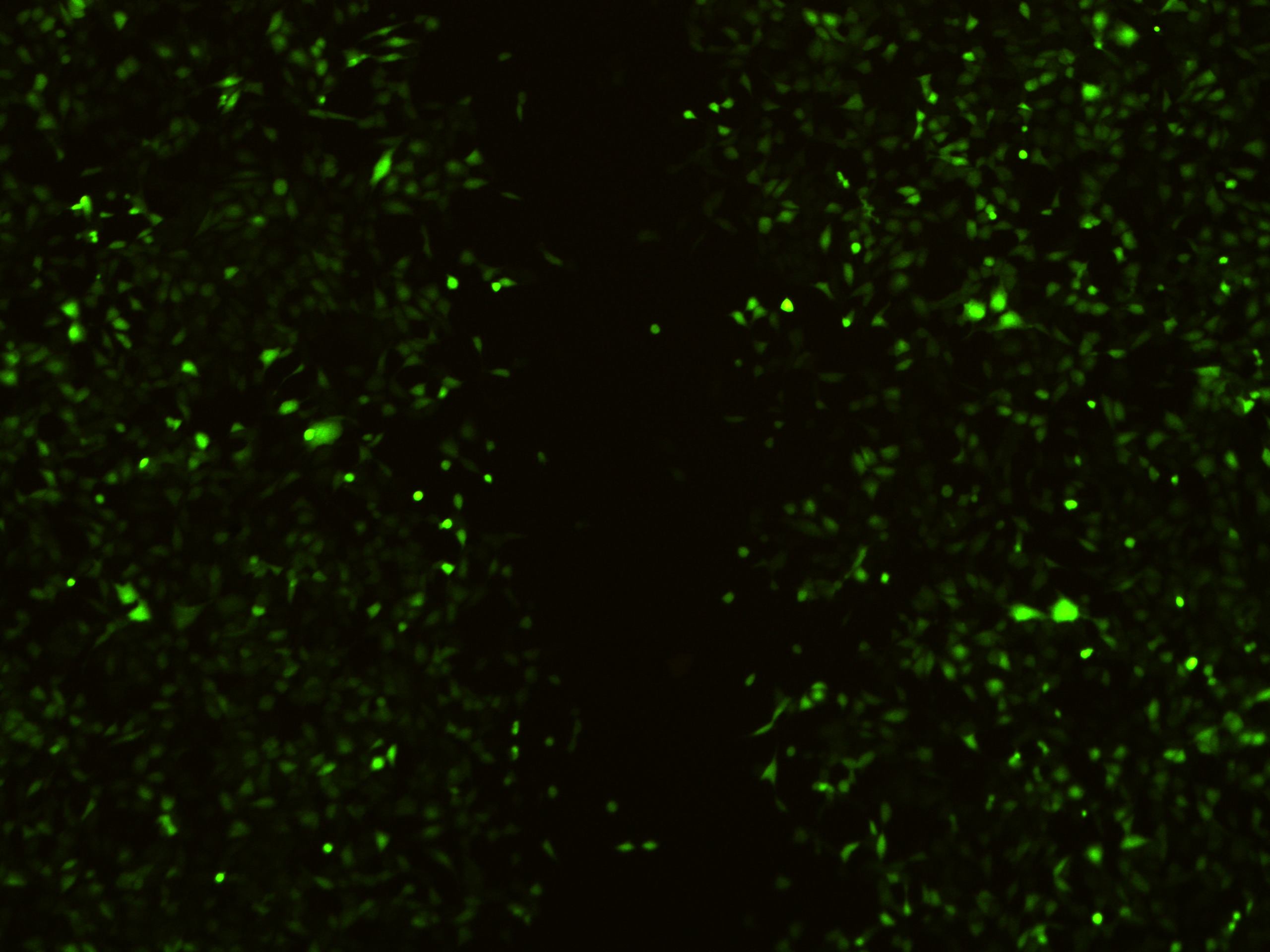

Supplement: Supplementary file 9 [file DataSheet_6.zip › Data Sheet 6/FigS1F/3-scrambled-24H.jpg]

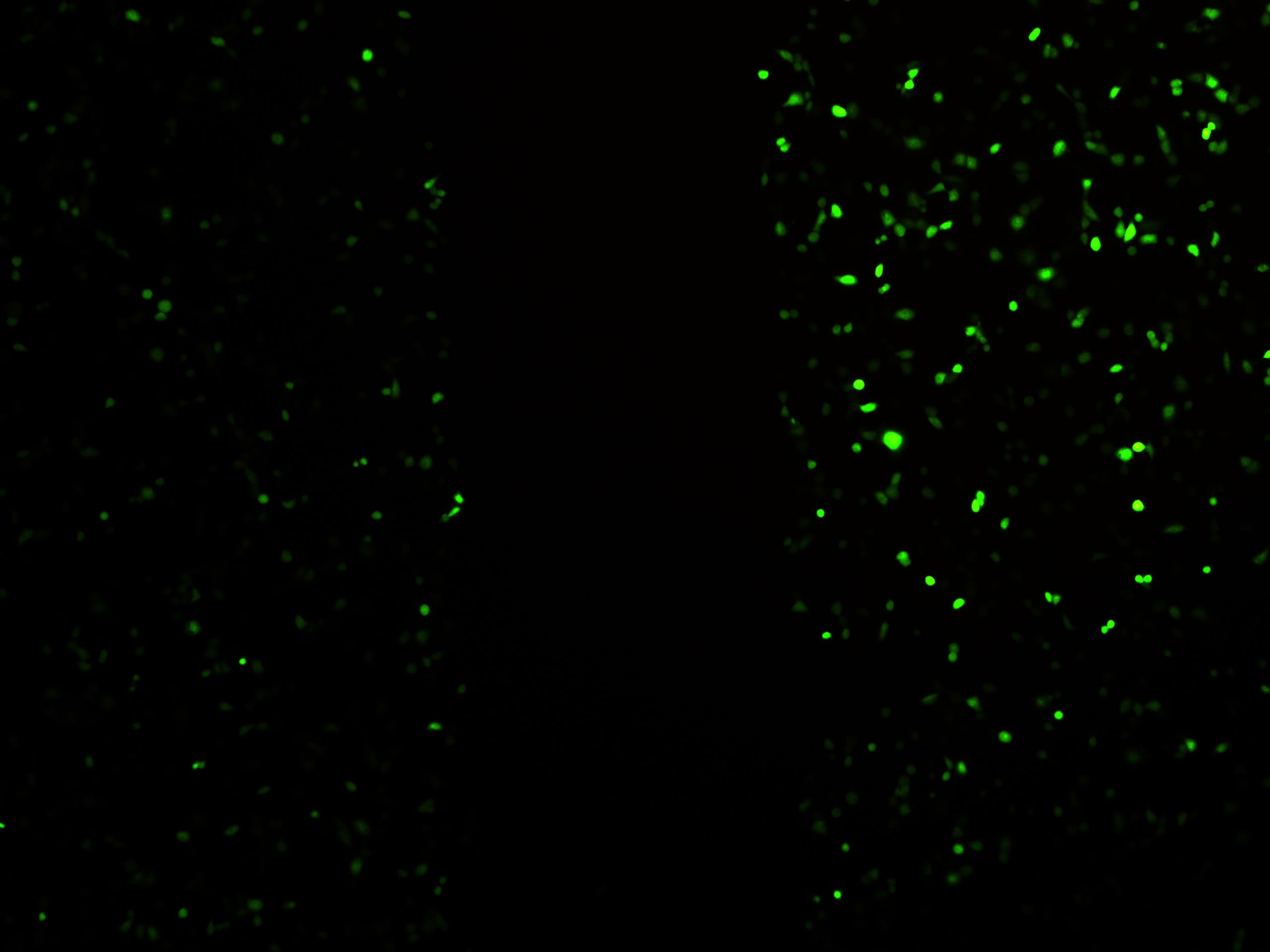

Supplement: Supplementary file 9 [file DataSheet_6.zip › Data Sheet 6/FigS1F/3-SiAC009948.5-0H.jpg]

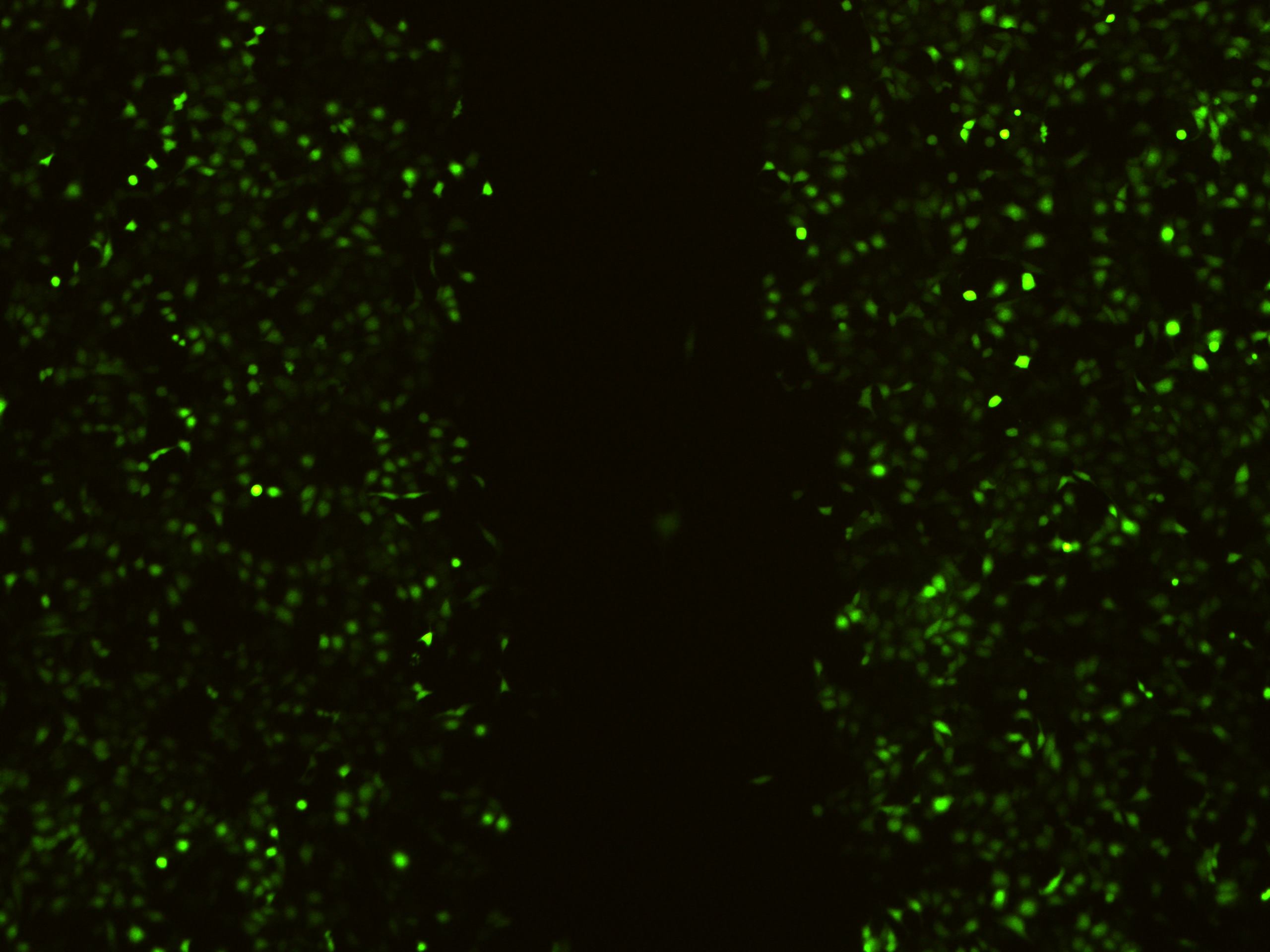

Supplement: Supplementary file 9 [file DataSheet_6.zip › Data Sheet 6/FigS1F/3-SiAC009948.5-24H.jpg]

**Fig4C**

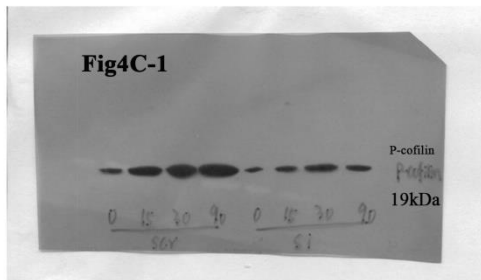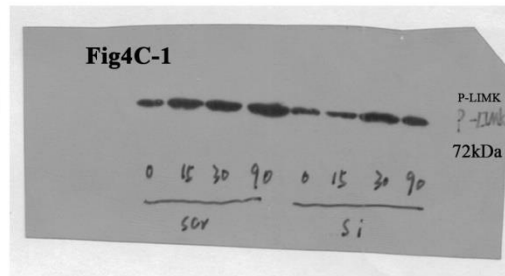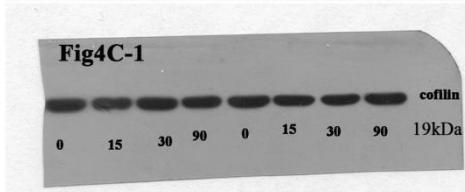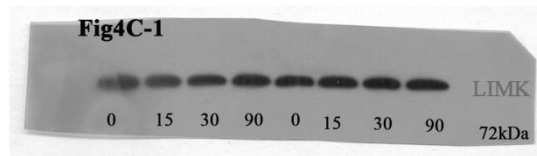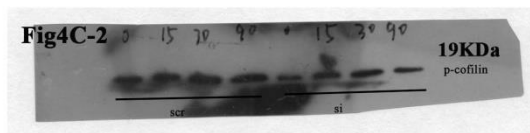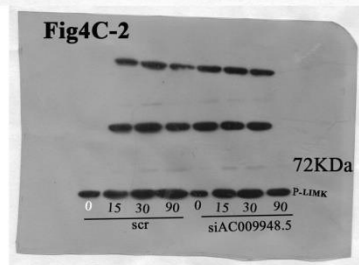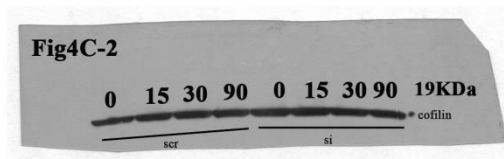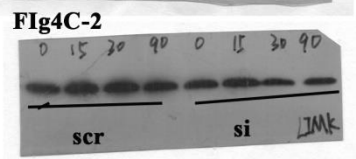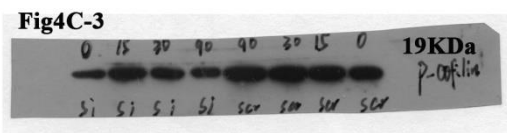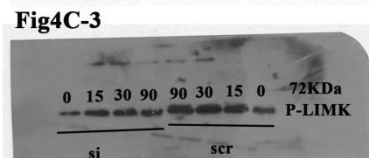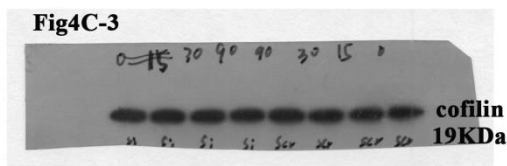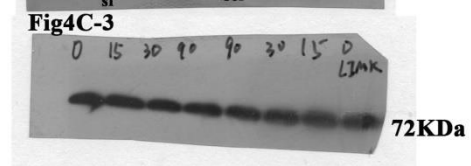

Fig4D

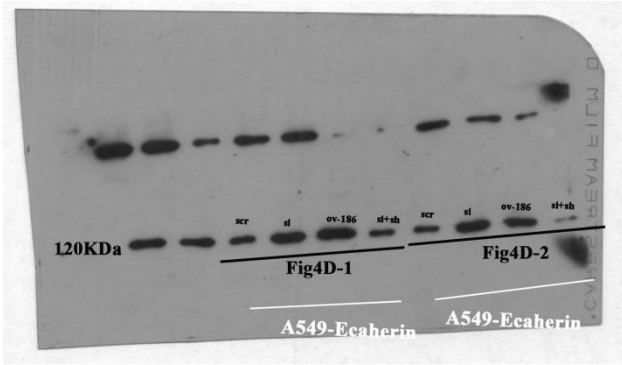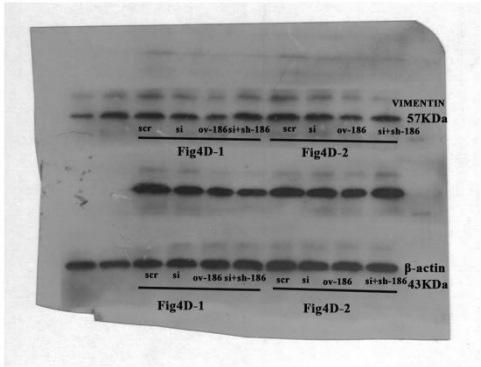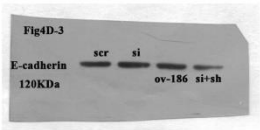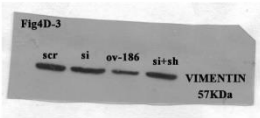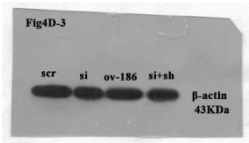

**Fig4E**

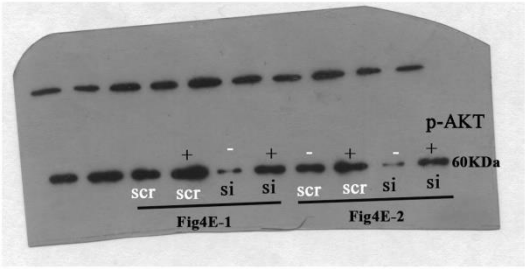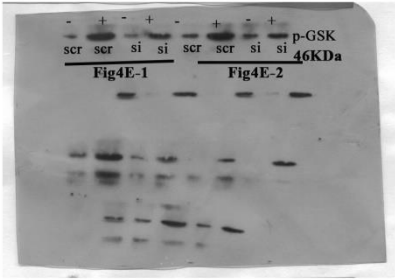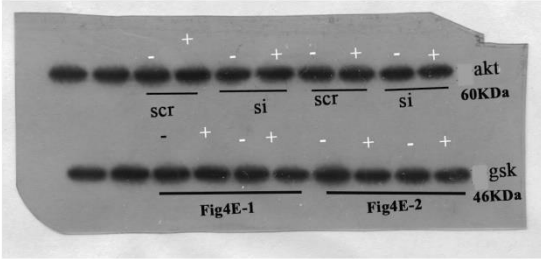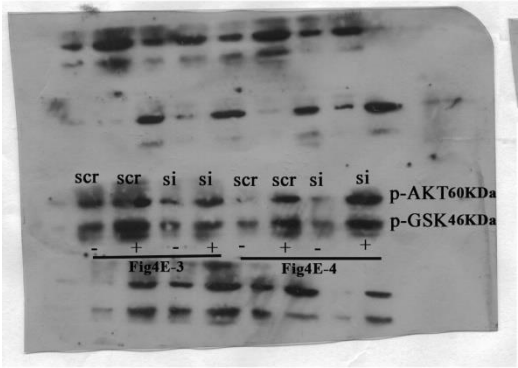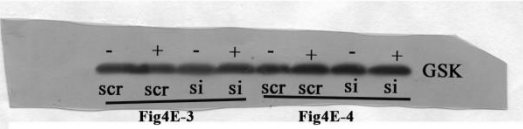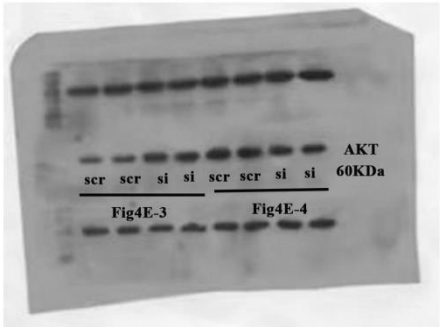

**Fig4F**

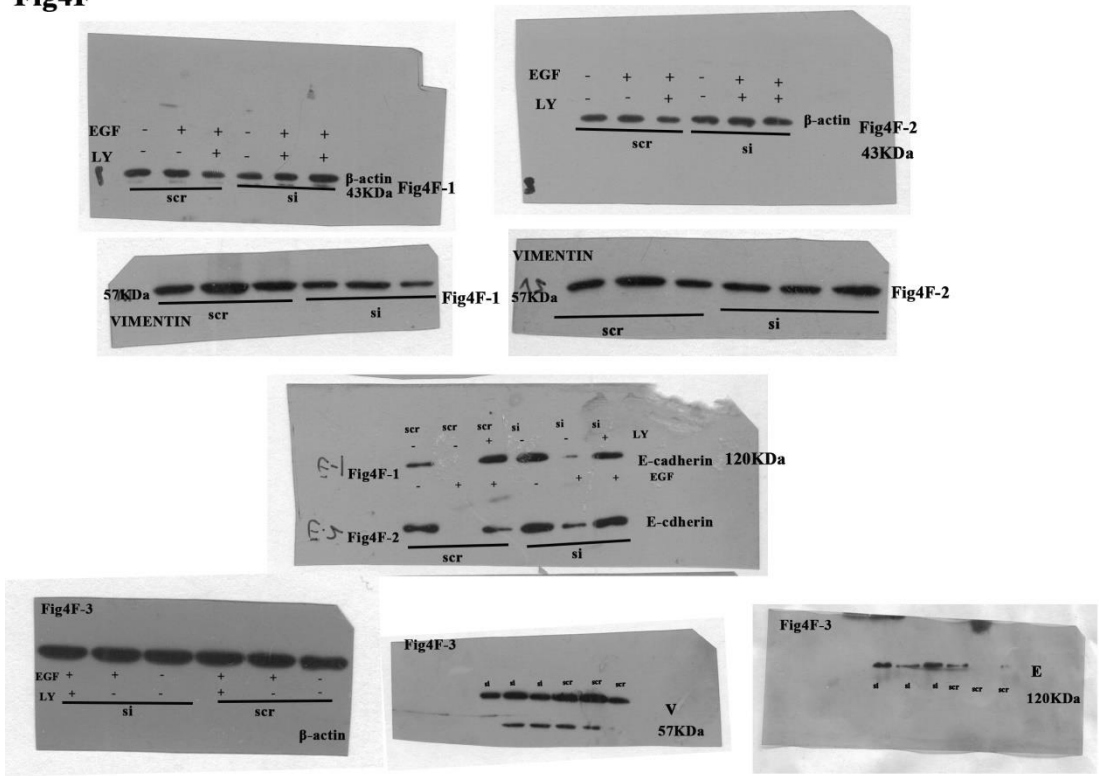

**Fig4G**

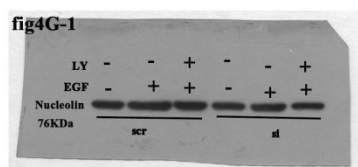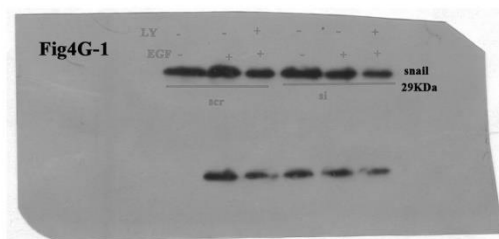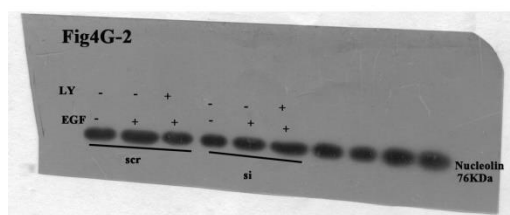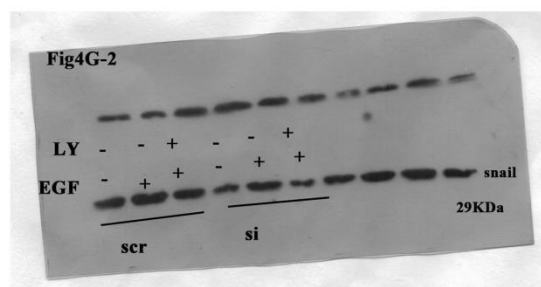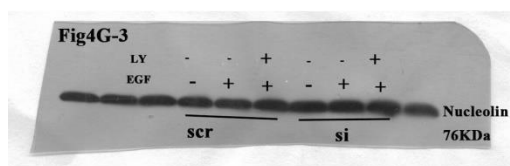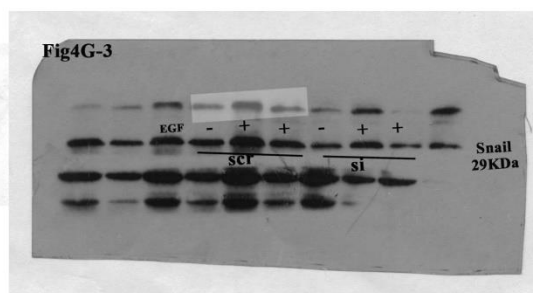

**Fig6C**

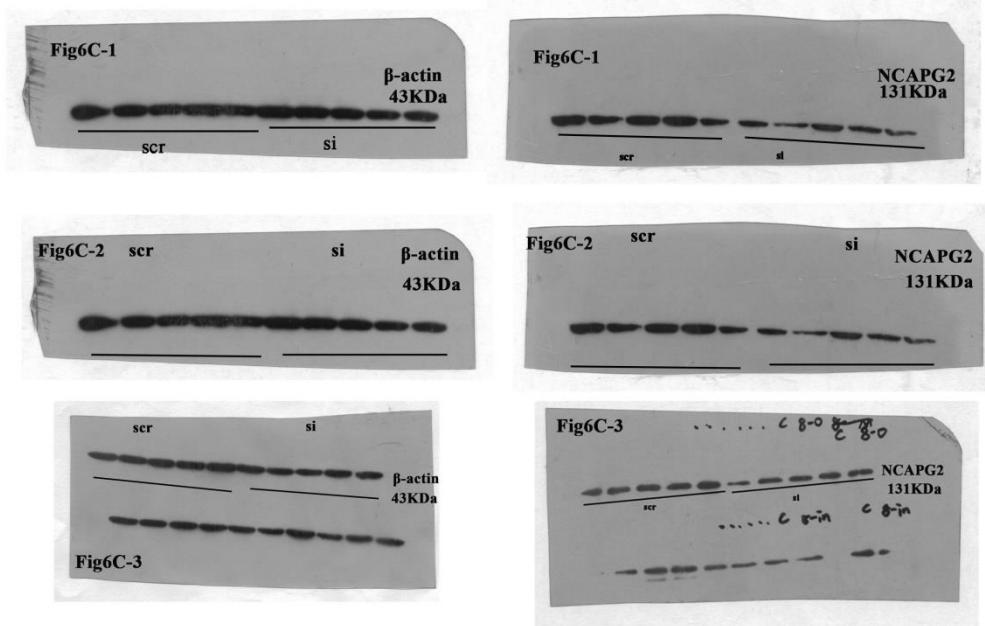

**FigS1B**

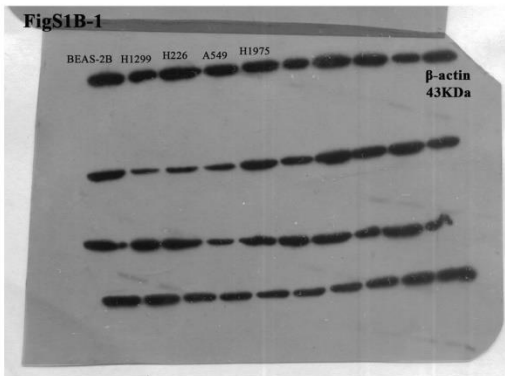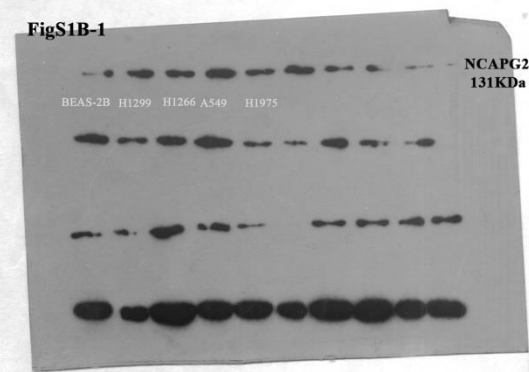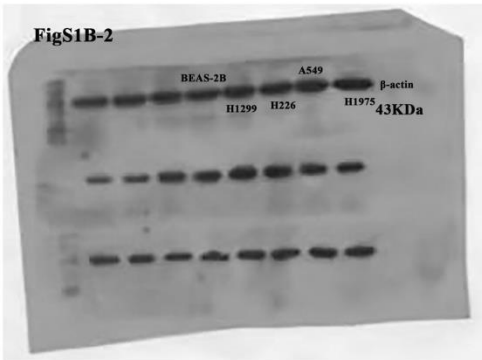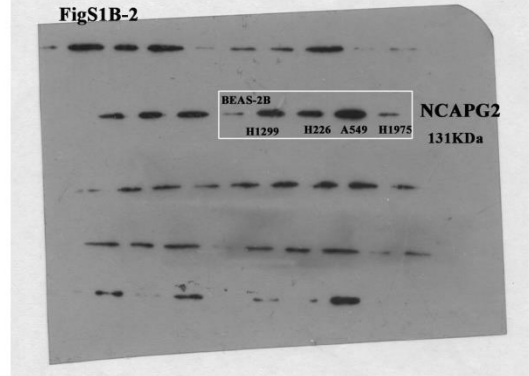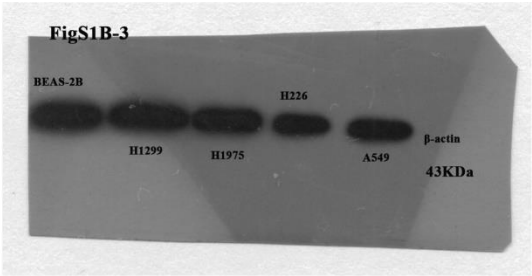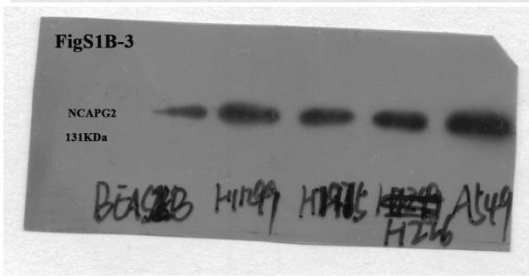

**FigS1C**

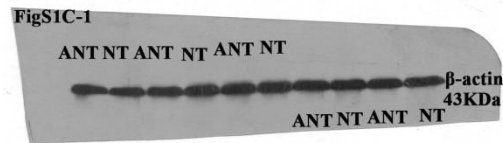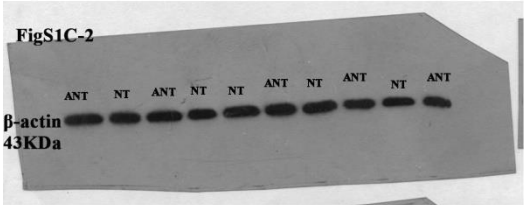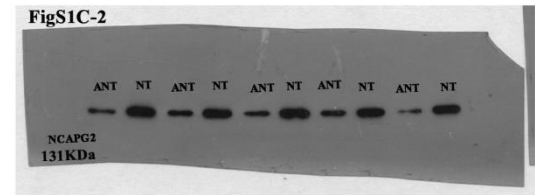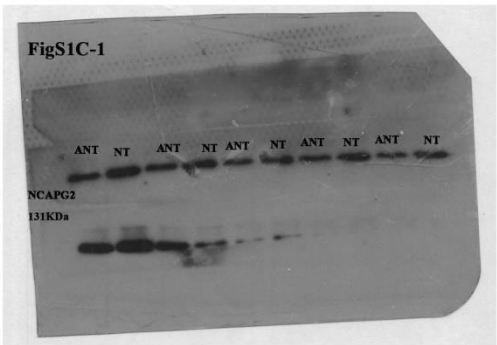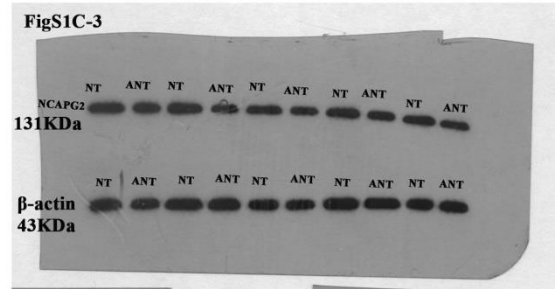

**FigS2E**

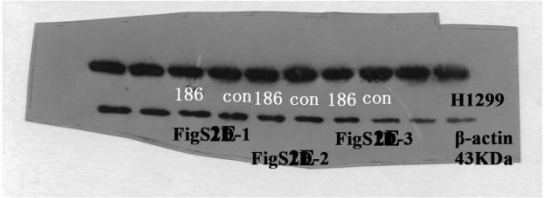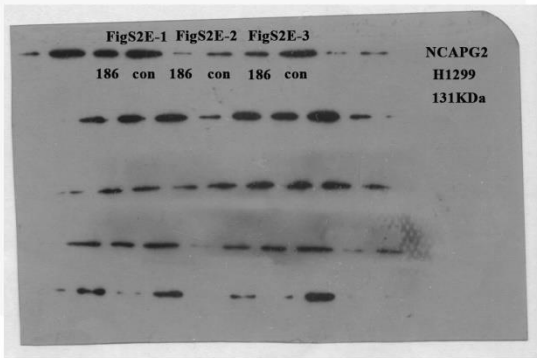

**FigS1E**

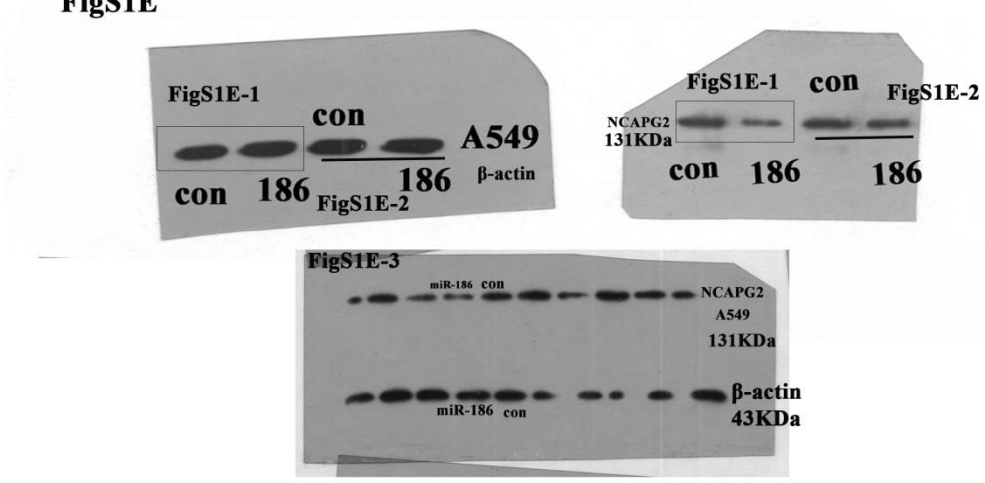

**FigS3A**

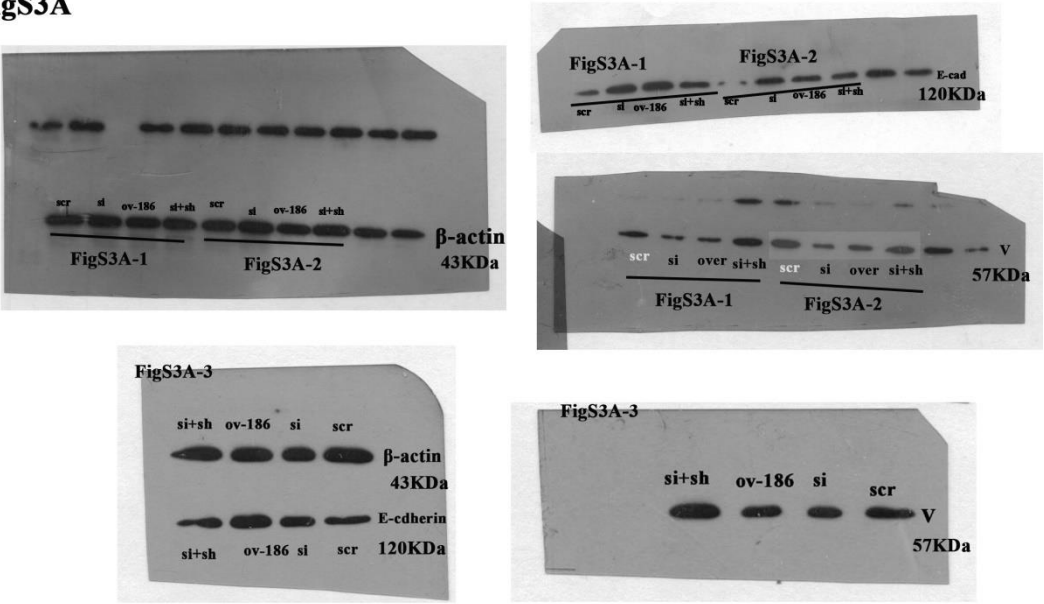

**FigS3B**

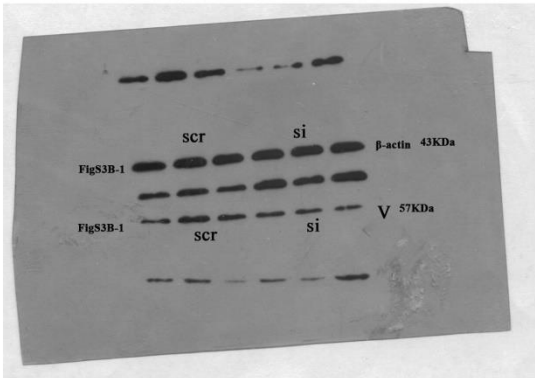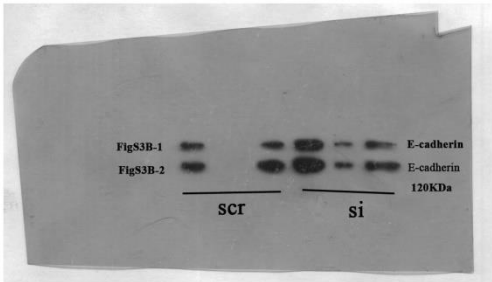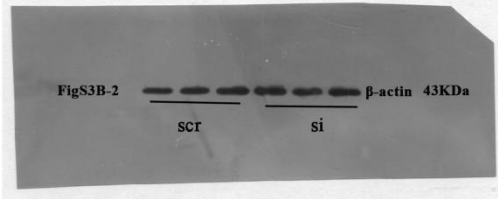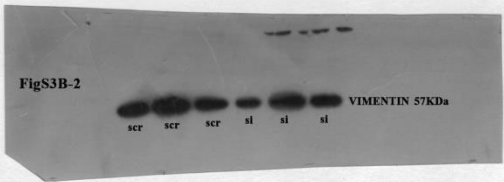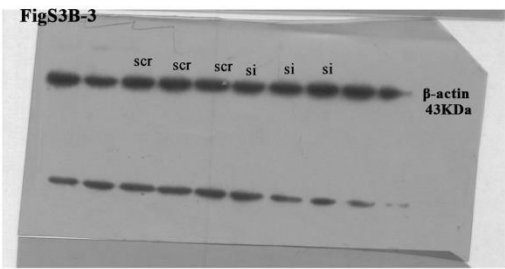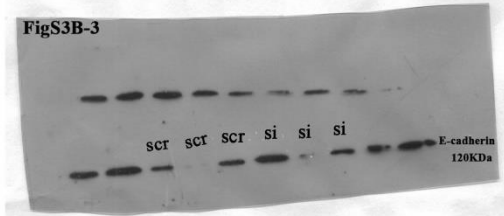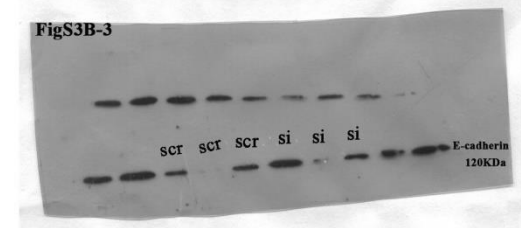

FigS3E

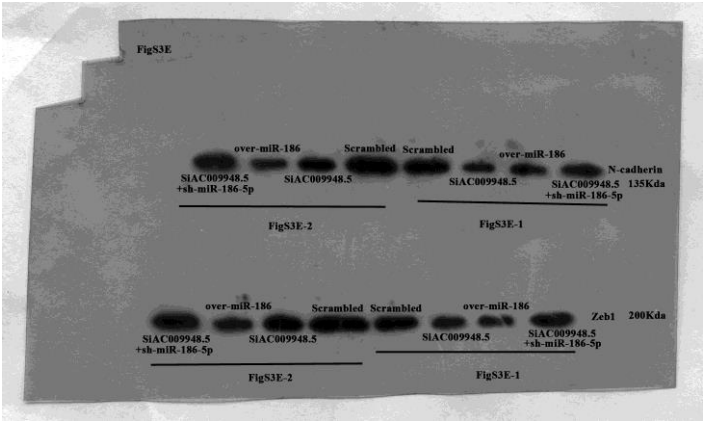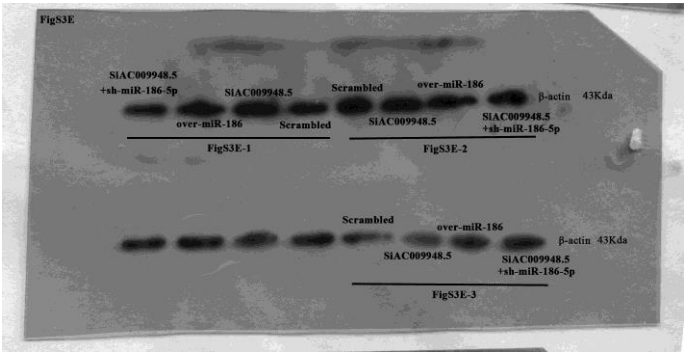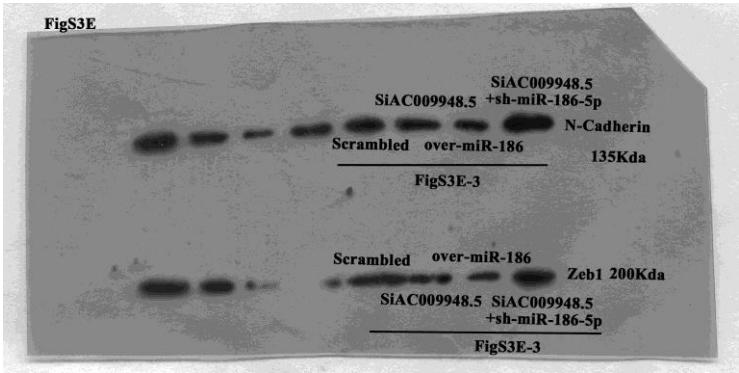

## FigS3F

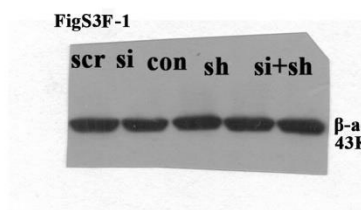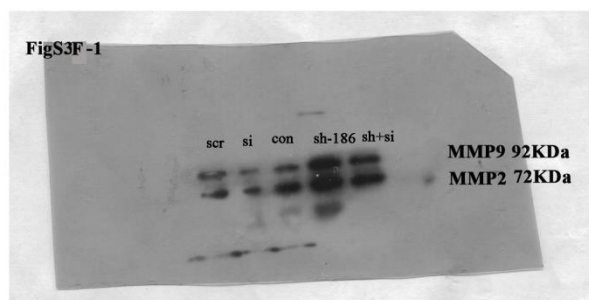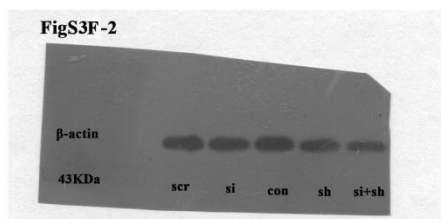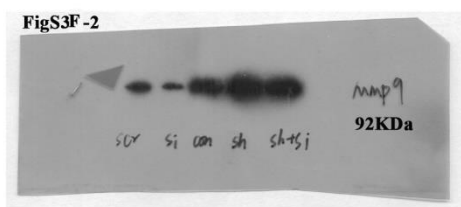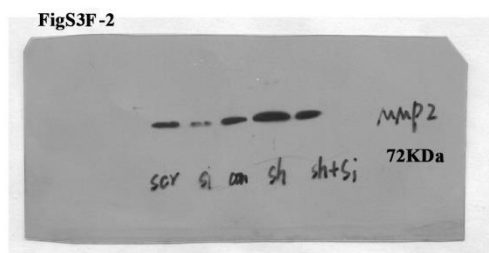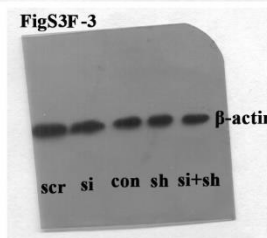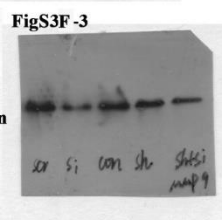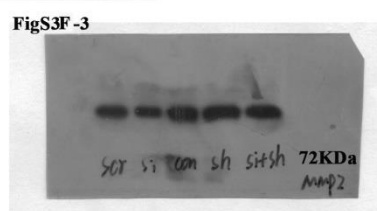

Supplement: Supplementary file 10 [file DataSheet_7.zip › 原始数据WB.pdf]
